# ﻿Catalogue of the Diptera (Insecta) of Morocco— an annotated checklist, with distributions and a bibliography

**DOI:** 10.3897/zookeys.1094.62644

**Published:** 2022-04-12

**Authors:** Kawtar Kettani, Martin J. Ebejer, David M. Ackland, Gerhard Bächli, David Barraclough, Miroslav Barták, Miguel Carles-Tolrá, Milos Černý, Pierfilippo Cerretti, Peter Chandler, Mohamed Dakki, Christophe Daugeron, Herman De Jong, Josef Dils, Henry Disney, Boris Droz, Neal Evenhuis, Paul Gatt, Gustavo Graciolli, Igor Y. Grichanov, Jean-Paul Haenni, Martin Hauser, Oumnia Himmi, Iain MacGowan, Bruno Mathieu, Mohamed Mouna, Lorenzo Munari, Emilia P. Nartshuk, Oleg P. Negrobov, Pjotr Oosterbroek, Thomas Pape, Adrian C. Pont, Grigory V. Popov, Knut Rognes, Marcela Skuhravá, Vaclav Skuhravý, Martin Speight, Guy Tomasovic, Bouchra Trari, Hans-Peter Tschorsnig, Jean-Claude Vala, Michael von Tschirnhaus, Rüdiger Wagner, Daniel Whitmore, Andrzej J. Woźnica, Tadeusz Zatwarnicki, Peter Zwick

**Affiliations:** 1 Laboratory Ecology, Systematics, and Conservation of Biodiversity (LESCB), URL-CNRST N°18, FS, Abdelmalek Essaadi University, Tetouan, Morocco Abdelmalek Essaadi University Tetouan Morocco; 2 National Museum and Galleries of Wales, Entomology Section, Department of Natural Sciences, Cathays Park, Cardiff CF1 3NP, Wales, UK National Museum and Galleries of Wales Cardiff United Kingdom; 3 Oxford University Museum of Natural History, Parks Road, Oxford OX1 3PW, UK Oxford University Museum of Natural History Oxford United Kingdom; 4 Zoological Museum Winterthurerstr. 190. CH-8057 Zürich, Switzerland Zoological Museum Winterthurerstr. Zürich Switzerland; 5 School of Life Sciences, University of Kwa Zulu-Natal, P. Bag X54001, Durban 4000, South Africa University of Kwa Zulu-Natal Durban South Africa; 6 Department of Zoology and Fisheries, Faculty of Agrobiology, Food and Natural Resources, Czech University of Life Sciences Prague, Kamýcká 129, 165 00 Praha-Suchdol, Czech Republic Czech University of Life Sciences Prague Praha-Suchdol Czech Republic; 7 Avda. Riera de Cassoles 30, ático 1. E-08012 Barcelona. Spain Unaffiliated Barcelona Spain; 8 CZ–763 63 Halenkovice 1, Czech Republic Unaffiliated Halenkovice Czech Republic; 9 Dipartimento di Biologia e Biotecnologie “Charles Darwin”, Università di Roma “La Sapienza”, Piazzale, Aldo Moro 5, 00185 Rome, Italy Università di Roma “La Sapienza” Rome Italy; 10 606B Berryfield Lane, Melksham, Wilts SN12 6EL, England, UK Unaffiliated Melksham United Kingdom; 11 Laboratoire de Géo-Biodiversité et Patrimoine naturel, Institut Scientifique, Université Mohammed V de Rabat, Rabat, Morocco Université Mohammed V de Rabat Rabat Morocco; 12 MECADEV, UMR 7179 CNRS/MNHN. Muséum national d’Histoire naturelle, CP 50 – Entomologie, 45 rue Buffon, 75005 Paris, France Muséum national d’Histoire naturelle Paris France; 13 Naturalis Biodiversity Center, Postbus 9517, 2300 RA Leiden, Netherlands Naturalis Biodiversity Center Leiden Netherlands; 14 Krekelberg, 149, 2940 Hoevenen, Belgium Unaffiliated Hoevenen Belgium; 15 Department of Zoology, University of Cambridge, Downing Street, Cambridge CB2 3EJ, England, UK University of Cambridge Cambridge United Kingdom; 16 Ruelle de l’Aurore 7, CH-2300, La Chaux-de-Fonds, Switzerland Unaffiliated La Chaux-de-Fonds Switzerland; 17 Linsley Gressitt Center for Research in Entomology, Bishop Museum, 1525 Bernice Street, Honolulu, Hawaii 96817-2704, USA Bishop Museum Honolulu United States of America; 18 44 Monarch Close, Wickford SS11 8GF, Essex, England, UK Unaffiliated Wickford United Kingdom; 19 Instituto de Biociências, Universidade Federal do Mato Grosso do Sul, Campo Grande, MS, Brasil Universidade Federal do Mato Grosso do Sul Campo Grande Brazil; 20 Institute of Plant Protection, Shosse Podbelskogo 3, VIZR, St. Petersburg-Pushkin 196608, Russia Institute of Plant Protection St. Petersburg-Pushkin Russia; 21 Muséum d’histoire naturelle, Rue des Terreaux 14, CH-2000 Neuchâtel, Switzerland Muséum d’histoire naturelle Neuchâtel Switzerland; 22 Plant Pest Diagnostics Branch, California Department of Food & Agriculture 3294 Meadowview Road, Sacramento, CA 95832-1448, USA California Department of Food & Agriculture Sacramento United States of America; 23 National Museums of Scotland, Collection Centre, 242 West Granton Road, Edinburgh EH5 1JA, Scotland National Museums of Scotland Edinburgh United Kingdom; 24 Institut de Parasitologie et de Pathologie Tropicale, UR7292 Dynamique des interactions hôte pathogène, Université de Strasbourg, 3 rue Koeberlé, 67000 Strasbourg, France Université de Strasbourg Strasbourg France; 25 Entomology Section, Natural History Museum, Fontego dei Turchi, S. Croce 1730, I-30135 Venezia, Italy Natural History Museum Venezia Italy; 26 Zoological Institute of Russian Academy of Sciences, Universitetskaya naberezhnaya 1, 199034, St. Petersburg, Russia Zoological Institute of Russian Academy of Sciences St. Petersburg Russia; 27 Voronezh State University, Universitetskaya sq., 1, 394006 Voronezh, Russia Voronezh State University Voronezh Russia; 28 Natural History Museum of Denmark, Zoological Museum, Universitetsparken 15, DK-2100 Copenhagen, Denmark Natural History Museum of Denmark Copenhagen Denmark; 29 Section of Entomology and Collection Management, I.I.Schmalhausen Institute of Zoology, National Academy of Sciences of Ukraine, Bohdan Khmelnytsky st., 15, 01601 Kyiv, Ukraine I.I.Schmalhausen Institute of Zoology, National Academy of Sciences of Ukraine Kyiv Ukraine; 30 University of Stavanger, Faculty of Arts and Education, Department of Early Childhood Education, NO-4036 Stavanger, Norway University of Stavanger Stavanger Norway; 31 Bítovská 1227, CZ–140 00 Praha 4, Czech Republic Unaffiliated Praha Czech Republic; 32 Department of Zoology, Trinity College, Dublin, Ireland Trinity College Dublin Ireland; 33 Université de Liège, Gembloux Agro-Bio Tech, Unité d’Entomologie fonctionnelle et évolutive (Prof. E. Haubruge), Passage des Déportés, 2, 5030 Gembloux, Belgium Université de Liège Gembloux Belgium; 34 Staatliches Museum für Naturkunde Stuttgart, Rosenstein 1, 70191 Stuttgart, Germany Staatliches Museum für Naturkunde Stuttgart Stuttgart Germany; 35 Résidence Belle Fontaine, Bat B, 16 Avenue de la Trillade, 84000 Avignon, France Unaffiliated Avignon France; 36 Fakultät Biologie, Universität Bielefeld, Postfach 100131, D-33501 Bielefeld, Germany Universität Bielefeld Bielefeld Germany; 37 Limnologische Fluss-Station des Max-Planck Instituts für Limnologie, P.O. Box 260, D-36105 Schlitz, Germany Limnologische Fluss-Station des Max-Planck Instituts für Limnologie Schlitz Germany; 38 Institute of Environmental Biology, Wrocław University of Environmental & Life Sciences, Kożuchowska 5b, 51-631 Wrocław, Poland Wrocław University of Environmental & Life Sciences Wrocław Poland; 39 Institute of Biology, Opole University, ul. Oleska 22, 45-052 Opole, Poland Opole University Opole Poland; 40 Schwarzer Stock 9, 36110 Schlitz, Germany Unaffiliated Schlitz Germany

**Keywords:** Bibliography, classification, gnats, midges, new combination, new records, taxonomy, true flies

## Abstract

The faunistic knowledge of the Diptera of Morocco recorded from 1787 to 2021 is summarized and updated in this first catalogue of Moroccan Diptera species. A total of 3057 species, classified into 948 genera and 93 families (21 Nematocera and 72 Brachycera), are listed. Taxa (superfamily, family, genus and species) have been updated according to current interpretations, based on reviews in the literature, the expertise of authors and contributors, and recently conducted fieldwork. Data to compile this catalogue were primarily gathered from the literature. In total, 1225 references were consulted and some information was also obtained from online databases. Each family was reviewed and the checklist updated by the respective taxon expert(s), including the number of species that can be expected for that family in Morocco. For each valid species, synonyms known to have been used for published records from Morocco are listed under the currently accepted name. Where available, distribution within Morocco is also included. One new combination is proposed: *Assuaniamelanoleuca* (Séguy, 1941), **comb. nov.** (Chloropidae).

## ﻿Foreword

Almost everyone is now aware that there is a biodiversity crisis, and even the popular press regularly reports on general declines in the diversity of species as well as more specific threats to the distribution and abundance of species. Despite this growing awareness of the importance of biodiversity to the health of our planet and thus to the welfare of humankind, relatively little attention has been paid to the glaringly obvious point that it is impossible to objectively assess changes such as declines in diversity in the absence of baseline data and a good taxonomic infrastructure. In other words, if you don’t know what you have to start with it is impossible to know what you are losing (or gaining). Recognition of invasions, extirpations, extinctions, range changes and changes in phenology all pivot on the documentation of what species occur in an area to begin with. Regional catalogues like this one provide both a baseline and a startling wake-up call; in this case with the announcements of 156 previously undocumented species, records of many hitherto unrecorded genera, and the first recognition of half a dozen families new to the country.

Documentation of the dipteran diversity of Morocco is of special significance both in terms of taxon and area. The taxon, Diptera or true flies, is one of the two or three largest orders of living things, with hundreds of thousands of undescribed species worldwide in addition to the approximately 160,000 currently named species. The Moroccan Diptera fauna is relatively well known compared to other parts of Africa, but nonetheless the current catalogue more than doubles the number of species known from the country as of the last inventories a couple of decades ago. This impressive advance in the documentation of Morocco’s significant dipteran fauna is of course in part due to the great dedication and Herculean effort on the part of the lead author, Prof. Kettani. But, it is also a great credit to the community of dipterists who rallied to the cause by meticulously cataloguing their chosen families.

Approximately 33 of the 60 contributors are retired entomologists or amateur dipterists who volunteered their time to contribute to this work. Sadly, a few have passed away since they made their contribution. It is both impressive and worrisome that more than half of those 60 top experts on the taxonomy of the region’s Diptera are retired or amateur, since there is a pressing and ongoing need for specialized expertise on all of the families of Diptera. Hopefully the framework, and the challenge, provided by this thorough description of our current state of knowledge of Moroccan flies will help and encourage new students of dipterology while at the same time providing a foundation for ongoing assessments of the dipteran diversity of North Africa.

I congratulate all involved for the production of such an important contribution to our understanding of the biodiversity of the region, and for the successful completion of the first full Diptera catalogue for any African country.

Stephen A. Marshall

Professor, University of Guelph, Canada

October 2020

## ﻿Contributions

This catalogue was conceived by K. Kettani (KK) in 2011 and then further developed and planned together with Martin J. Ebejer (MJE). These plans included more fieldwork, predominantly by KK and MJE, with some contribution by students, carried out in 2012–2019, which resulted in about 1000 new species records for Morocco and 23 species (in 12 families) new to science. These records were published prior to the end of 2020 and the papers are included in the references and cited under the appropriate families. KK compiled the data and the preliminary list of species for each family. The taxonomic specialists (co-authors and contributors) revised any taxonomic issues, added data available to them and updated nomenclature. Contributors are thanked in the Acknowledgements, as well as mentioned in their sections of the checklist. The distributions of species in Morocco, with the appropriate citations, were added by KK. The introduction was written by KK and supplemented with additional information by MJE, who also checked the English language.

### ﻿Introduction

﻿Aims of the catalogue

Although the national study on biodiversity, *Etude nationale sur la biodiversité* (Dakki 1997; Mouna 1998), was the only important synthesis of entomological studies carried out in Morocco until now, important data on the Diptera are lacking. For example, no taxonomic rank such as subfamily and tribe, and no synonyms or revised generic combinations, names of museums or private collections that house species described from Morocco were included. No information was provided on the habitats and distribution of species in Morocco. Furthermore, the information resulting from the national study on biodiversity is now outdated. A large number of important recent publications, cited appropriately under each family, provide much additional data to the lists published in 1997 and 1998, almost doubling the number of known species. The main aims of this catalogue are to bring together all this information by providing an up-to-date checklist according to the current nomenclature and systematics, to provide synonyms used in older literature that dealt with the Moroccan fauna and to give regional distribution data. Finally, an essential comprehensive bibliography is provided.

This catalogue is the first for a North African country and it may prove useful also for neighbouring countries. It is hoped that it will provide a sound foundation for anyone choosing to study a group of Moroccan Diptera. It could indicate where the greatest need for investigation lies, both with regard to areas of the country that have been poorly studied and taxonomic groups (or families) that may benefit from more attention. It is undoubtedly not a complete inventory and many more discoveries are expected.

The faunistic knowledge of the Diptera of Morocco from 1787 to 2021 is summarized and updated, providing the first catalogue of the Moroccan species of Diptera. In total, 3057 species (975 Nematocera and 2082 Brachycera) in 93 families, classified into 949 genera (250 in Nematocera and 699 in Brachycera) are listed (Tables 1, 2).

**Table 1.** Families of Nematocera with contributing specialists and numbers of taxa.

Family	Specialist(s)	Subfamilies	Genera	Species
Anisopodidae	Haenni	1	1	3
Bibionidae	Haenni	1	2	10
Blephariceridae	Zwick	1	1	4
Cecidomyiidae	Skuhravá & Skuhravý	3	31	57
Ceratopogonidae	Mathieu	3	6	62
Chaoboridae	Wagner	1	2	2
Chironomidae	-	7	100	412
Culicidae	Trari, Himmi & Dakki	2	7	43
Dixidae	Wagner	-	2	12
Keroplatidae	Chandler	1	2	2
Limoniidae	Oosterbroek	4	22	67
Mycetophilidae	Chandler	5	25	64
Pediciidae	Oosterbroek	1	2	6
Psychodidae	Wagner	2	12	51
Ptychopteridae	Wagner	1	1	5
Scatopsidae	Haenni	3	11	13
Sciaridae	Heller	-	12	70
Simuliidae	-	1	6	43
Thaumaleidae	Wagner	-	1	2
Tipulidae	Oosterbroek & de Jong	2	3	39
Trichoceridae	Krzemińska	1	1	8
			**Total: 250**	**Total: 975**

**Table 2.** Families of Brachycera with contributing specialists and numbers of taxa.

Family	Specialist(s)	Subfamilies	Genera	Species
Acroceridae	Nartshuk	2	6	13
Agromyzidae	Černý	2	12	62
Anthomyiidae	Ackland	2	14	36
Anthomyzidae	Ebejer	-	2	2
Asilidae	Tomasovic	9	42	131
Asteiidae	Ebejer	1	2	5
Atelestidae	Gatt	-	1	1
Athericidae	Mouna	1	1	2
Aulacigastridae	Ebejer	-	1	1
Bombyliidae	Ebejer & Dils	11	48	248
Braulidae	-	1	1	1
Calliphoridae	Rognes	5	5	8
Camillidae	Ebejer	-	1	4
Canacidae	Munari	2	3	15
Carnidae	Ebejer	-	1	3
Chamaemyiidae	Ebejer	2	5	18
Chloropidae	von Tschirnhaus & Ebejer	3	34	74
Chyromyidae	Ebejer	2	3	22
Clusiidae	Ebejer	-	1	1
Coelopidae	-	1	1	1
Conopidae	-	5	10	34
Cryptochetidae	Nartshuk	-	1	1
Diastatidae	Ebejer	2	2	2
Dolichopodidae	Grichanov & Negrobov	12	36	112
Drosophilidae	Bächli	2	9	26
Dryomyzidae	-	1	1	1
Empididae	Daugeron	3	8	40
Ephydridae	Zatwarnicki	5	43	117
Fanniidae	Pont	-	1	10
Helcomyzidae	-	1	1	1
Heleomyzidae	Woźnica	3	5	19
Hippoboscidae	Droz	3	10	17
Hybotidae	Gatt	2	10	44
Lauxaniidae	Ebejer	2	8	27
Lonchaeidae	MacGowan	1	4	5
Lonchopteridae	Barták	1	1	4
Micropezidae	Ebejer	-	1	1
Milichiidae	-	3	6	8
Muscidae	Pont	6	25	115
Mydidae	Dikow	3	4	9
Mythicomyiidae	Evenhuis	4	6	8
Nemestrinidae	Barraclough	2	4	13
Nycteribiidae	Graciolli	1	4	8
Odiniidae	Ebejer	1	1	2
Oestridae	Pape	2	5	10
Opomyzidae	-	-	2	5
Pallopteridae	Ebejer	-	1	1
Phoridae	Disney	-	2	3
Piophilidae	Ebejer	1	3	3
Pipunculidae	Ebejer	2	7	16
Platypezidae	Ebejer	1	3	3
Platystomatidae	Popov	1	2	4
Polleniidae	Rognes	-	1	12
Psilidae	Ebejer	-	1	1
Rhagionidae	Ebejer	1	1	4
Rhiniidae	Rognes	1	5	17
Rhinophoridae	Pape	1	6	8
Sarcophagidae	Whitmore & Pape	3	16	66
Scathophagidae	-	1	2	3
Scenopinidae	Carles-Tolrá	1	2	11
Sciomyzidae	Vala	1	12	25
Sepsidae	Haenni	1	4	12
Sphaeroceridae	Gatt	3	29	67
Stratiomyidae	Woodley	5	11	40
Streblidae	Graciolli	1	2	2
Syrphidae	Speight	2	50	166
Tabanidae	-	3	11	69
Tachinidae	Cerretti & Tschorsnig	4	88	147
Tephritidae	Norrbom	3	29	69
Therevidae	Hauser	2	9	27
Ulidiidae	Ebejer	2	8	13
Vermileonidae	Ebejer	1	2	6
			**Total: 698**	**Total: 2082**

### ﻿Brief history of Moroccan dipterology

The study of Diptera in Morocco is still far from being extensive despite the numerous investigations that have taken place so far. Until now, a synthesis of Moroccan Diptera had never been produced. The studies that have been devoted to this group of insects have been limited and sporadic. They were mainly concerned with either taxonomy and descriptions of new species, or faunal studies from specific areas of Morocco with little reference to the habitats of species. This was particularly so for early works such as Becker and Stein (1913), Séguy (1930a, 1934b, 1935a, 1941a, 1941d, 1949a, 1953a), Timon-David (1951), Vaillant (1956b) and, recently, Pârvu et al. (2006), Popescu-Mirceni (2011), and Ebejer et al. (2019). Very few recent works have provided relevant ecological data (Cassar et al. 2005, 2008). Becker and Stein (1913) were the first to publish a list of Moroccan Diptera, at that time comprising only 204 species. Séguy followed with a long series of valuable publications. In 1930 he produced his first list of Diptera, which included 471 species in 230 genera grouped into 37 families (Séguy 1930). Over the subsequent years, he contributed greatly to the knowledge of the Diptera of Morocco (Séguy 1926, 1934b, 1935a, 1941a, 1941d, 1949a, 1953a, 1957, 1961), where he also referenced an extensive bibliography. Later, Bailly-Choumara (1977) compiled a bibliography of Moroccan Diptera. It took until 1997–1998, with a national study on the biodiversity of Morocco co-ordinated by what was then the Ministry of the Environment, to update the list of the Moroccan insect fauna. This national study was dedicated to the compilation of the results of entomological research conducted in Morocco in order to highlight Moroccan biodiversity. It included a comprehensive inventory of published entomological research conducted in Morocco and was the last work to include a broad overview of Moroccan Diptera. It listed 623 species of aquatic Diptera in 210 genera and 25 families (Dakki 1997) and 928 species of terrestrial Diptera in 350 genera and 57 families (Mouna 1998).

### ﻿Terrain and bioclimatic regions

**Figure 1.**
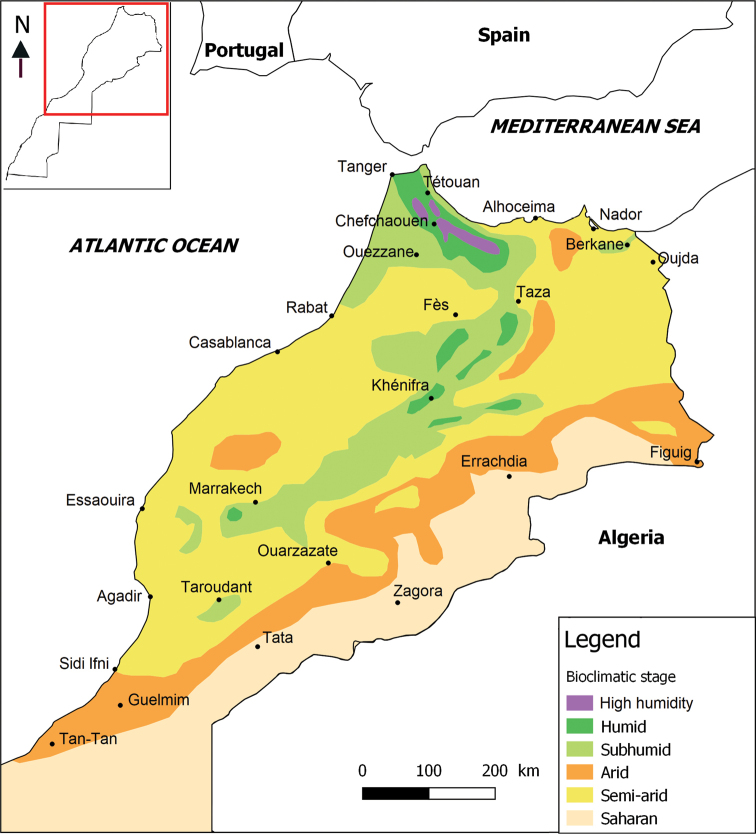
The major bioclimatic regions of the northern part of Morocco.

Morocco, located in the westernmost corner of North Africa, constitutes a biogeographical crossroads between the Afrotropical and the western Palaearctic faunas, which may have allowed some exchange of genetic material. The country has a remarkable variety of bioclimates, ranging from humid in the Rif and the Middle and High Atlas to very arid in the Sahara in the south and to the sub-humid and semi-arid plains and foothills (Fig. 1). As a result of this diversity of landscapes and climates, there exists a great biological and ecological diversity in the country.

**Figure 2.**
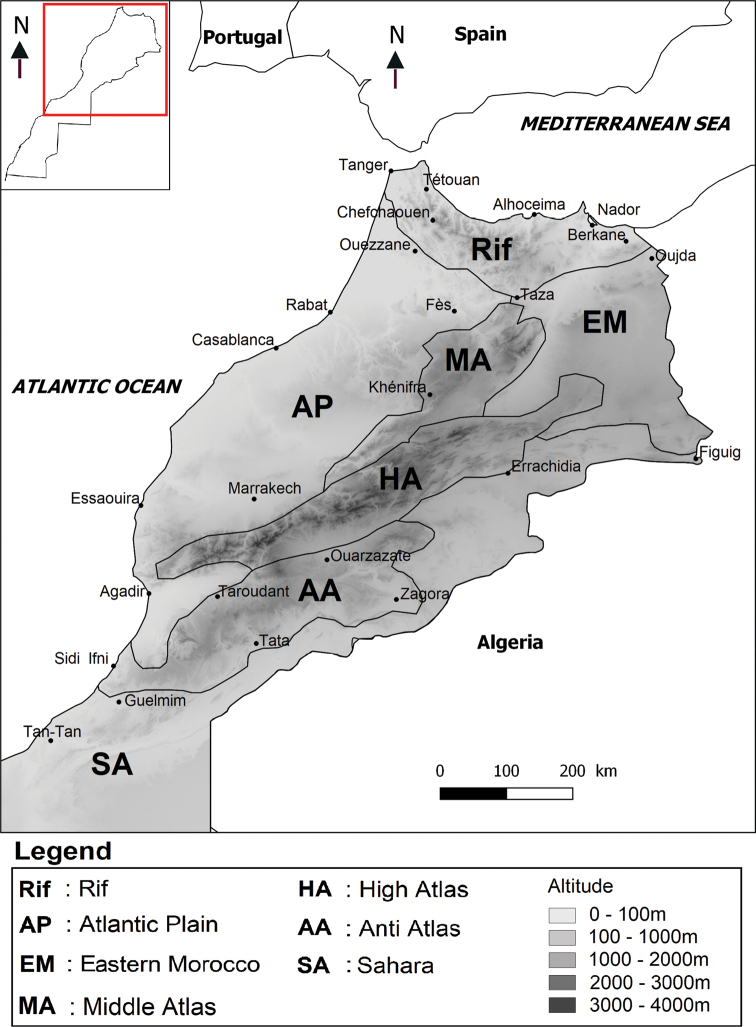
The seven biogeographical regions covering the studied area in the northern part of Morocco.

The geomorphological, orographic and bioclimatic diversity of the country is reflected in its seven distinct biogeographical areas: the Rif, the Atlantic Plain, Eastern Morocco, the Middle Atlas, the High Atlas, the Anti-Atlas and the Sahara. In the checklist, an abbreviated distribution (in bold) is given for each species according to these seven biogeographical areas (Fig. 2).

**Rif**: The Rif consists of a mountainous domain overlooking the Mediterranean Sea in the north of Morocco. It belongs to the Gibraltar Arc or Alborán Sea geological region, part of the Alpine orogenic belt, where the maximum altitude is 2,452 m in the Jbel Tidirrhine mountain range. The climate is mainly of the Mediterranean type and is characterised by high levels of precipitation. This domain is considered very rich and diverse in vegetation cover (Valdés et al. 2002), including nearly all of the Moroccan forest plant species (Médail and Quézel 1999) and integrating five bioclimatic stages.

**Atlantic Plain (AP)**: Also known as the Moroccan Central Plateau, the Atlantic Plain is an ancient massif located in the northwest of Morocco, between the Atlantic coast and the Middle Atlas Mountains. It consists of a plateau surrounded by medium-altitude mountain massifs and covers an area of 8500 km². With an asymmetrical shape, the plateau culminates in the south-east at Jebel Mtourzgene (1627 m), located near the town of Oulmès. Its complex geomorphology presents tectonic troughs that have favoured the formation of lakes and rivers. The annual rainfall of this region, concentrated in winter and spring, totals an average of 500 mm, allowing the establishment of Holm Oak and Cork Oak forests in a semi-arid bioclimate (Piqué 1994). Towards the coast, the vegetation is mainly a steppe with Jujube shrubs and Dwarf Palms (﻿*Chamaerops ﻿﻿﻿humilis*). The maritime fringe is underlined by cliffs and ancient consolidated dunes.

**Eastern Morocco (EM)**: This is an arid region covering the Hauts Plateaux to the northeast of the country. It is the only region of Morocco subject to a true Mediterranean bioclimate, with some influence from the Sahara. The annual rainfall is less than 300 mm, encouraging the dominance of esparto steppe (*Stipatenacissima*) (Piqué 1994). Its geomorphology is represented by a plateau at an altitude of 1000 m, which gradually decreases from 1500 to 500 m from the city of Midelt to the province of Guercif and is criss-crossed by the Moulouya wadi. In the northwestern part of Eastern Morocco is the Béni Snassen massif, beyond the Guercif depression.

**Middle Atlas (MA)**: A mountainous massif located in the centre of Morocco, oriented from south-west to north-east and extending over 350 km (Piqué 1994). This mass of highlands is essentially composed of limestone layers, tabular and of medium altitude in the southwest (tabular Middle Atlas) and undulating in the northeast (folded Middle Atlas) to culminate at 3340 m at Jebel Bou Naceur, which overlooks the high plateaux of Eastern Morocco. The climate, generally subhumid to humid, is cold in winter, giving rise to cedar and Holm Oak forests covering the western slope, which is wetter than the eastern slope and where lakes are abundant and many rivers and streams have their source.

**High Atlas (HA)**: The main mountainous massif of Morocco, oriented from west-south-west to east-north-east, is the High Atlas, stretching over more than 700 km from the Atlantic to the east of Morocco, with a north-south width of 50 to 100 km and reaching at Jebel Toubkal an altitude of 4165 m. The whole massif is high, incised by deep valleys that lead to passes through which the mountainous barrier is crossed: the Tizi-n’Test (2100 m) towards the Souss plain and the Tizi-n’Tichka (2200 m) towards the depression of Ouarzazate. The chain is formed by a massif of Palaeozoic rocks surrounded by sedimentary deposits. The relief of the High Atlas is divided into three different entities from west to east: the Western High Atlas, which is the oldest massif and is made up mainly of Jurassic or Cretaceous formations with deep valleys and culminates at Jebel Toubkal; the central High Atlas, which is an essentially limestone massif morphologically dominated by tabular zones culminating at an altitude of 2500 m, stretches between the towns of Azilal and Ouarzazate and is sheltered by the Jebel M’Goum (4071 m); and the Eastern High Atlas, which is formed by the vast high plateaux of the Upper Moulouya, stretches between the cities of Midelt and Imilchil and contains the Jebel Ayachi (3747 m) (Piqué 1994). The bioclimate of this area includes subhumid, humid and semi-arid zones, but with more than 800 mm of annual precipitation on the highest peaks and a significant snow cover from September to May, with temperatures dropping below -20 °C (AEFCS 1996) and bright sunshine even in winter. These climatic conditions have favoured large pine, Holm Oak, *Thuja* and thuriferous juniper forests (HCEFLCD 2017). The aridity of the southern slope of the High Atlas allows only steppe vegetation of sagebrush and esparto grass to grow.

**Anti-Atlas (AA)**: This is the pre-Saharan area, which extends north of the Wadi Draa and rises to an average altitude of 2000 m. It is a semi-arid region with Jebel Bani and Jebel Ouarkziz as its main elevations, and is home to numerous oases. The western part of the Anti-Atlas is characterised by the Saharan plateau, with a temperate winter except in the high mountains. On the other hand, in the eastern part of the Anti-Atlas, the greatest arid area is marked by the northward extension of the Saharan landscape and a bioclimate of cool and temperate winters. These bioclimatic stages are characterised by plant communities such as wooded steppes with *Acaciaraddiana*, *Artemisiaherba alba* and *Stipatenuissima*, interspersed with thuriferous juniper (*Juniperusthurifera*) at the top of the slopes (Piqué 1994). The vegetation is also represented by two formations that partially interpenetrate each other: groves of the Argan tree (*Arganiaspinosa*) and the *Euphorbia* steppe (*Euphorbiaechinus*).

**Sahara (SA)**: This area in the south of the country consists of a wide arid zone with a typical Saharan bioclimate of high daytime temperatures and very low precipitation. From a geological point of view it is part of the West African Craton and its margins. It is mainly ancient terrain with Precambrian basement rock and Palaeozoic cover (Piqué 1994). This area is represented by a peneplain of very modest altitudes from 200 to 500 m, established on ancient crystalline terrain. Precipitation from July to September is very low, of the order of 100 mm.

The dipteran species richness of each biogeographical region of Morocco is summarised in Table 3, which highlights the great disparity between the different regions and reflects the lack of knowledge about this insect order in some areas of Morocco. The Rif region in the north of the country has the greatest recorded diversity, but this undoubtedly reflects a greater sampling effort in this area, with large parts of the country still very poorly explored for Diptera. The catalogue does not include any data from the southern part of the Moroccan Sahara.

**Table 3.** Species richness of Diptera families each biogeographical region in Morocco. **Rif**; **AP** – Atlantic Plain; **EM** – Eastern Morocco; **MA** – Middle Atlas; **HA** – High Atlas; **AA** – Anti-Atlas; **SA** – Sahara. * No precise localities known.

	Rif	AP	EM	MA	HA	AA	SA
Acroceridae	7	1	0	1	3	0	0
Agromyzidae	7	15	0	10	22	10	0
Anisopodidae *							
Anthomyiidae	4	12	0	13	2	4	1
Anthomyzidae	1	0	0	0	1	0	0
Asilidae	34	27	4	35	25	24	5
Asteiidae	0	3	0	0	1	1	0
Atelestidae	1	0	0	0	0	0	0
Athericidae	0	0	0	1	2	0	0
Aulacigastridae	1	0	0	0	0	0	0
Bibionidae	4	2	0	3	1	2	2
Blephariceridae	1	0	0	0	3	0	0
Bombyliidae	35	80	12	59	48	56	19
Braulidae *							
Calliphoridae	3	8	2	4	4	0	0
Camillidae	1	2	0	0	1	0	0
Canacidae	6	10	0	0	0	6	0
Carnidae	1	0	0	0	0	0	0
Cecidomyiidae	8	27	21	25	20	7	4
Ceratopogonidae	15	43	14	35	33	24	36
Chamaemyiidae	5	5	0	4	8	6	0
Chaoboridae *							
Chironomidae	230	30	7	105	120	14	1
Chloropidae	35	22	0	7	6	25	1
Chyromyidae	6	6	0	0	1	12	1
Clusiidae	1	0	0	0	0	0	0
Coelopidae	1	0	0	0	0	0	0
Conopidae	7	13	0	16	7	9	0
Cryptochetidae	0	0	0	1	0	0	0
Culicidae	32	41	24	30	29	22	11
Diastatidae	0	1	0	0	1	0	0
Dixidae	2	2	0	2	5	0	0
Dolichopodidae	72	28	3	6	18	26	0
Drosophilidae	11	14	0	8	12	5	1
Dryomyzidae	1	0	0	0	0	0	0
Empididae	4	3	0	13	20	1	0
Ephydridae	47	44	1	60	9	20	1
Fanniidae	4	2	0	2	5	0	0
Helcomyzidae	1	0	0	0	0	0	0
Heleomyzidae	11	3	0	2	2	3	0
Hippoboscidae	0	2	3	4	4	4	1
Hybotidae	20	7	3	8	7	8	0
Keroplatidae	1	0	0	0	0	0	0
Lauxaniidae	23	3	2	1	8	2	0
Limoniidae	43	9	4	5	34	5	0
Lonchaeidae	3	3	0	1	1	0	0
Lonchopteridae	0	0	0	1	1	0	0
Micropezidae	1	0	0	0	0	0	0
Milichiidae	2	1	0	0	4	3	1
Muscidae	25	47	13	38	68	20	6
Mycetophilidae	60	3	7	3	0	0	0
Mydidae	0	3	2	1	0	3	1
Mythicomyiidae	2	0	0	0	1	2	1
Nemestrinidae	1	1	1	5	3	0	1
Nycteribiidae	6	5	0	6	0	2	1
Odiniidae	1	0	0	0	0	0	0
Oestridae	0	2	1	1	1	0	1
Opomyzidae	1	1	0	1	2	0	0
Pallopteridae	0	0	0	1	0	0	0
Pediciidae	3	1	0	0	0	0	0
Phoridae	0	2	0	0	0	0	0
Piophilidae	2	1	0	1	1	0	0
Pipunculidae	13	1	0	0	0	0	0
Platypezidae	2	1	0	0	0	0	0
Platystomatidae	0	2	1	1	0	0	0
Polleniidae	1	4	2	5	5	1	1
Psilidae	1	0	0	1	0	0	0
Psychodidae	25	12	12	12	34	9	2
Ptychopteridae *							
Rhagionidae	2	0	0	0	2	0	0
Rhiniidae	2	5	3	5	0	5	4
Rhinophoridae	6	2	0	1	0	0	0
Sarcophagidae	11	19	1	13	8	9	2
Scathophagidae	3	1	0	1	0	0	0
Scatopsidae	10	2	0	0	1	3	0
Scenopinidae	3	4	1	1	0	0	1
Sciaridae	29	12	2	8	45	9	3
Sciomyzidae	8	4	2	11	18	3	0
Sepsidae	3	6	0	4	7	3	0
Simuliidae	32	8	0	19	32	6	0
Sphaeroceridae	42	3	2	10	1	3	3
Stratiomyidae	20	13	5	13	6	1	0
Streblidae	1	1	0	0	0	1	0
Syrphidae	70	49	11	82	64	25	4
Tabanidae	26	27	11	35	21	7	1
Tachinidae	18	27	4	46	43	29	3
Tephritidae	34	13	4	16	13	23	3
Thaumaleidae	0	0	0	0	2	0	0
Therevidae	9	7	2	9	4	5	2
Tipulidae	28	7	0	16	18	2	0
Trichoceridae	7	1	3	1	0	0	0
Ulidiidae	6	2	0	1	4	4	1
Vermileonidae	1	2	1	1	4	0	0
**Total species**	**1126**	**770**	**191**	**831**	**876**	**474**	**126**

### ﻿Sources of data

Data for the present study were gathered from the literature, supplemented by information taken from a number of websites. The cut-off date for inclusion of species published in the literature is 30 June 2021. Taxonomic, nomenclatural, and distributional data for the present catalogue were obtained from:

- studies by various foreign and Moroccan researchers published between 1787 and 2021, including theses, books, catalogues, checklists and notes from the 1997–1998 synthesis studies on Morocco’s biodiversity.
- data from websites and organisations such as Global Species, the Global Biodiversity Information Facility, the BioSystematic Database of World Diptera, Systema Dipterorum, Catalogue of Life, Systema Naturae 2000 and others, or from relevant homepages specific to particular families.
- the collection of Diptera in the National Museum of Morocco (Muséum National d’Histoire Naturelle à l’Institut Scientifique, Université Mohammed V, Rabat).
- collaboration with specialists who communicated reliable identifications from their personal collections or provided data for species deposited in foreign museums.
- published records of specimens in the personal collection of the lead author (PCKK), deposited at Abdelmalek Essaadi University, Tetouan, identified with the assistance of specialists.

### ﻿Classification

The arrangement of families in this catalogue (Table 4) is mainly based on Pape et al. (2011) and Wiegmann et al. (2011). All families are listed alphabetically within each superfamily. Nomenclature follows the most recent version of Systema Dipterorum (Evenhuis and Pape 2021) and the Polleniidae are listed as a separate family (Gisondi et al. 2020).

**Table 4.** Diptera classification used in the catalogue.

Suborder NEMATOCERA	
Tipuloidea	Limoniidae
	Pediciidae
	Tipulidae
Trichoceridoidea	Trichoceridae
Psychodoidea	Psychodidae
Scatopsoidea	Scatopsidae
	Ptychopteridae
Culicoidea	Chaoboridae
	Culicidae
	Dixidae
Chironomoidea	Ceratopogonidae
	Chironomidae
	Simuliidae
	Thaumaleidae
	Blephariceridae
Bibionoidea	Anisopodidae
	Bibionidae
Sciaroidea	Cecidomyiidae
	Keroplatidae
	Mycetophilidae
	Sciaridae
Suborder BRACHYCERA	
Stratiomyoidea	Stratiomyidae
Tabanoidea	Athericidae
	Rhagionidae
	Tabanidae
	Vermileonidae
Nemestrinoidea	Acroceridae
	Nemestrinidae
Asiloidea	Asilidae
	Bombyliidae
	Mydidae
	Mythicomyiidae
	Scenopinidae
	Therevidae
Empidoidea	Atelestidae
	Empididae
	Dolichopodidae
	Hybotidae
Platypezoidea	Phoridae
	Platypezidae
	Lonchopteridae
Syrphoidea	Pipunculidae
	Syrphidae
Conopoidea	Conopidae
Nerioidea	Micropezidae
Tanypezoidea	Psilidae
Tephritoidea	Lonchaeidae
	Pallopteridae
	Piophilidae
	Platystomatidae
	Tephritidae
	Ulidiidae
Lauxanioidea	Chamaemyiidae
	Lauxaniidae
Sciomyzoidea	Coelopidae
	Dryomyzidae
	Helcomyzidae
	Sciomyzidae
	Sepsidae
Opomyzoidea	Agromyzidae
	Anthomyzidae
	Asteiidae
	Aulacigastridae
	Clusiidae
	Odiniidae
	Opomyzidae
Carnoidea	Canacidae
	Carnidae
	Chloropidae
	Milichiidae
Sphaeroceroidea	Chyromyidae
	Heleomyzidae
	Sphaeroceridae
Ephydroidea	Braulidae
	Camillidae
	Cryptochetidae
	Diastatidae
	Drosophilidae
	Ephydridae
Hippoboscoidea	Hippoboscidae
	Nycteribiidae
	Streblidae
Muscoidea	Anthomyiidae
	Fanniidae
Muscoidea	Muscidae
	Scathophagidae
Oestroidea	Calliphoridae
	Oestridae
	Polleniidae
	Rhiniidae
	Rhinophoridae
	Sarcophagidae
	Tachinidae

## ﻿Arrangement of the catalogue

The catalogue includes all names of taxonomically valid species of the Order Diptera so far known to occur in Morocco. For each family, synonyms and alternative combinations that have been published as records of Moroccan provenance are listed under the currently accepted name.

The transliteration of Moroccan place names in the literature, maps, and sometimes on data labels presented a small problem. Although it would have been preferable to use standard names, this would have meant changing some place names from their original spellings in the literature. Most transliteration used the French language for spelling and Roman letters, but not all. English was sometimes used, and occasionally also Spanish. Readers are therefore advised to take this into account when searching for place names given in the catalogue and in the cited references. Some common examples are “Jebel”, “Jbel”, “Djebel” or “Dj.”, used for “mountain”, or “Kbir” and “Kebir”, for “large” or “great”. The article “the” in Arabic is El or L; it is sometimes separated by a hyphen from its associated noun or joined to it, sometimes capitalised while the noun is not, both are capitalised, or only the noun is. When the article “el” is followed by a noun beginning with a certain consonant, like n, r, s and t, the l is changed to match the noun’s initial, for example “Er-Rifiyine”. In transliteration, Arabic speakers would make this change, whereas non-Arabic speakers may not. In cases where the article is joined to the noun, the resulting versions may appear as being different place names; for example, El Rachidia, Er Rachidia and Errachidia are alternative transliterations of the same place name. In some entries, the French terms “affluent” and “aval affluent” are left as in the original citation, because “tributary” may not be an accurate translation in all instances. For some of the sites, the French term refers to a downstream overflow of water into a side tributary or waterfall, whereas for others it refers to an actual tributary feeding into a main river or stream.

Older authors were imprecise with localities, and many references did not give the coordinates of localities from which species were collected. We could not reliably provide accurate coordinates for each species. Where known, species depositories are included. Individual family checklists may include, at the end, taxonomic notes and comments on species doubtfully present in the country. New records are indicated with an asterisk (*) and the relevant data listed at the end of the family section.

### ﻿Limitations

One of the major difficulties encountered while compiling the catalogue was the limited ability to check old identifications. In some cases, identifications were accepted as given in the original sources, even when doubtful, as it was usually not possible to re-examine specimens. The inaccessibility of some references was also an obstacle to updating the checklists for some families.

### ﻿Collections cited

The following museums and collections house some of the specimens mentioned in this catalogue. The National Museum of Natural History of Morocco is located in the Institut Scientifique, Université Mohammed V, Rabat. However, the Diptera collection housed in that museum is not yet included in any world database such as the GBIF Registry of Scientific Collections, https://www.gbif.org/grscicoll, or the Insect and Spider collections of the World Website, http://hbs.bishopmuseum.org/codens/.

**HNHM**Hungarian Natural History Museum, Budapest, Hungary;

**INHS**Illinois Natural History Survey, Urbana, USA;

**IRSNB**Institut Royal des Sciences Naturelles de Belgique, Brussels, Belgium;

**MfN**Museum für Naturkunde, Berlin, Germany;

**MHNN**Muséum d’Histoire Naturelle de Neuchâtel, Neuchâtel, Switzerland;

**MISR** Museum National d’Histoire Naturelle à l’Institut Scientifique, Université Mohammed V, Rabat, Morocco;

**MNCN**Museo Nacional de Ciencias Naturales, Madrid, Spain;

**MNHN**Muséum National d’Histoire Naturelle, Paris, France;

**NHMD** Natural History Museum of Denmark, Copenhagen, Denmark;

**NHMUK**Natural History Museum, London, United Kingdom;

**NMWC**National Museum of Wales, Cardiff, United Kingdom;

**OUMNH**Oxford University Museum of Natural History, Oxford, United Kingdom;

**RMNH** Rijksmuseum voor Natuurlijke Historie, Leiden, The Netherlands;

**SMNS**Staatliches Museum für Naturkunde Stuttgart, Stuttgart, Germany;

**ZMUM** Zoological Museum of Moscow University, Moscow, Russia;

**ZSM**Zoologische Staatssammlung, Munich, Germany;

**ZSM** Zoologische Staatssammlung, Munich, Germany.

The personal collection of Dr Michael von Tschirnhaus has been donated to the ZSM, but is currently on loan to Dr von Tschirnhaus.

#### Other abbreviations

**HT** Holotype;

**NPH** National Park of Al Hoceima;

**NPHAO** National Park of Haut Atlas Oriental;

**NPI** National Park of Ifrane;

**NPT**National Park of Talassemtane;

**PCJD** Private Collection of Jos Dils, Belgium;

**PCKK** Private Collection of Kawtar Kettani, Morocco;

**PNPB** Project of Natural Park of Bouhachem.

## ﻿Acknowledgements

The authors are deeply grateful to the reviewers for their constructive comments, which helped to improve the manuscript. Our sincere thanks are expressed to the subject editor, Torsten Dikow, for his significant contribution and support as editor.

We are most grateful to the following specialists who generously contributed in various ways to this catalogue by sharing information and knowledge of their group or by reviewing a family section: Patrick Ashe (Dublin, Ireland)—Chironomidae; Torsten Dikow (Washington, D.C., USA)—Mydidae; the late Amnon Freidberg (Tel Aviv, Israel)—Tephritidae; Kai Heller (Heikendorf, Germany)—Sciaridae; Gail Kampmeier (Champaign, USA)—Therevidae; Valery Korneyev (Kiev, Ukraine)—Tephritidae; Ewa Krzemińska (Kraków, Poland)—Trichoceridae; Allen Norrbom (Washington, D.C., USA)—Tephritidae; Adrian R. Plant (Mahasarakham, Thailand)—Empididae, Hemerodrominae; Bradley J. Sinclair (Ottawa, Canada)—Empididae, Clinocerinae; Jens-Hermann Stuke (Leer, Germany)—Conopidae; Norman Woodley (Hereford, AZ, USA)—Stratiomyidae. Mohamed Mouna and Mohamed Dakki are acknowledged and thanked for their encouragement and inspiration that led the first author to commence this project, as well as for generously donating to her a large bibliography on the subject. Mohamed Mouna was instrumental in ensuring that she had full access to the collections and library and full cooperation from the staff of these departments. He is immensely thanked by the first author for donating to her his library, consisting of many books and publications.

Mohammed Fekhaoui, Abdellatif Bayed, Mohamed-Aziz El Agbani, Mohamed Arahou from the administration of Museum National d’Histoire Naturelle à l’Institut Scientifique, Université Mohammed V, Rabat are warmly thanked for their valuable assistance in providing access to the library and Diptera collections of the museum. Thanks also to Amina Zouida, the librarian at the said institute, for her tireless and precious help in searching for obscure publications, and to Abderrahmane Mataame (responsible for the zoological collections of the museum) for his kind and patient assistance during the first author’s frequent visits to the museum. Yassine Fekrani is acknowledged for his kind help producing the maps.

## ﻿Suborder NEMATOCERA

### ﻿Tipuloidea

#### ﻿﻿LIMONIIDAE

K. Kettani, P. Oosterbroek

Number of species: **67**. Expected: 85

Faunistic knowledge of the family in Morocco: moderate

##### Chioneinae

﻿***Baeoura* Alexander, 1924**

﻿*Baeoura﻿ebenina* Starý, 1981

Driauach and Belqat 2015, **Rif**, Oued Tazarine (Mezine village); Dayat near Aïn Afersiw; Driauach and Belqat 2016; Oosterbroek 2020 (CCW: **Rif**)

﻿*Baeoura﻿staryi* Driauach & Belqat, 2015

Driauach and Belqat 2015, **Rif**, Jnane Niche; Driauach and Belqat 2016; Oosterbroek 2020 (CCW: **Rif**)

﻿***Cheilotrichia* Rossi, 1848**

﻿*Cheilotrichia (Empeda) ﻿cinerascens* (Meigen, 1804)

Driauach et al. 2013, **Rif**, Ikadjiouen; Driauach and Belqat 2016, **Rif**, Oued Ouara, Oued Amsa, Oued Maggou, Aïn Quanquben (Jebel Bou Bessoui), **EM**, Grotte du Chameau (Zegzel, Béni Snassen); Oosterbroek 2020 (CCW: **Rif**; **EM**)

﻿*Cheilotrichia (Empeda) ﻿fuscohalterata* (Strobl, 1906)

Driauach and Belqat 2016, **Rif**, Dayat Fifi, tributary of Oued El Fondak, Barrage Ajras, Dayat Mghara, Oued Zarka, Oued Tizekhte; Oosterbroek 2020 (CCW: **Rif**)

﻿*Cheilotrichia (Empeda) ﻿minima* (Strobl, 1898)

Driauach and Belqat 2016, **Rif**, Oued Zendoula, Oued Jnane Niche; Oosterbroek 2020 (CCW: **Rif**)

﻿***Ellipteroides* Becker, 1907**

﻿*Ellipteroides (Ellipteroides) lateralis* (Macquart, 1835)

= ﻿*Gonomyialateralis* Macquart, in Pierre 1922a: 22, Dakki 1997: 62

= ﻿*Gonomyia﻿cincta* Egger, in Séguy 1941a: 26

Pierre 1922a, **AP**, Dradek (near Rabat); Lackschewitz 1940a, **HA**, Tachdirt (2200–2900 m); Séguy 1941a, **HA**, Tachdirt (Toubkal, 2500 m); Savchenko et al. 1992; Dakki 1997; Starý and Freidberg 2007; Driauach et al. 2013; Driauach and Belqat 2016, **Rif**, Dayat Afrate; Oosterbroek 2020 (CCW: **Rif**)

﻿*Ellipteroides (Protogonomyia) ﻿alboscutellatus* (von Roser, 1840)

Lachschewitz 1940a, **HA**; Savchenko et al. 1992; Starý and Oosterbroek 2008; Starý 2009a; Driauach et al. 2013; Driauach and Belqat 2016; Oosterbroek 2020 (CCW)

﻿*Ellipteroides (Protogonomyia) ﻿hutsoni* (Starý, 1971)

Starý 1971, **HA**, Jebel Ayachi; Savchenko et al. 1992, **HA**, Jebel Ayachi; Driauach et al. 2013; Driauach and Belqat 2016; Oosterbroek 2020 (CCW)

﻿***Erioconopa* Starý, 1976**

﻿*Erioconopa﻿diuturna* (Walker, 1848)

Driauach et al. 2013; Driauach and Belqat 2016, **Rif**, Dayat Jebel Zemzem, Oued Mezine, Aïn Bab Tariouant; Oosterbroek 2020 (CCW: **Rif**)

﻿*Erioconopa﻿symplectoides* (Kuntze, 1914)

= ﻿*Dactylolabis﻿symplectoides* Egger, in Pierre 1922a: 23

Pierre 1922a, **HA**, Marrakech; Savchenko et al. 1992; Starý and Oosterbroek 2008, **HA**; Gavryushin pers. comm. 2012, **HA**; Driauach et al. 2013; Driauach and Belqat 2016; Oosterbroek 2020 (CCW: **HA**)

﻿***Erioptera* Meigen, 1803**

﻿*Erioptera (Erioptera) ﻿fuscipennis* Meigen, 1818

Pierre 1922a, **AP**, Casablanca (Oued Guerera); Pierre 1922b, **HA**, Savchenko et al. 1992; Dakki 1997; Driauach et al. 2013, **Rif**, Sidi Brahim Ben Arrif (Bab Hachef Aissa); Driauach and Belqat 2016, **Rif**, Aïn El Ma Bared, Oued Amsemlil, Oued Maggou (Bridge), Oued Tkarae, Dayat near Aïn Afersiw, Aïn Afersiw, Oued Abou Bnar, Oued Maggou (Zaouiet El Habtiyne), Dayat Amsemlil, Dayat Aïn Jdi­oui; Dayat Afrate, Oued Mezine; Oosterbroek 2020 (CCW: **Rif**, **HA**)

﻿*Erioptera (Erioptera) lutea lutea* Meigen, 1804

Driauach and Belqat 2016, **Rif**, Aïn Boughaba; Oosterbroek 2020 (CCW: **Rif**)

﻿*Erioptera (Erioptera) ﻿transmarina* Bergroth, 1889

= *Mesocyphona ﻿﻿﻿transmarina* Bergroth, in Pierre 1922a: 22, Pierre 1922b: 148, Dakki 1997: 62

Pierre 1922a, **HA**, Marrakech; Pierre 1922b, **HA**, Tannaout (1000 m), Marrakech; Dakki 1997; Savchenko et al. 1992; Driauach et al. 2013; Driauach and Belqat 2016; Oosterbroek 2020 (CCW)

﻿***Gonomyia* Meigen, 1818**

﻿*Gonomyia (Gonomyia) ﻿abscondita* Lackschewitz, 1935

Driauach and Belqat 2016, **Rif**, maison forestière; Oosterbroek 2020 (CCW: **Rif**)

﻿*Gonomyia (Gonomyia) ﻿sicula* Lackschewitz, 1940

Driauach and Belqat 2016, **Rif**, Oued Kbir, Dayat Jebel Zemzem, Aïn Sidi Brahim Ben Arrif, Dayat Tazia, Aïn El Maounzil; Oosterbroek 2020 (CCW: **Rif**)

﻿*Gonomyia (Gonomyia) ﻿subtenella* Savchenko, 1972

Starý and Oosterbroek 2008, **HA**, Massif Toubkal, Aghbalou, 43 km S Marrakech (1000 m); Starý 2009a; Driauach et al. 2013; Driauach and Belqat 2016, **Rif**, Oued Jnane Niche, Oued Sidi Yahia Aârab, **EM**, Oued Béni Ouachekradi; Oosterbroek 2020 (CCW: **Rif**, **EM**)

﻿*Gonomyia (Gonomyia) ﻿tenella* (Meigen, 1818)

Pierre 1924a, **HA**, Asni (1200 m); Séguy 1930a, **HA**, Asni; Savchenko et al. 1992; Dakki 1997; Starý 2009a; Driauach et al. 2013; Driauach and Belqat 2016; Oosterbroek 2020 (CCW)

﻿***Hoplolabis* Osten-Sacken, 1869**

﻿*Hoplolabis (Parilisia) ﻿obtusiapex* (Savchenko, 1982)

Starý 2006, **HA**, Oasis Meski, **AA**; Driauach et al. 2013; Driauach and Belqat 2016; Oosterbroek 2020 (CCW)

﻿*Hoplolabis (Parilisia) punctigera* (Lackschewitz, 1940)

Starý 2006, **HA**, Oasis Meski, **AA**; Koçak and Kemal 2010; Driauach et al. 2013; Driauach and Belqat 2016; Oosterbroek 2020 (CCW)

﻿*Hoplolabis (Parilisia) ﻿sororcula* (Lackschewitz, 1940)

Driauach et al. 2013; Driauach and Belqat 2016, **Rif**, Barrage Ajras, Oued Ouringa, Oued El Kanar, Oued Maggou; Oosterbroek 2020 (CCW: **Rif**)

﻿***Idiocera* Dale, 1842**

﻿*Idiocera (Euptilostena) ﻿jucunda* (Loew, 1873)

= ﻿*Gonomyia﻿jucunda* Loew, in Pierre 1924a: 201, Séguy 1930a: 22

Pierre 1924a, **HA**, Asni (1200 m); Séguy 1930a, **HA**, Asni; Savchenko et al. 1992; Dakki 1997; Driauach et al. 2013; Driauach and Belqat 2016; Oosterbroek 2020 (CCW)

﻿*Idiocera (Idiocera) ﻿ampullifera* (Starý, 1979)

Driauach and Belqat 2016, **AA**, Oued Zag; Oosterbroek 2020 (CCW: **AA**)

﻿*Idiocera (Idiocera) ﻿pulchripennis* (Loew, 1856)

= ﻿*Gonomyia﻿sexpunctata* Dale, in Pierre 1922b: 148

= ﻿*Gonomyia﻿pulchripennis* (Loew), in Dakki 1997: 62

Pierre 1922b, **AP**, Atlantic coast; Ramdani 1981, **AP**, Merja Sidi Boughaba; Savchenko et al. 1992; Dakki 1997; Koçak and Kemal 2010; Driauach et al. 2013; Driauach and Belqat 2016, **Rif**, Oued Jnane Niche, Oued Aârkoub, Aïn Jdioui; Oosterbroek 2020 (CCW)

﻿*Idiocera (Idiocera) ﻿sziladyi* (Lackschewitz, 1940)

Driauach and Belqat 2016, **Rif**, Oued Zarka; Oosterbroek 2020 (CCW: **Rif**)

﻿***Ilisia* Rondani, 1856**

﻿*Ilisia﻿maculata* (Meigen, 1804)

Driauach and Belqat 2016, **Rif**, Oued Maggou, **EM**, Oued Tafoughalt; Oosterbroek 2020 (CCW: **Rif**; **EM**)

﻿***Molophilus* Curtis, 1833**

﻿*Molophilus (Molophilus) ﻿ibericus* Starý, 2011

Starý 2011, **HA**; Driauach et al. 2013; Driauach and Belqat 2016; Oosterbroek 2020 (CCW)

﻿*Molophilus (Molophilus) ﻿obscurus* (Meigen, 1818)

Starý and Oosterbroek 2008, **HA**, Massif Toubkal, Oukaimeden, 2500–2800 m; Driauach et al. 2013; Driauach and Belqat 2016, **Rif**, tributary of Oued Ouara, tributary of Oued Maggou, Dayat El Ânassar, Dayat Jebel Zemzem, Aïn El Ma Bared, Dayat Rmali, Oued Amsemlil, Aïn Mâaze, Dayat Afrate, Dayat Lemtahane; Oosterbroek 2020 (CCW: **Rif**)

﻿*Molophilus (Molophilus) ﻿propinquus ﻿propinquus* (Egger, 1863)

Savchenko et al. 1992; Starý and Oosterbroek 2008, **HA**, Oukaimeden (2500–2800 m); Starý 2011; Driauach et al. 2013; Driauach and Belqat 2016, **Rif**, Oued Ouara, Oued Farda, tributary of Oued Taida, Oued Maggou; Oosterbroek 2020 (CCW: **Rif**)

﻿*Molophilus (Molophilus) ﻿testaceus* Lackschewitz, 1940

Driauach and Belqat 2016, **Rif**, Dayat Amsemlil, Dayat Lemtahane, Marj El kheyl, Oued Tkarae, Dayat near Aïn Afersiw, Dayat Mezine, Dayat Tazia, Dayat Rmali, Dayat Amsemlil, Dayat near Aïn Afersiw; Oosterbroek 2020 (CCW: **Rif**)

﻿***Symplecta* Meigen, 1830**

﻿*Symplecta (Symplecta) ﻿grata* Loew, 1873

Ebejer et al. 2020, **Rif**, Aïn Jdioui (Tahaddart, 8 m)

﻿*Symplecta (Symplecta) ﻿hybrida* (Meigen, 1804)

Lackshewitz 1940a, **HA**, Goundafa (1200 m); Séguy 1941a, **HA**, Taroudant; Savchenko et al. 1992; Oosterbroek et al. 2007; Koçak and Kemal 2010; Driauach et al. 2013; Driauach and Belqat 2016, **Rif**, Barrage Ajras, Oued El Kanar, Oued Aârkoub, Oued Jnane Niche, **MA**, Barrage Allal El Fassi; Oosterbroek 2020 (CCW: **Rif**; **MA**)

﻿Symplecta (Trimicra) pilipes (Fabricius, 1787)

= *Trimicra﻿pilipes* Fabricius, in Pierre 1922a: 23

= *Trimicra﻿﻿﻿﻿andalusiaca* Strobl, in Pierre 1922b: 149

= *Trimicra﻿hirsutipes* Macquart, in Séguy 1930a: 22

Pierre 1922a, **AP**, Dradek (near Rabat), **HA**, Marrakech; Pierre 1922b, **MA**, Volubilis, **HA**, Oued Tensift; Séguy 1930a; Séguy 1941d, **AA**, Taroudant; Savchenko et al. 1992; Dakki 1997; Pârvu and Zaharia 2007; Driauach et al. 2013; Driauach and Belqat 2016, **Rif**, tributary of Oued Hachef, Oued Aârk­ob, Oued El Kanar, Oued Mezine, **AP**, Aïn Chouk (Larache); Oosterbroek 2020 (CCW: **Rif**)

*Tasiocera (Dasymolophilus) ﻿murina* (Meigen, 1818)

Driauach and Belqat 2016, **Rif**, Oued Farda, Oued Amsemlil; Oosterbroek 2020 (CCW: **Rif**)

##### Dactylolabinae

﻿***Dactylolabis* Osten-Sacken, 1860**

﻿*Dactylolabis (Dactylolabis) ﻿symplectoidea* Egger, 1863

Pierre 1922a, **AP**, Around Casablanca (coastal meseta); Savchenko et al. 1992; Dakki 1997; Driauach et al. 2013; Driauach and Belqat 2016; Oosterbroek 2020 (CCW)

##### Limnophilinae

﻿***Austrolimnophila* Alexander, 1920**

﻿*Austrolimnophila (Austrolimnophila) ﻿latistyla* Starý, 1977

Driauach and Belqat 2016, **Rif**, Oued Maggou; Oosterbroek 2020 (CCW: **Rif**)

﻿***Dicranophragma* Osten-Sacken, 1860**

﻿*Dicranophragma (Brachylimnophila) ﻿adjunctum* (Walker, 1848)

= ﻿*Neolimnomyia﻿adjuncta* (Walker, 1848), in Pârvu et al. 2006: 273

Pârvu et al. 2006, **AP**, Merja Zerga; Driauach et al. 2013; Driauach and Belqat 2016; Oosterbroek 2020 (CCW)

﻿*Dicranophragma (Brachylimnophila) ﻿nemorale* (Meigen, 1818)

Lackschewitz 1940b, **HA**, Tachdirt (2200–2700 m); Savchenko et al. 1992; Driauach et al. 2013; Driauach and Belqat 2016, **Rif**, Aïn Sidi Brahim Ben Arrif, tributary Oued Ouara, Oued Maggou; Oosterbroek 2020 (CCW: **Rif**)

﻿***Eloeophila* Rondani**, **1856**

﻿*Eloeophila ﻿maroccana* Starý, 2009

Starý 2009b, **HA**, Okaïmeden (2500–2800 m); Driauach et al. 2013; Driauach and Belqat 2016; Oosterbroek 2020 (CCW)

﻿***Euphylidorea* Alexander, 1972**

﻿*Euphylidorea (Euphylidorea) ﻿crocotula* (Séguy, 1941)

= *Phylidorea﻿crocotula* (Séguy), in Séguy 1941a: 28

Séguy 1941a, **HA**, Tachdirt (Toubkal, 2500 m); Savchenko et al. 1992, **HA**, Tachdirt (Toubkal); Starý and Oosterbroek 2008, **HA**, Oukaimeden (2500 m–2800 m), Imlil (1400 m); Driauach et al. 2013; Driauach and Belqat 2016, **Rif**, Aïn Bab Tariouant; Oosterbroek 2020 (CCW: **Rif**; **HA**)

﻿*Euphylidorea (Euphylidorea) dispar* (Meigen, 1818)

Driauach and Belqat 2016, **Rif**, Oued at 15 km from Fifi; Oosterbroek 2020 (CCW: **Rif**)

﻿*Euphylidorea (Euphylidorea) ﻿lineola* (Meigen, 1804)

Lackschewitz 1940b, **HA**; Savchenko et al. 1992; Starý and Freidberg 2007; Driauch et al. 2013; Driauach and Belqat 2016; Oosterbroek 2020 (CCW)

﻿***Hexatoma* Latreille, 1809**

﻿*Hexatoma (Hexatoma) ﻿bicolor* (Meigen, 1818)

Driauach and Belqat 2016, **Rif**, Oued at 15 km from Fifi, tributary of Oued Ouara, Oued Madissouka, Oued Maggou, Aïn Quanquben (Jebel Bou Bessoui), Oued Tkarae, Oued Tamerte; Oosterbroek 2020 (CCW: **Rif**)

﻿*Hexatoma (Hexatoma) ﻿gaedii* (Meigen, 1830)

Lackschewitz 1940b, **HA**, Tachdirt (2200 m–2700 m); Savchenko et al. 1992; Starý and Freidberg 2007; Driauach et al. 2013; Driauach and Belqat 2016; Oosterbroek 2020 (CCW: **HA**)

﻿***Pseudolimnophila* Alexander, 1919**

﻿*Pseudolimnophila (Pseudolimnophila) ﻿sepium* (Verrall, 1886)

Driauach et al. 2013, **Rif**, Guelta Tazia; Driauach and Belqat 2016, **Rif**, Dayat Tazia, Aïn Sidi Brahim Ben Ar­rif, Aïn Afersiw, Oued Maggou, Dayat Tazia, Oued Taida, Aïn Sidi Brahim Ben Arrif; Oosterbroek 2020 (CCW: **Rif**)

##### Limoniinae

﻿***Dicranomyia* Stephens, 1829**

﻿*Dicranomyia (Dicranomyia) ﻿﻿﻿affinis* (Schummel, 1829)

Driauach et al. 2013; Driauach and Belqat 2016, **Rif**, Oued Amsemlil, Dayat Lemtahane, Oued Tkarae, Marj El Kheyl, Aïn Sidi Brahim Ben Arrif, Dayat Tazia, Aïn El Maounzil, Aïn El Malâab, Dayat near Aïn Afersiw, Aïn Bab Tariouant; Oosterbroek 2020 (CCW: **Rif**)

﻿*Dicranomyia (Dicranomyia) ﻿chorea* (Meigen, 1818)

Pierre 1922b, **HA**, Haute Réghaya, Tannaout, Asni (1000 m–1250 m); Savchenko et al. 1992; Dakki 1997; Starý and Freidberg 2007; Driauach et al. 2013; Driauach and Belqat 2016, **Rif**, Oued Farda, Oued Kelâa, Aïn Ras el Ma, Oued Jnane Niche, Cascade Chrafate, maison forestière, Oued El Kanar, tributary of Oued Zarka, **HA**, Oued Sidi Fares (National Park of Toubkal); Oosterbroek 2020 (CCW: **Rif**; **HA**)

﻿*Dicranomyia (Dicranomyia) ﻿didyma* (Meigen, 1804)

Pierre 1922b, **HA**, Haute Réghaya (2000 m); Dakki 1997; Savchenko et al. 1992; Driauach et al. 2013; Driauach and Belqat 2016; Oosterbroek 2020 (CCW: **HA**)

﻿*Dicranomyia (Dicranomyia) ﻿goritiensis* (Mik, 1864)

Lackschewitz 1940b; Vaillant 1956b; Savchenko et al. 1992; Driauach et al. 2013; Driauach and Belqat 2016, **Rif**, Oued Maggou, Cascade Chrafate, Oued El Koub, Cascade Zarka, Âounsar Aheramen, maison forestière; Oosterbroek 2020 (CCW: **Rif**)

﻿*Dicranomyia (Dicranomyia) longicollis* (Macquart, 1846)

= *Telecephalalongicollis* (Macquart), in Pierre 1922a: 21, Pierre 1922b: 148, Séguy 1930a: 22

Séguy 1930a, **AP**, Dradek (near Rabat); Dakki 1997; Pierre 1922a, **AP**, Dradek, **HA**, Marrakech; Pierre 1922b, **AP**, Rabat; Savchenko et al. 1992; Driauach et al. 2013; Driauach and Belqat 2016, **Rif**, Oued Aârate, Barrage Moulay Bouchta, Oued Kbir, **MA**, Barrage Allal El Fassi; Oosterbroek 2020 (CCW)

﻿*Dicranomyia (Dicranomyia) ﻿mitis* (Meigen, 1830)

= ﻿*Dicranomyia﻿hygropetrica* Vaillant, in Vaillant 1956b: 42

Vaillant 1956b, **HA**, Asif Tessaout (M’Goum), Izourar, Tahanaout, Tamesrit, Imi-N’Ifri, Aguelmous, Sidi Chamarouch, Lac Tamhda (Anremer), Oukaimeden; Savchenko et al. 1992; Pârvu et al. 2006, **AA**, near Agadir, Souss plain to High Atlas occidental; Pârvu and Zaharia 2007; Gavryushin pers. comm. 2012, **HA**; Driauach et al. 2013, **Rif**, Tazia, Tisgris; Driauach and Belqat 2016; Oosterbroek 2020 (CCW)

﻿*Dicranomyia (Dicranomyia) ﻿modesta* (Meigen, 1818)

Driauach and Belqat 2016, **Rif**, Oued Farda, Oued El Kanar, Oued Jnane Niche, Oued Sidi Yahia Aârab; Oosterbroek 2020 (CCW: **Rif**)

﻿*Dicranomyia (Dicranomyia) ﻿novemmaculata* (Strobl, 1906)

Driauach and Belqat 2016, **Rif**, tributary of Oued el Fondak, Oued Aârate; Oosterbroek 2020 (CCW: **Rif**)

﻿*Dicranomyia (Dicranomyia) ﻿ventralis* (Schummel, 1829)

Driauach and Belqat 2016, **Rif**, Lake Badriouen; Oosterbroek 2020 (CCW: **Rif**)

﻿Dicranomyia (Glochina) sericata (Meigen, 1830)

Driauach et al. 2013; Driauach and Belqat 2016; Oosterbroek 2020 (CCW)

﻿*Dicranomyia (Melanolimonia) ﻿hamata* Becker, 1908

Driauach and Belqat 2016, **Rif**, tributary of Oued Kbir, Oued Aârate, Aïn Sidi Brahim Ben Arrif, Dayat Tazia; Oosterbroek 2020 (CCW: **Rif**)

﻿*Dicranomyia (Melanolimonia) ﻿morio* (Fabricius, 1787)

= ﻿*Limonia﻿pauliani* Séguy, in Séguy 1941a: 26

Séguy 1941a, **HA**, Tachdirt (Toubkal, 2500 m); Savchenko et al. 1992, **HA**, Tachdirt (Toubkal); Driauach et al. 2013; Driauach and Belqat 2016, **Rif**, Seguia Lemtahane, Dayat near Aïn Afersiw, Dayat Jebel Zemzem; Driauach and Belqat 2016; Oosterbroek 2020 (CCW)

﻿*Dicranomyia﻿majuscula* Pierre, 1924^1^

Pierre 1924a, **HA**, Haut Imminen (2400 m); Séguy 1930a, **HA**, Haut Imminen; Dakki 1997; Savchenko et al. 1992, **HA**, Haut Imminen; Driauach et al. 2013; Driauach and Belqat 2016; Oosterbroek 2020 (CCW)

﻿***Dicranoptycha* Osten-Sacken, 1860**

﻿*Dicranoptychafuscescens* (Schummel, 1829)

Eiroa 2000, **Rif**; Driauach et al. 2013; Driauach and Belqat 2016, **Rif**, Oued Mlilah, Oued Zendoula, **AP**, Oued Loukous; Oosterbroek 2020 (CCW)

***Geranomyia* Haliday, 1833**

*Geranomyia﻿caloptera* (Mik, 1867)

Driauach et al. 2013, **HA**, Setti Fatma; Driauach and Belqat 2016; Oosterbroek 2020 (CCW)

*Geranomyia﻿obscura* Strobl, 1900

Vaillant 1956b, **HA**, Lac Tamhda (Anremer), Oukaimeden, Izourar, Sidi Chamarouch, Tamesrit; Savchenko et al. 1992; Driauach et al. 2013; Driauach and Belqat 2016; Oosterbroek 2020 (CCW: **HA**)

﻿***Helius* Lepeletier & Serville, 1828**

﻿*Helius (Helius) ﻿hispanicus* Lackschewitz, 1928

Starý and Oosterbroek 2008, **HA**, Massif Toubkal, Imlil (17 km S Asni, 1700–1900 m); Driauach et al. 2013; Driauach and Belqat 2016, **Rif**, Oued Amsemlil; Oosterbroek 2020 (CCW: **Rif**)

﻿*Helius (Helius) ﻿pallirostris* Edwards, 1921

Driauach and Belqat 2016, **AP**, Aïn Chouk (Larache); Oosterbroek 2020 (CCW: **Rif**)

﻿***Limonia* Meigen, 1803**

﻿*Limoniaflavipes* (Fabricius, 1787)

Pierre 1922b, **HA**, Haute Réghaya, Asni (1250 m); Savchenko et al. 1992; Dakki 1997; Driauach et al. 2013; Driauach and Belqat 2016; Oosterbroek 2020 (CCW)

﻿*Limonia﻿hercegovinae* (Strobl, 1898)

Starý and Oosterbroek 2008, **MA**, Ifrane (1700 m), **HA**; Gavryushin pers. comm. 2012, **HA**; Driauach et al. 2013; Driauach and Belqat 2016; Oosterbroek 2020 (CCW: **HA**)

﻿*Limonia﻿macrostigma* (Schummel, 1829)

Starý and Oosterbroek 2008, **HA**, 5 km from Oukaimeden (2350 m); Driauach et al. 2013; Driauach and Belqat 2016; Oosterbroek 2020 (CCW)

﻿*Limonia﻿nubeculosa* Meigen, 1804

Pierre 1922b, **HA**, Haute Réghaya, Asni (1250 m); Savchenko et al. 1992; Dakki 1997; Gavryushin pers. comm. 2012, **HA**; Driauach and Belqat 2016, **Rif**, tributary of Oued El Fondak, Aïn Ras el Ma, Aïn Boughaba, Âounsar Aheramen, Aïn Takhninjoute, maison forestière, Oued Madissouka, Aïn Quan­quben (Jebel Bou Bessoui), **EM**, Oued Azila; Oosterbroek 2020 (CCW: **Rif**; **HA**)

﻿*Limonia﻿phragmitidis* (Schrank, 1781)

Starý and Oosterbroek 2008, **MA**, Ifrane (1700 m); Driauach and Belqat 2016, **Rif**, Aïn Quanquben (Jebel Bou Bessoui); Oosterbroek 2020 (CCW)

#### ﻿﻿PEDICIIDAE

K. Kettani, P. Oosterbroek

Number of species: **6**. Expected: 10

Faunistic knowledge of the family in Morocco: moderate

##### Pediciinae

﻿***Dicranota* Zetterstedt, 1838**

﻿*Dicranota (Dicranota) ﻿bimaculata* (Schummel, 1829)

Pierre 1922b, **HA**, Haute Réghaya (1800 m); Savchenko et al. 1992 (? Morocco); Dakki 1997; Driauach et al. 2013; Driauach and Belqat 2016; Oosterbroek 2020 (CCW)

﻿*Dicranota (Dicranota) ﻿irregularis* Pierre, 1921

Pierre 1922b, **HA**, Haute Réghaya (1800 m); Savchenko et al. 1992, **HA**, Cirque d’Arround (Haute Réghaya); Dakki 1997; Driauach et al. 2013; Driauach and Belqat 2016; Oosterbroek 2020 (CCW)

﻿*Dicranota (Ludicia) ﻿claripennis* (Verrall, 1888)

Driauach and Belqat 2016, **Rif**, Oued Amsemlil, maison forestière; Oosterbroek 2020 (CCW)

﻿*Dicranota (Paradicranota) ﻿candelisequa* Starý, 1981

Pârvu et al. 2006, **AP**, Merja Zerga; Driauach et al. 2013; Driauach and Belqat 2016; Oosterbroek 2020 (CCW)

﻿*Dicranota (Paradicranota) ﻿landrocki* Czižek, 1931

Driauach et al. 2013, **Rif**, Fifi (1252 m); Driauach and Belqat 2016, **Rif**, Oued Ouara, Oued Taida, Aïn Sidi Brahim Ben Arrif, Âounsar Aheramen, Oued Tizekhte, Oued Mezine, maison forestière, Aïn Bab Tariouant, **HA**, Imlil (Assif Haouz); Oosterbroek 2020 (CCW)

﻿***Tricyphona* Zetterstedt, 1838**

﻿*Tricyphona (Tricyphona) ﻿immaculata* (Meigen, 1804)

Driauach and Belqat 2016, **Rif**, maison forestière, Dayat Lemtahane; Oosterbroek 2020 (CCW)

#### ﻿﻿TIPULIDAE

K. Kettani, P. Oosterbroek, H. de Jong

Number of species: **39**. Expected: 42

Faunistic knowledge of the family in Morocco: moderate

##### Dolichopezinae

﻿***Dolichopeza* Curtis, 1825**

﻿*Dolichopeza (Dolichopeza) ﻿hispanica* Mannheims, 1951

Theowald and Oosterbroek 1980, **HA**, Aghbalou, Oukaimeden, Imlil, Tadmant; Oosterbroek and Theowald 1992; Oosterbroek and Lantsov 2011, **HA**, Oukaimeden (2300 m), Aghbalou (Massif Toubkal), 43 km S Marrakech (1000 m), Imlil (17 km S Asni, 1700–1900 m), Tadmant (17 km E Asni); Adghir et al. 2018, **Rif**, Kitane, Aîn Ras el Ma (Chefchaouen); Oosterbroek 2020 (CCW)

##### Tipulinae

﻿***Nephrotoma* Meigen, 1803**

﻿*Nephrotoma﻿﻿﻿alluaudi* (Pierre, 1922)

= *Pachyrhina﻿lunulicornis* Schummel, in Pierre 1922a: 24

= *Pachyrhina﻿﻿﻿alluaudi* Pierre, in Pierre 1922b: 150, Dakki 1997: 62

= ﻿*Pales﻿﻿﻿alluaudi* (Pierre), in Mannheims 1951: 47

Pierre 1922b, **HA**, Tannaout (1000 m); Mannheims 1951, **Rif**, Beni Seddat, **AP**, Lagune Guedira; **HA**, Taddert north of Marrakech, Goundafa (1200 m); **AA**, Llano Amarillo, Tlata Reisana; Oosterbroek 1979b, **Rif**, **HA**, Imlil (1400 m), Tizi-n’Tichka (2200 m); Theowald and Oosterbroek 1980, **AP**, Rabat, Guedira lagoon, **MA**, Immouzer, Ifrane, Timahdit, Aghbalou, Tizi-n’Zou, **HA**, Marrakech, Goundafa, Taddert, Dayat, Tizi-n’Test, Tizi-n’Tichka, Asni, Imlil, Oukaimeden, Setti Fatma, Tinmel, Acif Tifni, **AA**, Taroudant, Mikdana, Sidi Said bou Merdoul, Tlata Reisana, Llano Amarillo; Eiroa 1990, **MA**, Azrou, Ajabo; Oosterbroek and Theowald 1992, **HA**, Tannaout; Dakki 1997; Mouna 1998; Adghir et al. 2018, **Rif**, Ras el Ma (Chefchaouen), Oued Laou, Dardara, Douar Mouarâa, Oued Zandoula, Laghmari-Rmal, Douar Louamera, Douar Laheyayda, Oued Jnane Niche, Cabo Negro, Oued Beni Said, Oued El Kanar, Oued Amsemlil; Oosterbroek 2020 (CCW) – MISR (**HA**), MHNV, MNCNM, MAKB

﻿*Nephrotoma﻿﻿appendiculata pertenua* Oosterbroek, 1978

Oosterbroek 1978, **Rif**, 9 km SW Chefchaouen; Theowald and Oosterbroek 1980, **Rif**, Dardara, **MA**, Fès, Immouzer, Khemisset; Oosterbroek and Theowald 1992; de Jong 1993, 1998, **Rif**; Adghir et al. 2018, **Rif**, Oued Ametrasse, Dayat Aïn Jdioui, Barrage Moulay Bouchta, Oued Sahil, Dayat Jebel Zemzem, Aïn Sidi Brahim Ben Arrif, Oued Nakhla, Oued Tizekhte, Lot Hemmadi, Douar Ayacha, Douar Louamera, Dayat Mezine, Aïn El Malâab, Aïn Takhninjoute, Dayat Tazia, Oued Taida, maison forestière Tazia, Tourbière Amesmlil, Oued El Hamma, Oued Kbir, Jebel Lakraâ; Oosterbroek 2020 (CCW)

﻿*Nephrotoma﻿astigma* Pierre, 1925

Theowald and Oosterbroek 1980, **Rif**, Dardara, **MA**, Taza; Adghir et al. 2018, **Rif**, Oued Tabandout, Etang Maggou, Aïn El Malâab, Douar Remla; Oosterbroek 2020 (CCW)

﻿*Nephrotoma﻿fontana* Oosterbroek, 1978

de Jong 1998, **Rif**; Adghir et al. 2018, **Rif**, Ketama, maison forestière Tazia, Dayat Tazia; Oosterbroek 2020 (CCW)

﻿*Nephrotomaguestfalica ﻿vaillanti* de Jong, Adghir & Bosch, 2021

de Jong et al. 2021, **Rif**, Ras el Ma (Chefchaouen, 500 m), Fomento, Oued Laou (6 km north-west of Chefchaouen, 200 m), Jebel Tissouka (3 km south of Chefchaouen, 500 m; 3 km south of Chefchaouen, 700 m; 4 km south-east of Chefchaouen, 700–800 m; 5 km southeast of Chefchaouen, 700–900 m; 5 km south of Chefchaouen, 900–1000 m), Bab Taza (25 km south-east of Chefchaouen, 750–800 m), Dardara (10 km south of Chefchaouen, 300 m), Oued Martil, Nakhla, Âounsar Aheramen, Boumerouil, Oued Maggou, Oued Maggou (Aïn Ras el Ma), Douar Kitane, Oued Sidi Yahia Aârab, Oued Tamerte, Oued Zandoula, Oued El Hamma, Lot Hemmadi, Oued Tamerte, Oued Sidi Mohamed Saâda, Oued El Koub, Douar Iholebatine, Dayat Tazia, Belouazen, Oued Lemtahane, Oued Siflaou, Oued Amsemlil, **AP**, Oued Loukous, Aïn el-Aouda, **MA**, Ifrane (road to Mischliffen, 1680 m), N.S. and W of Ifrane (1400–1800 m), Khemisset, Azrou, Oum-er-Rbia, **HA**, M’semrir (bord de l’Oued), Rich, Haute Rhégaya (identified by Mannheims in 1951 as ﻿*surcoufi*)

﻿*Nephrotoma ﻿﻿﻿luteata* (Meigen, 1818)

Oosterbroek 1979a, **Rif**, Targuist, Chefchaouen, **HA**, Kasba Taguendaft, near Oukaimeden; Theowald and Oosterbroek 1980, **Rif**, Targuist, Chefchaouen, **HA**, Kasba Taguendaft, near Oukaimeden; Oosterbroek and Theowald 1992; Pârvu et al. 2006, **AP**, Merja Zerga; Adghir et al. 2018, **Rif**, Oued Laou, Dardera, Oued Nakhla; Oosterbroek 2020 (CCW)

﻿*Nephrotoma ﻿﻿﻿subanalis* (Mannheims, 1951)

= ? *Pachyrhina ﻿﻿analis* Schummel, in Pierre 1922a: 24, Pierre 1922b: 150

= ﻿*Pales ﻿﻿﻿subanalis* Mannheims, in Mannheims 1951: 56

Mannheims 1951, **HA**, Tachdirt (2200–2900 m); Vaillant 1956b, **HA**, Oukaimeden (2250 m); Oosterbroek 1979c, **HA**, Tachdirt (2200–2900 m); Theowald and Oosterbroek 1980, **HA**, Tachdirt, Oukaimeden, M’Goum, Tadmant, Imlil, Tizi-N’Tichka; Oosterbroek and Theowald 1992, **HA**, Tachdirt; Oosterbroek 2020 (CCW)

﻿*Nephrotoma ﻿﻿﻿submaculosa* Edwards, 1928

Oosterbroek 1982, **Rif**, Ketama, Dardara, **MA**, Azrou; Oosterbroek and Theowald 1992; de Jong 1998, **Rif**, **Atlas**; Oosterbroek 2011; Adghir et al. 2018, **Rif**, Jebel Dahedouh, Ketama, Aïn Sidi Brahim Ben Arrif, Oued Taida, tributary of Oued Ouara, Sidi Chouiref, Dayat Tazia; Oosterbroek 2020 (CCW)

﻿*Nephrotoma ﻿﻿﻿sullingtonensis* Edwards, 1938

Oosterbroek 1978; Theowald and Oosterbroek 1980, **Rif**, Bab Berred; Oosterbroek 1982**Rif**, Bab Berred; de Jong 1993, 1998, **Rif**; Oosterbroek and Theowald 1992; Adghir et al. 2018, **Rif**, 4 km SE Ketama, Barrage Moulay Bouchta, Oued Aârate, Aïn Takhninjoute, Oued Jbara, Aïn El Malâab, Aïn El Ma Bared, Oued Lemtahane, maison forestière Tazia, Oued Taida, Dayat Tazia; Oosterbroek 2020 (CCW)

﻿***Tipula* Linnaeus, 1758**

﻿*Tipula (Acutipula) ﻿anormalipennis* Pierre, 1924

Pierre 1924b, **HA**, Haut Imminen; Séguy 1930a, **HA**, Haut Imminen; Séguy 1941a, **HA**, Tachdirt (2500 m), Haut Imminen; Mannheims 1952, **HA**, Tachdirt (2500 m), Imminen (2400–2500 m), Arround (1950 m); Vaillant 1956b, **HA**, Lake of Tamhda (Anremer, 2900 m); Theowald and Oosterbroek 1980, **HA**, Anremer, Haut-Immenen, Tachdirt, Arround, Oukaimeden; Vermoolen 1983, **HA**, Haut Imminen, Oukaimeden (2500–2800 m), Arround (1950 m), Tachdirt (2500 m); Oosterbroek and Theowald 1992; de Jong 1994a, **HA**; de Jong 1998, **HA**; Dakki 1997; Oosterbroek 2020 (CCW)

﻿*Tipula (Acutipula) ﻿repentina* Mannheims, 1952

= ﻿*Tipulamaxima* Poda, in Séguy 1941a: 26

Séguy 1941a, **HA**, Tachdirt (2500 m); Mannheims 1952, **HA**, Tachdirt (2200–2700 m); Vaillant 1956b, **HA**, Lac Tamhda (Anremer, 2900), M’Goum (2500 m); Theowald and Oosterbroek 1980, **HA**, Anremer, M’Goum, Tachdirt, Tizi-N’Tichka, Asni, Oukaimeden, Setti Fatma, Imlil, Tadmant; Vermoolen 1983, **MA**, Ifrane, **HA**, Androment, M’Goum, Tadmant, Tizi-N’Test, Setti Fatma, Oukaimeden, Tizi-N’Tichka; Oosterbroek and Theowald 1992, **HA**, Tachdirt; de Jong 1994a, **MA**, **HA**; de Jong 1998, **HA**; Dakki 1997; Oosterbroek 2020 (CCW)

﻿*Tipula (Acutipula) rifensis* Theowald & Oosterbroek, 1980

Theowald and Oosterbroek 1980, **Rif**, Targuist; Vermoolen 1983, **Rif**, Targuist, Tidiguin (90 km E. of Ouezzane, 2350 m); Oosterbroek and Theowald 1992, **Rif**; de Jong 1994a, 1998, **Rif**; Adghir et al. 2018; Oosterbroek 2020 (CCW)

﻿*Tipula (Emodotipula) ﻿leo* Dufour, 1991

= ﻿*Tipula (Emodotipula) ﻿obscuriventris* Strobl^2^, in Oosterbroek and Theowald 1992: 99

Oosterbroek and Theowald 1992 (?); Dufour 2003, **Rif**; Adghir et al. 2018, **Rif**, Jebel Tissouka; Oosterbroek 2020 (CCW)

﻿*Tipula (Lunatipula) ﻿bivittata* Pierre, 1922

Pierre 1922a, **AP**, Maâmora between Kénitra and Oued Beth, Dradek (near Rabat); Pierre 1922b, **AP**, Rabat; Mannheims 1968; Theowald and Oosterbroek 1980, **AP**, forest of Maâmora, Dradek; Oosterbroek and Theowald 1992, **AP**, forest of Maâmora, Dradek; Dakki 1997; Oosterbroek 2020 (CCW) – MISR (**AP**, Kénitra, Dradek)

﻿*Tipula (Lunatipula) ﻿cinereicolor* Pierre, 1924

Pierre 1924b, **HA**, Haut Imminen; Séguy 1930a, **HA**, Tachdirt (3100–3200 m); Theowald 1973, **HA**, Haut Imminen (2400 m); Theowald and Oosterbroek 1980, **MA**, Ifrane, **HA**, Oukaimeden, Tachdirt, Haut-Imminen; Eiroa 1990, **MA**, Ajabo; Oosterbroek and Theowald 1992, **HA**, Imminen; Dakki 1997; Oosterbroek 2020 (CCW)

﻿*Tipula (Lunatipula) ﻿cornicula* Pierre, 1922

Pierre 1922b, **HA**, Arround (2000 m); Séguy 1930a, **HA**, Tachdirt (3100–3200 m); Theowald 1973, **HA**, Cirque d’Arround (2000 m), Tachdirt (2200–2700 m), Goundafa (1200 m); Theowald and Oosterbroek 1980, **HA**, Oukaimeden, Tachdirt, Goundafa; Oosterbroek and Theowald 1992, **HA**, Tachdirt; Dakki 1997; Oosterbroek 2020 (CCW) – MISR (**HA**)

﻿*Tipula (Lunatipula) ﻿fabiola* Mannheims, 1968

Theowald and Oosterbroek 1980, **Rif**, Bab Berred, Ras El Ma (Chefchaouen), **MA**, Jebel Abad, Ifrane; Oosterbroek and Theowald 1992; Adghir et al. 2018; Oosterbroek 2020 (CCW)

﻿*Tipula (Lunatipula) ﻿hermes* Theischinger, 1977

Theischinger 1977, **Rif**, north of Ouezzane; Theowald 1980; Theowald and Oosterbroek 1980, **Rif**, north of Ouezzane; Oosterbroek and Theowald 1992, **Rif**, Ouezzane; Adghir et al. 2018, **Rif**, 4 km SE Ketama, Oued Ametrasse, Aïn El Ma Bared; Oosterbroek 2020 (CCW)

﻿*Tipula (Lunatipula) ﻿﻿iberica ﻿iberica* Mannheims, 1963

= ﻿*Tipula ﻿﻿﻿lunata* Linnaeus, in Pierre 1922b: 149, Séguy 1930a: 23

Pierre 1922b, **HA**, Haute Réghaya, Tannaout (1000 m); Séguy 1930a, **HA**, Haut Imminen; Mannheims 1963, **MA**; Theowald and Oosterbroek 1980, **Rif**, Bab Berred, **MA**, Ifrane, Taounate; Eiroa 1990, **MA**, Ifrane; Oosterbroek and Theowald 1992; Oosterbroek 2009; Adghir et al. 2018, **Rif**, Ketama; Oosterbroek 2020 (CCW) – MISR (**HA**)

﻿*Tipula (Lunatipula) iberica spinula* Theischinger, 1980

Theischinger 1980, **HA**, Oukaimeden (1300–2800 m); Theowald and Oosterbroek 1980, **HA**, Oukaimeden; Oosterbroek and Theowald 1992, **HA**, Oukaimeden; Oosterbroek 2020 (CCW)

﻿*Tipula (Lunatipula) ﻿peliostigma peliostigma* Schummel, 1833

Eiroa 1990, **MA**, Azrou; Mouna 1997; Oosterbroek 2020 (CCW)

﻿*Tipula (Lunatipula) ﻿pjotri* de Jong & Adghir, 2018

Adghir et al. 2018, **Rif**, Jebel El Kelâa (Talassemtane, 1340 m)

﻿*Tipula (Lunatipula) ﻿pseudocinerascens* Strobl, 1906

Adghir et al. 2018, **Rif**, Stehat, Oued Taida, Perdicaris Park, Dayat Tazia

﻿*Tipula (Lunatipula) ﻿rocina* Theischinger, 1979

Theowald and Oosterbroek 1980, **Rif**, Tétouan; Oosterbroek and Theowald 1992; Oosterbroek 2009; Adghir et al. 2018; Oosterbroek 2020 (CCW)

﻿*Tipula (Lunatipula) ﻿selenaria* Mannheims, 1967

Mannheims 1967, **MA**, Jebel Tazzeka (1500–1989 m), **HA**, Goundafa (1200 m); Theowald and Oosterbroek 1980, **MA**, Tazzeka, **HA**, Foum Keneg, Goundafa, Oukaimeden; Eiroa 1990, **MA**, Ajabo; Oosterbroek and Theowald 1992, **HA**, Goundafa; de Jong 1995, **MA**, **HA**, Oukaimeden; Oosterbroek 2020 (CCW)

﻿*Tipula (Lunatipula) ﻿stimulosa* Mannheims, 1973

Adghir et al. 2018, **Rif**, Jebel El Kelâa (Talassemtane, 1340 m)

﻿*Tipula (Lunatipula) ﻿subfalcata* Mannheims, 1967

de Jong 1995, 1998, **Rif**; Adghir et al. 2018, **Rif**, Jebel Tissouka, Oued Tamerte, Oued El Koub; Oosterbroek 2020 (CCW)

﻿*Tipula (Lunatipula) ﻿subpustulata* Mannheims, 1963

= ﻿*Tipula ﻿﻿﻿pustulata* Pierre, in Pierre 1922b: 150

Pierre 1922b, **AP**, Mogador; Vaillant 1956b, **HA**, M’Goun (2500 m); Mannheims 1963, **AP**, Aïn el Aouda, **MA**, Jebel Tazzeka (1600–1989 m), **HA**, Goundafa (1200 m), Tachdirt (2200–2900 m), **AA**, Lac Goulmima; Theowald 1972; Theowald and Oosterbroek 1980, **AP**, Aïn el Aouda, **MA**, Iebel Tazzeka, **HA**, M’Goum, Goundafa, Tachdirt, Oukaimeden, Imlil; Eiroa 1990, **MA**, Ajabo; Oosterbroek and Theowald 1992, **HA**, Goundafa; Dakki 1997; Adghir et al. 2018, **Rif**, 4 km SE Ketama, Aïn El Ma Bared; Oosterbroek 2020 (CCW) – MISR (**AP**, Mogador)

﻿*Tipula (Lunatipula) ﻿tazzekai* Theowald, 1973

Theowald 1973, **MA**, Jebel Tazzeka (1600–1989 m); Theowald and Oosterbroek 1980, **MA**, Jebel Tazzeka; Eiroa 1990, **MA**, Jebel Hebri; Oosterbroek and Theowald 1992, **MA**, Jebel Tazzeka; Oosterbroek 2020 (CCW)

﻿*Tipula (Savtshenkia) ﻿atlas* Pierre, 1924

Pierre 1924b, **HA**, Tachdirt (3100–3250 m); Séguy 1930a, **HA**, Tachdirt (3100–3200 m); Vaillant 1956b, **HA**, Cascade Siroua (3000 m), M’Goun (2500 m), Toubkal (3350 m), Lake of Tamhda (Anremer, 2900 m); Mannheims 1964, **HA**; Theowald 1973, **HA**; Theowald 1980, **HA**; Theowald and Oosterbroek 1980, **HA**, Toubkal, Sources de Tessaouts, M’Goum, Siroua, Anremer, Tachdirt, Oukaimeden, Tizi-N’Tichka, Tadmant; Eiroa 1990, **MA**, Oum-Er-Rbia; Oosterbroek and Theowald 1992, **HA**, Tachdirt; de Jong 1994b; de Jong 1998, **Atlas**; Dakki 1997; Oosterbroek 2020 (CCW)

﻿*Tipula (Savtshenkia) ﻿breviantennata* Lackschewitz, 1933

de Jong 1998, **Rif**; Adghir et al. 2018, **Rif**, Ras el Ma (Chefchaouen), Oued Maggou, Douar Aouzighen; Oosterbroek 2020 (CCW)

﻿*Tipula (Savtshenkia) ﻿confusa* van der Wulp, 1883

Adghir et al. 2018, **Rif**, maison forestière (Talassemtane)

﻿*Tipula (Savtshenkia) rufina ﻿rufina* Meigen, 1818

Theowald and Oosterbroek 1980, **HA**, Oukaimeden; Theowald and Oosterbroek 1983, **Rif**, **Atlas**; Oosterbroek and Theowald 1992; Adghir et al. 2018, **Rif**, Ras el Ma (Chefchaouen); Oosterbroek 2020 (CCW)

﻿*Tipula (Tipula) mediterranea* Lackschewitz, 1930

Vaillant 1956b, **HA**, Oukaimeden (2250 m), M’Goun (2500 m); Mannheims 1952; Theowald and Oosterbroek 1980, **MA**, Ifrane, **HA**, Oukaimeden, M’Goum, Asni, M’Semrir, Ifni, Bab-Rou-Idie, Tizi-N’Tichka, Setti Fatma; Theowald 1984, **Rif**; Eiroa 1990, **MA**, Azrou, Oum-Er-Rbia; Oosterbroek and Theowald 1992; Pârvu et al. 2006**AP**, Merja Zerga; Adghir et al. 2018, **Rif**, Ras el Ma (Chefchaouen), Jebel Tissouka, Bab Taza, Dardara, 4 km SE Ketama, Aïn Afersiw, Douar Kitane, Dayat Jebel Zemzem, Wilaya (Tétouan), Aïn El Ma Bared, Oued Tamerte, Douar Louamera, Tourbière Amesmlil, Dayat Mezine, Hejar Nehal, tributary of Oued Ouara, Oued Tkarae, Oued Jnane Niche, Dayat Afrate, Oued Ametrasse; Oosterbroek 2020 (CCW)

﻿*Tipula (Tipula) ﻿oleracea* Linnaeus, 1758

Mannheims 1952, **Rif**, Tlata Ketama; Theowald and Oosterbroek 1980, **Rif**, Tlata Ketama; Theowald 1984, **Rif**; Oosterbroek and Theowald 1992; Dakki 1997; Pârvu and Zaharia 2007; Oosterbroek 2011; Adghir et al. 2018, **Rif**, Oued Laou, Dardara, Jebel Tissouka, Ksar Rimal, 15 km from Fifi, Oued Aârate, Oued El Hamma, Douar Louamera, Oued Zaouya, Près de Beni Said, Wilaya (Tétouan), Tourbière Amesmlil, Oued Smir, Aïn Jdida; Oosterbroek 2020 (CCW) – MISR

﻿*Tipula (Vestiplex) ﻿vaillanti ﻿vaillanti* Theowald, 1977

Adghir et al. 2018, **Rif**, Jebel El Kelâa (Talassemtane, 1340 m), Douar Kitane

﻿*Tipula (Yamatotipula) ﻿afriberia ﻿afriberia* Theowald & Oosterbroek, 1980

Theowald and Oosterbroek 1980, **HA**, Oukaimeden; Oosterbroek and Theowald 1992, **HA**, Oukaimeden; Oosterbroek 1994a; Adghir et al. 2018, **Rif**, Jebel Tissouka, Dardara, Douar Mokedassen, Oued Zarka; Oosterbroek 2020 (CCW)

﻿*Tipula (Yamatotipula) ﻿barbarensis* Theowald & Oosterbroek, 1980

= ﻿*Tipulalateralis* Meigen, in Pierre 1922a: 23, Pierre 1922b: 150, Séguy 1930a: 23, Séguy 1941a: 26, Mannheims 1952: 98 (in part), Vaillant 1956b: 238

Pierre 1922a, **AP**, Dradek (Rabat), **MA**, Azrou (riverside of Oued Tigrigra); Pierre 1922b, **AP**, Mogador, **MA**, Beni Méllal, **HA**, Asni; Séguy 1930a; Séguy 1941a, **HA**, Imi n’Ouaka (1500 m); Mannheims 1952; Vaillant 1956b, **HA**, Oukaimeden; Theowald and Oosterbroek 1980, **Rif**, Dardara, **MA**, Ifrane, Aghbalou, **HA**, Asni, Oukaimeden, Setti Fatma, Imlil, Tizi-N’Tichka, Tadmant, Tachdirt; Eiroa 1990, **MA**, Azrou, Oum-er-Rbia, Ifrane; Oosterbroek and Theowald 1992, **HA**, Setti Fatma; Oosterbroek 1994a, **Rif**, Dardara, **MA**, Ifrane, Aghbalou, **HA**, Oukaimeden, Setti Fatma, Imlil, Tizi-N’Tichka, Tadmant, Asni, Tachdirt; Dakki 1997; Adghir et al. 2018, **Rif**, Ras el Ma (Chefchaouen), Jebel Tissouka, Bab Taza, Dardara, 4 km SE Ketama, Aïn El Manzela, Aïn Bab Tariouante, Dayat Aïn Afersiw, Oued El Kanar, Dayat Afrate, Douar Kitane, Wilaya (Tétouan), 15 km from Fifi, Aïn Sidi Brahim Ben Arrif, Oued Nakhla, Âounsar Aheramen, Oued Boumerouil, Douar Zaouya, Oued Tizekhte, Oued Samsa, Dayat Aïn Jdioui, Douar Ouled Laghmari-Rmal, maison forestière Tazia, Hejar Nehal, Lot Hemmadi, tributary of Oued Ouara, Oued Sidi Mohamed Saâda, Oued Amsemlil, Tourbière Amesmlil, Beni Salah, Oued Jnane Niche, Oued Maggou, Souk Lhed Beni Darkoul, Oued Imassouden, Aïn Helouma, Source Zarka, Aïn Kchour; Oosterbroek 2020 (CCW) – MISR

### ﻿﻿Trichoceridoidea

#### TRICHOCERIDAE

K. Kettani, E. Krzemińska

Number of species: **8**. Expected: 10

Faunistic knowledge of the family in Morocco: good

##### Trichocerinae

﻿***Trichocera* Meigen, 1803**

﻿*Trichocera (Trichocera) ﻿hiemalis* (DeGeer, 1776)

Pierre 1922b (**AP**, Rabat); Dakki 1997; Mouna 1998; **AP** (Rabat) – MISR

﻿*Trichocera (Trichocera) ﻿marocana* Driauach, Krzemińska & Belqat, 2015

Driauach et al. 2015, **Rif**, Oued Akrir

﻿*Trichocera (Saltrichocera) ﻿annulata* Meigen, 1818

Driauach et al. 2015, **Rif**, affluent Oued Akrir, Dayat Fifi, Oued Taria, Oued Kelâa, Oued Ouara, Oued à 15 km de Fifi, Aïn Mâaze, Oued Maggou, Aïn Quanquben, Oued Tizekhte, Forêt Jebel Bouhachem, **EM**, Grotte du Chameau, Oued Zegzel, Aïn Sidi Yahia, Cascade Grotte des pigeons, Oued Tafoughalt

﻿*Trichocera (Saltrichocera) ﻿pappi* Krzemińska, 2003

Driauach et al. 2015, **Rif**, Dayat Fifi, affluent Oued Akrir, Oued Amsemlil, Ruisseau Agoummir, maison forestière, **EM**, Cascade Grotte des Pigeons, **MA**, Seguia El Hajeb

﻿*Trichocera (Saltrichocera) ﻿saltator* (Harris, 1776)

Driauach et al. 2015, **Rif**, Dayat Fifi, Oued à 15 km de Fifi, Forêt Jebel Bouhachem, maison forestière, **EM**, Cascade Grotte des pigeons

﻿*Trichocera (Saltrichocera) ﻿sardiniensis* Petrašiūnas, 2009

Driauach et al. 2015, **Rif**, Oued à 15 km de Fifi, affluent Oued Akrir, Oued Amsemlil, maison forestière

﻿*Trichocera (Saltrichocera) ﻿regelationis* (Linnaeus, 1758)

Driauach et al. 2015, **Rif**, Oued Ouara, Aïn el Ma Bared, Oued à 15 km de Fifi, Cascade Chrafate, Aïn Quanquben

﻿*Trichocera (Saltrichocera) ﻿rufescens* Edwards, 1921

Driauach et al. 2015, **Rif**, affluent Oued Akrir, Dayat Fifi, Oued Tassikeste, Oued Farda, Aïn Mâaze

### ﻿Psychodoidea

#### ﻿﻿PSYCHODIDAE

K. Kettani, R. Wagner

Number of species: **51**. Expected: 70

Faunistic knowledge of the family in Morocco: moderate

##### Phlebotominae

﻿***Phlebotomus* Loew, 1845**

﻿*Phlebotomus (Larroussius) ﻿ariasi* Tonnoir, 1921

Gaud 1947a; Gaud and Laurent 1952, **AP**, Rabat; Bailly-Choumara et al. 1971, **AP**, **MA**, **HA**; Rioux et al. 1974; Mouna 1998; Guernaoui et al. 2005, **MA**, **HA**; Bounamous 2010; Boussaa et al. 2005; Boussaa et al. 2010

﻿*Phlebotomus (Larroussius) chadlii* Rioux, Juminer & Gibily, 1966

Rioux et al. 1974, **HA**; Rioux et al. 1975; Croset et al. 1978; Mouna 1998; Bounamous 2010

﻿*Phlebotomus (Larroussius) ﻿langeroni* Nitzulescu, 1930

Rislorcelli 1941, **HA**; Bailly-Choumara et al. 1971, **AP**, **EM**; Croset et al. 1978; Mouna 1998

﻿*Phlebotomus (Larroussius) longicuspis* Nitzulescu, 1930

Rislorcelli 1941, **HA**; Gaud and Laurent 1952, **AP**, Rabat; Bailly-Choumara et al. 1971, **Rif**, **EM**, **AP**, **MA**, **HA**, **AA**; Rioux et al. 1974, **HA**; Croset et al. 1978; Mouna 1998; Guernaoui et al. 2005, **Rif**, Chefchaouen, **HA**, **AA**, Agadir; Boussaa et al. 2005; Boussaa 2008; Boussaa et al. 2008; Boussaa et al. 2010; Bounamous 2010; Zarrouk et al. 2016

﻿*Phlebotomus (Larroussius) mariae* Rioux, Croset, Léger and Bailly-Choumara, 1974

Hervy et al. 1994; Rioux et al. 1974, **HA**; Mouna 1998; Guernaoui et al. 2005, **MA**, **HA**

﻿*Phlebotomus (Larroussius) perfiliewi* Parrot, 1930

Rioux et al. 1977; Croset et al. 1978; Mouna 1998

﻿*Phlebotomus (Larroussius) perniciosus* Newstead, 1911

Séguy 1930a; Gaud and Laurent 1952, **AP**, Rabat; Bailly-Choumara et al. 1971, **AP**, **EM**, **MA**, **HA**, **AA**; Mouna 1998; Guernaoui et al. 2005, **Rif**, Chefchaouen, **HA**; Boussaa et al. 2008; Bounamous 2010; Boussaa et al. 2010; Zarrouk et al. 2016

﻿*Phlebotomus (Paraphlebotomus) alexandri* Sinton, 1928

Bailly-Choumara et al. 1971, **AA**; Abonnenc 1972; Rioux et al. 1974, **HA**; Croset et al. 1978; Mouna 1998; Colacicco-Mayhugh et al. 2010, **EM**; Boussaa et al. 2010; Bounamous 2010

﻿*Phlebotomus (Paraphlebotomus) ﻿chabaudii* Croset, Abonnenc & Rioux, 1970

Rioux et al. 1974, **HA**; Rioux et al. 1975; Mouna 1998

*Phlebotmus (Paraphlebotomus) ﻿kazeruni* Theodor & Mesghali, 1964

Mouna 1998

﻿*Phlebotomus (Paraphlebotomus) ﻿riouxi* Depaquit, Killick-Kendrick & Léger, 1998

Bounamous 2010

﻿*Phlebotomus (Paraphlebotomus) sergenti* Parrot, 1917

Séguy 1930a; Rislorcelli 1941; Rislorcelli 1947; Gaud and Laurent 1952, **AP**, Rabat; Bailly-Choumara et al. 1971, **Rif**, **AP**, **EM**, **MA**, **HA**, **AA**; Abonnenc 1972; Rioux et al. 1974, **HA**; Mouna 1998; Boussaa et al. 2009; Bounamous 2010

﻿*Phlebotomus (Phlebotomus) bergeroti* Parrot, 1934

Rioux et al. 1975, **HA**; Mouna 1998; Bounamous 2010

﻿*Phlebotomus (Phlebotomus) papatasi* (Scopoli, 1786)

Séguy 1930a; Rislorcelli 1941, Rislorcelli 1947, **HA**; Bailly-Choumara et al. 1971, **AP**, **EM**, **MA**, **HA**, **AA**; Abonnenc 1972; Rioux et al. 1974, **HA**; Croset et al. 1978; Mouna 1998; Boussaa et al. 2005; Boussaa 2008; Colacicco-Mayhugh et al. 2010, Mediterranean region; Bounamous 2010; Boussaa et al. 2010; Prudhomme et al. 2012

﻿*Phlebotomus ﻿﻿﻿clydei* Sinton, 1928

Bailly-Choumara et al. 1971, **EM**; Mouna 1998

﻿*Phlebotomus ﻿﻿﻿lewisi* Parrot, 1948

Bailly-Choumara et al. 1971, South **AP**; Mouna 1998

﻿***Sergentomyia* França & Parrot, 1920**

﻿*Sergentomyia (Grassomyia) ﻿dreyfussi* (Parrot, 1933)

Rislorcelli 1941, Rislorcelli 1947, **HA**; Bailly-Choumara et al. 1971, **AP**, **EM**, **MA**, **HA**; Abonnenc 1972; Rioux et al. 1974, **HA**; Croset et al. 1978; Mouna 1998; Boumezzough et al. 2009, **HA**, Marrakech; Bounamous 2010

﻿﻿*Sergentomyia (Parrotomyia) africana* (Newstead, 1912)

= ﻿*Phlebotomus (Parrotomyia) africana* (Newstead), in Rislorcelli 1941: 522, Bailly-Choumara et al. 1971: 454; Rioux et al. 1974: 99

Séguy 1930a (as subspecies of ﻿*minutus* Rondani: 43), **HA**, Marrakech; Rislorcelli 1941 (as subspecies of ﻿*minutus* Rondani: 528), **AA**, Ksar es Souk; Gaud and Laurent 1952 (as subspecies of ﻿*minutus* Rondani: 75), **AP**, Rabat; Bailly-Choumara et al. 1971 (as subspecies of ﻿*minutus* Rondani: 438), **AP**, **AA**; Rioux et al. 1974, **HA**; Croset et al. 1978 (as subspecies of ﻿*minutus* Rondani: 722); Mouna 1998; Boussaa et al. 2005; Boumezzough et al. 2009, **HA**, Marrakech; Bounamous 2010; Boussaa et al. 2010

﻿*Sergentomyia (Sergentomyia) ﻿﻿﻿antennata* (Newstead, 1912)

= ﻿*Phlebotomuscinctus* Parrot & Martin, 1944, in Mouna 1998: 86

= ﻿*Phlebotomus ﻿﻿﻿signatipennis* Newstead, 1920, in Mouna 1998: 86

Bailly-Choumara et al. 1971, **EM**; Rioux et al. 1974, **HA**; Léger et al. 1974, **AA** (south of Morocco); Mouna 1998

﻿*Sergentomyia (Sergentomyia) ﻿fallax* (Parrot, 1921)

Rislorcelli 1947; Gaud 1954; Bailly-Choumara et al. 1971, **AP**, **EM**, **MA**, **AA**; Rioux et al. 1974, **HA**; Mouna 1998; Guernaoui et al. 2005; Boussaa et al. 2005; Boussaa et al. 2007; Boumezzough et al. 2009, **HA**, Marrakech; Boussaa et al. 2010; Bounamous 2010

﻿*Sergentomyia (Sergentomyia) minuta* (Rondani, 1843)

= ﻿*Phlebotomus ﻿﻿﻿minutus* Rondani, in Gaud and Laurent 1952: 75, Mouna 1998: 86

= ﻿*Phlebotomus (Sergentomyia) ﻿parroti* (Adler and Theodor), in Rislorcelli 1941: 526, Rislorcelli 1947: 487, Bailly-Choumara et al. 1971: 450, Rioux et al. 1974, 99

Séguy 1930a, **HA**, Marrakech; Gaud and Laurent 1952, **AP**, Rabat; Rislorcelli 1941; Rislorcelli 1947, **HA**; Bailly-Choumara et al. 1971, **Rif**, **AP**, **EM**, **MA**, **HA**; Rioux et al. 1974, **HA**; Croset et al. 1978 (from the mediterranean region to the Sahara); Mouna 1998; Boussaa et al. 2005; Boumezzough et al. 2009, **HA**, Marrakech; Bounamous 2010; Boussaa et al. 2010; Depaquit et al. 2015, **Rif**, Chefchaouen

﻿*Sergentomyia (Sergentomyia) schwetzi* Adier, Theodor & Parrot, 1929

Bailly-Choumara and Léger 1976, **SA**, Aouinet-Torkoz; Mouna 1998

﻿*Sergentomyia (Sintonius) christophersi* (Sinton, 1927)

Rioux et al. 1974, **HA**; Rioux et al. 1975; Croset et al. 1978; Mouna 1998

##### Psychodinae

Maruinini

﻿***Tonnoiriella* Vaillant, 1982**

﻿*Tonnoiriella ﻿﻿﻿paveli* Ježek, 1999

Ježek 1999, **HA**, **AA**; Afzan and Belqat 2016

﻿*Tonnoiriella ﻿﻿﻿pulchra* (Eaton, 1893) (?) [probably mis-identification]

Wagner 1990; Ježek and Hájek 2007; Afzan and Belqat 2016

##### Mormiini

﻿***Mormia* Enderlein, 1935**

﻿*Mormia ﻿﻿﻿tenebricosa* Vaillant, 1954

= *Telmatoscopus (Mormia) ﻿tenebricosus* Vaillant, in Vaillant 1956b: 244

Vaillant 1956b, **HA**, Imi-N’Ifri; Afzan and Belqat 2016, **Rif**, Oued Achekrade

##### Paramormiini

﻿***Clogmia* Enderlein, 1937**

﻿*Clogmia ﻿﻿﻿albipunctata* (Williston, 1893)

Afzan and Belqat 2016, **Rif**, Douar Kitane, Douar Moukhlata, Oued Mhannech, **AP**, Douar Aoulad Ali (Central Plateau (Coastal region))

﻿***Panimerus* Eaton, 1913**

﻿*Panimerusthienemanni* (Vaillant, 1954)

= ﻿*Panimerus ﻿﻿﻿maynei* (Tonnoir, 1919), in Dakki 1997: 62

Boumezzough and Vaillant 1986b, **HA**, Assif Réghaya; Afzan and Belqat 2016

﻿***Paramormia* Enderlein, 1935**

﻿*Paramormia ﻿﻿﻿ustulata* (Walker, 1856)

Vaillant 1956b, **HA**; Mouna 1998; Ježek and Yağcı 2005; Omelková and Ježek 2012a; Afzan and Belqat 2016, **Rif**, Seguia Barrage Dar Chaoui, Douar Kitane, Oued Jnane Niche

##### Pericomini

﻿***Bazarella* Vaillant, 1964**

﻿*Bazarella ﻿﻿﻿atra* (Vaillant, 1955)

= ﻿*Pericoma ﻿﻿﻿atra* Vaillant, in Vaillant 1956b: 234, 238, 242

Vaillant 1956b, **HA**, Assif Tasouat (M’Goum), Siroua, Imi-N’Ifri, Aguelmous, Sidi Chamarouch, Lac Tamhda (Anremer), Oukaimeden; Boumezzough and Thomas 1987, **HA**, Oued Réghaya (Imlil, 1750 m); Dakki and Himmi 2008; Afzan and Belqat 2016, **Rif**, Oued Inesmane, Oued Madissouka, Aïn Quanquben

﻿***Pericoma* Walker, 1856**

﻿*Pericoma (Pachypericoma) ﻿blandula* Eaton, 1893

Boumezzough and Vaillant 1986b, **HA**; Dakki 1997; Ježek 2004, **Rif**; Ježek and Hájek 2007; Dakki and Himmi 2008, **MA**, Oued Sebou; Omelková and Ježek 2012a; Afzan and Belqat 2016, **Rif**, Oued Taida, Âounsar Aherman, Oued Beni Ouachekradi, Oued Aâyaden, Cascade Ras el Ma

﻿*Pericoma (Pericoma) ﻿barbarica* Vaillant, 1955

Vaillant 1956b, **HA**, M’Goum; Afzan and Belqat 2016, **Rif**, Oued Taida, Douar Taria, Cascade Grotte des pigeons

﻿*Pericoma (Pericoma) ﻿granadica* Vaillant, 1978

Boumezzough and Vaillant 1986b, **HA**; Vaillant and Moubayed 1987; Dakki 1997; Dakki and Himmi 2008, **MA**, Oued Sebou; Afzan and Belqat 2016, **Rif**, Oued Taida, Ametrasse, Oued Farda, Oued Aâyaden, Oued Ras el Ma, **MA**, Aïn Vittel, **HA**, Cascade sur sol cuivreux, Oued Réghaya

﻿*Pericoma (Pericoma) ﻿diversa* Tonnoir, 1920

Vaillant 1978, **HA**; Afzan and Belqat 2016, **Rif**, Cascade Chrafate

﻿*Pericoma (Pericoma) ﻿exquisita* Eaton, 1893

Ježek 2004, **Rif**, **HA**; Afzan and Belqat 2016

﻿*Pericoma (Pericoma) ﻿latina* Sarà, 1954

Vaillant 1955, **HA**; Afzan and Belqat 2016, **Rif**, Cascade Chrafate, Oued Maggou, Nord Village Maggou, Oued Kelâa, Oued Talembote, Oued associé à Dayat Fifi, Oued Tiffert, Oued à 20 km de Fifi, Oued El Kanar, Beni Fenzar

﻿*Pericoma (Pericoma) maroccana* Vaillant, 1955

= ﻿*Pericoma﻿numidicavar.﻿marocana* Vaillant, 1955

Boumezzough and Vaillant 1986b, **HA**, Tissaout; Dakki 1997; Afzan and Belqat 2016, **Rif**, Cascade Chrafate, ruisseau maison forestière; Dakki and Himmi 2008, **MA**, Oued Sebou

﻿*Pericoma (Pericoma) ﻿modesta* Tonnoir, 1922

= ﻿*Pericoma ﻿﻿﻿numidica* Vaillant, in Vaillant 1956b: 236, 237, 240

Vaillant 1956b, **HA**, Assif Tassouat (M’Goum), Lac Tamhda (L’Anremer); Boumezzough and Vaillant 1986b, **HA**; Dakki 1997; Dakki and Himmi 2008, **MA**, Oued Sebou; Afzan and Belqat 2016

﻿*Pericoma ﻿﻿﻿pseudexquisita* Tonnoir, 1940

Afzan and Belqat 2016, **Rif**, Oued Azila

﻿***Pneumia* Enderlein, 1935**

﻿*Pneumia ﻿﻿﻿nubila* (Meigen, 1818)

Afzan and Belqat 2016, **Rif**, Aïn Mâaze

﻿*Pneumia ﻿﻿﻿pilularia* (Tonnoir, 1940)

Ježek 2004; Ježek and Hájek 2007; Omelková and Ježek 2012a

﻿*Pneumia ﻿﻿﻿propinqua* (Satchell, 1955)

Afzan and Belqat 2016, **Rif**, Chrafate, Oued Zarka

﻿*Pneumia ﻿﻿﻿reghayana* (Boumezzough & Vaillant, 1986)

= *Satchelliella ﻿﻿﻿reghayana* Boumezzough & Vaillant, 1986, in Boumezzough and Vaillant 1986b: 238

Boumezzough and Vaillant 1986b, **HA**; Dakki 1997; Dakki and Himmi 2008; Afzan and Belqat 2016

﻿*Pneumia ﻿﻿﻿toubkalensis* Omelková & Ježek, 2012

Omelková and Ježek 2012b, **HA**, Toubkal; Afzan and Belqat 2016, **Rif**, Oued Aâyaden, Aïn Ras el Ma

##### Psychodini

﻿***Philosepedon* Eaton, 1904**

﻿*Philosepedon (Philosepedon) ﻿humeralis* (Meigen, 1818)

Afzan and Belqat 2016, **Rif**, Oued Hachef, Cascade Ras el Ma, Oued El Kanar, 2 km de Douar Assoul, Oued Aâyaden

﻿***Psychoda* Latreille, 1796**

﻿*Psychoda (Logima) ﻿albipennis* (Zettersdedt, 1850)

= ﻿*Logima ﻿﻿﻿albipennis* (Zettersdedt), in Mouna 1998: 86

Mouna 1998

﻿*Psychoda (Psycha) ﻿grisescens* Tonnoir, 1922

Ježek 2004, **Rif**; Afzan and Belqat 2016, **Rif**, Douar Kitane, **MA**, Gîte Aït Ayoub

﻿*Psychoda (Psychoda) ﻿uniformata* Haseman, 1907

Ježek 2004, **Rif**; Afzan and Belqat 2016

﻿*Psychoda (Psychodocha) ﻿cinerea* Banks, 1894

Boumezzough and Thomas 1987, **HA**, Azib Oukaimeden (2730 m); Dakki 1997; Dakki and Himmi 2008; Afzan and Belqat 2016, **Rif**, Oued Tazzarine, Douar Taria, Douar Kitane, Oued Chrafate, Oued Aâyaden, **EM**, Cascade Grotte des Pigeons (Béni Snassen)

﻿*Psychoda (Psychodocha) ﻿gemina* (Eaton, 1904)

Afzan and Belqat 2016, **Rif**, Dayat Fifi, Oued Zarka, Douar Kitane, Oued Aâyaden

﻿*Psychoda (Tinearia) ﻿alternata* Say, 1824

Tonnoir 1920, **HA**, La Maire; Boumezzough and Thomas 1987, **HA**, Oued Réghaya (1740 m), Imlil; Dakki 1997; Dakki and Himmi 2008; Afzan and Belqat 2016, **Rif**, Oued Nakhla, Oued Farda, Oued Ouara, Oued Ametrasse, Oued Chrafate, Douar Derâa, Douar Ihermochene, Douar Ikhlafene, Douar Taria, Douar Idrene, Douar Kitane, Oued 2 km de Douar Assoul, Oued Aâyaden, ruisseau maison forestière, Oued Mhannech, Aïn Sidi Yahia, **MA**, Gîte Aït Ayoub

#### ﻿Scatopsoidea

﻿**﻿SCATOPSIDAE^3^**

K. Kettani, J.P. Haenni

Number of species: **13**. Expected: 30–40

Faunistic knowledge of the family in Morocco: poor

##### Ectaetiinae

﻿***Ectaetia* Enderlein, 1912**

﻿*Ectaetia ﻿﻿﻿clavipes* (Loew, 1846)

= ﻿*Scatopse ﻿﻿﻿clavipes* Loew, in Mouna 1998: 84

Mouna 1998; Haenni and Kettani 2016, **Rif**, Amsa – MISR

##### Psectrosciarinae

﻿***Anapausis* Enderlein, 1912**

﻿*Anapausis* ﻿sp.*

**Rif**, Source Aïn Tissemlal

﻿***Psectrosciara* Kieffer, 1911**

﻿*Psectrosciara* ﻿﻿sp. 1*

**Rif**, Adrou

﻿*Psectrosciara* ﻿﻿sp. 2*

**Rif**, Adrou

##### Scatopsinae

﻿***Coboldia* Melander, 1916**

﻿*Coboldia ﻿﻿﻿fuscipes* (Meigen, 1830)

Haenni and Kettani 2011, **Rif**, Kitane, **AP**, El Jadida, Rabat, **AA**, Souss; Haenni and Kettani 2016, **Rif**, Maggou, Aârkob, Jnane Niche; **Rif** (M’Diq farm) – MISR

﻿***Parascatopse* Cook, 1955**

﻿*Parascatopse* ﻿sp.

Haenni and Kettani 2011, **AA**, Ouarzazate ­– MHNN

﻿***Quateiella* Cook, 1975**

﻿*Quateiella ﻿﻿﻿inexpectata* Haenni, 1988

Haenni and Kettani 2016, **Rif**, Afertane

﻿***Reichertella* Enderlein, 1912**

﻿*Reichertella ﻿﻿﻿geniculata* (Zetterstedt, 1850)

Haenni and Kettani 2016, **Rif**, Jnane Niche

﻿*Reichertella ﻿maroccana* Haenni, 2011

Haenni and Kettani 2011, **HA**, Oukaimeden – RMNH

﻿***Rhegmoclemina* Enderlein, 1936**

﻿*Rhegmoclemina ﻿﻿﻿lunensis* Haenni & Godfrey, 2009

Haenni and Kettani 2011, **Rif**, Boujdad

﻿***Rhexoza* Enderlein, 1936**

﻿*Rhexoza ﻿﻿﻿freyi* (Duda, 1936)

Haenni and Kettani 2011, **AA**, Agadir – MHNN

﻿***Scatopse* Geoffroy, 1762**

﻿*Scatopse ﻿﻿﻿notata* (Linnaeus, 1758)

Mouna 1998; Haenni and Kettani 2016, **Rif**, Onsar Lile – MISR

﻿***Swammerdamella* Enderlein, 1912**

﻿*Swammerdamella ﻿﻿﻿brevicornis* (Meigen, 1830)

Haenni and Kettani 2011, **Rif**, Kitane, Oued Laou, Ketama, **AP**, El Jadida, Essaouira, **HA**, Ouarzazate; Haenni and Kettani 2016, **Rif**, Amsa, Jnane Niche, Ametrasse – MISR

##### New records for Morocco

An undescribed species of ﻿*Anapausis* has been collected in the Rif mountains in 2014 by K. Kettani and will be described elsewhere.

﻿*Anapausis* ﻿sp.

Rif: Forêt Azilane (NPT), Source Aïn Tissemlal, 1255 m, 35°11.67N, 5°15.20W, 7.vi.2014, Sapinière à *Abiesmaroccana* et *Pinus ﻿﻿﻿nigra*, 1♂.

Two undescribed species of ﻿*Psectrosciara* have been collected in the Rif mountains in 2013 by K. Kettani and will be described elsewhere.

﻿*Psectrosciara* ﻿﻿sp. 1

Rif: Taghzout (PNPB), Adrou, 556 m, 35°22.39N, 05°32.28W, chênes-liège, 14.vii–15.viii.2013, 6♂♂.

﻿*Psectrosciara* ﻿﻿sp. 2

Rif: Taghzout (PNPB), Adrou, 556m, 35°22.39N, 05°32.28W, chênes-liège, 14.vii–15.viii.2013, 3♂♂.

#### ﻿﻿PTYCHOPTERIDAE

K. Kettani, R. Wagner

Number of species: **5**. Expected: 8

Faunistic knowledge of the family in Morocco: moderate

##### Ptychopterinae

﻿***Ptychoptera* Meigen, 1803**

﻿*Ptychoptera ﻿﻿﻿albimana* (Fabricius, 1787)

MISR (No locality given)

﻿*Ptychoptera ﻿﻿﻿contaminata* (Linnaeus, 1758)

MISR (No locality given)

﻿*Ptychoptera ﻿﻿﻿lacustris* Meigen, 1830

MISR (No locality given)

﻿*Ptychoptera ﻿﻿﻿paludosa* Meigen, 1804

MISR (No locality given)

﻿*Ptychoptera ﻿﻿﻿scutellaris* Meigen, 1818

MISR (No locality given)

### ﻿Culicoidea

#### ﻿﻿CHAOBORIDAE

K. Kettani, R. Wagner

Number of species: **2**. Expected: 4

Faunistic knowledge of the family in Morocco: poor

##### Chaoborinae

﻿***Chaoborus* Lichtenstein, 1800**

﻿*Chaoborus ﻿﻿﻿crystallinus* (De Geer, 1776)

Dakki 1997: 60

﻿***Mochlonyx* Loew, 1844**

﻿*Mochlonyx ﻿﻿﻿culiciformis* (De Geer, 1776)

Dakki 1997: 60

#### ﻿﻿CULICIDAE

K. Kettani, B. Trari, O. Himmi, M. Dakki

Number of species: **43**. Expected: 60

Faunistic knowledge of the family in Morocco: good

##### Anophelinae

﻿***Anopheles* Meigen, 1818**

﻿*Anopheles (Anopheles) ﻿algeriensis* Theobald, 1903

Viallate 1922, **AP**, Kénitra; Séguy 1930a; Bonjean 1947, **EM**, **MA**; Gaud 1957a, **HA**, north of High Atlas; Guy 1959a, **MA**, Béni Mellal, **HA**; Guy 1959b, **HA**; Guy 1959a, **MA**, Béni Mellal; Bailly-Choumara 1967a, **MA**, Ghorm El Alem; Benmansour et al. 1972, **MA**, Barrage Bin El Ouidane; Bailly-Choumara 1973b, **AP**, Sidi Yahia du Gharb; Metge 1986, **AP**, Casablanca; Trari and Himmi 1987, **AP**; Himmi et al. 1995; Louah 1995, **Rif**, Tahaddart, Schroda; Dakki 1997; Ramdani 1997, **AP**, Skhirat, Casablanca; Trari et al. 2002; Trari et al. 2004b; Himmi 2007, **Rif**, Chefchaouen; El Ouali Lalami et al. 2010 a,b, **MA**, Fès, Boulmane; El Ouali Lalami 2012, **MA**, Fès; Trari 2017, **Rif**, Chefchaouen, **AA**, Tiznit; Trari and Dakki 2017a, **Rif**, Chefchaouen, **AA**, Tiznit; Trari and Dakki 2017b, **AA**, Tiznit; Trari et al. 2017

﻿*Anopheles (Anopheles) claviger* (Meigen, 1804)

Vialatte 1923, **AP**, Kénitra; Séguy 1930a, **AP**, Rabat; Langeron 1938, **HA**, Tounfit, Massou, Anefgou, Tirghist, Tighermine, Louggouargh; Callot 1940, **HA**, Anefgou, Tirghist; Bonjean 1947, **EM**, **MA**; Gaud 1947c, **MA**, Sefrou, Meknès; Gaud 1948, **AP**, Rabat, **MA**, El Hajeb, **AA**, Errachidia, Tadla; Gaud et al. 1948, **AP**, Skhirat; Guy 1963, **Rif**, Taounate, **MA**, Meknès, Ifrane; Bailly-Choumara 1967a, **MA**, piste Tafechna-Taoujgelt, maison forestière Ouiouane, piste Tafechna-Senoual-Itzer, piste Ksiba-Naour, piste Naour-Arbala, Zaouia Cheikh, Oued Sarif (environs El Ksiba), Dayet Aoua (environs Ifrane), Boulemane; Bailly-Choumara 1967b, **EM**, 8 km N Itzer; Bailly-Choumara 1967c, **Rif**, piste Ketama-Mt Tiguidin; Guy 1967, **HA**, Marrakech, **AA**, Tafilalt; Guy and Holstein 1968, **SA**; Metge 1986, **AP**, Casablanca; Himmi et al. 1995; Louah 1995, **Rif**, Haidra, Marina Smir; Dakki 1997; Ramdani 1997, **AP**, Skhirat, Tamaris drains, Tamaris merja; Trari et al. 2002; Trari et al. 2004b; Himmi 2007, **Rif**, Bab Berred, Tanakoub; Faraj et al. 2008b, **Rif**, Assoul, Mizgane; El Ouali Lalami et al. 2010a, **MA**, Boulmane; Larhbali et al. 2010, **MA**, Zhiliga, Boukachmir, Aït Ichou; Trari 2017; Trari and Dakki 2017b; Trari et al. 2017

﻿*Anopheles (Anopheles) labranchiae* Falleroni, 1926

d’Anfreville 1916, **AP**, Salé; Delanoe 1917, **AP**, El Jadida; Viallate 1922, **AP**, Rabat, Boulhaut, Bouznika, Gharb, **MA**, Sidi Kacem, Tiflet, Fès, Taza; Charrier 1924, **Rif**, Tanger; Séguy 1930a, **MA**; Roubaud 1935, **AP**, Rabat; Sicault et al. 1935, **AP**, Merja Ras Eddaoura, Merja Zerga, dayas entre Sebou et Maâmora, Dar bel Hamri (entre barrage El Kansera et Sidi Slimane), à proximité de Merja Zerga, Douar Anabsa; Langeron 1938, **AA**; Callot 1940, **AA**; Ristorcelli 1946a, b, **HA**, Oued Tensift, Oued Issil; Bonjean 1947, **AP**, Gharb; Gaud 1947c, **MA**, Oulmès, **HA**, Marrakech; Gaud et al. 1948, **AP**, Merja Ras Eddaoura; Gaud et al. 1949, **AP**, Salé, **SA**, Foum Zguid, Tagounit; Gaud 1953a, **AP**, from Tanger to El Jadida, **MA**, Tissa, Timhadit, Bekrit, Meknès, Ifrane (1700 m), **HA**, Sidi Aissa, Tizi-n’Tichka; Guy 1958, **HA**, Oued N’fis; Sacca and Guy 1960b, **Rif**, Tétouan, **AP**, Skhirat, Sidi Yahia, Sidi Bettache, Mazagan, Braila (près Sidi Allal Tazi), Aït Lahsen, **MA**, Meknès, **HA**, Marrakech; Guy 1962, **Rif**, Taounate, **AP**, Gharb, **HA**, Marrakech (ville et banlieu); Guy 1963, **Rif**, Tanger, Tétouan, **EM**, Berkane, Debdou, Oujda, **AP**, Kénitra, Souk Larba, Settat, Chamaîa, Safi, Casablanca, Rabat, Essaouira, **MA**, Meknès, Fès, Azrou, Oued Zem, Béni Mellal, **HA**, Kelaâ of Sraghna; Bailly-Choumara 1967a, **MA**, piste Tafechna-Znan Imes, piste Tafechna-Taoujgelt, piste Tafechna-Assoul, Aguelmane Azigza, piste Aguelmane Azigza-Aïn Leuh, maison forestière Ouiouane, route Khénifra-Tafechna, piste Tafechna-Senoual-Itzer, Ajdir, Itzer, Bords Moulouya, piste Itzer-Boumia, RN P.33 Boumia-El Kbab, Ouaoumana, piste Ksiba-Naour, piste Naour-Arbala, piste Arbala-El Kbab, Kafensour, Oued Sarif (environs El Ksiba), Dayet Aoua, Ifrane, Imouzer Marmoucha; Bailly-Choumara 1967b, **EM**, route Itzer-Midelt, Douar Sherba, Douar Aïn Shebbak, Douar Aïn Zabia, Douar Madarh, Douar Sidi Hashas, Mechraa safsaf; Bailly-Choumara 1967c, **Rif**, route Chaouen-Bab Taza, piste Bab Taza-Fifi, piste Bab Taza-Talassemtane, piste Bab Taza-Béni Ahmed, piste Bab Taza-Bab Berred, Anasar, piste Bab Berred-Tamorote, Ketama, route Ketama-Targuist, route nationale Jebha, Al Hoceima Club Med, Béni Bouayache, Targuist et environs; Guy 1967, **HA**, Marrakech, **AA**, Tafilalt; Bailly-Choumara 1968b, **AP**, Larache; Guy and Holstein 1968, **AA**, Ouarzazate; Bailly-Choumara 1970, **Rif**, Tétouan, **EM**, Berkane, **AP**, Larache, Sidi Yahia du Gharb, **HA**, Marrakech; Benmansour et al. 1972, **AA**, Agadir; Bailly-Choumara 1972a, **AP**, Merja Sheishat; Bailly-Choumara 1972b, **AP**, Merja Sheishat; Bailly-Choumara 1973a, **AP**, Merja de l’Oued Smir; Bailly-Choumara 1973b, **Rif**, Tétouan, **EM**, Berkane, **AP**, Merja Sheishat, **HA**, Souk des Oudaias; de Zulueta et al. 1983, **Rif**; Ibn Jilali 1984, **AP**, Maâmora; Metge 1986, **AP**, Entre Oulad Dlim et Al Ara’ra; Trari and Himmi 1987, **AP**, Gharb; Himmi 1991, **AP**, Kénitra, Maâmora; Metge 1991, **AP**, Sidi Bettache; Trari 1991, **Rif**, Chefchaouen, Tanger, Taounate, **EM**, Oujda, **AP**, Sidi Amira, Sidi Boughaba, Merja Zerga, Sidi Allal Tazi, Aïn Chouk, Oued Loukous, Merja Oulad Skhar, **MA**, Khémissat, Béni Mellal, Khouribga, **HA**, El Kelaâ des Sraghrna, **AA**, Ouarzazate; El Bermaki 1993, **AP**, entre l’aeroport Anfa et l’aménagement d’El Oulfa, Sidi Maârouf, El Oulfa; Chlaida and Bouzidi 1995, **AP**, Aïn Blal, Oued Sidi Messoud; Louah 1995, **Rif**, Tres piedras, Marina Smir, Bouzerlal, Oued Maleh, Zekri, Lajour, Azla, Tahaddart, Skroda, Stehat, Moulay Bouchta, Kebbache, Talembote, Loubart, Chefchaouen, Oued Maggou, Bab Berred, Sidi Kankoch, Oued kbir, Oued Jebel Lehbib; Louah et al. 1995; Chlaida 1997, **AP**, Aïn Blal, Oued Sidi Messoud, Douar Chlihat, Barrage Al Massira; Moussalim 1997, **AP**, Sidi Allal Tazi, Maâmora; Ramdani 1997, **AP**, Tamaris, Skhirat; Faraj et al. 1997, **AP**, Ouled Moussa; Handaq 1998, **HA**, Oukaimeden, Amizmiz, Tiguenziouine; Himmi et al. 1998, **AP**, Dayat d’El Menzeh (north-east of Kénitra), Sidi Boughaba; Alaoui Slimani et al. 1999, **AP**, Bou-Regreg Salé, Sidi Bouguettaya, Quartier industriel Takaddoum, Marjane; Alaoui Slimani 2002, **AP**, Rabat-Salé; Trari et al. 2002; Faraj et al. 2003, **Rif**, Azib Bouflou, Azib Jrou, Imzouren, Amezzaourou, Tizi-Tamalout, **MA**, Aït Abdelsalam, Aït Lamfadel; Faraj et al. 2004, **Rif**, Azib Jrou, Tanghaya Akarkar, Amezzaourou, Ouled Nsar, **AP**, Fedalate, **MA**, Talaa Chougaga, Aïn Smen (Fès), Aït Lamfadel; Trari et al. 2004a, **Rif**, Larache; Trari et al. 2004b, **Rif**, Ketama, Gzenaya, Taounate, Bouzaghlal, Oued Laou, Smir (Merja), Béni Hassane, Azib Jrou, Azib Bouflou, **AP**, Laaouamra, Aarabat Sidi Abdelaziz, Moukaouama, Dar Belamri, Laksibia, Mgadid, Beggara, Rabat (Chellah), **MA**, Adouz, Rommani (Khémisset), Aït Abderrahmane, Aït Ishaq, Oulad Messoud, Ouled Fennane, El karma, Oulad Zguida, Oulad Abbou; Aouinty et al. 2006, **AP**, Mohammedia; Himmi 2007, **Rif**, Bab Berred, Bab Taza, Tanakoub, **AP**, Skhirate, Maâmora, Oulja, Bouknadel, Sidi Azzouz, Tamaris, **MA**, Khémisset; Faraj et al. 2008a, **AP**, Laouamra, Boucharen, **MA**, Béni Khlef, Talaa Chougaga; Faraj et al. 2008b, **Rif**, Assoul, Mizgane; El Ouali Lalami et al. 2010b, **MA**, Fès; Faraj et al. 2010, **AP**, Begara, Boucharen, Ben Slimane, Skhirat, Rabat, Sehoul, **MA**, Sidi Allal Msader, Aïn Aghbal, Aïn Elouali, Sidi Kacem; Larhbali et al. 2010, **MA**, Aït Haddou Said; Adlaoui et al. 2011, **AP**, Larache; Larhbali et al. 2011, **MA**, Oulmès, Aït Yadin, Sfassif, Mâaziz, Rommani, Laghoualem, Ezzhiliga, Sidi Allal Bahraoui, Boukachmine, Aït Malek, Sidi Boukhalkhal, Bni Ounzar, Ganzra, Aït Siberne, Sidi Allal Msader, El Ghandour; El Ouali Lalami 2012, **MA**, Pont Diamant vert, Sidi Harazem, Oued El Himmer, Moulay Yakoub, Oued Sebou, Aïn Kansara, Oued Aïn Chkef, Sefrou, Boulemane; Laboudi et al. 2012, **AP**, Larache; Hadji et al. 2013, **AP**, Sidi Slimane; El Joubari et al. 2014, **Rif**, Smir lagoon; Laboudi et al. 2014, **Rif**, Tétouan, Tanger, Chefchaouen, Al Hoceima, **AP**, Larache, Salé, **MA**, Taza, Khémissat; El Joubari et al. 2015a, **Rif**, Smir lagoon; El Joubari et al. 2015b, **Rif**, Smir lagoon; Marc et al. 2016, **AP**, Kénitra; Trari 2017, **Rif**, Chefchaouen, Tétouan, **AP**, Larache, Rabat, Settat, **EM**, Oujda, **MA**, Khémisset, Meknès, Khouribga, **HA**, Marrakech, **AA**, Tiznit, Ouarzazate; Trari and Dakki 2017a, **Rif**, Chefchaouen, Tétouan, **AP**, Larache, Rabat, Settat, **EM**, Oujda, **MA**, Khémisset, Meknès, Khouribga, **HA**, Marrakech, **AA**, Tiznit, Ouarzazate; Trari and Dakki 2017b; Trari et al. 2017

﻿*Anopheles (Anopheles) ﻿marteri* Senevet & Prunnelle, 1927

Gaud 1945b, **MA**, El Hajeb, Khénifra, **HA**, Tizi-n’test, Tillougite; Gaud et al. 1949, **HA**, Tizi-n’test; Gaud 1953b, **HA**, Tizi-n’test; Bailly-Choumara 1967b, **EM**, Grotte du Zegzel; Bailly-Choumara 1967c, **Rif**, route Chaouen-Bab Taza, piste Bab Taza-Béni Ahmed, route Bab Taza-Bab Berred, piste Bab Taza-Asifane, piste Bab Berred-Tamorote, Ketama, route Ketama-Targuist, route nationale Jebha, Boured; Benmansour et al. 1972, **MA**, Taza; Trari 1991, **Rif**, Taounate; Himmi et al. 1995; Dakki 1997; Trari et al. 2002; Himmi 2007; Trari 2017, **Rif**, Chefchaouen, **AP**, Settat; Trari and Dakki 2017a, **Rif**, Chefchaouen, **AP**, Settat; Trari and Dakki 2017b; Trari et al. 2017

﻿*Anopheles (Anopheles) ﻿ziemanni* Grünberg, 1902

Senevet 1935, **MA**; Gaud et al. 1949, **HA**, plain of south and north of the Occidental Atlas; Gaud et al. 1950, **HA**, plain of south and north of the Occidental Atlas; Guy 1958, **HA**, Marrakech; Guy et al. 1958, **HA**, Oued N’fis; Guy 1967, **EM**, Oujda, **AP**, Rabat, **HA**, Marrakech, **AA**, Tafilelt; Bailly-Choumara 1970, **HA**, Marrakech; Benmansour et al. 1972, **MA**, Taza, **HA**, Haouz, Tadla Azilal; Bailly-Choumara 1973b, **HA**, Souk des Oudaias (Souk Tnine des Oudaias, 470 m); Trari 1991, **MA**, Tissa; Moussalim 1997, **AP**, 7.5 km de Sidi Allal Tazi; Trari et al. 2002; Trari et al. 2004b; Himmi 2007; Trari 2017, **Rif**, Tétouan, **AA**, Tiznit; Trari and Dakki 2017a, **Rif**, Tétouan, **AA**, Tiznit; Trari and Dakki 2017b; Trari et al. 2017

﻿*Anopheles (Cellia) ﻿cinereus* Theobald, 1901

Viallate 1922, **MA**, Sefrou, Sidi Lamine, **HA**, Mtougui; Sicault et al. 1935, **AP**, Souk Larba of Gharb; Senevet 1935, **AP**, Souk Larba of Zemmour; Langeron 1938, **HA**, Anefgou, Tirghist, Valley of Sidi Yahia Ouyoussef, Tighermine, Louggouargh, Massou; Callot 1940, **HA**, Anefgou, Tirghist; Gaud 1945a, **Rif**, Meridional Rif, **EM**, Moulouya, **HA**, Marrakech, **AA**, Tansikht, valley de Sous; Gaud and Duthu 1945, **HA**, Marrakech; Viamonte and Ramirez 1945, **Rif**; Viamonte and Ramirez 1946, **Rif**; Ristorcelli 1946a, **HA**, Oued Tensift, Oued Issil; Ristorcelli 1946b, **HA**, Oued Tensift, Oued Issil; Gaud 1945b, **AA**, Tansikht; Gaud et al. 1949, **Rif**, Meridional Rif, **EM**, Moulouya, **HA**, Marrakech, **AA**, Tansikht, valley de Sous; Gaud et al. 1950, **Rif**, Meridional Rif, **EM**, Moulouya, **HA**, Marrakech, **AA**, Tansikht, valley de Sous; Gaud 1953a; Gaud 1958, **HA**, Marrakech; Guy 1962, **Rif**, Taounate, **HA**, Marrakech; Guy 1963, **MA**, Midelt, **AA**, Hamada of Draa; Bailly-Choumara 1965, **EM**, Aman d’Aït Oussa, Tiglit, El Megrinat, Taskala, Aïn Aït delouine, Oued mesdourt, Talmesdourt, Assa, **AA**, Aït Melloul, Oued Teima, Issen, Taroudant, Talaint, Tiznit, Oued Assaka, Anezi, Pont de la route Agadir-Tiznit, valley of low Draa, Tafraoute, Tacharicht, Bou Izakarn, Jemâa N’tirhirte, Aït Erkha,Tazert, Barrage Taourirt, **AA**, Goulmima, **SA**, Aouinet Torkoz, Tirh Mzoun; Bailly-Choumara 1966, **AA**, piste Foum Zguid-Lac Iriqui, Agadir-Tissint, Akka-Iguiren, Tirhem, Taoujgelt, Souk El Khémis Dades, Akka, Aït Ouabelli, Foum-el-Hassan, Tarhjicht, Aït melloul, Taliouine, Tazenakht (Rocade of Draa); Bailly-Choumara 1967a, **MA**, route Azrou-Khénifra, Jnane Imasse, piste Tafechna-Taoujgelt, piste Tafechna-Assoul, Sources Oum-er-Rbia, Itzer, piste Itzer-Boumia, Ouaoumana, piste Ghorm El Alem-El Ksiba, piste Naour-Arbala, Zaouia Cheich, kafensour, Ifrane, Imouzzer Marmoucha; Bailly-Choumara 1967b, **EM**, 7 km N Itzer, 8 km N Itzer, route Itzer-Midelt, Aïn Srouna, Gouttitir, Cascade Oued Za, Grotte du Zegzel, Douar Aïn Soultane, Mechraa Safsaf, Oujda, Berguent (valley of Moulouya), Figuig; Bailly-Choumara 1967c, **Rif**, Chaouen ville, route Chaouen-Bab Taza, Bab Taza, piste Bab Taza-Fifi, piste Bab Taza-Talassemtane, piste Bab Taza-Béni Ahmed, route Bab Taza-Bab Berred, piste Bab Berred-Assifane, Anasar, piste Bab berred-Tamorote, Ketama, route Ketama-Targuist, route nationale Jebha, route Al Hoceima-Arba Taourirt, Arba Taourirt, Targuist et environs, route Targuist Al Hoceima, Jebha, Oued Ouergha, route Aknoul-Al Hoceima; Guy 1967, **AP**, Rabat, **EM**, Oujda, **HA**, Marrakech, **AA**, Tafilalet; Bailly-Choumara 1970, **HA**, Marrakech; Bailly-Choumara 1973b, **HA**, Souk des Oudaias; Trari 1991, **Rif**, Al Hoceima, Chefchaouen, Taounate, **EM**, Nador, Oujda, Figuig, **AP**, Larache, Settat, Ben Slimane, **MA**, Khénifra, Taza, Khouribga, **HA**, Kelaâ of Sraghna, **AA**, Ouarzazate, Goulmima; Bouallam 1992, **HA**, Oued N’fis; Louah 1995, **Rif**, Marina Smir, Bouzerlal, Tahaddart, Stehat, Moulay Bouchta, Kebbache, Loubart, 0ued Maggou; Louah et al. 1995; Bouallam et al. 1997b, **HA**, Marrakech; Handaq 1998, **HA**, Amizmiz, Tiguenziouine; Bouallam 2001, **HA**, Oued N’fis; Trari et al. 2002; Trari et al. 2004b, **Rif**, Ketama, Azib Jrou, Sidi Mokhfi, **MA**, Taghzirt, Aghbala, Aït Shak, Smaala, Mlalih, Ouled Fennane, Béni Khlef, Tachrafte; Faraj et al. 2007, **Rif**, Assoul, Mizgane; Himmi 2007, **Rif**, Bab Berred, Bab Taza, Stehat, Tanakoub; Faraj et al. 2008, **Rif**, Assoul, Mizgane; El Ouali Lalami et al. 2010a, **MA**, Fès; El Ouali Lalami et al. 2010b, **MA**, Oued El Himmer; Larhbali et al. 2010, **MA**, Roumani Aïn Sbite, Jamâa M. B., Ghoualem, Zhiliga, Oulmès, Tarmilate, Boukachmir, Mrirte, Aït Ichou, Mâaziz, Tiddas, Bni Ounzar, Ganzra; El Ouali Lalami 2012, **MA**, Oued El Himmer; Trari 2017, **Rif**, Chefchaouen, Tétouan, **MA**, Khouribga, **AA**, Tiznit; Trari and Dakki 2017a, **Rif**, Chefchaouen, Tétouan, **MA**, Khouribga, **AA**, Tiznit; Trari and Dakki 2017b; Trari et al. 2017; Mouatassem et al. 2019, **MA**, Fès

﻿*Anopheles (Cellia) ﻿dthali* Patton, 1905

Saccà 1960, **AA**, Aoufous, Meski, Erfoud, Agdz, Zogora, Tagounit, Tamsrruth; Guy 1961, **HA**, Sud de Zagora (en bordure Hamada du Draa); Guy 1963, **AA**, Zagora and south of the High Atlas (at edge of Hamada of Draa), Oued Ziz; Bailly-Choumara 1965c, **AA**, Tiznit, **EM**, Aïn Aït Delouine, Aouinet Torkoz, Rich Tamlougout (eastern borders of Jebel Bani); Bailly-Choumara 1966, **AA**, piste Foum Zguid au Lac Iriqui, Agadir-Tissint, Akka-Iguiren, Souk El Khémis Dades, Akka et Environs, Aït Ouabelli, Tarhjicht, piste Tazenakhte à Foum Zguid (Rocade du Draa); Bailly-Choumara 1967b, **EM**, valley of Moulouya; Guy 1967, **HA**, Marrakech, **AA**, Tafilalet; Guy and Holstein 1968, **EM**, N Outat El Haj (valley of Moulouya), Gouttitir (environs de Taourirt); Bailly-Choumara 1970, **AA**, Foum Zquid; Bailly-Choumara 1973b, **AA**, Foum Zquid; Himmi et al. 1995; Dakki 1997; Trari et al. 2002; Trari et al. 2004b; Faraj et al. 2007, **Rif**, Assoul, Mizgane (SE of Bab Berred); Faraj et al. 2008, **Rif**, Assoul, Mizgane (SE Bab Berred); Himmi 2007; Trari 2017; Trari and Dakki 2017b; Trari et al. 2017

﻿*Anopheles (Cellia) ﻿multicolor* Cambouliu, 1902

Messerlin and Treillard 1938, **HA**, Marrakech; Viamonte and Ramirez 1945, **Rif**; Guy 1963, **SA**; Bailly-Choumara 1965c, **EM**, Aït Oussa, Aman d’Aït Oussa, Aïn Aït Delouine, Aouinet Torkoz, Rich tamlougout (Confins orientaux du Jebel Bani), **AA**, Tafnidilt, Guelta Zerga, Aïn Temda (valley of low Draa), Tirhmert (Goulmima), **SA**, Vallée et embouchure de l’Oued Assaka, Tantan ville, Tirh Mzoun; Bailly-Choumara 1966, **AA**, Rocade of Draa; Bailly-Choumara 1967b, **EM**, 40 km N de Outat El Haj, Gouttitir, Cascade Oued Za, Douar Aïn Shebbak (valley of Moulouya); Guy 1967, **HA**, Marrakech, **AA**, Tafilelt; Guy and Holstein 1968, **HA**, south of Atlas, **AP**, plain located between Marrakech and the Atlantic from Tanger along the length of the Mediterranean; Bailly-Choumara 1970, **AA**, Foum Zquid; Bailly-Choumara 1973b, **AA**, Foum Zquid; Metge 1986, **AP**, Casablanca; Trari 1991, **Rif**, Al Hoceima, Taounate, Oued Maleh, Bab Berred, **AP**, Tamaris Merja, **AA**, Ouarzazate; Himmi et al. 1995; Dakki 1997; Trari et al. 2002; Trari et al. 2004b; El Joubari et al. 2014, **Rif**, Smir lagoon; Trari 2017; Trari and Dakki 2017b; Trari et al. 2017

﻿*Anopheles (Cellia) sergentii* (Theobald, 1907)

Séguy 1930a; Messerlin and Treillard 1938, **HA**, Tamelelt; Langeron 1938, **Rif**, Targhist; Callot 1940, **AA**, Taghjicht; Gaud 1947c, **AA**, Wadi Draa; Gaud et al. 1949, **Rif**, Zoumi, **HA**, Zaouia Sidi Hamza, Tizi-n’test (1700 m), Tillougit (1800 m); Gaud et al. 1950, **Rif**, Zoumi, **MA**; Guy et al. 1958, **HA**, Oued N’fis; Guy 1961, **Rif**, **AP**, south of Casablanca, **AA**, Sud de Zagora; Guy 1962, **Rif**, **AP**, south of Casablanca, **HA**, Marrakech; Guy 1963, **Rif**, Tanger, **EM**, Berkane, **AP**, south of Casablanca, **MA**, Béni Mellal, **HA**, Oued Tensift, Oued Ziz, Marrakech, Chichaoua, **AA**, Oued Draâ, Oued Dades, Goulmima, sud de Zagora, **SA**, Foum Zguid; Bailly-Choumara 1965c, **EM**, iglit, Aman d’Aït Oussa, Oued Isker, El Megrinat, Aïn Aït Delouine, Oued Mesdourt, almesdourt, Aouinet Torkoz, Bouanama, Rich Tamlougout, Assa, **AA**, Tiznit, Tafraoute, Oued Izi, Bou Izakarn, Abeino (region of Goulmima), **SA**, Aouzeroual, Tirhmert, Tacharicht, Jebel Bani, Tantan; Bailly-Choumara 1966, **AA**, Rocade of Draa, Taliouine; Bailly-Choumara 1967a, **Rif**, **AP**, south of Casablanca, **MA**, Itzer, Ghorm El Alem, Zaouia Cheikh, Kafensour, **HA**, north of High Atlas; Bailly-Choumara 1967b, **EM**, Aïn Srouna, Grotte du Zegzel, Douar Mardarh, Douar Aïn Soultane, Merja Boubker, Selouane, Driouch (valley of Moulouya); Bailly-Choumara 1967c, **Rif**, route nationale Jebha, Targuist-Béni Boufrah, Al Hoceima, Béni Bouayache, Marchica, Had El Rouadi, Pont du Srah; Guy 1967, **HA**, Marrakech, **AA**, Tafilelt; Guy and Holstein 1968, **AP**, Casablanca; Bailly-Choumara 1970, **EM**, Berkane, **HA**, Marrakech; Bailly-Choumara 1973b, **EM**, Berkane, **HA**, Marrakech; Metge 1986, **AP**, Casablanca; Trari 1991, **Rif**, Al Hoceima, Taounate, **AP**, Larache, **AA**, Ouarzazate; Himmi et al. 1995; Dakki 1997; Trari et al. 2002; Faraj et al. 2003, **Rif**, Al Hoceima, Chefchaouen, Taounate, **HA**, Khouribga; Trari et al. 2004b, **Rif**, Ketama, Sidi Mokhfi; Faraj et al. 2007, **Rif**, Assoul, Mizgane; Faraj et al. 2008, **Rif**, Assoul, Mizgane; El Ouali Lalami et al. 2010a, **MA**, Fès; Larhbaliet al. 2010, **MA**, Oulmès, Ganzra; Trari 2017, **Rif**, Chefchaouen, Tétouan, **EM**, Oujda, **MA**, Khouribga; Trari and Dakki 2017a, **Rif**, Chefchaouen, Tétouan, **EM**, Oujda, **MA**, Khouribga; Trari and Dakki 2017b; Trari et al. 2017; Benabdelkrim Filali et al. 2018; Mouatassem et al. 2019, **MA**, Fès

##### Culicinae

Aedini

﻿***Aedes* Meigen, 1818**

﻿*Aedes (Acartomyia) mariae* (Sergent & Sergent, 1903)

Séguy 1930b; Messerlin 1938, **AP**, Rabat; Séguy 1930a, **Rif**, littoral méditerranéen; Bailly-Choumara 1967b, **Rif**, Al Hoceima; Bailly-Choumara 1967c, **Rif**, Al Hoceima; Bailly-Choumara 1968a, **Rif**, Al Hoceima, **AP**, Larache, Sidi Yahia, Sidi Allal Tazi, Rabat, **HA**, Marrakech, **AA**, Tiznit; Himmi et al. 1995; Mestari 1997, **AP**, Mohammedia; Moussalim 1997, **AP**, Sidi Allal Tazi; Trari et al. 2002; Trari 2017; Trari and Dakki 2017b; Trari et al. 2017

﻿*Aedes (Aedimorphus) ﻿vexans* (Meigen, 1830)

Gaud 1947c, **AP**, Sidi Allal Tazi, **MA**, Khémisset; Metge 1986, **AP**, Littoral Casablanca; Himmi et al. 1995; Handaq 1998, **AP**, Gharbia; Dakki 1997; Trari et al. 2002; El Ouali Lalami et al. 2010a, **MA**, Fès; Trari 2017; Trari and Dakki 2017b; Trari et al. 2017

﻿*Aedes (Dahliana) echinus* (Edwards, 1920)

Séguy 1924; Séguy 1930a; Gaud 1953a, **AP**, Rabat, Sidi Yahia, **MA**, Moulay Bouazza, Taza, Fès, Meknès, Ifrane; Bailly-Choumara 1965b, **AP**, Maâmora; Bailly-Choumara 1967c, **Rif**, piste Bab Taza-Talassemtane; Himmi et al. 1995; Trari et al. 2002; Nikookar et al. 2010; El Joubari et al. 2014; Trari 2017; Trari and Dakki 2017b; Trari et al. 2017

﻿*Aedes (Dahliana) ﻿geniculatus* (Olivier, 1791)

Séguy 1924, **MA**; Séguy 1930a; Metge and El Alaoui 1987, **AP**, Subéraies de Béni Abid-Benslimane (Casablanca); Metge and Belakoul 1989, **AP**, Sidi Bettache; Himmi et al. 1995; El Ouali Lalami et al. 2010a, **MA**, Fès Boulmane; Trari et al. 2002; Trari 2017; Trari and Dakki 2017b; Trari et al. 2017

﻿*Aedes (Ochlerotatus) ﻿berlandi* (Séguy, 1921)

Séguy 1930a, **AP**, Rabat; Gaud 1953a, **MA**, Fès; Bailly-Choumara 1967a, **MA**, Jnane Imasse, piste Tafechna-Taoujgelt; Bailly-Choumara 1967c, **Rif**, piste Bab Taza-Béni Ahmed; Belakoul 1985, **AP**, Benslimane, Sidi Bettache; Metge and El Alaoui 1987, **AP**, Casablanca; Metge and Belakoul 1989, **AP**, Benslimane, Sidi Bettache; Trari et al. 2002; Trari 2017; Trari and Dakki 2017b; Trari et al. 2017

﻿*Aedes (Ochlerotatus) caspius* (Pallas, 1771)

Séguy 1930b; Viamonte et Ramirez 1946, **AP**, Larache; Gaud 1952, **AP**, Rabat, Casablanca; Gaud 1953a, **AP**, Rabat, Casablanca; Senevet and Andarelli 1954, **EM**, Embouchure de la Moulouya, Figuig, **AP**, Jorf Lasfar, Mohammedia, Rabat, **MA**, Meknès, Fès, Taza, **HA**, Marrakech, Midelt, **AA**, Tiznit; Bailly-Choumara 1966, **AA**, environs de Tiznit; Bailly-Choumara 1967a, **EM**, Bords Moulouya (près Itzer), Cherarba; Bailly-Choumara 1967b, **EM**, Cherarba, Aïn Shebbak, Saidia, Berguent; Bailly-Choumara 1967c, **Rif**, piste Al Hoceima-Arba Taourirt; Bailly-Choumara 1972a, **AP**, Merja Sheishat; Bailly-Choumara 1973b, **Rif**, Merja de l’Oued Smir; El Kaim 1972, **AP**, Bou-Regreg; Rioux et al. 1975, **AP**, Rabat-Salé; Metge 1986, **AP**, Littoral casablancais; Himmi 1991, **AP**, Sidi Boughaba; Trari 1991, **AP**, Sidi Boughaba, Merja Zerga, Oued Loukous; Himmi et al. 1995; Mestari 1997, **AP**, Mohammedia; Moussalim 1997, **AP**, Sidi Allal Tazi, Kénitra; Ramdani 1997, **AP**, Tamaris Merja; Handaq 1998, **AP**, Zemamra, B. Iffou (Entre El Oualidia et Youssoufia), **MA**, Béni Mellal, **HA**, Marrakech, Zaouiet Ben Sassi, Bengrir; Himmi et al. 1998, **AP**, Sidi Boughaba; Trari et al. 2002; Himmi 2007, **AP**, Sidi Boughaba; Alaoui Slimani 2002, **AP**, Rabat; Aouinty et al. 2006, **AP**, Mohammedia; El Joubari et al. 2014, **Rif**, Smir lagoon; El Joubari et al. 2015a, **Rif**, Smir lagoon; Trari 2017; Trari and Dakki 2017b; Trari et al. 2017

﻿*Aedes (Ochlerotatus) coluzzii* Rioux, Guilvard & Pasteur, 1998 et ﻿*Aedes (Ochlerotatus) detritus* (Haliday, 1833) [Complexe ﻿detritus]

Charrier 1924, **Rif**, Tanger; Séguy 1930a; Gaud 1953a, **EM**, Saidia, **AP**, Kénitra, Rabat, Bouznika, El jadida, Oualidia, **HA**, Marrakech, **AA**, Agadir, Tafnidilt; Bailly-Choumara 1965c, **EM**, Aïn Aït delouine, **SA**, Tirhmert; Bailly-Choumara 1970, **Rif**, Tétouan; Knight 1971, **AP**, Kénitra; El Kaim 1972, **AP**, Bou-Regreg; Bailly-Choumara 1973b, **Rif**, Merja de l’Oued Smir; Rioux et al. 1975, **AP**, Rabat; Pasteur et al. 1978, **AP**, Bou-Regreg; Metge 1986, **AP**, Littoral casablancais; Himmi 1991, **AP**, Sidi Boughaba; Trari 1991, **AP**, Sidi Boughaba, Merja Zerga, Oued Loukous; Louah 1995, **Rif**, Tres piedras, Cabo Negro, Lajour, Azla, Tahaddart; Himmi et al. 1995; Louah et al. 1995; Mestari 1997, **AP**, Mohammedia; Moussalim 1997, **AP**, Sidi Allal Tazi, Rabat; Ramdani 1997, **AP**, Tamaris Merja; Handaq 1998, **AP**, Essaouira, Zima-Chemaîa; Himmi et al. 1998, **AP**, Sidi Boughaba; Himmi 2007, **AP**, Sidi Boughaba; Alaoui Slimani 2002, **AP**, Rabat; Trari et al. 2002; El Joubari et al. 2014, **Rif**, Smir lagoon; El Joubari et al. 2015a, **Rif**, Smir lagoon; Trari 2017; Trari and Dakki 2017b; Trari et al. 2017

﻿*Aedes (Ochlerotatus) ﻿pulchritarsis* (Rondani, 1872)

Gaud 1953a, **AP**, Benslimane, Sidi Yahia, Rabat, **MA**, Oued Zem, Khénifra, Fès; Metge and El Alaoui 1987, **AP**, Benslimane; Himmi et al. 1995; Trari et al. 2002; Trari 2017; Trari and Dakki 2017b; Trari et al. 2017

﻿*Aedes (Rusticoidus) ﻿rusticus* (Rossi, 1790)

Viamonte and Ramirez 1946, **Rif**, Tétouan, **AP**, Larache; Gaud 1953a, **MA**, Taza; Himmi et al. 1995; Handaq 1998, **HA**, Bengrir; Trari et al. 2002; El Ouali Lalami et al. 2010a, **MA**, Fès; Trari 2017; Trari and Dakki 2017b; Trari et al. 2017

﻿*Aedes (Stegomyia) aegypti* (Linnaeus in Hasselquist, 1762)

d’Anfreville 1916, **AP**, Salé; Vialatte 1923, **AP**, Rabat, Casablanca, **HA**, Marrakech; Charrier 1924, **Rif**, Tanger; Gaud 1953a, **AP**, Salé, **HA**, Marrakech; Himmi et al. 1995; Dakki 1997; Handaq 1998, **HA**, Bengrir; Trari et al. 2002; Trari 2017; Trari and Dakki 2017b; Trari et al. 2017

﻿*Aedes (Stegomyia) albopictus* (Skuse, 1895)

Bennouna et al. 2016, **AP**, Agdal (Rabat); Trari 2017; Trari and Dakki 2017b; Trari et al. 2017; Faraj et al. 2018; Amraoui et al. 2019

##### Culicini

﻿***Culex* Linnaeus, 1758**

﻿*Culex (Barraudius) ﻿modestus* Ficalbi, 1889

Séguy 1930a; Bailly-Choumara 1968a, **AP**, Larache; Trari 1991, **AP**, Gharb; Himmi et al. 1995; Dakki 1997; Handaq 1998, **HA**, Bengrir; Trari et al. 2002; Himmi 2007, **Rif**, Bab Berred, **AP**, Maâmora; Hadji et al. 2013, **AP**, Sidi yahia du Gharb, Kcebia, Sidi Hagouch (Sidi Slimane); Trari 2017; Trari and Dakki 2017b; Trari et al. 2017

﻿*Culex (Culex) ﻿brumpti* Galliard, 1931

Bailly-Choumara 1968a, **AP**, Merja Bokka, Larache, **HA**, Marrakech; Bailly-Choumara 1972a, **AP**, Merja Sheishat; Himmi et al. 1995; Dakki 1997; Himmi 2007; Trari et al. 2002; El Ouali Lalami et al. 2010a, **MA**, Fès; El Joubari et al. 2014, **Rif**, Smir lagoon; Trari 2017; Trari and Dakki 2017b; Trari et al. 2017

﻿*Culex (Culex) ﻿laticintus* Edwards, 1913

Charrier 1924, **Rif**, Tanger; Callot 1940, **AA**, Goulmima (mares); Gaud 1953a, **HA**, Marrakech, **AA**, Agadir; Gaud 1957a, **EM**, Nador; Bailly-Choumara 1965c, **EM**, Oued Isker, Aïn Aït delouine, Talmesdourt, **AA**, Ouled Teima, ounaamane, Bou Izakarn, Agunil Khnufa, Akka-guiren; Bailly-Choumara 1966, **AA**, Akka-Iguiren, Tirherm, Taoujgelt, Aït Ouabelli, Anamere-Smougue, Aït melloul, Tiznit (Rocade de Draa); Bailly-Choumara 1967b, **Rif**, Béni Bouayache, Targuist et environs, **EM**, Grotte du Zegzel; Himmi et al. 1995; Dakki 1997; Trari et al. 2002; Himmi 2007, **AP**, Skhirat; Faraj et al. 2008b, **AP**, Louamra; Hadji et al. 2013, **AP**, Sidi Hagouch (Sidi Slimane); Trari 2017; Trari and Dakki 2017b; Trari et al. 2017

﻿*Culex (Culex) ﻿mimeticus* Noè, 1899

Séguy 1930a; Viamonte and Ramirez 1946, **Rif**, Béni Ider, Fnideq, Khemis Anjra, Ketama, Oued Amsa, Oued Krikra, Oued Martil, Oued Laou; Gaud 1953a, **Rif**, Ouezzane, Ghafsai, **EM**, Berkane, Martinpray (près Berkane), El Aïoun Sidi Mellouk, **AP**, Tamri, **MA**, Meknès, Fès, Ifrane, Taza, Béni Mellal, **HA**, Midelt, Marrakech, Azilal, **AA**, Tinghir, Tichka; Guy et al. 1958, **HA**, Oued N’fis; Bailly-Choumara 1965c, **AA**, Oued Noun, Anezi, Tafraoute; Bailly-Choumara 1966, **AA**, Agadir; Bailly-Choumara 1967a, **MA**, route Azrou-Khénifra, piste Tafechna-Taoujgelt, Source Oumerrbia, Ghorm El Alem, piste Ksiba-Naour, piste Naour-Arbala, Zaouia Cheikh, Oued Sarif; Bailly-Choumara 1967b, **EM**, 7 km N d’Itzer, 8 km d’Itzer; Bailly-Choumara 1967c, **Rif**, route Chaouen-Bab Taza, Bab Taza, piste Bab Taza-Fifi, piste Bab Taza-Talassemtane, piste Bab Taza-Béni Ahmed, route Bab Taza-Bab Berred, piste Bab Taza-Asifane, piste Bab Berred-Tamorote, Ketama, piste Ketama-Jebel Tidighine, route Ketama-Targuist, route nationale Jebha, Al Hoceima, Béni Bouayache, Marchica, Targuist et environs, Jebha, Boured; Trari 1991, **AP**, Sidi Yahia du Gharb; Himmi et al. 1995; Louah 1995, **Rif**, Riffien, Tres piedras, Marina Smir, Bouzaghlal, M’diq, Oued Maleh, Azla, Tahaddart, Moulay Bouchta, Schroda, Kebbache, Talambote, Oued Maggou; Louah et al. 1995; Chlaida 1997, **AP**, Oued Sidi Messoud, Aïn Blal, Douar Chlihat, Barrage Al Massira; Chlaida and Bouzidi 1995, **AP**, Barrage El Massira; Dakki 1997; Ramdani 1997, **AP**, Skhirat, Tamaris Merja; Handaq 1998, **HA**, Oukaimeden, Amizmiz, Tiguenziouine (près Oued N’fis); Trari et al. 2002; Himmi 2007, **Rif**, Bab Berred, **AP**, Bouknadel, Douar jdid (Skhirat); El Ouali Lalami et al. 2010a, **MA**, Fès; Larhbali et al. 2010, **MA**, Oulmès; Trari 2017; Trari and Dakki 2017b; Trari et al. 2017

﻿*Culex (Culex) ﻿perexiguus* Theobald, 1903

Séguy 1930a; Callot 1940, **AA**, Assa; Senevet and Andarelli 1959a, **EM**, Oujda, Taourirt, **AP**, Aïn el Aouda, Arbaoua, Had Kourt, Oued Beht, Rabat, Allal Tazi, Oued Sahli, Zaouia Ech cheikh, Taghzirt, **MA**, Béni Mellal, Foum Zabel, Ifrane, Meknès, Aït Atta du Rteb, Fès, Sidi Mokhfi, Tahala, **HA**, Tazert, Midelt, **AA**, akka, Tamri; Bailly-Choumara 1965c, **EM**, Assa; Bailly-Choumara 1966, **AA**, Souk El Khémis Dades, Aït Ouabelli, Tarhjicht, Tirherm, Taoujgelt, Aït melloul; Bailly-Choumara 1967a, **MA**, piste Tafechna-Taoujgelt, piste Tafechna-Assoul, Ajdir; Bailly-Choumara 1967b, **EM**, Madagh, Merja Boubker, Aïn Béni Mathar; Bailly-Choumara 1967c, **Rif**, piste Bab Taza-Fifi, piste Bab Taza-Talassemtane, piste Bab Taza-Béni Ahmed, route Bab Taza-Bab Berred, Anasar, piste Bab Berred-Tamorote, Ketama, route Ketama-Targuist, route nationale Jebha, Béni Bouayache, Targusit et environs; Bailly-Choumara 1972a, **AP**, Merja Sheishat; Louah 1995, **Rif**, Riffien, Tres piedras, Marina Smir, Bouzeghlal, Oued Maleh, Tahaddart, Talembote, Schroda, Tanger; Louah et al. 1995; Mestari 1997, **AP**, Mohammedia; Moussalim 1997, **AP**, Sidi Allal Tazi, El Oulja, Fouarate; Ramdani 1997, **AP**, Skhirat, Tamaris; Handaq 1998, **HA**, Marrakech, entre Oued N’fis et Chichaoua, Kelaâ Sraghna; Alaoui Slimani 2002, **AP**, Rabat, Salé; Trari et al. 2002; Himmi 2007, **Rif**, Bab Berred, Bab Taza; Faraj et al. 2008c, **AP**, Larache Louamra; El Ouali Lalami et al. 2010a, **MA**, Fès; Trari 2017; Trari and Dakki 2017b; Trari et al. 2017; Mouatassem et al. 2019, **MA**, Fès

﻿*Culex (Culex) pipiens* Linnaeus, 1758

d’Anfreville 1916, **AP**, Salé; Charrier 1924, **Rif**, Tanger; Séguy 1930a; Callot 1940, **SA**, Goulimine; Viamonte and Ramirez 1946, **Rif**, Boudinar, Dar Benkarrich, Tétouan, Tanger, Asilah, Ksar El Kébir, Chefchaouen, Ketama, Nador; Gaud 1952, **AP**, Gharb; Séguy 1953a, **SA**, Tindouf; Guy 1958, **HA**, Oued N’fis; Guy et al. 1958, **HA**, Oued N’fis; Bailly-Choumara 1965c, **EM**, Aouinet Aït Oussa, Aïn Isker, Aïn Aït Delouine, Oued Mesdourt, Talmesdourt, Toudi, **AA**, Aït Onmar, Oulad Teima, Taroudant, Tiznit ville, Talaint, Hassi Tafnidilt, Zaouiat Cheikh, Aïn Guerzim, Tafraout ville, **SA**, Goulimine ville, Vallée de l’Oued Assaka, Ouaroun, Zriouila, Labyar, Tighmert, Abeino, Tantan ville, Zag; Bailly-Choumara 1966, **AA**, piste d’Akka au Draa, Akka et environs, Aït Ouabelli, Anamere-Smougue, Tarhjicht, Aït Melloul, Tiznit et environs; Bailly-Choumara 1967a, **MA**, route Azrou-Khénifra, piste Tafechna-Taoujgelt, piste Tafechna-Assoul, maison forestière Ouiouane, piste Tafechna-Senoual-Itzer, Ajdir, Itzer, piste Itzer-Boumia, Ghorm El Alem, Ghorm El Alem-El Ksiba, El Ksiba, piste Ksiba-Naour, Zaouia Cheikh, Oued Sarif, Dayet Aoua, Pont Tarmilate, Ifrane, Imilchil; Bailly-Choumara 1967b, **EM**, route Itzer-Midelt, Gaada de Debdou, Guercif ville, Cascade Oued Za, Grotte du Zegzel, Environs Saidia, Douar Aïn Shebbak, Douar Aïn Zabia, Douar Mardarh, Douar Sidi Hashas, Saidia, Merja Boubker, Berguent, Tendrara, Figuig; Bailly-Choumara 1967c, **Rif**, Chaouen ville, route Chaouen-Bab Taza, piste Bab Taza-Talassemtane, piste Bab Taza-Béni Ahmed, route Bab Taza-Bab Berred, Anasar, piste Bab Berred-Tamorote, Ketama, route Ketama-Targuist, route nationale Jebha, Al Hoceima, Al Hoceima Club Med, Béni Bouayache, Marchica, Targuist et environs, Jebha, Ghafsai; Bailly-Choumara 1972a, **AP**, Merja Sheishat; Bailly-Choumara 1973b, **AP**, Merja Bokka, Merja Qodiya; Metge and Belakoul 1989, **AP**, Benslimane, Sidi Bettache; Himmi 1991, **AP**, Sidi Boughaba, Sidi Amira; Trari 1991, **AP**, Maâmora, El Menzeh, Sidi Boughaba, Chkaïfien, Sidi Yahia du Gharb, Bokka, Merja Zerga, Sidi Allal Tazi, Oued Loukous, Aïn Chouk, Merja Bargha, Merja Oulad Skhar; Bouallam and Ramdani 1992, **HA**, Marrakech; El Bermaki 1993, **AP**, Sidi Maârouf; Himmi et al. 1995; Louah 1995, **Rif**, Fnideq, Riffien, Tres piedras, Marina Smir, M’diq, Cabo Negro, Zekri, Azla, Tahaddart, Schroda, Kebbache, Ouadras, Punta cirres, Sidi Kankoch, Oued Kbir; Louah et al. 1995; Bouallam et al. 1997b, **HA**, Marrakech; Dakki 1997; Mestari 1997, **AP**, Mohammedia; Himmi et al. 1998, **AP**, Sidi Boughaba, Puits de Sidi Amira (forest of Maâmora); Moussalim 1997, **AP**, El Oulja, Maâmora, Fouarate, Sidi Boughaba; Ramdani 1997, **AP**, meseta côtière (Témara-Casablanca); Faraj et al. 1997, **AP**, Kénitra; Handaq 1998, **AP**, Zemamra, **MA**, Oued Zem, **HA**, Marrakech, Zaouiet Ben Sassi, Sidi Bou Othmane, Kelaâ Sraghna, Bengrir; Bouallam 2001, **HA**, Marrakech; Alaoui Slimani 2002, **AP**, Rabat; Trari et al. 2002; Aouinty et al. 2006, **AP**, Mohammedia; Faraj et al. 2006, **AP**, Salé; Himmi 2007, **Rif**, Bab Berred, Bab Taza, Stehat, **AP**, Skhirate, forest of Maâmora, forest of Hilton, Sidi Boughaba, El Oulja, Ouled Salem, Ouled dlim, Douar Ould Yahia Ben Ali, Larouaza, Douar Elarja, Douar Jdid, Douar Jnaja; Faraj et al. 2008b, **AP**, Louamra; El Ouali Lalami et al. 2010a, **MA**, Fès; Larhbali et al. 2010, **MA**, Oulmès, Tarmilate, Bni Ounzar, Ganzra;Louali Lalami et al. 2010b, **MA**, Oued El Himmer; Amraoui 2012, **HA**, Marrakech; Amraoui et al. 2012, **Rif**, Tanger, **AP**, Mohammedia, Casablanca, **HA**, Marrakech; Amraoui et al. 2012, **AP**, Mohammedia, Casablanca; El Ouali lalami 2012, **MA**, route de Sidi Harazem; Hadji et al. 2013, **AP**, Sidi Slimane; El Joubari et al. 2014, **Rif**, Smir lagoon; El Joubari et al. 2015a, **Rif**, Smir lagoon; Marc et al. 2016, **AP**, Kénitra; Trari 2017; Trari and Dakki 2017b; Trari et al. 2017; Bkhache et al. 2018, **Rif**, Tanger, **AP**, Rabat, Mohammedia, **HA**, Marrakech; Tmimi et al. 2018, **AP**, Mohammedia; Mouatassem et al. 2019, **MA**, Fès

﻿*Culex (Culex) ﻿simpsoni* Theobald, 1905

Callot 1940, **AA**, Taghicht; Senevet et al. 1949, **AA**, Oued Noun, Tafnidelt, Akka; Gaud 1953a, **AA**, Imsouane, Aït Melloul, Akka, O’Noun, Taghjicht, Assa, Tafnidilt; Bailly-Choumara 1965c, **EM**, Aman d’Aït Oussa, El Megrinat, **AA**, Oued Izi, Oued Massa-Pont de la route Agadir-Tiznit, Guelta Zerga, Tafnidilt, **SA**, Poste militaire de Boujrif, Tirhmert, Taourirt-Barrage, Aouinet Torkoz; Bailly-Choumara 1966, **AA**, Tirherm, Taoujgelt, Taghjicht, Aït melloul, Tiznit; Chlaida and Bouzidi 1995, **AP**, Sidi M’barek, Oued Sidi Messoud, Mechrâa, Sidi Boulâarais, Douar Chlihat (south of Settat); Chlaida 1997, **AP**, Sidi M’barek, Mechrâa Settir, Sidi Boulâarais, Douar Chlihat (south of Settat); Dakki 1997; Handaq 1998, **HA**, Sidi Bou Othmane (Marrakech); Trari et al. 2002; Himmi 2007, **Rif**, Bab Berred, **MA**, Khémisset, **AA**, sud Anti Atlas; El Ouali Lalami et al. 2010a, **MA**, Fès; Trari 2017; Trari and Dakki 2017b; Trari et al. 2017

﻿*Culex (Culex) ﻿theileri* Theobald, 1903

Callot 1940, **AA**, Taghjicht; Viamonte and Ramirez 1946, **Rif**, Dar Benkarrich, Boudinar, Ketama, Tanger, Tétouan, Chefchaouen, **AP**, Larache; Guy 1958, **HA**, Oued N’fis; Guy et al. 1958, **HA**, Oued N’fis; Bailly-Choumara 1965c, **EM**, Aouinet Aït Oussa, Aïn Oumesdour, Aouinet Torkoz, **SA**, Tirh Mzoun; Bailly-Choumara 1966, **AA**, Souk El Khémis Dades, Akka et environs, Aït Melloul, Tazenakhte, piste Tiznit-Tafraout, **SA**, Goulimine; Bailly-Choumara 1967a, **MA**, piste Tafechna-Taoujgelt, piste Tafechna-Assoul, Aguelmane Azigza, piste Aguelmane Azigza-Aïn Leuh, maison forestière Ouiouane, Khénifra, piste Tafechna-Senoual-Itzer, Ajdir, Itzer, Col du Zad, piste Itzer-Boumia, Ghorm El Alem, piste Ghorm El Alem-El Ksiba, El Ksiba, piste Ksiba-Naour, Aguelmane Moulay Yakoub, Zaouia Cheich, Kafensour, Dayet Aoua, Pont Tarmilate; Bailly-Choumara 1967b, **EM**, route Itzer-Midelt, 40 km N Outat El Haj, Taourirt, Driouch, Figuig; Bailly-Choumara 1967c, **Rif**, piste Bab Taza-Fifi, piste Bab Taza-Talassemtane, route Bab Taza-Bab Berred, Anasar, piste Bab Berred-Tamorote, Ketama, route Ketama-Targuist, ArbaaTaourirt, Al Hoceima, Targuist et environs, Ghafsai; Bailly-Choumara 1972a, **AP**, Merja Sheishat; Bailly-Choumara 1973b, **AP**, Merja Sheishat, Merja Bokka; Himmi 1991, **AP**, Sidi Boughaba, El menzeh, Sidi Amira; Trari 1991, **AP**, Maâmora, Oued Sebou, Bordure Oued Loukous, Sidi Yahia du Gharb, Bokka, Entre Moulay Bousselham et Larache, Merja Zerga, Larache; El Bermaki 1993, **AP**, Sidi Maârouf, El Oulfa; Louah 1995, **Rif**, Bouzeghlal, M’Diq, Oued Maleh, Zekri, Lajour, Tahaddart, Loubart, Chefchaouen, Tanger; Himmi et al. 1995; Louah et al. 1995; Dakki 1997; Mestari 1997, **AP**, Mohammedia; Moussalim 1997, **AP**, Sidi Allal Tazi, Maâmora, Rabat, Kénitra; Ramdani 1997, **AP**, Skhirat, Tamaris; Handaq 1998, **AP**, Zemamra, **MA**, Béni Mellal, **HA**, Oukaimeden, Amizmiz, Tiguenziouine, Marrakech, Sidi Bou Othmane, Seguarta-Kelaâ, Had Mhara, Aîn Äounate, Bengrir; Himmi et al. 1998, **AP**, Sidi Boughaba, Dayat d’El Menzeh (north east of Kénitra), Gharb; Alaoui Slimani 2002, **AP**, Rabat; Trari et al. 2002; Himmi 2007, **Rif**, Bab Berred, Tanakoub, **AP**, Skhirate, forest of Maâmora, Chiahna, Sidi Amira, Sidi Boughaba, Bouknadel, Sidi Azzouz, Ehssaïne, Bettana, Sidi Yahia, Sebbah, Larouaza, Douar Elarja, Douar Jdid, Douar Jnaja; Faraj et al. 2008b, **AP**, Larache Louamra; El Ouali Lalami et al. 2010a, **AP**, Boucharen;Larhbali et al. 2010, **MA**, Roumani, Aïn Sbite, Ezzhiliga, Oulmès, Tarmilate, M’rirt, Bni Ounzar, Ganzra (Khémisset); Hadji et al. 2013, **AP**, Sidi yahia du Gharb, Kcebia, Sidi Hagouch, Dar Belamri (Sidi Slimane); El Joubari et al. 2014, **Rif**, Smir lagoon; Trari 2017; Trari and Dakki 2017b; Trari et al. 2017; Mouatassem et al. 2019, **MA**, Fès

﻿*Culex (Maillotia) ﻿deserticola* Kirkpatrick, 1925

Gaud 1947c, **AA**, Tansikht; Gaud 1953a, **EM**, Figuig, Berguent, Anoual, Boudnib, Aïn Chair, Aoufous, Tarhit, **HA**, Tazenakht, Tichka, Tizi-n’Telghemt, **AA**, Ouarzazate, Zagora; Bailly-Choumara 1965c, **EM**, Oued Isker, El Megrinat, Taskala, Aïn Aït Delouine, Talmesdourt, Aouinet Torkoz, Bouanama, Rich Tamlougout, Assa, **AA**, Tafraout ville, Oued Jemâa Idaousmaal, Aït abdallah, Issedrim Igmur Igues, **SA**, Vallée de l’Oued Assaka, Tacharicht, Bou Izakarn, Aïn Erkha; Chlaida and Bouzidi 1995, **AP**, Sud de Settat; Himmi et al. 1995; Chlaida 1997, **AP**, Barrage Al Massira; Dakki 1997; Trari et al. 2002; Himmi 2007; El Ouali Lalami et al. 2010a, **MA**, Fès; Trari 2017; Trari and Dakki 2017b; Trari et al. 2017

﻿*Culex (Maillotia) ﻿hortensis* Ficalbi, 1889

Charrier 1924, **Rif**, Tanger; Séguy 1930a; Langeron 1938, **HA**, Tounfite; Callot 1940, **HA**, Anefgou (2500 m), Tirghist (2500 m), Tighermine (2500 m); Gaud 1953a, **HA**, Tizi-n’Telghemt, Tizi-n’Tichka; Bailly-Choumara 1967a, **MA**, piste tafechna-Znan Imes, Khénifra, piste Tafechna-Senoual-Itzer, Ajdir, Itzer, Ghorm El Alem, El Ksiba, piste Ksiba-Naour, Piste Naour-Arbala, piste Arbala-El Kbab, Zouia Cheikh, Oued Sarif, Ifrane, Boulmane, Imilchil; Bailly-Choumara 1967b, **EM**, Tafraout, Grotte du Zegzel; Bailly-Choumara 1967c, **Rif**, Chaouen ville, route Chaouen-Bab Taza, Bab Taza, piste Bab Taza-Fifi, piste Bab Taza-Talassemtane, piste Bab Taza-Béni Ahmed, route Bab Taza-Bab Berred, piste Bab Berred-Tamorote, route Bab Berred-Ketama, Ketama, piste Ketama-Mt Tiguidin, route nationale Jebha, route Targuist-Béni Boufrah,Trari 1991, **AP**, Gharb; El Bermaki 1993, **AP**, Casablanca; Himmi et al. 1995; Louah 1995, **Rif**, Riffien, Marina Smir, M’diq, Oued Maleh, Lajour, Tahaddart, Schroda, Talembote, Bab Berred, Punta Cirres, Sidi Kankoch, Oued kbir, Oued Jebel Lehbib; Louah et al. 1995; Chlaida and Bouzidi 1995, **AP**, Barrage Al Massira; Chlaida 1997, **AP**, Barrage Al Massira; Dakki 1997; Ramdani 1997, **AP**, Skhirat, Tamaris; Handaq 1998, **HA**, Amizmiz, Sidi Bou Othmane, Had Mhara, Bengrir; Trari et al. 2002; Himmi 2007, **Rif**, Bab Berred, Bab Taza, **AP**, Skhirate; El Ouali Lalami et al. 2010b, **MA**, Oued Fès, Oued El Himmer, Camping Sidi Harazem; Larhbali et al. 2010, **MA**, Merchouch, Ghoualem, Ezzhiliga, Oulmès, Tarmilate, M’rirt, Bni Ounzar, Ganzra (Khémisset); El Ouali Lalami 2012, **MA**, Oued Fès; Oued El Himmer, route de Sidi Harazem, Camping Sidi Harazem; Hadji et al. 2013, **AP**, Sidi Yahia du Gharb, Kcebia, Sidi Hagouch, Dar Belamri, Lalla Itto, Soualem (Sidi Slimane); Trari 2017; Trari and Dakki 2017b; Trari et al. 2017; Mouatassem et al. 2019, **MA**, Fès

﻿*Culex (Neoculex) ﻿impudicus* Ficalbi, 1890

Charrier 1924, **Rif**, Tanger; Séguy 1930a; Bailly-Choumara 1966, **AA**, Taliouine; Bailly-Choumara 1967a, **MA**, route Azrou-Khénifra, piste Tafechna-Senoual-Itzer, Itzer, piste Naour-Arbala, Zaouia Cheikh, Pont Tarmilate; Bailly-Choumara 1967b, **EM**, route Itzer-Midelt, Cascade Oued Za, Grotte du Zegzel; Bailly-Choumara 1967c, **Rif**, Chaouen ville, route Chaouen-Bab Taza, piste Bab Taza-Fifi, piste Bab Taza-Talassemtane, piste Bab Taza-Béni Ahmed, route Bab Taza-Bab Berred, piste Bab Berred-Asifane, piste Bab Berred-Tamorote, Ketama, route nationale Jebha, Al Hoceima Club Med, Targuist et environs; Trari 1991, **AP**, Sidi Amira, El Menzeh, Oued Sebou, Sidi Yahia du Gharb, Moulay Bousselham, Larache, Bordure Oued Loukous; Dakki 1997; Himmi et al. 1998, **AP**, Sidi Boughaba, Dayat d’El Menzeh (north east of Kénitra), Puits de Sidi Amira (forest of Maâmora); Trari et al. 2002; Himmi 1991, **AP**, Sidi Boughaba, El Menzeh, Sidi Amira; Himmi et al. 1995; Louah 1995, **Rif**, Haidra, Marina Smir, Tahaddart, Tanger; Louah et al. 1995; Ramdani 1997, **AP**, Tamaris; Alaoui Slimani 2002, **AP**, Rabat; Himmi 2007, **Rif**, Bab Berred, Bab Taza, **AP**, forest Maâmora; El Ouali Lalami et al. 2010a, **MA**, Fès; El Joubari et al. 2014, **Rif**, Smir lagoon; Trari 2017; Trari and Dakki 2017b; Trari et al. 2017

﻿*Culex (Neoculex) martinii* Medschid, 1930

Bailly-Choumara 1968a, **Rif**, Al Hoceima, **AP**, Sidi Yahia du Gharb, Sidi Allal Tazi, Larache, Rabat, **HA**, Marrakech, **AA**, Tiznit; Himmi et al. 1995; Louah 1995, **Rif**, Haidra, Marina Smir, Cabo Negro, Oued Maleh; Louah et al. 1995; Dakki 1997; Trari et al. 2002; Himmi 2007; Trari 2017; Trari and Dakki 2017b; Trari et al. 2017

##### Culisetini

﻿***Culiseta* Felt, 1904**

﻿*Culiseta (Allotheobaldia) longiareolata* (Macquart, 1838)

d’Anfreville 1916, **AP**, Salé; Séguy 1930a; Gaud 1947c, **AA**, Tansikht; Gaud 1953a, **HA**, Marrakech; Bailly-Choumara 1965c, **EM**, El Aïoun du Draa, Aïn Aït Delouine, Talmesdourt, Aouinet Torkoz, Rich Tamlougout, **AA**, Aït Onmar, Ouled Teima, Tiznit ville, Bounaamane, Id Baha, Tafnidilt, Guelta Zerga, Aïn Kerma, Tafraout ville, Oued Jemâa Idaousmaal, Toudi, Aït Abdallah, Igherm, Issedrim Igmur Igues, **SA**, Goulimine ville, Poste militaire de Boujrif, Embouchure de l’Oued Assaka, Ouaroun, Labyar, Asrir, Tacharicht, Bou Izakarn ville, Jemâa N’Tirhirte, Aït Erkha, Tantan ville; Bailly-Choumara 1966, **AA**, Aït melloul, Tiznit; Bailly-Choumara 1967a, **MA**, Jnane Imasse, Khénifra, piste Tafechna-Senoual-Itzer, Itzer, Ghorm El Alem, El ksiba, piste Naour-Arbala, Ifrane, Pont Tarmilate, Imouzzer Marmoucha; Bailly-Choumara 1967b, **EM**, Tafraout, Guercif ville, Grotte du Zegzel, Berguent, Tendrara; Bailly-Choumara 1967c, **Rif**, Chaouen ville, route Chaouen-Bab Taza, Bab Taza, route Bab Taza-Bab Berred, route Ketama-Targuist, route nationale Jebha, Targuist et environs, Aïn Hamra; Bailly-Choumara 1968a, **AP**, Meja Bokka, **AA**, Tiznit; Himmi 1991, **AP**, Sidi Boughaba, El Menzeh; Trari 1991, **AP**, El Menzeh, Gharb; El Bermaki 1993, **AP**, Sidi Maârouf; Himmi et al. 1995; Louah 1995, **Rif**, Fnideq, Riffien, Marina Smir, M’diq, Cabo Negro, Lajour, Ouadras, Punta Cirres, Ksar Sghir, Tanger; Louah et al. 1995; Mestari 1997, **AP**, Mohammedia; Moussalim 1997, **AP**, Maâmora, Fouarate; Ramdani 1997, **AP**, Témara, Skhirat, Tamaris; Dakki 1997; Handaq 1998, **HA**, Marrakech, Kelaâ Sraghna, Bengrir Himmi et al. 1998, **AP**, Sidi Boughaba, Dayat d’El Menzeh (north east of Kénitra); Bouallam 2001, **HA**, Bordure Oued N’fis; Alaoui Slimani 2002, **AP**, Rabat; Trari et al. 2002; Aouinty et al. 2006, **AP**, Mohammedia; Himmi 2007, **Rif**, Bab Berred, **AP**, Skhirate, Maâmora, forest of Hilton; Koçak and Kemal 2013; El Joubari et al. 2014, **Rif**, Smir lagoon; Trari 2017; Trari and Dakki 2017b; Trari et al. 2017; Mouatassem et al. 2019, **MA**, Fès

﻿*Culiseta (Culicella) ﻿fumipennis* (Stephens, 1825)

Gaud 1947c, **AP**, Rabat, Sidi Allal Tazi, **MA**, Khémisset; Senevet and Andarelli 1959, **AP**, Rabat, Casablanca, Bouznika, **MA**, Fès; Bailly-Choumara 1967a, **MA**, Jnane Imasse; Bailly-Choumara 1967c, **Rif**, route Bab Taza-Bab Berred; Himmi et al. 1995; Dakki 1997; Trari et al. 2002; Trari 2017; Trari and Dakki 2017b; Trari et al. 2017

﻿*Culiseta (Culicella) ﻿litorea* (Shute, 1928)

Metge 1986, **AP**, Casablanca; Dakki 1997; Carles-Tolrá 2002; Trari et al. 2002; Trari 2017; Trari and Dakki 2017b; Trari et al. 2017

﻿*Culiseta (Culiseta) ﻿annulata* (Schrank, 1776)

d’Anfreville 1916, **AP**, Salé; Charrier 1924, **Rif**, Tanger; Séguy 1930a, **Rif**, Tanger; Viamonte and Ramirez 1946, **Rif**, Ben Karrich, Asilah, Ketama, Malaliene, Tétouan, **AP**, Larache; Gaud 1952, **AP**, Souk El Hadd (Gharb); Gaud 1953a, **Rif**, Tanger, Tétouan, **EM**, Saidia, Talsint, **AP**, Casablanca, Rabat, Kénitra, **MA**, Meknès, Fès, Ifrane, Taza, **HA**, Marrakech, Aït Bouguemez, Midelt; Gaud 1957b, **Rif**, Tétouan, Tanger, **AP**, Sidi Allal Tazi, Rabat, Casablanca, **MA**, Meknès, Ifrane; Bailly-Choumara 1967a, **MA**, Jnane Imasse, maison forestière Ouiouane, piste Tafechna-Senoual-Itzer, Ghorm El Alem, Zaouia Cheikh, Pont Tarmilate, Ifrane; Bailly-Choumara 1967b, **EM**, Tafraout, route Itzer-Midelt; Bailly-Choumara 1967c, **Rif**, piste Bab Taza-Talassemtane; Bailly-Choumara 1972a, **AP**, Merja Sheishat; Himmi et al. 1995; Louah 1995, **Rif**, Riffien, M’diq, Tanger; Louah et al. 1995; Chlaida 1997, **AP**, Barrage Al Massira; Dakki 1997; Mestari 1997, **AP**, Mohammedia; Moussalim 1997, **AP**, Rabat; Ramdani 1997, **AP**, meseta côtière (Casablanca-Rabat); Himmi et al. 1998, **AP**, Sidi Boughaba; Himmi 1991, **AP**, Sidi Boughaba, El Menzeh; Trari 1991, **AP**, Sidi Boughaba, El Menzeh; Trari et al. 2002; Himmi 2007, **AP**, Skhirate, Maâmora, forest of Hilton, Oulja; Trari 2017; Trari and Dakki 2017b; Trari et al. 2017

﻿*Culiseta (Culiseta) subochrea* (Edwards, 1921)

Gaud 1952, **AP**, Gharb; Gaud 1957b, **Rif**, Tanger, Chefchaouen, **EM**, Oujda, **AP**, Sidi Allal Tazi, Casablanca; Bailly-Choumara 1967a, **MA**, Imilchil; Bailly-Choumara 1967c, **Rif**, Ketama; Bailly-Choumara 1972a, **AP**, Merja Sheishat; Himmi 1991, **AP**, Sidi Boughaba; Trari 1991, **AP**, Sidi Boughaba; Louah 1995, **Rif**, Riffien, Tres piedras, M’diq, Cabo Negro, Oued Maleh, Tanger; Louah et al. 1995; Dakki 1997; Mestari 1997, **AP**, Mohammedia; Moussalim 1997, **AP**, Kénitra; Ramdani 1997, **AP**, Témara, Skhirat, Casablanca; Handaq 1998, **AP**, El Jadida, **HA**, Bengrir; Himmi et al. 1998, **AP**, Sidi Boughaba; Trari et al. 2002; Himmi 2007, **AP**, Skhirat, Dayet Aïn Chems, Maâmora, forest of Hilton; El Joubari et al. 2014, **Rif**, Smir lagoon; Trari 2017; Trari and Dakki 2017b; Trari et al. 2017

##### Mansoniini

﻿***Coquillettidia* Dyar, 1905**

﻿*Coquillettidia (Coquillettidia) buxtoni* (Edwards, 1923)

Bailly-Choumara 1965a, **AP**, Merja Bokka; Bailly-Choumara 1970, **AP**, Merja Bokka; Bailly-Choumara 1972a, **AP**, Merja Sheishat (Larache); Bailly-Choumara 1973b, **AP**, Merja Bokka, Larache; Himmi et al. 1995; Trari et al. 2002; Trari 2017; Trari and Dakki 2017b; Trari et al. 2017

﻿*Coquillettidia (Coquillettidia) richiardii* (Ficalbi, 1889)

Bailly-Choumara 1965a, **AP**, Merja Bokka; Bailly-Choumara 1967a, **MA**, Zaouia Cheikh; Bailly-Choumara 1970, **AP**, Merja Bokka; Bailly-Choumara 1972a, **AP**, Merja Sheishat (Larache); Bailly-Choumara 1973b, **AP**, Merja Bokka; Trari 1991, **AP**, Sidi Yahia du Gharb, Aïn Chouk, Merja Bargha; Himmi et al. 1995; Dakki 1997; Moussalim 1997, **AP**, Fouarate sur les bordures de la Merja; Trari et al. 2002; Trari 2017; Trari and Dakki 2017b; Trari et al. 2017

##### Orthopodomyiini

﻿***Orthopodomyia* Theobald, 1904**

﻿*Orthopodomyia ﻿﻿﻿pulcripalpis* (Rondani, 1872)

Bailly-Choumara 1965b, **AP**, Maâmora; Himmi et al. 1995; Dakki 1997; Trari et al. 2002; Trari 2017; Trari and Dakki 2017b; Trari et al. 2017

##### Uranotaeniini

﻿***Uranotaenia* Lynch Arribálzaga, 1891**

﻿*Uranotaenia (Pseudoficalbia) ﻿unguiculata* Edwards, 1913

Séguy 1930a; Gaud 1953a, **HA**, Marrakech; Senevet and Andarelli 1959a, **EM**, Oujda, Berguent, Guercif, **AP**, Sidi Allal Tazi, Bouznika, Casablanca, Béni Moussa, **MA**, Oulad Massine, Meknès, Moulay Yacoub, Béni Mellal, **AA**, Ksar Mzizel, Aït Melloul; Bailly-Choumara 1965c, **EM**, Aïn Aït Delouine, Talmesdourt, Rich Tamlougout; Bailly-Choumara 1966, **AA**, Aït Ouabelli; Bailly-Choumara 1967a, **MA**, Pont Tarmilate; Bailly-Choumara 1967b, **EM**, environs de Taourirt, Madagh, Merja Boubker; Bailly-Choumara 1967c, **Rif**, route Bab Taza-Bab Berred, piste Ketama-Jebel Tidighine; Bailly-Choumara 1968a, **AP**, Oued Loukous, Merja Bokka, Kénitra, Oued Bou-Regreg; Himmi 1991, **AP**, Sidi Boughaba; Trari 1991, **AP**, Oued Sebou; El Bermaki 1993, **AP**, El Oulfa (Casablanca); Himmi et al. 1995; Dakki 1997; Ramdani 1997, **AP**, Tamaris; Handaq 1998, **AP**, Sidi Bennour, **HA**, Zima Chemaîa (Bengrir); Trari et al. 2002; Himmi 2007, **AP**, Sidi Boughaba; Trari 2017; Trari and Dakki 2017b; Trari et al. 2017; Mouatassem et al. 2019, **MA**, Fès

﻿*Uranotaenia (Uranotaenia) ﻿balfouri* Theobald, 1904

Bailly-Choumara 1968a, **AP**, Merja Bokka, Oued Loukous, Sidi Yahia, Oued Sebou (Kénitra), Oued Bou-Regreg; Himmi et al. 1995; Dakki 1997; Moussalim 1997, **AP**, Sidi Allal Tazi, Fouarate, Sidi Boughaba; Trari et al. 2002; Himmi 2007, **AP**, Sidi Boughaba; El Ouali Lalami et al. 2010a, **MA**, Fès Boulmane; Trari 2017; Trari and Dakki 2017b; Trari et al. 2017; Mouatassem et al. 2019, **MA**, Fès

#### ﻿﻿DIXIDAE

K. Kettani, R. Wagner

Number of species: **12**. Expected: 15

Faunistic knowledge of the family in Morocco: moderate

﻿***Dixa* Meigen, 1818**

﻿*Dixa ﻿﻿﻿caudatula* Séguy, 1928

Séguy 1930a, **HA**, Arround, Skoutana (2400 m); Vaillant 1959; Dakki 1997; Mouna 1998

﻿*Dixa ﻿﻿﻿dilatata* Strobl, 1900

= ﻿*Dixa ﻿﻿﻿riparia* Vaillant, in Vaillant 1959: 180

Vaillant 1959, **HA**, Source de M’Goum (2500 m), Gorges d’Imi-N’Ifri (1050 m); Vaillant 1965; Dakki 1997

﻿*Dixa ﻿﻿﻿maculata* Meigen, 1818

Séguy 1930a; Dakki 1997; Mouna 1998

﻿*Dixa ﻿﻿﻿mera* Séguy, 1930

Séguy 1930a, **MA**, forest of Timelilt (1900 m); Vaillant 1959; Dakki 1997; Mouna 1998

﻿*Dixa ﻿﻿﻿nebulosa* Meigen, 1830

Séguy 1930a, **HA**; Dakki 1997; Mouna 1998

﻿*Dixa ﻿﻿﻿perexilis* Séguy, 1928

Séguy 1930a, **HA**, riverside of Oued Imminen (Tachdirt, 2400 m); Vaillant 1959; Dakki 1997; Mouna 1998

﻿*Dixa ﻿﻿﻿puberula* Loew, 1849

Vaillant 1959, **HA**, headwaters of Asif M’Goum (2500 m); Vaillant 1965; Dakki 1997; Mouna 1998

﻿*Dixa ﻿﻿﻿submaculata* Edwards, 1920

Séguy 1930a, **MA**, Sidi Yahia, Talzent (1800 m); Dakki 1997; Mouna 1998

﻿***Dixella* Dyar & Shannon, 1924**

﻿*Dixella ﻿﻿﻿aestivalis* (Meigen, 1818)

Séguy 1930a, **AP**, Merja Boughaba; Vaillant 1965; Ramdani 1981; Ramdani and Tourenq 1982; Mouna 1998

﻿*Dixella ﻿﻿﻿attica* (Pandazis, 1933)

= ﻿*Dixella ﻿﻿﻿numidica* (Sicart, 1955)

Ebejer et al. 2019, **Rif**, Cabo Negro (indoors: 10 m)

﻿*Dixella ﻿﻿﻿martinii* (Peus, 1934)

Ebejer et al. 2019, **Rif**, Moulay Abdelsalam (965 m)

﻿*Dixella ﻿﻿﻿serotina* (Meigen, 1818)

= ﻿*Dixa ﻿﻿﻿serotina* Wied, in Mouna 1998: 85

Séguy 1930a, **AP**, Casablanca (between Kénitra and Oued Beth); Dakki 1997; Mouna 1998

### ﻿Chironomoidea

#### ﻿﻿CERATOPOGONIDAE

K. Kettani, B. Mathieu

Number of species: **62**. Expected: 80

Faunistic knowledge of the family in Morocco: moderate

##### Ceratopogoninae

Culicoidini

﻿***Culicoides* Latreille, 1809**

﻿*Culicoides (Avaritia) ﻿imicola* Kieffer, 1913

Kremer et al. 1971, **MA**, Fès-Meknès, **SA**, Guelmim-Oued Noun; Kremer et al. 1975; Kremer et al. 1979; Chaker et al. 1979, **SA**, Guelmim-Oued Noun; Chaker et al. 1980, **MA**, Fès, Rhafsai, **AA**, Torkoz, Tarhjisht; Remm 1988a; Dakki 1997; Bouayoune et al. 1998, **Rif**, Tanger-Tétouan-Al Hoceima, **EM**, Oriental, **AP**, Rabat-Salé-Kénitra, Safi, **MA**, Fès-Meknès, Béni Mellal-Khénifra, **HA**, Marrakech, **AA**, Draa-Tafilalet, Souss-Massa, **SA**, Guelmim-Oued Noun; Cêtre-Sossah and Baldet 2004, **AP**, Rabat-Salé-Kénitra; Lhor et al. 2015, **Rif**, Sahel Chamali, **MA**, Sidi Hammadi, Benioukil, Aïn Leuh, Ait Siberne, Meknès, **AA**, Errachidia, Sidi Dahmane, **SA**, Foum El Oued; Bourquia et al. 2019, **AP**, Rabat

﻿*Culicoides (Avaritia) ﻿montanus* Shakirzjanova, 1962

Kremer et al. 1971, **AP**, Rabat-Salé-Kénitra, Safi, **HA**, Marrakech, **SA**, Guelmim-Oued Noun; Kremer et al. 1975; Kremer et al. 1979, **Rif**, Tanger-Tétouan-Al Hoceima, **AP**, Safi, **HA**, Marrakech; Chaker et al. 1980, **Rif**, Al Hoceima, **AP**, Oued Cherrat, **HA**, Souk Tnine de Oudaias (Haouz), Marrakech, **AA**, Torkoz; Remm 1988a; Dakki 1997; Bourquia et al. 2019, **AP**, Rabat

﻿*Culicoides (Avaritia) obsoletus* (Meigen, 1818)

Callot et al. 1968, **Rif**, Al Hoceima, **AP**, Merja Bokka, Sidi Yahia du Gharb, Sidi-Bettache (Zaeir), Rabat-Salé-Kénitra, **HA**, El Harcha (plateau central); Bailly-Choumara and Kremer 1970, **AP**, estuaire de Bou-Regreg; Kremer et al. 1971, **AP**, Rabat-Salé-Kénitra, Safi, **MA**, Béni Mellal-Khénifra, **HA**, Marrakech; Kremer et al. 1975; Kremer et al. 1979; Chaker et al. 1979, **Rif**, Tanger-Tétouan-Al Hoceima, **AP**, Rabat-Salé-Kénitra, Casablanca, Settat, Safi, **HA**, Marrakech; Chaker et al. 1980, **Rif**, Al Hoceima, **AP**, Zaers, Sidi Bettache, **HA**, El Harcha, Talet Inaouane (Haouz); Remm 1988a; Dakki 1997; Bouayoune et al. 1998, **Rif**, Tanger-Tétouan-Al Hoceima, **AP**, Rabat-Salé-Kénitra, Casablanca, Settat, Safi, **MA**, Fès-Meknès, Béni Mellal-Khénifra, **HA**, Marrakech, **AA**, Souss-Massa, **SA**, Guelmim-Oued Noun; Cêtre-Sossah and Baldet 2004; Lhor et al. 2015, **Rif**, Sahel Chamali, **MA**, Ait Siberne; Bourquia et al. 2019, **AP**, Rabat

﻿*Culicoides (Avaritia) ﻿scoticus* Downes & Kettle, 1952

Kremer et al. 1971, **AP**, Safi, **MA**, Béni Mellal-Khénifra, **HA**, El Harcha, Talet Inaouane (Haouz), Marrakech; Kremer et al. 1975; Kremer et al. 1979; Chaker et al. 1979, **AP**, Casablanca-Settat, Safi, **HA**, Marrakech; Remm 1988a; Dakki 1997; Bourquia et al. 2019, **AP**, Rabat

﻿*Culicoides (Beltranmyia) circumscriptus* Kieffer, 1918

Callot et al. 1968; Bailly-Choumara and Kremer 1970, **Rif**, Smir lagoon, Oued Negro, **EM**, Merja Boubker (Berkane), Gouttitir (NE Guercif), **AP**, Merja Sheishat (Larache), Aïn Muelha (near Oued Sidi Allal Tazi, estuaire Oued Bou-Regreg, Dayat Qoudiya (Sidi Yahia Gharb); Kremer et al. 1971, **AP**, Rabat-Salé-Kénitra, Casablanca, Settat, Safi, **MA**, Fès-Meknès, **HA**, Marrakech, **SA**, Guelmim-Oued Noun; Kremer et al. 1975; Kremer et al. 1979; Chaker et al. 1979, **Rif**, Tanger-Tétouan-Al Hoceima, **AP**, Rabat-Salé-Kénitra, Casablanca, Settat, Safi, **MA**, Fès-Meknès, **AA**, Souss Massa; Chaker et al. 1980, **Rif**, Al Hoceima, **AP**, Merja Qoudiya, Merja Bokka, Sidi Yahia du Gharb, **MA**, Aïn Karma (Saiss), Oulmès, **HA**, Setti Fatma, **AA**, Aït Melloul (Souss); Dakki 1997; Cêtre-Sossah and Baldet 2004, **AP**, Rabat-Salé-Kénitra; Lhor et al. 2015, **Rif**, Sahel Chamali, **MA**, Sidi Hammadi, Benioukil, Meknès, **AA**, Errachidia, **SA**, Foum El Oued; Bourquia et al. 2019, **AP**, Rabat

﻿*Culicoides (Culicoides) ﻿fagineus* Edwards, in Edwards et al. 1939

Kremer et al. 1971, **AP**, Rabat-Salé-Kénitra, Safi, **MA**, Béni Mellal-Khénifra, **HA**, Marrakech; Kremer et al. 1975; Chaker et al. 1979, **AP**, Rabat-Salé-Kénitra; Kremer et al. 1979; Chaker et al. 1980, **AP**, Rabat, Sidi Bettache, **MA**, Khemisset, **HA**, Marrakech; Remm 1988a; Dakki 1997; Bourquia et al. 2019, **AP**, Rabat

﻿*Culicoides (Culicoides) ﻿newsteadi* Austen, 1921

= ﻿*Culicoides (Culicoides) ﻿halophilus* Kieffer, in Callot et al. 1968: 886, Bailly-Choumara and Kremer 1970: 386, Dakki 1997: 60

Callot et al. 1968, **Rif**, Cabo Negro (Ferma), Tétouan, Talerhza, Tanger-Tétouan-Al Hoceima, **AP**, Larache, Merja Bokka (Gharb), Aïn Chok, **HA**, Talet-Inaouan (Haouz); Bailly-Choumara and Kremer 1970, **Rif**, Smir lagoon, Oued Negro, **AP**, Merja Sheishat (Larache), Aïn Muelha (near Oued Sidi Allal Tazi, estuaire Oued Bou-Regreg, Dayat Qoudiya (Sidi Yahia du Gharb), **EM**, Merja Boubker (Berkane), Ksabi (NE Midelt), **HA**, Souk Tnine des Oudaias (bordure Oued N’fis), **AA**, Aïn Sefra (south Foum Zquid); Kremer et al. 1971, **AP**, Rabat-Salé-Kénitra, Casablanca, Settat, **MA**, Fès-Meknès, Béni Mellal-Khénifra, **SA**, Guelmim-Oued Noun; Kremer et al. 1975; Chaker et al. 1979, **Rif**, Tanger-Tétouan-Al Hoceima, **AP**, Rabat-Salé-Kénitra, Safi, Casablanca, Settat, **MA**, Béni Mellal- Khénifra, **HA**, Marrakech; Kremer et al. 1979; Baylis et al. 1997, **Rif**, Tanger, **HA**, Marrakech; Remm 1988a; Dakki 1997; Cêtre-Sossah and Baldet 2004, **AP**, Rabat-Salé-Kénitra; Lhor et al. 2015, **MA**, Aïn Leuh, Ait Siberne, Meknès, **SA**, Foum El Oued; Bourquia et al. 2019, **AP**, Rabat

﻿*Culicoides (Culicoides) ﻿pulicaris* (Linnaeus, 1758)

Kremer et al. 1971, **AP**, Rabat-Salé-Kénitra, **MA**, Fès-Meknès, Béni Mellal-Khénifra, **SA**, Guelmim-Oued Noun; Kremer et al. 1975; Kremer et al. 1979; Chaker et al. 1979, **AP**, Rabat-Salé-Kénitra, Safi, **HA**, Marrakech, **SA**, Guelmim-Oued Noun; Chaker et al. 1980, **MA**, Lalla Outka, Khénifra, Oulmès, **HA**, Talet Inaouan (Haouz), **AA**, Aouinet-Torkoz, Tarhjicht; Remm 1988a; Dakki 1997; Bouayoune et al. 1998, **Rif**, Tanger-Tétouan-Al Hoceima, **EM**, Oriental, **AP**, Rabat-Salé-Kénitra, Casablanca, Settat, Safi, **MA**, Fès-Meknès, Béni Mellal-Khénifra, **HA**, Marrakech, **AA**, Drâa-Tafilalet, Souss-Massa; Cêtre-Sossah and Baldet 2004; Lhor et al. 2015, **Rif**, Sahel Chamali, **MA**, Aïn Leuh, Aït Siberne, Meknès; Bourquia et al. 2019

﻿*Culicoides (Culicoides) ﻿punctatus* (Meigen, 1804)

Callot et al. 1968, **Rif**, Tanger-Tétouan-Al Hoceima; Bailly-Choumara and Kremer 1970, **Rif**, Merja Smir, Oued Negro, **AP**, Merja Sheishat (Larache), estuaire Bou-Regreg, **EM**, Merja Boubker (Berkane); Kremer et al. 1971, **Rif**, Cabo Negro (Ferma), Tétouan, **EM**, Berkane, **AP**, Rabat-Salé-Kénitra, **MA**, Fès-Meknès, **AA**, Foum Zguid; Kremer et al. 1975; Kremer et al. 1979; Chaker et al. 1979, **AP**, Casablanca-Settat; Dakki 1997; Lhor et al. 2015, **Rif**, Sahel Chamali, **MA**, Sidi Hammadi, Benioukil, Aïn Leuh, Meknès; Bourquia et al. 2019

﻿*Culicoides (Culicoides) ﻿subfagineus* Delécolle & Ortega, 1998

Bourquia et al. 2019, **AP**, Rabat

﻿*Culicoides (Monoculicoides) ﻿parroti* Kieffer, 1922

Bailly-Choumara and Kremer 1970, **HA**, Dar Saâda (Haouz); Kremer et al. 1971, **AP**, Rabat-Salé-Kénitra; Kremer et al. 1975; Kremer et al. 1979, **AP**, Safi, **HA**, Marrakech; Chakeret al. 1980, **AP**, Rabat, **HA**, Marrakech, Souk Tnine des Oudaias (Haouz); Remm 1988a; Dakki 1997; Bourquia et al. 2019

﻿*Culicoides (Monoculicoides) ﻿puncticollis* (Becker, 1903)

Callot et al. 1968, **AP**, Merja Qoudiya, Sidi Yahia du Gharb, Romani (Zaers), Rabat-Salé-Kénitra, **MA**, Aïn Karma (Saiss), **HA**, Souk Tnine des Oudaias (Haouz); Bailly-Choumara and Kremer 1970 (reported as *C.riethi* and corrected by Kremer et al. 1971), **Rif**, Merja Smir, **AP**, Merja Sheishat (Larache), estuaire Bou-Regreg, Dayat Qoudiya (Sidi Yahia du Gharb), **HA**, Souk Tnine des Oudaias (bordure de l’Oued N’fis), Dar Saâda (Haouz), Talet Inouane (bordure marécageuse du lac du Barrage Lalla Taguergoust); Kremer et al. 1971, **AP**, Rabat-Salé-Kénitra, **MA**, Fès-Meknès; Kremer et al. 1975; Kremer et al. 1979, **AP**, Rabat-Salé-Kénitra, Safi, **MA**, Fès-Meknès, **HA**, Marrakech; Chaker et al. 1980, **AP**, Aïn Karma, Zaers, Roumani, **HA**, Souk-Tnine des Oudaias; Remm 1988a; Cêtre-Sossah and Baldet 2004, **AP**, Rabat-Salé-Kénitra; Bourquia et al. 2019, **AP**, Rabat

﻿*Culicoides (Oecacta) ﻿azerbajdzhanicus* Dhzafarov, 1962

Bailly-Choumara and Kremer 1970, **HA**, Souk Tnine des Oudaias (bordure Oued N’fis); Kremer et al. 1971, **AP**, Safi, **MA**, Fès-Meknès, Beni Mellal-Khénifra, **HA**, Marrakech, **SA**, Guelmim-Oued Noun; Kremer et al. 1975; Kremer et al. 1979, **AA**, Souss-Massa **SA**, Guelmim-Oued Noun; Chaker et al. 1980, **MA**, Kkénifra, Rhafsai, **HA**, Marrakech, **AA**, Torkoz, Tarhjisht; Dakki 1997; Bourquia et al. 2019

﻿*Culicoides (Oecacta) ﻿longipennis* Khalaf, 1957

Kremer et al. 1971, **EM**, Berkane **AP**, Safi, **MA**, Fès-Meknès, **HA**, Marrakech; Kremer et al. 1975; Remm 1988a; Dakki 1997; Bourquia et al. 2019, **AP**, Rabat

﻿*Culicoides (Oecacta) ﻿marcleti* Callot, Kremer & Basset, 1968

Kremer et al. 1971, **MA**, Rhafsai, Fès-Meknès, **SA**, Guelmim-Oued Noun; Kremer et al. 1975; Remm 1988a; Dakki 1997; Bourquia et al. 2019

﻿*Culicoides (Oecacta) ﻿pallidus* Khalaf, 1957

= ﻿*Culicoides ﻿﻿﻿stackelbergi* Dhzafarov, in Kremer et al. 1971: 662, Dakki 1997: 61

Kremer et al. 1971, **AA**, Torkoz, **SA**, Guelmim-Oued Noun; Kremer et al. 1971; Remm 1988a; Dakki 1997; Bourquia et al. 2019

﻿*Culicoides (Oecacta) ﻿ravus* De Meillon, 1936

= ﻿Culicoides (Synhelea) subravus Cornet and Château, in Kremer et al. 1971: 664, Chaker et al. 1980: 85, Dakki 1997: 61

Kremer et al. 1971, **AP**, Safi, **HA**, Marrakech, **SA**, Guelmim-Oued Noun; Kremer et al. 1975; Kremer et al. 1979; Chaker et al. 1979, **AA**, Souss-Massa, **SA**, Guelmim-Oued Noun; Chaker et al. 1980, **HA**, Marrakech, **AA**, Torkoz, Tarhjicht, Aït Ouaballi (Draa); Remm 1988a; Dakki 1997; Bourquia et al. 2019

﻿*Culicoides (Oecacta) ﻿sahariensis* Kieffer, 1923

= ﻿*Culicoides ﻿﻿﻿colluzzii* Callot, Kremer and Bailly-Choumara, in Bailly-Choumara and Kremer 1970: 386, Chaker et al. 1980: 83, Dakki 1997: 61

Bailly-Choumara and Kremer 1970, **AP**, Merja Sheishat (Larache), **EM**, Merja Boubker (Berkane), **HA**, Souk Tnine des Oudaias (bordure Oued N’fis); Callot et al. 1970, **AP**, Larache, **HA**, Marrakech; Kremer et al. 1971, **AP**, Rabat-Salé-Kénitra, **MA**, Fès-Meknès, **SA**, Guelmim-Oued Noun; Kremer et al. 1975; Kremer et al. 1979; Chaker et al. 1979, **AP**, Rabat-Salé-Kénitra, **SA**, Guelmim-Oued Noun; Chaker et al. 1980, **AP**, Merja Bokka (Gharb), Rabat (Zaers), **EM**, Berkane, **MA**, Fès, Khémisset, **AA**, Tarhjisht; Baylis et al. 1997; Dakki 1997; Bouayoune et al. 1998; Cêtre-Sossah and Baldet 2004; Bourquia et al. 2019, **AP**, Rabat

﻿*Culicoides (Oecacta) ﻿santonicus* Callot, Kremer, Rault & Bach, 1966

Bailly-Choumara and Kremer 1970, **AP**, Merja Sheishat (Larache); Kremer et al. 1971, **AP**, Rabat-Salé-Kénitra; Kremer et al. 1975; Kremer et al. 1979; Chaker et al. 1979, **AP**, Rabat-Salé-Kénitra; Bailly-Choumara et al. 1980, **AP**, Larache, Sidi Bettache, **MA**, Oulmès, **EM**, El-Harcha; Remm 1988a; Dakki 1997; Bourquia et al. 2019

﻿*Culicoides (Oecacta) ﻿semimaculatus* Clastrier, 1958

Kremer et al. 1975, **Rif**, Tanger-Tétouan-Al Hoceima, **AP**, Larache, Casablanca-Settat, **MA**, Plateau Central (Khatouate); Chaker et al. 1979; Remm 1988a; Dakki 1997; Szadziewski and Dominiak 2006; Bourquia et al. 2019

﻿*Culicoides (Oecacta) sergenti* Kieffer, 1921

= ﻿*Culicoides (Oecacta) ﻿mosulensis* Khalaf, in Chaker et al. 1980: 84, Dakki 1997: 60

Kremer et al. 1979, **EM**, Oriental, **SA**, Guelmim-Oued Noun; Chaker et al. 1980, **AA**, Tarhjicht, **SA**, Bou-Arfa; Dakki 1997; Bourquia et al. 2019

﻿*Culicoides (Oecacta) ﻿similis* Carter, Ingram & Macfie, 1920

Kremer et al. 1971, **AA**, Torkoz, **SA**, Guelmim-Oued Noun; Kremer et al. 1975; Remm 1988a; Dakki 1997; Bourquia et al. 2019

﻿*Culicoides (Oecacta) ﻿truncorum* Edwards, 1939

= ﻿*Culicoides (Oecacta) ﻿sylvarum* Callot and Kremer, in Kremer et al. 1971: 662, Remm 1988a: 65, Dakki 1997: 61

Kremer et al. 1971, **AP**, Rabat-Salé-Kénitra, **MA**, Béni Mellal- Khénifra; Kremer et al. 1975; Remm 1988a; Dakki 1997; Bourquia et al. 2019

﻿*Culicoides (Pontoculicoides) ﻿saevus* Kieffer, 1922

Callot et al. 1968, **AP**, Sidi Yahia du Gharb, Rabat-Salé-Kénitra, Safi, **MA**, Aïn Karma (Saiss), **HA**, Talet Inouane, Marrakech, Souk Tnine des Oudaias (Haouz), **AA**, Aït Melloul (Souss), Ksar er Souk (Tafilalt), Tarhjicht, **SA**, Bou-Arfa; Bailly-Choumara and Kremer 1970, **AP**, Merja Sheishat (Larache), **HA**, Souk Tnine des Oudaias (bordure de l’Oued N’fis); Kremer et al. 1971, **AP**, Rabat-Salé-Kénitra, **MA**, Fès-Meknès **SA**, Guelmim-Oued Noun; Kremer et al. 1975; Kremer et al. 1979; Chaker et al. 1979, **EM**, Oriental, **AP**, Safi, **MA**, Fès-Meknès, **HA**, Marrakech, **AA**, Drâa-Tafilalet, Souss-Massa, **SA**, Guelmim-Oued Noun; Bailly-Choumara and Kremer 1980; Dakki 1997; Cêtre-Sossah and Baldet 2004, **AP**, Rabat-Salé-Kénitra; Bourquia et al. 2019

﻿*Culicoides (Pontoculicoides) ﻿sejfadinei* Dzhafarov, 1958

Kremer et al. 1975; Kremer et al. 1979; Chaker et al. 1979, **EM**, Oriental, **AA**, Drâa-Tafilalet; Bourquia et al. 2019

﻿*Culicoides (Remmia) ﻿kingi* Austen, 1912

Bailly-Choumara and Kremer 1970, **AA**, Mrimima (Oued de Foum Zquid), Souss-Massa; Chaker et al. 1980, **MA**, Meknès, **AA**, Tarhjisht; Cornet and Brunhes 1994; Bourquia et al. 2019, **AP**, Rabat

﻿*Culicoides (Remmia) schultzei* (Enderlein, 1908)

Callot et al. 1968, **AP**, Safi, **HA**, Talet Inouane, Marrakech; Dakki 1997; Bourquia et al. 2019

﻿*Culicoides (Sensiculicoides) ﻿badooshensis* Khalaf, 1961

Bailly-Choumara and Kremer 1970, **AP**, Merja Sheishat (Larache), **HA**, Marrakech, Souk Tnine des Oudaias; Kremer et al. 1971, **AP**, Rabat-Salé-Kénitra, Safi, **MA**, Fès-Meknès; Kremer et al. 1975; Kremer et al. 1979, **AP**, Rabat-Salé-Kénitra, Safi, **HA**, Marrakech; Dakki 1997; Chaker et al. 1980, **AP**, Oued Cherrat, Bousselham, Sidi Yahia, **MA**, Fès, **HA**, Marrakech; Bourquia et al. 2019

﻿*Culicoides (Sensiculicoides) ﻿cataneii* Clastrier, 1957

Kremer et al. 1971, **AP**, Rabat-Salé-Kénitra; Kremer et al. 1975; Kremer et al. 1979; Chaker et al. 1979, **Rif**, Tanger-Tétouan-Al Hoceima, **AP**, Rabat-Salé-Kénitra; Chaker et al. 1980, **AP**, Oued Cherrat, Rabat; Remm 1988a; Dakki 1997; Bourquia et al. 2019, **AP**, Rabat

﻿*Culicoides (Sensiculicoides) ﻿clastrieri* Callot, Kremer & Deduit, 1962

Bourquia et al. 2019, **MA**, Fès-Meknès

﻿*Culicoides (Sensiculicoides) ﻿derisor* Callot & Kremer, 1965

Chaker et al. 1980, **AP**, Rabat; Kremer et al. 1971, **AP**, Rabat-Salé-Kénitra, **MA**, Fès-Meknès; Kremer et al. 1975; Chaker et al. 1979, **AP**, Rabat-Salé-Kénitra; Kremer et al. 1979; Remm 1988a; Dakki 1997; Bourquia et al. 2019

﻿*Culicoides (Sensiculicoides) ﻿duddingstoni* Kettle & Lawson, 1955

Bourquia et al. 2019, **MA**, Fès-Meknès

﻿*Culicoides (Sensiculicoides) ﻿dzhafarovi* Remm, 1967

Kremer et al. 1971, **AP**, Rabat-Salé-Kénitra; Kremer et al. 1975; Kremer et al. 1979; Chaker et al. 1979, **SA**, Guelmim-Oued Noun; Chaker et al. 1980, **AP**, Oued Cherrat, **AA**, Tarhjisht; Remm 1988a; Dakki 1997; Bourquia et al. 2019

﻿*Culicoides (Sensiculicoides) ﻿festivipennis* Kieffer, 1914

= ﻿*Culicoides (Oecacta) ﻿odibilis* Austen, in Bailly-Choumara and Kremer 1970: 387, Chaker et al. 1980: 84, Dakki 1997: 61

Callot et al. 1968, **Rif**, Tanger-Tétouan-Al Hoceima; Bailly-Choumara and Kremer 1970, **Rif**, Merja Smir, **HA**, Souk Tnine des Oudaias (bordure Oued N‘fis); Kremer et al. 1971, **AP**, Rabat-Salé-Kénitra; Kremer et al. 1975; Kremer et al. 1979; Chaker et al. 1979, **Rif**, Tanger-Tétouan-Al Hoceima, **AP**, Rabat-Salé-Kénitra, Safi, Casablanca-Settat, **HA**, Marrakech; Chaker et al. 1980, **Rif**, Tétouan, **EM**, El-Harcha, **AP**, Aïn Chok, Rabat, Larache, **MA**, Oulmès, **HA**, Marrakech, Talet-Inaouan (Haouz); Dakki 1997; Cêtre-Sossah and Baldet 2004, **AP**, Rabat-Salé-Kénitra; Bourquia et al. 2019, **AP**, Rabat

﻿*Culicoides (Sensiculicoides) ﻿heteroclitus* Kremer and Callot, in Callot & Kremer, 1965

Kremer et al. 1975, **AP**, Safi, **HA**, Marrakech, **AA**, Tafraout, Tiznit, Souss-Massa; Chaker et al. 1979; **HA**, Haouz; Remm 1988a; Dakki 1997; Bourquia et al. 2019

﻿*Culicoides (Sensiculicoides) ﻿jumineri* Callot & Kremer, 1969

Kremer et al. 1971, **AP**, Rabat-Salé-Kénitra, **MA**, Fès-Meknès, **SA**, Guelmim-Oued Noun; Kremer et al. 1975; Kremer et al. 1979; Chaker et al. 1979, **EM**, Oriental, **AP**, Rabat-Salé-Kénitra, Safi, **HA**, Marrakech, **AA**, Souss-Massa, **SA**, Guelmim-Oued Noun; Chaket et al. 1980, **AP**, Oued Cherrat, Merja Bokka, Rabat, **MA**, Fès, **HA**, Talet-Inaouan (Haouz), **AA**, Torkoz, Tarhjisht, Aït Oubelli, **SA**, Bou-Arfa; Remm 1988a; Dakki 1997; Cêtre-Sossah and Baldet 2004, **AP**, Rabat-Salé-Kénitra; Bourquia et al. 2019, **AP**, Rabat

﻿*Culicoides (Sensiculicoides) ﻿kibunensis* Tokunaga, 1937

= ﻿Culicoides (Oecata) cubitalis Edwards, in Kremer et al. 1975: 205, Dakki 1997: 60

Kremer et al. 1975, **AP**, Safi, **MA**, Ifrane, Imouzzer-du-Kander, Fès-Meknès, **HA**, Haouz, Marrakech; Chaker et al. 1979; Dakki 1997; Bourquia et al. 2019

﻿*Culicoides (Sensiculicoides) ﻿kurensis* Dzhafarov in Gutsevich, 1960

Remm 1988a; Cêtre-Sossah and Baldet 2004, **AP**, Rabat-Salé-Kénitra; Bourquia et al. 2019

﻿*Culicoides (Sensiculicoides) landauae* Kremer, Rebholtz-Hirtzel & Bailly-Choumara, 1975

Kremer et al. 1975, **MA**, Imouzzer-du-Kander, Sefrou, Fès-Meknès; Chaker et al. 1979; Hervy et al. 1994; Borkent and Wirth 1997; Remm 1988a; Dakki 1997; Chilasse and Dakki 2004, **MA**; Borkent 2012; Bourquia et al. 2019

﻿*Culicoides (Sensiculicoides) ﻿langeroni* Kieffer, 1921

Bailly-Choumara and Kremer 1970, **HA**, Tnine des Oudaias (bordure de Oued N‘fis); Kremer et al. 1971, **AP**, Rabat-Salé-Kénitra; Kremer et al. 1975; Kremer et al. 1979; Bailly-Choumara and Kremer 1980, **MA**, Khénifra, **AA**, Tarhjicht, Torkoz (Draa); Dakki 1997; Bourquia et al. 2019

﻿*Culicoides (Sensiculicoides) ﻿maritimus* Kieffer, 1924

Bailly-Choumara and Kremer 1970, **AP**, estuaire de Bou-Regreg; Kremer et al. 1971, **Rif**, Tanger-Tétouan-Al Hoceima, **AP**, Rabat-Salé-Kénitra; Kremer et al. 1975; Kremer et al. 1979; Bailly-Choumara and Kremer 1980, **Rif**, Tétouan, **AP**, Larache, Rabat, Sidi Bettache, **HA**, Talet-Inaouan (Haouz), Souk Tnine des Oudaias (Haouz); Remm 1988; Dakki 1997; Bourquia et al. 2019

﻿*Culicoides (Sensiculicoides) ﻿odiatus* Austen, 1921

= ﻿*Culicoides ﻿﻿﻿lailae* Khalaf, in Bailly-Choumara and Kremer 1970: 387, Dakki 1997: 60

= ﻿*Culicoides ﻿﻿﻿indistinctus* Khalaf, in Kremer et al. 1975: 206, Dakki 1997: 60

Bailly-Choumara and Kremer 1970, **HA**, Tnine des Oudaias (bordure de Oued N‘fis); Kremer et al. 1971, **AP**, Rabat-Salé-Kénitra, **MA**, Fès-Meknès, **AA**, Torkoz, **SA**, Guelmim-Oued Noun; Kremer et al. 1975, **AA**, Tafraout, Souss-Massa; Kremer et al. 1979; Chaker et al. 1979, **AP**, Rabat-Salé-Kénitra, Safi, **HA**, Marrakech, **SA**, Guelmim-Oued Noun; Chaker et al. 1980, **AP**, Kénitra, Merja Bokka (Gharb), **HA**, Souk Tnine des Oudaias (Haouz), **AA**, Tarhjicht; Remm 1988a; Dakki 1997; Baylis et al. 1997; Bouayoune et al. 1998; Cêtre-Sossah and Baldet 2004, **AP**, Rabat-Salé-Kénitra; Sarvašová et al. 2014; Bourquia et al. 2019

﻿*Culicoides (Sensiculicoides) ﻿paolae* Boorman, 1996

Bourquia et al. 2019, **AP**, Rabat

﻿*Culicoides (Sensiculicoides) ﻿pictipennis* (Staeger, 1839)

Bailly-Choumara and Kremer 1970, **AP**, estuaire de Bou-Regreg; Kremer et al. 1971; Remm 1988a; Bourquia et al. 2019, **AP**, Rabat

﻿*Culicoides (Sensiculicoides) ﻿pseudopallidus* Khalaf, 1961

Bailly-Choumara and Kremer 1970, **HA**, Souk Tnine des Oudaias (bordure Oued N’fis); Kremer et al. 1971, **AP**, Safi, **MA**, Rhafsai, Fès-Meknès, **HA**, Marrakech; Kremer et al. 1975; Remm 1988a; Dakki 1997; Bourquia et al. 2019

﻿*Culicoides (Sensiculicoides) ﻿shaklawensis* Khalaf, 1957

Kremer et al. 1975, **AP**, Safi, **MA**, Sefrou, Fès-Meknès, **HA**, Setti Fatma, Marrakech; Chaker et al. 1979; Remm 1988a; Dakki 1997; Bourquia et al. 2019

﻿*Culicoides (Sensiculicoides) ﻿simulator* Edwards, 1939

Kremer et al. 1975, **MA**, Ifrane, Fès-Meknès, **HA**, Setti Fatma; Chaker et al. 1979; Dakki 1997; Bourquia et al. 2019

﻿*Culicoides (Sensiculicoides) ﻿univittatus* Vimmer, 1932

= ﻿*Culicoides ﻿﻿﻿agathensis* Callot, Kremer and Rioux, in Bailly-Choumara and Kremer 1970: 386, Kremer et al. 1971: 663, Chaker et al. 1980: 82, Dakki 1997: 60

Bailly-Choumara and Kremer 1970, **AP**, estuaire Bou-Regreg; Kremer et al. 1971, **AP**, Rabat-Salé-Kénitra, **Rif**, Tanger-Tétouan-Al Hoceima; Kremer et al. 1975; Kremer et al. 1979; Chaker et al. 1979, **Rif**, Tanger-Tétouan-Al Hoceima, **AP**, Rabat-Salé-Kénitra, Safi, **MA**, Fès-Meknès, **HA**, Marrakech; Chaker et al. 1980, **Rif**, Tétouan, **AP**, Larache, Sidi Bettache **MA**, Oulmès; Dakki 1997; Bourquia et al. 2019

﻿*Culicoides (Sensiculicoides) ﻿vidourlensis* Callot, Kremer, Molet & Bach, 1968

Bailly-Choumara and Kremer 1970, **AP**, Merja Sheishat (Larache), estuaire de Oued Bou-Regreg, **HA**, Souk Tnine des Oudaias (bordure de Oued N’fis); Kremer et al. 1971; Remm 1988a; Bourquia et al. 2019

﻿*Culicoides (Silvaticulicoides) ﻿pallidicornis* Kieffer, 1919

Bailly-Choumara and Kremer 1970, **HA**, Souk Tnine des Oudaias (bordure Oued N’fis); Kremer et al. 1971, **AA**, Torkoz, **SA**, Guelmim-Oued Noun; Dakki 1997; Balenghien et al. 2014, **SA**, Guelmim-Oued Noun; Bourquia et al. 2019

﻿*Culicoides (Silvaticulicoides) ﻿picturatus* Kremer & Deduit, 1961

Bailly-Choumara and Kremer 1970, **AP**, Merja Sheishat (Larache); Kremer et al. 1971, **Rif**, Talerhza, **EM**, El-Harcha, **AP**, Bousselham, Rabat-Salé-Kénitra, **MA**, Oulmès; Kremer et al. 1975; Kremer et al. 1979; Chaker et al. 1979, **AP**, Casablanca-Settat, **MA**, Béni Mellal-Khénifra; Remm 1988a; Dakki 1997; Sarvašová et al. 2014; Bourquia et al. 2019

﻿*Culicoides (Silvaticulicoides) ﻿subfasciipennis* Kieffer, 1919

Kremer et al. 1971, **AP**, Rabat-Salé-Kénitra, **MA**, Fès-Meknès, Béni Mellal-Khénifra; Kremer et al. 1975; Kremer et al. 1979; Chaker et al. 1979, **Rif**, Tanger-Tétouan-Al Hoceima; Bailly-Choumara and Kremer 1980, **AP**, Larache, Zaers, Rabat, Aïn Chok, **MA**, Sefrou; Remm 1988a; Hervy et al. 1994; Dakki 1997; Cêtre-Sossah and Baldet 2004, **AP**, Rabat-Salé-Kénitra; Bourquia et al. 2019

﻿*Culicoides (Wirthomyia) faghihi* Navai, 1971

Kremer et al. 1975, **AA**, Tafraout, Souss-Massa; Chaker et al. 1979; Remm 1988a; Hervy et al. 1994; Dakki 1997; Bourquia et al. 2019

﻿*Culicoides (Wirthomyia) ﻿minutissimus* (Zetterstedt, 1855)

Referred as *C.pumilus*

﻿*Culicoides (Wirthomyia) ﻿pumilus* (Winnertz, 1852)

Kremer et al. 1975, **AP**, Safi, **MA**, Ifrane, Imouzzer-du-Kander, Fès-Meknès, **HA**, Setti Fatma, Marrakech; Chaker et al. 1979; Dakki 1997; Bourquia et al. 2019

﻿*Culicoidescalloti* Kremer, Delécolle, Bailly-Choumara & Chaker, 1979

Chaker et al. 1979, **AA**, Souss Massa, **SA**, Guelmim-Oued Noun; Kremer et al. 1979, **AA**, Tarhjigt, Aït Ouaballi, Souss Massa, **SA**, Guelmim-Oued Noun; Chaker et al. 1980; Remm 1988a; Hervy et al. 1994; Borkent and Wirth 1997; Baylis et al. 1997; Dakki 1997; Koçak and Kemal 2010; Borkent 2012; Bourquia et al. 2019

##### Ceratopogonini

﻿***Alluaudomyia* Kieffer, 1913**

﻿*Alluaudomyia ﻿﻿﻿hygropetrica* Vaillant, 1954

Vaillant 1956b, **HA**, Sidi Chamarouch

##### Palpomyiini

﻿***Bezzia* Kieffer, 1899**

﻿*Bezzia (Bezzia) ﻿nigritula* (Zetterstedt, 1838)

= ﻿*Palpomyia ﻿﻿﻿tenebricosa* Goetghebuer, 1912, in Vaillant 1956b: 241

Vaillant 1956b, **HA**, Tamesrit

﻿***Palpomyia* Meigen, 1818**

﻿*Palpomyia ﻿﻿﻿helviscutellata* Borkent, in Borkent & Wirth 1997

= ﻿*Dasyhelea ﻿﻿﻿flavoscutellata* (Zetterstedt, 1850), in Vaillant 1956b: 244

Vaillant 1956b, **HA**, Tahanaout

##### Forcipomyiinae

Dasyheleini

﻿***Dasyhelea* Kieffer, 1911**

﻿*Dasyhelea (Prokempia) ﻿flaviventris* (Goetghebuer, 1910)

Dominiak 2012

﻿*Dasyhelea (Pseudoculicoides) ﻿turficola* Kieffer, 1925

Dominiak 2012

##### Leptoconopinae

﻿***Leptoconops* Skuse, 1889**

﻿*Leptoconops (Holoconops) ﻿laurae* (Weiss, 1912)

Remm 1988b

#### ﻿﻿CHIRONOMIDAE

K. Kettani

Number of species: **412**. Expected: 600

Faunistic knowledge of the family in Morocco: good

##### Buchonomyiinae

﻿***Buchonomyia* Fittkau, 1955**

﻿*Buchonomyia ﻿thienemanni* Fittkau, 1955

Ashe and O’Connor 2009; Kettani et al. 2010, **Rif**, Oued Kelaâ (Akoumi, 400 m); Kettani and Langton 2012; Ashe et al. 2015, **Rif**, Oued Kelaâ (Akoumi, 400 m); Kettani and Moubayed-Breil 2018, **Rif**

##### Podonominae

﻿***Paraboreochlus* Thienemann, 1939**

﻿*Paraboreochlus ﻿﻿﻿minutissimus* (Strobl, 1895)

Azzouzi et al. 1992, **HA**, Oued Tensift; Kettani et al. 2001; Ashe and O’Connor 2009; Kettani and Langton 2012; Moubayed-Breil and Kettani 2019, **Rif**, Chrafate, Challal Sghir (Akchour)

##### Tanypodinae

Macropelopiini

﻿***Apsectrotanypus* Fittkau, 1962**

﻿*Apsectrotanypus ﻿﻿﻿trifascipennis* (Zetterstedt, 1838)

Kettani et al. 2010, **Rif**, Aïn Abou Hayane (Tiouertiouane, 880 m), Oued Maggou (Maggou village, 777 m), Oued Kanar (Gorges Kanar, 280 m); Kettani and Langton 2012

﻿***Macropelopia* Thienemann, 1916**

﻿*Macropelopia ﻿﻿﻿adaucta* Kieffer, 1916

Kettani and Langton 2011, **Rif**, Fifi, Issaguen; Kettani and Langton 2012

﻿*Macropelopia ﻿﻿﻿nebulosa* (Meigen, 1804)

Azzouzi et al. 1992, **HA**, Oued Tensift; Kettani et al. 1994, **Rif**, Oued Siflaou; Kettani et al. 1996; Dakki 1997; Kettani et al. 1997, **Rif**, Oued Khizana (Oued Laou); Kettani et al. 2001; Kettani and El Ouazzani 2005, **Rif**, amont Oued Nakhla; Ashe and O’Connor 2009; Kettani and Langton 2012; Kettani and Moubayed-Breil 2018, **Rif**

﻿***Psectrotanypus* Kieffer, 1909**

﻿*Psectrotanypus ﻿﻿﻿varius* (Fabricius, 1787)

Kettani et al. 1996; Kettani et al. 1997, **Rif**, Ras el Ma (Chefchaouen); Kettani et al. 2001; Ashe and O’Connor 2009; Kettani et al. 2010, **Rif**, Oued Tassikeste (Afechtal, 240 m); Kettani and Langton 2012

##### Pentaneurini

﻿***Ablabesmyia* Johannsen, 1905**

﻿*Ablabesmyia (Ablabesmyia) ﻿ebbae* Lehmann, 1981

Lehmann 1981; Azzouzi and Laville 1987; Kettani et al. 2001; Ashe and O’Connor 2009; Kettani and Langton 2012

﻿*Ablabesmyia (Ablabesmyia) ﻿longistyla* Fittkau, 1962

El Mezdi and Giudicelli 1985, **HA**, Khettaras de Marrakech; Azzouzi et al. 1992, **HA**, Oued Tensift; Kettani et al. 1994, **Rif**, Haut Laou, Oued Siflaou, aval Barrage Talembote, aval Oued Laou; Kettani et al. 1995, **Rif**, aval Oued El Kbir, Oued El Kbir, amont Oued Nakhla, Oued Mhajrat; Kettani et al. 1996; Dakki 1997; Kettani et al. 1997, **Rif**, Maggou (Oued Laou), Oued Khizana (Oued Laou); Kettani et al. 2001; Ashe and O’Connor 2009; Kettani et al. 2010, **Rif**, source Maggou (Maggou, 1300 m), Oued Talembote; Kettani and Langton 2012

﻿*Ablabesmyia (Ablabesmyia) ﻿monilis* (Linnaeus, 1758)

Reiss 1977, **Rif**, Tétouan, **HA**, kranichsee (Dra-Tal); Azzouzi and Laville 1987, **Rif**, retenue El Makhazine; El Mezdi and Giudicelli 1985, **HA**, Khettaras de Marrakech; Naya 1988, **MA**, Haut Sebou; Kettani et al. 2001; Ashe and O’Connor 2009; Kettani and Langton 2012

﻿***Conchapelopia* Fittkau, 1957**

﻿*Conchapelopia (Conchapelopia) ﻿melanops* (Meigen, 1818)

Kettani et al. 1996; Kettani et al. 1997, **Rif**, Ras el Ma (Chefchaouen); Kettani et al. 2001; Ashe and O’Connor 2009; Kettani and Langton 2012

﻿*Conchapelopia (Conchapelopia) ﻿pallidula* (Meigen, 1818)

Kettani and Moubayed-Breil 2018, **Rif**

﻿*Conchapelopia (Conchapelopia) ﻿viator* (Kieffer, 1911)

= ﻿*Conchapelopia* Pe 1 Langton 1991 in Kettani et al. 1994: 28, Kettani et al. 1995: 256

Azzouzi et al. 1992, **HA**, Gorges de Dadès (Imdiazen, 1900 m); Kettani et al. 1994; Kettani et al. 1996; Dakki 1997; Kettani et al. 1997, **Rif**, Oued Khizana (Oued Laou); Kettani et al. 2001; Kettani and El Ouazzani 2005, **Rif**, amont Oued Nakhla; Ashe and O’Connor 2009; Kettani and Langton 2012

﻿***Larsia* Fittkau, 1962**

﻿*Larsia ﻿﻿﻿atrocincta* (Goetghebuer, 1942)

Azzouzi et al. 1992, **HA**, Gorges de Dadès (Imdiazen, 1900 m); Kettani et al. 1994, **Rif**, Oued Moulay Bouchta; Kettani et al. 1996; Dakki 1997; Kettani et al. 1997, **Rif**, Oued Khizana (Oued Laou); Kettani et al. 2001; Ashe and O’Connor 2009; Kettani and Langton 2012; Kettani and Moubayed-Breil 2018, **Rif**

﻿*Larsia ﻿﻿﻿curticalcar* (Kieffer, 1918)

Azzouzi et al. 1992, **HA**, Oued Tensift; Kettani et al. 2001; Ashe and O’Connor 2009; Kettani et al. 2010, **Rif**, Nord Maggou village (Maggou, 905 m); Kettani and Langton 2012

﻿***Nilotanypus* Kieffer, 1923**

﻿*Nilotanypus ﻿﻿﻿dubius* (Meigen, 1804)

Azzouzi et al. 1992, **HA**, Oued Tensift; Kettani et al. 1994, **Rif**, Oued Siflaou, Oued Moulay Bouchta, aval Oued Tassikeste; Kettani et al. 1995, **Rif**, aval Oued El Kbir, aval Oued Krikra, Oued El Kbir, amont Oued Nakhla, Oued Mhajrat, aval Oued Khemis; Kettani et al. 1996; Dakki 1997; Kettani et al. 1997, **Rif**, Oued Khizana (Oued Laou); Kettani et al. 2001; Kettani et al. 2010, **Rif**, Oued Kanar (Gorges Kanar, 280 m), Oued Tassikeste (Afechtal, 240 m), Oued Talembote (Usine électrique, 120 m), Oued Laou (Afertane, 55 m); Ashe and O’Connor 2009; Kettani and Langton 2012; Kettani and Moubayed-Breil 2018, **Rif**

﻿***Paramerina* Fittkau, 1962**

﻿*Paramerina ﻿﻿﻿cingulata* (Walker, 1856)

Azzouzi et al. 1992, **HA**, Oued Tensift; Kettani et al. 1994, **Rif**, Haut Laou, Oued Siflaou, Oued Moulay Bouchta, aval Barrage Talembote; Kettani et al. 1995, **Rif**, aval Oued El Kbir, aval Oued Krikra, Oued El Kbir, amont Oued Nakhla, Oued Mhajrat, aval Oued Khemis; Kettani et al. 1996; Dakki 1997; Kettani et al. 1997, **Rif**, Ras el Ma (Chefchaouen); Kettani et al. 2001; Kettani and El Ouazzani 2005, **Rif**, amont Oued Nakhla; Ashe and O’Connor 2009; Kettani et al. 2010, **Rif**, Oued Talembote (aval Barrage Talembote, 245 m); Kettani and Langton 2012; Kettani and Moubayed-Breil 2018, **Rif**

﻿*Paramerina ﻿﻿﻿divisa* (Walker, 1856)

Kettani et al. 1996; Kettani et al. 1997, **Rif**, Oued Khizana (Oued Laou); Kettani et al. 2001; Kettani and Langton 2012

﻿*Paramerina ﻿﻿﻿mauretanica* Fittkau, 1962

Fittkau 1962, **Atlas** (850 m), **SA**; Azzouzi and Laville 1987; Ashe and Cranston 1990, **EM**, Figuig; Kettani et al. 2001; Ashe and O’Connor 2009, **EM**, Figuig; Kettani et al. 2010, **Rif**, Oued Tassikeste (Afechtal, 240 m); Kettani and Langton 2012

﻿*Paramerina* ﻿spec. Greichenland (Fittkau, 1962)

Kettani et al. 1994, **Rif**, Haut Laou, Oued Siflaou, Oued Moulay Bouchta, aval Barrage Talembote; Kettani et al. 1995, **Rif**, aval Oued El Kbir, aval Oued Krikra, Oued El Kbir, amont Oued Nakhla, Oued Mhajrat; Kettani et al. 1996; Dakki 1997; Kettani et al. 2001; Laville and Langton 2002; Kettani et al. 2010, **Rif**, Oued Chrafat (Armotah, 900 m), Oued Talembote (aval Barrage Talembote, 245 m); Kettani and Moubayed-Breil 2018, **Rif**

﻿***Pentaneurella* Fittkau & Murray, 1983**

﻿*Pentaneurella* ﻿﻿sp. Ourika

Azzouzi et al. 1992, **HA**; Kettani et al. 2001

﻿***Rheopelopia* Fittkau, 1962**

﻿*Rheopelopiamaculipennis* (Zetterstedt, 1838)

Naya 1988, **MA**, Haut et Moyen Sebou; Azzouzi and Laville 1987, **MA**, Oum-er-Rbia, **HA**, Tensift; Kettani et al. 1994, **Rif**, Oued Siflaou, Oued Moulay Bouchta; Kettani et al. 1995, **Rif**, aval Oued El Kbir, Oued Mhajrat, aval Oued Khemis; Kettani et al. 1996; Dakki 1997; Kettani et al. 1997, **Rif**, Maggou (Oued Laou), Ras el Ma (Chefchaouen); Kettani et al. 2001; Kettani and El Ouazzani 2005, **Rif**, amont Oued Nakhla; Dakki et al. 2008, **MA**, Oued Sebou; Ashe and O’Connor 2009; Kettani et al. 2010, **Rif**, Ruisselet maison forestière (Talassemtane, 1683 m), Source Maggou (Maggou, 1300 m), Oued Talembote (avant village Talembote, 320 m), Oued Tassikeste (Afechtal, 240 m); Kettani and Langton 2012; Kettani and Moubayed-Breil 2018, **Rif**

﻿*Rheopelopia ﻿﻿﻿murrayi* Dowling, 1983

Dowling 1983, **AA**, Tata (Moyen Draa); Ashe and Cranston 1990; Kettani et al. 2001; Ashe and O’Connor 2009; 2012; Kettani and Langton 2012; Kettani and Moubayed-Breil 2018, **Rif**

﻿*Rheopelopia ﻿﻿﻿ornata* (Meigen, 1838)

Azzouzi and Laville 1987, **MA**, Oued Fès; Kettani et al. 1995, **Rif**, Oued El Kbir, amont Oued Nakhla, aval Oued Khemis; Kettani et al. 1996; Kettani et al. 1997, **Rif**, Oued Khizana (Oued Laou), Ras el Ma (Chefchaouen); Kettani et al. 2001; Kettani and El Ouazzani 2005, **Rif**, amont Oued Nakhla; Dakki et al. 2008, **MA**, Oued Sebou; Ashe and O’Connor 2009; Kettani et al. 2010, **Rif**, Oued Talembote (avant village Talembote, 320 m); Kettani and Langton 2012; Kettani and Moubayed-Breil 2018, **Rif**

﻿***Telopelopia* Roback, 1971**

﻿*Telopelopia ﻿﻿﻿fascigera* (Verneaux, 1970)

= ﻿*Telopelopia ﻿maroccana* Murray, 1980, in Reiss 1977: 91, Murray 1980: 151, Azzouzi and Laville 1987: 218, Ashe and Cranston 1990: 133

Reiss 1977, **AP**, Larache, **HA**, Dra-Tal; Murray 1980, **AP**, Larache, **HA**, Dra-Tal; Azzouzi and Laville 1987; Ashe and Cranston 1990; Azzouzi and Laville 1987, **MA**, Oum-er-Rbia; Kettani et al. 2001; Ashe and O’Connor 2009; Kettani and Langton 2012

﻿***Telmatopelopia* Fittkau, 1962**

﻿*Telmatopelopia ﻿﻿﻿nemorum* (Goetghebuer, 1921)

Kettani et al. 1996, **Rif**, Oued Khizana (Oued Laou); Kettani et al. 1997, **Rif**, Oued Khizana (Oued Laou); Kettani et al. 2001; Ashe and O’Connor 2009; Kettani and Langton 2012

﻿***Thienemannimyia* Fittkau, 1957**

﻿*Thienemannimyia (Thienemannimyia) ﻿berkanea* Dowling, 1987

Dowling 1987, **EM**, Berkane; Azzouzi et al. 1992, **EM**, Environs de Berkane, **HA**, Ouarzazate (1160 m), Oasis Meski (1160 m), Aït Saoun; Kettani et al. 2001; Ashe and O’Connor 2009; Kettani and Langton 2012; Kettani and Moubayed-Breil 2018, **Rif**

﻿*Thienemannimyia (Thienemannimyia) ﻿carnea* (Fabricius, 1805)

Kettani and Langton 2012, **Rif**

﻿*Thienemannimyia (Thienemannimyia) ﻿choumara* Dowling, 1983

Dowling 1983, **EM**, Environ de Berkane (Monts de Bni Snassen), **HA**, Souk des Judais (Marrakech); Azzouzi et al. 1987, **HA**, Dra-Tal; Ashe and Cranston 1990; Kettani et al. 2001; Ashe and O’Connor 2009; Kettani and Langton 2012

﻿*Thienemannimyia (Thienemannimyia) ﻿geijskesi* (Goetghebuer, 1934)

Kettani and Langton 2012, **Rif**, Oued Zarka

﻿*Thienemannimyia (Thienemannimyia) ﻿laeta* (Meigen, 1818)

Azzouzi et al. 1992, **HA**, Oued Tensift; Kettani et al. 2001; Ashe and O’Connor 2009; Kettani and Langton 2012; Kettani and Moubayed-Breil 2018, **Rif**

﻿*Thienemannimyia (Thienemannimyia) ﻿lentiginosa* (Fries, 1823)

Azzouzi et al. 1992, **HA**, Oued Tensift; Kettani et al. 2001; Ashe and O’Connor 2009; Kettani and Langton 2012; Kettani and Moubayed-Breil 2018, **Rif**

﻿*Thienemannimyia (Thienemannimyia) ﻿northumbrica* (Edwards, 1929)

Fittkau 1962; Azzouzi and Laville 1987, **MA**, Oum-er-Rbia; Kettani et al. 1994, **Rif**, Haut Laou, Oued Siflaou, Oued Moulay Bouchta; Kettani et al. 1995, **Rif**, aval Oued El Kbir, aval Oued Krikra, Oued El Kbir, amont Oued Nakhla, aval Oued Khemis; Kettani et al. 1996; Dakki 1997; Kettani et al. 1997, **Rif**, Oued Khizana (Oued Laou); Kettani et al. 2001; Dakki et al. 2008, **MA**, Oued Sebou; Ashe and O’Connor 2009; Kettani and Langton 2012

﻿***Trissopelopia* Kieffer, 1923**

﻿*Trissopelopia ﻿﻿﻿longimana* (Staeger, 1839)

Azzouzi et al. 1992, **HA**, Oued Tensift; Kettani et al. 2001; Ashe and O’Connor 2009; Kettani and Langton 2012; Kettani and Moubayed-Breil 2018, **Rif**

﻿***Xenopelopia* Fittkau, 1962**

﻿*Xenopelopia ﻿﻿﻿falcigera* (Kieffer, 1911)

Kettani and Langton 2011, **Rif**, Anasser, Fifi, **AP**, marais de Loukous; Kettani and Langton 2012

﻿*Xenopelopia ﻿﻿﻿nigricans* (Goetghebuer, 1927)

Kettani et al. 1994, **Rif**, aval Oued Talembote (usine éléctrique); Kettani et al. 1996; Dakki 1997; Kettani et al. 2001; Ashe and O’Connor 2009; Kettani et al. 2010, **Rif**, Oued Talembote (aval affluent Talembote, 155 m); Kettani and Langton 2012; Kettani and Moubayed-Breil 2018, **Rif**

﻿***Zavrelimyia* Fittkau, 1962**

﻿*Zavrelimyia (Zavrelimyia) ﻿barbatipes* (Kieffer, 1911)

Naya 1988, **MA**, Moyen Sebou; Kettani et al. 2001; Ashe and O’Connor 2009 (?); Kettani et al. 2010, **Rif**, Oued Tiffert (Tiffert Talassemtane, 1230 m), Aïn Abou Hayane (Tiouertiouane, 880 m), Oued Abiyati (Ifansa, 140 m); Kettani and Langton 2012; Kettani and Moubayed-Breil 2018, **Rif**

﻿*Zavrelimyia (Zavrelimyia) ﻿berberi* Fittkau, 1962

Azzouzi and Laville 1987; Ashe and Cranston 1990, **HA**, Tamhda; Kettani et al. 2001; Ashe and O’Connor 2009; Kettani and Langton 2012; Kettani and Moubayed-Breil 2018, **Rif**

﻿*Zavrelimyia (Zavrelimyia) ﻿hirtimana* (Kieffer, 1918)

Kettani and Langton 2012

﻿*Zavrelimyia (Zavrelimyia) ﻿melanura* (Meigen, 1804)

Azzouzi et al. 1992, **HA**, Oued Tensift; Kettani et al. 2001; Ashe and O’Connor 2009; Kettani and Langton 2012; Kettani and Moubayed-Breil 2018, **Rif**

﻿*Zavrelimyia (Zavrelimyia) ﻿nubila* (Meigen, 1830)

Kettani and Langton 2011, **Rif**, marais de Lemtahane (PNPB), Dayat Aïn Rami, Dayat Amlay; Kettani and Langton 2012

##### Procladiini

﻿***Procladius* Skuse, 1889**

﻿*Procladius (Holotanypus) ﻿brevipetiolatus* (Goetghebuer, 1935)

Azzouzi et al. 1992, **HA**, Oued Meski (1160 m), Khettaras de Marrakech; Kettani et al. 2001; Kettani and Langton 2012

﻿*Procladius (Holotanypus) ﻿choreus* (Meigen, 1804)

Ramdani and Tourenq 1982, **AP**, Merja Sidi Boughaba; El Mezdi and Giudicelli 1985, **HA**, Khettaras de Marrakech; Azzouzi and Laville 1987, **AP**, Merja Sidi Boughaba; Kettani et al. 1994, **Rif**, Haut Laou, Oued Siflaou, Oued Moulay Bouchta, aval Barrage Talembote; Kettani et al. 1995, **Rif**, amont Oued Nakhla, aval Oued Khemis; Kettani et al. 1996; Dakki 1997; Kettani et al. 2010, **Rif**, Aïn Talassemtane (Talassemtane, 1700 m), Oued Talembote (aval Barrage Talembote, 245 m), Oued Tassikeste (Afechtal, 240 m); Kettani and Langton 2012; Kettani and Moubayed-Breil 2018, **Rif**

﻿*Procladius (Holotanypus) ﻿culiciformis* (Linnaeus, 1767)

Kettani and Moubayed-Breil 2018, **Rif**

﻿*Procladius (Holotanypus) ﻿noctivagus* (Kieffer, 1910)

Azzouzi et al. 1992, **HA**, Ouarzazate (1160 m); Kettani et al. 2001; Kettani and Langton 2012

﻿*Procladius (Holotanypus) ﻿sagittalis* (Kieffer, 1909)

Kettani et al. 1996; Kettani et al. 1997, **Rif**, Oued Khizana (Oued Laou); Kettani et al. 2001; Kettani and Langton 2012; Kettani and Moubayed-Breil 2018, **Rif**

﻿*Procladius (Psilotanypus) ﻿anomalus* Kieffer, 1906

*Nomen ﻿﻿dubium* in Ashe and O’Connor 2009: 213

Naya 1988, **MA**; Kettani et al. 2001; Kettani and Langton 2012

﻿*Procladius* Pe 3 Langton 1991

Kettani et al. 1994, Kettani et al. 1995, **Rif**, Oued Mhajrat; Kettani et al. 1996; Kettani et al. 2001; Dakki 1997

##### Tanypodini

﻿***Tanypus* Meigen, 1803**

﻿*Tanypus (Tanypus) ﻿brevipalpis* (Kieffer, 1923)

Reiss 1977, **EM**, Berkane; Ashe and O’Connor 2009 (?); Kettani and Langton 2012

﻿*Tanypus (Tanypus) ﻿kraatzi* (Kieffer, 1912)

Azzouzi et al. 1992, **HA**, Oasis Meski; Kettani et al. 2001; Ashe and O’Connor 2009; Kettani and Langton 2012

﻿*Tanypus (Tanypus) ﻿punctipennis* Meigen, 1818

Reiss 1977, **EM**, Berkane; El Mezdi and Giudicelli 1985, **HA**, Khettaras de Marrakech; Azzouzi and Laville 1987, **HA**, Oued Tensift; Kettani et al. 1996; Kettani et al. 1997, **Rif**, Oued Khizana (Oued Laou); Kettani et al. 2001; Ashe and O’Connor 2009; Kettani and Langton 2012; Kettani and Moubayed-Breil 2018, **Rif**

##### Diamesinae

Boreoheptagyiini

﻿***Boreoheptagyia* Brundin, 1966**

﻿*Boreoheptagyia ﻿﻿﻿legeri* (Goetghebuer, 1933)

= ﻿*Boreoheptagyia ﻿﻿﻿punctulata* (Goetghebuer, 1934), in Kettani et al. 2001: 327

Ashe and Cranston 1990; Azzouzi et al. 1992, **HA**, Oued Tensift; Kettani et al. 2001; Ashe and O’Connor 2009; Kettani and Langton 2012; Kettani and Moubayed-Breil 2018, **Rif**

##### Diamesini

﻿***Diamesa* Meigen, 1835**

﻿*Diamesa ﻿﻿﻿aberrata* Lundbeck, 1898

Saether 1968; Serra-Tosio 1973; Fittkau and Reiss 1987; Serra-Tosio 1983; Azzouzi and Laville 1987, **HA** (2500–3350 m); Ashe and Cranston 1990; Kettani et al. 2001; Ashe and O’Connor 2009; Kettani and Langton 2012

﻿*Diamesa ﻿﻿﻿bertrami* Edwards, 1935

Serra-Tosio 1983, **HA**, Gorges de Todra (2500 m); Azzouzi and Laville 1987, **HA**, Gorges Todra; Ashe and Cranston 1990; Kettani et al. 2001; Ashe and O’Connor 2009; Kettani and Langton 2012

﻿*Diamesa ﻿﻿﻿hamaticornis* Kieffer, 1924

Reiss 1977; Serra-Tosio 1983, **HA**, M’Goum; Azzouzi and Laville 1987, **HA**, M’Goum; Kettani et al. 2001; Ashe and O’Connor 2009; Kettani and Langton 2012; Kettani and Moubayed-Breil 2018, **Rif**

﻿*Diamesa ﻿﻿﻿insignipes* Kieffer, 1908

Serra-Tosio 1983, **HA** (2500 m); Azzouzi and Laville 1987; Naya 1988, **MA**, Haut and Moyen Sebou; Ashe and Cranston 1990; Kettani et al. 2001; Ashe and O’Connor 2009; Kettani and Langton 2012; Kettani and Moubayed-Breil 2018, **Rif**; Moubayed-Breil and Kettani 2019, **Rif**, Chrafate, Challal Sghir (Akchour)

﻿*Diamesa ﻿﻿﻿latitarsis* (Goetghebuer, 1921)

Vaillant 1955b; Vaillant 1956b, **HA**, Asif Tessaout (M’Goum), Lac Tamhda (Anremer); Serra-Tosio 1967; Serra-Tosio 1967; Saether 1968; Serra-Tosio 1973; Azzouzi and Laville 1987, **HA**; Ashe and Cranston 1990; Kettani et al. 2001, Ashe and O’Connor 2009; Kettani and Langton 2012; Kettani and Moubayed-Breil 2018, **Rif**

﻿*Diamesa ﻿﻿﻿steinboecki* Goetghebuer, 1933

Vaillant 1956b, **HA**, Cascade Siroua, Oukaimeden, Sidi Chamarouch

﻿*Diamesa ﻿﻿﻿tonsa* (Haliday in Walker, 1856)

= ﻿*Diamesathienemanni* Kieffer, 1909

Naya 1988, **MA**, Haut Sebou (Arhbalou Yahya, Oued Arbi, Pont Aït hamza); Kettani et al. 2001; Ashe and O’Connor 2009; Kettani and Langton 2011, **Rif**, Oued Ketama, Oued Sgara; Kettani and Langton 2012

﻿*Diamesa ﻿﻿﻿vaillanti* Serra-Tosio, 1972

Azzouzi et al. 1992, **HA**, Oued Tensift; Kettani et al. 2001; Ashe and O’Connor 2009; Kettani and Langton 2012

﻿*Diamesa ﻿﻿﻿veletensis* Serra-Tosio, 1971

Serra-Tosio 1983, **HA** (2500 m); Azzouzi and Laville 1987, **HA**; Ashe and Cranston 1990; Kettani et al. 2001; Ashe and O’Connor 2009; Kettani and Langton 2012; Kettani and Moubayed-Breil 2018, **Rif**

﻿*Diamesa ﻿﻿﻿zernyi* Edwards, 1933

Azzouzi et al. 1992, **HA**, Oued Tensift; Kettani et al. 2001; Ashe and O’Connor 2009; Kettani and Langton 2012

﻿***Potthastia* Kieffer, 1922**

﻿*Potthastia ﻿﻿﻿gaedii* (Meigen, 1838)

Azzouzi and Laville 1987, **MA**, oued Boufekrane, Oued Fès, Oued Oum-er-Rbia; Kettani et al. 1994, **Rif**, Haut Laou, Oued Siflaou, Oued Moulay Bouchta, aval Oued Talembote (usine éléctrique), Oued Afertane, aval Oued Laou; Kettani et al. 1995, **Rif**, aval Oued El Kbir, Oued El Kbir, amont Oued Nakhla, Oued Mhajrat; Kettani et al. 1996; Dakki 1997; Kettani et al. 2001; Ashe and O’Connor 2009; Kettani et al. 2010, **Rif**, Oued Laou, Oued Afertane; Kettani and Langton 2012; Kettani and Moubayed-Breil 2018, **Rif**; Moubayed-Breil and Kettani 2019, **Rif**, Chrafate, Challal Sghir (Akchour)

﻿*Potthastia ﻿﻿﻿pastoris* (Edwards, 1933)

Kettani and Moubayed-Breil 2018, **Rif**

﻿***Pseudodiamesa* Goetghebuer, 1939**

﻿*Pseudodiamesa (Pseudodiamesa) ﻿branickii* (Nowicki, 1873)

Naya 1988, **MA**, Haut Sebou; Ashe and Cranston 1990; Dakki et al. 2008, **MA**, Oued Sebou; Kettani et al. 2001; Kettani and Langton 2012

﻿*Pseudodiamesa (Pseudodiamesa) ﻿nivosa* (Goetghebuer, 1928)

Naya 1988, **MA**, Moyen Sebou; Kettani et al. 2001; Dakki et al. 2008, **MA**, Oued Sebou; Ashe and O’Connor 2009; Kettani and Langton 2012

﻿***Sympothastia* Pagast, 1947**

﻿*Sympothastia ﻿﻿﻿zavreli* Pagast, 1947

Azzouzi et al. 1992, **HA**, Oued Tensift; Kettani et al. 1994, **Rif**, Haut Laou; Kettani et al. 1995, **Rif**, aval Oued Krikra; Kettani et al. 1996; Dakki 1997; Kettani et al. 2001; Ashe and O’Connor 2009; Kettani and Langton 2012; Kettani and Moubayed-Breil 2018, **Rif**

﻿***Syndiamesa* Kieffer, 1918**

﻿*Syndiamesa ﻿﻿﻿hygropterica* (Kieffer, 1909)

Naya 1988, **MA**, Moyen Sebou (Sidi Abdellah, Dar El Arsa, Pont Oulad Slimane, Pont Portugais); Ashe and Cranston 1990; Kettani et al. 2001; Ashe and O’Connor 2009; Kettani and Langton 2012

##### Protanypini

﻿***Protanypus* Kieffer, 1906**

﻿*Protanypus ﻿﻿﻿morio* (Zetterstedt, 1838)

Naya 1988, **MA**, Moyen Sebou; Dakki et al. 2008, **MA**, Oued Sebou; Kettani et al. 2001; Ashe and O’Connor 2009; Kettani and Langton 2012

##### Prodiamesinae

﻿***Odontomesa* Pagast, 1947**

﻿*Odontomesa ﻿﻿﻿fulva* (Kieffer, 1919)

Azzouzi and Laville 1987, **MA**, Oued Oum-er-Rbia; Kettani et al. 2001; Ashe and O’Connor 2009; Kettani and Langton 2012

﻿***Prodiamesa* Kieffer, 1906**

﻿*Prodiamesa ﻿﻿﻿olivacea* (Meigen, 1818)

Naya 1988, **MA**, Haut Sebou (Haut Guigou); Azzouzi et al. 1992, **HA**, Oued Tensift; Kettani et al. 1994, **Rif**, Haut Laou; Kettani et al. 1996; Dakki 1997; Kettani et al. 1997, **Rif**, Ras el Ma (Chefchaouen); Kettani et al. 2001; Ashe and O’Connor 2009; Kettani et al. 2010, **Rif**, Maggou village, Ifansa; Kettani and Langton 2012; Kettani and Moubayed-Breil 2018, **Rif**

##### Orthocladiinae

Orthocladiini

﻿***Acricotopus* Kieffer, 1921**

﻿*Acricotopus ﻿﻿﻿lucens* (Zetterstedt, 1850)

Kettani and Moubayed-Breil 2018, **Rif**

﻿***Brilla* Kieffer, 1913**

*Brillia ﻿﻿﻿bifida* (Kieffer, 1909)

= ﻿*Brilla ﻿﻿﻿modesta* (Meigen, 1830)

Azzouzi et al. 1992, **HA**, Oued Tensift; Kettani et al. 1996; Kettani et al. 1997, **Rif**, Ras el Ma (Chefchaouen); Kettani and El Ouazzani 2005, **Rif**, amont Oued Nakhla; Kettani et al. 2001; Kettani et al. 2010, **Rif**, Oued Tamaridine (Zaouiet El Habtiyine, 819 m); Kettani and Langton 2012; Ashe and O’Connor 2012; Kettani and Moubayed-Breil 2018, **Rif**

*Brillia ﻿﻿﻿flavifrons* (Johannsen, 1905)

Kettani and Langton 2012

﻿*Brilla ﻿﻿﻿longifurca* Kieffer, 1921

Azzouzi and Laville 1987, **MA**, Oued Boufekrane, Oued Sebou, Oued Oum-er-Rbia; Kettani et al. 1995, **Rif**, amont Oued Nakhla, Oued Mhajrat; Kettani et al. 1996; Kettani et al. 2001; Dakki et al. 2008, **MA**, Oued Sebou; Kettani et al. 2010, **Rif**, Oued Talembote (avant village Talembote, 320 m), Oued Talembote (Usine électrique, 120 m); Kettani and Langton 2012; Ashe and O’Connor 2012

﻿***Bryophaenocladius* Thienemann, 1934**

﻿*Bryophaenocladius ﻿﻿﻿aestivus* (Brundin, 1947)

Kettani and Moubayed-Breil 2018, **Rif**; Moubayed-Breil and Kettani 2019, **Rif**, Chrafate, Challal Sghir (Akchour)

﻿*Bryophaenocladius ﻿﻿﻿flexidens* (Brundin, 1947)

Kettani and Moubayed-Breil 2018, **Rif**

﻿*Bryophaenocladius* ﻿﻿cf. ﻿*furcatus* Thienemann & Strenzke, 1940

Kettani and Moubayed-Breil 2018, **Rif**

﻿*Bryophaenocladius ﻿﻿﻿illimbatus* (Edwards, 1929)

Kettani and Moubayed-Breil 2018, **Rif**; Moubayed-Breil and Kettani 2019, **Rif**, Chrafate, Challal Sghir (Akchour)

﻿*Bryophaenocladius ﻿﻿﻿muscicola* (Kieffer, 1906)

Kettani and Moubayed-Breil 2018, **Rif**

﻿*Bryophaenocladius ﻿﻿﻿nidorum* (Edwards, 1929)

Kettani and Moubayed-Breil 2018, **Rif**

﻿*Bryophaenocladius ﻿﻿﻿subvernalis* (Edwards, 1929)

Azzouzi and Laville 1987, **MA**, Oued Boufekrane; Kettani et al. 2001; Kettani and Langton 2011, **Rif**, Oued Taida (Moulay Abdelsalam); Kettani and Langton 2012; Ashe and O’Connor 2012; Kettani and Moubayed-Breil 2018, **Rif**; Moubayed-Breil and Kettani 2019, **Rif**, Chrafate, Challal Sghir (Akchour)

﻿*Bryophaenocladius ﻿﻿﻿tuberculatus* (Edwards 1929)

Kettani and Moubayed-Breil 2018, **Rif**

﻿***Camptocladius* Wulp, 1874**

﻿*Camptocladius ﻿﻿﻿stercorarius* (De Geer, 1976)

Kettani and Moubayed-Breil 2018, **Rif**

﻿***Cardiocladius* Kieffer, 1912**

﻿*Cardiocladius ﻿﻿﻿capucinus* (Zetterstedt, 1850)

Azzouzi and Laville 1987, **MA**, Oued Oum-er-Rbia; Kettani et al. 1994, **Rif**, Haut Laou, Oued Siflaou, Oued Moulay Bouchta, aval Barrage Talembote, Oued Afertane, aval Oued Tassikeste; Kettani et al. 1995, **Rif**, aval Oued El Kbir, aval Oued Krikra, amont Oued Nakhla, Oued Mhajrat, aval Oued Khemis; Kettani et al. 1996; Kettani et al. 2001; Kettani and El Ouazzani 2005, **Rif**, amont Oued Nakhla; Kettani et al. 2010, **Rif**, Oued Talembote (aval Barrage Talembote, 245 m), Oued Tassikeste (Afechtal, 240 m), Oued Laou (Afertane, 55 m); Kettani and Langton 2012; Ashe and O’Connor 2012; Kettani and Moubayed-Breil 2018, **Rif**

﻿*Cardiocladius ﻿﻿﻿fuscus* Kieffer, 1924

Azzouzi and Laville 1987, **MA**, Oued Boufekrane, Oued Oum-er-Rbia; Naya 1988, **MA**, Haut Sebou (Amont de Aïn Tadout, Skhounate, amont confluence avec Oued Atchane, Pont Aït Hamza); Kettani et al. 1994, **Rif**, Oued Siflaou; Kettani et al. 1996; Dakki 1997; Kettani et al. 2001; Kettani and El Ouazzani 2005, **Rif**, amont Oued Nakhla; Dakki et al. 2008, **MA**, Oued Sebou; Kettani and Langton 2012; Ashe and O’Connor 2012; Kettani and Moubayed-Breil 2018, **Rif**

﻿***Chaetocladius* Kieffer, 1911**

﻿*Chaetocladius (Chaetocladius) acuticornis* (Kieffer in Potthast, 1914)

Azzouzi et al. 1992, **HA**, Oued Tensift; Kettani et al. 2001; Kettani and Langton 2012; Kettani and Moubayed-Breil 2018, **Rif**

﻿*Chaetocladius ﻿﻿﻿dentiforceps* (Edwards, 1929)

Kettani and Moubayed-Breil 2018, **Rif**; Moubayed-Breil and Kettani 2019, **Rif**, Chrafate, Challal Sghir (Akchour)

﻿*Chaetocladiusdissipatus* (Edwards, 1929)

Moubayed-Breil and Kettani 2019, **Rif**, Chrafate, Challal Sghir (Akchour)

﻿*Chaetocladius (Chaetocladius) ﻿melaleucus* (Meigen, 1818)

Kettani and Langton 2011, **Rif**, Oued Sgara, Bab Tariouant, Bouztata; Kettani and Langton 2012; Ashe and O’Connor 2012; Kettani and Moubayed-Breil 2018, **Rif**

﻿*Chaetocladius ﻿﻿﻿piger* (Goetghebuer, 1913)

Kettani and Moubayed-Breil 2018, **Rif**

﻿*Chaetocladius (Chaetocladius) ﻿perennis* (Meigen, 1830)

Kettani and Langton 2011, **Rif**, Oued Hamma; Kettani and Langton 2012

﻿*Chaetocladius (Chaetocladius) ﻿vitellinus* (Kieffer in Kieffer & Thienemann, 1908)

Azzouzi et al. 1992, **HA**, Oued Tensift; Kettani et al. 2001; Kettani and Langton 2012

﻿***Corynoneura* Winnertz, 1846**

﻿*Corynoneura ﻿﻿﻿carriana* Edwards, 1924

Naya 1988, **MA**, Haut Sebou (Haut Guigou, Aïn Nokra); Kettani et al. 1995, **Rif**, aval Oued El Kbir, aval Oued Krikra, amont Oued Nakhla, Oued Mhajrat, aval Oued Khemis; Kettani et al. 1996; Kettani et al. 2001; Kettani and El Ouazzani 2005, **Rif**, amont Oued Nakhla; Dakki et al. 2008, **MA**, Oued Sebou; Kettani and Langton 2012; Ashe and O’Connor 2012; Kettani and Moubayed-Breil 2018, **Rif**

﻿*Corynoneura ﻿﻿﻿celtica* Edwards, 1924

Kettani and Langton 2011, **Rif**, Oued Hamma; Kettani and Langton 2012; Ashe and O’Connor 2012; Kettani and Moubayed-Breil 2018, **Rif**

﻿*Corynoneura ﻿﻿﻿coronata* Edwards, 1924

Kettani and Langton 2011, **Rif**, Oued Hamma; Kettani and Langton 2012; Ashe and O’Connor 2012

﻿*Corynoneura ﻿﻿﻿edwardsi* Brundin, 1949

Kettani and Langton 2012

﻿*Corynoneura ﻿﻿﻿lacustris* Edwards, 1924

El Mezdi and Giudicelli 1985, **HA**, Khettaras de Marrakech; Kettani et al. 2001; Kettani and Langton 2012; Ashe and O’Connor 2012; Kettani and Moubayed-Breil 2018, **Rif**

﻿*Corynoneura ﻿﻿﻿lobata* Edwards, 1924

Kettani et al. 1996, **Rif**, Oued Nakhla; Kettani et al. 2001; Kettani and El Ouazzani 2005, **Rif**, amont Oued Nakhla; Kettani et al. 2010, **Rif**, Oued Kelaâ (Akoumi, 400 m); Azzouzi et al. 1992, **HA**; Kettani and Langton 2012; Ashe and O’Connor 2012; Kettani and Moubayed-Breil 2018, **Rif**

﻿*Corynoneura ﻿﻿﻿scutellata* Winnertz, 1846

Kettani and Moubayed-Breil 2018, **Rif**

﻿*Corynoneura* ﻿Pe 2 Langton 1991

Azzouzi et al. 1992, **HA**, Oued Tensift; Kettani et al. 2001; Kettani and Langton 2012

﻿***Corynoneurella* Brundin, 1949**

﻿*Corynoneurella ﻿﻿﻿paludosa* Brundin, 1949

Kettani et al. 2010, **Rif**, Nord Maggou village (Maggou, 905 m), Oued Kelaâ (Akoumi, 400 m), Oued Talembote (avant village Talembote, 320 m), Oued Laou (Afertane, 55 m); Kettani and Langton 2012; Ashe and O’Connor 2012; Kettani and Moubayed-Breil 2018, **Rif**

﻿***Cricotopus* van der Wulp, 1874**

﻿*Cricotopus (Cricotopus) ﻿albiforceps* (Kieffer in Thienemann and Kieffer 1916)

Kettani et al. 1996; Kettani et al. 1997, **Rif**, Oued Khizana (Oued Laou), Ras el Ma (Chefchaouen); Kettani et al. 2001; Kettani and Langton 2012; Ashe and O’Connor 2012; Kettani and Moubayed-Breil 2018, **Rif**

﻿*Cricotopus (Cricotopus) ﻿annulator* Goetghebuer, 1927

Azzouzi et al. 1992, **HA**, Oued Tensift; Kettani et al. 2001; Kettani et al. 2010, **Rif**, Oued Talembote (avant village Talembote, 320 m), Oued Tassikeste (Afechtal, 240 m), Oued Laou (Afertane, 55 m); Kettani and Langton 2011, **Rif**, Oued Sgara; Kettani and Langton 2012; Ashe and O’Connor 2012; Kettani and Moubayed-Breil 2018, **Rif**

﻿*Cricotopus (Cricotopus) ﻿beckeri* Hirvenoja, 1973

Azzouzi et al. 1992, **HA**, Oued Tensift; Kettani et al. 1995, **Rif**, aval Oued El Kbir; Kettani et al. 1996; Kettani et al. 1997, **Rif**, Maggou (Oued Laou), Oued Khizana (Oued Laou); Kettani et al. 2001; Langton and Laville 2002; Kettani and Langton 2012; Ashe and O’Connor 2012; Kettani and Moubayed-Breil 2018, **Rif**

﻿*Cricotopus (Cricotopus) ﻿bicinctus* (Meigen, 1818)

Azzouzi and Laville 1987, **MA**, Oued Boufekrane; Naya 1988, **MA**, Haut et Moyen Sebou; Kettani et al. 1994, **Rif**, Haut Laou, Oued Siflaou, Oued Moulay Bouchta, aval Barrage Talembote, Oued Afertane, aval Oued Laou; Kettani et al. 1995, **Rif**, aval Oued El Kbir, aval Oued Krikra, Oued El Kbir, amont Oued Nakhla, Oued Mhajrat, aval Oued Khemis, Oued Martil (Tamuda); Kettani et al. 1996; Dakki 1997; Kettani et al. 1997, **Rif**, Maggou (Oued Laou), Oued Khizana (Oued Laou), Ras el Ma (Chefchaouen); Kettani et al. 2001; Dakki et al. 2008, **MA**, Oued Sebou; Kettani et al. 2010, **Rif**, Source Maggou (Maggou, 1300 m), Oued Talembote (aval Barrage Talembote, 245 m), Oued Laou (Afertane, 55 m); Kettani and Langton 2012; Ashe and O’Connor 2012; Kettani and Moubayed-Breil 2018, **Rif**

﻿*Cricotopus (Cricotopus) ﻿caducus* Hirvenoja, 1973

Kettani and Moubayed-Breil 2018, **Rif**

﻿*Cricotopus (Cricotopus) ﻿ephippium* (Zetterstedt, 1838)

Kettani and Moubayed-Breil 2018, **Rif**

﻿*Cricotopus (Cricotopus) ﻿levantinus* Moubayed & Hirvenoja, 1986

Kettani et al. 1996, **Rif**, Haut Maggou; Kettani et al. 1997, **Rif**, Maggou (Oued Laou); Kettani et al. 2001; Laville and Langton 2002; Kettani et al. 2010, **Rif**, Source Maggou (Maggou, 1300 m), Oued Inesmane (Adeldal, 1173 m); Kettani and Langton 2012; Ashe and O’Connor 2012

﻿*Cricotopus (Cricotopus) ﻿pallidipes* Edwards, 1929

Azzouzi et al. 1992, **HA**, Oued Tensift; Kettani et al. 1995, **Rif**, Oued Martil (Tamuda); Kettani et al. 1996; Kettani et al. 1997, **Rif**, Oued Khizana (Oued Laou); Kettani et al. 2001; Kettani and Langton 2012; Ashe and O’Connor 2012; Kettani and Moubayed-Breil 2018, **Rif**

﻿*Cricotopus (Cricotopus) ﻿pulchripes* Verrall, 1912

Kettani and Moubayed-Breil 2018, **Rif**

﻿*Cricotopus (Cricotopus) ﻿similis* Goetgnebuer, 1921

Azzouzi and Laville 1987, **MA**, Oued Oum-er-Rbia; Kettani et al. 1994, **Rif**, Haut Laou, Oued Siflaou, Oued Afertane, aval Oued Talembote (usine éléctrique), aval Oued Tassikeste; Kettani et al. 1995, **Rif**, aval Oued Krikra, Oued El Kbir, amont Oued Nakhla, Oued Mhajrat, aval Oued Khemis; Kettani et al. 1996; Dakki 1997; Kettani et al. 1997, **Rif**, Maggou (Oued Laou), Oued Khizana (Oued Laou), Ras el Ma (Chefchaouen); Kettani et al. 2001; Kettani et al. 2010, **Rif**, Oued Talembote (aval Barrage Talembote, 245 m), Oued Talembote (aval affluent Talembote, 155 m), Oued Laou (Afertane, 55 m); Kettani and Langton 2012; Ashe and O’Connor 2012; Kettani and Moubayed-Breil 2018, **Rif**

﻿*Cricotopus (Cricotopus) ﻿tremulus* (Linnaeus, 1758)

Kettani et al. 2010, **Rif**, Oued Maggou (Maggou village, 777 m); Kettani and Langton 2012; Kettani and Moubayed-Breil 2018, **Rif**

﻿*Cricotopus (Cricotopus) ﻿triannulatus* (Macquart, 1826)

Kettani et al. 1994, **Rif**, Haut Laou, Oued Moulay Bouchta, Oued Afertane; Kettani et al. 1995, **Rif**, aval Oued El Kbir, aval Oued Krikra; Kettani et al. 1996; Dakki 1997; Kettani et al. 2001; Kettani and El Ouazzani 2005, **Rif**, amont Oued Nakhla; Kettani et al. 2010, **Rif**, Oued Talembote (aval affluent Talembote, 155 m); Kettani and Langton 2012; Ashe and O’Connor 2012; Kettani and Moubayed-Breil 2018, **Rif**

﻿*Cricotopus (Cricotopus) ﻿trifascia* Edwards, 1929

Azzouzi and Laville 1987, **MA**, Oued Boufekrane, Oued Fès, Oued Oum-er-Rbia; Kettani et al. 1994, **Rif**, Haut Laou, Oued Siflaou, Oued Moulay Bouchta, aval Barrage Talembote, aval Oued Talembote (usine éléctrique), aval Oued Tassikeste; Kettani et al. 1995, **Rif**, aval Oued Krikra, amont Oued Nakhla, Oued Mhajrat; Kettani et al. 1996; Dakki 1997; Kettani et al. 1997, **Rif**, Maggou (Oued Laou), Oued Khizana (Oued Laou), Ras el Ma (Chefchaouen); Kettani et al. 2001; Dakki et al. 2008, **MA**, Oued Sebou; Kettani et al. 2010, **Rif**, Oued Maggou (Maggou village, 777 m), Oued Talembote (aval Barrage Talembote, 245 m); Kettani and Langton 2012; Ashe and O’Connor 2012; Kettani and Moubayed-Breil 2018, **Rif**

﻿*Cricotopus (Cricotopus) ﻿vierriensis* Goetghebuer, 1935

El Mezdi and Giudicelli 1985, **HA**, Khettaras de Marrakech; Azzouzi and Laville 1987, **MA**, Oued Boufekrane, Oued Fès, Oued Sebou, Oued Oum-er-Rbia, **HA**, Oued Tensift; Kettani et al. 1994, **Rif**, Haut Laou, Oued Siflaou, Oued Moulay Bouchta, aval Barrage Talembote, aval Oued Talembote (usine éléctrique), Oued Afertane, aval Oued Tassikeste, aval Oued Laou; Kettani et al. 1995, **Rif**, aval Oued El Kbir, aval Oued Krikra, Oued El Kbir, amont Oued Nakhla, Oued Mhajrat, aval Oued Khemis; Kettani et al. 1996; Dakki 1997; Kettani et al. 1997, **Rif**, Maggou (Oued Laou), Oued Khizana (Oued Laou), Ras el Ma (Chefchaouen); Kettani et al. 2001; Kettani and El Ouazzani 2005, **Rif**, amont Oued Nakhla; Dakki et al. 2008, **MA**, Oued Sebou; Kettani et al. 2010, **Rif**, Oued Talembote (aval Barrage Talembote, 245 m), Oued Talembote (aval affluent Talembote, 155 m); Kettani and Langton 2012; Ashe and O’Connor 2012; Kettani and Moubayed-Breil 2018, **Rif**

﻿*Cricotopus (Isocladius) ﻿brevipalpis* Kieffer, 1909

Azzouzi et al. 1992, **HA**, Oued Tensift; Kettani et al. 2001; Kettani and Langton 2012; Ashe and O’Connor 2012

﻿*Cricotopus (Isocladius) ﻿laetus* Hirvenoja, 1973

Kettani et al. 1994, **Rif**, Oued Siflaou; Kettani et al. 1996; Dakki 1997; Kettani et al. 2001; Kettani and Langton 2012; Ashe and O’Connor 2012

﻿*Cricotopus (Isocladius) ﻿ornatus* (Meigen, 1818)

Azzouzi et al. 1992, **HA**, Oued Tensift; Kettani et al. 2001; Kettani and Langton 2012; Ashe and O’Connor 2012

﻿*Cricotopus (Isocladius) ﻿sylvestris* (Fabricius, 1794)

Fittkau and Reiss 1978; Ramdani and Tourenq 1982, **AP**, Merja Sidi Boughaba; El Mezdi and Giudicelli 1985, **HA**, Khettaras de Marrakech; Azzouzi and Laville 1987, **MA**, Oued Boufekrane, Oued Fès, **HA**, Oued Tensift; Kettani et al. 1994, **Rif**, Haut Laou; Kettani et al. 1996; Dakki 1997; Kettani et al. 1997, **Rif**, Oued Khizana (Oued Laou); Kettani et al. 2001; Dakki et al. 2008, **MA**, Oued Sebou; Kettani and Langton 2012; Ashe and O’Connor 2012; Kettani and Moubayed-Breil 2018, **Rif**

﻿*Cricotopus (Isocladius) ﻿tricinctus* (Meigen, 1818)

Kettani and Moubayed-Breil 2018, **Rif**

﻿*Cricotopus (Paratrichocladius) ﻿micans* (Kieffer, 1918)

Kettani et al. 1994, **Rif**, Haut Laou, Oued Siflaou, Oued Moulay Bouchta, aval Barrage Talembote, aval Oued Talembote (usine éléctrique), Oued Afertane, aval Oued Tassikeste; Kettani et al. 1995, **Rif**, aval Oued El Kbir, aval Oued Krikra, Oued El Kbir, amont Oued Nakhla, Oued Mhajrat, aval Oued Khemis; Kettani et al. 1996; Kettani et al. 1997, **Rif**, Maggou (Oued Laou); Dakki 1997; Kettani et al. 2001; Kettani and El Ouazzani 2005, **Rif**, amont Oued Nakhla; Kettani et al. 2010, **Rif**, Haut Maggou (1300 m), Oued Talembote, Oued Laou (Afertane, 56 m); Kettani and Langton 2012; Ashe and O’Connor 2012; Kettani and Moubayed-Breil 2018, **Rif**

﻿*Cricotopus (Paratrichocladius) ﻿osellai* Rossaro, 1990

Kettani and Moubayed-Breil 2018, **Rif**

﻿*Cricotopus (Paratrichocladius) ﻿rufiventris* (Meigen, 1830)

El Mezdi and Giudicelli 1985, **HA**, Khettaras de Marrakech; Azzouzi and Laville 1987, **MA**, Oued Fès, Oued Boufekrane, Oued Sebou, Oued Oum-er-Rbia, **HA**, Oued Tensift; (Dar El Arsa, Pont oulad Slimane, Pont portugais); Naya 1988, **MA**, Haut et Moyen Sebou; Kettani et al. 1995, **Rif**, Oued Mhajrat; Kettani et al. 1996; Kettani et al. 2001; Kettani et al. 2010, **Rif**, Oued Kelaâ (Akoumi, 400 m), Oued Talembote; Dakki et al. 2008, **MA**, Oued Sebou; Kettani and Langton 2012; Ashe and O’Connor 2012; Kettani and Moubayed-Breil 2018, **Rif**

﻿*Cricotopus (Paratrichocladius) ﻿skirwithensis* (Edwards, 1929)

Azzouzi et al. 1992, **HA**, Oued Tensift; Kettani et al. 2001; Kettani and Langton 2012; Ashe and O’Connor 2012; Kettani and Moubayed-Breil 2018, **Rif**

﻿***Eukieferiella* Thienemann, 1926**

﻿*Eukieferiella ﻿﻿﻿ancyla* Svensson, 1986

Kettani and Langton 2011, **Rif**, Oued Tkarae; Kettani and Langton 2012; Ashe and O’Connor 2012

*Eukiefferiella ﻿﻿﻿bedmari* Vilchez-Quero & Laville, 1988

Azzouzi and Laville 1987, **MA**, Oued Fès, Oued Oum-er-Rbia; Kettani et al. 1994, **Rif**, Haut Laou, aval Oued Talembote (usine éléctrique), Oued Afertane, aval Oued Tassikeste; Kettani et al. 1995, **Rif**, aval Oued Krikra, amont Oued Nakhla, Oued Mhajrat, aval Oued Khemis; Kettani et al. 1996; Dakki 1997; Kettani et al. 2001; Laville and Langton 2002; Dakki et al. 2008, **MA**, Oued Sebou; Kettani et al. 2010, **Rif**, Oued Kelaâ (Akoumi, 400 m), Oued Talembote (aval Barrage Talembote, 245 m), Oued Talembote (aval affluent Talembote, 155 m); Kettani and Langton 2012; Ashe and O’Connor 2012; Kettani and Moubayed-Breil 2018, **Rif**

*Eukiefferiella ﻿﻿﻿brehmi* Gowin, 1943

Kettani et al. 1996; Kettani et al. 1997, **Rif**, Maggou (Oued Laou); Kettani et al. 2001; Kettani et al. 2010, **Rif**, Source Maggou (Maggou, 1300 m), Oued Kelaâ (Akoumi, 400 m), Oued Talembote (avant village Talembote, 320 m), Oued Talembote (Usine électrique, 120 m)

*Eukiefferiella ﻿﻿﻿brevicalcar* (Kieffer, 1911)

Azzouzi et al. 1992, **HA**, Oued Tensift; Kettani et al. 2001; Kettani et al. 2010, **Rif**, Nord Maggou village (Maggou, 905 m), Oued Ametrasse (Ametrasse, 820 m); Kettani and Langton 2011, **Rif**, Oued Issaguen, Oued Ketama, Oued Sgara; Bab Tariouant, Bouztate (Fifi); Kettani and Langton 2012; Ashe and O’Connor 2012; Kettani and Moubayed-Breil 2018, **Rif**

*Eukiefferiella ﻿﻿﻿claripennis* (Lundbeck, 1898)

Fittkau and Reiss 1978; Naya 1988, **MA**, Moyen Sebou (Dar Cheik Harazem); Azzouzi and Laville 1987, **MA**, Oued Oum-er-Rbia; Kettani et al. 1994, **Rif**, Oued Siflaou; Kettani et al. 1996; Kettani et al. 1997, **Rif**, Oued Khizana (Oued Laou), Ras el Ma (Chefchaouen); Kettani et al. 2001; Kettani and El Ouazzani 2005, **Rif**, amont Oued Nakhla; Dakki et al. 2008, **MA**, Oued Sebou; Kettani and Langton 2012; Ashe and O’Connor 2012; Kettani and Moubayed-Breil 2018, **Rif**

*Eukieffeiella ﻿﻿﻿clypeata* (Thienemann, 1919)

Kettani et al. 1994, **Rif**, Haut Laou, Oued Siflaou, Oued Moulay Bouchta, aval Oued Talembote (usine éléctrique); Kettani et al. 1995, **Rif**, aval Oued El Kbir, aval Oued Krikra, Oued El Kbir, amont Oued Nakhla, Oued Mhajrat; Kettani et al. 1996; Kettani et al. 1997, **Rif**, Maggou (Oued Laou), Oued Khizana (Oued Laou), Ras el Ma (Chefchaouen); Kettani et al. 2001; Kettani and El Ouazzani 2005, **Rif**, amont Oued Nakhla; Kettani et al. 2010, **Rif**, Oued Laou (Afertane, 55 m); Kettani and Langton 2012; Ashe and O’Connor 2012; Kettani and Moubayed-Breil 2018, **Rif**

*Eukiefferiella ﻿﻿﻿coerulescens* (Kieffer in Zavřel, 1926)

Azzouzi and Laville 1987, **MA**, Oued Oum-er-Rbia; Naya 1988, **MA**, Haut Sebou (Skhounate, Arhbalou Aberchane); Kettani et al. 1994, **Rif**, Haut Laou, Oued Siflaou, Oued Moulay Bouchta, aval Barrage Talembote, aval Oued Talembote (usine éléctrique), aval Oued Tassikeste, aval Oued Laou; Kettani et al. 1995, **Rif**, aval Oued El Kbir, aval Oued Krikra, Oued El Kbir, amont Oued Nakhla, Oued Mhajrat, aval Oued Khemis; Kettani et al. 1996; Dakki 1997; Kettani et al. 1997, **Rif**, Maggou (Oued Laou), Oued Khizana (Oued Laou); Kettani et al. 2001; Kettani and El Ouazzani 2005, **Rif**, amont Oued Nakhla; Dakki et al. 2008, **MA**, Oued Sebou; Kettani et al. 2010, **Rif**, Source Maggou (Maggou, 1300 m), Nord Maggou village (Maggou, 905 m), Oued Tamaridine (Zaouiet El Habtiyine, 819 m), Oued Maggou (Maggou village, 777 m), Oued Kelaâ (Akoumi, 400 m), Oued Talembote (aval Barrage Talembote, 245 m), Oued Talembote (aval affluent Talembote, 155 m), Oued Laou (Afertane, 55 m); Kettani and Langton 2012; Ashe and O’Connor 2012; Kettani and Moubayed-Breil 2018, **Rif**

*Eukiefferiella ﻿﻿﻿cyanea* Thienemann, 1936

Vaillant 1955b, **HA**; Fittkau and Reiss 1978; Azzouzi and Laville 1987, **HA**; Kettani et al. 1996, **Rif**, Oued Nakhla; Kettani et al. 2001; Kettani and El Ouazzani 2005, **Rif**, amont Oued Nakhla; Kettani and Langton 2012; Ashe and O’Connor 2012; Kettani and Moubayed-Breil 2018, **Rif**

*Eukiefferiella ﻿﻿﻿devonica* (Edwards, 1929)

Azzouzi and Laville 1987, **MA**, Oued Oum-er-Rbia; Kettani et al. 1995, **Rif**, aval Oued El Kbir, aval Oued Krikra, Oued El Kbir, amont Oued Nakhla, Oued Mhajrat, aval Oued Khemis; Kettani et al. 1996; Kettani et al. 1997, **Rif**, Oued Khizana (Oued Laou); Kettani et al. 2001; Dakki et al. 2008, **MA**, Oued Sebou; Kettani and Langton 2012; Ashe and O’Connor 2012; Kettani and Moubayed-Breil 2018, **Rif**; Moubayed-Breil and Kettani 2019, **Rif**, Chrafate, Challal Sghir (Akchour)

*Eukiefferiella ﻿﻿﻿dittmari* Lehmann, 1972

Kettani and Langton 2011, **Rif**, Oued Boujdad, Fifi; Kettani and Langton 2012, **Rif**, Oued Zarka; Ashe and O’Connor 2012; Kettani and Moubayed-Breil 2018, **Rif**

*Eukiefferiella ﻿﻿﻿fittkaui* Lehmann, 1972

Azzouzi et al. 1992, **HA**, Oued Tensift; Kettani et al. 2001; Kettani and Langton 2012; Ashe and O’Connor 2012

*Eukiefferiella ﻿﻿﻿fuldensis* Lehmann, 1972

Azzouzi and Laville 1987, **MA**, Oued Oum-er-Rbia; Kettani et al. 2001; Kettani and Langton 2012; Ashe and O’Connor 2012; Kettani and Moubayed-Breil 2018, **Rif**; Moubayed-Breil and Kettani 2019, **Rif**, Chrafate, Challal Sghir (Akchour)

*Eukiefferiella ﻿﻿﻿gracei* (Edwards, 1929)

Azzouzi et al. 1992, **HA**, Oued Tensift; Kettani et al. 1994, **Rif**, aval Oued Tassikeste; Kettani et al. 1996; Dakki 1997; Kettani et al. 2001; Kettani and El Ouazzani 2005, **Rif**, amont Oued Nakhla; Kettani et al. 2010, **Rif**, Oued Talembote (aval Barrage Talembote, 245 m); Kettani and Langton 2012; Ashe and O’Connor 2012; Moubayed-Breil and Kettani 2019, **Rif**, Chrafate, Challal Sghir (Akchour)

*Eukiefferiella ﻿﻿﻿ilkleyensis* (Edwards, 1929)

Azzouzi and Laville 1987, **MA**, Oued Oum-er-Rbia; Kettani et al. 2001; Kettani and Langton 2012; Ashe and O’Connor 2012; Kettani and Moubayed-Breil 2018, **Rif**

*Eukiefferiella ﻿﻿﻿lobifera* Goetghebuer, 1934

Azzouzi and Laville 1987, **MA**, Oued Fès, Oued Oum-er-Rbia; Kettani et al. 2001; Kettani and Langton 2011, **Rif**, Oued Ketama, Oued Sgara; Kettani and Langton 2012; Ashe and O’Connor 2012

*Eukiefferiellaminor* (Edwards, 1929)

Vaillant 1955b, **HA** (1050 m); Vaillant 1956b, **HA**, Imi-N’Ifri; Azzouzi and Laville 1987, **HA**; Kettani et al. 2001; Kettani and Langton 2012; Ashe and O’Connor 2012

*Eukiefferiella ﻿﻿﻿pseudomontana* Goetghebuer, 1935

Kettani et al. 2010, **Rif**, Oued Madissouka (Talassemtane, 1530 m), Oued Dchar d’Amran (Béni M’Hamed, 1180 m); Kettani and Langton 2012; Ashe and O’Connor 2012; Kettani and Moubayed-Breil 2018, **Rif**

*Eukiefferiella ﻿﻿﻿similis* Goetghebuer, 1939

Azzouzi and Laville 1987, **MA**, Oued Boufekrane, Oued Fès, Oued Sebou, Oued Oum-er-Rbia; Kettani et al. 2001; Kettani and Langton 2012; Ashe and O’Connor 2012; Kettani and Moubayed-Breil 2018, **Rif**

*Eukiefferiella ﻿﻿﻿tirolensis* Goetghebuer, 1938

Kettani et al. 1994, **Rif**, Oued Afertane; Kettani et al. 1996; Kettani et al. 2001; Kettani et al. 2010, **Rif**, Oued Talembote (aval affluent Talembote, 155 m); Azzouzi et al. 1992, **HA**, Oued Tensift; Dakki 1997; Kettani and Langton 2012; Ashe and O’Connor 2012; Kettani and Moubayed-Breil 2018, **Rif**

*Eukiefferiella* ﻿Pe 2 Langton 1991

Kettani et al. 2010, **Rif**, Oued Kelaâ (Akoumi, 400 m); Kettani and Langton 2012

﻿***Halocladius* Hirvenoja, 1973**

﻿*Halocladius (Halocladius) ﻿varians* (Staeger, 1839)

Ramdani and Tourenq 1982, **AP**, Merja Sidi Boughaba; Azzouzi and Laville 1987; Kettani et al. 1996; Kettani et al. 1997, **Rif**, Maggou (Oued Laou); Kettani et al. 2001; Kettani et al. 2010, **Rif**, Source Maggou (Maggou, 1300 m); Ashe and Cranston 1990; Kettani and Langton 2012; Ashe and O’Connor 2012

﻿***Heleniella* Gowin, 1943**

﻿*Heleniella ﻿﻿﻿dorieri* Serra-Tosio, 1967

Kettani and Langton 2012

﻿*Heleniella ﻿﻿﻿ornaticollis* (Edwards, 1929)

Kettani et al. 1995, **Rif**, aval Oued El Kbir, aval Oued Krikra, amont Oued Nakhla, Oued Mhajrat; Kettani et al. 1996; Kettani et al. 1997, **Rif**, Maggou (Oued Laou); Kettani et al. 2001; Kettani and El Ouazzani 2005, **Rif**, amont Oued Nakhla; Kettani et al. 2010, **Rif**, Source Maggou (Maggou, 1300 m), Oued Kelaâ (Akoumi, 400 m); Kettani and Langton 2012; Ashe and O’Connor 2012

﻿*Heleniella ﻿﻿﻿serratosioi* Ringe, 1976

Kettani and Langton 2011, **Rif**, Oued Hamma, Bouztate (Fifi); Kettani and Langton 2012; Ashe and O’Connor 2012

﻿***Heterotrissocladius* Spärck, 1923**

﻿*Heterotrissocladius ﻿﻿﻿marcidus* (Walker, 1856)

Naya 1988, **MA**, Moyen Sebou; Kettani et al. 2001; Kettani and Langton 2012; Dakki et al. 2008, **MA**, Oued Sebou; Ashe and O’Connor 2012

﻿***Hydrobaenus* Fries, 1830**

﻿*Hydrobaenus ﻿﻿﻿conformis* (Holmgren, 1869)

Kettani and Moubayed-Breil 2018, **Rif**

﻿***Hydrosmittia* Ferrington & Sæther, 2011**

﻿*Hydrosmittia ﻿﻿﻿oxoniana* (Edwards, 1929)

= ﻿*Pseudosmittia ﻿﻿﻿recta* (Edwards, 1929), in Azzouzi and Laville 1987: 218, Kettani et al. 2001: 330, Kettani and Langton 2012: 422

Azzouzi and Laville 1987, **HA**, Oued Tensift; Kettani et al. 2001; Kettani and Langton 2012; Kettani and Moubayed-Breil 2018, **Rif**

﻿*Hydrosmittia ﻿﻿﻿ruttneri* (Strenzke & Thienemann, 1942)

Kettani and Moubayed-Breil 2018, **Rif**

﻿***Krenosmittia* Thienemann & Krüger, 1939**

﻿*Krenosmittia ﻿﻿﻿boreoalpina* (Goetghebuer, 1944)

Kettani et al. 1996, **Rif**, Oued Nakhla; Kettani et al. 2001; Kettani and El Ouazzani 2005, **Rif**, amont Oued Nakhla; Kettani and Langton 2012; Ashe and O’Connor 2012

﻿*Krenosmittia ﻿﻿﻿camptophleps* (Edwards, 1929)

Azzouzi et al. 1992, **HA**, Oued Tensift; Kettani et al. 2001; Kettani and Langton 2011, **Rif**, Oued Ketama, Oued Sgara; Kettani and Langton 2012; Ashe and O’Connor 2012; Kettani and Moubayed-Breil 2018, **Rif**

﻿*Krenosmittia ﻿﻿﻿halvorseni* (Cranston & Sæther, 1986)

Kettani et al. 1995, **Rif**, aval Oued El Kbir; Kettani et al. 1996; Kettani et al. 2001; Kettani and Langton 2012; Ashe and O’Connor 2012

﻿*Krenosmittia ﻿﻿﻿hispanica* Wülker, 1957

Kettani et al. 2001; Laville and Langton 2002; Ashe and O’Connor 2012

﻿***Limnophyes* Eaton, 1875**

﻿*Limnophyes ﻿﻿﻿difficilis* Brunidin, 1947

Kettani and Moubayed-Breil 2018, **Rif**

﻿*Limnophyes ﻿﻿﻿gelasinus* Saether, 1990

Kettani and Moubayed-Breil 2018, **Rif**

﻿*Limnophyes ﻿﻿﻿habilis* (Walker, 1856)

Kettani and Moubayed-Breil 2018, **Rif**

﻿*Limnophyes ﻿﻿﻿madeirae* Sæther, 1985

Kettani and Moubayed-Breil 2018, **Rif**

﻿*Limnophyes ﻿﻿﻿minimus* (Meigen, 1818)

Ramdani and Tourenq 1982, **AP**, Merja Sidi Boughaba; Azzouzi and Laville 1987, **MA**, Lac Aguelmane Azigza; Kettani et al. 2001; Kettani and Langton 2011; Kettani and Langton 2012; Ashe and O’Connor 2012; Kettani and Moubayed-Breil 2018, **Rif**

﻿*Limnophyes ﻿﻿﻿natalensis* (Kieffer, 1914)

Kettani and Moubayed-Breil 2018, **Rif**

﻿*Limnophyes ﻿﻿﻿ninae* Sæther, 1975

Kettani et al. 1996, **Rif**, Oued Nakhla; Kettani et al. 2001; Kettani and El Ouazzani 2005, **Rif**, amont Oued Nakhla; Kettani and Langton 2012; Ashe and O’Connor 2012; Kettani and Moubayed-Breil 2018, **Rif**

﻿*Limnophyes ﻿﻿﻿pentaplastus* (Kieffer, 1921)

Kettani and Moubayed-Breil 2018, **Rif**

﻿*Limnophyes ﻿﻿﻿pumilio* (Holmgren, 1869)

Kettani and Moubayed-Breil 2018, **Rif**

﻿*Limnophyes ﻿﻿﻿punctipennis* (Goetghebuer, 1919)

Kettani and Langton 2012

﻿*Limnophyes* ﻿Pe 1a Langton 1991

Kettani et al. 2010, **Rif**, Oued Talembote (Usine électrique, 120 m); Kettani and Langton 2012

﻿***Metriocnemus* van der Wulp, 1874**

﻿*Metriocnemus (Metriocnemus) ﻿albolineatus* Meigen, 1818

Kettani and Moubayed-Breil 2018, **Rif**

﻿*Metriocnemus (Metriocnemus) ﻿eurynotus* (Holmgren, 1883)

= ﻿*Metriocnemus ﻿﻿﻿hygropetricus* Kieffer, 1912, in Ashe and O’Connor 2012: 372

= ﻿*Metriocnemus (Metriocnemus) ﻿obscuripes* (Holmgren, 1869), in Azzouzi et al. 1992: 229, Kettani et al. 2001: 329, Kettani and Langton 2012: 421

Boumezzough and Thomas 1987, **HA**, Oued Réghaya (1740 m), Imlil; Azzouzi and Laville 1987, **HA**, Oued Tensift; Azzouzi et al. 1992, **HA**, Oued Tensift; Kettani et al. 2001; Kettani and Langton 2012; Ashe and O’Connor 2012; Kettani and Moubayed-Breil 2018, **Rif**; Moubayed-Breil and Kettani 2019, **Rif**, Chrafate, Challal Sghir (Akchour)

﻿*Metriocnemus (Metriocnemus) ﻿fuscipes* (Meigen, 1818)

Azzouzi and Laville 1987, **HA**, Oued Tensift; Kettani et al. 2001; Kettani and Langton 2012; Ashe and O’Connor 2012; Kettani and Moubayed-Breil 2018, **Rif**

﻿*Metriocnemus (Metriocnemus) ﻿hirticollis* (Staeger, 1839)

Kettani and Moubayed-Breil 2018, **Rif**

﻿*Metriocnemus (Metriocnemus) ﻿ursinus* Holmgren, 1869

Kettani and Moubayed-Breil 2018, **Rif**

﻿***Nanocladius* Kieffer, 1913**

﻿*Nanocladius (Nanocladius) ﻿balticus* (Palmén, 1959)

Kettani et al. 1995, **Rif**, Oued El Kbir, amont Oued Nakhla; Kettani et al. 1996; Kettani et al. 2001; Kettani and Langton 2012; Ashe and O’Connor 2012; Kettani and Moubayed-Breil 2018, **Rif**

﻿*Nanocladius (Nanocladius) ﻿dichromus* (Kieffer 1906)

Kettani and Moubayed-Breil 2018, **Rif**

﻿*Nanocladius (Nanocladius) ﻿parvulus* (Kieffer 1909)

Kettani and Moubayed-Breil 2018, **Rif**

﻿*Nanocladius (Nanocladius) ﻿rectinervis* (Kieffer, 1911)

Azzouzi and Laville 1987, **MA**, Oued Boufekrane, Oued Oum-er-Rbia; Kettani et al. 1994, **Rif**, Haut Laou, Oued Siflaou, aval Oued Tassikeste; Kettani et al. 1995, **Rif**, aval Oued El Kbir, aval Oued Krikra, Oued El Kbir, amont Oued Nakhla, Oued Mhajrat; Kettani et al. 1996; Dakki 1997; Kettani et al. 1997, **Rif**, Maggou (Oued Laou), Oued Khizana (Oued Laou); Kettani et al. 2001; Kettani and El Ouazzani 2005, **Rif**, amont Oued Nakhla; Kettani et al. 2010, **Rif**, Source Maggou (Maggou, 1300 m), Oued Inesmane (Adeldal, 1173 m), Oued Talembote (aval Barrage Talembote, 245 m); Dakki et al. 2008, **MA**, Oued Sebou; Kettani and Langton 2012; Ashe and O’Connor 2012; Kettani and Moubayed-Breil 2018, **Rif**

﻿***Orthocladius* van der Wulp, 1874**

﻿*Orthocladius (Eudactylocladius) ﻿fuscimanus* (Kieffer, 1908)

Azzouzi et al. 1992, **HA**, Oued Tensift; Kettani et al. 1994, **Rif**, Haut Laou, Oued Siflaou, aval Oued Talembote (usine éléctrique); Kettani et al. 1995, **Rif**, aval Oued Krikra, Oued Mhajrat; Kettani et al. 1996; Dakki 1997; Kettani et al. 1997, **Rif**, Maggou (Oued Laou), Ras el Ma (Chefchaouen); Kettani et al. 2001; Kettani et al. 2010, **Rif**, source Maggou (Maggou, 1300 m), Oued Chrafat (Armotah, 900 m), Oued Tamaridine (Zaouiet El Habtiyine, 819 m), Oued Talembote (Usine électrique, 120 m), Oued Laou (Afertane, 55 m); Kettani and Langton 2012; Ashe and O’Connor 2012; Kettani and Moubayed-Breil 2018, **Rif**

﻿*Orthocladius (Euorthocladius) ﻿ashei* Soponis, 1990

= ﻿*Orthocladius ﻿﻿﻿luteipes* Goetghebuer, in Azzouzi and Laville 1987: 218

= ﻿*Orthocladius ﻿﻿﻿rivicola* Kieffer, in Azzouzi and Laville 1987: 218

Azzouzi and Laville 1987, **MA**, Oued Fès, Oued boufekrane, Oued Sebou, Oued Oum-er-Rbia; Kettani et al. 1994, **Rif**, aval Oued Talembote (usine éléctrique), aval Oued Tassikeste, aval Oued Laou; Kettani et al. 1995, **Rif**, aval Oued El Kbir, aval Oued Krikra, Oued El Kbir, amont Oued Nakhla, Oued Mhajrat, aval Oued Khemis; Kettani et al. 1996; Dakki 1997; Kettani et al. 2001; Kettani and El Ouazzani 2005, **Rif**, amont Oued Nakhla; Dakki et al. 2008, **MA**, Oued Sebou; Kettani et al. 2010, **Rif**, Oued Talembote (avant village Talembote, 320 m), Oued Kanar (Gorges Kanar, 280 m), Oued Talembote (aval Barrage Talembote, 245 m), Oued Talembote (Usine électrique, 120 m), Oued Laou (Afertane, 55 m); Kettani and Langton 2011, **Rif**, Bouztate (Fifi); Kettani and Langton 2012; Ashe and O’Connor 2012; Kettani and Moubayed-Breil 2018, **Rif**

﻿*Orthocladius (Euorthocladius) ﻿rivulorum* Kieffer, 1909

Azzouzi et al. 1992, **HA**, Oued Tensift; Kettani et al. 1994, **Rif**, Haut Laou; Kettani et al. 1995, **Rif**, Oued Mhajrat, aval Oued Khemis; Kettani et al. 1996; Dakki 1997; Kettani et al. 2001; Kettani and Langton 2012; [Bibr B47][Bibr B599], **Rif**

﻿*Orthocladius (Euorthocladius) thienemanni* Kieffer, 1906

Azzouzi et al. 1992, **HA**, Oued Tensift; Kettani et al. 1994, **Rif**, aval Barrage Talembote, aval Oued Talembote (usine éléctrique), aval Oued Tassikeste; Kettani et al. 1995, **Rif**, aval Oued El Kbir, aval Oued Krikra, Oued El Kbir, amont Oued Nakhla, Oued Mhajrat, aval Oued Khemis; Kettani et al. 1996; Dakki 1997; Kettani et al. 2001; Kettani and El Ouazzani 2005, **Rif**, amont Oued Nakhla; Kettani et al. 2010, **Rif**, Oued Tamaridine (Zaouiet El Habtiyine, 819 m), Oued Kelaâ (Akoumi, 400 m), Oued Talembote (avant village Talembote, 320 m), Oued Talembote (aval Barrage Talembote, 245 m), Oued Tassikeste (Afechtal, 240 m), Oued Talembote (Usine électrique, 120 m), Oued Laou (Afertane, 55 m); Kettani and Langton 2012; Ashe and O’Connor 2012; Kettani and Moubayed-Breil 2018, **Rif**

﻿*Orthocladius (Mesorthocladius) ﻿frigidus* (Zetterstedt, 1838)

Vaillant 1955, **HA** (2900 m); Vaillant 1956b, **HA**, Lac Tamhda (Anremer); Azzouzi and Laville 1987; Naya 1988, **MA**, Haut Sebou (Haut Guigou); Fekhaoui et al. 1993; Kettani et al. 1994, **Rif**, Haut Laou; Kettani et al. 1996; Dakki 1997; Kettani et al. 1997, **Rif**, Ras el Ma (Chefchaouen); Kettani et al. 2001; Kettani and El Ouazzani 2005, **Rif**, amont Oued Nakhla; Dakki et al. 2008, **MA**, Oued Sebou; Kettani et al. 2010, **Rif**, Oued Dchar d’Amran (Béni M’Hamed, 1180 m), Nord Maggou village (Maggou, 905 m), Oued Tamaridine (Zaouiet El Habtiyine, 819 m), Oued Maggou (Maggou village, 777 m); Kettani and Langton 2012; Ashe and O’Connor 2012; Kettani and Moubayed-Breil 2018, **Rif**; Moubayed-Breil and Kettani 2019, **Rif**, Chrafate, Challal Sghir (Akchour)

﻿*Orthocladius (Orthocladius) ﻿oblidens* (Walker, 1856)

Azzouzi et al. 1992, **HA**, Oued Tensift; Kettani et al. 2001; Kettani and Langton 2011, **Rif**, Bouztate (Fifi); Kettani and Langton 2012; Ashe and O’Connor 2012; Kettani and Moubayed-Breil 2018, **Rif**

﻿*Orthocladius (Orthocladius) ﻿obumbratus* Johannsen, 1905

= ﻿*Orthocladiusexcavatus* Brundin, in Azzouzi and Laville 1987: 218

Azzouzi and Laville 1987, **MA**, Oued Fès, Oued Oum-er-Rbia; Kettani et al. 1994, **Rif**, Haut Laou, Oued Siflaou, Oued Moulay Bouchta, aval Barrage Talembote, aval Oued Talembote (usine éléctrique), Oued Afertane, aval Oued Tassikeste, aval Oued Laou; Kettani et al. 1995, **Rif**, aval Oued El Kbir, aval Oued Krikra, Oued El Kbir, amont Oued Nakhla, Oued Mhajrat, aval Oued Khemis, Oued Martil (Tamuda); Kettani et al. 1996; Dakki 1997; Kettani et al. 2001; Dakki et al. 2008, **MA**, Oued Sebou; Kettani et al. 2010, **Rif**, Oued Talembote (avant village Talembote, 320 m), Oued Talembote (aval Barrage Talembote, 245 m), Oued Talembote (aval affluent Talembote, 155 m), Oued Laou (Afertane, 55 m); Kettani and Langton 2012

﻿*Orthocladius (Orthocladius) ﻿pedestris* Kieffer, 1909

Kettani et al. 2010, **Rif**, Oued Tassikeste (Afechtal, 240 m), Oued Laou (Afertane, 55 m); Kettani and Langton 2012; Ashe and O’Connor 2012; Kettani and Moubayed-Breil 2018, **Rif**

﻿*Orthocladius (Orthocladius) ﻿rubicundus* (Meigen, 1818)

= ﻿*Orthocladiussaxicola* Kieffer, in Azzouzi and Laville 1987: 218

Azzouzi and Laville 1987, **MA**, Oued Boufekrane, Oued Sebou, Oued Oum-er-Rbia, **HA**, Oued Tensift; Kettani et al. 1994, **Rif**, Haut Laou, Oued Siflaou, Oued Moulay Bouchta, aval Barrage Talembote, aval Oued Talembote (usine éléctrique), Oued Afertane, aval Oued Tassikeste, aval Oued Laou; Kettani et al. 1995, **Rif**, aval Oued El Kbir, aval Oued Krikra, Oued El Kbir, amont Oued Nakhla, Oued Mhajrat, aval Oued Khemis, Oued Martil (Tamuda); Kettani et al. 1996; Dakki 1997; Kettani et al. 1997, **Rif**, Maggou (Oued Laou), Oued Khizana (Oued Laou), Ras el Ma (Chefchaouen); Kettani et al. 2001; Kettani and El Ouazzani 2005, **Rif**, amont Oued Nakhla; Dakki et al. 2008, **MA**, Oued Sebou; Kettani et al. 2010, **Rif**, Oued Talembote (avant village Talembote, 320 m), Oued Kanar (Gorges Kanar, 280 m), Oued Talembote (aval Barrage Talembote, 245 m), Oued Talembote (Usine électrique, 120 m), Oued Laou (Afertane, 55 m); Kettani and Langton 2012; Ashe and O’Connor 2012; Kettani and Moubayed-Breil 2018, **Rif**

﻿*Orthocladius (Orthocladius) ﻿vaillanti* Langton & Cranston, 1991

Kettani and Moubayed-Breil 2018, **Rif**

﻿*Orthocladius (Symposiocladius) ﻿lignicola* Kieffer in Potthast, 1914

= ﻿*Symposiocladius ﻿﻿﻿lignicola* Kieffer, in Kettani et al. 2010: 70

Kettani et al. 2010, **Rif**, Oued Kelaâ (Akoumi, 400 m); Kettani and Langton 2012; Ashe and O’Connor 2012

﻿*Orthocladius (Symposiocladius) ﻿ruffoi* Rossaro & Prato, 1991

= ﻿*Orthocladius* ﻿Pe 1 Langton 1991, in Azzouzi and Laville 1987: 218

= *Rheortocladius* ﻿sp A Langton 1991, in Kettani et al. 1995: 257

= *Rheorthocladius ﻿﻿﻿ruffoi* Rossaro, in Kettani et al. 1997: 184

Azzouzi and Laville 1987, **MA**, Oum-er-Rbia; Kettani et al. 1996; Kettani et al. 1997, **Rif**, Maggou (Oued Laou), Ras el Ma (Chefchaouen); Kettani et al. 2001; Kettani and El Ouazzani 2005, **Rif**, amont Oued Nakhla; Kettani et al. 2010, **Rif**, Oued Tamaridine (Zaouiet El Habtiyine, 819 m), Oued Maggou (Maggou village, 777 m), Oued Kelaâ (Akoumi, 400 m), Oued Talembote (avant village Talembote, 320 m), Oued Kanar (Gorges Kanar, 280 m), Oued Talembote (aval Barrage Talembote, 245 m), Oued Tassikeste (Afechtal, 240 m), Oued Talembote (aval affluent Talembote, 155 m), Oued Laou (Ifansa, 105 m), Oued Laou (Afertane, 55 m); Kettani and Langton 2012; Ashe and O’Connor 2012

﻿***Paracricotopus* Brundin, 1956**

﻿*Paracricotopus ﻿﻿﻿niger* (Kieffer, 1913)

Azzouzi and Laville 1987, **MA**, Oued Boufekrane, Oued Fès; Kettani et al. 1994, **Rif**, Oued Afertane; Kettani et al. 1995, **Rif**, amont Oued Nakhla, Oued Mhajrat, aval Oued Khemis; Kettani et al. 1996; Dakki 1997; Kettani et al. 1997, **Rif**, Maggou (Oued Laou); Dakki et al. 2008, **MA**, Oued Sebou; Kettani et al. 2001; Kettani et al. 2010, **Rif**, Haut Maggou, Oued Tamaridine (Zaouit et Habtyiène, 819 m), Oued Kelaâ (Akoumi, 400 m), Oued Kanar (Gorges Kanar, 280 m), Oued Talembote (155 m), Oued Tassikeste (240 m), Oued Laou (Ifansa, 105 m); Kettani and Langton 2012; Ashe and O’Connor 2012; Kettani and Moubayed-Breil 2018, **Rif**

﻿***Parakiefferiella* Thienemann, 1936**

﻿*Parakiefferiella ﻿﻿﻿coronata* (Edwards, 1929)

Azzouzi et al. 1992, **HA**, Oued Tensift; Kettani et al. 2001; Kettani and Langton 2012; Ashe and O’Connor 2012

﻿*Parakiefferiella ﻿﻿﻿wuelkeri* Moubayed, 1994

= ﻿*Parakiefferiella* ﻿﻿sp. d Wülker, in Azzouzi et al. 1992: 230

Azzouzi et al. 1992, **HA**, Oued Tensift; Kettani et al. 1995, **Rif**, aval Oued El Kbir; Kettani et al. 1996; Kettani et al. 2001; Kettani and Langton 2012; Ashe and O’Connor 2012

﻿***Parametriocnemus* Goetghebuer, 1932**

﻿*Parametriocnemus ﻿﻿﻿boreoalpinus* Gowin & Thienemann, 1942

Kettani and Langton 2011, **Rif**, Oued Taida (Moulay Abdelsalam); Kettani and Langton 2012; Ashe and O’Connor 2012

﻿*Parametriocnemus ﻿﻿﻿stylatus* (Spärck, 1923)

Azzouzi and Laville 1987, **MA**, Oued Boufekrane, Oued Fès, Oued Oum-er-Rbia; Kettani et al. 1994, **Rif**, Haut Laou, Oued Siflaou, Oued Moulay Bouchta, aval Barrage Talembote, aval Oued Talembote (usine éléctrique), Oued Afertane, aval Oued Tassikeste; Kettani et al. 1995, **Rif**, aval Oued El Kbir, aval Oued Krikra, Oued El Kbir, amont Oued Nakhla, Oued Mhajrat, aval Oued Khemis; Kettani et al. 1996; Dakki 1997; Kettani et al. 1997, **Rif**, Maggou (Oued Laou), Oued Khizana (Oued Laou), Ras el Ma (Chefchaouen); Kettani et al. 2001; Kettani and El Ouazzani 2005, **Rif**, amont Oued Nakhla; Dakki et al. 2008, **MA**, Oued Sebou; Kettani et al. 2010, **Rif**, Oued Béni M’Hamed (1330 m), Haut Maggou (1300 m), Oued Kelaâ (Akoumi, 400 m), Oued Talembote (320 m), Oued Tassikeste (Afechtal, 240 m), Oued Laou (Afertane, 56 m); Kettani and Langton 2012; Ashe and O’Connor 2012; Kettani and Moubayed-Breil 2018, **Rif**; Moubayed-Breil and Kettani 2019, **Rif**, Chrafate, Challal Sghir (Akchour)

﻿*Parametriocnemus ﻿﻿﻿valescurensis* Moubayed & Langton, 1999

Kettani and Langton 2011, **Rif**, Oued Issaguen; Kettani and Langton 2012; Ashe and O’Connor 2012; Kettani and Moubayed-Breil 2018, **Rif**

﻿*Parametriocnemus* ﻿Pe 1 Langton 1991

Kettani et al. 1995, **Rif**, aval Oued El Kbir, aval Oued Krikra, Oued El Kbir, amont Oued Nakhla, Oued Mhajrat, aval Oued Khemis; Kettani et al. 1996; Kettani et al. 1997, **Rif**, Maggou (Oued Laou), Oued Khizana (Oued Laou), Ras el Ma (Chefchaouen); Kettani et al. 2001; Kettani and El Ouazzani 2005, **Rif**, amont Oued Nakhla; Kettani et al. 2010, **Rif**, Haut Maggou (1300 m), Oued Talembote (320 m); Kettani and Langton 2012

﻿***Paraphaenocladius* Thienemann, 1924**

﻿*Paraphaenocladius ﻿﻿﻿exagitans* ssp. 1

Kettani and Moubayed-Breil 2018, **Rif**

﻿*Paraphaenocladiusimpensus impensus* (Walker, 1856)

Kettani and Moubayed-Breil 2018, **Rif**

﻿*Paraphaenocladius ﻿﻿﻿irritus* Walker, 1856

Kettani and Moubayed-Breil 2018, **Rif**

﻿*Paraphaenocladius ﻿﻿﻿pseudirritus* Strenzke, 1950

Kettani and Moubayed-Breil 2018, **Rif**

﻿***Paratrissocladius* Zavřel, 1937**

﻿*Paratrissocladius ﻿﻿﻿excerptus* (Walker, 1856)

Kettani et al. 1996, **Rif**, Ras el Ma (Chefchaouen); Kettani et al. 1997, **Rif**, Ras el Ma (Chefchaouen); Kettani et al. 2001; Kettani et al. 2010, **Rif**, Oued Kelaâ (Akoumi, 400 m); Kettani and Langton 2012; Ashe and O’Connor 2012; Kettani and Moubayed-Breil 2018, **Rif**

﻿***Parorthocladius* Thienemann, 1935**

﻿*Parorthocladius ﻿﻿﻿nudipennis* (Kieffer in Kieffer & Thienemann 1908)

Azzouzi et al. 1992, **HA**, Oued Tensift; Kettani et al. 2001; Kettani and Langton 2012; Ashe and O’Connor 2012; Kettani and Moubayed-Breil 2018, **Rif**

***Psecrocladius* Kieffer, 1906**

﻿*Psectrocladius (Allopsectrocladius) ﻿obvius* (Walker, 1856)

= ﻿*Psectrocladius ﻿﻿﻿dilatatus* (van der Wulp, 1859), in Naya 1988: 48

Naya 1988, **MA**, Moyen Sebou; Azzouzi et al. 1992, **HA**, Oued Tensift; Kettani et al. 2001; Dakki et al. 2008, **MA**, Oued Sebou; Kettani and Langton 2011, **Rif**, sources de Issaguen; Kettani and Langton 2012; Ashe and O’Connor 2012; Kettani and Moubayed-Breil 2018, **Rif**

﻿*Psectrocladius (Allopsectrocladius) ﻿platypus* (Edwards, 1929)

Kettani and Moubayed-Breil 2018, **Rif**

﻿*Psectrocladius (Mesopsectrocladius) ﻿barbatipes* Kieffer, 1923

Kettani et al. 1994, **Rif**, aval Oued Talembote (usine éléctrique); Kettani et al. 1995, **Rif**, Oued Mhajrat; Kettani et al. 1996; Kettani et al. 1997, **Rif**, Maggou (Oued Laou); Kettani et al. 2001; Kettani et al. 2010, **Rif**, Haut Maggou (1300 m), Oued Laou (Afertane, 56 m); Dakki 1997; Kettani and Langton 2012; Ashe and O’Connor 2012

*Psecrocladius (Psectrocladius) ﻿brehmi* Kieffer, 1923

Kettani et al. 1995, **Rif**, Oued Mhajrat; Kettani et al. 1996; Kettani et al. 2001; Kettani and Langton 2012; Ashe and O’Connor 2012

﻿*Psectrocladius (Psectrocladius) ﻿fennicus* Storå, 1939

Kettani and Langton 2012

﻿*Psectrocladius (Psectrocladius) ﻿limbatellus* (Holmgren, 1869)

Wülker 1959; Azzouzi and Laville 1987, **HA**, Lac Tamhda (2800 m); Kettani et al. 2001; Kettani and Langton 2011, **AP**, marais de Loukous; Kettani and Langton 2012; Ashe and O’Connor 2012; Kettani and Moubayed-Breil 2018, **Rif**

﻿*Psectrocladius (Psectrocladius*) *octomoculatus* Wülker, 1956

Kettani et al. 1995, **Rif**, Oued Mhajrat; Kettani et al. 1996; Kettani et al. 2001; Kettani and Langton 2012; Ashe and O’Connor 2012

﻿*Psectrocladius (Psectrocladius) ﻿sordidellus* (Zetterstedt, 1838)

Azzouzi and Laville 1987, **MA**, Lac Aguelmane Azigza; Kettani et al. 2001; Kettani and Langton 2011, **AP**, marais de Loukous (NE Boucharene); Kettani and Langton 2012; Ashe and O’Connor 2012; Kettani and Moubayed-Breil 2018, **Rif**

﻿*Psectrocladius (Psectrocladius) ﻿ventricosus* Kieffer, 1925

Azzouzi et al. 1992, **HA**, Oued Tensift; Kettani et al. 2001; Kettani and Langton 2012; Ashe and O’Connor 2012

﻿***Pseudosmittia* Edwards, 1932**

﻿*Pseudosmittia ﻿﻿﻿albipennis* (Goetghebuer, 1921)

Kettani and Moubayed-Breil 2018, **Rif**

﻿*Pseudosmittia ﻿﻿﻿baueri* Strenzke, 1960

Kettani and Moubayed-Breil 2018, **Rif**

﻿*Pseudosmittia ﻿﻿﻿danconai* (Marcuzzi, 1947)

Kettani and Moubayed-Breil 2018, **Rif**

﻿*Pseudosmittia ﻿﻿﻿holsata* Thienemann & Stenzke, 1940

Kettani and Moubayed-Breil 2018, **Rif**

﻿*Pseudosmittia ﻿﻿﻿obtusa* Strenzke, 1960

Kettani and Moubayed-Breil 2018, **Rif**

﻿*Pseudosmittia ﻿﻿﻿trilobata* Edwards, 1929

Kettani and Moubayed-Breil 2018, **Rif**

﻿***Pseudorthocladius* Goetghebuer, 1943**

﻿*Pseudorthocladius (Pseudorthocladius) ﻿berthelemyi* Moubayed, 1990

Azzouzi et al. 1992, **HA**, Oued Tensift; Kettani et al. 2001; Laville and Langton 2002; Kettani and Langton 2012; Ashe and O’Connor 2012; Moubayed-Breil and Kettani 2019, **Rif**, Chrafate, Challal Sghir (Akchour)

﻿*Pseudorthocladius (Pseudorthocladius) ﻿curtistylus* (Goetghebuer, 1921)

Azzouzi et al. 1992, **HA**, Oasis Meski (1160 m); Kettani et al. 2001; Kettani and Langton 2012; Ashe and O’Connor 2012

﻿*Pseudorthocladius* near ﻿Pe 3 Langton 1991

Kettani and Langton 2011, **Rif**, Bouztate (Fifi); Kettani and Langton 2012

﻿***Rheocricotopus* Brundin, 1956**

﻿*Rheocricotopus (Psilocricotopus) ﻿atripes* (Kieffer, 1913)

= ﻿*Rheocricotopus (Psilocricotopus) ﻿foveatus*﻿*foveatus* (Edwards, 1929), in Naya 1988: 40

Naya 1988, **MA**, Haut Sebou (Haut Guigou); Azzouzi et al. 1992, **HA**, Oued Tensift, Gorges de Dadès (Imdiazen, 1900 m); Kettani et al. 1994, **Rif**, Haut Laou, Oued Moulay Bouchta, aval Barrage Talembote, aval Oued Talembote (usine éléctrique), Oued Afertane; Kettani et al. 1995, **Rif**, aval Oued El Kbir, aval Oued Krikra, Oued El Kbir, amont Oued Nakhla, Oued Mhajrat; Kettani et al. 1996; Kettani et al. 1997, **Rif**, Maggou (Oued Laou), Oued Khizana (Oued Laou), Ras el Ma (Chefchaouen); Dakki 1997; Kettani et al. 2001; Kettani and El Ouazzani 2005, **Rif**, amont Oued Nakhla; Kettani et al. 2010, **Rif**, Oued Talembote, Oued Tassikeste (Afechtal, 240 m), Oued Laou (Afertane, 56 m); Dakki et al. 2008, **MA**, Oued Sebou; Kettani and Langton 2012; Ashe and O’Connor 2012; Kettani and Moubayed-Breil 2018, **Rif**

﻿Rheocricotopus (Psilocricotopus) ﻿chalybeatus subsp. chalybeatus (Edwards, 1929)

Azzouzi and Laville 1987, **MA**, Oued Boufekrane, Oued Sebou, Oued Oum-er-Rbia; Kettani et al. 1994, **Rif**, Haut Laou, Oued Siflaou, Oued Moulay Bouchta, aval Barrage Talembote, Oued Afertane, aval Oued Tassikeste, aval Oued Laou; Kettani et al. 1995, **Rif**, aval Oued El Kbir, aval Oued Krikra, Oued El Kbir, amont Oued Nakhla, Oued Mhajrat, aval Oued Khemis, Oued Martil (Tamuda); Kettani et al. 1996; Dakki 1997; Kettani et al. 1997, **Rif**, Maggou (Oued Laou), Oued Khizana (Oued Laou), Ras el Ma (Chefchaouen); Kettani et al. 2001; Kettani and El Ouazzani 2005, **Rif**, amont Oued Nakhla; Kettani et al. 2010, **Rif**, Oued Tamaridine (Zaouiet et Habtiyiène, 819 m); Dakki et al. 2008, **MA**, Oued Sebou; Kettani and Langton 2012; Ashe and O’Connor 2012; Kettani and Moubayed-Breil 2018, **Rif**

﻿*Rheocricotopus (Psilocricotopus) ﻿gallicus* Lehamnn 1969

Kettani and Moubayed-Breil 2018, **Rif**

﻿*Rheocricotopus (Psilocricotopus) ﻿glabricollis* (Meigen, 1830)

Azzouzi and Laville 1987, **MA**, Oued Boufekrane; Kettani et al. 2001; Kettani et al. 2010, **Rif**, Oued Ametrasse (Ametrasse, 820 m), Kettani and Langton 2012; Ashe and O’Connor 2012; Kettani and Moubayed-Breil 2018, **Rif**

﻿*Rheocricotopus (Psilocricotopus) meridionalis* Moubayed-Breil, 2016

Kettani and Moubayed-Breil 2018, **Rif**

﻿*Rheocricotopus (Psilocricotopus) ﻿tirolus* Lehmann, 1969

Kettani et al. 1994, **Rif**, Haut Laou, Oued Siflaou; Kettani et al. 1995, **Rif**, aval Oued El Kbir, aval Oued Krikra, Oued El Kbir, Oued Mhajrat; Kettani et al. 1996; Kettani et al. 1997, **Rif**, Oued Khizana (Oued Laou); Kettani et al. 2001; Kettani and El Ouazzani 2005, **Rif**, amont Oued Nakhla; Azzouzi et al. 1992, **HA**, Oued Tensift; Dakki 1997; Kettani and Langton 2012; Ashe and O’Connor 2012; Kettani and Moubayed-Breil 2018, **Rif**

﻿*Rheocricotopus (Rheocricotopus) ﻿effusus* (Walker, 1856)

Reiss 1977; Naya 1988, **MA**, Haut et Moyen Sebou; Fekhaoui et al. 1993; Kettani et al. 2001; Dakki et al. 2008, **MA**, Oued Sebou; Kettani et al. 2010, **Rif**, Oued Tamaridine (Zaouiet et Habtiyiène, 819 m); Kettani and Langton 2012; Ashe and O’Connor 2012; Kettani and Moubayed-Breil 2018, **Rif**

﻿*Rheocricotopus (Rheocricotopus) ﻿fuscipes* (Kieffer, 1909)

Azzouzi et al. 1992, **HA**, Oued Tensift; Kettani et al. 2001; Kettani et al. 2010, **Rif**, Maggou (905 m); Kettani and Langton 2012; Ashe and O’Connor 2012; Kettani and Moubayed-Breil 2018, **Rif**

﻿*Rheocricotopus (Rheocricotopus) rifensis* Moubayed & Kettani, 2019

Moubayed-Breil and Kettani, **Rif**, Chrafate, Challal Sghir (Akchour)

﻿***Synorthocladius* Thienemann, 1935**

﻿*Synorthocladius ﻿﻿﻿semivirens* (Kieffer, 1909)

Kettani et al. 1996; Kettani et al. 1997, **Rif**, Oued Khizana (Oued Laou), Ras el Ma (Chefchaouen); Kettani et al. 2001; Azzouzi et al. 1992, **HA**, Oued Tensift; Kettani and Langton 2012; Ashe and O’Connor 2012; Kettani and Moubayed-Breil 2018, **Rif**

﻿***Smittia* Holmgren, 1869**

﻿*Smittia ﻿﻿﻿﻿alpicola* Goetghebuer, 1941

Kettani and Moubayed-Breil 2018, **Rif**

﻿*Smittia ﻿﻿﻿aterrima* Meigen, 1818

Kettani and Moubayed-Breil 2018, **Rif**

﻿*Smittia ﻿﻿﻿contingens* Walker, 1856

Kettani and Moubayed-Breil 2018, **Rif**

﻿*Smittia ﻿﻿﻿foliacea* (Kieffer, 1921)

Kettani and Moubayed-Breil 2018, **Rif**

﻿*Smittia ﻿﻿﻿pratorum* Goetghebuer, 1927

Kettani and Moubayed-Breil 2018, **Rif**

﻿***Thienemannia* Kieffer, 1909**

﻿*Thienemannia* ﻿﻿cf. ﻿*fulvofasciata* (Kieffer, 1921)

Kettani and Moubayed-Breil 2018, **Rif**

﻿*Thienemannia ﻿﻿﻿gracilis* Kieffer, 1909

Kettani and Moubayed-Breil 2018, **Rif**

﻿***Thienemanniella* Kieffer, 1911**

﻿*Thienemanniella ﻿﻿﻿﻿﻿﻿acuticornis* (Kieffer, 1912)

Fittkau and Reiss 1978; Azzouzi and Laville 1987, **MA**, Oued Oum-er-Rbia; Kettani et al. 2001; Kettani et al. 2010, **Rif**, Oued Kelaâ (Akoumi, 400 m), Oued Talembote (320 m); Kettani and Langton 2011, **Rif**, Oued Hamma, Oued Ketama, Oued Sgara; Kettani and Langton 2012; Ashe and O’Connor 2012

﻿*Thienemanniella ﻿﻿﻿clavicornis* (Kieffer, 1911)

Kettani and Moubayed-Breil 2018, **Rif**

﻿*Thienemanniella ﻿﻿﻿majuscula* (Edwards, 1924)

Kettani et al. 1995, **Rif**, aval Oued El Kbir; Kettani et al. 1996; Kettani et al. 2001; Kettani and Langton 2012; Ashe and O’Connor 2012; Kettani and Moubayed-Breil 2018, **Rif**

﻿*Thienemanniella ﻿﻿﻿vittata* (Edwards, 1924)

Kettani et al. 1996, **Rif**, Haut Maggou; Kettani et al. 1997, **Rif**, Maggou (Oued Laou); Kettani et al. 2001; Kettani et al. 2010, **Rif**, Haut Maggou (1300 m); Kettani and Langton 2012; Ashe and O’Connor 2012; Kettani and Moubayed-Breil 2018, **Rif**

﻿*Thienemanniella* ﻿Pe 2a Langton 1991

Kettani et al. 2010, **Rif**, Oued Maggou (905 m), Oued Kelaâ (Akoumi, 400 m), Oued Talembote; Kettani and Langton 2012

﻿*Thienemanniella* ﻿Pe 2b Langton 1991

Kettani et al. 2010, **Rif**, Oued Maggou (905 m), Oued Talembote; Kettani and Langton 2012

﻿***Trissocladius* Kieffer, 1908**

﻿*Trissocladius ﻿﻿﻿brevipalpis* Kieffer in Kieffer & Thienemann 1908

Azzouzi et al. 1992, **HA**, Oued Tensift; Kettani et al. 2001; Kettani and Langton 2012; Ashe and O’Connor 2012

﻿***Tvetenia* Kieffer, 1922**

﻿*Tvetenia ﻿﻿﻿bavarica* (Goetghebuer, 1934)

Azzouzi and Laville 1987, **MA**, Oued Oum-er-Rbia; Kettani et al. 2001; Kettani and Langton 2012; Ashe and O’Connor 2012

﻿*Tvetenia ﻿﻿﻿calvescens* (Edwards, 1929)

Naya 1988, **MA**, Moyen Sebou; Azzouzi et al. 1992, **HA**, Oued Tensift; Kettani et al. 1994, **Rif**, Haut Laou, Oued Siflaou, Oued Moulay Bouchta, aval Oued Talembote (usine éléctrique), Oued Afertane, aval Oued Tassikeste; Kettani et al. 1995, **Rif**, aval Oued El Kbir, aval Oued Krikra, Oued El Kbir, amont Oued Nakhla, Oued Mhajrat; Kettani et al. 1996; Dakki 1997; Kettani et al. 1997, **Rif**, Maggou (Oued Laou), Ras el Ma (Chefchaouen); Kettani et al. 2001; Kettani and El Ouazzani 2005, **Rif**, amont Oued Nakhla; Kettani et al. 2010, **Rif**, Oued Tamaridine (Zaouiet et Habtiyiène, 819 m), Oued Talembote (245 m), Oued Laou (Afertane, 56 m); Dakki et al. 2008, **MA**, Oued Sebou; Kettani and Langton 2012; Ashe and O’Connor 2012; Kettani and Moubayed-Breil 2018, **Rif**

﻿*Tvetenia ﻿﻿﻿discoloripes* (Goetghebuer & Thienemann in Thienemann, 1936)

Kettani and Langton 2011, **Rif**, Oued Nakhla, Bouztate (Fifi); Kettani and Langton 2012; Ashe and O’Connor 2012

﻿*Tvetenia ﻿﻿﻿verralli* (Edwards, 1929)

Azzouzi et al. 1992, **HA**, Oued Tensift; Kettani et al. 1994, **Rif**, Haut Laou, Oued Siflaou, Oued Moulay Bouchta; Kettani et al. 1995, **Rif**, aval Oued El Kbir, amont Oued Nakhla; Kettani et al. 1996; Dakki 1997; Kettani et al. 1997, **Rif**, Maggou (Oued Laou), Oued Khizana (Oued Laou), Ras el Ma (Chefchaouen); Kettani et al. 2001; Kettani and El Ouazzani 2005, **Rif**, amont Oued Nakhla; Kettani et al. 2010, **Rif**, ruisselet maison forestière Talassemtane (1683 m), Oued Tamaridine (Zaouiet et Habtiyiène, 819 m), Oued Talembote (245 m), Oued Laou (Afertane, 56 m); Kettani and Langton 2012; Ashe and O’Connor 2012; Kettani and Moubayed-Breil 2018, **Rif**

﻿***Zalutschia* Lipina, 1939**

﻿*Zalutschia ﻿﻿﻿humphriesiae* Dowling & Murray, 1980

Kettani and Langton 2011, **Rif**, marais de Lemtahane (PNPB), Dayat Fifi; Kettani and Langton 2012; Ashe and O’Connor 2012

##### Chironominae

Chironomini

﻿***Chironomus* Meigen, 1803**

﻿*Chironomus (Baeotendipes) ﻿noctivagus* (Kieffer, 1911)

Kettani et al. 2001; Kettani and Langton 2012; Kettani and Moubayed-Breil 2018, **Rif**

﻿*Chironomus (Chironomus) ﻿annularius* Meigen, 1818

Azzouzi and Laville 1987, **HA**, Oued Tensift; Kettani et al. 2001; Kettani and Langton 2012; Kettani and Moubayed-Breil 2018, **Rif**

﻿*Chironomus (Chironomus) ﻿aprilinus* sensu Meigen, 1818

= ﻿*Chironomushalophilus* Kieffer, in Ramdani and Tourenq 1982: 180, in Naya 1988: 50

Ramdani and Tourenq 1982, **AP**, Merja Sidi Boughaba; Azzouzi and Laville 1987; Naya 1988, **MA**, Haut Sebou; Fekhaoui et al. 1993; Kettani et al. 2001; Dakki et al. 2008, **MA**, Oued Sebou; Kettani and Langton 2012; Kettani and Moubayed-Breil 2018, **Rif**

﻿*Chironomus (Chironomus) ﻿bernensis* Klötzli, 1973

= ﻿*Chironomus* sp 1 Kettani 1994

Kettani et al. 1994; Kettani et al. 1996; Kettani et al. 2001; Dakki 1997; Kettani and Langton 2012; Kettani and Moubayed-Breil 2018, **Rif**

﻿*Chironomus (Chironomus) ﻿calipterus* Kieffer, 1908

Reiss 1977, **AP**, Larache; Fittkau and Reiss 1978; Ramdani and Tourenq 1982**AP**, Merja Sidi Boughaba; Azzouzi and Laville 1987; Kettani et al. 2001; Kettani and Langton 2012; Kettani and Moubayed-Breil 2018, **Rif**

﻿*Chironomus (Chironomus) ﻿longistylus* Goetghebuer, 1921

Kettani et al. 2011, **Rif**, Oued Ketama; Kettani and Langton 2012

﻿*Chironomus (Chironomus) ﻿luridus* Strenzke, 1959

Ramdani and Tourenq 1982, **AP**, Merja Sidi Boughaba; Azzouzi and Laville 1987; Kettani et al. 2001; Kettani and Langton 2011, **Rif**, merja Mtalssi (Tamuda, 31 m); Kettani and Langton 2012; Kettani and Moubayed-Breil 2018, **Rif**

﻿*Chironomus (Chironomus) ﻿nuditarsis* Keyl, 1961

Kettani et al. 2011, **Rif**, Oued Boujdad (Kitane, 42 m), Oued El Hatba (SIBE Jebel Moussa, 165 m); Kettani and Langton 2012, **Rif**, SIBE Jebel Moussa

﻿*Chironomus (Chironomus) ﻿piger* (Strenzke, 1956)

Ramdani and Tourenq 1982, **AP**, Merja Sidi Boughaba; Azzouzi and Laville 1987; Kettani et al. 2001; Kettani and Langton 2012; Kettani and Moubayed-Breil 2018, **Rif**

﻿*Chironomus (Chironomus) ﻿plumosus* (Linnaeus, 1758)

Reiss 1977, **AP**, Larache, **AA**, Dra-Tal; Ramdani and Tourenq 1982, **AP**, Merja Sidi Boughaba; El Mezdi and Giudicelli 1985, **HA**, Khettaras de Marrakech; Azzouzi and Laville 1987; Naya 1988, **MA**, Sidi Abdellah, Dar Cheih Harazem, Dar El Arsa; Fekhaoui et al. 1993; Kettani et al. 1994, **Rif**, Haut Laou, Oued Siflaou; Kettani et al. 1996; Dakki 1997; Kettani et al. 2001; Kettani et al. 2010, **Rif**, Aïn Tissmelal (Tissmelal, 1046 m); Dakki et al. 2008, **MA**, Oued Sebou; Kettani and Langton 2012; Kettani and Moubayed-Breil 2018, **Rif**

﻿*Chironomus (Chironomus) ﻿prasinus* sensu Pinder, 1978

Kettani et al. 2011, **Rif**, merja Mtalssi (Tamuda, 31 m); Kettani and Langton 2012

﻿*Chironomus (Chironomus) ﻿riparius* Meigen, 1804

= ﻿*Chironomus ﻿﻿﻿thummi* Kieffer, in Naya 1988: 51, Fekhaoui et al. 1993: 26

Ramdani and Tourenq 1982, **AP**, Merja Sidi Boughaba; El Mezdi and Giudicelli 1985, **HA**, Khettaras de Marrakech; Azzouzi and Laville 1987, **MA**, Oued Boufekrane, Oued Fès, Oued Sebou; Naya 1988, **MA**, Moyen Sebou; Kettani et al. 1994, **Rif**, Haut Laou, Oued Siflaou, aval Barrage Talembote; Kettani et al. 1996; Dakki 1997; Kettani et al. 1997, **Rif**, Ras el Ma (Chefchaouen); Kettani et al. 2001; Kettani and El Ouazzani 2005, **Rif**, Oued Nakhla; Kettani et al. 2010, **Rif**, Oued Talembote (aval Barrage Talembote, 245 m); Dakki et al. 2008, **MA**, Oued Sebou; Kettani and Langton 2012; Kettani and Moubayed-Breil 2018, **Rif**

﻿*Chironomus (Chironomus) ﻿salinarius* Kieffer, 1915

Ramdani and Tourenq 1982, **AP**, Merja Sidi Boughaba; Azzouzi and Laville 1987; Kettani et al. 2001; Kettani and Langton 2011, **Rif**, merja Mtalssi (Tamuda, 31 m); Kettani and Langton 2012; Kettani and Moubayed-Breil 2018, **Rif**

﻿*Chironomus (Chironomus) ﻿tentans* Fabricius, 1805

= *Camptochironomus﻿tentans* Fabricius, 1805, in Naya 1988: 50

Naya 1988, **MA**, Moyen Sebou; Kettani et al. 2001; Kettani and Langton 2012

﻿***Cladopelma* Kieffer, 1921**

﻿*Cladopelma ﻿﻿﻿virescens* (Meigen, 1818)

Kettani and Moubayed-Breil 2018, **Rif**

﻿***Cryptochironomus* Kieffer, 1918**

﻿*Cryptochironomus (Cryptochironomus) ﻿albofasciatus* (Staeger, 1839)

= ﻿*Cryptochironomusobreptans* Walker, 1856, in Kettani 1994: 28

Kettani et al. 1994, **Rif**, Oued Siflaou; Kettani et al. 1996; Kettani et al. 2001; Dakki 1997; Kettani and Langton 2012

﻿*Cryptochironomus (Cryptochironomus) ﻿psittacinus* (Meigen, 1830)

Kettani et al. 1996, **Rif**, Oued Nakhla; Kettani et al. 2001; Kettani and El Ouazzani 2005, **Rif**, Oued Nakhla; Kettani and Langton 2012

﻿*Cryptochironomus (Cryptochironomus) ﻿rostratus* Kieffer, 1921

El Mezdi and Giudicelli 1985, **HA**, Khettaras de Marrakech; Azzouzi and Laville 1987, **MA**, Oued Fès, Oued Sebou, oued Oum-er-Rbia, Oued Boufekrane, **HA**, Oued Tensift; Kettani et al. 1994, **Rif**, Haut Laou, aval Oued Laou; Kettani et al. 1995, **Rif**, aval Oued Khemis; Kettani et al. 1996; Dakki 1997; Kettani et al. 2001; Kettani and El Ouazzani 2005, **Rif**, Oued Nakhla; Dakki et al. 2008, **MA**, Oued Sebou; Kettani and Langton 2012; Kettani and Moubayed-Breil 2018, **Rif**

﻿*Cryptochironomus (Cryptochironomus) ﻿supplicans* (Meigen, 1830)

Kettani and Moubayed-Breil 2018, **Rif**

﻿*Cryptochironomus* ﻿Pe 5 Langton 1991

Kettani et al. 1994, **Rif**, Haut Laou, Oued Siflaou; Kettani et al. 1996; Dakki 1997; Kettani et al. 2001; Kettani and Langton 2012

﻿***Demicryptochironomus* Lenz, 1941**

﻿*Demicryptochironomus (Demicryptochironomus) ﻿vulneratus* (Zetterstedt, 1838)

Kettani et al. 1994, **Rif**, Haut Laou; Kettani et al. 1996; Dakki 1997; Kettani et al. 2001; Kettani et al. 2010, **Rif**, Nord Maggou village (Maggou, 905 m); Kettani and Langton 2012

﻿*Demicryptochironomus (Irmakia) ﻿neglectus* Reiss, 1988

Kettani and Moubayed-Breil 2018, **Rif**

﻿*Demicryptochironomus (Irmakia*) ﻿Pe 1 Langton 1991

Kettani et al. 1995, **Rif**, aval Oued El Kbir, Oued El Kbir, aval Oued Khemis; Kettani et al. 2001; Kettani and Langton 2012

﻿***Dicrotendipes* Kieffer, 1913**

﻿*Dicrotendipes ﻿﻿﻿collarti* (Goetghebuer, 1936)

El Mezdi and Giudicelli 1985, **HA**, Khettaras de Marrakech; Kettani et al. 2001; Kettani and Langton 2012

﻿*Dicrotendipes ﻿﻿﻿cordatus* Kieffer, 1922

Kettani et al. 1996, **Rif**, Oued Khizana (Oued Laou); Kettani et al. 1997, **Rif**, Oued Khizana (Oued Laou); Kettani et al. 2001; Kettani and Langton 2012

﻿*Dicrotendipes ﻿﻿﻿fusconotatus* (Kieffer, 1922)

Azzouzi et al. 1992, **HA**, Oued Tensift; Kettani et al. 1994, **Rif**, Haut Laou, Oued Siflaou, aval Barrage Talembote; Kettani et al. 1996; Dakki 1997; Kettani et al. 2001; Kettani et al. 2010, **Rif**, Oued Talembote (aval Barrage Talembote, 245 m); Kettani and Langton 2012; Kettani and Moubayed-Breil 2018, **Rif**

﻿*Dicrotendipes ﻿﻿﻿modestus* (Say, 1823)

Kettani et al. 2011, **Rif**, merja Mtalssi (Tamuda, 31 m); Kettani and Langton 2012

﻿*Dicrotendipes ﻿﻿﻿nervosus* (Staeger, 1839)

= *Limnochirononomus ﻿﻿﻿nervosus* Staeger, in Naya 1988: 53

Naya 1988, **MA**, Moyen Sebou (Sidi Abdellah); Kettani et al. 1995, **Rif**, aval Oued El Kbir, aval Oued Krikra, aval Oued Khemis; Kettani et al. 1996; Kettani et al. 2001; Dakki et al. 2008, **MA**, Oued Sebou; Kettani and Langton 2012; Kettani and Moubayed-Breil 2018, **Rif**

﻿*Dicrotendipes ﻿﻿﻿notatus* (Meigen, 1818)

Kettani and Moubayed-Breil 2018, **Rif**

﻿*Dicrotendipes ﻿﻿﻿pallidicornis* (Goetghebuer, 1934)

Azzouzi and Laville 1987, **Rif**, Retenue El Makhazine, **MA**, Oued Boufekrane; Kettani et al. 2001; Kettani and Langton 2012; Kettani and Moubayed-Breil 2018, **Rif**

﻿*Dicrotendipes ﻿﻿﻿peringueyanus* Kieffer, 1924

Ramdani and Tourenq 1982, **AP**, Merja Sidi Boughaba; Kettani et al. 1994, **Rif**, Haut Laou, Oued Siflaou, aval Barrage Talembote; Kettani et al. 1995, **Rif**, aval Oued Krikra, aval Oued Khemis; Kettani et al. 1996; Dakki 1997; Kettani et al. 2001; Kettani et al. 2010, **Rif**, Oued Talembote (aval Barrage Talembote, 245 m); Kettani and Langton 2012

﻿*Dicrotendipes ﻿﻿﻿septemmaculatus* (Becker, 1908)

= ﻿*Dicrotendipespilosimanus* Kieffer, in Reiss 1977: 91, Azzouzi and Laville 1987: 219

Reiss 1977, **AP**, Larache; Fittkau and Reiss 1978; Azzouzi and Laville 1987, **AP**, Larache; Kettani et al. 1994, **Rif**, Haut Laou, Oued Siflaou, aval Barrage Talembote, aval Oued Laou; Kettani et al. 1995, **Rif**, aval Oued Krikra, amont Oued Nakhla, aval Oued Khemis, Oued Martil (Tamuda); Kettani et al. 1996; Dakki 1997; Kettani et al. 1997, **Rif**, Oued Khizana (Oued Laou); Kettani et al. 2001; Kettani et al. 2010, **Rif**, Oued Talembote (aval Barrage Talembote, 245 m); Kettani and Langton 2012

﻿***Endochironomus* Kieffer, 1918**

﻿*Endochironomus ﻿﻿﻿albipennis* (Meigen, 1830)

Naya 1988, **MA**, Haut Sebou (Skhounata); Kettani et al. 2001; Dakki et al. 2008, **MA**, Oued Sebou; Kettani and Langton 2012

﻿*Endochironomus ﻿﻿﻿tendens* (Fabricius, 1775)

Naya 1988, **MA**, Moyen Sebou (Gantra Mdez, Azzaba); Kettani et al. 2001; Dakki et al. 2008, **MA**, Oued Sebou; Kettani and Langton 2012

﻿***Glyptotendipes* Kieffer, 1913**

﻿*Glyptotendipes (Caulochironomus) ﻿viridis* (Macquart, 1834)

Naya 1988, **MA**, Moyen Sebou; Kettani et al. 2001; Dakki et al. 2008, **MA**, Oued Sebou; Kettani and Langton 2012

﻿*Glyptotendipes (Glyptotendipes) ﻿cauliginellus* (Kieffer, 1913)

= ﻿*Glyptotendipesgripekoveni* (Kieffer)

Naya 1988, **MA**, Haut Sebout (Guigou); Kettani et al. 2001; Dakki et al. 2008, **MA**, Oued Sebou; Kettani and Langton 2012

﻿*Glyptotendipes (Glyptotendipes) ﻿pallens* (Meigen, 1804)

Azzouzi and Laville 1987, **Rif**, Retenue El Makhazine; Naya 1988, **MA**, Moyen Sebou; Kettani et al. 2001; Dakki et al. 2008, **MA**, Oued Sebou; Kettani and Langton 2012

﻿*Glyptotendipes* ﻿sp A Langton 1991

Naya 1988, **MA**, Oued Sebou; Kettani et al. 2001; Kettani and Langton 2012

﻿*Glyptotendipes* ﻿sp B Langton 1991

Naya 1988, **MA**, Oued Sebou; Kettani et al. 2001; Kettani and Langton 2012

﻿***Harnischia* Kieffer, 1921**

﻿*Harnischia ﻿﻿﻿curtilamellata* (Malloch, 1915)

Azzouzi and Laville 1987, **MA**, Oued Fès, Oued Sebou; Kettani et al. 1994, **Rif**, Oued Siflaou, Oued Afertane, aval Oued Laou; Kettani et al. 1995, **Rif**, amont Oued Nakhla, aval Oued Khemis, Oued Martil (Tamuda); Kettani et al. 1996; Dakki 1997; Kettani et al. 2001; Kettani et al. 2010, **Rif**, Source Maggou (Maggou, 1300 m); Kettani and Langton 2012; Kettani and Moubayed-Breil 2018, **Rif**

﻿*Harnischia ﻿﻿﻿fuscimanus* Kieffer, 1921

Azzouzi and Laville 1987, **Rif**, Retenue El Makhazine, **MA**, Oued Boufekrane; Kettani et al. 1995, **Rif**, Oued El Kbir, amont Oued Nakhla, aval Oued Khemis; Kettani et al. 1996; Kettani et al. 1997, **Rif**, Maggou (Oued Laou), Oued Khizana (Oued Laou); Kettani et al. 2001; Kettani et al. 2010, **Rif**, Oued Laou (Afertane, 55 m); Kettani and Langton 2012; Kettani and Moubayed-Breil 2018, **Rif**

﻿***Kiefferulus* Goetghebuer, 1922**

﻿*Kiefferulus (Kiefferulus) ﻿tendipediformis* (Goetghebuer, 1921)

Reiss 1977, **Rif**, Tétouan; Fittkau and Reiss 1978; Ramdani and Tourenq 1982, **AP**, Merja Sidi Boughaba; Azzouzi and Laville 1987; Ashe and Cranston 1990; Kettani et al. 2010, **Rif**, Guelta 1 km après Amariguen (Jebel Setsou, 1280 m); Kettani et al. 2001; Kettani and Langton 2011, **Rif**, Dayat Dalia (SIBE Jebel Moussa, 169 m); Kettani and Langton 2012; Kettani and Moubayed-Breil 2018, **Rif**

﻿***Kloosia* Kruseman, 1933**

﻿*Kloosia ﻿﻿﻿pusilla* (Linnaeus, 1767)

Azzouzi et al. 1992, **HA**, Oued Tensift; Kettani et al. 2001; Kettani and Langton 2012

﻿***Lauterborniella* Thienemann & Bause, 1913**

﻿*Lauterborniella ﻿﻿﻿agrayloides* (Kieffer, 1911)

Naya 1988, **MA**, Haut Sebou; Kettani et al. 2001; Kettani and Langton 2012

﻿***Microchironomus* Kieffer, 1918**

﻿*Microchironomus ﻿﻿﻿deribae* (Freeman, 1957)

= *Leptochirononomus ﻿﻿﻿deribae* Freeman, in Reiss 1977: 91, Ramdani and Tourenq 1982: 180

Reiss 1977, **AP**, Rabat; Ramdani and Tourenq 1982, **AP**, Merja Sidi Boughaba; Azzouzi and Laville 1987; Ashe and Cranston 1990; Kettani et al. 2001; Kettani and Langton 2012

﻿*Microchironomus ﻿﻿﻿lendli* (Kieffer, 1918)

Reiss 1986, **AA**, Oasis Meski; Azzouzi and Laville 1987; Kettani et al. 2001; Kettani and Langton 2012

﻿*Microchironomus ﻿﻿﻿tener* (Kieffer, 1918)

Kettani et al. 1994, **Rif**, Oued Siflaou; Kettani et al. 1996; Dakki 1997; Kettani et al. 1997, **Rif**, Oued Khizana (Oued Laou); Kettani et al. 2001; Azzouzi et al. 1992, **HA**, Oued Tensift, Barrage Lalla Takerkoust; Kettani and Langton 2012

﻿***Microtendipes* Kieffer, 1915**

﻿*Microtendipes ﻿﻿﻿britteni* (Edwards, 1929)

Kettani et al. 1994, **Rif**, Haut Laou, Oued Siflaou, Oued Moulay Bouchta, aval Barrage Talembote, aval Oued Talembote (usine éléctrique), Oued Afertane, aval Oued Laou; Kettani et al. 1995, **Rif**, aval Oued El Kbir, aval Oued Krikra, Oued El Kbir, amont Oued Nakhla, Oued Mhajrat, aval Oued Khemis, Oued Martil (Tamuda); Kettani et al. 1996; Dakki 1997; Kettani et al. 1997, **Rif**, Maggou (Oued Laou), Oued Khizana (Oued Laou); Kettani et al. 2001; Kettani and El Ouazzani 2005, **Rif**, Oued Nakhla; Kettani et al. 2010, **Rif**, Oued Talembote (aval Barrage Talembote, 245 m), Oued Talembote (aval affluent Talembote, 155 m), Oued Laou (Afertane, 55 m); Kettani and Langton 2012

﻿*Microtendipes ﻿﻿﻿chloris* (Meigen, 1818)

Kettani et al. 2011, **Rif**, Dayat En-Nâsser (Khandek En-Nâsser, 1177 m), source Bab Karn (Fifi, 1216 m), Dayat Fifi (1179 m); Kettani and Langton 2012

﻿*Microtendipes ﻿﻿﻿confinis* (Meigen, 1830)

Kettani et al. 1996; Kettani et al. 1997, **Rif**, Maggou (Oued Laou), Oued Khizana (Oued Laou), Ras el Ma (Chefchaouen); Kettani et al. 2001; Kettani and El Ouazzani 2005, **Rif**, Oued Nakhla; Azzouzi et al. 1992, **HA**, Oued Tensift; Kettani and Langton 2012

﻿*Microtendipes ﻿﻿﻿diffinis* (Edwards, 1929)

Reiss 1977, **AA**, Dra-Tal; Fittkau and Reiss 1978; Azzouzi and Laville 1987; Ashe and Cranston 1990; Kettani et al. 2001; Kettani et al. 2011, **Rif**, Dayat En-Nâsser (Khandek En-Nâsser, 1177 m), Dayat Aïn Rami, source Bab Karn (Fifi, 1216 m); Kettani and Langton 2012

﻿*Microtendipes ﻿﻿﻿pedellus* (De Geer, 1776)

Reiss 1977, **Rif**, Environ de Tétouan; Fittkau and Reiss 1978; El Mezdi and Giudicelli 1985, **HA**, Khettaras de Marrakech; Azzouzi and Laville 1987, **Rif**, Tétouan, **HA**; Naya 1988, **MA**, Haut Sebou (amont Aîn Tadout, Skhounate, Arhbalou Aberchane); Ashe and Cranston 1990; Kettani et al. 2001; Dakki et al. 2008, **MA**, Oued Sebou; Kettani and Langton 2012; Kettani and Moubayed-Breil 2018, **Rif**

﻿***Nubensia* Spies, 2015**

﻿*Nubensia ﻿﻿﻿nubens* (Edwards, 1929)

= ﻿*Polypedilum ﻿﻿﻿nubens* (Edwards, 1929), in Azzouzi and Laville 1987: 219; Kettani et al. 1994: 28, 1995: 257, 1996: 137, 1997: 184, 2001: 331, 2010: 70; Dakki 1997: 65; Dakki et al. 2008: 32, Kettani and Langton 2012: 423

Azzouzi and Laville 1987, **MA**, Oued Sebou; Kettani et al. 1994, **Rif**, Haut Laou, Oued Siflaou, Oued Moulay Bouchta, aval Barrage Talembote, Oued Afertane, aval Oued Tassikeste, aval Oued Laou; Kettani et al. 1995, **Rif**, aval Oued El Kbir, aval Oued Krikra, Oued El Kbir, amont Oued Nakhla, Oued Mhajrat, aval Oued Khemis, Oued Martil (Tamuda); Kettani et al. 1996; Dakki 1997; Kettani et al. 1997, **Rif**, Oued Khizana (Oued Laou); Kettani et al. 2001; Dakki et al. 2008, **MA**, Oued Sebou; Kettani et al. 2010, **Rif**, Oued Talembote (aval Barrage Talembote, 245 m), Oued Laou (Ifansa, 105 m), Oued Laou (Afertane, 55 m); Kettani and Langton 2012; Kettani and Moubayed-Breil 2018, **Rif**

﻿***Parachironomus* Lenz, 1921**

﻿*Parachironomus ﻿﻿﻿frequens* (Johannsen, 1905)

Kettani et al. 1995, **Rif**, aval Oued Khemis; Kettani et al. 1996; Kettani et al. 2001; Kettani and Langton 2012

﻿*Parachironomus ﻿﻿﻿parilis* (Walker, 1856)

Reiss 1977, **AP**, Environ de Larache; Azzouzi and Laville 1987, **MA**, Lac Aguelmane Azigza; Ashe and Cranston 1990; Kettani et al. 1995, **Rif**, aval Oued Khemis; Kettani et al. 1996; Kettani et al. 2001; Dakki et al. 2008, **MA**, Oued Sebou; Kettani and Langton 2012

﻿***Paracladopelma* Harnisch, 1923**

﻿*Paracladopelma ﻿﻿﻿camptolabis* (Kieffer, 1913)

Kettani et al. 1994, **Rif**, Haut Laou, Oued Siflaou, aval Barrage Talembote; Kettani et al. 1995, **Rif**, aval Oued El Kbir, Oued Mhajrat, aval Oued Khemis, Oued Martil (Tamuda); Kettani et al. 1996; Dakki 1997; Kettani et al. 2001; Kettani and El Ouazzani 2005, **Rif**, Oued Nakhla; Kettani et al. 2010, **Rif**, Oued Talembote (aval Barrage Talembote, 245 m); Kettani and Langton 2012; Kettani and Moubayed-Breil 2018, **Rif**

﻿*Paracladopelma ﻿﻿﻿galaptera* (Townes, 1945)

Azzouzi et al. 1992, **HA**, Ouarzazate (1140 m), Gorges de Todra (1400 m); Kettani et al. 2001; Kettani and Langton 2012

﻿*Paracladopelma ﻿﻿﻿graminicolor* (Kieffer, 1925)

= *Cryptotendipes ﻿﻿﻿graminicolor* (Kieffer), in Azzouzi et al. 1992: 230

Azzouzi et al. 1992, **HA**, Oued Tensift; Kettani et al. 2001; Kettani and Langton 2012

﻿*Paracladopelma ﻿﻿﻿laminatum* (Kieffer, 1921)

Reiss 1977, **AA**, Dra-Tal; Fittkau and Reiss 1978; Azzouzi and Laville 1987; Ashe and Cranston 1990; Kettani et al. 2001; Kettani and Langton 2012; Kettani and Moubayed-Breil 2018, **Rif**

﻿*Paracladopelma ﻿﻿﻿mikianum* (Goetghebuer, 1937)

Kettani et al. 1996, **Rif**, Oued Nakhla; Kettani et al. 2001; Kettani and El Ouazzani 2005, **Rif**, amont Oued Nakhla; Azzouzi et al. 1992, **HA**, Oued Tensift; Kettani and Langton 2012; Kettani and Moubayed-Breil 2018, **Rif**

﻿***Paralauterborniella* Lenz, 1941**

﻿*Paralauterborniella ﻿﻿﻿nigrohalteralis* (Malloch, 1915)

Azzouzi and Laville 1987, **MA**, Oued Boufekrane, Oued Fès, Oued Sebou; Kettani et al. 2001; Kettani and Langton 2012

﻿***Paratendipes* Kieffer, 1911**

﻿*Paratendipes ﻿﻿﻿﻿albimanus* (Meigen, 1818)

Naya 1988, **MA**, Moyen Sebou (Mdez); Kettani et al. 1994, **Rif**, aval Barrage Talembote; Kettani et al. 1995, **Rif**, aval Oued Krikra, Oued Mhajrat, aval Oued Khemis, Oued Laou (Tamuda); Kettani et al. 1996; Dakki 1997; Kettani et al. 1997, **Rif**, Oued Khizana (Oued Laou), Ras el Ma (Chefchaouen); Kettani et al. 2001; Dakki et al. 2008, **MA**, Oued Sebou; Kettani et al. 2010, **Rif**, Oued Talembote (aval Barrage Talembote, 245 m); Kettani and Langton 2012; Kettani and Moubayed-Breil 2018, **Rif**

﻿*Paratendipes ﻿﻿﻿nudisquama* (Edwards, 1929)

Kettani and Moubayed-Breil 2018, **Rif**

﻿*Paratendipes ﻿﻿﻿striatus* (Kieffer, 1925)

El Mezdi and Giudicelli 1985, **HA**, Khettaras de Marrakech; Kettani et al. 2001; Kettani and Langton 2012

﻿***Phaenopsectra* Kieffer, 1921**

﻿*Phaenopsectraflavipes* (Meigen, 1818)

Kettani et al. 1994, **Rif**, Haut Laou; Kettani et al. 1995, **Rif**, aval Oued El Kbir, Oued Mhajrat; Kettani et al. 1996; Kettani et al. 2001; Kettani and Langton 2012; Kettani and Moubayed-Breil 2018, **Rif**

﻿***Polypedilum* Kieffer, 1912**

﻿*Polypedilum (Pentapedilum) ﻿ruandae* Freeman, 1955

El Mezdi and Giudicelli 1985, **HA**, Khettaras de Marrakech; Kettani et al. 2001; Kettani and Langton 2012

﻿*Polypedilum (Pentapedilum) ﻿sordens* (van der Wulp, 1875)

= ﻿*Polypedilum* sp 1, in Kettani et al. 1994: 28

Kettani et al. 1994, **Rif**, Oued Siflaou; Kettani et al. 1996; Dakki 1997; Kettani et al. 1997, **Rif**, Ras el Ma (Chefchaouen); Kettani et al. 2001; Kettani and El Ouazzani 2005, **Rif**, Oued Nakhla; Kettani and Langton 2012; Kettani and Moubayed-Breil 2018, **Rif**

﻿*Polypedilum (Pentapedilum) ﻿uncinatum* (Goetghebuer, 1921)

Azzouzi and Laville 1987, **MA**, Oued Boufekrane, Oued Fès; Kettani et al. 2001; Kettani and Langton 2012

﻿*Polypedilum (Polypedilum) ﻿albicorne* (Meigen, 1838)

Naya 1988, **MA**, Haut Sebou; Kettani et al. 1995, **Rif**, aval Oued Krikra, aval Oued Khemis; Kettani et al. 1996; Kettani et al. 2001; Kettani and El Ouazzani 2005, **Rif**, amont Oued Nakhla; Dakki et al. 2008, **MA**, Oued Sebou; Kettani and Langton 2012; Kettani and Moubayed-Breil 2018, **Rif**

﻿*Polypedilum (Polypedilum) ﻿arundineti* (Goetghebuer, 1921)

Azzouzi et al. 1992, **HA**, Oued Tensift; Kettani et al. 1994, **Rif**, aval Oued Talembote (usine éléctrique); Kettani et al. 1996; Dakki 1997; Kettani et al. 1997, **Rif**, Maggou (Oued Laou); Kettani et al. 2001; Kettani and El Ouazzani 2005, **Rif**, amont Oued Nakhla; Kettani et al. 2010, **Rif**, Oued Talembote (aval affluent Talembote, 155 m); Kettani and Langton 2012

﻿*Polypedilum (Polypedilum) ﻿laetum* (Meigen, 1818)

Azzouzi et al. 1992, **HA**, Oued Tensift; Kettani et al. 2001; Kettani et al. 2010, **Rif**, Oued Kelaâ (Akoumi, 400 m); Kettani and Langton 2012; Kettani and Moubayed-Breil 2018, **Rif**

﻿*Polypedilum (Polypedilum) ﻿nubeculosum* (Meigen, 1804)

Reiss 1977, **Rif**, Environ de Tétouan; Fittkau and Reiss 1978; Azzouzi and Laville 1987, **Rif**, Environ Tétouan, **MA**, Oued Sebou; Kettani et al. 1995, **Rif**, aval Oued El Kbir, aval Oued Krikra, aval Oued Khemis, Oued Martil (Tamuda); Kettani et al. 1996; Kettani et al. 1997, **Rif**, Oued Khizana (Oued Laou); Ashe and Cranston 1990; Kettani et al. 2001; Kettani and El Ouazzani 2005, **Rif**, amont Oued Nakhla; Dakki et al. 2008, **MA**, Oued Sebou; Kettani and Langton 2012; Kettani and Moubayed-Breil 2018, **Rif**

﻿*Polypedilum (Polypedilum) ﻿nubifer* (Skuse, 1889)

= ﻿*Polypedilum ﻿﻿﻿pharao* Kieffer, in Reiss 1977: 91, Naya 1998: 55, Ramdani and Tourenq 1982: 180

Kügler and Wool 1968; Reiss 1977, **AP**, Larache, Rabat; Ramdani and Tourenq 1982, **AP**, Merja Sidi Boughaba; Azzouzi and Laville 1987, **AP**, Environ de Larache, Rabat, Merja Sidi Boughaba; Naya 1988, **MA**, Haut Sebou; Ashe and Cranston 1990; Kettani et al. 2001; Dakki et al. 2008, **MA**, Oued Sebou; Kettani and Langton 2012; Kettani and Moubayed-Breil 2018, **Rif**

﻿*Polypedilum (Polypedilum) ﻿pedestre* (Meigen, 1830)

Reiss 1977; Azzouzi and Laville 1987, **MA**, Oued Boufekrane; Kettani et al. 1994, **Rif**, aval Barrage Talembote; Kettani et al. 1995, **Rif**, Oued Mhajrat, aval Oued Khemis; Kettani et al. 1996; Dakki 1997; Kettani et al. 2001; Kettani et al. 2005, **Rif**, Oued Nakhla; Kettani et al. 2010, **Rif**, Oued Talembote (aval Barrage Talembote, 245 m); Kettani and Langton 2012; Kettani and Moubayed-Breil 2018, **Rif**

﻿*Polypedilum (Tripodura) ﻿acifer* Townes, 1945

Reiss 1977, **AA**, Dra-Tal; Azzouzi and Laville 1987, **MA**, Oued Boufekroune, Oued Fès, Oued Sebou; Kettani et al. 1996; Kettani et al. 1997, **Rif**, Maggou (Oued Laou); Kettani et al. 2001; Kettani et al. 2010, **Rif**, Oued Talembote (aval affluent Talembote, 155 m), Oued Laou (Afertane, 55 m); Kettani and Langton 2012

﻿*Polypedilum (Tripodura) ﻿aegyptium* Kieffer, 1925

= ﻿*Polypedilumpruina* Freeman, in Reiss 1977: 91

Reiss 1977, **AP**, Larache, **HA**, Marrakech, **AA**, Dra-Tal; Reiss 1985; Azzouzi and Laville 1987, **AP**, Larache, **HA**, Marrakech, **AA**, Gorges de Todra; Kettani et al. 1994, **Rif**, Haut Laou, Oued Siflaou, aval Oued Talembote (usine éléctrique), Oued Afertane; Kettani et al. 1995, **Rif**, aval Oued El Kbir, aval Oued Krikra, Oued El Kbir, amont Oued Nakhla, Oued Mhajrat, aval Oued Khemis, Oued Martil (Tamuda); Kettani et al. 1996; Dakki 1997; Kettani et al. 2001; Kettani and El Ouazzani 2005, **Rif**, Oued Nakhla; Kettani et al. 2010, **Rif**, Oued Tassikeste (Afechtal, 240 m); Kettani and Langton 2012

﻿*Polypedilum (Tripodura) ﻿bicrenatum* Kieffer, 1921

Azzouzi and Laville 1987, **MA**, Oued Sebou; Kettani et al. 2001; Kettani and Langton 2012

﻿*Polypedilum (Tripodura) ﻿pullum* (Zetterstedt, 1838)

El Mezdi and Giudicelli 1985, **HA**, Khettaras de Marrakech; Azzouzi and Laville 1987, **MA**, Oued Boufekrane, Oued Oum-er-Rbia, **HA**, Oued Tensift; Kettani et al. 2001; Kettani and Langton 2012

﻿*Polypedilum (Tripodura) ﻿quadriguttatum* Kieffer, 1921

Naya 1988, **MA**, Moyen Sebou; Kettani et al. 1995, **Rif**, aval Oued Khemis; Kettani et al. 1996; Kettani et al. 2001; Kettani and El Ouazzani 2005, **Rif**, amont Oued Nakhla; Dakki et al. 2008, **MA**, Oued Sebou; Kettani and Langton 2012

﻿*Polypedilum (Tripodura) ﻿scalaenum* (Schrank, 1803)

Reiss 1977, **AA**, Dra-Tal; Fittkau and Reiss 1978; Azzouzi and Laville 1987; Kettani et al. 1996, **Rif**, Ras el Ma (Chefchaouen); Ashe and Cranston 1990; Kettani et al. 2001; Kettani and Langton 2012

﻿*Polypedilum (Tripodura) ﻿tetracrenatum* Hirvenoja, 1962

Azzouzi et al. 1992, **HA**, Oued Tensift; Kettani et al. 2001; Kettani and Langton 2012

﻿*Polypedilum (Tripodura) ﻿tridens* Freeman, 1955

El Mezdi and Giudicelli 1985, **HA**, Khettaras de Marrakech; Kettani et al. 2001; Kettani and Langton 2012

﻿*Polypedilum (Uresipedilum) ﻿convictum* (Walker, 1856)

Reiss 1977, **AP**, Environ de Larache; Fittkau and Reiss 1978; Azzouzi and Laville 1987, **MA**, Oued Boufekrane (Gantra Mdez), Naya 1988, **MA**, Haut Sebou; Kettani et al. 1994, **Rif**, Haut Laou, Oued Siflaou, Oued Moulay Bouchta, aval Barrage Talembote, aval Oued Tassikeste; Kettani et al. 1995, **Rif**, aval Oued El Kbir, aval Oued Krikra, Oued El Kbir, amont Oued Nakhla, Oued Mhajrat, aval Oued Khemis; Kettani et al. 1996; Dakki 1997; Kettani et al. 1997, **Rif**, Maggou (Oued Laou), Oued Khizana (Oued Laou), Ras el Ma (Chefchaouen); Kettani et al. 2001; Kettani and El Ouazzani 2005, **Rif**, Oued Nakhla; Dakki et al. 2008, **MA**, Oued Sebou; Kettani et al. 2010, **Rif**, Oued pont Béni M’Hamed (Béni M’Hamed, 1330 m), Oued Talembote (avant village Talembote, 320 m), Oued Talembote (aval Barrage Talembote, 245 m), Oued Tassikeste (Afechtal, 240 m); Kettani and Langton 2012; Kettani and Moubayed-Breil 2018, **Rif**

﻿*Polypedilum (Uresipedilum) ﻿cultellatum* Goetghebuer, 1931

Fittkau and Reiss 1978; Azzouzi and Laville 1987, **MA**, Oued Oum-er-Rbia; Kettani et al. 1996, **Rif**, Oued Nakhla; Kettani et al. 2001; Kettani and El Ouazzani 2005, **Rif**, Oued Nakhla; Kettani and Langton 2012

﻿*Polypedilum ﻿﻿﻿ontario*-group ﻿﻿sp. 1

Kettani et al. 1995, **Rif**, aval Oued Khemis; Kettani et al. 1996; Kettani et al. 2001; Kettani and Langton 2012

﻿***Rheomus* Laville & Reiss, 1988**

﻿*Rheomus ﻿﻿﻿alatus* Laville & Reiss, 1988

Azzouzi and Laville 1987, **HA**, Oued Tensift; Kettani et al. 2001; Kettani and Langton 2012

﻿*Rheomus ﻿﻿﻿yahiae* Laville & Reiss, 1988

Azzouzi and Laville 1987, **MA**, Oued Fès; Kettani et al. 2001; Kettani and Langton 2012

﻿***Stenochironomus* Kieffer, 1919**

﻿*Stenochironomus ﻿﻿﻿gibbus* Fabricius, 1794

Kettani and Moubayed-Breil 2018, **Rif**

﻿***Stictochironomus* Kieffer, 1919**

﻿*Stictochironomus ﻿﻿﻿caffrarius* (Kieffer, 1921)

Reiss 1977; Azzouzi and Laville 1987; Kettani et al. 2001; Kettani and Langton 2012

﻿*Stictochironomusmaculipennis* (Meigen, 1818)

Azzouzi and Laville 1987, **MA**, Oued Sebou; Kettani et al. 1994, **Rif**, Haut Laou, Oued Siflaou, Oued Afertane; Kettani et al. 1995, **Rif**, aval Oued Khemis, Oued Martil (Tamuda); Kettani et al. 1996; Dakki 1997; Kettani et al. 1997, **Rif**, Maggou (Oued Laou), Oued Khizana (Oued Laou), Ras el Ma (Chefchaouen); Kettani et al. 2001; Kettani and El Ouazzani 2005, **Rif**, amont Oued Nakhla; Kettani et al. 2010, **Rif**, Oued Laou (Afertane, 55 m); Dakki et al. 2008, **MA**, Oued Sebou; Kettani and Langton 2012; Kettani and Moubayed-Breil 2018, **Rif**

﻿*Stictochironomus ﻿﻿﻿pictulus* (Meigen, 1830)

Reiss 1977, **AP**, Environ de Larache; Fittkau and Reiss 1978; Azzouzi and Laville 1987; Ashe and Cranston 1990; Kettani et al. 1994, **Rif**, Haut Laou, Oued Siflaou; Kettani et al. 1995, **Rif**, aval Oued Kbir, aval Oued Krikra, Oued El Kbir; Kettani et al. 1996; Dakki 1997; Kettani et al. 1997, **Rif**, Oued Khizana (Oued Laou); Kettani et al. 2001; Kettani and El Ouazzani 2005, **Rif**, Oued Nakhla; Kettani and Langton 2012

﻿*Stictochironomus ﻿﻿﻿rosenschoeldi* Zetterstedt, 1838

Kettani and Moubayed-Breil 2018, **Rif**

﻿*Stictochironomus ﻿﻿﻿reissi* Cranston, 1989

= ﻿*Stictochironomus* ﻿﻿sp. nov. Reiss, in Reiss 1977: 91

Reiss 1977; Azzouzi and Laville 1987, **AA**, M’Hamid, Dra-Tal; Kettani et al. 2001; Kettani and Langton 2012

﻿*Stictochironomus ﻿﻿﻿sticticus* (Fabricius, 1781)

= ﻿*Stictochironomus ﻿﻿﻿histrio* (Fabricius, 1794), in Kettani et al. 1996: 138

Azzouzi and Laville 1987, **HA**, Oued Tensift; Kettani et al. 2001; Kettani and Langton 2011, **Rif**, Oued Berranda (Bouztate, 1259 m), Dayat Dalia (SIBE Jebel Moussa); Kettani et al. 2010, **Rif**, Oued Kelaâ (Akoumi, 400 m); Kettani and Langton 2012

﻿*Stictochironomus* Pe 2 Langton 1991

Kettani et al. 2001

﻿***Xenochironomus* Kieffer, 1921**

﻿*Xenochironomus ﻿﻿﻿xenolabis* (Kieffer, 1916)

Azzouzi and Laville 1987, **MA**, Oued Fès; Kettani et al. 2001; Kettani and Langton 2012

##### Tanytarsini

﻿***Cladotanytarsus* Kieffer, 1921**

﻿*Cladotanytarsus (Cladotanytarsus) ﻿atridorsum* Kieffer, 1924

Ramdani and Tourenq 1982, **AP**, Merja Sidi Boughaba; Azzouzi and Laville 1987; Azzouzi et al. 1992, **HA**, Aït Saoun, Gorges de Dadès (1900 m), vallée de Drâa, Marrakech; Kettani et al. 1994, **Rif**, Haut Laou, Oued Siflaou, aval Barrage Talembote, aval Oued Laou; Kettani et al. 1995, **Rif**, aval Oued El Kbir, aval Oued Krikra, amont Oued Nakhla, aval Oued Khemis; Kettani et al. 1996; Dakki 1997; Kettani et al. 1997, **Rif**, Oued Khizana (Oued Laou); Kettani et al. 2001; Kettani et al. 2010, **Rif**, Oued Talembote (aval Barrage Talembote, 245 m); Kettani and Langton 2012; Kettani and Moubayed-Breil 2018, **Rif**

﻿*Cladotanytarsus (Cladotanytarsus) ﻿capensis* (Freeman, 1954)

El Mezdi and Giudicelli 1985, **HA**, Khettaras de Marrakech; Kettani et al. 2001; Kettani and Langton 2012

﻿*Cladotanytarsus (Cladotanytarsus) ﻿ecristatus* Reiss, 1991

= ﻿*Tanytarsus* ﻿﻿sp. nov. (Morokko) Reiss, in Azzouzi and Laville 1987: 219

Reiss 1977, **AA**, Dra-Tal; Azzouzi and Laville 1987, **EM**, Berkane; Reiss 1991; Azzouzi et al. 1992, **HA**; Kettani et al. 2001; Kettani and Langton 2012

﻿*Cladotanytarsus (Cladotanytarsus) ﻿mancus* (Walker, 1856)

Ramdani and Tourenq 1982, **AP**, Merja Sidi Boughaba; Azzouzi and Laville 1987; Kettani et al. 2001; Kettani and Langton 2012; Kettani and Moubayed-Breil 2018, **Rif**

﻿*Cladotanytarsus (Cladotanytarsus) ﻿pallidus* Kieffer, 1922

= ﻿*Cladotanytarsus* ﻿Pe 5 Langton 1984

Azzouzi and Laville 1987, **MA**, Oued Sebou, Oum Rbia; Kettani et al. 2001; Kettani and Langton 2012

﻿*Cladotanytarsus (Cladotanytarsus) ﻿vanderwulpi* (Edwards, 1929)

Azzouzi and Laville 1987, **HA**, Oued Tensift; Kettani et al. 1995, **Rif**, aval Oued El Kbir, Oued Mhajrat, Oued Martil (Tamuda); Kettani et al. 1996; Kettani et al. 2001; Kettani and Langton 2012; Kettani and Moubayed-Breil 2018, **Rif**

﻿***Lithotanytarsus* Thienemann, 1933**

﻿*Lithotanytarsus ﻿﻿﻿dadesi* Reiss, 1991

Reiss 1991; Azzouzi et al. 1992, **HA**, Gorges de Dadès (Imdiazen, 1900 m); Kettani et al. 1994, **Rif**, aval Oued Talembote (usine éléctrique), Oued Afertane; Kettani et al. 1995, **Rif**, Oued Mhajrat; Kettani et al. 1996; Dakki 1997; Kettani et al. 1997, **Rif**, Maggou (Oued Laou), Oued Khizana (Oued Laou); Kettani et al. 2001; Kettani et al. 2010, **Rif**, Oued Tassikeste (Afechtal, 240 m), Oued Talembote (aval affluent Talembote, 155 m), Oued Laou (Afertane, 55 m); Kettani and Langton 2012; Kettani and Moubayed-Breil 2018, **Rif**

﻿*Lithotanytarsus ﻿﻿﻿emarginatus* (Goetghebuer, 1933)

Azzouzi and Laville 1987, **MA**, Oued Oum-er-Rbia; Kettani and Langton 2012

﻿***Micropsectra* Kieffer, 1909**

﻿*Micropsectra ﻿﻿﻿﻿﻿﻿andalusiaca* Marcuzzi, 1950

Kettani and Moubayed-Breil 2018, **Rif**

﻿*Micropsectra ﻿﻿﻿apposita* (Walker, 1856)

= ﻿*Micropsectra ﻿﻿﻿contracta* Reiss, 1965

Azzouzi et al. 1992, **HA**, Oued Tensift; Kettani et al. 1994, **Rif**, aval Oued Talembote (usine éléctrique); Kettani et al. 1996; Dakki 1997; Kettani et al. 1997, **Rif**, Ras el Ma (Chefchaouen); Kettani et al. 2001; Kettani and El Ouazzani 2005, **Rif**, amont Oued Nakhla; Kettani et al. 2010, **Rif**, Oued Chrafat (Armotah, 900 m), Oued Kelaâ (Akoumi, 400 m), Oued Talembote (aval affluent Talembote, 155 m); Kettani and Langton 2012; Kettani and Moubayed-Breil 2018, **Rif**

﻿*Micropsectra ﻿﻿﻿aristata* Pinder, 1976

Kettani and Langton 2012, **Rif**, Oued Zarka

﻿*Micropsectra ﻿﻿﻿atrofasciata* (Kieffer, 1911)

= ﻿*Micropsectra ﻿﻿﻿bidentata* (Goetghebuer, 1921), in Azzouzi et al. 1992: 230; Kettani et al. 2001: 332; Kettani and Langton 2011: 590, 2012: 424

Fittkau and Reiss 1978; El Mezdi and Giudicelli 1985, **HA**, Khettaras de Marrakech; Azzouzi and Laville 1987, **MA**, Oued Sebou (Arhbalou Aberchane), Oued Oum-er-Rbia; Naya 1988, **MA**, Haut Sebou; Azzouzi et al. 1992, **HA**, Oued Tensift; Kettani et al. 1994, **Rif**, Haut Laou, Oued Siflaou, Oued Moulay Bouchta, aval Oued Talembote (usine éléctrique); Kettani et al. 1995, **Rif**, aval Oued El Kbir, aval Oued Krikra, Oued El Kbir, Oued Mhajrat, aval Oued Khemis; Kettani et al. 1996; Dakki 1997; Kettani et al. 1997, **Rif**, Oued Khizana (Oued Laou), Ras el Ma (Chefchaouen); Kettani et al. 2001; Kettani et al. 2010, **Rif**, Oued Madissouka (Talassemtane, 1530 m), Oued Chrafat (Armotah, 900 m), Oued Kelaâ (Akoumi, 400 m), Oued Talembote (aval affluent Talembote, 155 m); Dakki et al. 2008, **MA**, Oued Sebou; Kettani and Langton 2011, **Rif**, Oued Taida (Moulay Abdelsalam, 650 m), cascade Zarka, Dayat En-Nâsser (Khandek En-Nâsser, 1177 m); Kettani and Langton 2012; Kettani and Moubayed-Breil 2018, **Rif**

﻿*Micropsectrajunci* (Meigen, 1818)

Azzouzi et al. 1992, **HA**, Oued Tensift; Kettani et al. 2001; Kettani and Langton 2012; Kettani and Moubayed-Breil 2018, **Rif**

﻿*Micropsectra ﻿﻿﻿lacustris* Säwedal, 1975

Kettani and Langton 2012, **Rif**, Oued Zarka

﻿*Micropsectra ﻿﻿﻿lindrothi* Goetghebuer, 1931

Azzouzi et al. 1992, **HA**, Oued Tensift; Kettani et al. 2001; Kettani and Langton 2012; Kettani and Moubayed-Breil 2018, **Rif**

﻿*Micropsectra ﻿﻿﻿notescens* (Walker, 1856)

Kettani et al. 2010, **Rif**, Oued Talembote (aval affluent Talembote, 155 m); Kettani and Langton 2011, **Rif**, Oued Ketama, Oued Sgara, ruisselet Bab Tariouant, Oued Berranda (Bouztate, 1259 m), source Bab Karn (Fifi, 1220 m), Dayat Fifi (Fifi, 1179); Kettani and Langton 2012; Kettani and Moubayed-Breil 2018, **Rif**

﻿*Micropsectra ﻿﻿﻿pallidula* (Meigen, 1830)

Kettani and Moubayed-Breil 2018, **Rif**

﻿*Micropsectra ﻿﻿﻿schrankelae* Stur & Ekrem, 2006

Kettani and Moubayed-Breil 2018, **Rif**

﻿*Micropsectra ﻿﻿﻿zernyi* Marcuzzi, 1950

Kettani and Moubayed-Breil 2018, **Rif**

﻿***Paratanytarsus* Thienemann & Bause, 1913**

﻿*Paratanytarsus ﻿﻿﻿bituberculatus* (Edwards, 1929)

Azzouzi et al. 1992, **MA**, Lac Aguelmane Azigza (1510 m); Kettani et al. 1995, **Rif**, Oued Martil (Tamuda); Kettani et al. 1996; Kettani et al. 1997, **Rif**, Oued Khizana (Oued Laou); Kettani et al. 2001; Kettani and Langton 2012

﻿*Paratanytarsus ﻿﻿﻿dissimilis* (Johannsen, 1905)

= ﻿*Paratanytarsus ﻿﻿﻿confusus* Palmén, 1960, in Naya 1988: 40; Dakki et al. 2008: 32; Kettani et al. 2001: 332; Kettani and Langton 2012: 423

Naya 1988, **MA**, Haut Sebou; Dakki et al. 2008, **MA**, Oued Sebou; Azzouzi et al. 1992, **HA**, Oued Tensift; Kettani et al. 1996; Kettani et al. 1997, **Rif**, Oued Khizana (Oued Laou); Kettani et al. 2001; Kettani and Langton 2012; Kettani and Moubayed-Breil 2018, **Rif**

﻿*Paratanytarsus ﻿﻿﻿grimmii* (Schneider, 1885)

Kettani et al. 2010, **Rif**, Oued Laou (Afertane, 55 m); Kettani and Langton 2012

﻿*Paratanytarsus ﻿﻿﻿inopertus* (Walker, 1856)

Reiss 1977, **Rif**, Environ Tétouan; Fittkau and Reiss 1978; Reiss and Säwedal 1981; Azzouzi and Laville 1987; Ashe and Cranston 1990; Kettani et al. 2001; Kettani and Langton 2011, **Rif**, merja Mtalssi (Tamuda, 31 m); Kettani and Langton 2012; Kettani and Moubayed-Breil 2018, **Rif**

﻿*Paratanytarsus ﻿﻿﻿mediterraneus* Reiss & Säwedal, 1981

Reiss and Säwedal 1981, **Rif**, Estuaire Oued Mharka (Tanger), **AP**, Oued Loukous; Azzouzi and Laville 1987; Ashe and Cranston 1990; Kettani et al. 2001; Kettani and Langton 2011, **AP**, marais de Loukous; Kettani and Langton 2012

﻿*Paratanytarsus ﻿﻿﻿tenellulus* (Goetghebuer, 1921)

= *Microspsectra ﻿﻿﻿tenellula*Reiss 1977: 91; Azzouzi and Laville 1987: 219

Reiss 1977, **MA**, Lac Kranichsee; Fittkau and Reiss 1978; Azzouzi and Laville 1987; Ashe and Cranston 1990; Kettani et al. 2001; Kettani and Langton 2012

﻿*Paratanytarsus ﻿﻿﻿tenuis* (Meigen, 1830)

= ﻿*Tanytarsus ﻿﻿﻿tenuis* Meigen, in Naya 1988: 57

Naya 1988, **MA**, Moyen Sebou; Kettani et al. 1995, **Rif**, aval Oued El Kbir, aval Oued Krikra, Oued El Kbir; Kettani et al. 1996; Kettani et al. 1997, **Rif**, Oued Khizana (Oued Laou); Kettani et al. 2001; Dakki et al. 2008, **MA**, Oued Sebou; Kettani and Langton 2012

﻿***Rheotanytarsus* Thienemann & Bause, 1913**

﻿*Rheotanytarsus ﻿﻿﻿ceratophylli* Dejoux, 1973

Naya 1988, **MA**, Moyen et Bas Sebou; Kettani et al. 2001; Dakki et al. 2008, **MA**, Oued Sebou; Kettani and Langton 2012

﻿*Rheotanytarsus ﻿﻿﻿curtistylus* (Goetghebuer, 1921)

Azzouzi et al. 1992, **HA**, Oasis Meski (1160 m); Kettani et al. 2001; Kettani and Langton 2012; Kettani and Moubayed-Breil 2018, **Rif**

﻿*Rheotanytarsus ﻿langtoni* Moubayed & Kettani, 2018

Moubayed-Breil and Kettani 2018, **Rif**, Oued Farda; Moubayed-Breil and Kettani 2019, **Rif**, Chrafate, Challal Sghir (Akchour)

﻿*Rheotanytarsus ﻿﻿﻿muscicola* Thienemann, 1929

Reiss 1977, **AP**, Environ de Larache, **AA**, Dra-Tal (Tissint Moyen Dra); Fittkau and Reiss 1978; Azzouzi and Laville 1987; Ashe and Cranston 1990; Kettani et al. 2001; Kettani and Langton 2012; Kettani and Moubayed-Breil 2018, **Rif**

﻿*Rheotanytarsus ﻿﻿﻿nigricauda* Fittkau, 1960

Kettani and Moubayed-Breil 2018, **Rif**

﻿*Rheotanytarsus ﻿﻿﻿pellucidus* (Walker, 1818)

= ﻿*Rheotanytarsus ﻿﻿﻿distinctissimus* (Brundin, 1947), in Kettani et al. 1995: 258; Kettani et al. 1996: 138, 1997: 185; Kettani and Langton 2012: 424

Kettani et al. 1995, **Rif**, aval Oued El Kbir, aval Oued Krikra; Kettani et al. 1996; Kettani et al. 1997, **Rif**, Maggou (Oued Laou); Kettani and Langton 2012; Kettani and Moubayed-Breil 2018, **Rif**

﻿*Rheotanytarsus ﻿﻿﻿pentapoda* (Kieffer, 1909)

= ﻿*Rheotanytarsus* sp 1, in Kettani et al. 1994: 28

Kettani et al. 1994, **Rif**, Oued Siflaou, Oued Moulay Bouchta, aval Barrage Talembote; Kettani et al. 1995, **Rif**, aval Oued Krikra, Oued El Kbir, amont Oued Nakhla, Oued Mhajrat, aval Oued Khemis; Kettani et al. 1996; Dakki 1997; Kettani et al. 1997, **Rif**, Maggou (Oued Laou), Oued Khizana (Oued Laou), Ras el Ma (Chefchaouen); Kettani et al. 2001; Kettani and El Ouazzani 2005, **Rif**, amont Oued Nakhla; Kettani et al. 2010, **Rif**, Source Maggou (Maggou, 1300 m), Oued Talembote (avant village Talembote, 320 m), Oued Talembote (aval Barrage Talembote, 245 m); Kettani and Langton 2012; Kettani and Moubayed-Breil 2018, **Rif**; Moubayed-Breil and Kettani 2019, **Rif**, Chrafate, Challal Sghir (Akchour)

﻿*Rheotanytarsus ﻿﻿﻿photophilus* (Goetghebuer, 1921)

Naya 1988, **MA**, Haut Sebou; Kettani et al. 2001; Kettani and Langton 2012

﻿*Rheotanytarsus ﻿﻿﻿procerus* Reiss, 1991

Reiss 1991, **HA**; Azzouzi et al. 1992, **HA**, Gorges de Dadès (Imdiazen, 1900 m); Kettani et al. 2001; Kettani and Langton 2012; Kettani and Moubayed-Breil 2018, **Rif**

﻿*Rheotanytarsus ﻿﻿﻿reissi* Lehmann, 1970

Lehmann, 1970; Azzouzi and Laville 1987, **MA**, Oued Boufekrane, Oued Oum-er-Rbia; Kettani et al. 1994, **Rif**, Haut Laou, Oued Siflaou, Oued Moulay Bouchta, aval Barrage Talembote, aval Oued Talembote (usine éléctrique), Oued Afertane, aval Oued Tassikeste, aval Oued Laou; Kettani et al. 1995, **Rif**, aval Oued El Kbir, aval Oued Krikra, Oued El Kbir, amont Oued Nakhla, Oued Mhajrat, aval Oued Khemis, Oued Martil (Tamuda); Kettani et al. 1996; Dakki 1997; Kettani et al. 1997, **Rif**, Maggou (Oued Laou), Oued Khizana (Oued Laou), Ras el Ma (Chefchaouen); Kettani et al. 2001; Kettani and El Ouazzani 2005, **Rif**, amont Oued Nakhla; Kettani et al. 2010, **Rif**, Source Maggou (Maggou, 1300 m), Oued Kelaâ (Akoumi, 400 m), Oued Talembote (aval Barrage Talembote, 245 m), Oued Tassikeste (Afechtal, 240 m), Oued Talembote (aval affluent Talembote, 155 m), Oued Laou (Afertane, 55 m); Kettani and Langton 2012; Kettani and Moubayed-Breil 2018, **Rif**

﻿*Rheotanytarsus ﻿﻿﻿rhenanus* Klink, 1983

Kettani and Moubayed-Breil 2018, **Rif**

﻿*Rheotanytarsus ﻿﻿﻿ringei* Lehmann, 1970

Lehmann, 1970; Reiss 1977, **Rif**, Environ Tétouan; Fittkau and Reiss 1978; Azzouzi and Laville 1987, **Rif**, Tétouan, **MA**, Oued Boufekrane, Oued Fès, Oued Sebou, Oued Oum-er-Rbia; Kettani et al. 2001; Kettani and Langton 2012; Kettani and Moubayed-Breil 2018, **Rif**

﻿*Rheotanytarsus* ﻿Pe 3 Langton 1991

Kettani et al. 2010; Kettani and Langton 2011, **Rif**, Oued Sgara (Ketama, 1300 m); Kettani and Langton 2012

﻿***Stempellina* Thienemann & Bause, 1913**

﻿*Stempellina ﻿﻿﻿almi* Brundin, 1947

Fittkau and Reiss 1978; Azzouzi and Laville 1987, **MA**, Oued Boufekrane; Kettani et al. 2001; Kettani and Langton 2012

﻿*Stempellina ﻿﻿﻿bausei* (Kieffer, 1911)

Kettani and Langton 2012, **Rif**, Ketama; Kettani and Moubayed-Breil 2018, **Rif**

﻿***Stempellinella* Brundin, 1947**

﻿*Stempellinella ﻿﻿﻿brevis* (Edwards, 1929)

Kettani et al. 2010, **Rif**, Oued Kelaâ (Akoumi, 400 m); Kettani and Langton 2012

﻿***Tanytarsus* van der Wulp, 1874**

﻿*Tanytarsus ﻿﻿﻿brundini* Lindeberg, 1963

Kettani et al. 1994, **Rif**, Oued Moulay Bouchta, aval Oued Laou; Kettani et al. 1995, **Rif**, amont Oued Nakhla, aval Oued Khemis; Kettani et al. 1996; Kettani et al. 2001; Dakki 1997; Kettani and Langton 2012; Kettani and Moubayed-Breil 2018, **Rif**

﻿*Tanytarsus ﻿﻿﻿chinyensis* Goetghebuer, 1934

Azzouzi et al. 1992, **HA**, Oued Tensift; Kettani et al. 2001; Kettani and Langton 2011, **Rif**, Dayat Fifi (Fifi, 1179 m); Kettani and Langton 2012; Kettani and Moubayed-Breil 2018, **Rif**

﻿*Tanytarsus ﻿﻿﻿cretensis* Reiss, 1987

= ﻿*Tanytarsus* ﻿﻿sp. nov. (*creticus*), in Reiss 1977: 91; Azzouzi and Laville 1987: 219

= ﻿*Cladotanytarsus* sp 1, in Kettani et al. 1995: 258

Reiss and Fittkau 1971; Reiss 1977, **EM**, Environ de Berkane; Reiss 1987; Azzouzi and Laville 1987, **Rif**, Tétouan, **AP**, Larache, Kénitra; Kettani et al. 1996; Kettani et al. 2001; Kettani and Langton 2012

﻿*Tanytarsus ﻿﻿﻿dibranchius* Kieffer, 1926

= ﻿*Tanytarsus ﻿﻿﻿separabilis* Brundin, 1947, in Kettani et al. 1994: 29; Kettani et al. 1995: 258, 1996: 138, 2001: 332; Dakki 1997: 63; Kettani and Langton 2012: 424

Kettani et al. 1994, **Rif**, Haut Laou, Oued Siflaou, aval Barrage Talembote, aval Oued Talembote (usine éléctrique); Kettani et al. 1995, **Rif**, aval Oued El Kbir, aval Oued Krikra, amont Oued Nakhla, Oued Mhajrat, aval Oued Khemis, Oued Martil (Tamuda); Kettani et al. 1996; Dakki 1997; Kettani et al. 2001; Kettani and Langton 2012

﻿*Tanytarsus ﻿﻿﻿ejuncidus* (Walker, 1856)

Kettani and Moubayed-Breil 2018, **Rif**

﻿*Tanytarsus ﻿﻿﻿eminulus* (Walker, 1856)

Kettani et al. 2010, **Rif**, Oued Kelaâ (Akoumi, 400 m); Kettani and Langton 2012; Kettani and Moubayed-Breil 2018, **Rif**

﻿*Tanytarsus ﻿﻿﻿formosanus* Kieffer, 1912

= ﻿*Tanytarsus ﻿﻿﻿horni* Goetghebuer, 1934, in Reiss and Fittkau 1971: 122; Reiss 1977: 91; Fittkau and Reiss 1978: 439; Ramdani and Tourenq 1982: 180; El Mezdi and Giudicelli 1985: 292; Azzouzi and Laville 1987: 219; Ashe and Cranston 1990: 341; Kettani et al. 2001: 332; Kettani and Langton 2012: 424

Reiss and Fittkau 1971, **Rif**, M’Diq; Reiss 1977, **Rif**, Environ Tétouan, **AP**, Larache, Rabat, Kénitra; Fittkau and Reiss 1978; Ramdani and Tourenq 1982, **AP**, Merja Sidi Boughaba; El Mezdi and Giudicelli 1985, **HA**, Khettaras de Marrakech; Azzouzi and Laville 1987, **HA**, Oued Tensift; Ashe and Cranston 1990; Kettani et al. 2001; Kettani and Langton 2012; Kettani and Moubayed-Breil 2018, **Rif**

﻿*Tanytarsus ﻿﻿﻿gregarius* Kieffer, 1909

Naya 1988, **MA**, Moyen Sebou; Kettani et al. 2001; Dakki et al. 2008, **MA**, Oued Sebou; Kettani and Langton 2012

﻿*Tanytarsus ﻿﻿﻿heusdensis* Goetghebuer, 1923

Reiss 1977, **AA**, Dra-Tal; Fittkau and Reiss 1978; Azzouzi and Laville 1987; Ashe and Cranston 1990; Kettani et al. 1994, **Rif**, aval Oued Talembote (usine éléctrique); Kettani et al. 1996; Dakki 1997; Kettani et al. 1997, **Rif**, Maggou (Oued Laou), Oued Khizana (Oued Laou); Kettani et al. 2001; Kettani et al. 2010, **Rif**, Oued Kelaâ (Akoumi, 400 m), Oued Talembote (avant village Talembote, 320 m), Oued Talembote (aval affluent Talembote, 155 m), Oued Laou (Ifansa, 105 m); Kettani and Langton 2012; Kettani and Moubayed-Breil 2018, **Rif**

﻿*Tanytarsus ﻿﻿﻿mendax* Kieffer, 1925

Kettani and Moubayed-Breil 2018, **Rif**

﻿*Tanytarsus ﻿﻿﻿medius* Reiss & Fittkau, 1971

Kettani et al. 1994, **Rif**, Haut Laou, Oued Siflaou, Oued Moulay Bouchta, aval Barrage Talembote, aval Oued Laou; Kettani et al. 1995, **Rif**, aval Oued El Kbir, aval Oued Krikra, Oued El Kbir, amont Oued Nakhla, Oued Mhajrat, aval Oued Khemis, Oued Martil (Tamuda); Kettani et al. 1996; Dakki 1997; Kettani et al. 1997, **Rif**, Maggou (Oued Laou), Oued Khizana (Oued Laou), Ras el Ma (Chefchaouen); Kettani et al. 2001; Kettani and El Ouazzani 2005, **Rif**, amont Oued Nakhla; Kettani et al. 2010, **Rif**, Oued Talembote (aval Barrage Talembote, 245 m), Oued Talembote (aval affluent Talembote, 155 m); Kettani and Langton 2012; Kettani and Moubayed-Breil 2018, **Rif**

﻿*Tanytarsus ﻿﻿﻿palettaris* Verneaux, 1969

Kettani et al. 1994, **Rif**, aval Oued Laou; Kettani et al. 1996; Dakki 1997; Kettani et al. 2001; Kettani and Langton 2012; Kettani and Moubayed-Breil 2018, **Rif**

﻿*Tanytarsus ﻿﻿﻿pallidicornis* (Walker, 1856)

Kettani and Langton 2011, **Rif**, Dayat Fifi (Fifi, 1179 m), Oued El Hatba (SIBE Jebel Moussa, 165 m); Kettani and Langton 2012

﻿*Tanytarsus ﻿﻿﻿recurvatus* Brundin, 1947

Kettani and Langton 2011, **Rif**, Oued El Hamma (El Hamma, 240 m); Kettani and Langton 2012

﻿*Tanytarsus ﻿﻿﻿signatus* (van der Wulp, 1859)

= ﻿*Tanytarsus* ﻿Pe 5 Langton 1991, in Azzouzi and Laville 1987: 219

Kügler and Reiss 1973; Reiss 1977, **AA**, Dra-Tal; Azzouzi and Laville 1987; Kettani et al. 2001; Kettani and Langton 2011, **Rif**, Dayat Aïn Rami (373 m), Dayat Amlay (258 m); Kettani and Langton 2012; Kettani and Moubayed-Breil 2018, **Rif**

﻿*Tanytarsus ﻿﻿﻿verralli* Goetghebuer, 1928

Kettani and Langton 2011, **Rif**, Oued Taida (650 m); Kettani and Langton 2012

﻿*Tanytarsus ﻿﻿﻿volgensis* Miseiko, 1967

= ﻿*Tanytarsus ﻿﻿﻿fimbriatus* Reiss & Fittkau, 1971, in Fittkau and Reiss 1978: 439; Azzouzi and Laville 1987: 219; Kettani et al. 2001: 332; Kettani and Langton 2012: 424

Fittkau and Reiss 1978; Azzouzi and Laville 1987, **MA**, Oued Fès, Oued Sebou, **HA**, Oued Tensift; Kettani et al. 2001; Kettani and Langton 2012; Kettani and Moubayed-Breil 2018, **Rif**

﻿*Tanytarsus* ﻿Pe 14 Langton 1991

Kettani and Langton 2011, **Rif**, source Issaguen (Ketama, 1600 m); Kettani and Langton 2012

﻿*Tanytarsus* ﻿Pe 23 Langton 1991

Kettani and Langton 2011, **Rif**, Oued El Hamma (El Hamma, 240 m); Kettani and Langton 2012

﻿***Virgatanytarsus* Pinder, 1982**

﻿*Virgatanytarsus ﻿﻿﻿albisutus* (Santos-Abreu, 1918)

= ﻿*Virgatanytarsusmaroccanus* Kügler and Reiss, in Azzouzi and Laville 1987: 219

Fittkau and Reiss 1978; Reiss and Schurch 1984, **AA**, Dra-Tal; Reiss 1986; Azzouzi and Laville 1987, **MA**, Oued Oum-er-Rbia, **AA**, Dra-Tal; Ashe and Cranston 1990; Kettani et al. 1994, **Rif**, Haut Laou, Oued Siflaou, Oued Moulay Bouchta, aval Barrage Talembote, aval Oued Talembote (usine éléctrique), Oued Afertane, aval Oued Tassikeste, aval Oued Laou; Kettani et al. 1995, **Rif**, aval Oued El Kbir, Oued El Kbir, amont Oued Nakhla, Oued Mhajrat, aval Oued Khemis, Oued Martil (Tamuda); Kettani et al. 1996; Dakki 1997; Kettani et al. 1997, **Rif**, Maggou (Oued Laou), Oued Khizana (Oued Laou), Ras el Ma (Chefchaouen); Kettani et al. 2001; Kettani and El Ouazzani 2005, **Rif**, amont Oued Nakhla; Kettani et al. 2010, **Rif**, Oued Talembote (aval Barrage Talembote, 245 m), Oued Talembote (aval affluent Talembote, 155 m); Kettani and Langton 2012; Kettani and Moubayed-Breil 2018, **Rif**

﻿*Virgatanytarsusansatus* Reiss & Schürch, 1984

Reiss and Schurch 1984, **HA**; Azzouzi and Laville 1987, **MA**, Lac Aguelmane Azigza; Ashe and Cranston 1990; Kettani et al. 2001; Kettani and Langton 2012

﻿*Virgatanytarsus ﻿﻿﻿arduennensis* (Goetghebuer, 1922)

Azzouzi et al. 1992, **HA**, Oued Tensift; Kettani et al. 1994, **Rif**, aval Oued Talembote (usine éléctrique); Kettani et al. 1996; Dakki 1997; Kettani et al. 1997, **Rif**, Maggou (Oued Laou); Kettani et al. 2001; Kettani et al. 2010, **Rif**, Oued Talembote (aval affluent Talembote, 155 m); Kettani and Langton 2012; Kettani and Moubayed-Breil 2018, **Rif**

﻿*Virgatanytarsus ﻿﻿﻿triangularis* (Goetghebuer, 1928)

Azzouzi et al. 1992, **HA**, Oued Tensift; Kettani et al. 2001; Kettani and Langton 2012

﻿*Virgatanytarsus* ﻿Pe 1 Langton 1991

Kettani et al. 1996; Kettani et al. 1997, **Rif**, Maggou (Oued Laou), Oued Khizana (Oued Laou); Kettani et al. 2001; Kettani and El Ouazzani 2005, **Rif**, amont Oued Nakhla; Kettani and Langton 2012

﻿***Zavrelia* Kieffer, Thienemann & Bause, 1913**

﻿*Zavrelia ﻿﻿﻿pentatoma* Kieffer & Bause, 1913

Kettani and Langton 2012

﻿*Zavrelia* ﻿Pe 1 Langton, 1991

Kettani and Langton 2011, **Rif**, Oued Berranda (Bouztate, 1259 m); Kettani and Langton 2012

##### Acknowledgment

We gratefully acknowledge the invaluable assistance and cooperation of Patrick Ashe (Dublin, Ireland) who contributed greatly to the revision of this family.

#### ﻿﻿SIMULIIDAE

K. Kettani

Number of species: **43**.

Faunistic knowledge of the family in Morocco: good

##### Simulinae

Prosimuliini

﻿***Helodon* Enderlein, 1921**

﻿*Helodonlaamii* (Beaucournu-Saguez and Bailly-Choumara, 1981)

Beaucournu-Saguez and Bailly-Choumara 1981, **Rif**; Clergue-Gazeau et al. 1991; Hervy et al. 1994; Belqat et al. 2001a; Belqat 2002; Belqat and Dakki 2004; Dakki et al. 2008, **MA**, Oued Sebou; Belqat et al. 2011, **Rif**; Adler and Crosskey 2017; Belqat et al. 2018; Adler 2019

﻿***Prosimulium* Roubaud, 1906**

﻿*Prosimulium ﻿hirtipes* species group

Bailly-Choumara and Beaucournu-Saguez 1981: 53–54: groupe ﻿*latimucro* (species ﻿nova ?); Beaucournu-Saguez and Bailly-Choumara 1981: 119: groupe ﻿*latimucro*, groupe ﻿*tomosvaryi* and groupe ﻿*rufipes*-﻿*hirtipes*; Clergue-Gazeau et al. 1991: 54 as «﻿sp. gr. *Hirtipes*»

﻿*Prosimulium﻿latimucro* (Enderlein, 1925)^4^

Bailly-Choumara and Beaucournu-Saguez 1981; Beaucournu-Saguez and Bailly-Choumara 1981; Giudicelli and Thiery 1985, **HA**; Giudicelli and Bouzidi 1989, **HA**; Giudicelli et al. 2000, **HA**, Oued Réghaya (Neltner, 3800 m), Oued Réghaya (Sidi Chamharouch, 2300 m), Oued Réghaya (lmlil, 1740 m), Oued Réghaya (Aguersioual, 1550 m), Oued Réghaya (Moulay Brahim, 1200 m), Oued Réghaya (Tahanaout, 890 m), ruisselet émissaire de source débouchant dans Oued Réghaya en amont d’lmlil (1750 m), ruisselet émissaire de source débouchant dans l’assif M’zik (1850 m), ruisselet émissaire de source débouchant dans l’assif N’Ouarzane (3000 m), ruisseau émissaire de source (assif N’Ouarzane, 3000 m), assif N’Ouarzane (Irhoulidene, 2800 m), ruisseau affluent en rive droite de l’assif N’Ouarzane (2400 m), Oued N’fis (amont Ijoukak, 1600 m), Oued N’fis (amont Wirgan, 1200 m), Oued N’fis (980 m), Oued N’fis (amont retenue Lalla Takerkoust, 660 m), ruisseau de Tinzart (émissaire de source: 2850 m), ruisseau de Tifni (émissaire de source: 2780 m), ruisseau de Likemt (émissaire de source: 2670 m), ruisseau de Tougroudadene (émissaire de source: 2660 m), assif Oukaimeden (2600 m), source hélocrène au niveau du cirque d’Oukaimeden (2660 m), assif Tiferguine (2500 m), assif Oukaimeden (2450 m), ruisseau émissaire de source débouchant dans l’assif Oukaimeden (1740 m), complexe rhéocrène formé par des émissaires de source débouchant dans l’assif Oukaimeden (1730 m), affluent temporaire en rive gauche de l’assif Oukaimeden (1630 m), affluent temporaire en rive gauche de l’assif Oukaimeden (1360 m), affluent temporaire en rive droite de l’assif Oukaimeden (1260 m), affluent en rive droite de l’assif Oukaimeden (1300 m), assif Tarzaza (1200 m), assif Tarzaza (1000 m), cours inférieur de l’oued Ourika (850 m), Oued Rdat en amont de Taddert (1850 m), affluent temporaire en rive gauche de Oued Rdat (1400 m), Oued Rdat (1600 m), Oued Rdat (1230 m), Oued Tensift (600–700 m), khetarras (450–600 m), Oued Tessaout au niveau d’Aït Tamli (1620 m), Oued Lakdar en aval de la retenue de Sidi Driss (1030 m), ruisseau émissaire de source formant le début de l’assif Imini (2090 m), assif Imini (1560 m), Oued Ounila (1820 m), ruisseau affluent en rive gauche de l’oued Ounila (1820 m), Oued Ounila (Timhlt, 1600 m), Oued Mellah (Anghessa, 1400 m), Oued Dadès en amont des gorges (1630 m), Oued Dadès (Boumalne, 1530 m), Oued Dadès (Sidi Flah, 1100 m), Oued M’goun (1530 m), Oued M’Goum en aval de Kelaâ (1370 m), ruisseau émissaire de source débouchant dans un affluent de l’Oued Souss (2350 m); Adler and Belqat 2001, **Rif**, Oued Iouchirene, Oued Ketama (Al Hoceima); Belqat et al. 2001a, **Rif**, **HA**; Belqat and Adler 2001, **Rif**, Aïn Khandek En Nâsser, Oued Iouchirene, Oued Ketama; Belqat 2002, **Rif**; Belqat and Dakki 2004, **Rif**; Belqat et al. 2005, **Rif**; Koçak and Kemal 2010; Belqat et al. 2011; Adler and Crosskey 2017; Belqat et al. 2018; Adler 2019

﻿*Prosimuliumrachiliense* Djafarov, 1954 (complex)^5^

Beaucournu-Saguez and Bailly-Choumara 1981; Adler and Belqat 2001; Belqat and Adler 2001; Belqat 2002; Belqat and Dakki 2004; Belqat et al. 2005; Belqat et al. 2008; Belqat et al. 2011; Adler and Crosskey 2017; Belqat et al. 2018; Adler 2019

﻿*Prosimulium ﻿﻿﻿tomosvaryi* (Enderlein, 1921)

Beaucournu-Saguez and Bailly-Choumara 1981; Giudicelli and Thiery 1985, **HA**; Giudicelli and Bouzidi 1989, Giudicelli et al. 2000; Adler and Belqat 2001, **Rif**, Oued Iouchirene (Al Hoceima); Belqat and Adler 2001, **Rif**, Oued Ouringa Tamdâ, oued Iouchirene, Oued Mrinet, Oued Ketama, Aîn Ksour, Oued Tisgris, Aîn Sidi Brahim Ben Arrif, Oued Hannacha; Belqat et al. 2001a, **Rif**; Belqat et al. 2001b; Belqat and Dakki 2004, **Rif**; Belqat et al. 2005, **Rif**; Koçak and Kemal 2010; Belqat et al. 2011; Adler and Crosskey 2017; Belqat et al. 2018; Adler 2019

﻿***Urosimulium* Contini, 1963**

﻿*Urosimuliumfaurei* (Bernard, Grenier & Bailly-Choumara, 1972)

Grenier et al. 1957, **MA**; Bernard et al. 1972: 63–68 (original description), **MA**, Plateau de Talerhza (environ de Meknès); Clergue-Gazeau et al. 1991, **MA**; Hervy et al. 1994; Belqat and Adler 2001, **Rif**, Oued Iouchirene, Oued Mrinet, Oued Biyada, Oued Hannacha, Oued Ankouda; Belqat et al. 2001a, **Rif**, **MA**; Belqat 2002, **Rif**, **MA**; Belqat and Dakki 2004, **Rif**; Belqat et al. 2005, **Rif**; Dakki et al. 2008, **MA**, Oued Sebou; Belqat et al. 2011, **Rif**, **MA**; Adler and Crosskey 2017; Belqat et al. 2018; Adler 2019

##### Simuliini

﻿***Greniera* Doby & David, 1959**

﻿*Greniera ﻿﻿﻿fabri* Doby & David, 1959

Clergue-Gazeau et al. 1991, **MA**; Dakki 1997; Belqat et al. 2001a, **Rif**; Belqat 2002, **Rif**; Belqat and Dakki 2004, **Rif**; Belqat et al. 2005, **Rif**; Dakki et al. 2008, **MA**, Oued Sebou; Belqat et al. 2011, **Rif**, **MA**; Adler and Crosskey 2017; Belqat et al. 2018; Adler 2019

﻿***Metacnephia* Crosskey, 1969**

﻿*Metacnephia ﻿﻿﻿blanci* (Grenier & Théodoridès, 1953)

= *Cnephia* ﻿sp. in Grenier 1953: 157

= *Cnephia ﻿﻿﻿blanci* Grenier and Théodoridès, in Grenier and Théodoridès 1953: 430–435

= ﻿*Eusimulium ﻿﻿﻿latinum* Rubzov, in Benhoussa et al. 1988: 160–164

Grenier 1953, **HA**; Grenier and Théodoridès 1953, **HA**; Grenier et al. 1957, **MA**; Bailly-Choumara and Beaucournu-Saguez 1978, **Rif**; Bailly-Choumara and Beaucournu-Saguez 1981, **HA**; Benhoussa et al. 1988, **AP**, Oued Bou-Regreg; Clergue-Gazeau et al. 1991, **AA**; Dakki 1997; Giudicelli et al. 2000, **HA**, Oued Réghaya (Neltner, 3800 m), Oued Réghaya (Sidi Chamharouch, 2300 m), Oued Réghaya (lmlil, 1740 m), Oued Réghaya (Aguersioual, 1550 m), Oued Réghaya (Moulay Brahim, 1200 m), Oued Réghaya (Tahanaout, 890 m), ruisselet émissaire de source débouchant dans Oued Réghaya en amont d’lmlil (1750 m), ruisselet émissaire de source débouchant dans l’assif M’zik (1850 m), ruisselet émissaire de source débouchant dans l’assif N’Ouarzane (3000 m), ruisseau émissaire de source (assif N’Ouarzane: 3000 m), assif N’Ouarzane (Irhoulidene, 2800 m), ruisseau affluent en rive droite de l’assif N’Ouarzane (2400 m), Oued N’fis (amont Ijoukak, 1600 m), Oued N’fis (amont Wirgan, 1200 m), Oued N’fis (980 m), Oued N’fis (amont retenue Lalla Takerkoust, 660 m), ruisseau de Tinzart (émissaire de source: 2850 m), ruisseau de Tifni (émissaire de source: 2780 m), ruisseau de Likemt (émissaire de source: 2670 m), ruisseau de Tougroudadene (émissaire de source: 2660 m), assif Oukaimeden (2600 m), source hélocrène au niveau du cirque d’Oukaimeden (2660 m), assif Tiferguine (2500 m), assif Oukaimeden (2450 m), ruisseau émissaire de source débouchant dans l’assif Oukaimeden (1740 m), complexe rhéocrène formé par des émissaires de source débouchant dans l’assif Oukaimeden (1730 m), affluent temporaire en rive gauche de l’assif Oukaimeden (1630 m), affluent temporaire en rive gauche de l’assif Oukaimeden (1360 m), affluent temporaire en rive droite de l’assif Oukaimeden (1260 m), affluent en rive droite de l’assif Oukaimeden (1300 m), assif Tarzaza (1200 m), assif Tarzaza (1000 m), cours inférieur de l’oued Ourika (850 m), Oued Rdat en amont de Taddert (1850 m), affluent temporaire en rive gauche de Oued Rdat (1400 m), Oued Rdat (1600 m), Oued Rdat (1230 m), Oued Tensift (600–700 m), khetarras (450–600 m), Oued Tessaout au niveau d’Aït Tamli (1620 m), Oued Lakdar en aval de la retenue de Sidi Driss (1030 m), ruisseau émissaire de source formant le début de l’assif Imini (2090 m), assif Imini (1560 m), Oued Ounila (1820 m), ruisseau affluent en rive gauche de l’oued Ounila (1820 m), Oued Ounila (Timhlt, 1600 m), Oued Mellah (Anghessa, 1400 m), Oued Dadès en amont des gorges (1630 m), Oued Dadès (Boumalne, 1530 m), Oued Dadès (Sidi Flah, 1100 m), Oued M’goun (1530 m), Oued M’goun en aval de Kelaâ (1370 m), ruisseau émissaire de source débouchant dans un affluent de l’Oued Souss (2350 m); Belqat et al. 2001a, **Rif**, **MA**, **HA**, **AA**; Belqat 2002, **Rif**, **MA**, **HA**, **AA**; Belqat and Dakki 2004, **Rif**; Belqat et al. 2005, **Rif**; Belqat et al. 2008; Dakki et al. 2008, **MA**, Oued Sebou; Belqat et al. 2011, **Rif**, **AP**, **MA**, **HA**, **AA**; Adler and Crosskey 2017; Belqat et al. 2018; Adler 2019

﻿*Metacnephianuragica* Rivosecchi, Raastad & Contini, 1975^6^

= *Cnephia ﻿﻿﻿tredecimatum* (Edwards), in Grenier et al. 1957: 226

Grenier et al. 1957, **AP**, Coastal meseta (region of Rabat); Belqat et al. 2001a, **AP**, Rabat; Belqat 2002, **AP**, Rabat; Belqat and Dakki 2004, **AP**, Rabat; Belqat et al. 2011, **AP**; Belqat et al. 2018

﻿***Simulium* Latreille, 1802**

﻿*Simulium (Crosskeyellum) gracilipes* Edwards, 1921

Edwards 1921: 143 (original description), **MA**; Séguy 1925: 233, **MA**; Séguy 1930a, **MA**; Grenier 1953, **MA**; Crosskey 1964, **MA**, Fès; Grenier and Bailly-Choumara 1970: 96–102 (original description of subgenus ﻿*Crosskeyellum*, description of ﻿*gracilipes*), **MA**; Clergue-Gazeau et al. 1991, **MA**; Hervy et al. 1994; Dakki 1997; Belqat et al. 2001a, **MA**; Belqat 2002, **MA**; Belqat and Dakki 2004, **MA**; Belqat et al. 2011, **MA**; Adler and Crosskey 2017; Belqat et al. 2018; Adler 2019

﻿*Simulium (Eusimulium) ﻿angustipes* Edwards, 1915

Clergue-Gazeau et al. 1991, **MA**, **HA**; Dakki 1997; Belqat et al. 2001a, **MA**, **HA**; Belqat 2002, **MA**, **HA**; Belqat and Dakki 2004, **MA**, **HA**; Dakki et al. 2008, **MA**, O. Sebou; Koçak and Kemal 2010; Belqat et al. 2011, **MA**, **HA**; Adler and Crosskey 2017; Belqat et al. 2018; Adler 2019

﻿*Simulium (Eusimulium) ﻿mellah* Giudicelli & Bouzidi, 2000 [in Giudicelli, Bouzidi and Abdelaali 2000]

Giudicelli et al. 2000: 63 (original description), **HA**, Oued Mellah (Bassin Draa); Belqat et al. 2001a, **HA**; Belqat 2002, **MA**, **HA**; Belqat and Dakki 2004, **MA**, **HA**; Dakki et al. 2008, **MA**, Oued Sebou; Koçak and Kemal 2010; Belqat et al. 2011, **MA**, **HA**; Adler et al. 2015, **HA**; Adler and Crosskey 2017; Belqat et al. 2018; Adler 2019

﻿*Simulium (Eusimulium) ﻿petricolum* (Rivosecchi, 1963)

= ﻿*Simulium ﻿﻿﻿latizonum* Bailly-Choumara and Beaucournu-Saguez, in Bailly-Choumara and Beaucournu-Saguez 1978: 143–144 (misidentified); Bailly-Choumara and Beaucournu-Saguez 1981: 53–54

Bailly-Choumara and Beaucournu-Saguez 1978, **Rif**, **HA**; Bailly-Choumara and Beaucournu-Saguez 1981, **HA**; Clergue-Gazeau et al. 1991, **HA**; Dakki 1997; Giudicelli et al. 2000, **HA**, Oued Réghaya (Neltner, 3800 m), Oued Réghaya (Sidi Chamharouch, 2300 m), Oued Réghaya (lmlil, 1740 m), Oued Réghaya (Aguersioual, 1550 m), Oued Réghaya (Moulay Brahim, 1200 m), Oued Réghaya (Tahanaout, 890 m), ruisselet émissaire de source débouchant dans Oued Réghaya en amont d’lmlil (1750 m), ruisselet émissaire de source débouchant dans l’assif M’zik (1850 m), ruisselet émissaire de source débouchant dans l’assif N’Ouarzane (3000 m), ruisseau émissaire de source (assif N’Ouarzane, 3000 m), assif N’Ouarzane (Irhoulidene, 2800 m), ruisseau affluent en rive droite de l’assif N’Ouarzane (2400 m), Oued N’fis (amont Ijoukak, 1600 m), Oued N’fis (amont Wirgan, 1200 m), Oued N’fis (980 m), Oued N’fis (amont retenue Lalla Takerkoust, 660 m), ruisseau de Tinzart (émissaire de source: 2850 m), ruisseau de Tifni (émissaire de source: 2780 m), ruisseau de Likemt (émissaire de source: 2670 m), ruisseau de Tougroudadene (émissaire de source: 2660 m), assif Oukaimeden (2600 m), source hélocrène au niveau du cirque d’Oukaimeden (2660 m), assif Tiferguine (2500 m), assif Oukaimeden (2450 m), ruisseau émissaire de source débouchant dans l’assif Oukaimeden (1740 m), complexe rhéocrène formé par des émissaires de source débouchant dans l’assif Oukaimeden (1730 m), affluent temporaire en rive gauche de l’assif Oukaimeden (1630 m), affluent temporaire en rive gauche de l’assif Oukaimeden (1360 m), affluent temporaire en rive droite de l’assif Oukaimeden (1260 m), affluent en rive droite de l’assif Oukaimeden (1300 m), assif Tarzaza (1200 m), assif Tarzaza (1000 m), cours inférieur de l’oued Ourika (850 m), Oued Rdat en amont de Taddert (1850 m), affluent temporaire en rive gauche de Oued Rdat (1400 m), Oued Rdat (1600 m), Oued Rdat (1230 m), Oued Tensift (600–700 m), khetarras (450–600 m), Oued Tessaout au niveau d’Aït Tamli (1620 m), Oued Lakdar en aval de la retenue de Sidi Driss (1030 m), ruisseau émissaire de source formant le début de l’assif Imini (2090 m), assif Imini (1560 m), Oued Ounila (1820 m), ruisseau affluent en rive gauche de l’oued Ounila (1820 m), Oued Ounila (Timhlt, 1600 m), Oued Mellah (Anghessa, 1400 m), Oued Dadès en amont des gorges (1630 m), Oued Dadès (Boumalne, 1530 m), Oued Dadès (Sidi Flah, 1100 m), Oued M’goun (1530 m), Oued M’goun en aval de Kelaâ (1370 m), ruisseau émissaire de source débouchant dans un affluent de l’Oued Souss (2350 m); Belqat et al. 2001a, **Rif**, **HA**; Belqat 2002, **Rif**, **HA**; Belqat and Dakki 2004, **Rif**, **HA**; Belqat et al. 2005, **Rif**; Dakki et al. 2008, **Rif**, **MA**, Oued Sebou; Koçak and Kemal 2010; Belqat et al. 2011, **HA**; Adler et al. 2015, **Rif**, **HA**; Adler and Crosskey 2017; Belqat et al. 2018; Adler 2019

﻿*Simulium (Eusimulium) ﻿rubzovianum* (Sherban, 1961)

Adler et al. 2015, **Rif**, **HA**; Adler and Crosskey 2017; Belqat et al. 2018; Adler 2019

﻿*Simulium (Eusimulium) ﻿velutinumsensu stricto* (Santos Abreu, 1922)

= *Eusimilium﻿latinum* Rubzov, in El Mezdi and Giudicelli 1985: 292–295; Benhoussa et al. 1988: 160–164

Bailly-Choumara and Beaucournu-Saguez 1978, **Rif**; Bailly-Choumara and Beaucournu-Saguez 1981, **HA**; El Mezdi and Giudicelli 1985, **HA**, Khettaras of Marrakech; Benhoussa et al. 1988, **AP**, Oued Bou-Regreg; Clergue-Gazeau et al. 1991, **AA**; Benhoussa et al. 1993, **AP**, Oued Bou-Regreg; Dakki 1997; Giudicelli et al. 2000, **HA**, Oued Réghaya (Neltner, 3800 m), Oued Réghaya (Sidi Chamharouch, 2300 m), Oued Réghaya (lmlil, 1740 m), Oued Réghaya (Aguersioual, 1550 m), Oued Réghaya (Moulay Brahim, 1200 m), Oued Réghaya (Tahanaout, 890 m), ruisselet émissaire de source débouchant dans Oued Réghaya en amont d’lmlil (1750 m), ruisselet émissaire de source débouchant dans l’assif M’zik (1850 m), ruisselet émissaire de source débouchant dans l’assif N’Ouarzane (3000 m), ruisseau émissaire de source (assif N’Ouarzane, 3000 m), assif N’Ouarzane (Irhoulidene, 2800 m), ruisseau affluent en rive droite de l’assif N’Ouarzane (2400 m), Oued N’fis (amont Ijoukak, 1600 m), Oued N’fis (amont Wirgan, 1200 m), Oued N’fis (980 m), Oued N’fis (amont retenue Lalla Takerkoust, 660 m), ruisseau de Tinzart (émissaire de source: 2850 m), ruisseau de Tifni (émissaire de source: 2780 m), ruisseau de Likemt (émissaire de source: 2670 m), ruisseau de Tougroudadene (émissaire de source: 2660 m), assif Oukaimeden (2600 m), source hélocrène au niveau du cirque d’Oukaimeden (2660 m), assif Tiferguine (2500 m), assif Oukaimeden (2450 m), ruisseau émissaire de source débouchant dans l’assif Oukaimeden (1740 m), complexe rhéocrène formé par des émissaires de source débouchant dans l’assif Oukaimeden (1730 m), affluent temporaire en rive gauche de l’assif Oukaimeden (1630 m), affluent temporaire en rive gauche de l’assif Oukaimeden (1360 m), affluent temporaire en rive droite de l’assif Oukaimeden (1260 m), affluent en rive droite de l’assif Oukaimeden (1300 m), assif Tarzaza (1200 m), assif Tarzaza (1000 m), cours inférieur de l’oued Ourika (850 m), Oued Rdat en amont de Taddert (1850 m), affluent temporaire en rive gauche de Oued Rdat (1400 m), Oued Rdat (1600 m), Oued Rdat (1230 m), Oued Tensift (600–700 m), khetarras (450–600 m), Oued Tessaout au niveau d’Aït Tamli (1620 m), Oued Lakdar en aval de la retenue de Sidi Driss (1030 m), ruisseau émissaire de source formant le début de l’assif Imini (2090 m), assif Imini (1560 m), Oued Ounila (1820 m), ruisseau affluent en rive gauche de l’oued Ounila (1820 m), Oued Ounila (Timhlt, 1600 m), Oued Mellah (Anghessa, 1400 m), Oued Dadès en amont des gorges (1630 m), Oued Dadès (Boumalne, 1530 m), Oued Dadès (Sidi Flah, 1100 m), Oued M’goun (1530 m), Oued M’goun en aval de Kelaâ (1370 m), ruisseau émissaire de source débouchant dans un affluent de l’Oued Souss (2350 m); Belqat et al. 2001a, **Rif**, **MA**, **HA**, **AA**; Belqat 2002, **Rif**, **MA**, **HA**, **AA**; Belqat and Dakki 2004, **Rif**, **MA**, **HA**, **AA**; Belqat et al. 2005, **Rif**; Dakki et al. 2008, **MA**, Oued Sebou; Koçak and Kemal 2010; Belqat et al. 2011, **Rif**, **AP**, **MA**, **HA**, **AA**; Adler et al. 2015, **Rif**, **HA**; Adler and Crosskey 2017; Belqat et al. 2018; Adler 2019

﻿*Simulium (Eusimulium) ﻿velutinum* cytospecies ‘5’

Adler et al. 2015, **Rif**, Tanger-Anjra, **HA**, Marrakech; Belqat et al. 2018

﻿*Simulium (Nevermannia) ﻿ruficorne* species group

﻿*Simulium (Nevermannia) angustitarse* (Lundström, 1911)

Belqat et al. 2001a, **Rif**; Belqat et al. 2001b, **Rif**; Belqat 2002, **Rif**; Belqat and Dakki 2004, **Rif**; Belqat et al. 2005, **Rif**; Belqat et al. 2011, **Rif**; Adler and Crosskey 2017; Belqat et al. 2018; Adler 2019

﻿*Simulium (Nevermannia) ﻿ibleum* (Rivosecchi, 1966)

Clergue-Gazeau et al. 1991, **HA**; Dakki 1997; Giudicelli et al. 2000, **HA**, Oued Réghaya (Neltner, 3800 m), Oued Réghaya (Sidi Chamharouch, 2300 m), Oued Réghaya (lmlil, 1740 m), Oued Réghaya (Aguersioual, 1550 m), Oued Réghaya (Moulay Brahim, 1200 m), Oued Réghaya (Tahanaout, 890 m), ruisselet émissaire de source débouchant dans Oued Réghaya en amont d’lmlil (1750 m), ruisselet émissaire de source débouchant dans l’assif M’zik (1850 m), ruisselet émissaire de source débouchant dans l’assif N’Ouarzane (3000 m), ruisseau émissaire de source (assif N’Ouarzane, 3000 m), assif N’Ouarzane (Irhoulidene, 2800 m), ruisseau affluent en rive droite de l’assif N’Ouarzane (2400 m), Oued N’fis (amont Ijoukak, 1600 m), Oued N’fis (amont Wirgan, 1200 m), Oued N’fis (980 m), Oued N’fis (amont retenue Lalla Takerkoust, 660 m), ruisseau de Tinzart (émissaire de source: 2850 m), ruisseau de Tifni (émissaire de source: 2780 m), ruisseau de Likemt (émissaire de source: 2670 m), ruisseau de Tougroudadene (émissaire de source: 2660 m), assif Oukaimeden (2600 m), source hélocrène au niveau du cirque d’Oukaimeden (2660 m), assif Tiferguine (2500 m), assif Oukaimeden (2450 m), ruisseau émissaire de source débouchant dans l’assif Oukaimeden (1740 m), complexe rhéocrène formé par des émissaires de source débouchant dans l’assif Oukaimeden (1730 m), affluent temporaire en rive gauche de l’assif Oukaimeden (1630 m), affluent temporaire en rive gauche de l’assif Oukaimeden (1360 m), affluent temporaire en rive droite de l’assif Oukaimeden (1260 m), affluent en rive droite de l’assif Oukaimeden (1300 m), assif Tarzaza (1200 m), assif Tarzaza (1000 m), cours inférieur de l’oued Ourika (850 m), Oued Rdat en amont de Taddert (1850 m), affluent temporaire en rive gauche de Oued Rdat (1400 m), Oued Rdat (1600 m), Oued Rdat (1230 m), Oued Tensift (600–700 m), khetarras (450–600 m), Oued Tessaout au niveau d’Aït Tamli (1620 m), Oued Lakdar en aval de la retenue de Sidi Driss (1030 m), ruisseau émissaire de source formant le début de l’assif Imini (2090 m), assif Imini (1560 m), Oued Ounila (1820 m), ruisseau affluent en rive gauche de l’oued Ounila (1820 m), Oued Ounila (Timhlt, 1600 m), Oued Mellah (Anghessa, 1400 m), Oued Dadès en amont des gorges (1630 m), Oued Dadès (Boumalne, 1530 m), Oued Dadès (Sidi Flah, 1100 m), Oued M’goun (1530 m), Oued M’goun en aval de Kelaâ (1370 m), ruisseau émissaire de source débouchant dans un affluent de l’Oued Souss (2350 m); Belqat et al. 2001a, **Rif**, **HA**; Belqat 2002, **Rif**, **HA**; Belqat and Dakki 2004, **Rif**, **HA**; Belqat et al. 2005, **Rif**; Belqat et al. 2011, **Rif**, **HA**; Adler and Crosskey 2017; Belqat et al. 2018; Adler 2019

﻿*Simulium (Nevermannia) ﻿lundstromi* (Enderlein, 1921)

Clergue-Gazeau et al. 1991, **HA**; Dakki 1997; Giudicelli et al. 2000, **HA**; Belqat et al. 2001a, **HA**; Belqat 2002, **HA**; Belqat and Dakki 2004, **HA**; Belqat et al. 2011, **Rif**, Kanar (280 m), Majjo (905 m), 10 km before the Issaguen source (1200 m), **HA**; Adler and Crosskey 2017; Belqat et al. 2018; Adler 2019

﻿*Simulium (Nevermannia) ﻿ruficorne* Macquart, 1838

= ﻿*Eusimulium ﻿﻿﻿ruficorne* Macquart, in El Mezdi and Giudicelli 1985: 292, 294–295

Grenier et al. 1957, **AA**; Bailly-Choumara and Beaucournu-Saguez 1978, **Rif**; Bailly-Choumara and Beaucournu-Saguez 1981, **HA**; El Mezdi and Giudicelli 1985, **HA**, Khettaras of Marrakech; Clergue-Gazeau et al. 1991, **HA**; Dakki 1997; Giudicelli et al. 2000, **HA**, Oued Réghaya (Neltner, 3800 m), Oued Réghaya (Sidi Chamharouch, 2300 m), Oued Réghaya (lmlil, 1740 m), Oued Réghaya (Aguersioual, 1550 m), Oued Réghaya (Moulay Brahim, 1200 m), Oued Réghaya (Tahanaout, 890 m), ruisselet émissaire de source débouchant dans Oued Réghaya en amont d’lmlil (1750 m), ruisselet émissaire de source débouchant dans l’assif M’zik (1850 m), ruisselet émissaire de source débouchant dans l’assif N’Ouarzane (3000 m), ruisseau émissaire de source (assif N’Ouarzane, 3000 m), assif N’Ouarzane (Irhoulidene, 2800 m), ruisseau affluent en rive droite de l’assif N’Ouarzane (2400 m), Oued N’fis (amont Ijoukak, 1600 m), Oued N’fis (amont Wirgan, 1200 m), Oued N’fis (980 m), Oued N’fis (amont retenue Lalla Takerkoust, 660 m), ruisseau de Tinzart (émissaire de source: 2850 m), ruisseau de Tifni (émissaire de source: 2780 m), ruisseau de Likemt (émissaire de source: 2670 m), ruisseau de Tougroudadene (émissaire de source: 2660 m), assif Oukaimeden (2600 m), source hélocrène au niveau du cirque d’Oukaimeden (2660 m), assif Tiferguine (2500 m), assif Oukaimeden (2450 m), ruisseau émissaire de source débouchant dans l’assif Oukaimeden (1740 m), complexe rhéocrène formé par des émissaires de source débouchant dans l’assif Oukaimeden (1730 m), affluent temporaire en rive gauche de l’assif Oukaimeden (1630 m), affluent temporaire en rive gauche de l’assif Oukaimeden (1360 m), affluent temporaire en rive droite de l’assif Oukaimeden (1260 m), affluent en rive droite de l’assif Oukaimeden (1300 m), assif Tarzaza (1200 m), assif Tarzaza (1000 m), cours inférieur de l’oued Ourika (850 m), Oued Rdat en amont de Taddert (1850 m), affluent temporaire en rive gauche de Oued Rdat (1400 m), Oued Rdat (1600 m), Oued Rdat (1230 m), Oued Tensift (600–700 m), khetarras (450–600 m), Oued Tessaout au niveau d’Aït Tamli (1620 m), Oued Lakdar en aval de la retenue de Sidi Driss (1030 m), ruisseau émissaire de source formant le début de l’assif Imini (2090 m), assif Imini (1560 m), Oued Ounila (1820 m), ruisseau affluent en rive gauche de l’oued Ounila (1820 m), Oued Ounila (Timhlt, 1600 m), Oued Mellah (Anghessa, 1400 m), Oued Dadès en amont des gorges (1630 m), Oued Dadès (Boumalne, 1530 m), Oued Dadès (Sidi Flah, 1100 m), Oued M’goun (1530 m), Oued M’goun en aval de Kelaâ (1370 m), ruisseau émissaire de source débouchant dans un affluent de l’Oued Souss (2350 m); Belqat et al. 2001a, **Rif**, **HA**, **AA**; Belqat 2002, **Rif**, **HA**, **AA**; Crosskey et al. 2002; Belqat and Dakki 2004, **Rif**, **HA**, **AA**; Belqat et al. 2005, **Rif**; Belqat et al. 2011, **Rif**, **AP**, **HA**, **AA**; Adler and Crosskey 2017; Belqat et al. 2018; Adler 2019

﻿*Simulium (Nevermannia) ﻿vernum* species group

﻿*Simulium (Nevermannia) ﻿brevidens* (Rubtsov, 1956)

Clergue-Gazeau et al. 1991, **HA**; Giudicelli et al. 2000, **HA**, Oued Réghaya (Neltner, 3800 m), Oued Réghaya (Sidi Chamharouch, 2300 m), Oued Réghaya (lmlil, 1740 m), Oued Réghaya (Aguersioual, 1550 m), Oued Réghaya (Moulay Brahim, 1200 m), Oued Réghaya (Tahanaout, 890 m), ruisselet émissaire de source débouchant dans Oued Réghaya en amont d’lmlil (1750 m), ruisselet émissaire de source débouchant dans l’assif M’zik (1850 m), ruisselet émissaire de source débouchant dans l’assif N’Ouarzane (3000 m), ruisseau émissaire de source (assif N’Ouarzane, 3000 m), assif N’Ouarzane (Irhoulidene, 2800 m), ruisseau affluent en rive droite de l’assif N’Ouarzane (2400 m), Oued N’fis (amont Ijoukak, 1600 m), Oued N’fis (amont Wirgan, 1200 m), Oued N’fis (980 m), Oued N’fis (amont retenue Lalla Takerkoust, 660 m), ruisseau de Tinzart (émissaire de source: 2850 m), ruisseau de Tifni (émissaire de source: 2780 m), ruisseau de Likemt (émissaire de source: 2670 m), ruisseau de Tougroudadene (émissaire de source: 2660 m), assif Oukaimeden (2600 m), source hélocrène au niveau du cirque d’Oukaimeden (2660 m), assif Tiferguine (2500 m), assif Oukaimeden (2450 m), ruisseau émissaire de source débouchant dans l’assif Oukaimeden (1740 m), complexe rhéocrène formé par des émissaires de source débouchant dans l’assif Oukaimeden (1730 m), affluent temporaire en rive gauche de l’assif Oukaimeden (1630 m), affluent temporaire en rive gauche de l’assif Oukaimeden (1360 m), affluent temporaire en rive droite de l’assif Oukaimeden (1260 m), affluent en rive droite de l’assif Oukaimeden (1300 m), assif Tarzaza (1200 m), assif Tarzaza (1000 m), cours inférieur de l’oued Ourika (850 m), Oued Rdat en amont de Taddert (1850 m), affluent temporaire en rive gauche de Oued Rdat (1400 m), Oued Rdat (1600 m), Oued Rdat (1230 m), Oued Tensift (600–700 m), khetarras (450–600 m), Oued Tessaout au niveau d’Aït Tamli (1620 m), Oued Lakdar en aval de la retenue de Sidi Driss (1030 m), ruisseau émissaire de source formant le début de l’assif Imini (2090 m), assif Imini (1560 m), Oued Ounila (1820 m), ruisseau affluent en rive gauche de l’oued Ounila (1820 m), Oued Ounila (Timhlt, 1600 m), Oued Mellah (Anghessa, 1400 m), Oued Dadès en amont des gorges (1630 m), Oued Dadès (Boumalne, 1530 m), Oued Dadès (Sidi Flah, 1100 m), Oued M’goun (1530 m), Oued M’goun en aval de Kelaâ (1370 m), ruisseau émissaire de source débouchant dans un affluent de l’Oued Souss (2350 m); Belqat et al. 2001a, **HA**; Belqat 2002, **HA**; Belqat and Dakki 2004, **HA**; Belqat et al. 2011, **HA**; Belqat et al. 2018; Adler 2019

﻿*Simulium (Nevermannia) ﻿carthusiense* (Grenier & Dorier, 1959)

Giudicelli and Dakki 1984, **Rif**; Dakki 1997; Belqat et al. 2001a, **Rif**; Belqat 2002, **Rif**; Belqat and Dakki 2004, **Rif**; Belqat et al. 2005, **Rif**; Belqat et al. 2008, **Rif**; Belqat et al. 2011, **Rif**; Adler and Crosskey 2017; Belqat et al. 2018; Adler 2019

﻿*Simulium (Nevermannia) ﻿costatum* Friederichs, 1920

Grenier et al. 1957, **Rif**, Pré-Rif, **MA**; Bailly-Choumara and Beaucournu-Saguez 1981, **HA**; Giudicelli and Bouzidi 1989, **HA**; Clergue-Gazeau et al. 1991, **HA**; Dakki 1997; Giudicelli et al. 2000, **HA**, Oued Réghaya (Neltner, 3800 m), Oued Réghaya (Sidi Chamharouch, 2300 m), Oued Réghaya (lmlil, 1740 m), Oued Réghaya (Aguersioual, 1550 m), Oued Réghaya (Moulay Brahim, 1200 m), Oued Réghaya (Tahanaout, 890 m), ruisselet émissaire de source débouchant dans Oued Réghaya en amont d’lmlil (1750 m), ruisselet émissaire de source débouchant dans l’assif M’zik (1850 m), ruisselet émissaire de source débouchant dans l’assif N’Ouarzane (3000 m), ruisseau émissaire de source (assif N’Ouarzane, 3000 m), assif N’Ouarzane (Irhoulidene, 2800 m), ruisseau affluent en rive droite de l’assif N’Ouarzane (2400 m), Oued N’fis (amont Ijoukak, 1600 m), Oued N’fis (amont Wirgan, 1200 m), Oued N’fis (980 m), Oued N’fis (amont retenue Lalla Takerkoust, 660 m), ruisseau de Tinzart (émissaire de source: 2850 m), ruisseau de Tifni (émissaire de source: 2780 m), ruisseau de Likemt (émissaire de source: 2670 m), ruisseau de Tougroudadene (émissaire de source: 2660 m), assif Oukaimeden (2600 m), source hélocrène au niveau du cirque d’Oukaimeden (2660 m), assif Tiferguine (2500 m), assif Oukaimeden (2450 m), ruisseau émissaire de source débouchant dans l’assif Oukaimeden (1740 m), complexe rhéocrène formé par des émissaires de source débouchant dans l’assif Oukaimeden (1730 m), affluent temporaire en rive gauche de l’assif Oukaimeden (1630 m), affluent temporaire en rive gauche de l’assif Oukaimeden (1360 m), affluent temporaire en rive droite de l’assif Oukaimeden (1260 m), affluent en rive droite de l’assif Oukaimeden (1300 m), assif Tarzaza (1200 m), assif Tarzaza (1000 m), cours inférieur de l’oued Ourika (850 m), Oued Rdat en amont de Taddert (1850 m), affluent temporaire en rive gauche de Oued Rdat (1400 m), Oued Rdat (1600 m), Oued Rdat (1230 m), Oued Tensift (600–700 m), khetarras (450–600 m), Oued Tessaout au niveau d’Aït Tamli (1620 m), Oued Lakdar en aval de la retenue de Sidi Driss (1030 m), ruisseau émissaire de source formant le début de l’assif Imini (2090 m), assif Imini (1560 m), Oued Ounila (1820 m), ruisseau affluent en rive gauche de l’oued Ounila (1820 m), Oued Ounila (Timhlt, 1600 m), Oued Mellah (Anghessa, 1400 m), Oued Dadès en amont des gorges (1630 m), Oued Dadès (Boumalne, 1530 m), Oued Dadès (Sidi Flah, 1100 m), Oued M’goun (1530 m), Oued M’goun en aval de Kelaâ (1370 m), ruisseau émissaire de source débouchant dans un affluent de l’Oued Souss (2350 m); Belqat et al. 2001a, **Rif**, **MA**, **HA**; Belqat 2002, **Rif**, **MA**, **HA**; Belqat and Dakki 2004, **Rif**, **MA**, **HA**; Belqat et al. 2005, **Rif**; Belqat et al. 2008, **Rif**; Belqat et al. 2011, **Rif**, **MA**, **HA**; Adler and Crosskey 2017; Belqat et al. 2018; Adler 2019

﻿*Simulium (Nevermannia) ﻿cryophilum* (Rubtsov, 1959) (complex)

= ﻿*Simulium ﻿﻿﻿pusillum* Fries, in Séguy 1930a: 52 (misidentification); Grenier 1953: 159 (after Séguy)

Séguy 1930a, **HA**; Grenier 1953, **Rif**, **HA**, Lac Ifni; Bouzidi and Giudicelli 1986, **HA**; Bouzidi and Giudicelli 1989, **HA**; Clergue-Gazeau et al. 1991, **HA**; Giudicelli et al. 2000, **HA**, Oued Réghaya (Neltner, 3800 m), Oued Réghaya (Sidi Chamharouch, 2300 m), Oued Réghaya (lmlil, 1740 m), Oued Réghaya (Aguersioual, 1550 m), Oued Réghaya (Moulay Brahim, 1200 m), Oued Réghaya (Tahanaout, 890 m), ruisselet émissaire de source débouchant dans Oued Réghaya en amont d’lmlil (1750 m), ruisselet émissaire de source débouchant dans l’assif M’zik (1850 m), ruisselet émissaire de source débouchant dans l’assif N’Ouarzane (3000 m), ruisseau émissaire de source (assif N’Ouarzane, 3000 m), assif N’Ouarzane (Irhoulidene, 2800 m), ruisseau affluent en rive droite de l’assif N’Ouarzane (2400 m), Oued N’fis (amont Ijoukak, 1600 m), Oued N’fis (amont Wirgan, 1200 m), Oued N’fis (980 m), Oued N’fis (amont retenue Lalla Takerkoust, 660 m), ruisseau de Tinzart (émissaire de source: 2850 m), ruisseau de Tifni (émissaire de source: 2780 m), ruisseau de Likemt (émissaire de source: 2670 m), ruisseau de Tougroudadene (émissaire de source: 2660 m), assif Oukaimeden (2600 m), source hélocrène au niveau du cirque d’Oukaimeden (2660 m), assif Tiferguine (2500 m), assif Oukaimeden (2450 m), ruisseau émissaire de source débouchant dans l’assif Oukaimeden (1740 m), complexe rhéocrène formé par des émissaires de source débouchant dans l’assif Oukaimeden (1730 m), affluent temporaire en rive gauche de l’assif Oukaimeden (1630 m), affluent temporaire en rive gauche de l’assif Oukaimeden (1360 m), affluent temporaire en rive droite de l’assif Oukaimeden (1260 m), affluent en rive droite de l’assif Oukaimeden (1300 m), assif Tarzaza (1200 m), assif Tarzaza (1000 m), cours inférieur de l’oued Ourika (850 m), Oued Rdat en amont de Taddert (1850 m), affluent temporaire en rive gauche de Oued Rdat (1400 m), Oued Rdat (1600 m), Oued Rdat (1230 m), Oued Tensift (600–700 m), khetarras (450–600 m), Oued Tessaout au niveau d’Aït Tamli (1620 m), Oued Lakdar en aval de la retenue de Sidi Driss (1030 m), ruisseau émissaire de source formant le début de l’assif Imini (2090 m), assif Imini (1560 m), Oued Ounila (1820 m), ruisseau affluent en rive gauche de l’oued Ounila (1820 m), Oued Ounila (Timhlt, 1600 m), Oued Mellah (Anghessa, 1400 m), Oued Dadès en amont des gorges (1630 m), Oued Dadès (Boumalne, 1530 m), Oued Dadès (Sidi Flah, 1100 m), Oued M’goun (1530 m), Oued M’goun en aval de Kelaâ (1370 m), ruisseau émissaire de source débouchant dans un affluent de l’Oued Souss (2350 m); Giudicelli Belqat et al. 2001a, **Rif**, **HA**; Belqat 2002, **Rif**, **HA**; Belqat and Dakki 2004, **Rif**, **HA**; Belqat et al. 2005, **Rif**; Belqat et al. 2008, **Rif**; Belqat et al. 2011; Adler and Crosskey 2017; Belqat et al. 2018; Adler 2019

﻿*Simulium (Nevermannia) toubkal* (Bouzidi & Giudicelli, 1986)

Bouzidi and Giudicelli 1986: 41–52 (original description), **HA**, assif n’Ouarzane (Oued Nfis); Giudicelli and Bouzidi 1989, **HA**; Clergue-Gazeau et al. 1991, **HA**; Dakki 1997; Giudicelli et al. 2000, **HA**, Oued Réghaya (Neltner, 3800 m), Oued Réghaya (Sidi Chamharouch, 2300 m), Oued Réghaya (lmlil, 1740 m), Oued Réghaya (Aguersioual, 1550 m), Oued Réghaya (Moulay Brahim, 1200 m), Oued Réghaya (Tahanaout, 890 m), ruisselet émissaire de source débouchant dans Oued Réghaya en amont d’lmlil (1750 m), ruisselet émissaire de source débouchant dans l’assif M’zik (1850 m), ruisselet émissaire de source débouchant dans l’assif N’Ouarzane (3000 m), ruisseau émissaire de source (assif N’Ouarzane, 3000 m), assif N’Ouarzane (Irhoulidene, 2800 m), ruisseau affluent en rive droite de l’assif N’Ouarzane (2400 m), Oued N’fis (amont Ijoukak, 1600 m), Oued N’fis (amont Wirgan, 1200 m), Oued N’fis (980 m), Oued N’fis (amont retenue Lalla Takerkoust, 660 m), ruisseau de Tinzart (émissaire de source: 2850 m), ruisseau de Tifni (émissaire de source: 2780 m), ruisseau de Likemt (émissaire de source: 2670 m), ruisseau de Tougroudadene (émissaire de source: 2660 m), assif Oukaimeden (2600 m), source hélocrène au niveau du cirque d’Oukaimeden (2660 m), assif Tiferguine (2500 m), assif Oukaimeden (2450 m), ruisseau émissaire de source débouchant dans l’assif Oukaimeden (1740 m), complexe rhéocrène formé par des émissaires de source débouchant dans l’assif Oukaimeden (1730 m), affluent temporaire en rive gauche de l’assif Oukaimeden (1630 m), affluent temporaire en rive gauche de l’assif Oukaimeden (1360 m), affluent temporaire en rive droite de l’assif Oukaimeden (1260 m), affluent en rive droite de l’assif Oukaimeden (1300 m), assif Tarzaza (1200 m), assif Tarzaza (1000 m), cours inférieur de l’oued Ourika (850 m), Oued Rdat en amont de Taddert (1850 m), affluent temporaire en rive gauche de Oued Rdat (1400 m), Oued Rdat (1600 m), Oued Rdat (1230 m), Oued Tensift (600–700 m), khetarras (450–600 m), Oued Tessaout au niveau d’Aït Tamli (1620 m), Oued Lakdar en aval de la retenue de Sidi Driss (1030 m), ruisseau émissaire de source formant le début de l’assif Imini (2090 m), assif Imini (1560 m), Oued Ounila (1820 m), ruisseau affluent en rive gauche de l’oued Ounila (1820 m), Oued Ounila (Timhlt, 1600 m), Oued Mellah (Anghessa, 1400 m), Oued Dadès en amont des gorges (1630 m), Oued Dadès (Boumalne, 1530 m), Oued Dadès (Sidi Flah, 1100 m), Oued M’goun (1530 m), Oued M’goun en aval de Kelaâ (1370 m), ruisseau émissaire de source débouchant dans un affluent de l’Oued Souss (2350 m); Belqat et al. 2001a, **HA**; Belqat 2002, **HA**; Belqat and Dakki 2004, **HA**; Belqat et al. 2011, **HA**; Adler and Crosskey 2017; Belqat et al. 2018; Adler 2019

﻿*Simulium (Nevermannia) ﻿vernum* Macquart, 1826 (complex) [*latipes* authors pre-1972, not Meigen]

Clergue-Gazeau et al. 1991, **HA**; Dakki 1997; Belqat et al. 2001a, **Rif**, **HA**; Belqat 2002, **Rif**; Belqat and Dakki 2004, **Rif**, **HA**; Belqat et al. 2005, **Rif**; Belqat et al. 2011, **Rif**, **HA**; Adler and Crosskey 2017; Belqat et al. 2018; Adler 2019

﻿*Simulium (Rubzovia) ﻿knidirii* (Giudicelli & Thiery, 1985)

Giudicelli and Thiery 1985: 109–123 (original description in new subgenus ﻿Simulium (Crenosimulium), **HA**; Clergue-Gazeau et al. 1991, **HA**; Dakki 1997; Giudicelli et al. 2000, **HA**, Oued Réghaya (Neltner, 3800 m), Oued Réghaya (Sidi Chamharouch, 2300 m), Oued Réghaya (lmlil, 1740 m), Oued Réghaya (Aguersioual, 1550 m), Oued Réghaya (Moulay Brahim, 1200 m), Oued Réghaya (Tahanaout, 890 m), ruisselet émissaire de source débouchant dans Oued Réghaya en amont d’lmlil (1750 m), ruisselet émissaire de source débouchant dans l’assif M’zik (1850 m), ruisselet émissaire de source débouchant dans l’assif N’Ouarzane (3000 m), ruisseau émissaire de source (assif N’Ouarzane, 3000 m), assif N’Ouarzane (Irhoulidene, 2800 m), ruisseau affluent en rive droite de l’assif N’Ouarzane (2400 m), Oued N’fis (amont Ijoukak, 1600 m), Oued N’fis (amont Wirgan, 1200 m), Oued N’fis (980 m), Oued N’fis (amont retenue Lalla Takerkoust, 660 m), ruisseau de Tinzart (émissaire de source: 2850 m), ruisseau de Tifni (émissaire de source: 2780 m), ruisseau de Likemt (émissaire de source: 2670 m), ruisseau de Tougroudadene (émissaire de source: 2660 m), assif Oukaimeden (2600 m), source hélocrène au niveau du cirque d’Oukaimeden (2660 m), assif Tiferguine (2500 m), assif Oukaimeden (2450 m), ruisseau émissaire de source débouchant dans l’assif Oukaimeden (1740 m), complexe rhéocrène formé par des émissaires de source débouchant dans l’assif Oukaimeden (1730 m), affluent temporaire en rive gauche de l’assif Oukaimeden (1630 m), affluent temporaire en rive gauche de l’assif Oukaimeden (1360 m), affluent temporaire en rive droite de l’assif Oukaimeden (1260 m), affluent en rive droite de l’assif Oukaimeden (1300 m), assif Tarzaza (1200 m), assif Tarzaza (1000 m), cours inférieur de l’oued Ourika (850 m), Oued Rdat en amont de Taddert (1850 m), affluent temporaire en rive gauche de Oued Rdat (1400 m), Oued Rdat (1600 m), Oued Rdat (1230 m), Oued Tensift (600–700 m), khetarras (450–600 m), Oued Tessaout au niveau d’Aït Tamli (1620 m), Oued Lakdar en aval de la retenue de Sidi Driss (1030 m), ruisseau émissaire de source formant le début de l’assif Imini (2090 m), assif Imini (1560 m), Oued Ounila (1820 m), ruisseau affluent en rive gauche de l’oued Ounila (1820 m), Oued Ounila (Timhlt, 1600 m), Oued Mellah (Anghessa, 1400 m), Oued Dadès en amont des gorges (1630 m), Oued Dadès (Boumalne, 1530 m), Oued Dadès (Sidi Flah, 1100 m), Oued M’goun (1530 m), Oued M’goun en aval de Kelaâ (1370 m), ruisseau émissaire de source débouchant dans un affluent de l’Oued Souss (2350 m); Belqat et al. 2001a, **HA**; Belqat 2002, **HA**; Belqat and Dakki 2004, **HA**; Belqat et al. 2011, **HA**; Adler and Crosskey 2017; Belqat et al. 2018; Adler 2019

﻿*Simulium (Rubzovia) ﻿lamachi* (Doby & David, 1960)

Giudicelli and Dakki 1984, **Rif**; Giudicelli and Thiery 1985, **Rif**; Clergue-Gazeau et al. 1991, **HA**; Dakki 1997; Giudicelli et al. 2000, **HA**, Oued Réghaya (Neltner, 3800 m), Oued Réghaya (Sidi Chamharouch, 2300 m), Oued Réghaya (lmlil, 1740 m), Oued Réghaya (Aguersioual, 1550 m), Oued Réghaya (Moulay Brahim, 1200 m), Oued Réghaya (Tahanaout, 890 m), ruisselet émissaire de source débouchant dans Oued Réghaya en amont d’lmlil (1750 m), ruisselet émissaire de source débouchant dans l’assif M’zik (1850 m), ruisselet émissaire de source débouchant dans l’assif N’Ouarzane (3000 m), ruisseau émissaire de source (assif N’Ouarzane: 3000 m), assif N’Ouarzane (Irhoulidene, 2800 m), ruisseau affluent en rive droite de l’assif N’Ouarzane (2400 m), Oued N’fis (amont Ijoukak, 1600 m), Oued N’fis (amont Wirgan, 1200 m), Oued N’fis (980 m), Oued N’fis (amont retenue Lalla Takerkoust, 660 m), ruisseau de Tinzart (émissaire de source: 2850 m), ruisseau de Tifni (émissaire de source: 2780 m), ruisseau de Likemt (émissaire de source: 2670 m), ruisseau de Tougroudadene (émissaire de source: 2660 m), assif Oukaimeden (2600 m), source hélocrène au niveau du cirque d’Oukaimeden (2660 m), assif Tiferguine (2500 m), assif Oukaimeden (2450 m), ruisseau émissaire de source débouchant dans l’assif Oukaimeden (1740 m), complexe rhéocrène formé par des émissaires de source débouchant dans l’assif Oukaimeden (1730 m), affluent temporaire en rive gauche de l’assif Oukaimeden (1630 m), affluent temporaire en rive gauche de l’assif Oukaimeden (1360 m), affluent temporaire en rive droite de l’assif Oukaimeden (1260 m), affluent en rive droite de l’assif Oukaimeden (1300 m), assif Tarzaza (1200 m), assif Tarzaza (1000 m), cours inférieur de l’oued Ourika (850 m), Oued Rdat en amont de Taddert (1850 m), affluent temporaire en rive gauche de Oued Rdat (1400 m), Oued Rdat (1600 m), Oued Rdat (1230 m), Oued Tensift (600–700 m), khetarras (450–600 m), Oued Tessaout au niveau d’Aït Tamli (1620 m), Oued Lakdar en aval de la retenue de Sidi Driss (1030 m), ruisseau émissaire de source formant le début de l’assif Imini (2090 m), assif Imini (1560 m), Oued Ounila (1820 m), ruisseau affluent en rive gauche de l’oued Ounila (1820 m), Oued Ounila (Timhlt, 1600 m), Oued Mellah (Anghessa, 1400 m), Oued Dadès en amont des gorges (1630 m), Oued Dadès (Boumalne, 1530 m), Oued Dadès (Sidi Flah, 1100 m), Oued M’goun (1530 m), Oued M’Goun en aval de Kelaâ (1370 m), ruisseau émissaire de source débouchant dans un affluent de l’Oued Souss (2350 m); Belqat et al. 2001a, **Rif**, **HA**; Belqat 2002, **Rif**, **HA**; Belqat and Dakki 2004, **Rif**; Belqat et al. 2005, **Rif**; Belqat et al. 2011, **Rif**, **HA**; Adler and Crosskey 2017; Belqat et al. 2018; Adler 2019

﻿*Simulium (Simulium) ﻿bezzii* species group

﻿*Simulium (Simulium) ﻿bezzii* (Corti, 1914) (complex)

= ﻿*Simulium ﻿﻿﻿atlas* Séguy, 1930, in Séguy 1930a: 50 (original description); Grenier 1953: 158 (synonymy of ﻿*atlas* Séguy with ﻿*bezzii* suggested)

Séguy 1930a, **MA**; Grenier 1953; Grenier 1953, **MA**, **HA**; Grenier and Théodoridès 1953; Grenier et al. 1957, **AA**; Bailly-Choumara and Beaucournu-Saguez 1978, **Rif**; Bailly-Choumara and Beaucournu-Saguez 1981, **HA**; Bouzidi and Giudicelli 1986, **HA**; Clergue-Gazeau et al. 1991, **HA**; Dakki 1997; Giudicelli et al. 2000, **HA**, Oued Réghaya (Neltner, 3800 m), Oued Réghaya (Sidi Chamharouch, 2300 m), Oued Réghaya (lmlil, 1740 m), Oued Réghaya (Aguersioual, 1550 m), Oued Réghaya (Moulay Brahim, 1200 m), Oued Réghaya (Tahanaout, 890 m), ruisselet émissaire de source débouchant dans Oued Réghaya en amont d’lmlil (1750 m), ruisselet émissaire de source débouchant dans l’assif M’zik (1850 m), ruisselet émissaire de source débouchant dans l’assif N’Ouarzane (3000 m), ruisseau émissaire de source (assif N’Ouarzane, 3000 m), assif N’Ouarzane (Irhoulidene, 2800 m), ruisseau affluent en rive droite de l’assif N’Ouarzane (2400 m), Oued N’fis (amont Ijoukak, 1600 m), Oued N’fis (amont Wirgan, 1200 m), Oued N’fis (980 m), Oued N’fis (amont retenue Lalla Takerkoust, 660 m), ruisseau de Tinzart (émissaire de source: 2850 m), ruisseau de Tifni (émissaire de source: 2780 m), ruisseau de Likemt (émissaire de source: 2670 m), ruisseau de Tougroudadene (émissaire de source: 2660 m), assif Oukaimeden (2600 m), source hélocrène au niveau du cirque d’Oukaimeden (2660 m), assif Tiferguine (2500 m), assif Oukaimeden (2450 m), ruisseau émissaire de source débouchant dans l’assif Oukaimeden (1740 m), complexe rhéocrène formé par des émissaires de source débouchant dans l’assif Oukaimeden (1730 m), affluent temporaire en rive gauche de l’assif Oukaimeden (1630 m), affluent temporaire en rive gauche de l’assif Oukaimeden (1360 m), affluent temporaire en rive droite de l’assif Oukaimeden (1260 m), affluent en rive droite de l’assif Oukaimeden (1300 m), assif Tarzaza (1200 m), assif Tarzaza (1000 m), cours inférieur de l’oued Ourika (850 m), Oued Rdat en amont de Taddert (1850 m), affluent temporaire en rive gauche de Oued Rdat (1400 m), Oued Rdat (1600 m), Oued Rdat (1230 m), Oued Tensift (600–700 m), khetarras (450–600 m), Oued Tessaout au niveau d’Aït Tamli (1620 m), Oued Lakdar en aval de la retenue de Sidi Driss (1030 m), ruisseau émissaire de source formant le début de l’assif Imini (2090 m), assif Imini (1560 m), Oued Ounila (1820 m), ruisseau affluent en rive gauche de l’oued Ounila (1820 m), Oued Ounila (Timhlt, 1600 m), Oued Mellah (Anghessa, 1400 m), Oued Dadès en amont des gorges (1630 m), Oued Dadès (Boumalne, 1530 m), Oued Dadès (Sidi Flah, 1100 m), Oued M’Goun (1530 m), Oued M’Goun en aval de Kelaâ (1370 m), ruisseau émissaire de source débouchant dans un affluent de l’Oued Souss (2350 m); Belqat et al. 2001a, **Rif**, **MA**, **HA**, **AA**; Belqat 2002, **Rif**, **MA**, **HA**, **AA**; Belqat and Dakki 2004, **Rif**, **MA**, **HA**, **AA**; Belqat et al. 2005, **Rif**; Dakki et al. 2008, **MA**, Oued Sebou; Belqat et al. 2011, **Rif**, **MA**, **HA**, **AA**; Adler and Crosskey 2017; Belqat et al. 2018; Adler 2019

﻿*Simulium (Simulium) ﻿ornatum* species group

﻿*Simulium (Simulium) ﻿egregium* Séguy, 1930

Grenier 1930, **HA**; Séguy 1930a: 51 (original description), **HA**; Belqat et al. 2001a, **HA**; Belqat 2002, **HA**; Belqat and Dakki 2004, **HA**; Belqat et al. 2011, **HA**; Adler and Crosskey 2017; Belqat et al. 2018; Adler 2019

﻿*Simulium (Simulium) ﻿intermedium* Roubaud, 1906

= ﻿Simuliumreptansvar.fasciatum Séguy, in Séguy 1930a: 52 (misidentification)

= ﻿*Simulium﻿ornatumvar.﻿nitidifrons* Edwards, in Grenier 1953: 159, Grenier and Théodoridès 1953: 441, Grenier and Faure 1957 [1956]: 840, Grenier and Bailly-Choumara 1970: 102, Bailly-Choumara and Beaucournu-Saguez 1978: 143–144

= *Odagmia ﻿﻿﻿nitidifrons* Edwards, in Giudicelli and Dakki 1984: 95, Benhoussa et al. 1988: 160–164

= ﻿*Simulium ﻿﻿﻿nitidifrons* Edwards, in El Mezdi and Giudicelli 1985: 292, 294–295

Séguy 1930a, **HA**; Grenier 1953, **MA**, **HA**; Grenier and Théodoridès 1953, **MA**; Grenier and Faure 1957 [1956], **Rif**, Pré-Rif, **AP**, S Rabat; **MA**, Plain of Meknès; Grenier et al. 1957, **Rif**, Pré-Rif, **HA**; Grenier and Bailly-Choumara 1970, **MA**; Bernard et al. 1972, **MA**; Bailly-Choumara and Beaucournu-Saguez 1978, **Rif**; Giudicelli and Dakki 1984, **Rif**, **MA**; El Mezdi and Giudicelli 1985, **HA**, Khettaras de Marrakech; Benhoussa et al. 1988, **AP**, Oued Bou-Regreg; Giudicelli and Bouzidi 1989, **HA**; Clergue-Gazeau et al. 1991, **MA**, **HA**; Benhoussa et al. 1993, **AP**, Oued Bou-Regreg; Dakki 1997; Giudicelli et al. 2000, **HA**, Oued Réghaya (Neltner, 3800 m), Oued Réghaya (Sidi Chamharouch, 2300 m), Oued Réghaya (lmlil, 1740 m), Oued Réghaya (Aguersioual, 1550 m), Oued Réghaya (Moulay Brahim, 1200 m), Oued Réghaya (Tahanaout, 890 m), ruisselet émissaire de source débouchant dans Oued Réghaya en amont d’lmlil (1750 m), ruisselet émissaire de source débouchant dans l’assif M’zik (1850 m), ruisselet émissaire de source débouchant dans l’assif N’Ouarzane (3000 m), ruisseau émissaire de source (assif N’Ouarzane, 3000 m), assif N’Ouarzane (Irhoulidene, 2800 m), ruisseau affluent en rive droite de l’assif N’Ouarzane (2400 m), Oued N’fis (amont Ijoukak, 1600 m), Oued N’fis (amont Wirgan, 1200 m), Oued N’fis (980 m), Oued N’fis (amont retenue Lalla Takerkoust, 660 m), ruisseau de Tinzart (émissaire de source: 2850 m), ruisseau de Tifni (émissaire de source: 2780 m), ruisseau de Likemt (émissaire de source: 2670 m), ruisseau de Tougroudadene (émissaire de source: 2660 m), assif Oukaimeden (2600 m), source hélocrène au niveau du cirque d’Oukaimeden (2660 m), assif Tiferguine (2500 m), assif Oukaimeden (2450 m), ruisseau émissaire de source débouchant dans l’assif Oukaimeden (1740 m), complexe rhéocrène formé par des émissaires de source débouchant dans l’assif Oukaimeden (1730 m), affluent temporaire en rive gauche de l’assif Oukaimeden (1630 m), affluent temporaire en rive gauche de l’assif Oukaimeden (1360 m), affluent temporaire en rive droite de l’assif Oukaimeden (1260 m), affluent en rive droite de l’assif Oukaimeden (1300 m), assif Tarzaza (1200 m), assif Tarzaza (1000 m), cours inférieur de l’oued Ourika (850 m), Oued Rdat en amont de Taddert (1850 m), affluent temporaire en rive gauche de Oued Rdat (1400 m), Oued Rdat (1600 m), Oued Rdat (1230 m), Oued Tensift (600–700 m), khetarras (450–600 m), Oued Tessaout au niveau d’Aït Tamli (1620 m), Oued Lakdar en aval de la retenue de Sidi Driss (1030 m), ruisseau émissaire de source formant le début de l’assif Imini (2090 m), assif Imini (1560 m), Oued Ounila (1820 m), ruisseau affluent en rive gauche de l’oued Ounila (1820 m), Oued Ounila (Timhlt, 1600 m), Oued Mellah (Anghessa, 1400 m), Oued Dadès en amont des gorges (1630 m), Oued Dadès (Boumalne, 1530 m), Oued Dadès (Sidi Flah, 1100 m), Oued M’Goun (1530 m), Oued M’oun en aval de Kelaâ (1370 m), ruisseau émissaire de source débouchant dans un affluent de l’Oued Souss (2350 m); Belqat et al. 2001a, **Rif**, **MA**, **HA**; Belqat 2002, **Rif**, **MA**, **HA**; Belqat and Dakki 2004, **Rif**, **MA**, **HA**; Belqat et al. 2005, **Rif**; Belqat et al. 2008, **Rif**; Belqat et al. 2011, **Rif**, **AP**, **MA**, **HA**; Adler and Crosskey 2017; Belqat et al. 2018; Adler 2019

﻿*Simulium (Simulium) ﻿ornatum* Meigen, 1818 (complex)

= *reptans*varfasciatum, in Séguy 1930: 52

[*subornatum*: Séguy 1925/1930, not Edwards]

Séguy 1930a: 52 (﻿*ornatum* and *subornatum* records), **HA**; Grenier 1953, **HA**; Bailly-Choumara and Beaucournu-Saguez 1978, **Rif**; Clergue-Gazeau et al. 1991, **MA**, **HA**; Dakki 1997; Giudicelli et al. 2000, **HA**, Oued Réghaya (Neltner, 3800 m), Oued Réghaya (Sidi Chamharouch, 2300 m), Oued Réghaya (lmlil, 1740 m), Oued Réghaya (Aguersioual, 1550 m), Oued Réghaya (Moulay Brahim, 1200 m), Oued Réghaya (Tahanaout, 890 m), ruisselet émissaire de source débouchant dans Oued Réghaya en amont d’lmlil (1750 m), ruisselet émissaire de source débouchant dans l’assif M’zik (1850 m), ruisselet émissaire de source débouchant dans l’assif N’Ouarzane (3000 m), ruisseau émissaire de source (assif N’Ouarzane, 3000 m), assif N’Ouarzane (Irhoulidene, 2800 m), ruisseau affluent en rive droite de l’assif N’Ouarzane (2400 m), Oued N’fis (amont Ijoukak, 1600 m), Oued N’fis (amont Wirgan, 1200 m), Oued N’fis (980 m), Oued N’fis (amont retenue Lalla Takerkoust, 660 m), ruisseau de Tinzart (émissaire de source: 2850 m), ruisseau de Tifni (émissaire de source: 2780 m), ruisseau de Likemt (émissaire de source: 2670 m), ruisseau de Tougroudadene (émissaire de source: 2660 m), assif Oukaimeden (2600 m), source hélocrène au niveau du cirque d’Oukaimeden (2660 m), assif Tiferguine (2500 m), assif Oukaimeden (2450 m), ruisseau émissaire de source débouchant dans l’assif Oukaimeden (1740 m), complexe rhéocrène formé par des émissaires de source débouchant dans l’assif Oukaimeden (1730 m), affluent temporaire en rive gauche de l’assif Oukaimeden (1630 m), affluent temporaire en rive gauche de l’assif Oukaimeden (1360 m), affluent temporaire en rive droite de l’assif Oukaimeden (1260 m), affluent en rive droite de l’assif Oukaimeden (1300 m), assif Tarzaza (1200 m), assif Tarzaza (1000 m), cours inférieur de l’oued Ourika (850 m), Oued Rdat en amont de Taddert (1850 m), affluent temporaire en rive gauche de Oued Rdat (1400 m), Oued Rdat (1600 m), Oued Rdat (1230 m), Oued Tensift (600–700 m), khetarras (450–600 m), Oued Tessaout au niveau d’Aït Tamli (1620 m), Oued Lakdar en aval de la retenue de Sidi Driss (1030 m), ruisseau émissaire de source formant le début de l’assif Imini (2090 m), assif Imini (1560 m), Oued Ounila (1820 m), ruisseau affluent en rive gauche de l’oued Ounila (1820 m), Oued Ounila (Timhlt, 1600 m), Oued Mellah (Anghessa, 1400 m), Oued Dadès en amont des gorges (1630 m), Oued Dadès (Boumalne, 1530 m), Oued Dadès (Sidi Flah, 1100 m), Oued M’Goun (1530 m), Oued M’Goun en aval de Kelaâ (1370 m), ruisseau émissaire de source débouchant dans un affluent de l’Oued Souss (2350 m); Belqat et al. 2001a, **Rif**, **MA**, **HA**, **AA**; Belqat 2002, **Rif**, **MA**, **HA**, **AA**; Belqat and Dakki 2004, **Rif**, **MA**, **HA**, **AA**; Belqat et al. 2005, **Rif**; Belqat et al. 2008, **Rif**; Dakki et al. 2008, **MA**, Oued Sebou; Belqat et al. 2011, **Rif**, **MA**, **HA**, **AA**; Adler and Crosskey 2017; Belqat et al. 2018; Adler 2019

﻿*Simulium (Simulium) trifasciatum* Curtis, 1839

Belqat et al. 2001a, **Rif**; 2001b, **Rif**; Belqat 2002, **Rif**; Belqat and Dakki 2004, **Rif**; Belqat et al. 2005, **Rif**; Belqat et al. 2008, **Rif**; Dakki et al. 2008, **MA**, Oued Sebou; Belqat et al. 2011, **Rif**; Adler and Crosskey 2017; Belqat et al. 2018; Adler 2019

﻿*Simulium (Simulium) ﻿variegatum* species group

Bailly-Choumara and Beaucournu-Saguez (1981: 52–54): groupe *monticola* (“﻿sp. ﻿nova A” and “﻿sp. ﻿nova B”)

﻿*Simulium (Simulium) atlasicum* Giudicelli & Bouzidi, 1989

Giudicelli and Bouzid 1989: 146–151 (original description), **HA**, near village Aguelmous; Clergue-Gazeau et al. 1991, **HA**; Dakki 1997; Giudicelli et al. 2000, **HA**, Oued Réghaya (Neltner, 3800 m), Oued Réghaya (Sidi Chamharouch, 2300 m), Oued Réghaya (lmlil, 1740 m), Oued Réghaya (Aguersioual, 1550 m), Oued Réghaya (Moulay Brahim, 1200 m), Oued Réghaya (Tahanaout, 890 m), ruisselet émissaire de source débouchant dans Oued Réghaya en amont d’lmlil (1750 m), ruisselet émissaire de source débouchant dans l’assif M’zik (1850 m), ruisselet émissaire de source débouchant dans l’assif N’Ouarzane (3000 m), ruisseau émissaire de source (assif N’Ouarzane, 3000 m), assif N’Ouarzane (Irhoulidene, 2800 m), ruisseau affluent en rive droite de l’assif N’Ouarzane (2400 m), Oued N’fis (amont Ijoukak, 1600 m), Oued N’fis (amont Wirgan, 1200 m), Oued N’fis (980 m), Oued N’fis (amont retenue Lalla Takerkoust, 660 m), ruisseau de Tinzart (émissaire de source: 2850 m), ruisseau de Tifni (émissaire de source: 2780 m), ruisseau de Likemt (émissaire de source: 2670 m), ruisseau de Tougroudadene (émissaire de source: 2660 m), assif Oukaimeden (2600 m), source hélocrène au niveau du cirque d’Oukaimeden (2660 m), assif Tiferguine (2500 m), assif Oukaimeden (2450 m), ruisseau émissaire de source débouchant dans l’assif Oukaimeden (1740 m), complexe rhéocrène formé par des émissaires de source débouchant dans l’assif Oukaimeden (1730 m), affluent temporaire en rive gauche de l’assif Oukaimeden (1630 m), affluent temporaire en rive gauche de l’assif Oukaimeden (1360 m), affluent temporaire en rive droite de l’assif Oukaimeden (1260 m), affluent en rive droite de l’assif Oukaimeden (1300 m), assif Tarzaza (1200 m), assif Tarzaza (1000 m), cours inférieur de l’oued Ourika (850 m), Oued Rdat en amont de Taddert (1850 m), affluent temporaire en rive gauche de Oued Rdat (1400 m), Oued Rdat (1600 m), Oued Rdat (1230 m), Oued Tensift (600–700 m), khetarras (450–600 m), Oued Tessaout au niveau d’Aït Tamli (1620 m), Oued Lakdar en aval de la retenue de Sidi Driss (1030 m), ruisseau émissaire de source formant le début de l’assif Imini (2090 m), assif Imini (1560 m), Oued Ounila (1820 m), ruisseau affluent en rive gauche de l’oued Ounila (1820 m), Oued Ounila (Timhlt, 1600 m), Oued Mellah (Anghessa, 1400 m), Oued Dadès en amont des gorges (1630 m), Oued Dadès (Boumalne, 1530 m), Oued Dadès (Sidi Flah, 1100 m), Oued M’Goun (1530 m), Oued M’Goun en aval de Kelaâ (1370 m), ruisseau émissaire de source débouchant dans un affluent de l’Oued Souss (2350 m); Belqat et al. 2001a, **HA**; Belqat 2002, **HA**; Belqat and Dakki 2004, **HA**; Belqat et al. 2011, **HA**; Adler and Crosskey 2017; Belqat et al. 2018; Adler 2019

﻿*Simulium (Simulium) berberum* Giudicelli & Bouzidi, 1989

Giudicelli and Bouzidi 1989: 151–156 (original description), **HA**, assif n’Ouarzane; Clergue-Gazeau et al. 1991, **HA**; Dakki 1997; Giudicelli et al. 2000, **HA**, Oued Réghaya (Neltner, 3800 m), Oued Réghaya (Sidi Chamharouch, 2300 m), Oued Réghaya (lmlil, 1740 m), Oued Réghaya (Aguersioual, 1550 m), Oued Réghaya (Moulay Brahim, 1200 m), Oued Réghaya (Tahanaout, 890 m), ruisselet émissaire de source débouchant dans Oued Réghaya en amont d’lmlil (1750 m), ruisselet émissaire de source débouchant dans l’assif M’zik (1850 m), ruisselet émissaire de source débouchant dans l’assif N’Ouarzane (3000 m), ruisseau émissaire de source (assif N’Ouarzane, 3000 m), assif N’Ouarzane (Irhoulidene, 2800 m), ruisseau affluent en rive droite de l’assif N’Ouarzane (2400 m), Oued N’fis (amont Ijoukak, 1600 m), Oued N’fis (amont Wirgan, 1200 m), Oued N’fis (980 m), Oued N’fis (amont retenue Lalla Takerkoust, 660 m), ruisseau de Tinzart (émissaire de source: 2850 m), ruisseau de Tifni (émissaire de source: 2780 m), ruisseau de Likemt (émissaire de source: 2670 m), ruisseau de Tougroudadene (émissaire de source: 2660 m), assif Oukaimeden (2600 m), source hélocrène au niveau du cirque d’Oukaimeden (2660 m), assif Tiferguine (2500 m), assif Oukaimeden (2450 m), ruisseau émissaire de source débouchant dans l’assif Oukaimeden (1740 m), complexe rhéocrène formé par des émissaires de source débouchant dans l’assif Oukaimeden (1730 m), affluent temporaire en rive gauche de l’assif Oukaimeden (1630 m), affluent temporaire en rive gauche de l’assif Oukaimeden (1360 m), affluent temporaire en rive droite de l’assif Oukaimeden (1260 m), affluent en rive droite de l’assif Oukaimeden (1300 m), assif Tarzaza (1200 m), assif Tarzaza (1000 m), cours inférieur de l’oued Ourika (850 m), Oued Rdat en amont de Taddert (1850 m), affluent temporaire en rive gauche de Oued Rdat (1400 m), Oued Rdat (1600 m), Oued Rdat (1230 m), Oued Tensift (600–700 m), khetarras (450–600 m), Oued Tessaout au niveau d’Aït Tamli (1620 m), Oued Lakdar en aval de la retenue de Sidi Driss (1030 m), ruisseau émissaire de source formant le début de l’assif Imini (2090 m), assif Imini (1560 m), Oued Ounila (1820 m), ruisseau affluent en rive gauche de l’oued Ounila (1820 m), Oued Ounila (Timhlt, 1600 m), Oued Mellah (Anghessa, 1400 m), Oued Dadès en amont des gorges (1630 m), Oued Dadès (Boumalne, 1530 m), Oued Dadès (Sidi Flah, 1100 m), Oued M’Goun (1530 m), Oued M’Goun en aval de Kelaâ (1370 m), ruisseau émissaire de source débouchant dans un affluent de l’Oued Souss (2350 m); Belqat et al. 2001a, **HA**; Belqat 2002, **HA**; Belqat and Dakki 2004, **HA**; Belqat et al. 2011, **HA**; Adler and Crosskey 2017; Belqat et al. 2018; Adler 2019

﻿*Simulium (Simulium) ﻿variegatum* Meigen, 1818

Bailly-Choumara and Beaucournu-Saguez 1978, **Rif**, **HA**; Bailly-Choumara and Beaucournu-Saguez 1981, **HA**; Giudicelli and Bouzidi 1989; Clergue-Gazeau et al. 1991; Dakki 1997; Giudicelli et al. 2000, **HA**, Oued Réghaya (Neltner, 3800 m), Oued Réghaya (Sidi Chamharouch, 2300 m), Oued Réghaya (lmlil, 1740 m), Oued Réghaya (Aguersioual, 1550 m), Oued Réghaya (Moulay Brahim, 1200 m), Oued Réghaya (Tahanaout, 890 m), ruisselet émissaire de source débouchant dans Oued Réghaya en amont d’lmlil (1750 m), ruisselet émissaire de source débouchant dans l’assif M’zik (1850 m), ruisselet émissaire de source débouchant dans l’assif N’Ouarzane (3000 m), ruisseau émissaire de source (assif N’Ouarzane, 3000 m), assif N’Ouarzane (Irhoulidene, 2800 m), ruisseau affluent en rive droite de l’assif N’Ouarzane (2400 m), Oued N’fis (amont Ijoukak, 1600 m), Oued N’fis (amont Wirgan, 1200 m), Oued N’fis (980 m), Oued N’fis (amont retenue Lalla Takerkoust, 660 m), ruisseau de Tinzart (émissaire de source: 2850 m), ruisseau de Tifni (émissaire de source: 2780 m), ruisseau de Likemt (émissaire de source: 2670 m), ruisseau de Tougroudadene (émissaire de source: 2660 m), assif Oukaimeden (2600 m), source hélocrène au niveau du cirque d’Oukaimeden (2660 m), assif Tiferguine (2500 m), assif Oukaimeden (2450 m), ruisseau émissaire de source débouchant dans l’assif Oukaimeden (1740 m), complexe rhéocrène formé par des émissaires de source débouchant dans l’assif Oukaimeden (1730 m), affluent temporaire en rive gauche de l’assif Oukaimeden (1630 m), affluent temporaire en rive gauche de l’assif Oukaimeden (1360 m), affluent temporaire en rive droite de l’assif Oukaimeden (1260 m), affluent en rive droite de l’assif Oukaimeden (1300 m), assif Tarzaza (1200 m), assif Tarzaza (1000 m), cours inférieur de l’oued Ourika (850 m), Oued Rdat en amont de Taddert (1850 m), affluent temporaire en rive gauche de Oued Rdat (1400 m), Oued Rdat (1600 m), Oued Rdat (1230 m), Oued Tensift (600–700 m), khetarras (450–600 m), Oued Tessaout au niveau d’Aït Tamli (1620 m), Oued Lakdar en aval de la retenue de Sidi Driss (1030 m), ruisseau émissaire de source formant le début de l’assif Imini (2090 m), assif Imini (1560 m), Oued Ounila (1820 m), ruisseau affluent en rive gauche de l’oued Ounila (1820 m), Oued Ounila (Timhlt, 1600 m), Oued Mellah (Anghessa, 1400 m), Oued Dadès en amont des gorges (1630 m), Oued Dadès (Boumalne, 1530 m), Oued Dadès (Sidi Flah, 1100 m), Oued M’Goun (1530 m), Oued M’Goun en aval de Kelaâ (1370 m), ruisseau émissaire de source débouchant dans un affluent de l’Oued Souss (2350 m); Belqat et al. 2001a, **Rif**, **HA**; Belqat 2002, **Rif**, **HA**; Belqat and Dakki 2004, **Rif**, **HA**; Belqat et al. 2005, **Rif**, **HA**; Belqat et al. 2011, **Rif**, **HA**; Adler and Crosskey 2017; Belqat et al. 2018; Adler 2019

﻿*Simulium (Simulium) ﻿xanthinum* Edwards, 1933

= ﻿*Simulium﻿gaudi* Grenier and Faure, in Grenier and Faure 1957 [1956]: 838–840

Grenier and Faure 1957 [1956], **Rif**, Pré-Rif, **HA**; Grenier et al. 1957; Bailly-Choumara and Beaucournu-Saguez 1978, **Rif**; Clergue-Gazeau et al. 1991, **MA**; Giudicelli et al. 2000, **HA**, Oued Réghaya (Neltner, 3800 m), Oued Réghaya (Sidi Chamharouch, 2300 m), Oued Réghaya (lmlil, 1740 m), Oued Réghaya (Aguersioual, 1550 m), Oued Réghaya (Moulay Brahim, 1200 m), Oued Réghaya (Tahanaout, 890 m), ruisselet émissaire de source débouchant dans Oued Réghaya en amont d’lmlil (1750 m), ruisselet émissaire de source débouchant dans l’assif M’zik (1850 m), ruisselet émissaire de source débouchant dans l’assif N’Ouarzane (3000 m), ruisseau émissaire de source (assif N’Ouarzane, 3000 m), assif N’Ouarzane (Irhoulidene, 2800 m), ruisseau affluent en rive droite de l’assif N’Ouarzane (2400 m), Oued N’fis (amont Ijoukak, 1600 m), Oued N’fis (amont Wirgan, 1200 m), Oued N’fis (980 m), Oued N’fis (amont retenue Lalla Takerkoust, 660 m), ruisseau de Tinzart (émissaire de source: 2850 m), ruisseau de Tifni (émissaire de source: 2780 m), ruisseau de Likemt (émissaire de source: 2670 m), ruisseau de Tougroudadene (émissaire de source: 2660 m), assif Oukaimeden (2600 m), source hélocrène au niveau du cirque d’Oukaimeden (2660 m), assif Tiferguine (2500 m), assif Oukaimeden (2450 m), ruisseau émissaire de source débouchant dans l’assif Oukaimeden (1740 m), complexe rhéocrène formé par des émissaires de source débouchant dans l’assif Oukaimeden (1730 m), affluent temporaire en rive gauche de l’assif Oukaimeden (1630 m), affluent temporaire en rive gauche de l’assif Oukaimeden (1360 m), affluent temporaire en rive droite de l’assif Oukaimeden (1260 m), affluent en rive droite de l’assif Oukaimeden (1300 m), assif Tarzaza (1200 m), assif Tarzaza (1000 m), cours inférieur de l’oued Ourika (850 m), Oued Rdat en amont de Taddert (1850 m), affluent temporaire en rive gauche de Oued Rdat (1400 m), Oued Rdat (1600 m), Oued Rdat (1230 m), Oued Tensift (600–700 m), khetarras (450–600 m), Oued Tessaout au niveau d’Aït Tamli (1620 m), Oued Lakdar en aval de la retenue de Sidi Driss (1030 m), ruisseau émissaire de source formant le début de l’assif Imini (2090 m), assif Imini (1560 m), Oued Ounila (1820 m), ruisseau affluent en rive gauche de l’oued Ounila (1820 m), Oued Ounila (Timhlt, 1600 m), Oued Mellah (Anghessa, 1400 m), Oued Dadès en amont des gorges (1630 m), Oued Dadès (Boumalne, 1530 m), Oued Dadès (Sidi Flah, 1100 m), Oued M’Goun (1530 m), Oued M’Goun en aval de Kelaâ (1370 m), ruisseau émissaire de source débouchant dans un affluent de l’Oued Souss (2350 m); Belqat et al. 2001a, **Rif**, **MA**, **HA**; Belqat 2002, **Rif**, **MA**, **HA**; Carles-Tolrá 2002; Belqat and Dakki 2004, **Rif**, **MA**, **HA**; Belqat et al. 2005, **Rif**; Belqat et al. 2008, **Rif**; Belqat et al. 2011, **Rif**, **MA**, **HA**; Adler and Crosskey 2017; Belqat et al. 2018; Adler 2019

﻿*Simulium (Trichodagmia*) *albellum* species group

﻿*Simulium (Trichodagmia) auricoma* Meigen, 1818

= ﻿*Simulium (Obuchovia) auricoma* Meigen, 1818, in Belqat et al. 2011: 52

Belqat 2000, **Rif**; Belqat et al. 2001a, **Rif**; Belqat 2002, **Rif**; Belqat and Dakki 2004, **Rif**; Belqat et al. 2005, **Rif**; Belqat et al. 2011, **Rif**; Adler and Crosskey 2017; Belqat et al. 2018; Adler 2019

﻿*Simulium (Trichodagmia) galloprovinciale* Giudicelli, 1963 [1962]

= ﻿*Simulium (Obuchovia) galloprovinciale* Giudicelli, 1963, in Belqat et al. 2011: 52

Belqat 2000, **Rif**; Belqat et al. 2001a, **Rif**; Belqat 2002, **Rif**; Belqat and Dakki 2004, **Rif**; Belqat et al. 2005, **Rif**; Belqat et al. 2011, **Rif**; Adler and Crosskey 2017; Belqat et al. 2018; Adler 2019

﻿*Simulium (Trichodagmia) marocanum* Bouzidi & Giudicelli, 1988 [1987]

= ﻿*Simulium (Obuchovia) marocanum* Bouzidi & Giudicelli, 1987, in Belqat et al. 2011: 52

Bouzidi and Giudicelli 1987: 185–195 (original description), **Rif**, near village Bou Adel, **HA**, Oued Rdat (affluent de l’Oued Tensift); Clergue-Gazeau et al. 1991, **HA**; Dakki 1997; Giudicelli et al. 2000, **HA**, Oued Réghaya (Neltner, 3800 m), Oued Réghaya (Sidi Chamharouch, 2300 m), Oued Réghaya (lmlil, 1740 m), Oued Réghaya (Aguersioual, 1550 m), Oued Réghaya (Moulay Brahim, 1200 m), Oued Réghaya (Tahanaout, 890 m), ruisselet émissaire de source débouchant dans Oued Réghaya en amont d’lmlil (1750 m), ruisselet émissaire de source débouchant dans l’assif M’zik (1850 m), ruisselet émissaire de source débouchant dans l’assif N’Ouarzane (3000 m), ruisseau émissaire de source (assif N’Ouarzane, 3000 m), assif N’Ouarzane (Irhoulidene, 2800 m), ruisseau affluent en rive droite de l’assif N’Ouarzane (2400 m), Oued N’fis (amont Ijoukak, 1600 m), Oued N’fis (amont Wirgan, 1200 m), Oued N’fis (980 m), Oued N’fis (amont retenue Lalla Takerkoust, 660 m), ruisseau de Tinzart (émissaire de source: 2850 m), ruisseau de Tifni (émissaire de source: 2780 m), ruisseau de Likemt (émissaire de source: 2670 m), ruisseau de Tougroudadene (émissaire de source: 2660 m), assif Oukaimeden (2600 m), source hélocrène au niveau du cirque d’Oukaimeden (2660 m), assif Tiferguine (2500 m), assif Oukaimeden (2450 m), ruisseau émissaire de source débouchant dans l’assif Oukaimeden (1740 m), complexe rhéocrène formé par des émissaires de source débouchant dans l’assif Oukaimeden (1730 m), affluent temporaire en rive gauche de l’assif Oukaimeden (1630 m), affluent temporaire en rive gauche de l’assif Oukaimeden (1360 m), affluent temporaire en rive droite de l’assif Oukaimeden (1260 m), affluent en rive droite de l’assif Oukaimeden (1300 m), assif Tarzaza (1200 m), assif Tarzaza (1000 m), cours inférieur de l’oued Ourika (850 m), Oued Rdat en amont de Taddert (1850 m), affluent temporaire en rive gauche de Oued Rdat (1400 m), Oued Rdat (1600 m), Oued Rdat (1230 m), Oued Tensift (600–700 m), khetarras (450–600 m), Oued Tessaout au niveau d’Aït Tamli (1620 m), Oued Lakdar en aval de la retenue de Sidi Driss (1030 m), ruisseau émissaire de source formant le début de l’assif Imini (2090 m), assif Imini (1560 m), Oued Ounila (1820 m), ruisseau affluent en rive gauche de l’oued Ounila (1820 m), Oued Ounila (Timhlt, 1600 m), Oued Mellah (Anghessa, 1400 m), Oued Dadès en amont des gorges (1630 m), Oued Dadès (Boumalne, 1530 m), Oued Dadès (Sidi Flah, 1100 m), Oued M’Goun (1530 m), Oued M’Goun en aval de Kelaâ (1370 m), ruisseau émissaire de source débouchant dans un affluent de l’Oued Souss (2350 m); Belqat et al. 2001a, **HA**; Belqat 2002, **HA**; Belqat and Dakki 2004, **HA**; Belqat et al. 2011, **Rif**, **HA**; Adler and Crosskey 2017; Belqat et al. 2018; Adler 2019

﻿*Simulium (Wilhelmia) ﻿equinum* species group

﻿*Simulium (Wilhelmia) ﻿equinum* (Linnaeus, 1758)

= *﻿Simulium ﻿equinum* Linnaeus, in Grenier et al. 1957: 231–232

Grenier et al. 1957, **MA**; Bailly-Choumara and Beaucournu-Saguez 1981, **HA**; Dakki 1997; Belqat et al. 2001a, **HA**; Belqat 2002, **HA**; Belqat and Dakki 2004, **HA**; Dakki et al. 2008, **MA**, Oued Sebou; Belqat et al. 2011, **MA**, **HA**; Adler and Crosskey 2017; Belqat et al. 2018; Adler 2019

﻿*Simulium (﻿Wilhelmia) pseudequinum* Séguy, 1921

= ﻿*Simulium ﻿﻿﻿barbaricum* Séguy, in Séguy 1930a: 51

= ﻿Simulium ﻿equinum var. mediterraneum Puri, in Grenier 1953: 145–148; Grenier and Théodoridès 1953: 436

= ﻿Simulium﻿﻿equinum mediterraneum Puri, in Grenier and Faure 1957 [1956]: 840; Grenier et al. 1957: 232–234

= ﻿*Wilhelmiapseudequinum* Séguy, in Benhoussa et al. 1988: 160–164

Séguy 1930a, **HA**; Grenier 1953, **HA**; Grenier and Théodoridès 1953, **HA**; Grenier and Faure 1957 [1956], **Rif**, Pré-Rif, **AP**, **HA**, **AA**; Meknès; Grenier et al. 1957, **HA**; Bailly-Choumara and Beaucournu-Saguez 1978, **Rif**; Bailly-Choumara and Beaucournu-Saguez 1981, **HA**; Benhoussa et al. 1988, **AP**, Oued Bou-Regreg; Benhoussa et al. 1993, **AP**, Oued Bou-Regreg; Dakki 1997; Clergue-Gazeau et al. 1991, **HA**; Benhoussa et al. 1993, **AP**, Oued Bou-Regreg; Giudicelli et al. 2000, **HA**, Oued Réghaya (Neltner, 3800 m), Oued Réghaya (Sidi Chamharouch, 2300 m), Oued Réghaya (lmlil, 1740 m), Oued Réghaya (Aguersioual, 1550 m), Oued Réghaya (Moulay Brahim, 1200 m), Oued Réghaya (Tahanaout, 890 m), ruisselet émissaire de source débouchant dans Oued Réghaya en amont d’lmlil (1750 m), ruisselet émissaire de source débouchant dans l’assif M’zik (1850 m), ruisselet émissaire de source débouchant dans l’assif N’Ouarzane (3000 m), ruisseau émissaire de source (assif N’Ouarzane, 3000 m), assif N’Ouarzane (Irhoulidene, 2800 m), ruisseau affluent en rive droite de l’assif N’Ouarzane (2400 m), Oued N’fis (amont Ijoukak, 1600 m), Oued N’fis (amont Wirgan, 1200 m), Oued N’fis (980 m), Oued N’fis (amont retenue Lalla Takerkoust, 660 m), ruisseau de Tinzart (émissaire de source: 2850 m), ruisseau de Tifni (émissaire de source: 2780 m), ruisseau de Likemt (émissaire de source: 2670 m), ruisseau de Tougroudadene (émissaire de source: 2660 m), assif Oukaimeden (2600 m), source hélocrène au niveau du cirque d’Oukaimeden (2660 m), assif Tiferguine (2500 m), assif Oukaimeden (2450 m), ruisseau émissaire de source débouchant dans l’assif Oukaimeden (1740 m), complexe rhéocrène formé par des émissaires de source débouchant dans l’assif Oukaimeden (1730 m), affluent temporaire en rive gauche de l’assif Oukaimeden (1630 m), affluent temporaire en rive gauche de l’assif Oukaimeden (1360 m), affluent temporaire en rive droite de l’assif Oukaimeden (1260 m), affluent en rive droite de l’assif Oukaimeden (1300 m), assif Tarzaza (1200 m), assif Tarzaza (1000 m), cours inférieur de l’oued Ourika (850 m), Oued Rdat en amont de Taddert (1850 m), affluent temporaire en rive gauche de Oued Rdat (1400 m), Oued Rdat (1600 m), Oued Rdat (1230 m), Oued Tensift (600–700 m), khetarras (450–600 m), Oued Tessaout au niveau d’Aït Tamli (1620 m), Oued Lakdar en aval de la retenue de Sidi Driss (1030 m), ruisseau émissaire de source formant le début de l’assif Imini (2090 m), assif Imini (1560 m), Oued Ounila (1820 m), ruisseau affluent en rive gauche de l’oued Ounila (1820 m), Oued Ounila (Timhlt, 1600 m), Oued Mellah (Anghessa, 1400 m), Oued Dadès en amont des gorges (1630 m), Oued Dadès (Boumalne, 1530 m), Oued Dadès (Sidi Flah, 1100 m), Oued M’Goun (1530 m), Oued M’Goun en aval de Kelaâ (1370 m), ruisseau émissaire de source débouchant dans un affluent de l’Oued Souss (2350 m); Belqat et al. 2001a, **Rif**, **MA**, **HA**, **AA**; Belqat 2002, **Rif**, **MA**, **HA**, **AA**; Belqat and Dakki 2004, **Rif**, **MA**, **HA**, **AA**; Belqat et al. 2005, **Rif**; Belqat et al. 2008, **Rif**; Dakki et al. 2008, **MA**, Oued Sebou; Belqat et al. 2011, **Rif**, **AP**, **MA**, **HA**, **AA**; Adler and Crosskey 2017; Belqat et al. 2018

﻿*Simulium (Wilhelmia) quadrifila* Grenier, Faure & Laurent, 1957 [1956]

Grenier et al. 1957: 238–239 (original description as form of ﻿*sergenti*), **Rif**, Pré-Rif, **AP**, S Casablanca, **MA**, Meknès, **HA**; Bailly-Choumara and Beaucournu-Saguez 1978, **Rif**, **MA**, **HA**; Clergue-Gazeau et al. 1991, **Rif**, Pré-Rif; Dakki 1997; Belqat et al. 2001a, **Rif**; Belqat 2002, **Rif**, **AP**, S Casablanca, **MA**, **HA**; Belqat and Dakki 2004, **Rif**; Belqat et al. 2005, **Rif**; Dakki et al. 2008, **MA**, Oued Sebou; Belqat et al. 2011, **Rif**, **AP**, **MA**, **HA**; Adler and Crosskey 2017; Belqat et al. 2018; Adler 2019

﻿*Simulium (Wilhelmia) sergenti* (Edwards, 1923)

= ﻿*Simulium ﻿ariasi* Séguy, in Séguy 1925: 231–238; Séguy 1930a: 50; Grenier 1953: 144

= ﻿*Simulium ﻿﻿equinum mediterraneum* Puri, in Grenier and Faure 1957 [1956]: 840; Grenier et al. 1957: 238–240

= ﻿*Wilhelmia ﻿sergenti* Edwards, in Benhoussa et al. 1993: 249

Séguy 1930a, **MA**; Grenier 1953, **MA**; Grenier and Théodoridès 1953, **HA**; Grenier and Faure 1957 [1956], **Rif**, Pré-Rif; Grenier et al. 1957, **Rif**, Pré-Rif, **AP**, S Casablanca, **MA**, **HA**; Bailly-Choumara and Beaucournu-Saguez 1978, **Rif**, **AP**, **MA**, **HA**; Bailly-Choumara and Beaucournu-Saguez 1981, **HA**; Clergue-Gazeau et al. 1991, **Rif**, Pré-Rif, **HA**; Benhoussa et al. 1993, **AP**, Oued Bou-Regreg; Dakki 1997; Giudicelli et al. 2000, **HA**, Oued Réghaya (Neltner, 3800 m), Oued Réghaya (Sidi Chamharouch, 2300 m), Oued Réghaya (lmlil, 1740 m), Oued Réghaya (Aguersioual, 1550 m), Oued Réghaya (Moulay Brahim, 1200 m), Oued Réghaya (Tahanaout, 890 m), ruisselet émissaire de source débouchant dans Oued Réghaya en amont d’lmlil (1750 m), ruisselet émissaire de source débouchant dans l’assif M’zik (1850 m), ruisselet émissaire de source débouchant dans l’assif N’Ouarzane (3000 m), ruisseau émissaire de source (assif N’Ouarzane, 3000 m), assif N’Ouarzane (Irhoulidene, 2800 m), ruisseau affluent en rive droite de l’assif N’Ouarzane (2400 m), Oued N’fis (amont Ijoukak, 1600 m), Oued N’fis (amont Wirgan, 1200 m), Oued N’fis (980 m), Oued N’fis (amont retenue Lalla Takerkoust, 660 m), ruisseau de Tinzart (émissaire de source: 2850 m), ruisseau de Tifni (émissaire de source: 2780 m), ruisseau de Likemt (émissaire de source: 2670 m), ruisseau de Tougroudadene (émissaire de source: 2660 m), assif Oukaimeden (2600 m), source hélocrène au niveau du cirque d’Oukaimeden (2660 m), assif Tiferguine (2500 m), assif Oukaimeden (2450 m), ruisseau émissaire de source débouchant dans l’assif Oukaimeden (1740 m), complexe rhéocrène formé par des émissaires de source débouchant dans l’assif Oukaimeden (1730 m), affluent temporaire en rive gauche de l’assif Oukaimeden (1630 m), affluent temporaire en rive gauche de l’assif Oukaimeden (1360 m), affluent temporaire en rive droite de l’assif Oukaimeden (1260 m), affluent en rive droite de l’assif Oukaimeden (1300 m), assif Tarzaza (1200 m), assif Tarzaza (1000 m), cours inférieur de l’oued Ourika (850 m), Oued Rdat en amont de Taddert (1850 m), affluent temporaire en rive gauche de Oued Rdat (1400 m), Oued Rdat (1600 m), Oued Rdat (1230 m), Oued Tensift (600–700 m), khetarras (450–600 m), Oued Tessaout au niveau d’Aït Tamli (1620 m), Oued Lakdar en aval de la retenue de Sidi Driss (1030 m), ruisseau émissaire de source formant le début de l’assif Imini (2090 m), assif Imini (1560 m), Oued Ounila (1820 m), ruisseau affluent en rive gauche de l’oued Ounila (1820 m), Oued Ounila (Timhlt, 1600 m), Oued Mellah (Anghessa, 1400 m), Oued Dadès en amont des gorges (1630 m), Oued Dadès (Boumalne, 1530 m), Oued Dadès (Sidi Flah, 1100 m), Oued M’Goun (1530 m), Oued M’Goun en aval de Kelaâ (1370 m), ruisseau émissaire de source débouchant dans un affluent de l’Oued Souss (2350 m); Belqat et al. 2001a, **Rif**, **MA**, **HA**; Belqat 2002, **Rif**, **MA**, **HA**; Belqat and Dakki 2004, **Rif**, **MA**, **HA**; Belqat et al. 2005, **Rif**; Belqat et al. 2008, **Rif**; Dakki et al. 2008, **MA**, Oued Sebou; Belqat et al. 2011, **Rif**, **AP**, **MA**, **HA**; Adler and Crosskey 2017; Belqat et al. 2018; Adler 2019

#### ﻿﻿THAUMALEIDAE

K. Kettani, R. Wagner

Number of species: **2**. Expected: 10

Faunistic knowledge of the family in Morocco: poor

﻿***Thaumalea* Ruthe, 1831**

﻿*Thaumalea ﻿﻿﻿bernardi* Vaillant, 1956

Vaillant 1956b, **HA**, Toubkal, Siroua, Lac Tamhda (Anremer), Sidi Chamarouch, Izourar, M’Goum, Oukaimeden; Dakki 1997

﻿*Thaumalea ﻿﻿﻿spinata* Vaillant, 1954^7^

Vaillant 1954a, **HA**, M’Goum, springs powering d’Ameskeur el Fougani, springs powering Izourar lagoon (Azourki), springs powering the lake Tamhda (Anremer), torrent at the bottom of Jebel Siroua, Oukaimeden (Toubkal), Jebel Toubkal, Atend (Sidi Chamarouch)

#### ﻿﻿BLEPHARICERIDAE

K. Kettani, P. Zwick

Number of species: **4**

Blepharicerinae

﻿***Liponeura* Loew, 1844**

﻿*Liponeura ﻿﻿﻿﻿alticola* Giudicelli & Bouzidi, 1987

Giudicelli and Bouzidi 1987, **HA**, Oued Réghaya; Dakki 1997

﻿*Liponeura ﻿﻿﻿megalatlantica* (Vaillant, 1956)

Vaillant 1956c, **HA**, Izourar, Imi-N’Ifri; Giudicelli and Lavandier 1974; Dakki 1997

﻿*Liponeura ﻿rifincola* Zwick, 2013

Zwick 2013, **Rif**, Issaguen (Ketama, 1800 m)

﻿*Liponeura ﻿﻿﻿sirouana* (Vaillant, 1956)

= *Cardiocrepsis ﻿﻿﻿sirouana* Vaillant, in Vaillant 1956b: 234

Vaillant 1956b, **HA**, Siroua (3000 m); Giudicelli and Lavandier 1974; Dakki 1997

### ﻿Bibionoidea

#### ﻿﻿ANISOPODIDAE^8^

K. Kettani, J.-P. Haenni

Number of species: **3**. Expected: 7–8

Faunistic knowledge of the family in Morocco: poor

#### Anisopodinae

﻿***Sylvicola* Harris, 1780**

﻿*Sylvicola ﻿﻿﻿fenestralis* (Scopoli, 1763)^9^

= *Rhyphus ﻿﻿﻿fenestralis* (Scopoli, 1763), in Mouna 1998: 86

Mouna 1998 (no locality given) – MISR

﻿*Sylvicola ﻿﻿﻿fuscatus* (Fabricius, 1775)^**1**^

= *Rhyphus ﻿﻿﻿fuscatus* (Fabricius, 1775), in Mouna 1998: 86

Mouna 1998 (no locality given) – MISR

﻿*Sylvicola ﻿﻿﻿punctatus* (Fabricius, 1787)^**1**^

= *Rhyphus ﻿﻿﻿punctatus* (Fabricius, 1787), in Mouna 1998: 86

Mouna 1998 (no locality given) – MISR

#### ﻿﻿BIBIONIDAE^10^

K. Kettani, J.-P. Haenni

Number of species: **10**. Expected: 20

Faunistic knowledge of the family in Morocco: poor

##### Bibioninae

﻿***Bibio* Geoffroy, 1762**

﻿*Bibio ﻿﻿﻿hortulanus* (Linnaeus, 1758)

= ﻿*Bibio ﻿﻿﻿hortularum* Linnaeus, 1758, in Becker and Stein 1913: 85

Becker and Stein 1913, **Rif**, Tanger; Séguy 1949a, **SA**, Foum Zguid

﻿*Bibio ﻿﻿﻿lanigerus* Meigen, 1818

Ebejer et al. 2019, **Rif**, Amsemlil (PNPB, 1067 m)

﻿*Bibio ﻿﻿﻿laufferi* Strobl, 1906

Ebejer et al. 2019, **MA**, 3.5 km S of Azrou (Ifrane, 1450 m), 20 km S of Azrou (Ifrane, 1720 m)

﻿*Bibio ﻿﻿﻿leucopterus* (Meigen, 1804)

Ebejer et al. 2019, **MA**, 6 km S of Azrou (Ifrane, 1610 m)

﻿*Bibio ﻿﻿﻿marci* (Linnaeus, 1758)

Becker and Stein 1913, **Rif**, Tanger; Haenni 2009, **Rif**, Tanger, **MA**, Ifrane

﻿***Dilophus* Meigen, 1803**

﻿*Dilophus ﻿﻿﻿antipedalis* Wiedemann in Meigen, 1818

Ebejer et al. 2019, **Rif**, Oued Azla (Nwawel, 57 m), Oued Azla (Hallila, 95 m)

﻿*Dilophus* (﻿﻿cf. ﻿*bispinosus* Lundström, 1914)^11^

Séguy 1953a, **AA**, Aïn Chaib (Souss)

﻿*Dilophus ﻿﻿﻿febrilis* (Linnaeus, 1758)

= ﻿*Dilophus ﻿﻿﻿vulgaris* Meigen, 1818, in Séguy 1949a: 153

Séguy 1949a, **AA**, Goulimine, Foum-el-Hassan

﻿*Dilophus ﻿﻿﻿femoratus* Meigen, 1804

= *Philia ﻿﻿﻿femorata* Meigen, 1804, in Séguy 1941: 2

Séguy 1941d, **HA**, Tizi-n’Test (2000 m); Pârvu et al. 2006, **AP**, Merja Zerga

﻿*Dilophus ﻿﻿﻿tridentatus* Walker, 1848

Ebejer et al. 2019, **AP**, Sidi Mokhtar (Essaouira) – MHNN (J.-P. Haenni leg.), MNHN (**SA**, Foum-el-Hassan)

### ﻿Sciaroidea

#### ﻿﻿CECIDOMYIIDAE

K. Kettani, M. Skuhravá, V. Skuhravý

Number of species: **57**. Expected: 100

Faunistic knowledge of the family in Morocco: moderate

##### Lestremiinae

﻿***Lestremia* Macquart, 1826**

﻿*Lestremia ﻿﻿﻿parvostylia* Jaschhof, 1994

Jaschhof 1994, **SA**, Abeino, 15 km N Goulimine; Papp 2007; Gagné 2010; Gagné and Jaschhof 2014; Skuhravá et al. 2017

##### Micromyinae

﻿***Campylomyza* Meigen, 1818**

﻿*Campylomyzaflavipes* Meigen, 1818

Jaschhof 1998, **HA**, Telouet; Skuhravá et al. 2017

﻿*Campylomyza ﻿﻿﻿fusca* Winnertz, 1853

Jaschhof 1998, **HA**, Telouet; Skuhravá et al. 2017

﻿*Campylomyza ﻿﻿﻿mohrigi* Jaschhof, 2009

Jaschhof 2009 (south Morocco); Gagné 2010; Skuhravá et al. 2017

﻿***Monardia* Kieffer, 1895**

﻿*Monardia (Xylopriona) ﻿toxicodendri* (Felt, 1907)

Jaschhof 1998 (South Morocco); Jaschhof 2009; Gagné 2010; Skuhravá et al. 2017

##### Cecidomyiinae

﻿***Asphondylia* Loew, 1850**

﻿*Asphondylia ﻿﻿﻿capparis* Rübsaamen, 1893

Houard 1921, **MA**, Fès; Skuhravá et al. 1984; Skuhravá 1986; Skuhravá et al. 2017

﻿*Asphondylia ﻿﻿﻿cytisi* Frauenfeld, 1873

Mimeur 1949, **HA**, Tanzat (1800 m), **AA**, Jebel Sargho, Amalou Bou Mansour (2000 m); Skuhravá et al. 2017

﻿*Asphondylia ﻿﻿﻿punica* Marchal, 1897

= ﻿*Asphondylia ﻿﻿﻿conglomerata* De Stefani, 1900

Houard 1922, **EM**, Zousfana (Jebel Tagla); Mimeur 1949, **AA**, Agdz; Mouna 1998; Skuhravá et al. 2017

﻿*Asphondylia ﻿﻿﻿scrophulariae* Schiner, 1856

Mimeur 1949, **AP**, Rabat, Arcilla; Skuhravá et al. 2017

﻿*Asphondylia ﻿﻿﻿verbasci* (Vallot, 1827)

Mimeur 1949, **AP**, Maâmora, Rabat, Zaërs; Skuhravá et al. 2017

﻿***Baldratia* Kieffer, 1897**

﻿*Baldratia ﻿﻿﻿salicorniae* Kieffer, 1897

Mimeur 1949, **AP**, Rabat, Salé, Bou-Regreg; Möhn 1966, **EM**, Melilla, Bocona, **AP**, Rabat; Skuhravá et al. 1984; Skuhravá 1986; Skuhravá et al. 2014a; Skuhravá et al. 2017

﻿***Bayeriola* Gagné, 1991**

﻿*Bayeriola ﻿﻿﻿thymicola* (Kieffer, 1888)

Houard 1923, **HA**, Réghaya; Mimeur 1949, **EM**, Sidi Ali Oujda, Jebel Hamra, Itzer, **MA**, Ifrane, Azrou, Bordj-Doumergue, Timhadite, Aguelmane; Skuhravá et al. 1984; Skuhravá 1986; Skuhravá et al. 1993; Gagné 2010 (south of Morocco); Bruun et al. 2012; Gagné and Jaschhof 2014; Skuhravá et al. 2017

﻿***Blastomyia* Kieffer, 1913**

﻿*Blastomyia ﻿﻿﻿origani* (Tavares, 1901)

Houard 1922, **MA**, Col de Bouchtata, Zalagh (Mouret); Skuhravá et al. 1984; Skuhravá 1986; Gagné 2010 (south of Morocco); Gagné and Jaschhof 2014; Skuhravá et al. 2017

﻿***Braueriella* Kieffer, 1896**

﻿*Braueriella ﻿﻿﻿phillyreae* (Löw, 1877)

Houard 1922, **Rif**, Jebel Kébir; Houard 1923, **AP**, Cap Ghir (south of Morocco); Mimeur 1949, **Rif**, Zoumi, Ouezzane, **EM**, Béni Snassen, **AP**, Larache, Zaërs, Mehdia, Sehoul, **MA**, Jebel Said, Taza, Tahala, Tadla; Mouna 1998; Skuhravá et al. 2017 – MNHN (**AP**, Mehdia)

﻿***Contarinia* Rondani, 1860**

﻿*Contarinia ﻿﻿﻿ilicis* Kieffer, 1898

Houard 1919, **MA**, Immouzer; Mimeur 1949, **EM**, Béni Snassen, Ras Foughal, El-Harcha, **MA**, Ifrane, Aït Bou-Mzil, Monts Zaian, Agoumi-n´Aït Mguild; Skuhravá et al. 2017

﻿*Contarinia ﻿﻿﻿﻿luteola* Tavares, 1902

Mimeur 1949, **EM**, El Harcha, **MA**, Ifrane, Djaba, Imouzzer-du-Kandar, Tafechna; Mouna 1998; Skuhravá et al. 2017

﻿*Contarinia ﻿﻿﻿nasturtii* (Kieffer, 1888)

Mouna 1998: 85 (no accurate locality); Skuhravá et al. 2017

﻿*Contarinia ﻿﻿﻿pyrivora* (Riley, 1886)

= *Diplosis ﻿﻿﻿pirivora* Riley, in Mouna 1998: 85

Mouna 1998: 85 (no accurate locality); Skuhravá et al. 2017

﻿***Dasineura* Rondani, 1840**

﻿*Dasineura ﻿﻿﻿affinis* (Kieffer, 1886)

= *﻿﻿Perrisiaaffinis* (Kieffer), in Mouna 1998: 85

Mimeur 1949, **Rif**, Tanger, **EM**, Oujda, **AP**, Gharb, Port-Lyautey, Rabat, Fedala, Casablanca, Mazagan, Settat, Oued-Zem, Mogador, **MA**, Fès, Tahala, Marchand; Mouna 1998; Skuhravá et al. 2017; **AP** (Rabat) – MISR

﻿*Dasineura ﻿﻿﻿asparagi* (Tavares, 1902)

Mimeur 1949, **AP**, Rabat, Zaërs, **HA**, Chaouia; Skuhravá et al. 2017

﻿*Dasineura ﻿﻿﻿crataegi* (Winnertz, 1853)

Mimeur 1949, **EM**, Chaouia des Béni Snassen, **AP**, Rabat, Zaërs, **MA**, Ifrane, Tahala Aguelmane de Sidi Ali, Fès, Agoumi-n´Aït Mguild, Tarhzirt, **AA**, Argana, Imi-n-Tanoute; Skuhravá et al. 2017

﻿*Dasineura ﻿﻿﻿ericaescopariae* (Dufour, 1837)

Houard 1912, **Rif**, Cap Spartel; Rübsaamen 1899; Skuhravá et al. 2017

﻿*Dasineura ﻿﻿﻿helianthemi* (Hardy, 1850)

= ﻿*Contarinia ﻿﻿﻿helianthemi* (Hardy, 1850)

Mimeur 1949, **AP**, Gharb, Maâmora, Zaërs; Skuhravá et al. 2017

﻿*Dasineura ﻿﻿﻿napi* (Loew, 1850)

= ﻿*Dasineura ﻿﻿﻿brassicae* (Winnertz, 1853) in Mouna 1998: 85

Mouna 1998 (no accurate locality); Skuhravá et al. 2017

﻿*Dasineura ﻿﻿﻿periclymeni* (Rübsaamen, 1889)

Mimeur 1949, **AP**, Rabat, Chellah, Yquem, Grou, Korifla, **EM**, Berkane; Skuhravá et al. 2017

﻿*Dasineura ﻿﻿﻿plicatrix* (Loew, 1850)

Mimeur 1949, **EM**, Massif des Béni Snassen, El-Harcha, **AP**, Larache, Korifla, Grou, Yquem, Malah, Rabat, **MA**, Azrou, Ifrane, Monts Zaian, Oulmés, Khénifra, **HA**, Tizi-Machou, **AA**, Bigoudine; Skuhravá et al. 2017

﻿*Dasineura ﻿﻿﻿rosae* (Loew, 1850)

= *Wachtliella ﻿﻿﻿rosarum* (Hardy, 1850)

Mimeur 1949, **MA**, Ifrane, Aguelmane de Sidi Ali, Aguelmane Azigza, Zaad, Bordj-Doumergue, Bekrite, **HA**, Tizi-Machou; Skuhravá et al. 2017

﻿***Dicrodiplosis* Kieffer, 1895**

﻿*Dicrodiplosis ﻿﻿﻿pseudococci* (Felt, 1914)

Harris 1968, **AP**, Rabat, Salé, **HA**, Asni; Skuhravá et al. 1984; Skuhravá 1986; Skuhravá et al. 2017

﻿***Dryomyia* Kieffer, 1898**

﻿*Dryomyia ﻿﻿﻿lichtensteinii* (Löw, 1878)

Mimeur 1949, **EM**, Debdou, Ras Foughal, El-Harcha, **MA**, Taza, Oulmès, Azrou, Aguelmane Azigza, Ifrane, Imouzzer-du-Kandar, Tizi-n´Tretten, Ida-ou Tanane, Azilal, **HA**, Ayachi; Skuhravá et al. 1984; Skuhravá 1986; Oosterbroek 2007; Gagné and Jaschhof 2014; Skuhravá et al. 2017; **MA** (Oulmès) – MISR

﻿***Etsuhoa* Inouye, 1959**

﻿*Etsuhoa ﻿﻿﻿thuriferae* Skuhravá, 1996

Mimeur 1949, **HA**, Sidi-Chamharouch, Aït Bou-Jafar; Skuhravá 1995; Skuhravá et al. 2017

﻿***Feltiella* Rübsaamen, 1910**

﻿*Feltiella ﻿﻿﻿acarisuga* (Vallot, 1827)

Gagné 1995, **AP**, Rabat; Osborne et al. 2002 (productive areas of cereals); Gagné 2004; Gagné and Jaschhof 2014; Skuhravá et al. 2017

﻿***Gephyraulus* Rübsaamen, 1916**

﻿*Gephyraulus ﻿﻿﻿diplotaxis* (Solinas, 1982)

Houard 1922, **EM**, Oasis de Figuig, Jebel Ouazzani; Mimeur 1949, **AP**, Vallée de l´Oued Korifla, Sidi Bouknadel; Skuhravá et al. 2017

﻿*Gephyraulus ﻿﻿﻿raphanistri* (Kieffer, 1886)

Houard 1923, **AP**, Mogador; Mimeur 1949, **AP**, Gharb, Skhirat, Bouznika; Skuhravá et al. 2017

﻿***Houardiella* Kieffer, 1912**

﻿*Houardiella ﻿﻿﻿salicorniae* Kieffer, 1912

Trotter 1904, **Rif**, Tingis; Houard 1912, **Rif**, Tanger; Mimeur 1949, **AP**, Loukous, Larache, Ksob, Ameur, Mogador, Rabat, Salé, Bou-Regreg, **EM**, Moulouya, **AA**, Agadir; Skuhravá et al. 2017

﻿***Iteomyia* Kieffer, 1913**

﻿*Iteomyia ﻿﻿﻿major* (Kieffer, 1889)

Mimeur 1949, **AP**, Korifla, Grou, **MA**, Leghzel; Skuhravá et al. 2017

﻿***Jaapiella* Rübsaamen, 1916**

﻿*Jaapiella ﻿﻿﻿bryoniae* (Bouché, 1847)

Mimeur 1949, **Rif**, Ouezzane, **AP**, Sidi Bouknadel, Mehdia, Temara, Skhirat, Pont-Blondin, Larache, Monod, Boulhaut, Safi, **MA**, Tahala, Fès, Khemisset, Marchand, **AA**, Agadir; Skuhravá et al. 2017

﻿***Lasioptera* Meigen, 1818**

﻿*Lasioptera ﻿﻿﻿berlesiana* Paoli, 1907

Mimeur 1949; Skuhravá et al. 1984; Skuhravá 1986; Skuhravá et al. 2017

﻿*Lasioptera ﻿﻿﻿rubi* (Schrank, 1803)

Mimeur 1949, **AP**, Tinkert, **MA**, Ifrane, Taza, Tarhzirt, **HA**, N´Fis, Oumer-Rbia, Jebel Tardema; Skuhravá et al. 2017

﻿*Lasioptera ﻿﻿﻿thapsiae* Kieffer, 1898

= ﻿*Lasioptera ﻿﻿﻿carophila* F. Löw, in Houard 1923: 699

Houard 1921; Houard 1923, **Rif**, Tanger; Mimeur 1949, **EM**, Béni Snassen, Guercif, **AP**, Settat, **MA**, Tiddas; Mouna 1998; Skuhravá et al. 2017

﻿***Lestodiplosis* Kieffer, 1894**

﻿*Lestodiplosis ﻿﻿﻿aonidiellae* Harris, 1968

Harris 1968, **EM**, Oujda, **AP**, Rabat, **MA**, Fès; Skuhravá et al. 2017

﻿***Mayetiola* Kieffer, 1896**

﻿*Mayetiolaavenae* (Marchal, 1895)

Mouna 1998; Lahloui et al. 2005, **AP**, Settat, Safi, El Jadida, **MA**, Béni Mellal, Khouribga, **HA**, Marrakech, El Kelaâ; Skuhravá et al. 2017

﻿*Mayetiola﻿destructor* (Say, 1817)

Vayssière 1920, **EM**, Oujda; Jourdan 1937; Hudault and Zelensky 1939; Mimeur 1949; Skuhravá et al. 1984; Skuhravá 1986; Gagné et al. 1991; Amri et al. 1992 (productive areas of cereals); El Bouhssini et al. 1992a, 1992b; Lhaloui et al. 1992; El Bouhssini et al. 1996a, 1996b; Khalifi et al. 1996; Azzam et al. 1997; El Bouhssini et al. 1997, 1999; El Bouhssini et al. 1998; Mouna 1998; Naber et al. 2000, 2003; Lhaloui et al. 2001; Lhaloui et al. 2005; Gagné 2010; Gagné and Jaschhof 2014; Skuhravá et al. 2017; **AP** (Rabat), **MA** – MISR

﻿*Mayetiola ﻿﻿﻿hordei* Kieffer, 1909

Mouna 1998; Gagné et al. 1991; Lhaloui et al. 2005, **AP**, Settat, Safi, El Jadida, **MA**, Béni Mellal, Khouribga, **HA**, Marrakech, El Kelaâ; Skuhravá et al. 2005; Gagné 2010; Gagné and Jaschhof 2014; Skuhravá et al. 2017

﻿***Oligotrophus* Latreille, 1805**

﻿*Oligotrophus ﻿﻿﻿panteli* Kieffer, 1898

Mimeur 1949, **EM**, Béni Snassen, **MA**, Aguelmane Azigza, Azrou, **HA**, Ayachi, Aït Bou-Jafar; Skuhravá et al. 2017

﻿*Oligotrophus ﻿﻿﻿valerii* (Tavares, 1904)

= *Arceuthomyia ﻿﻿﻿valerii* (Tavares, 1904)

Mimeur 1949, **HA**, Ayachi, Aït Bou-Jafar; Skuhravá et al. 2017

﻿***Orseolia* Kieffer & Massalongo, 1902**

﻿*Orseolia ﻿﻿﻿cynodontis* Kieffer & Massalongo, 1902

Houard 1922, **Rif**, Tanger, Aïn Dalia; Skuhravá et al. 1984; Skuhravá 1986; Gagné 2010; Gagné and Jaschhof 2014; Skuhravá et al. 2017

﻿***Phyllodiplosis* Kieffer, 1912**

﻿*Phyllodiplosis ﻿﻿﻿cocciferae* (Tavares, 1902)

= *Blastodiplosis ﻿﻿﻿cocciferae* (Tavares, 1902)

Trotter 1904, **Rif**, Cap Spartel; Houard 1912; Mimeur 1949, **Rif**, Cap Spartel, Ouezzane, **EM**, Béni Snassen, El-Harcha, **AP**, Larache, Maâmora, Boulhaut, Zaërs, **MA**, Taza, Djaba, Ifrane, Imouzzer-du-Kandar, Dayet Achlaf, Dayet Ifrah, Michlifen, Bordj-Doumergue, Zaad, Bekrite, Aguelmane Azigza, Agoumi-n´Aït Mguild, Bou-Mzil, **HA**, Bou-Jafar, Jebel Tardema; Skuhravá et al. 1984; Skuhravá 1986; Gagné 2010; Gagné and Jaschhof 2014

﻿***Psectrosema* Kieffer, 1904**

﻿*Psectrosema ﻿﻿﻿tamaricum* (Kieffer, 1912)

= *Amblardiella ﻿﻿﻿tamaricum* Kieffer, in Mouna 1998: 85

Houard 1922, **EM**, Zousfana, near Sidi Youssef; Mimeur 1949, Morocco Mediterranean, continental and sub-saharan, **EM**, Berkane, Moulouya, **AP**, **MA**, Tadla, **HA**, Haouz, **AA**, Tafilalet, **SA**, Agad; Skuhravá et al. 1984; Skuhravá 1986; Mouna 1998; Skuhravá et al. 2014a; Skuhravá et al. 2017

﻿***Resseliella* Seitner, 1906**

﻿*Resseliella ﻿﻿﻿oleisuga* (Targioni-Tozzetti, 1887)

= ﻿*Clinodiplosis ﻿﻿﻿oleisuga* Targioni-Tozzetti, in Mouna 1998: 85

Hafraoui 1966; Skuhravá et al. 1984; Skuhravá 1986; Mouna 1998; Gagné 2010; Gagné and Jaschhof 2014; Skuhravá et al. 2017

﻿***Rhopalomyia* Rübsaamen, 1892**

﻿*Rhopalomyia ﻿﻿﻿navasi* Tavares, 1904

Houard 1922, **EM**, Jebel Mais, Djahifa, **HA**, Aït Ameli; Houard 1923; Mimeur 1949, **EM**, Figuig, Jebel Nokra, Ifkern, Haute Moulouya, **MA**, Tadla; Skuhravá et al. 1993; Skuhravá et al. 2014b; Skuhravá et al. 2017

﻿***Schizomyia* Kieffer, 1889**

﻿*Schizomyia ﻿﻿﻿buboniae* (Frauenfeld, 1859)

Houard 1917, **EM**, Djorf de Taouriet; Houard 1922, **EM**, Jebel Tagla, Aïn Yalon; Skuhravá et al. 2014a; Skuhravá et al. 2017

﻿***Stefaniella* Kieffer, 1898**

﻿*Stefaniella ﻿﻿﻿trinacriae* De Stefani, 1900

Mimeur 1949, **AP**, Oualidia, Zima, Casablanca; Skuhravá et al. 2017

﻿***Stefaniola* Kieffer, 1913**

﻿*Stefaniola ﻿﻿﻿﻿﻿﻿africana* Möhn, 1971

Mimeur 1949, **AP**, Rabat, Salé, Bou-Regreg; Skuhravá et al. 2017

﻿*Stefaniola ﻿﻿﻿bilobata* (Kieffer, 1913)

Houard 1922–1923; Mimeur 1949, **MA**, Tadla, **HA**, Ksar-es-Souk, **AA**, Haouz; Skuhravá et al. 1984; Skuhravá 1986; Skuhravá et al. 1993; Gagné 2010; Gagné and Jaschhof 2014; Skuhravá et al. 2017

﻿*Stefaniola ﻿﻿﻿opulenta* Möhn, 1971

Möhn 1971; Skuhravá et al. 1984; Skuhravá 1986; Gagné 2010; Gagné and Jaschhof 2014; Skuhravá et al. 2017; Pape and Thompson 2019

﻿*Stefaniola ﻿﻿﻿ventriosa* Möhn, 1971

Möhn 1971, **MA**, Oued Gheris; Skuhravá et al. 1984; Skuhravá 1986; Gagné 2010; Gagné and Jaschhof 2014; Skuhravá et al. 2014a; Skuhravá et al. 2017

﻿***Thecodiplosis* Kieffer, 1895**

﻿*Thecodiplosis ﻿﻿﻿brachyntera* (Schwägrichen, 1835)

Mouna 1998: 85 (no accurate locality); Skuhravá et al. 2017

#### ﻿﻿KEROPLATIDAE

K. Kettani, P.J. Chandler

Number of species: **2**. Expected: 50

Faunistic knowledge of the family in Morocco: poor

##### Keroplatinae

﻿***Keroplatus* Bosc, 1792**

﻿*Keroplatus ﻿﻿﻿reaumurii* (Dufour, 1839)

Matile 1986; Chandler et al. 2005; Evenhuis 2006

﻿***Macrocera* Meigen, 1803**

﻿*Macrocera ﻿fasciata* Meigen, 1804

Becker and Stein 1913, **Rif**, Tanger; Chandler and Ribeiro 1995; Evenhuis 2006

#### ﻿﻿MYCETOPHILIDAE

K. Kettani, P.J. Chandler

Number of species: **64**. Expected: 250

Faunistic knowledge of the family in Morocco: poor

##### Mycetophilinae

Exechiini

﻿***Allodiopsis* Tuomikoski, 1966**

﻿*Allodiopsisrustica* Edwards, 1941

Banamar et al. 2020, **Rif**, Dayat Tazia

﻿***Anatella* Winnertz, 1864**

﻿*Anatella ﻿﻿﻿concava* Plassmann, 1990

Banamar et al. 2020, **Rif**, Oued Aârate

﻿***Brevicornu* Marshall, 1896**

﻿*Brevicornu ﻿﻿﻿intermedium* (Santos Abréu, 1920)

Banamar et al. 2020, **Rif**, Forêt Jebel Lakraâ, Oued Tisgris, Oued Aârate, Dayat Tazia, Oued Maggou (Maggou Village), Douar Tizga, maison forestière de Talassemtane, Dayat Jebel Zemzem, Aïn Takhninjoute, Forêt Adrou, Dayat Amsemlil

﻿*Brevicornu ﻿﻿﻿griseicolle* Staeger, 1840

Banamar et al. 2020, **Rif**, Dayat Fifi, Oued Aârate, Dayat Tazia, Oued Maggou (Maggou Village), Aïn El Malaâb, maison forestière de Talassemtane, Douar Tizga, Aïn Takhninjoute, Dayat Amsemlil, Dayat Lemtahane, Dayat avant Taida

﻿*Brevicornu ﻿﻿﻿sericoma* (Meigen, 1830)

Banamar et al. 2020, **Rif**, Chefchaouen, Dayat Fifi, Forêt Jebel Lakraâ, Dayat Fifi, Oued Maggou (Maggou Village), maison forestière de Talassemtane, Douar Tizga, Oued Amsemlil, Grotte d’Hercule, Forêt Adrou, Cascade Chrafate, Oued Aârate, Bab el Karn, Dayat Amsemlil – MNHN (coll. J. Beaucournu)

﻿*Brevicornu ﻿﻿﻿verralli* (Edwards, 1925)

Banamar et al. 2020, **Rif**, Dayat Afersiw, Forêt Jebel Lakraâ

﻿***Cordyla* Meigen, 1803**

﻿*Cordylacrassicornis* Meigen, 1818

Chandler and Ribeiro 1995; Banamar et al. 2020, **Rif**, Douar Abou Boubnar, maison forestière de Talassemtane, Forêt Adrou, Oued Sidi Yahia Aârab, **EM**, Oued Tafoughalt; **Rif** (Chefchaouen) – MNHN

﻿*Cordyla ﻿﻿﻿insons* Laštovka & Matile, 1974

Banamar et al. 2020, **Rif**, Oued Maggou

﻿*Cordyla ﻿﻿﻿murina* Winnertz, 1864

Banamar et al. 2020, **Rif**, Forêt Aïn Boughaba

﻿*Cordyla ﻿﻿﻿styliforceps* (Bukowski, 1934)

Banamar et al. 2020, **Rif**, maison forestière de Talassemtane, Oued Tkaraâ, Oued Sidi Yahia Aârab

﻿***Exechia* Winnertz, 1863**

﻿*Exechia ﻿﻿﻿bicincta* (Staeger, 1840)

Banamar et al. 2020, **Rif**, Oued Kelaâ, Oued Aârate, **EM**, Grotte des Pigeons, Oued Tafoughalt

﻿*Exechia ﻿﻿﻿dorsalis* (Staeger, 1840)

Banamar et al. 2020, **Rif**, Bab el Karn

﻿*Exechia ﻿﻿﻿fulva* Santos Abreu, 1920

= ﻿*Rymosia ﻿﻿﻿exornata* Séguy, in Séguy 1941a: 26

Séguy 1941a, **HA**, Toubkal; Chandler and Ribeiro 1995; Banamar et al. 2020, **Rif**, Oued Kelaâ, Forêt Aïn Boughaba, Forêt Jebel Lakraâ, Dayat Fifi, Aïn Sidi Brahim Ben Arrif, Oued Maggou (Maggou Village), Aïn Takhninjoute, maison forestière de Talassemtane, Oued Amsemlil, Dayat Jebel Zemzem, Aïn Takhninjoute, Bab el Karn, Dayat Fifi, Dayat Amsemlil; **Rif** (Chefchaouen, coll. J. Beaucournu) – MNHN; **Rif** (20 km west of Targuist, coll. A.M. Hutson) – NHMUK

﻿*Exechia ﻿﻿﻿fusca* (Meigen, 1804)

Banamar et al. 2020, **Rif**, Oued Kelaâ, Forêt Jebel Lakraâ, Douar Kitane, Dayat Amsemlil

﻿***Exechiopsis* Tuomikoski, 1966**

﻿*Exechiopsis ﻿﻿﻿coremura* (Edwards, 1928)

Banamar et al. 2020, **Rif**, Cascade Chrafate

﻿***Pseudexechia* Tuomikoski, 1966**

﻿*Pseudexechia ﻿﻿﻿tuomikoskii* (Kjærandsen, 2009)

Banamar et al. 2020, **Rif**, Source Aheramen

﻿***Rymosia* Winnertz, 1864**

﻿*Rymosia ﻿﻿﻿affinis* Winnertz, 1864

Banamar et al. 2020, **Rif**, maison forestière de Talassemtane, Dayat Amsemlil, Aïn Tiouila, Dayat Fifi

﻿*Rymosia ﻿﻿﻿beaucournui* Matile, 1963

Chandler 1994; Chandler and Ribeiro 1995; Chandler et al. 2005; Banamar et al. 2020, **EM**, Grotte des Pigeons; **AP** (Oued y Kern, coll. H. Choumara) – MNHN

﻿*Rymosia ﻿﻿﻿pseudocretensis* Burghele-Balacesco, 1966

Chandler 1994; Chandler et al. 2005; Banamar et al. 2020; **AP** (Oued y Kern, coll. H. Choumara) – MNHN

﻿***Stigmatomeria* Tuomikoski, 1966**

﻿*Stigmatomeriacrassicornis* (Stannius, 1831)

Banamar et al. 2020, **Rif**, Forêt Jebel Lakraâ, Dayat Fifi, Aïn Takhninjoute, maison forestière de Talassemtane, Dayat Amsemlil; **Rif** (Chefchaouen, coll. J. Beaucournu) – MNHN

﻿***Tarnania* Tuomikoski, 1966**

﻿*Tarnania ﻿﻿﻿dziedzickii* (Edwards, 1941)

Banamar et al. 2020, **Rif**, maison forestière de Talassemtane, Dayat Amsemlil, Cascade Chrafate, Dayat Amsemlil, Grotte Aïn El-Aouda – MNHN

##### Mycetophilini

﻿***Mycetophila* Meigen, 1804**

﻿*Mycetophila ﻿﻿﻿alea* Laffoon, 1965

Banamar et al. 2020, **Rif**, Aïn el Ma Bared, maison forestière de Talassemtane, Oued Aârate, Bab el Karn; **Rif** (Chefchaouen, coll. J. Beaucournu) – MNHN

﻿*Mycetophila ﻿﻿﻿britannica* Laštovka & Kidd, 1975

Chandler and Ribeiro 1995; Banamar et al. 2020, **Rif**, Forêt Aïn Boughaba, Forêt Jebel Lakraâ, Douar Kitane, Aïn El Malaâb, oued à 15 km de Fifi, Aïn El Ma Bared, maison forestière de Talassemtane, Oued Maggou (Maggou Village), Aïn Takhninjoute, Grotte d’Hercule, Oued Aârate, Bab el Karn, Dayat Amsemlil, Dayat Lemtahane, Dayat avant Taida, Forêt Taghzout – MNHN

﻿*Mycetophila ﻿﻿﻿deflexa* Chandler, 2001

Banamar et al. 2020, **Rif**, Forêt Taghzout, route Ksar el Kebir–Chefchaouen

﻿*Mycetophila ﻿﻿﻿edwardsi* Lundström, 1913

Banamar et al. 2020, **Rif**, Dayat Fifi, Dayat Tazia, Oued Tkarae, Forêt Jebel Lakraâ, Forêt Taghzout, Grotte d’Hercule, **EM**, Béni Snassen

﻿*Mycetophila ﻿﻿﻿formosa* Lundström, 1911

Banamar et al. 2020, **Rif**, Forêt Jebel Lakraâ, Oued Amsemlil, Dayat Amsemlil, Dayat avant Taida

﻿*Mycetophilamarginata* Winnertz, 1864

Banamar et al. 2020, **Rif**, Aïn Ras el Ma, Oued Maggou (Maggou Village), maison forestière de Talassemtane, Dayat Amsemlil; **Rif** (Chefchaouen, coll. J. Beaucournu) – MNHN

﻿*Mycetophila ﻿﻿﻿perpallida* Chandler, 1993

Banamar et al. 2020, **Rif**, Forêt Aïn Boughaba, Aïn El Malaâb, maison forestière de Talassemtane, Bab el Karn, Dayat Amsemlil, Douar Kitane

﻿*Mycetophila ﻿﻿﻿pictula* Meigen, 1830

Chandler and Ribeiro 1995; Banamar et al. 2020, **Rif**, Forêt Jebel Lakraâ, Oued Maggou (Maggou Village); **Rif** (Chefchaouen, coll. J. Beaucournu) – MNHN

﻿*Mycetophila ﻿﻿﻿sordida* van der Wulp, 1874

Chandler 1994; Banamar et al. 2020, **Rif**, Forêt Jebel Lakraâ; Oued Maggou (Maggou Village); **MA** (Khenolap-el-Ouaer, 1580 m, coll. J. Beaucournu) – MNHN

﻿*Mycetophila ﻿﻿﻿spectabilis* Winnertz, 1864

Banamar et al. 2020, **Rif**, Forêt Jebel Lakraâ, Forêt Aïn Boughaba, oued à 15 km de Fifi, Aïn El Malaâb

﻿*Mycetophila ﻿﻿﻿strigatoides* (Landrock, 1927)

Banamar et al. 2020, **Rif**, Forêt Taghzout, Oued Aârate

﻿*Mycetophila ﻿﻿﻿unicolor* Stannius, 1831

Banamar et al. 2020, **Rif**, Oued Kelaâ

﻿*Mycetophila ﻿﻿﻿vittipes* Zetterstedt, 1852

Banamar et al. 2020, **Rif**, Forêt Jebel Lakraâ, maison forestière de Talassemtane, Dayat Amsemlil

﻿***Phronia* Winnertz, 1863**

﻿*Phronia ﻿﻿﻿biarcuata* (Becker, 1908)

Chandler 1994; Chandler and Ribeiro 1995; Chandler et al. 2005; Banamar et al. 2020, **Rif**, Dayat Tazia, Aïn el Ma Bared, Aïn El Malaâb, Aïn Takhninjoute, maison forestière de Talassemtane, Grotte d’Hercule, Dayat Amsemlil; **Rif** (Chefchaouen, coll. J. Beaucournu) – MNHN

﻿*Phronia ﻿﻿﻿cinerascens* Winnertz, 1864

Banamar et al. 2020, **Rif**, Dayat Amsemlil

﻿*Phronia ﻿﻿﻿nitidiventris* (van der Wulp, 1858)

Banamar et al. 2020, **Rif**, Dayat Afersiw, Oued Aârate

﻿*Phronia ﻿﻿﻿tenuis* Winnertz, 1864

Banamar et al. 2020, **Rif**, Oued Kelaâ, Oued Aârate, Oued Maggou (Maggou Village), Aïn Takhninjoute, Grotte d’Hercule

﻿*Phronia ﻿﻿﻿tyrrhenica* Edwards, 1928

Banamar et al. 2020, **Rif**, Forêt Jebel Lakraâ, maison forestière de Talassemtane, Dayat Amsemlil, Aïn Takhninjoute

﻿*Phronia ﻿﻿﻿willistoni* Dziedzicki, 1889

Banamar et al. 2020, **Rif**, Forêt Jebel Lakraâ, Oued Aârate, maison forestière de Talassemtane, Dayat Amsemlil, Cascade Chrafate, Bab el Karn

﻿***Sceptonia* Winnertz, 1864**

﻿*Sceptonia ﻿﻿﻿intestata* Plassmann & Schacht, 1990

Banamar et al. 2020, **Rif**, maison forestière de Talassemtane, Aïn El Malaâb

﻿*Sceptonia ﻿﻿﻿membranacea* Edwards, 1925

Banamar et al. 2020, **Rif**, oued à 15 km de Fifi, Oued Aârate

﻿***Trichonta* Winnertz, 1864**

﻿*Trichonta ﻿﻿﻿foeda* Loew, 1869

Banamar et al. 2020, **Rif**, Aïn Takhninjoute, Bab el Karn, Dayat Amsemlil, Oued Tkarae

﻿*Trichonta ﻿﻿﻿icenica* Edwards, 1925

Banamar et al. 2020, **EM**, Grotte du Chameau

﻿*Trichonta ﻿﻿﻿vitta* (Meigen, 1830)

Banamar et al. 2020, **Rif**, Forêt Jebel Lakraâ, Bab el Karn

﻿*Trichonta ﻿﻿﻿vulcani* Dziedzicki, 1889

Banamar et al. 2020, **EM**, Grotte du Chameau

﻿***Zygomyia* Winnertz, 1864**

﻿*Zygomyia ﻿﻿﻿humeralis* (Wiedemann, 1817)

Banamar et al. 2020, **Rif**, maison forestière de Talassemtane

﻿*Zygomyia ﻿﻿﻿valida* Winnertz, 1864

Banamar et al. 2020, **Rif**, Aïn Ras el Ma, Oued Aârate

##### Leiinae

﻿***Docosia* Winnertz, 1864**

﻿*Docosia ﻿﻿﻿gilvipes* (Walker, 1856)

Banamar et al. 2020, **Rif**, Forêt Jebel Lakraâ, maison forestière de Talassemtane, Aïn Takhninjoute, Dayat Amsemlil

﻿***Leia* Meigen, 1818**

﻿*Leia ﻿﻿﻿arsona* Hutson, 1978

Banamar et al. 2020, **Rif**, Oued Maggou (Maggou village), Oued Sidi Yahia Aârab, **MA**, Aïn Walili

﻿*Leia ﻿﻿﻿beckeri* Landrock, 1940

Banamar et al. 2020, **Rif**, Aïn Ras el Ma

﻿*Leia ﻿﻿﻿bimaculata* (Meigen, 1804)

Chandler 1994; Chandler et al. 2005; Banamar et al. 2020, **Rif**, Dayat Tazia, maison forestière de Talassemtane, Aïn el Ma Bared, **MA**, Forêt 3.5 km S Azrou; **MA** (Forêt Ifrane, coll. P.N. Lawrence) – NHMUK

﻿***Novakia* Strobl, 1893**

﻿*Novakia ﻿﻿﻿scatopsiformis* Strobl, 1893

Banamar et al. 2020, **Rif**, maison forestière de Talassemtane

﻿*Novakia ﻿﻿﻿simillima* Strobl, 1910

Banamar et al. 2020, **Rif**, Oued Aârate, maison forestière de Talassemtane

##### Gnoristinae

﻿***Boletina* Staeger, 1840**

﻿*Boletina ﻿﻿﻿gripha* Dziedzicki, 1885

Banamar et al. 2020, **Rif**, Dayat Fifi, Oued Aârate, Aïn Sidi Brahim Ben Arrif, Dayat Amsemlil, Grotte d’Hercule, Cascade Chrafate, Oued Sidi Yahia Aârab, Aïn el Ma Bared, Bab el Karn, **MA**, forêt 3.5 km S Azrou

﻿***Coelosia* Winnertz, 1864**

﻿*Coelosia ﻿﻿﻿fusca* Bezzi, 1892

Banamar et al. 2020, **Rif**, Dayat Fifi, Oued Kelaâ, Oued Amsemlil, Dayat Amsemlil, Aïn Takhninjoute, Cascade Chrafate, Bab el Karn, Dayat avant Taida

﻿***Synapha* Meigen, 1818**

﻿*Synapha ﻿fasciata* Meigen, 1818

Banamar et al. 2020, **Rif**, Aïn Sidi Brahim Ben Arrif, Dayat Tazia, Forêt Adrou, Dayat Amsemlil, Dayat avant Taida

﻿*Synaphavitripennis* (Meigen, 1818)

Banamar et al. 2020, **Rif**, Dayat Amsemlil

﻿***Tetragoneura* Winnertz, 1846**

﻿*Tetragoneura ﻿﻿﻿﻿﻿ambigua* Grzegorzek, 1885

Banamar et al. 2020, **Rif**, Forêt Aïn Boughaba, **EM**, Oued Tafoughalt

##### Mycomyinae

﻿***Mycomya* Rondani, 1856**

﻿*Mycomya ﻿﻿﻿flavicollis* (Zetterstedt, 1852)

Banamar et al. 2020, **Rif**, Aïn El Malaâb, maison forestière de Talassemtane

﻿*Mycomya ﻿﻿﻿pygmalion* Väisänen, 1984

Banamar et al. 2020, **Rif**, Oued Amsemlil, Aïn Sidi Brahim Ben Arrif

﻿*Mycomya ﻿﻿﻿tumida* (Winnertz, 1864)

Banamar et al. 2020, **Rif**, Dayat Fifi

##### Sciophilinae

﻿***Azana* Walker, 1856**

﻿*Azana ﻿﻿﻿anomala* Staeger, 1840

Banamar et al. 2020, **Rif**, Oued Maggou (Maggou village), maison forestière de Talassemtane

﻿***Sciophila* Meigen, 1818**

﻿*Sciophila ﻿﻿﻿iberolutea* Chandler & Blasco-Zumeta, 2001

Chandler and Gatt 2000, **AP**, Oued y Kern; Chandler and Blasco-Zumeta 2001, **AP**, Oued y Kern; Bechev and Koç 2006; Banamar et al. 2020, **Rif**, maison forestière de Talassemtane, Dayat Jebel Zemzem, Oued Sidi Yahia Aârab, Bab el Karn, Marabout el Khaloua; **AP** (Oued y Kern, coll. H. Choumara) – MNHN

#### ﻿﻿SCIARIDAE

K. Kettani, K. Heller

Number of species: **70**. Expected: 200–500

Faunistic knowledge of the family in Morocco: poor

﻿***Austrosciara* Schmitz & Mjöberg, 1924**

﻿*Austrosciara ﻿﻿﻿hyalipennis* (Meigen, 1804)

El Ouazzani et al. 2019, **Rif**, Douar El Hamma, **MA**, Lac Ouiouane

﻿***Bradysia* Winnertz, 1867**

﻿*Bradysia ﻿﻿﻿﻿alpicola* (Winnertz, 1867)

El Ouazzani et al. 2019, **MA**, Lac Ouiouane

﻿*Bradysia ﻿﻿﻿bulbigera* Mohrig & Kauschke, 1994

El Ouazzani et al. 2019, **Rif**, Oued Ouara, Oued Ametrasse, Merzouk Bni Salah, Dayat Bayn widane, **HA**, Ouirgane

﻿*Bradysia ﻿﻿﻿cavernicola* Mohrig & Eckert, 1999

Menzel and Heller 2004, **HA**, Ouirgane

﻿*Bradysia ﻿﻿﻿cinerascens* (Grzegorzek, 1884)

El Ouazzani et al. 2019, **Rif**, Issaguen, Anissar (PNPB)

﻿*Bradysia ﻿﻿﻿crinita* Mohrig, 1992

El Ouazzani et al. 2019, **Rif**, Issaguen, Douar El Hamma

﻿*Bradysia ﻿﻿﻿fenestralis* (Zetterstedt, 1838)

El Ouazzani et al. 2019, **Rif**, Forêt R’milat

﻿*Bradysia ﻿﻿﻿fenestrata* (Meigen, 1818)

El Ouazzani et al. 2019, **Rif**, Jebel Zemzem, Oued Tkarâa, Ben Karrich, Dayat Tazia, Perdicaris Park, Tourbière Amsemlil, Dayat Tazia

﻿*Bradysia ﻿﻿﻿flavipila* Tuomikoski, 1960

El Ouazzani et al. 2019, **HA**, Ouirgane

﻿*Bradysia ﻿﻿﻿iberiana* Rudzinski & Baumjohann, 2009

El Ouazzani et al. 2019, **HA**, Ouirgane

﻿*Bradysia ﻿﻿﻿lembkei* Mohrig & Menzel, 1990

El Ouazzani et al. 2019, **Rif**, Oued Maggou, Dayat Tazia, **AP**, Forêt Maâmora, **HA**, Ouirgane, Gerifodene

﻿*Bradysia ﻿﻿﻿lucichaeta* Mohrig & Krivosheina, 1989

Mohrig et al. 1997, **AA**, Sidi Rbat (40 km S Agadir)

﻿*Bradysiamediterranea* Mohrig & Menzel, 1992

Mohrig and Menzel 1992, **HA**

﻿*Bradysia ﻿﻿﻿nigrispina* Menzel, 2006

El Ouazzani et al. 2019, **HA**, Gerifodene

﻿*Bradysia ﻿﻿﻿pectoralis* (Staeger, 1840)

El Ouazzani et al. 2019, **MA**, Lac Ouiouane, **HA**, Ouirgane

﻿*Bradysia ﻿﻿﻿placida* (Winnertz, 1867)

El Ouazzani et al. 2019, **HA**, Ouirgane

﻿*Bradysia ﻿﻿﻿promissa* Mohrig & Röschmann, 1999

El Ouazzani et al. 2019, **Rif**, Beni Barou, Anissar (PNPB), Oued Tkarâa (PNPB), Taida, Marabout Moulay Abdelsalam

﻿*Bradysia ﻿﻿﻿reflexa* Tuomikoski, 1960

El Ouazzani et al. 2019, **HA**, Ouirgane

﻿*Bradysia ﻿﻿﻿regularis* (Lengersdorf, 1934)

El Ouazzani et al. 2019, **Rif**, Talassemtane (maison forestière), **HA**, Ouirgane

﻿*Bradysia ﻿﻿﻿ruginosa* Mohrig, 1994

Mohrig et al. 1997, **SA**, Ablino (15 km N Goulimine); El Ouazzani et al. 2019, **Rif**, Jebel Lakraâ, Aïn El Fakir, **HA**, Amizmiz

﻿*Bradysia ﻿﻿﻿santorina* Mohrig & Menzel, 1992

Mohrig et al. 1997, **AA**, Sidi Rbat (40 km S Agadir)

﻿*Bradysia ﻿﻿﻿scabricornis* Tuomikoski, 1960

El Ouazzani et al. 2019, **Rif**, Oued Ouara, Maggou, Oued Azla, Douar El Hamma, **HA**, Ouirgane, Setti Fatma, Oued Imlil

﻿*Bradysia ﻿﻿﻿subrufescens* Mohrig & Krivosheina, 1989

El Ouazzani et al. 2019, **HA**, Ouirgane

﻿*Bradysia ﻿﻿﻿subsantorina* Mohrig & Kauschke, 1997

El Ouazzani et al. 2019, **HA**, Ouirgane

﻿*Bradysia ﻿﻿﻿tilicola* (Loew, 1850)

El Ouazzani et al. 2019, **Rif**, Douar Tissouka, **MA**, Lac Ouiouane, **HA**, Ouirgane

﻿*Bradysia ﻿﻿﻿transitata* Rudzinski & Baumjohann, 2013

El Ouazzani et al. 2019, **Rif**, Oued Ez-Zarka, Oued Tkarâa (PNPB), Oued Laou, **AP**, Larache (Strawberry farm), Forêt Maâmora, **AA**, Barrage Aoulouz, Assif Tifnout

﻿*Bradysia ﻿﻿﻿trivittata* (Staeger, 1840)

Mohrig and Röschmann 1993, **HA**, **AA**, Sidi Rbat (40 km S Agadir); El Ouazzani et al. 2019, **Rif**, Aïn Tayattine, Oued Ez-Zarka, Oued Bayine, Beni Barou, Douar El Hamma, Douar Mouarâa, Tétouan, **MA**, Lac Ouiouane, **HA**, Télouet, Ouirgane

﻿*Bradysia ﻿﻿﻿vagans* (Winnertz, 1868)

misidentified as ﻿*Bradysia ﻿﻿﻿rufescens* (Zetterstedt, 1852) in Röschmann and Mohrig 1993: 111

Röschmann and Mohrig 1993, **HA**, Talouete; El Ouazzani et al. 2019, **Rif**, Aïn Fouara, **HA**, Ouirgane

﻿*Bradysia ﻿﻿﻿xenoreflexa* Mohrig & Menzel, 1993

El Ouazzani et al. 2019, **AP**, Forêt Maâmora

﻿***Bradysiopsis* Tuomikoski, 1960**

﻿*Bradysiopsis ﻿﻿﻿vittata* (Meigen, 1830)

El Ouazzani et al. 2019, **HA**, Setti Fatma

﻿***Camptochaeta* Hippa & Vilkamaa, 1994**

﻿*Camptochaetajeskei* (Mohrig & Röschmann, 1993)

= ﻿*Corynoptera ﻿jeskei* Mohrig and Röschmann, in Röschmann and Mohrig 1993: 109

Röschmann and Mohrig 1993, **HA**, Talouete (1800 m)

﻿***Corynoptera* Winnertz, 1867**

﻿*Corynoptera ﻿﻿﻿andalusica* Hippa, Vilkamaa & Heller, 2010

El Ouazzani et al. 2019, **MA**, Lac Ouiouane, **HA**, Ouirgane

﻿*Corynoptera ﻿﻿﻿bicuspidata* (Lengersdorf, 1926)

Hippa et al. 2010, **HA**, Ouirgane, Lac Ouiouane

﻿*Corynoptera ﻿﻿﻿bispinulosa* Mohrig & Dimitrova, 1992

El Ouazzani et al. 2019, **EM**, Tafoughalt

﻿*Corynoptera ﻿﻿﻿caesula* Hippa & Menzel, 2004

El Ouazzani et al. 2019, **Rif**, Aïn Kchour, **AP**, Forêt Maâmora

﻿*Corynoptera ﻿﻿﻿cincinnata* Mohrig & Blasco-Zumeta, 1996

Hippa et al. 2010, **HA**, Ouirgane

﻿*Corynoptera ﻿﻿﻿dentiforceps* (Bukowski & Lengersdorf, 1936)

El Ouazzani et al. 2019, **HA**, Ouirgane

﻿*Corynoptera ﻿﻿﻿deserta* Heller & Menzel, 2006

El Ouazzani et al. 2019, **HA**, Ouirgane

﻿*Corynoptera ﻿﻿﻿fatigans* (Johannsen, 1912)

= ﻿*Corynoptera ﻿﻿﻿perpusilla* Winnertz, 1867, in Mohrig et al. 1997: 384

Mohrig et al. 1997, **HA**, Anezal; Hippa et al. 2010 [for nomenclature see Mohrig et al. 2013]

﻿*Corynoptera ﻿﻿﻿gemina* (Hippa & Vilkamaa, 1994)

El Ouazzani et al. 2019, **HA**, Ouirgane

﻿*Corynoptera ﻿﻿﻿globiformis* (Frey, 1945)

El Ouazzani et al. 2019, **Rif**, Talassemtane (maison forestière)

﻿*Corynoptera ﻿﻿﻿hemiacantha* Mohrig & Mamaev, 1992

El Ouazzani et al. 2019, **HA**, Ouirgane

﻿*Corynoptera ﻿﻿﻿iberica* Hippa, Vilkamaa & Heller, 2010

El Ouazzani et al. 2019, **AP**, Forêt Maâmora, Sidi Boughaba

﻿*Corynoptera ﻿﻿﻿inclinata* Hippa, Vilkamaa & Heller, 2010

Hippa et al. 2010, **HA**, Ouirgane

﻿*Corynoptera ﻿﻿﻿irmgardis* (Lengersdorf, 1930)

Hippa et al. 2010, **HA**, Ouirgane

﻿*Corynoptera ﻿﻿﻿postglobiformis* Mohrig, 1993

El Ouazzani et al. 2019, **Rif**, Talassemtane (maison forestière), **HA**, Ouirgane

﻿*Corynoptera ﻿﻿﻿praeparvula* Mohrig & Krivosheina, 1983

El Ouazzani et al. 2019, **HA**, Ouirgane, Amizmiz, Gerifodene

﻿*Corynoptera ﻿﻿﻿saccata* Tuomikoski, 1960

Hippa et al. 2010, **SA**, Goulimine; Mohrig et al. 2012

﻿*Corynoptera ﻿﻿﻿semipedestris* Mohrig & Blasco-Zumeta, 1996

Mohrig et al. 1997, **SA**, Ablino (15 km N Goulimine)

﻿*Corynoptera ﻿﻿﻿spiciceps* Hippa, Vilkamaa & Heller, 2010

Hippa et al. 2010, **HA**, Ouirgane

﻿*Corynoptera ﻿﻿﻿stipidaria* Mohrig, 1994

Hippa et al. 2010, **HA**, Ouirgane

﻿*Corynoptera ﻿﻿﻿subcavipes* Menzel & Smith, 2007

El Ouazzani et al. 2019, **Rif**, Douar El Hamma, Talassemtane (maison forestière)

﻿*Corynoptera ﻿﻿﻿subparvula* Tuomikoski, 1960

El Ouazzani et al. 2019, **HA**, Ouirgane

﻿***Epidapus* Haliday, 1851**

﻿*Epidapus ﻿﻿﻿atomarius* (De Geer, 1778)

El Ouazzani et al. 2019, **Rif**, Aïn el Ma Bared (Fifi)

﻿***Leptosciarella* Tuomikoski, 1960**

﻿*Leptosciarella ﻿﻿﻿dives* (Johannsen, 1912)

Mohrig et al. 2012, **HA**, Ouirgane

﻿*Leptosciarella ﻿﻿﻿parcepilosa* (Strobl, 1900)

El Ouazzani et al. 2019, **AP**, Sidi Boughaba

﻿*Leptosciarella ﻿﻿﻿subviatica* Mohrig & Menzel, 1997

El Ouazzani et al. 2019, **HA**, Ouirgane

﻿*Leptosciarella ﻿﻿﻿tomentosa* (Mohrig & Kauschke, 1994)

El Ouazzani et al. 2019, **AP**, Forêt Maâmora

﻿***Lycoriella* Frey, 1942**

﻿*Lycoriella ﻿﻿﻿agraria* (Felt, 1898)

El Ouazzani et al. 2019, **Rif**, Douar Tissouka

﻿*Lycoriella ﻿﻿﻿sativae* (Johannsen, 1912)

El Ouazzani et al. 2019, **Rif**, M’Diq, Oued Zaouya, **AP**, Larache (strawberry farm), **HA**, Ouirgane

﻿***Pseudolycoriella* Menzel & Mohrig, 1998**

﻿*Pseudolycoriella ﻿﻿﻿morenae* (Strobl, 1900)

El Ouazzani et al. 2019, **Rif**, Perdicaris Park, Bab Tariouant, Oued Maggou, **EM**, Zegzel, **HA**, Ouirgane

﻿***Scatopsciara* Edwards, 1927**

﻿Scatopsciara (Scatopsciara) atomaria (Zetterstedt, 1851)

= ﻿*Scatopsciara ﻿﻿﻿vivida* (Winnertz, 1867), in Röschmann and Mohrig 1993: 111

Röschmann and Mohrig 1993, **HA**, Talouete; El Ouazzani et al. 2019, **Rif**, Oued Ez-Zarka, Oued Tkarâa (PNPB), Oued Guallet, Marécage Lemtahane (PNPB), Oued Ametrasse, Issaguen, Talassemtane (maison forestière), Douar El Hamma, Aïn Fouara, Oued Souk El Had, Merzouk Bni Salah, Oued Maggou, **AP**, Sidi Boughaba, **HA**, Assif Tifnout, Gerifodene, Armed, Amzmiz, Lac Tislit, **AA**, Barrage Mokhtar Soussi

Scatopsciara (Scatopsciara) maroccoensis Mohrig & Jaschhof, 1997

Mohrig et al. 1997, **AA**, Sidi Rbat (40 km S Agadir)

Scatopsiara (Scatopsciara) nana (Winnertz, 1871)

El Ouazzani et al. 2019, **Rif**, Oued Ez-Zarka, Oued Maâmala, Oued Aârkob, Ben Karrich, Merja Sidi Lhaj Merzouk, Tétouan, **HA**, Ouirgane, Anafgou

Scatopsciara (Scatopsciara) ﻿vitripennis (Meigen, 1818)

El Ouazzani et al. 2019, **Rif**, Oued Ouarra, Oued Tkarâa (PNPB), Oued Aoudour, Oued Ametrasse, Oued Aârkob, Oued Boumarouil, Ben Karrich, Dayat Tazia, Aïn El Fakir, Azib de Khmis Mdik, Merzouk Bni Salah, Oued Souk El Had, Oued Maggou, El Malâab (Talassemtane), **AP**, Sidi Boughaba, **MA**, Lac Ouiouane, **HA**, Ouirgane, **AA**, Barrage Aoulouz, Assif Tifnout, Barrage Mokhtar Soussi

﻿Scatopsciara (Xenopygina) curvilinea (Lengersdorf, 1934)

El Ouazzani et al. 2019, **AP**, Sidi Boughaba, **HA**, Aïn Taferaout, Amzmiz, **AA**, Assif Tifnout, Barrage Mokhtar Soussi

﻿﻿Scatopsciara (Xenopygina) subarmata Mohrig & Mamaev, 1983

El Ouazzani et al. 2019, **Rif**, Oued Amsa, **AP**, Larache, **MA**, Mont Habri, **HA**, Ouirgane, **AA**, Id Aissa, Tissint

﻿﻿***Schwenckfeldina* Frey, 1942**

﻿*Schwenckfeldina ﻿﻿﻿carbonaria* (Meigen, 1830)

= ﻿*Sciara ﻿﻿﻿carbonaria* Meigen, in Séguy 1941d: 2

Séguy 1941d, **HA**, Tizi-n’Test (2000 m)

﻿***Sciara* Meigen, 1803**

﻿*Sciara ﻿﻿﻿flavimana* Zetterstedt, 1851

El Ouazzani et al. 2019, **Rif**, Douar El Hamma

*Sciara ﻿﻿﻿﻿hemerobioides* (Scopoli, 1763)

= ﻿Lycoria (Sciara) thomae Linnaeus, in Becker and Stein 1913: 85

Becker and Stein 1913, **Rif**, Tanger

## ﻿Suborder BRACHYCERA

### ﻿Stratiomyoidea

#### ﻿﻿STRATIOMYIDAE

K. Kettani, N. Woodley

Number of species: **40**. Expected: 50–60

Faunistic knowledge of the family in Morocco: moderate

##### Beridinae

﻿***Beris* Latreille, 1802**

﻿*Beris ﻿﻿﻿rozkosnyi* Kassebeer, 1996

Kassebeer 1996; Woodley 2001, **MA**, Meknès, Ifrane; Kehlmaier 2004; Yimlahi et al. 2017

﻿***Chorisops* Rondani, 1856**

﻿*Chorisops ﻿tunisiae* (Becker, 1915)

Haenni 1990, **Rif**, Tanger; Woodley 2001; Kehlmaier 2004; Mason et al. 2006; Koçak and Kemal 2010; Yimlahi et al. 2017; Lebard et al. 2020

##### Clitellariinae

﻿***Pycnomalla* Gerstaecker, 1857**

﻿*Pycnomalla ﻿﻿﻿aterrima* Sack, 1912

Séguy 1930a, **MA**, Tizi-s’Tkrine (1700 m); Séguy 1953a, **MA**, Dayat Aoua; Woodley 2001; Yimlahi et al. 2017

﻿*Pycnomalla ﻿﻿﻿auriflua* (Erichson, 1841)

Séguy 1930a, **MA**, Soufouloud (1900–2100 m), Boulhaut; Duisit 1960, **AP**, Cap Cantin; Woodley 2001

﻿*Pycnomalla ﻿﻿﻿splendens* (Fabricius, 1787)

Séguy 1930a; Séguy 1953a, **MA**, Dayat Aoua; Duisit 1960, **AP**, forest of Maâmora, Casablanca, Cap Cantin; Rozkošný 1983, **AP**, Cap Cantin; Woodley 2001; Kehlmaier 2004; Koçak and Kemal 2010; Yimlahi et al. 2017

##### Nemotelinae

﻿***Lasiopa* Brullé, 1832**

﻿*Lasiopabenoisti* Séguy, 1930

Séguy 1930a, **MA**, Meknès; Duisit 1960, **EM**, Aïn Guettara (Middle Moulouya); Woodley 2001; Koçak and Kemal 2010; Yimlahi et al. 2017

﻿*Lasiopa ﻿﻿﻿pantherina* Séguy, 1930

Séguy 1930a, **EM**, Maharidja; Woodley 2001; Yimlahi et al. 2017

﻿***Nemotelus* Geoffroy, 1762**

﻿*Nemotelus (Camptopelta) ﻿nigrinus* Fallén, 1817

Duisit 1960, **AP**, Khatouat (S Rabat); Dakki 1997; Woodley 2001; Yimlahi et al. 2017

﻿*Nemotelus (Nemotelus) ﻿atriceps* Loew, 1856

Yimlahi et al. 2017, **AA**, village Massa

﻿*Nemotelus (Nemotelus) ﻿cingulatus* Dufour, 1852

Rozkošný 1977, **AP**, Skhirat; Rozkošný 1983, **Rif**, Tanger; Woodley 2001; Yimlahi et al. 2017, **Rif**, Dayat Afrate, Oued Koub

﻿*Nemotelus (Nemotelus) cylindricornis* Rozkošný, 1977

Faucheux 2009, **AP**, Oualidia

﻿*Nemotelus (Nemotelus) ﻿danielssoni* Mason, 1989

Yimlahi et al. 2017, **Rif**, Oued Izelfane (Beni Boufrah)

﻿*Nemotelus (Nemotelus) ﻿latiusculus* Loew, 1871

Lindner 1949; Rozkošný 1977; Rozkošný 1983, **Rif**, Tanger; Woodley 2001; Kehlmaier 2004; Yimlahi et al. 2017, **Rif**, Barrage Moulay Bouchta

﻿*Nemotelus (Nemotelus) ﻿longirostris* (Wiedemann, 1824)

Becker and Stein 1912, 1913, **Rif**, Tanger; Séguy 1930a, **Rif**, Tanger; Duisit 1960, **AP**, Mechra-bel-Ksiri (Gharb), **EM**, Saïdia, Rozkošný 1977; Dakki 1997; Woodley 2001; Yimlahi et al. 2017

﻿*Nemotelus (Nemotelus) ﻿maculiventris* Bigot, 1861

Yimlahi et al. 2017, **Rif**, Oued Zandoula

﻿*Nemotelus (Nemotelus) ﻿nigrifrons* Loew, 1846

Linder 1936; Becker and Stein 1912, **Rif**, Tanger; Rozkošný 1977, **Rif**, Tanger; Woodley 2001; Yimlahi et al. 2017, **Rif**, affluent Tarmast (NPH)

﻿*Nemotelus (Nemotelus) ﻿pantherinus* (Linnaeus, 1758)

Séguy 1930a, **Rif**, Tanger; Duisit 1960, **AP**, Zëar; Rozkošný 1977; Dakki 1997; Woodley 2001; Koçak and Kemal 2010; Üstüner and Hasbenli 2013; Yimlahi et al. 2017

﻿*Nemotelus (Nemotelus) ﻿proboscideus* Loew, 1846

Linder 1936; Yimlahi et al. 2017

﻿*Nemotelus (Nemotelus) ﻿subuliginosus* Rozkošný, 1974

Rozkošný 1977; Woodley 2001, **Rif**, Tanger; Yimlahi et al. 2017

﻿*Nemotelus (Nemotelus) ﻿ventralis* Meigen, 1830

Woodley 2001, **AP**, Essaouira; Yimlahi et al. 2017

﻿*Nemotelus (Nemotelus) ﻿uliginosus* (Linnaeus, 1767)

Duisit 1960, **AP**, Dradek

##### Pachygastrinae

﻿***Pachygaster* Meigen, 1803**

﻿*Pachygaster ﻿﻿﻿atra* Panzer, 1798

Yimlahi et al. 2017, **Rif**, Dayat Mezine

﻿*Pachygaster ﻿﻿﻿maura* Lindner, 1939

Lindner 1939; Woodley 2001, **MA**, Tagzirt; Yimlahi et al. 2017

##### Sarginae

﻿***Chloromyia* Duncan, 1837**

﻿*Chloromyia ﻿﻿﻿formosa* (Scopoli, 1763)

Becker and Stein 1912, 1913, **Rif**, Tanger; Séguy 1930a, **HA**, M’Rassine; Duisit 1960, **AP**, Rabat, Korifla, Khatouat; Woodley 2001; Pârvu et al. 2006, **AP**, Merja Zerga; Pârvu and Zaharia 2007; Popescu-Mirceni 2011; Yimlahi et al. 2017, **Rif**, Taghbalout, Lac Ametrasse, Douar Kitane

##### Stratiomyinae

Oxycerini

﻿***Oxycera* Meigen, 1803**

﻿*Oxycera ﻿﻿﻿germanica* (Szilády, 1932)

= Hermione﻿dorierivar.﻿barbarica, in Vaillant 1956b: 232, 237, 242

Vaillant 1956b, **HA**, Lac Tamhda (Anremer), Tahanaout, Sidi Chamarouch, Aguelmous

﻿*Oxycera ﻿﻿﻿ochracea* (Vaillant, 1950)

= *Hermione ﻿﻿﻿ochracea* Vaillant, in Vaillant 1956b: 237, 244

Vaillant 1956b, **HA**, Lac Tamhda (Anremer), Imi-N’Ifri

﻿*Oxycera ﻿﻿﻿pardalina* (Meigen, 1822)

Yimlahi et al. 2017, **Rif**, Oued Abou Bnar (NPT), Oued Maggou, Oued Achekrade, Ruisseau maison forestière (NPT), **MA**, Cascade Aïn Vittel, Mchacha Aïn Vittel

﻿*Oxycera ﻿﻿﻿rara* (Scopoli, 1763)

= Hermione﻿pulchellavar.﻿similis Vaillant, in Vaillant 1956b: 242

Vaillant 1956b, **HA**, Sidi Chamarouch, Imi-N’Ifri

﻿*Oxycera ﻿﻿﻿terminata* Meigen, 1822

Yimlahi et al. 2017, **Rif**, Cascade Chrafate

﻿*Oxycera ﻿﻿﻿torrentium* (Vaillant, 1950)

= *Hermione ﻿﻿﻿torrentium* Vaillant, in Vaillant 1956b: 240, 241

Vaillant 1956b, **HA**, Izourar

﻿*Oxycera ﻿﻿﻿trilineata* (Linnaeus, 1767)

= *Hermione﻿bucheti* Séguy, *in* Séguy 1939: 62

= Hermione﻿trilineatavar. ﻿﻿﻿﻿﻿﻿﻿﻿﻿algira Vaillant, in Vaillant 1956b: 244

Becker and Stein 1912, 1913, **Rif**, Tanger; Séguy 1930a; Villeneuve 1933; Vaillant 1956b, **HA**, Imi-N’Ifri; Woodley 2001; Üstüner and Hasbenli 2013; Yimlahi et al. 2017, **Rif**, Dayat Aïn Jdioui

﻿***Vanoyia* Villeneuve, 1908**

﻿*Vanoyia ﻿﻿﻿tenuicornis* (Macquart, 1834)

Lindner 1936; Woodley 2001, **Rif**, Tanger; Yimlahi et al. 2017

##### Stratiomyini

﻿***Odontomyia* Meigen, 1803**

﻿*Odontomyia ﻿﻿﻿alolena* (Séguy, 1930)

= *Eulalia ﻿﻿﻿alolena* Séguy, in Séguy 1930a: 65

Séguy 1930a, **Rif**, Tanger, **EM**, Maharidja, **AP**, Casablanca, **MA**, Aïn Leuh (1400–1500 m); Dakki 1997; Woodley 2001, **Rif**, Tanger, **EM**, Maharidja, **AP**, Casablanca, **MA**, Aïn Leuh; Yimlahi et al. 2017

﻿*Odontomyia ﻿﻿﻿﻿angulata* (Panzer, 1798)

Becker and Stein 1912, 1913, **Rif**, Tanger; Woodley 2001; Koçak and Kemal 2010; Mohammadi and Khaghaninia 2015; Yimlahi et al. 2017

﻿*Odontomyia ﻿﻿﻿discolor* (Loew, 1846)

Becker and Stein 1912, 1913, **Rif**, Tanger; Rozkošný 1982, **Rif**, Tanger; Woodley 2001; Koçak and Kemal 2010; Üstüner and Hasbenli 2013; Yimlahi et al. 2017

﻿*Odontomyia ﻿﻿﻿flavissima* (Rossi, 1790)

= *Hadracantha ﻿﻿flavissina ﻿nigripes* Pleske, in Pleske 1925: 27, 32; Séguy 1930a: 66

= *Eulalia ﻿﻿﻿flavissima* Rossi, in Duisit 1960: 121

Séguy 1926a; Séguy 1930a; Duisit 1960, **MA**, Boulhaut; Dakki 1997; Woodley 2001; Yimlahi et al. 2017

﻿*Odontomyia ﻿﻿﻿limbata* (Wiedemann, 1822)

= *Eulalia ﻿﻿﻿limbata* Wiedemann, in Séguy 1930a: 65

Becker and Stein 1912, 1913, **Rif**, Tanger; Séguy 1930a, **Rif**, Tanger, **MA**, Meskedell (1800–1900 m); Dakki 1997; Woodley 2001; Yimlahi et al. 2017, **Rif**, Lac Ametrasse, Aïn Sidi Brahim Ben Arrif, Dayat Afrate, ruisseau mai­son forestière (NPT), Aïn El Malaâb (NPT), Dayat Rmali El Malaâb (NPT), Dayat Tazia; **Rif** (Tahaddart) – MISR

﻿*Odontomyia ﻿﻿﻿microcera* (Séguy, 1930)

= *Eulalia ﻿﻿﻿﻿microcera* Séguy, in Séguy 1930a: 65

Séguy 1930a; Dakki 1997; Woodley 2001, **MA**, Meknès (550 m); Yimlahi et al. 2017

﻿***Stratiomys* Geoffroy, 1762**

﻿*Stratiomys ﻿﻿﻿cenisia* Meigen, 1822

Séguy 1930a, **Rif**, Tanger, **AP**, Rabat, Sidi Bettache, **MA**, Tizi-s’Tkrine (Jebel Ahmar, 1700 m), Meknès, Aïn Sferguila, **HA**, Timhadit; Duisit 1960, **AP**, Maâmora, **MA**, Arhbala (1700 m); Dakki 1997; Woodley 2001; Koçak and Kemal 2010; Mohammadi and Khaghaninia 2015; Yimlahi et al. 2017

﻿*Stratiomys ﻿﻿﻿longicornis* (Scopoli, 1763)

= ﻿Stratiomys (Hirtea) anubis Wiedemann, in Séguy 1930a: 63

Séguy 1930a, **EM**, Itzer (Haute Moulouya), **AP**, Chellah (Rabat), Casablanca, **MA**, Ras el Ksar (1900 m), **HA**, Marrakech; Duisit 1960, **AP**, Rabat, **MA**, Aguelmane Azigza (1800 m); Dakki 1997; Woodley 2001; Barták and Kubik 2005; Koçak and Kemal 2010; Üstüner and Hasbenli 2013; Yimlahi et al. 2017

### ﻿Tabanoidea

#### ﻿﻿ATHERICIDAE

K. Kettani, M. Mouna

Number of species: **2**. Expected: 2

Faunistic knowledge of the family in Morocco: good

##### Athericinae

﻿***Atherix* Meigen, 1803**

﻿*Atherixamicorum* (Thomas, 1985)

= *Ibisiaamicorum* Thomas, in Thomas 1985: 89

Thomas 1985, **HA**, Oued Réghaya near Marabout Sidi Chamarouch (Toubkal, 2310 m); Boumezzough and Thomas 1987

﻿*Atherix ﻿maroccana* (Séguy, 1930)

= *Ibisia ﻿maroccana* Séguy, in Thomas et al. 1995: 64

Séguy 1930a, **MA**, Oued Tigrigra; Thomas et al. 1995, **MA**, Oued Tigrigra (900 m), Timahdit (1830 m), **HA**, Asif Aït Bou Guemmaz (1900 m); Dakki 1997

#### ﻿﻿RHAGIONIDAE

K. Kettani, M.J. Ebejer

Number of species: **4**. Expected: 7

Faunistic knowledge of the family in Morocco: poor

##### Rhagioninae

﻿***Chrysopilus* Macquart, 1826**

﻿*Chrysopilus ﻿﻿﻿asiliformis* (Preyssler, 1791)

= ﻿*Chrysopilus ﻿﻿﻿aureus* (Meigen, 1804), in Séguy 1941a: 29; Dakki 1997: 62

Séguy 1941a, **HA**, Tachdirt (Toubkal, 2500 m); Dakki 1997

﻿*Chrysopilus ﻿﻿﻿pullus* Loew, 1869

Ebejer et al. 2019, **Rif**, Jebel Lakraâ (NPT, 1377–1541 m), Adrou (PNPB, 556 m)

﻿*Chrysopilus ﻿﻿﻿splendidus* (Meigen, 1820)

Ebejer et al. 2019, **Rif**, Oued Kbir (Béni Ratene, 157 m)

﻿*Chrysopilus ﻿tsacasi* Thomas, 1979

Thomas 1979, **HA**, Jebel Toubkal (Tachdirt, 2500 m); Boumezzough and Thomas 1987, **HA**, Oued Réghaya (Imlil, 1750 m), l’azib Oukaimeden (2730 m); Dakki 1997; Kerr 2004

#### ﻿﻿TABANIDAE

K. Kettani

Number of species: **69**. Expected: 75

Faunistic knowledge of the family in Morocco: good

##### Chrysopsinae

Chrysopsini

﻿***Chrysops* Meigen, 1803**

﻿*Chrysops ﻿﻿﻿caecutiens* Linnaeus, 1758

El Haouari and Kettani 2014, **Rif**, Oued Rha, Oued Samsa, Oued Laou (Afertane), Oued Jnane Niche, Oued Berranda, Oued Biyada; El Haouari et al. 2014, **HA**, Imi-n’Tadart

﻿*Chrysops ﻿﻿﻿connexus* Loew, 1858

Becker and Stein 1913, **Rif**, Tanger; Séguy 1930a, **MA**, Timhadit, Oued Yquem, Volubilis, Kenitra, Meknès, **Rif**, Tanger; Leclercq 1967; Leclercq and Maldès 1987; Chvála et al. 1972; **SA** (Guelmim) – MISR

﻿*Chrysopsflavipes* Meigen, 1804

= *Heterochrysops﻿perspicillaris* Loew, in Séguy 1930a: 79

= ﻿*Chrysops ﻿﻿﻿punctifer* Loew, in Séguy 1930a, 79

Séguy 1930a, **AP**, Mogador, **EM**, Haute Moulouya, **MA**, Fès, Volubilis, **AA**, Taroudant; Leclercq 1967, **AA**, Agadir-Tissint (Rocade du Draa); Chvála et al. 1972; Leclercq and Maldès 1987; Kiliç 1999; Müller et al. 2012

﻿*Chrysops ﻿﻿﻿italicus* Meigen, 1804

Chvála et al. 1972; Leclercq and Maldès 1987; Müller et al. 2012

﻿*Chrysopsmauritanicus* Costa, 1893

Séguy 1930a, **AP**, Rabat, Fedhala, Larache, **MA**, Itzer, **HA**, Haute Réghaya; Leclercq 1967, **AP**, Rabat (salt marshes on *Salicornia*); Chvála et al. 1972; Leclercq and Maldès 1987; **AP** (Kénitra) – MISR

﻿*Chrysops ﻿﻿﻿pallidiventris* Kröber, 1922

Séguy 1930a, **AP**, Mogador, **MA**, Fès; Leclercq 1967; Chvála et al. 1972; Leclercq and Maldès 1987; Pape and Thompson 2019

﻿*Chrysops ﻿﻿﻿relictus* Meigen, 1820

Chvála et al. 1972; El Haouari and Kettani 2014, **Rif**, Oued Kbir (Tamuda), Oued Kelaâ (Talembote), Oued Bou Ahmed, Oued Jnane Niche, Oued Berranda, Oued Biyada; El Haouari et al. 2014, **HA**, Imi-n’Tadart, Oulmès, Setti Fatma, Tafza

﻿*Chrysops ﻿﻿﻿viduatus* (Fabricius, 1794)

El Haouari et al. 2014, **HA**, Setti Fatma

﻿***Silvius* Meigen, 1920**

﻿*Silvius ﻿﻿﻿algirus* Meigen, 1830

Séguy 1930a; Leclercq and Maldès 1987; Kiliç 1999

﻿*Silvius ﻿﻿﻿alpinus* (Scopoli, 1763)

= ﻿*Silvius ﻿﻿﻿vituli* Fabricius, 1805, in Séguy 1930a: 78

Séguy 1930a, **MA**, Meknès, Forêt Zaers; Leclercq and Maldès 1987, **AP**, Rabat, **EM**, Béni Snassen, Haute Moulouya, **MA**, Aïn Leuh

﻿*Silvius ﻿﻿﻿variegatus* (Fabricius, 1805)

= *Diachlorus ﻿maroccanus* Bigot, in Surcouf 1921: 143; Séguy 1930a: 78

Surcouf 1921, **Rif**, Tanger; Becker and Stein 1913, **Rif**, Tanger; Séguy 1930a, **Rif**, Tanger, **AP**, Rabat, **EM**, Haute Moulouya; Leclercq 1960, **Rif**, Tanger, **AP**, Larache, Rabat, Salé **EM**, Haute Moulouya; Leclercq 1967; Leclercq and Maldès 1987; Koçak and Kemal 2013a; **AP** (Rabat, Larache) – MISR

##### Pangoniinae

Pangoniini

﻿***Pangonius* Latreille, 1802**

﻿*Pangonius ﻿﻿﻿﻿﻿alluaudi* Séguy, 1930

Séguy 1930a, **MA**, Azrou, Aïn Leuh, Timhadit, Tasrah des Ighrezrane, Talzent, Aharmoumou; Leclercq 1967, **MA**, Ifrane; Leclercq and Maldès 1987; **MA** – MISR

﻿*Pangonius ﻿﻿﻿brevicornis* (Kröber, 1921)

Leclercq 1967; Leclercq and Maldès 1987

﻿*Pangonius ﻿hassani* (Leclercq, 1968)

Leclercq 1968, **MA**, Ifrane, Dayat Aoua; Leclercq and Olsufjev 1975; Leclercq and Maldès 1987, **MA**, Sidi Allal El Bahraoui

﻿*Pangonius ﻿﻿﻿haustellatus* (Fabricius, 1781)

= ﻿*Pangoniusmarginata* (Fabricius, 1805), in Séguy 1930a: 74

= ﻿*Pangonius ﻿﻿﻿aterrima* Dufour 1853, in Séguy 1930a: 74

= ﻿*Pangonius ﻿﻿﻿funebris* Macquart, 1846, in Séguy 1930a: 74

Séguy 1930a, **MA**, Volubilis, Tizi-s’Tkrine, Aïn Leuh, Azrou, **HA**, Asni; Leclercq 1961b, **MA**, Ifrane; Leclercq 1967, 1968; Chvála and Lyneborg 1970; Leclercq and Maldès 1987, **MA**, Sidi Allal El Bahraoui (forest of *Quercussuber* of Maâmora); Müller et al. 2012; **AP** (Dradek near Rabat, Kénitra), **MA** (wide distribution between Azrou and Ras el Ma), **HA** – MISR

﻿*Pangonius ﻿﻿﻿mauritanus* (Linnaeus, 1767)

= ﻿*Pangonius ﻿﻿﻿funebris* Fabricius, 1794, in Séguy 1930a: 76

= ﻿*Pangonius ﻿﻿﻿maculatus* (Fabricius), in Séguy 1953a: 78

Séguy 1930a; Séguy 1953a, **AP**, Cap Ghir; Séguy 1949a, **AA**, Guelmim; Leclercq 1967; Leclercq and Maldès 1987, **MA**, Maamar (800 m); **AP** (Dradek, El Maazi, Mazagan) – MISR

﻿*Pangonius ﻿﻿﻿micans* Meigen, 1820

Leclercq and Maldès 1987

﻿*Pangonius ﻿﻿﻿powelli* Séguy, 1930^12^

= ﻿*Pangoniussobradieli* Séguy, 1934e: 21

Séguy 1930a, **MA**, Bekrit, Tizi-s’Tkrine, Soufouloud; Séguy 1934e; Séguy 1949a, **AA**, Guelmim; Leclercq and Maldès 1987

﻿*Pangonius ﻿raclinae* Leclercq, 1960

Leclercq 1960, **HA**, Tifni by Demnate; Leclercq 1967; Leclercq and Maldès 1987; Bisby et al. 2011

##### Philolichini

﻿***Ectinocerella* Séguy, 1929**

﻿*Ectinocerella ﻿﻿﻿surcoufi* Séguy, 1929

= ﻿*Pangonius ﻿﻿﻿ectinocerella ﻿surcoufi* Séguy, in Séguy 1929a: 100

Séguy 1929a, **MA**, Azrou, Ank El Djemel **AA**, Agadir; Séguy 1930a, **MA**, Azrou, **AA**, Agadir; Leclercq and Maldès 1987, **MA**, From Meknès to Khemisset, near Beth river; Leclercq 1967; **HA** (Tifni) – MISR

##### Tabaninae

Diachlorini

﻿***Dasybasis* Macquart, 1847**

﻿*Dasybasis ﻿﻿﻿barbata* Coscaron & Philip, 1967

= *Surcoufia ﻿﻿﻿barbata* Bigot, 1892, in Séguy 1930a: 78

= *Surcoufia ﻿﻿﻿paradoxa* Kröber, 1925, in Séguy 1930a: 78

Séguy 1930a, **Rif**, Tanger

﻿***Dasyrhamphis* Enderlein, 1922**

﻿*Dasyrhamphis ﻿﻿﻿algirus* (Macquart, 1838)

= ﻿*Atylotus ﻿﻿﻿algirus* Auct, in Séguy 1930a: 82

Séguy 1930a, **AP**, Dradek, **EM**, Oujda, **HA**, Talouet Glaoua; Chvála et al. 1972; Leclercq and Maldès 1987; **AP** (Sibara) – MISR

﻿*Dasyrhamphis ﻿﻿﻿anthracinus* (Meigen, 1820)

= ﻿*Atylotus ﻿﻿﻿anthracinus* Surcouf, 1924, in Séguy 1930a: 82

Séguy 1930a, **AP**, Rabat, Sidi Bettache, **MA**, M’Rirt, Aïn Sferguila, Volubilis

﻿*Dasyrhamphis ﻿﻿﻿ater* (Rossi, 1790)

= ﻿*Tabanus ﻿﻿﻿ater* (Rossi, 1790), in Becker and Stein 1913: 77

= ﻿*Atylotus ﻿﻿﻿ater* Barotte, 1926, in Séguy 1930a: 82

= ﻿*Dasyrhamphis ﻿﻿﻿ater* (Rossi, 1790), in Leclercq and Maldès 1987: 80

Becker and Stein 1913, **Rif**, Tanger; Séguy 1930a, **MA**; Leclercq 1967, **MA**, Ifrane; Leclercq and Maldès 1987, **HA**, Jebel Tazzeka, Bab Ahzar (1200 m), Idni (1700 m); **MA**

– MISR

﻿*Dasyrhamphis ﻿﻿﻿tomentosus* (Macquart, 1846)

= ﻿*Atylotus ﻿﻿﻿tomentosus* Macquart, in Séguy 1930a: 84

Séguy 1930a, **AP**, Rabat, Oued Cherrat, **MA**, Azrou, El Hajeb, Meknès, Aïn Leuh, Tizi-S’Tkrine, Forêt Tiffert, Talzent, Tazarine, Meskedall; Séguy 1949a, **AA**, Guelmim; Leclercq and Maldès 1987

﻿*Dasyrhamphis ﻿﻿﻿villosus* (Macquart, 1838)

= ﻿*Atylotus ﻿﻿﻿villosus* Macquart, 1838, in Séguy 1930a: 84

Séguy 1930a, **MA**, Tameghilt; Leclercq 1967; Leclercq and Maldès 1987; **MA** – MISR

﻿*Dasyrhamphis ﻿﻿﻿nigritus* (Fabricius, 1794)

= *Therioplectes ﻿﻿﻿alexandrinus* Wiedemann, 1830, in Séguy 1930a: 83

Séguy 1930a, **MA**, Aïn Leuh, El Hajeb, M’Rirt, Dar M’Tougui, Dar Kaid M’Tougui, **EM**, Oujda; Leclercq and Maldès 1987

##### Haematopotini

﻿***Haematopota* Meigen, 1803**

﻿*Haematopota ﻿﻿﻿﻿﻿﻿﻿﻿﻿algira* Kröber, 1922

Séguy 1930a; Leclercq 1961b, **MA**, Dayat Aoua; Leclercq 1967; Leclercq 1968, **MA**, Bab Ferrich, Dayat Aoua; Leclercq and Maldès 1987

﻿*Haematopotabenoisti* Séguy, 1930

Séguy 1930a, **AP**, Rabat, **MA**, M’Rirt; Leclercq 1967; Leclercq and Maldès 1987; Pape and Thompson 2019

﻿*Haematopota ﻿﻿﻿bigoti* Gobert, 1880

Séguy 1930a, **MA**, Volubilis; Séguy 1926a; Leclercq 1961b, **MA**, Dayat Aoua; Leclercq 1967; **MA** (Ifrane) – MISR

﻿*Haematopotacrassicornis* Wahlberg, 1848

Becker and Stein 1913, **Rif**, Tanger; Séguy 1930a

﻿*Haematopota ﻿﻿﻿fuscicornis* Becker, 1914

= *Chrysozona ﻿﻿﻿fuscicornis* Povolny, in Becker and Stein 1913: 78

Becker and Stein 1913, **Rif**, Tanger; Séguy 1930a (*sic! fusicornis*), **MA**, Fès; Chvála et al. 1972; Leclercq and Maldès 1987

﻿*Haematopota ﻿﻿﻿grandis* Meigen, 1820

Leclercq 1967, **AP**, Kénitra; Chvála et al. 1972; Leclercq and Maldès 1987

﻿*Haematopota ﻿﻿﻿italica* Meigen, 1804

= ﻿*Haematopota ﻿﻿﻿tenuicornis* Macquart, 1834, in Séguy 1930a: 81

= ﻿*Haematopota ﻿﻿﻿longicornis* Macquart, 1834, in Séguy 1930a: 81

Becker and Stein 1913, **Rif**, Tanger; Séguy 1930a; Chvála et al. 1972

﻿*Haematopota ﻿﻿﻿lambi* Villeneuve, 1921

Leclercq 1961b, **MA**, Dayat Aoua; Leclercq 1967, 1968; Leclercq and Maldès 1987

﻿*Haematopota ﻿﻿﻿ocelligera* (Kröber, 1922)

Leclercq 1961b, **MA**, Dayat Aoua (on a horse); Leclercq 1967, **AP**, Sidi Yahia du Gharb (on *Juncusacutus*); Leclercq 1968, **MA**, Azrou; Leclercq and Maldès 1987

﻿*Haematopotapluvialis* (Linnaeus, 1758)

Becker and Stein 1913, **Rif**, Tanger; Leclercq and Maldès 1987; El Haouari and Kettani 2014, **Rif**, Oued Rha, marshes of Smir, Oued Kbir (Tamuda), Oued Moukhlata (Boujdad), Oued Azla (Mokdassen Oulya), Oued Moulay Bouchta, Oued Jnane Niche, Oued Koudiat Shiba; El Haouari et al. 2014, **HA**, Oulmès, Tafza

﻿*Haematopota ﻿﻿﻿pseudolusitanica* Szilády, 1923

= *Chrysozona ﻿﻿﻿lusitanica* Guérin, 1835, in Séguy 1930a: 81

Séguy 1930a, **MA**, M’Rirt, Sebou; Leclercq 1967, **MA**, Sebou

﻿*Haematopota ﻿﻿﻿subcylindrica* Pandellé, 1888

Leclercq 1967, **AP**, Sidi Yahia du Gharb; El Haouari and Kettani 2014, **Rif**, Oued Rha, Oued Boumarouil, Oued Jnane Niche; El Haouari et al. 2014, **HA**, Tafza

﻿***Heptatoma* Meigen, 1803**

﻿*Heptatoma ﻿﻿﻿pellucens* (Fabricuis, 1779)

El Haouari and Kettani 2014, **Rif**, Oued Rha, Oued Achiar (Bounezzal), Oued Azla (Mokdassen Oulya), Oued Azla (Mokdassen soufla), Oued Imsa (Centre Imsa), bog of Amsemlil, Oued Ouara (Khizana), Oued Boumarouil, Oued Laou (Siflaou), Oued Talembote, Oued Laou (Afertane), Oued Tizharine, Oued Bouhya (Kanar), Bab Tariouant, Oued Taysra (Ketama), Oued Srâ (Ketama); El Haouari et al. 2014, **HA**, Imi-n’Tadart, Oulmès

##### Tabanini

﻿***Atylotus* Osten-Sacken, 1876**

﻿*Atylotus ﻿﻿﻿agrestis* (Wiedemann, 1828)

Ovazza et al. 1968; Pape and Thompson 2019

﻿*Atylotus ﻿﻿﻿agricola* Wiedemann, 1828

= ﻿*Tabanus ﻿﻿﻿agricola* var. *Kröberi* Surcouf, in Séguy 1953a: 78

Séguy 1953a, **SA**, entre Tagounit et Zegdou

﻿*Atylotus ﻿﻿﻿fulvus* (Meigen, 1804)

Leclercq 1961b, **MA**, Aïn Leuh, Bordj Doumergue; Leclercq 1967, **Rif**, Ketama; Chvála et al. 1972; Leclercq and Maldès 1987; Barták and Kubik 2005

﻿*Atylotus ﻿﻿﻿latistriatus* (Brauer, 1880)

= *Dasystipia ﻿﻿﻿nigrifacies* Gobert, 1881, in Séguy 1930a: 84

Séguy 1930a, **MA**, Aïn Leuh; Chvála et al. 1972; Kiliç 1999

﻿*Atylotus ﻿﻿﻿loewianus* (Villeneuve, 1920)

Leclercq 1967, **MA**, Aguelmane Azigza (marshy meadow), Aguelmane de Sidi Ali Leclercq 1968; Chvála et al. 1972

﻿*Atylotus ﻿﻿﻿pulchellus* Loew, 1858

Becker and Stein 1913**Rif**, Tanger; Chvála et al. 1972

﻿*Atylotus ﻿﻿﻿quadrifarius* (Loew, 1874)

Chvála et al. 1972; Müller et al. 2011

﻿*Atylotus ﻿﻿﻿sublunaticornis* (Zetterstedt, 1842)

El Haouari and Kettani 2014, **Rif**, Oued Rha, Oued Kbir (Koudiat Krikra), Oued Martil, Oued Khizana, Oued Laou (Ifansa), Oued Bou Ahmed, Oued Biyada; El Haouari et al. 2014, **HA**, Imi-n’Tadart, Oulmès

﻿***Hybomitra* Enderlein, 1922**

﻿*Hybomitra ﻿﻿﻿arpadi* Szilády 1923

El Haouari et al. 2014, **HA**, Oulmès

﻿*Hybomitra ﻿﻿﻿bimaculata* Macquart, 1826

Ježek 1995; El Haouari and Kettani 2014, **Rif**, Oued Rha, marshes of Lemtahane (PNPB), Oued Raouz, Oued Zarka, Oued Mokhlata (Boujdad), Oued Amsa (Er-Rifiyine), bog of Amsemlil, Oued Talembote (Talembote), Oued Jnane Niche, Oued Biyada, Oued Aârkob, Oued Sidi Yahia Aârab; El Haouari et al. 2014, **HA**, Oulmès

﻿*Hybomitra ﻿﻿﻿distinguenda* (Verrall, 1909)

Ježek et al. 2012; El Haouari et al. 2014, **HA**, Imi-n’Tadart

﻿*Hybomitra ﻿﻿﻿vittata* (Fabricius, 1794)

= *Straba ﻿﻿﻿vittata* Fabricius, 1794, in Séguy 1930a: 83

= ﻿*Tabanus ﻿﻿﻿spectabilis* Loew, 1858, in Séguy 1930a: 83

Séguy 1930a, **Rif**, Tanger **AP**, Maâmora, Rabat, Casablanca, **MA**, Oued Yquem, M’Rirt; Chvála and Lyneborg 1970, **Rif**, Tanger, **EM**, Haute Moulouya; Leclercq and Maldès 1987

﻿***Tabanus* Linnaeus, 1758**

﻿*Tabanus ﻿﻿﻿autumnalis* Linnaeus, 1761

= *Straba ﻿﻿﻿autumnalis* Linnaeus, 1761, in Séguy 1930a: 82

= *Strabaautomnalisvar.﻿brunnescens* Szilády, 1941, in Séguy 1930a: 82

= Strabaautomnalisvar.molestans Becker, 1914, in Séguy 1930a: 83

Loew 1860; Becker and Stein 1913, **Rif**, Tanger; Séguy 1930a, **AP**, Rabat, **EM**, Béni Snassen, Itzer (Haute Moulouya), **MA**, Aïn Leuh; Séguy 1953a, **HA**, Ksar-es-Souk; Leclercq 1961b, **MA**, Dayat Aoua, Aïn Leuh, Immouzer Kander; Leclercq 1967, **AP**, Kénitra (on *Mimosa* grove), Gharb (Sidi Yahia, Sidi Allal Tazi), **MA**, Adjir by Khenifra (on livestock), Leclercq 1968, **MA**, Dayat Aoua; Leclercq and Maldès 1987, **HA**, edges near river Tessaout, Kelaâ des Sraghna; Pârvu et al. 2006, **AP**, Merja Zerga, **MA**, Kasba Tadla; El Haouari et al. 2014, **HA**, Setti Fatma; **MA** (Allal Tazi) – MISR

﻿*Tabanusbarbarus* Coquebert, 1804

Becker and Stein 1913, **Rif**, Tanger; Leclercq 1961b**Rif**, Azib de Ketama; Chvála and Lyneborg 1970, **Rif**, Tanger; Portillo 1982; Leclercq and Maldès 1987, **MA**, marshes around Kasba Tadla; Popescu-Mirceni 2011, **AP**, Merja Zerga

﻿*Tabanus ﻿﻿﻿bifarius* Loew, 1858

= ﻿*Atylotus ﻿﻿﻿bifarius* Loew, 1858, in Séguy 1930a: 83

Séguy 1930a, **EM**, Haute Moulouya

﻿*Tabanus ﻿﻿﻿bovinus* Linnaeus, 1758

Loew 1860; Séguy 1930a; Leclercq 1967; Portillo 1982; Leclercq and Maldès 1987; Barták and Kubik 2005, **MA**, M’Rirt; El Haouari and Kettani 2014, **Rif**, Oued Khemis (Khemis Anjra), Oued Kelaâ (Akchour)

﻿*Tabanus ﻿﻿﻿bromius* Linnaeus, 1758

= *Straba ﻿﻿﻿bromius* Linnaeus, 1758, in Séguy 1930a: 83

Loew 1860; Séguy 1930a, **EM**, Haute Moulouya, **MA**, Aïn Leuh, Ras El Ksar; Leclercq 1961b, **Rif**, Azib de Ketama, **MA**, Ifrane, Immouzer, Azrou, Bordj Doumergue, Dayat Aoua, Aïn Leuh; Leclercq 1967, **MA**, Immouzer des Marmoucha, Adjir by Khenifra; Leclercq 1968, **Rif**, Ketama, **MA**, Bab-Bou-Idir, Bab Ferrich, Ifrane, Col du Zad; Pernot-Visentin and Beaucournu-Saguez 1974, **Rif**; Leclercq and Maldès 1987; El Haouari and Kettani 2014, **Rif**, Oued Samsa, Oued Raouz, Oued Zarka, Oued Khemis (Khemis Anjra), Oued Azla (Mokdassen Oulya), Oued Kelaâ (Akchour), Oued Laou (Sifalaou), Oued Jnane Niche, Oued Berranda; El Haouari et al. 2014, **HA**, Oulmès; **AA** (Errachidia) – MISR

﻿*Tabanus ﻿﻿﻿choumarae* Leclercq, 1967

Leclercq 1967, **AA**, Aouinet Torkoz (down Draa); Leclercq and Olsufjev 1975; Leclercq and Maldès 1987; Pârvu et al. 2006, **AA**, Ouarzazate

﻿*Tabanus ﻿﻿﻿cordiger* Meigen, 1820

Leclercq 1961b, **MA**, Bordj Doumergue, Dayat Aoua; Leclercq 1967, **Rif**, Had el Rouadi, **MA**, Arbala par Ksiba, Boulemane, **AA**, Agadir-Tissint (Rocade du Draa), Akka; Leclercq 1968, **Rif**, Ketama, **MA**, Bab Termas, Taza, Doniet; Leclercq 1986; Leclercq and Maldès 1987, **AA**, Tinmal, edges of Draa river, Agdz, Ourika near Ouarzazate; Barták and Kubik 2005; Müller et al. 2011; Popescu-Mirceni 2011, **HA**, Ouarzazate; El Haouari and Kettani 2014, **Rif**, Oued Berranda; El Haouari et al. 2014, **Rif**, Oued Oueghra

﻿*Tabanus ﻿﻿﻿darimonti* Leclercq, 1964

Leclercq 1967, **MA**, Aïn Leuh; Leclercq 1968, **MA**, Bab Boudir, forêt Bab-Azhar, Immouzer Kander; Portillo 1982; Leclercq and Maldès 1987; Mikuška et al. 2008

﻿*Tabanus ﻿﻿﻿eggeri* Schiner, 1868

= ﻿*Tabanus ﻿﻿﻿intermedius* Egger, 1859, in Séguy 1930a: 83

Séguy 1930a, **MA**, M’Rirt; Leclercq 1968, **Rif**, M’Diq, **MA**, Bab Boudir, El Hajeb, Azrou, Miscliffen; Portillo 1982; Leclercq and Maldès 1987; Mikuška et al. 2008

﻿*Tabanus ﻿﻿﻿leleani* Austen, 1920

= ﻿*Atylotus ﻿﻿﻿leleani* Austen, 1920, in Séguy 1930a: 84

Séguy 1930a, **HA**, upstream of Réghaya; Leclercq 1967; Leclercq and Maldès 1987; **MA** – MISR

﻿*Tabanus ﻿﻿﻿lunatus* Fabricius, 1794

= ﻿*Atylotus ﻿﻿﻿lunatus* Fabricius, 1974, in Séguy 1930a: 84

Séguy 1930a, **EM**, Haute Moulouya, **MA**, Meknès; Leclercq 1961b, **MA**, Ifrane, Immouzer, Bordj Doumergue, Dayat Aoua, Aïn Leuh; Leclercq 1967, **EM**, Haute Moulouya, **MA**, Ajdir by Khenifra (on livestock); Leclercq 1968, **Rif**, Ketama, **MA**, Bab Bouder, Immouzer Kander, El Hajeb, Mishliffen, Jebel Hebri; Portillo 1982; Leclercq and Maldès 1987, **MA**, Afourer (800 m) – MISR

﻿*Tabanus ﻿﻿﻿maculicornis* Zetterstedt, 1842

El Haouari and Kettani 2014, **Rif**, Oued Rha, marshes of Lemtahane, Oued Boumarouil, Oued Laou (Dardara), Oued Kanar, Oued Jnane Niche; El Haouari et al. 2014, **HA**, Imi-n’Tadart, Oulmès, Tafza

﻿*Tabanus ﻿﻿﻿miki* Brauer, 1880

El Haouari and Kettani 2014, **Rif**, Oued Khemis, Oued Boumarouil, Oued Berranda, Oued Biyada; El Haouari et al. 2014, **HA**, Setti Fatma

﻿*Tabanus ﻿﻿﻿nemoralis* Meigen, 1820

Leclercq 1968, **Rif**, Ketama; Portillo 1982; Leclercq and Maldès 1987

﻿*Tabanus ﻿﻿﻿quatuornotatus* Meigen, 1820

Portillo 1982; Leclercq and Maldès 1987; Koçak and Kemal 2010; Müller et al. 2011; El Haouari and Kettani 2014, **Rif**, Oued Maggou, Oued Ouara (Khizana); El Haouari et al. 2014, **HA**, Setti Fatma; **Rif** (Talassemtane) – MISR

﻿*Tabanus ﻿﻿﻿sudeticus* Zeller, 1842

Leclercq 1967; Portillo 1982; Leclercq and Maldès 1987; Gioia Martins-Neto 2003

﻿*Tabanus ﻿﻿﻿tinctus* Walker, 1850

Leclercq 1961b, **MA**, forêt Bab Azhar, Aïn Leuh; Pernot-Visentin and Beaucournu-Saguez 1974, **MA**, **HA** (2300 m); Portillo 1982; Leclercq and Maldès 1987; **MA** (Azrou) – MISR

#### ﻿﻿VERMILEONIDAE

K. Kettani, M.J. Ebejer

Number of species: **6**. Expected: 6

Faunistic knowledge of the family in Morocco: good

##### Vermileoninae

﻿***Lampromyia* Macquart, 1835**

﻿*Lampromyiacylindrica* (Fabricius, 1794)

Stuckenberg 1998, **HA**

﻿*Lampromyia ﻿﻿﻿lecerfi* Séguy, 1928

= ﻿*Lampromyia Le Cerfi* Séguy, in Séguy 1928a: 45

Séguy 1928a, **HA**, Tinmel (Goundafa), Asni; Stuckenberg 1998, **HA**; Kirk-Spriggs and McGregor 2009; Kehlmaier 2014, **HA**; **AP** (Tamri) – MHNN

﻿*Lampromyia ﻿﻿﻿nigripennis* Séguy, 1930

Séguy 1930a, **EM**, Berkane, grove in Tlet n’Rhohr; Stuckenberg 1998, **MA**; Kirk-Spriggs and McGregor 2009; Kehlmaier 2014, **MA**, south of Azrou (1500 m), **HA**

﻿*Lampromyia ﻿pallida* Macquart, 1835

Stuckenberg 1998, **HA**

﻿***Vermileo* Macquart, 1834**

﻿*Vermileo ﻿vermileo* (Linnaeus, 1758)^13^

= ﻿*Vermileo ﻿﻿﻿degeeri* Macquart 1834, in Séguy 1953a: 78

Séguy 1953a, **AP**, Rabat

﻿*Vermileo ﻿﻿﻿nigriventris* Strobl, 1906

Ebejer et al. 2019, **Rif**, Cap Spartel (Tanger, 15 m), Anissar (PNPB, 987 m)

### ﻿Nemestrinoidea

#### ﻿﻿ACROCERIDAE

K. Kettani, E.P. Nartshuk

Number of species: **13**. Expected: 25

Faunistic knowledge of the family in Morocco: poor

##### Acrocerinae

﻿***Acrocera* Meigen, 1803**

﻿*Acrocera ﻿﻿﻿orbicula* (Fabricius, 1787)

Weinberg and Bächli 2002, **HA**, Marrakech, Ouirgane (1000 m)

﻿***Cyrtus* Latreille, 1796**

﻿*Cyrtus ﻿﻿﻿gibbus* (Fabricius, 1794)

Becker and Stein 1913, **Rif**, Tanger; Séguy 1926, **Rif**; Pleske 1930, **Rif**, Tanger; Schlinger 1972; Mouna 1998

﻿*Cyrtusmaroccanus* Séguy, 1930

Schlinger 1972, **Rif**; Mouna 1998

﻿*Cyrtus ﻿﻿﻿pallidus* Gil Collado, 1929

Gil Collado 1929b, **Rif**, Tanger; Schlinger 1972; Mouna 1998

﻿*Cyrtus ﻿﻿﻿pusillus* Macquart, 1834

Pleske 1930, **Rif**, Tanger; Schlinger 1972, **Rif**, Tanger; Mouna 1998

﻿***Ogcodes* Latreille, 1797**

﻿*Ogcodes ﻿﻿﻿zonatus* (Erichson, 1840)

Becker and Stein 1913, **Rif**, Tanger; Séguy 1926; Pleske 1930

﻿***Opsebius* Costa, 1856**

﻿*Opsebius ﻿﻿﻿cyrtus* Séguy, 1930

Mouna 1998; **HA** (Lac Ifni) – MISR

﻿*Opsebius ﻿﻿﻿formosus* Loew, 1871

Becker and Stein 1913, **Rif**, Tanger

﻿*Opsebius ﻿﻿﻿inclinatus* Séguy, 1930

Mouna 1998

﻿*Opsebius ﻿﻿﻿inflatus* (Loew, 1857)

Weinberg and Bächli 2002, **MA**, Azrou, Timahdit Ighboula (1850 m), **HA**, Marrakech, Ouirgane (1000 m)

﻿*Opsebius ﻿﻿﻿pepo* Loew, 1870

Pleske 1930, **Rif**, Tanger

##### Panopinae

﻿***Astomella* Latreille, 1809**

﻿*Astomella ﻿﻿﻿hispaniae* Lamarck, 1816

Gil Collado 1929b, **AP**, Mogador; Mouna 1998

﻿***Physegastrella* Brunetti, 1926**

﻿*Physegastrella ﻿maroccana* Brunetti, 1926

Brunetti 1926; Pleske 1930; Mouna 1998

#### ﻿﻿NEMESTRINIDAE

K. Kettani, D. Barraclough

Number of species: **13**

Faunistic knowledge of the family in Morocco: poor

##### Nemestrininae

﻿***Nemestrinus* Latreille, 1802**

﻿*Nemestrinus ﻿﻿﻿﻿aegyptiacus* (Wiedemann, 1828)

Timon-David 1951; Séguy 1953a, **HA**, Amsed; Bernardi 1973; Mouna 1998; **SA** (Oued el Ma) – MISR

﻿*Nemestrinus ﻿﻿﻿ater* (Olivier, 1810)

Mouna 1998; **EM** (Zaouillet El Atenf) – MISR

﻿*Nemestrinus ﻿﻿﻿escalerai* Arias, 1913

Paramonow 1945; Bernardi 1973, **HA**, Marrakech; Mouna 1998

﻿*Nemestrinus ﻿﻿﻿exalbidus* (Lichtwardt, 1907)

Mouna 1998

﻿*Nemestrinus ﻿﻿﻿fasciatus* (Olivier, 1810)

= *Rhynchocephalus ﻿﻿﻿fasciatus* Olivier, in Mouna 1998: 86

Bernardi 1973; Mouna 1998; **MA** (Immouzer) – MISR

﻿*Nemestrinus ﻿﻿﻿nigrovillosus* Lichtwardt, 1909

Arias 1913; Séguy 1930a, **MA**, Ras el Ma, Azrou, Forêt Tiffert (2000–2200 m), **HA**, Tizi-n’Test, Jebel Imdress (2000–2450 m), Goundafa; Séguy 1941d, **HA**, Tizi-n’Test; Timon-David 1951, **MA**, Tizi-n’Tretten; Bernardi 1973; Mouna 1998

﻿*Nemestrinus ﻿﻿﻿pieltaini* (Gil Collado, 1934)

= *Nemestrellus ﻿﻿﻿pieltaini* Gil Collado, in Gil Collado 1934: 325

Gil Collado 1934, **Rif**, Imasinen, Bab Chiquer, Bab Bagla; Bernardi 1973; Mouna 1998

﻿*Nemestrinus ﻿﻿﻿ruficornis* (Macquart, 1840)

Mouna 1998

﻿*Nemestrinus ﻿﻿﻿rufipes* (Olivier, 1810)

Mouna 1998; **MA** (Timahdit) – MISR

﻿*Nemestrinus ﻿﻿﻿striatus* (Lichtwardt, 1907)

Mouna 1998

##### Trichopsideinae

﻿***Fallenia* Meigen, 1820**

﻿*Fallenia ﻿fasciata* (Fabricius, 1805)

Arias 1913; Séguy 1930a, **AP**, Casablanca, Rabat, Bou Knadel, **MA**, M’Rirt (1200 m); Timon-David 1951, **AP**, Forêt Maâmora; Mouna 1998; **AP** (Rabat, Bou Knadel), **MA** (Aïn Leuh) – MISR

﻿***Neorhynchocephalus* Lichtwardt, 1909**

﻿*Neorhynchocephalus ﻿﻿﻿tauscheri* (Fisher, 1812)

= *Rhynchocephalus ﻿﻿﻿tauscheri* Fischer, in Mouna 1998: 86

Mouna 1998

﻿***Trichopsidea* Westwood, 1839**

﻿*Trichopsidea ﻿﻿﻿costata* (Loew, 1857)

= *Symmictus ﻿﻿﻿costatus* Loew, in Arias 1913: 26, Séguy 1930a: 89

Arias 1913; Séguy 1930a, **MA**, Tameghilt (1900 m); Mouna 1998

### ﻿Asiloidea

#### ﻿﻿ASILIDAE

K. Kettani, G. Tomasovic

Number of species: **131**. Expected: 230

Faunistic knowledge of the family in Morocco: moderate

##### Apocleinae

﻿***Apoclea* Macquart, 1838**

﻿*Apoclea ﻿﻿﻿﻿﻿﻿﻿﻿﻿algira* (Linnaeus, 1767)

Séguy 1953a, **AA**, Tata; Tomasovic 1997; Mouna 1998; Geller-Grimm 2007; Koçak and Kemal 2010; El Hawagry 2011; Ghahari et al. 2014

﻿*Apoclea ﻿﻿﻿micracantha* Loew, 1856

Tomasovic 1997, **HA**, Sidi Mhejmed Ou Said; Geller-Grimm 2007; Koçak and Kemal 2010; El Hawagry 2011; Ghahari et al. 2014

﻿***Eremonotus* Theodor, 1980**

﻿*Eremonotus ﻿﻿﻿hauseri* Geller-Grimm & Hradský, 1998

Geller-Grimm and Hradský 1998, **HA**; Geller-Grimm 2007, **AA**, Agadir

##### Asilinae

﻿***Afroepitriptus* Lehr, 1992**

﻿*Afroepitriptus ﻿﻿﻿beckeri* Lehr, 1992

Geller-Grimm 2007; Koçak and Kemal 2010

﻿***Antiphrisson* Loew, 1849**

﻿*Antiphrisson ﻿﻿﻿trifarius* Loew, 1849

Tomasovic 1997, **HA**, Errachidia, Ziz, Oasis Zouala; Geller-Grimm 2007; Koçak and Kemal 2010; El Hawagry 2011; Ghahari et al. 2014; **HA** (Asni) – MISR

﻿***Asilus* Linnaeus, 1758**

﻿*Asilusbarbarus* Linnaeus, 1758

Becker and Stein 1913, **Rif**, Tanger; Séguy 1930a; Séguy 1941a, **HA**, Tizi-Tamatert (Toubkal, 2250 m); Mouna 1998; Weinberg and Blasco-Zumeta 2004; Geller-Grimm 2007; Koçak and Kemal 2010; Ghahari et al. 2014

﻿*Asilus ﻿﻿﻿crabroniformis* Linnaeus, 1758

Geller-Grimm 2007; Hayat et al. 2008; Koçak and Kemal 2010; Ghahari et al. 2014

﻿*Asilus ﻿﻿﻿tingitanus* Boisduval, 1835

Geller-Grimm 2007, **Rif**, Tanger

﻿***Dysmachus* Loew, 1860**

﻿*Dysmachus ﻿﻿﻿albisetosus* (Macquart, 1850)

Geller-Grimm 2007

﻿*Dysmachus ﻿﻿﻿cochleatus* (Loew, 1854)

Becker and Stein 1913, **Rif**, Tanger; Geller-Grimm 2007

﻿*Dysmachus ﻿﻿﻿cristatus* (Wiedemann, 1820)

= ﻿*Dysmachus ﻿﻿﻿dasynotus* Loew, in Becker and Stein 1913: 72, Timon-David 1951: 138

Becker and Stein 1913, **Rif**, Tanger; Timon-David 1951, **AP**, Rabat, Harcha, Salé, Oued Ksab, **MA**, Ifrane; Mouna 1998; Geller-Grimm 2007; **AP** (Rabat, Cap Cantia) – MISR

﻿*Dysmachus ﻿﻿﻿digitulus* Becker, 1923

Geller-Grimm 2007

﻿*Dysmachus ﻿﻿﻿elapsus* Villeneuve, 1933

Villeneuve 1933, **AP**, Mazagan, Mogador; Mouna 1998; Tomasovic 2001b; Geller-Grimm 2007; **AP** (Cap Cantia) – MISR

﻿*Dysmachus ﻿﻿﻿evanescens* Villeneuve, 1912

Timon-David 1951, **AP**, Sehoul; Mouna 1998; Geller-Grimm 2007

﻿*Dysmachus ﻿﻿﻿trigonus* (Meigen, 1804)

Timon-David 1951, **AP**, Rabat, Chellah, Forêt Maâmora, Ras el Arba, Sehoul, Zaër

﻿***Eccoptopus* Loew, 1860**

﻿*Eccoptopus ﻿﻿﻿longitarsis* (Macquart, 1838)

Timon-David 1951, **AA**, Zagora; Mouna 1998; Geller-Grimm 2007; Hayat et al. 2008; Koçak and Kemal 2010; El Hawagry 2011; Ghahari et al. 2014

﻿***Engelepogon* Lehr, 1992**

﻿*Engelepogon ﻿﻿﻿brunnipes* (Fabricius, 1794)

= *Heligmoneura ﻿﻿﻿brunnipes* Fabricius, in Séguy 1930a: 125

= *Acanthopleura ﻿﻿﻿brunnipes* Fabricius, in Timon-David 1951: 137

Becker and Stein 1913, **Rif**, Tanger; Séguy 1930a, **MA**, Meknès; Timon-David 1951, **MA**, Meknès; Mouna 1998; Geller-Grimm 2007; Koçak and Kemal 2010; Ghahari et al. 2014

﻿***Epitriptus* Loew, 1849**

﻿*Epitriptus ﻿﻿﻿cingulatus* (Fabricius, 1871)

Séguy 1941a, **AA**, Agadir; Mouna 1998

﻿***Eremisca* Hull, 1962**

﻿*Eremisca ﻿﻿﻿heleni heleni* (Efflatoun, 1934)

Geller-Grimm 2007; Hayat et al. 2008; Koçak and Kemal 2010; El Hawagry 2011; Ghahari et al. 2014

﻿*Eremisca ﻿﻿﻿osiris* (Wiedemann, 1828)

Geller-Grimm 2007; El Hawagry 2011

﻿***Eutolmus* Loew, 1848**

﻿*Eutolmuswahisi* Tomasovic, 2001

Tomasovic 2001a, **Rif**, Tétouan (Jebel Tazout, 1650 m); Geller-Grimm 2007; Koçak and Kemal 2010

﻿***Filiolus* Lehr, 1967**

﻿*Filiolus ﻿﻿﻿﻿﻿﻿﻿﻿﻿﻿apicalis* (Becker in Becker & Stein, 1913)

= ﻿*Eutolmus ﻿﻿﻿﻿﻿﻿﻿﻿﻿﻿apicalis* Becker, in Becker and Stein 1913: 75

Becker and Stein 1913, **Rif**, Tanger; Geller-Grimm 2007

﻿***Machimus* Loew, 1849**

﻿*Machimus ﻿﻿﻿cribratus* (Loew, 1849)

Geller-Grimm 2007; **AP** (Cap Cantia) – MISR

﻿*Machimus ﻿﻿﻿fimbriatus* (Meigen, 1804)

Geller-Grimm 2007

﻿*Machimus ﻿﻿﻿fortis* (Loew, 1849)

Becker and Stein 1913, **Rif**, Tanger; Séguy 1930a, **AP**, Rabat; Mouna 1998; Geller-Grimm 2007

﻿*Machimus ﻿﻿﻿gonatistes* (Zeller, 1840)

Geller-Grimm 2007

﻿*Machimusmauritanicus* Bequaert, 1964

Tomasovic 2003; Geller-Grimm 2007; **AP** (Forêt Boulhaut, Salé) – MISR

﻿*Machimus ﻿﻿﻿nigrosetosus* Séguy, 1941

Séguy 1941d**AA**, Agadir; Mouna 1998; Geller-Grimm 2007

﻿*Machimus ﻿﻿﻿perplexus* Becker, 1915

Geller-Grimm 2007

﻿*Machimus ﻿﻿﻿pilipes* (Meigen, 1820)

= ﻿*Eutolmus ﻿﻿﻿hispanus* Loew, in Becker and Stein 1913: 74

Becker and Stein 1913, **Rif**, Tanger; Séguy 1930a, **MA**, Forêt Zaers, Tizi-n’Bouftene (2300 m), **HA**, bords de l’Imminen (Tachdirt: 2400–2600 m); Geller-Grimm 2007

﻿*Machimus ﻿﻿﻿pseudogonatistes* Villeneuve, 1930

= ﻿*Machimus ﻿﻿﻿ermineus* Becker, in Mouna 1998: 84

Villeneuve 1933; Mouna 1998; Geller-Grimm 2007

﻿***Neoepitriptus* Lehr, 1992**

﻿*Neoepitriptus ﻿﻿﻿inconstans* (Wiedemann in Meigen, 1820)

= ﻿*Machimus ﻿﻿﻿micropyga* Becker, in Becker and Stein 1913: 74

Becker and Stein 1913, **Rif**, Tanger; Mouna 1998; Geller-Grimm 2007; El Hawagry 2011, **Rif**, Tanger

﻿*Neoepitriptus ﻿﻿﻿minusculus* (Bezzi, 1898)

= ﻿*Machimus ﻿﻿﻿minusculus* Bezzi, in Timon-David 1951: 138, Mouna 1998: 84

Timon-David 1951, **MA**, Ifrane; Mouna 1998; Geller-Grimm 2007

﻿***Neomochtherus* Osten-Sacken, 1878**

*Neomochterus ﻿﻿﻿brevipennis* Séguy, 1932

Mouna 1998; Geller-Grimm 2007; Koçak and Kemal 2010

﻿*Neomochtherus ﻿﻿﻿grandicollis* (Becker, 1914)

Becker and Stein 1913, **Rif**, Tanger; Geller-Grimm 2007

*Neomochterus ﻿﻿﻿ochriventris* (Loew, 1854)

Timon-David 1951, **AP**, Sidi Moussa el Harati; Mouna 1998; Geller-Grimm 2007; Koçak and Kemal 2010

﻿***Pashtshenkoa* Lehr, 1995**

﻿*Pashtshenkoa ﻿﻿clypeatus maroccanus* (Tsacas, 1968)

Geller-Grimm 2007

﻿***Phileris* Tsacas & Weinberg, 1976**

﻿*Phileris ﻿﻿﻿haplopygus* Tsacas & Weinberg, 1976

Geller-Grimm 2007

﻿*Phileris ﻿﻿﻿pilosus* Tsacas & Weinberg, 1976

Geller-Grimm 2007

﻿***Satanas* Jacobson, 1908**

﻿*Satanas ﻿﻿﻿gigas* (Eversmann, 1855)

Maldès 2000, **ME**, Oujda, **HA**, Errachidia, Meski

﻿***Turka* Őzdikmen, 2008**

﻿*Turka ﻿﻿﻿cervinus* (Loew, 1856)

= ﻿*Stenopogon ﻿﻿﻿cervinus* Loew, in Séguy 1930a: 122

Séguy 1930a, **MA**, pont de l’Oued Korifla (Zaers), **HA**, Sidi Bou Rziguine; Geller-Grimm 2007; Hayat et al. 2008; Özdikmen 2008; Koçak and Kemal 2010; El Hawagry 2011; Ghahari et al. 2014

##### Dasypogoninae

﻿***Dasypogon* Meigen, 1803**

﻿*Dasypogon ﻿﻿﻿atratus* (Fabricius, 1794)

= *Selidopogon ﻿﻿﻿atratus* Meigen, in Séguy 1930a: 118

= *Selidopogon ﻿﻿﻿atratus* Fabricius, in Timon-David 1951: 136

Séguy 1930a, **MA**; Timon-David 1951, **Rif**, Ouezzane, **AP**, Rabat **MA**, Oued Beth; Mouna 1998; Geller-Grimm 2007

﻿*Dasypogon ﻿﻿﻿auripilus* (Séguy, 1934)

Mouna 1998; Geller-Grimm 2007; **AP** (Casablanca) – MISR

﻿*Dasypogon ﻿﻿﻿crassus* Macquart in Lucas, 1849

= *Selidopogon ﻿﻿﻿crassus* Macquart, in Séguy 1930a: 119, Timon-David 1951: 136

Séguy 1930a, **Rif**, Tanger, **MA**, Meknès; Timon-David 1951, **AP**, M’Soun, Guerrouaou; Mouna 1998; Geller-Grimm 2007

﻿*Dasypogon ﻿﻿﻿diadema* (Fabricius, 1781)

= *Selidopogon ﻿﻿﻿cylindricus* Fabricius, in Séguy 1930a: 119

= *Selidopogon ﻿﻿﻿diadema* Fabricius, in Séguy 1930a: 118

= *Selidopogon ﻿﻿﻿sicanus* Costa, 1853, in Hayat et al. 2008: 183

Séguy 1930a, **AP**, Dar Salem, Tarfaya, Oued Korifla (Zaers), **HA**, Bou Tazzert; Timon-David 1951, **AP**, Port Lyautey; Mouna 1998; Geller-Grimm 2007; Hayat et al. 2008; Koçak and Kemal 2010; Ghahari et al. 2014

﻿*Dasypogon ﻿﻿﻿gougeleti* (Bigot, 1878)

= *Selidopogon ﻿﻿﻿gougeleti* Bigot, in Timon-David 1951: 136

Becker and Stein 1913, **Rif**, Tanger; Timon-David 1951, **AP**, Oued Korifla; Mouna 1998; Geller-Grimm 2007

﻿*Dasypogon ﻿﻿﻿olcesci* (Bigot, 1878)

Becker and Stein 1913, **Rif**, Tanger; Geller-Grimm 2007

﻿*Dasypogon ﻿﻿﻿rubinipes* (Becker in Becker & Stein, 1913)

Becker and Stein 1913, **Rif**, Tanger; Geller-Grimm 2007

﻿*Dasypogon ﻿﻿﻿ruficauda* (Fabricius, 1805)

Geller-Grimm 2007

﻿***Saropogon* Loew, 1847**

﻿*Saropogon ﻿﻿﻿aretalogus* Séguy, 1953

Séguy 1953a, **MA**, Ifrane; Geller-Grimm 2007

﻿*Saropogon ﻿﻿﻿aurifrons* (Macquart in Lucas, 1850)

Timon-David 1951, **AP**, Zaers; Mouna 1998; Geller-Grimm 2007; El Hawagry 2011

﻿*Saropogon ﻿﻿﻿clausus* Becker, 1906

Becker and Stein 1913, **Rif**, Tanger; Séguy 1930a, **EM**, Itzer, Moulay Aïn Djemine (Haute Moulouya); Timon-David 1951, **AP**, Zaers; Mouna 1998; Geller-Grimm 2007; Koçak and Kemal 2010; Ghahari et al. 2014

﻿*Saropogon ﻿﻿﻿jugulum* (Loew, 1847)

Timon-David 1951, **AP**, Zaers; Mouna 1998; Geller-Grimm 2007; Hayat et al. 2008; Koçak and Kemal 2010; El Hawagry 2011; Ghahari et al. 2014

﻿*Saropogon ﻿﻿﻿leucocephalus* (Meigen, 1820)

Séguy 1930a, **MA**, Forêt Tiffert (2000–2200 m); Mouna 1998; Geller-Grimm 2007; El Hawagry 2011; Ghahari et al. 2014

﻿*Saropogonmaroccanus* Séguy, 1930

Séguy 1930a, **MA**, Ras El Ksar (1900 m); Séguy 1949a, **SA**, Goulimine; Mouna 1998; Carles-Tolrá 2002; Geller-Grimm 2007

﻿*Saropogon ﻿﻿﻿obscuripennis* (Macquart in Lucas, 1849)

Becker and Stein 1913, **Rif**, Tanger; Séguy 1930a, **AP**, Rabat, **MA**, Aïn Leuh, Tizi-s’Tkrine (1700 m), **HA**, Imi-M’Tanout, Dar M’Tougui; Séguy 1941d, **AA**, Agadir; Timon-David 1951, **EM**, Guenfouda; Mouna 1998; Geller-Grimm 2007

﻿*Saropogon ﻿﻿﻿philocalus* Séguy, 1941

Séguy 1941d, **AA**, Agadir; Mouna 1998; Geller-Grimm 2007

﻿*Saropogon ﻿﻿﻿rufipes* (Gimmerthal, 1847)

Becker and Stein 1913, **Rif**, Tanger; Geller-Grimm 2007

﻿*Saropogon ﻿﻿﻿tassilaensis* Séguy, 1953

Séguy 1953a, **AA**, Tassila (Souss); Geller-Grimm 2007

##### Dioctriinae

﻿***Dioctria* Meigen, 1803**

﻿*Dioctria ﻿﻿﻿atrorubens* Séguy, 1930

Séguy 1930a, **MA**, Tizi-s’Tkine (1700 m); Villeneuve 1933; Mouna 1998; Geller-Grimm 2007

﻿*Dioctria ﻿﻿﻿cothurnata* Meigen, 1820

Ebejer et al. 2019, **Rif**, Dardara (484 m)

﻿*Dioctria ﻿﻿﻿fuscipes* Macquart, 1834

Timon-David 1951, **MA**, Aguelmane Sidi Ali (2070 m)

﻿*Dioctria ﻿﻿﻿gagates* Wiedemann in Meigen, 1820

Becker and Stein 1913, **Rif**, Tanger; Geller-Grimm 2007

﻿*Dioctria ﻿﻿﻿notha* Séguy, 1941

Séguy 1941d, **AA**, Agadir; Mouna 1998; Geller-Grimm 2007

﻿*Dioctria ﻿﻿﻿rufa* Strobl, 1906

Ebejer et al. 2019, **Rif**, Dardara (484 m)

﻿*Dioctria ﻿﻿﻿rungsi* Timon-David, 1951

Timon-David 1951, **MA**, Ifrane (1650 m); Mouna 1998; Geller-Grimm 2007

##### Laphriinae

﻿***Glyphotriclis* Hermann, 1920**

﻿*Glyphotriclis ﻿﻿﻿ornatus* (Schiner, 1868)

= *Triclis ﻿﻿﻿ornatus* Schiner, in Becker and Stein 1913: 67

Becker and Stein 1913, **Rif**, Tanger; Séguy 1930a, **HA**, Marrakech; Mouna 1998; Geller-Grimm 2007; El Hawagry 2011

﻿***Laphria* Meigen, 1803**

﻿*Laphria ﻿﻿﻿bomboides* Macquart, 1849

= ﻿*Laphriapraelusia* Séguy, in Séguy 1930a: 124

Séguy 1930a, **MA**, Soufouloud (1900–2100 m); Mouna 1998; **MA** (Meghraona, Tamtraekt) – MISR

﻿***Pogonosoma* Rondani, 1856**

﻿*Pogonosoma ﻿﻿﻿maroccanum* (Fabricius, 1794)

Loew 1860; Becker and Stein 1913, **Rif**, Tanger; Mouna 1998; Carles-Tolrá 2002; Geller-Grimm 2004; Geller-Grimm 2007; Ghahari et al. 2007; Koçak and Kemal 2010; Koçak and Kemal 2013a; Ghahari et al. 2014

﻿***Stiphrolamyra* Engel, 1928**

﻿*Stiphrolamyra ﻿﻿﻿rubicunda* Oldroyd, 1947

Timon-David 1951, **AP**, Sidi Moussa el Harati; Mouna 1998; Geller-Grimm 2007; El Hawagry 2011; Ghahari et al. 2014

﻿*Stiphrolamyra ﻿﻿﻿vitai* Hradský & Geller-Grimm, 1997

Hradský and Geller-Grimm 1997, **HA**, Taroudant; Geller-Grimm 2007

##### Laphystiinae

﻿***Perasis* Hermann, 1905**

﻿*Perasis ﻿﻿﻿sareptana* Hermann, 1906

Séguy 1930a, **HA**, Asni; Mouna 1998

﻿***Scytomedes* Röder, 1882**

﻿*Scytomedes ﻿﻿﻿haemorrhoidalis* (Fabricius, 1794)

= *Triclishaemorrhoidalis* Fabricius, in Mouna 1998: 84

Séguy 1930a, **MA**, Meknès; Mouna 1998; Geller-Grimm 2007; Hayat et al. 2008; Koçak and Kemal 2010; Ghahari et al. 2014

﻿***Trichardis* Hermann, 1906**

﻿*Trichardis ﻿﻿﻿leucocomus* (Van der Wulp, 1899)

= ﻿*Trichardis ﻿﻿﻿leucicoma* Van der Wulp, in Timon-David 1951: 132

Timon-David 1951, **AA**, Tata, piste de Fask Tahrjicht; Mouna 1998; Geller-Grimm 2007; Hayat et al. 2008; Koçak and Kemal 2010; El Hawagry 2011; Ghahari et al. 2014

##### Leptogastrinae

﻿***Leptogaster* Meigen, 1803**

﻿*Leptogaster ﻿cylindrica* (De Geer, 1776)

= ﻿*Leptogaster ﻿﻿﻿hispanica* Meigen, in Séguy 1930a: 117

Séguy 1930a, **MA**, Meknès; Mouna 1998; Tomasovic 2006, **Rif**; Geller-Grimm 2007; Hayat et al. 2008; Koçak and Kemal 2010; Ghahari et al. 2014

﻿*Leptogaster ﻿﻿﻿pedunculata* Loew, 1847

= *Gonypes ﻿﻿﻿pedunculatus* Loew, in Becker and Stein 1913: 72

Becker and Stein 1913, **Rif**, Tanger; Séguy 1930a, **HA**, Haute Réghaya; Mouna 1998

﻿*Leptogasterstraminea* Becker, 1907

Timon-David 1951, **MA**, Aguelmane Sidi Ali (2070 m); Mouna 1998; Geller-Grimm 2007

##### Stenopogoninae

﻿***Afroholopogon* Londt, 1994**

﻿*Afroholopogon ﻿﻿﻿waltlii* (Meigen, 1838)

= ﻿*Heteropogon ﻿﻿﻿waltlii* Meigen, in Séguy 1930a: 123

Séguy 1930a, **MA**, Meknès; Mouna 1998; Geller-Grimm 2007; Koçak and Kemal 2010

﻿***Amphisbetetus* Hermann, 1906**

﻿*Amphisbetetus ﻿﻿﻿sexspinus* Tomasovic, 2008

Tomasovic and Weyer 2008, **AA**, Imsouane (Agadir); Geller-Grimm 2007

﻿***Ancylorhynchus* Berthold in Latreille, 1827**

*Ancylorrhyncus ﻿﻿﻿gummigutta* (Becker, 1906)

Séguy 1930a, **Rif**, Tanger; Mouna 1998; Geller-Grimm 2007; Koçak and Kemal 2010

*Ancylorrhyncus ﻿﻿﻿limbatus* (Fabricius, 1794)

Séguy 1930a, **MA**, Meknès, Timhadit (2000 m); Mouna 1998; Geller-Grimm 2007; Koçak and Kemal 2010

*Ancylorrhyncus ﻿﻿﻿vultur* Séguy, 1930

Séguy 1930a, **MA**, Timhadit (2000 m); Mouna 1998; Geller-Grimm 2007; Koçak and Kemal 2010

﻿***Eriopogon* Loew, 1847**

﻿*Eriopogon ﻿﻿﻿jubatus* Becker, 1906

Timon-David 1951, **Rif**, Tanger, **AP**, Rabat; Hradský and Hüttinger 1995, **AP**, Rabat; Mouna 1998; Geller-Grimm 2007; **AP** (Forêt Temara) – MISR

﻿*Eriopogon ﻿﻿﻿laniger* Meigen, 1804

= ﻿*Holopogonflavescens* Jaennicke, in Séguy 1930a: 123

Séguy 1930a, **HA**, Aguergour; Mouna 1998; Geller-Grimm 2007; Hayat et al. 2008; Koçak and Kemal 2010; Ghahari et al. 2014

﻿*Eriopogonspatenkai* Hradský & Hüttinger, 1995

Geller-Grimm 2007; Hradský and Hüttinger 1995, **MA**, Mishliffen

﻿***Galactopogon* Engel, 1929**

﻿*Galactopogon ﻿﻿﻿hispidus* Engel, 1929

Ebejer et al. 2019, **AA**, 23 km S of Rich (Errachidia, 2012 m)

﻿***Habropogon* Loew, 1847**

﻿*Habropogon ﻿﻿﻿aerivagus* (Séguy, 1953)

Séguy 1953a, **SA**, Aouletis

﻿*Habropogon ﻿﻿﻿appendiculatus* Schiner, 1867

Timon-David 1951, **AA**, Aïn Chaïb; Mouna 1998; Weinberg and Blasco-Zumeta 2004; Hradský and Geller-Grimm 2005; Geller-Grimm 2007; Koçak and Kemal 2010; El Hawagry 2011

﻿*Habropogon ﻿﻿﻿bacescui* Weinberg & Tsacas, 1973

Geller-Grimm 2007; Koçak and Kemal 2010

﻿*Habropogon ﻿﻿﻿distipilosus* Weinberg & Tsacas, 1973

Geller-Grimm 2007

﻿*Habropogon ﻿﻿﻿hauseri* Hradský & Geller-Grimm, 2005

Hradský and Geller-Grimm 2005, **HA**, Tizi-n’Test; Geller-Grimm 2007; Koçak and Kemal 2010

﻿*Habropogon ﻿﻿﻿odontophallus* Weinberg & Tsacas, 1973

Geller-Grimm 2007

﻿*Habropogon ﻿﻿﻿parappendiculatus* Weinberg & Tsacas, 1973

Hradský and Geller-Grimm 2005, **HA**, Aït Saoun; Geller-Grimm 2007; Kirk-Spriggs and McGregor 2009, **HA**; Koçak and Kemal 2010

﻿*Habropogon ﻿﻿﻿prionophallus* Weinberg & Tsacas, 1973

Geller-Grimm 2007

﻿*Habropogon ﻿﻿﻿pyrrhophaeus* Weinberg & Tsacas, 1973

Geller-Grimm 2007

﻿*Habropogon ﻿﻿﻿rubriventris* Macquart, 1849

Becker and Stein 1913, **Rif**, Tanger; Séguy 1930a, **AP**, Aïn el Hadjar (near Mogador), **MA**, Meknès, Tlet n’Rhohr, **EM**, Berkane (1350–1400 m); Mouna 1998

﻿*Habropogon ﻿﻿﻿senilis* Wulp, 1899

Geller-Grimm 2007

﻿*Habropogon ﻿﻿﻿spissipes* Hermann, 1909

Geller-Grimm 2007; Koçak and Kemal 2010; El Hawagry 2011; Ghahari et al. 2014

﻿*Habropogon ﻿﻿﻿striatus* (Fabricius, 1794)

= ﻿*Habropogon ﻿﻿﻿heteroneurus* Timon-David, in Timon-David: 135

Séguy 1941d, **AA**, Agadir; Mouna 1998; Geller-Grimm 2007; El Hawagry 2011, **AP**, Rabat

﻿***Heteropogon* Loew, 1847**

﻿*Heteropogon ﻿﻿﻿biplex* Becker, in Becker & Stein 1913: 65

Becker and Stein 1913, **Rif**, Tanger; Geller-Grimm 2007

﻿*Heteropogon ﻿﻿﻿manicatus* (Meigen, 1820)

Séguy 1930a, **MA**, Azrou, Meknès, Aïn Leuh, **HA**, Asni; Mouna 1998; Geller-Grimm 2007; Koçak and Kemal 2010; **MA** (Ifrane) – MISR

﻿*Heteropogon ﻿﻿﻿nubilus* (Meigen, 1820)

= *Isopogon ﻿﻿﻿brevis* Schiner, in Becker and Stein 1913: 64

= ﻿*Sisyrnodytes ﻿﻿﻿brevis* Macquart, in Timon-David 1951: 134, Séguy 1953a: 79

Becker and Stein 1913, **Rif**, Tanger; Séguy 1953a, **AA**, Imiter; Mouna 1998; Geller-Grimm 2007; Koçak and Kemal 2010; Shoeibi and Karimpour 2010; Ghahari et al. 2014

﻿***Holopogon* Loew, 1847**

﻿*Holopogon ﻿﻿﻿dimidiatus* (Meigen, 1820)

Séguy 1941d, **AA**, Agadir

﻿*Holopogon ﻿﻿﻿dusmeti* Strobl in Czerny & Strobl 1909

= ﻿*Eriopogon ﻿﻿﻿dusmeti* Strobl, in Timon-David 1951: 132

Timon-David 1951, **EM**, Guenfouda, **HA**, Tifni; Mouna 1998; Geller-Grimm 2007; **HA** (Tifni Demnat) – MISR

﻿*Holopogon ﻿﻿﻿melaleucus* (Meigen, 1820)

Séguy 1930a, **AP**, Forêt Maâmora, Dar Salem (Rabat); Séguy 1941d, **AA**, Agadir; Mouna 1998; Geller-Grimm 2007

﻿*Holopogon ﻿﻿﻿pusillus* (Macquart, 1838)

= ﻿*Habropogon ﻿﻿﻿pusillus* (Macquart), in Séguy 1949a: 154

Séguy 1949a, **SA**, Goulimine; Mouna 1998

﻿*Holopogon ﻿﻿﻿quadrinotatus* Séguy, 1953

Séguy 1953a, **SA**, Amguilli Sguelma

﻿***Acnephalum* Macquart, 1838**

= ﻿*Pycnopogon* Loew, 1847 in Londt 2010

﻿*Acnephalum ﻿﻿﻿apiformis* (Macquart in Lucas, 1849)

Séguy 1930a, **MA**, Timhadit (2000 m), Meskedall (1800–1900 m); Timon-David 1951, **MA**, Ifrane (1650 m); Mouna 1998; Geller-Grimm 2007

﻿*Acnephalum ﻿﻿﻿denudatus* (Séguy, 1949)

= ﻿*Stenopogon ﻿﻿﻿denudatus* Loew, in Séguy 1930a: 123, Séguy 1934b: 162

Séguy 1930a, **MA**, Tizi-n’Tkrine; Séguy 1934b, **HA**, Haute Réghaya; Séguy 1949b, **AP**, Bou Tazzert near Mogador; Séguy 1953a, **AA**, Oasis du Ferkla; Mouna 1998; Geller-Grimm 2007

﻿*Acnephalum ﻿﻿﻿fasciculatus* (Loew, 1847)

Séguy 1930a, **MA**, Azrou, Timelilt, Sidi Bettache, **HA**, Asni, bords Imminen (Tachdirt), Likount (2500–2800 m), Lac Ifni (Skoutana), **SA**, Béni Mgild; Timon-David 1951, **AP**, Oued Korifla, **MA**, Lac Aguelmane Sidi Ali (2070 m), Oued N’Zala; Mouna 1998; Geller-Grimm 2007; Lehr et al. 2007; Koçak and Kemal 2010; Ghahari et al. 2014; **MA** (Ras el Ma) – MISR

﻿***Stenopogon* Loew, 1847**

﻿*Stenopogon ﻿﻿﻿costatus* Loew, 1871

= ﻿*Stenopogon ﻿﻿﻿costarus* Loew, in Mouna 1998: 84

Séguy 1930a, **MA**, Tizi-n’Tkrine (Jebel Ahmar, 1700 m); Mouna 1998

﻿*Stenopogon ﻿﻿﻿gracilis* (Macquart, 1838)

= ﻿*Stenopogonfumipenis* Becker, in Becker and Stein 1913: 68

Becker and Stein 1913, **Rif**, Tanger; Geller-Grimm 2007

﻿*Stenopogon ﻿﻿﻿heteroneurus* (Macquart, 1838)

Timon-David 1951, **AP**, Forêt Maâmora, Oued Akreuch, **HA**, Mouldikht; Mouna 1998; Hayat et al. 2008; Geller-Grimm 2007; Koçak and Kemal 2010; El Hawagry 2011; Ghahari et al. 2014

﻿*Stenopogon ﻿﻿﻿iphippus* Séguy, 1932

Séguy 1932b, **MA**, Volubilis; Mouna 1998; Geller-Grimm 2007

﻿*Stenopogon ﻿﻿﻿iphis* Séguy, 1932

Séguy 1932b, **MA**, Azrou; Timon-David 1951, **Rif**, Plateau de Tisserouine (2000 m), **MA**, Ifrane (1650 m), Ito (Rabat); Geller-Grimm 2007

﻿*Stenopogon ﻿﻿﻿ischyrus* Séguy, 1932

Séguy 1932b, **MA**, Tizi-s’Tkrine (Jebel Ahmar, 1700 m); Mouna 1998; Geller-Grimm 2007

﻿*Stenopogon ﻿﻿﻿junceus* (Wiedemann in Meigen, 1820)

Timon-David 1951, **AP**, Oued Akreuch, Zaër, **MA**, Sefrou; Mouna 1998; Geller-Grimm 2007; Hayat et al. 2008; Koçak and Kemal 2010; Ghahari et al. 2014

﻿*Stenopogon ﻿﻿﻿kocheri* Timon-David, 1951

Timon-David 1951, **HA**, Tifni; Mouna 1998; Geller-Grimm 2007

﻿*Stenopogon ﻿﻿﻿porcus* Loew, 1871

Séguy 1949a, **AA**, Akka; Mouna 1998; Geller-Grimm 2007

﻿*Stenopogon ﻿﻿﻿werneri* Engel, 1933

Geller-Grimm 2007; Koçak and Kemal 2010, **MA**, Fès, Zalagh

﻿***Sisyrnodytes* Loew, 1856**

﻿*Sisyrnodytes ﻿﻿﻿leucophaetus* Séguy, 1930

Séguy 1930a, **MA**, Béni Berberi; Mouna 1998; Geller-Grimm 2007; Londt 2009

﻿*Sisyrnodytes ﻿﻿﻿nilicola* (Rondani, 1850)

Oldroyd 1980; Londt 1987; Geller-Grimm 2007; Londt 2009, **AA**, Ifni, Tiznit, Tata, Tazegzout; El Hawagry 2011; Samin et al. 2011; Ghahari et al. 2014

##### Stichopogoninae

﻿***Stichopogon* Loew, 1847**

﻿*Stichopogon ﻿﻿﻿albellus* Loew, 1856

Séguy 1949a, **AA**, Foum-el-Hassan; Mouna 1998

﻿*Stichopogon ﻿﻿﻿albofasciatus* (Meigen, 1820)

Séguy 1930a, **HA**, Kasba Taguendaft (Goundafa); Mouna 1998; Geller-Grimm 2007

﻿*Stichopogon ﻿﻿﻿elegantulus* (Wiedemann, 1820)

Séguy 1930a, **HA**, Kasba Taguendaft (Goundafa), Aguerd el Had, **AA**, Talekjount (Souss); Mouna 1998; Geller-Grimm 2007; Ghahari et al. 2007; Koçak and Kemal 2010; El Hawagry 2011; Ghahari et al. 2014; **AP** (Kénitra) – MISR

﻿*Stichopogon ﻿﻿﻿inaequalis* Loew, 1847

Séguy 1930a, **HA**, Aguerd el Had, **AA**, Talekjount (Souss); Mouna 1998; Geller-Grimm 2007

﻿*Stichopogonmaroccanus* (Becker, 1914)

Becker and Stein 1913, **Rif**, Tanger; Geller-Grimm 2007; El Hawagry 2011, **Rif**, Tanger

﻿*Stichopogon ﻿﻿﻿punctiferus* Bigot, 1878

Geller-Grimm 2007

﻿*Stichopogon ﻿﻿﻿pusio* (Macquart in Lucas, 1849)

Séguy 1930a, **HA**, Kasba Taguendaft (Goundafa); Mouna 1998; Geller-Grimm 2007; El Hawagry 2011

﻿*Stichopogon ﻿﻿﻿schineri* Koch, 1872

Timon-David 1951, **AA**, Backkoum (Jebel Siroua); Mouna 1998; Hayat et al. 2008; Geller-Grimm 2007; Koçak and Kemal 2010; Ghahari et al. 2014

#### ﻿﻿BOMBYLIIDAE

K. Kettani, M.J. Ebejer, J. Dils

Number of species: **248**. Expected: 270

Faunistic knowledge of the family in Morocco: good

##### Usiinae

﻿***Apolysis* Loew, 1860**

﻿*Apolysis ﻿﻿﻿eremophila* Loew, 1873

= ﻿*Usia ﻿﻿﻿tomentosa* Engel, in Paramonow 1947: 209

= ﻿*Parageron ﻿﻿﻿ornata* Engel, in Zaitzev 2007: 160

Paramonow 1947; Mouna 1998; Zaitzev 2007, **AP** (south), Tamri; Koçak and Kemal 2010; El Hawagri 2011; Evenhuis and Greathead 2015

﻿***Parageron* Paramonov, 1929**

﻿*Parageron ﻿﻿﻿gratus* (Loew, 1856)

= ﻿*Usia ﻿﻿﻿grata* Loew, in Timon-David 1951: 143

Timon-David 1951, **AP**, Oued Grou; Mouna 1998; Bader and Arabyat 2004; Zaitzev 2007, **SA**; Koçak and Kemal 2010; El Hawagri 2011; Evenhuis and Greathead 2015 – MISR

﻿*Parageron ﻿﻿﻿griseus* Paramonov, 1947

Zaitzev 2007, **SA**

﻿*Parageron ﻿﻿﻿hyalipennis* (Séguy, 1941)

= *Oligodranes ﻿﻿﻿hyalipennis* Séguy, in Séguy 1941d: 9

Séguy 1941d, **AA**, Agadir (Forêt Admine); Mouna 1998; Koçak and Kemal 2010; Evenhuis and Greathead 2015, **HA**

﻿*Parageron ﻿﻿﻿incisus* (Wiedemann, 1830)

= ﻿*Usia ﻿﻿﻿incisa* Wiedemann, in Timon-David 1951: 143

Séguy 1930a, **AP**, Mogador, Casablanca, Sidi Bettache, Aïn Sferguila, **MA**, Forêt Zaers, Ras el Ma, Aïn Leuh, **HA**, Tenfecht; Timon-David 1951, **AP**, Rabat, Sehoul, **MA**, Ifrane; Mouna 1998; Koçak and Kemal 2010; Evenhuis and Greathead 2015

﻿*Parageron ﻿﻿﻿major* Macquart, 1840

Mouna 1998; Pârvu and Zaharia 2007; Zaitzev 2007, **AP**, Rabat, Nkheila; Evenhuis and Greathead 2015

﻿***Usia* Latreille, 1802**

﻿*Usia (Micrusia) aurata* (Fabricius, 1794)

= ﻿*Usia (Micrusia) ﻿taeniolata* Costa, 1883, in, Koçak and Kemal 2010

Séguy 1930a, **Rif**, Tanger, **AP**, Rabat, Sidi Bettache; Paramonov 1950, **Rif**, Tanger; Mouna 1998; Koçak and Kemal 2010; El Hawagry 2011; Evenhuis and Greathead 2015

﻿*Usia (Micrusia) ﻿crispa* Gibbs, 2011

Gibbs 2011, **HA**, Marrakech, **AA**, Agadir, Taroudant, Tafraoute, Ouarzazate

﻿*Usia (Micrusia) ﻿cryptocrispa* Gibbs, 2011

Gibbs 2011, **AP**, Ben Slimane, Rabat

﻿*Usia (Micrusia) ﻿dilsi* Gibbs, 2011

Gibbs 2011, **Rif**, Tétouan, Al Hoceima, **HA**, Taourirt

﻿*Usia (Micrusia) echinus* Gibbs, 2011

Gibbs 2011, **AP**, Agadir, Guelmim, Sidi Ifni, Cap Ghir, **MA**, Tafraoute, **HA**, Marrakech, Tizi-n-Test, **AA**, Tafingoult

﻿*Usia (Micrusia) ﻿falcata* Gibbs, 2011

Gibbs 2011, **MA**, Azrou, Ifrane

﻿*Usia (Micrusia) ﻿forcipata* Brullé, 1833^14^

Mouna 1998: 84

﻿*Usia (Micrusia) ﻿globicauda* Gibbs, 2011

Gibbs 2011, **AP**, Essaouira

﻿*Usia (Micrusia) ﻿loewi* Becker, 1906

Zaitzev 2007, **Rif**, Tanger, **AP**, Skhirate, Nkheila, **MA**, Taferiate, **HA**, Taourirt

﻿﻿*Usia (Micrusia) ﻿novakii* Strobl, 1902

Séguy 1941d, **HA**, Tizi-n’Test; Mouna 1998; Koçak and Kemal 2010; Evenhuis and Greathead 2015

﻿*Usia (Micrusia) ﻿parascripa* Gibbs, 2011

Gibbs 2011, **MA**, Ifrane, Mischliffen (2200 m)

﻿*Usia (Micrusia) ﻿pusilla* Meigen, 1820

Séguy 1930a, **AP**, Rabat, **MA**, Azrou, **HA**, Tafingoult (Goundafa, 1500–1600 m); Séguy 1934b, **AP**, Rabat (on *Calendula*); Séguy 1953a, **AP**, Cap Ghir; Mouna 1998; Koçak and Kemal 2010; Evenhuis and Greathead 2015

﻿*Usia (Micrusia) versicolor* (Fabricius, 1787)

Séguy 1930a, **AP**, Berrchid, Casablanca, M’Rassine; Timon-David 1951, **AP**, Rabat, **MA**, Oulmès; Mouna 1998; Koçak and Kemal 2010; Gibbs 2011, **HA**; Evenhuis and Greathead 2015; **EM** (Oujda) – MNHNR

﻿*Usia (Usia*) ﻿﻿﻿*aenea* (Rossi, 1794)

Séguy 1949a, **AA**, Foum-el-Hassan; Mouna 1998; Bader and Arabyat 2004; Koçak and Kemal 2010; Evenhuis and Greathead 2015

﻿*Usia (Usia*) ﻿﻿﻿*angustifrons* Becker, 1906

Mouna 1998

﻿*Usia (Usia) ﻿atrata* (Fabricius, 1798)

= *Voluccella ﻿﻿﻿atrata* Fabricius, in Fabricius 1798: 570

= ﻿*Usia ﻿﻿﻿claripennis* Macquart, in Macquart 1840: 105

Meigen 1820; Séguy 1930a, **Rif**, Tanger, **MA**, Aïn Leuh; Timon-David 1951, **AP**, Rabat, Guerrouaou; Mouna 1998; Zaitzev 2007, **HA**, Tizi-n’Tichka; Koçak and Kemal 2010; Gibbs 2014, **AP**, Mogador, Arbaa-Sahel (320 m), Tamri (215 m), **MA**, Khemisset, Oulmès (700 m), Ifrane, El Merabtine, **HA**, Marrakech, Aït Ourirr (530 m), Oukaimeden (2200 m), Tizi-n’Test (1450 m), Timzit (1700 m), **AA**, Agadir, Tiznit, Igherm (1660 m), Taroudant, Tata, Iguiour (1260 m), **SA**, Bou Jarif, Goulimine; Evenhuis and Greathead 2015

﻿*Usia (Usia) ﻿bicolor* Macquart, 1855^15^

Mouna 1998: 84

﻿*Usia (Usia) ﻿cornigera* Gibbs, 2014

Gibbs 2014, **Rif**, Tanger, **AP**, Sidi Bettache, Rabat, **MA**, Meknès (550 m), Aïn Leuh (1350 m), **HA**, Dar Kaid M’tougui

﻿*Usia (Usia) ﻿florea* (Fabricius, 1794)

= ﻿*Volucella ﻿﻿﻿florea* Fabricius, in Becker 1906: 203

= ﻿*Usia ﻿﻿﻿cuprea* Macquart, 1834, in Becker 1906: 203

Becker 1906a; Séguy 1930a, **Rif**, Tanger, **AP**, Mogador, Sidi Bettache, **HA**, Tinmel (Goundafa), around (Skoutana); Timon-David 1951, **EM**, Oued Moulouya; Mouna 1998; Pârvu and Zaharia 2007; Koçak and Kemal 2010; Gibbs 2014; Evenhuis and Greathead 2015

﻿*Usia (Usia) ﻿ignorata* Becker, 1906

Becker 1906a; Mouna 1998; Bader and Arabyat 2004; Pârvu and Zaharia 2007; Koçak and Kemal 2010; El Hawagry 2011

﻿*Usia (Usia) maghrebensis* Gibbs, 2014

Gibbs 2014, **Rif**, Tanger, Tétouan, El Biutz (150 m), **AP**, Mogador, **MA**, Aïn Leuh (1350 m)

﻿*Usia (Usia) ﻿vestita* Macquart, 1846

Mouna 1998: 84; Gibbs 2011

##### Phthiriinae

﻿***Phthiria* Meigen, 1802**

﻿*Phthiria ﻿﻿﻿albogilva* Séguy, 1941

Séguy 1941d, **AA**, Agadir; Mouna 1998; Evenhuis and Greathead 2015

﻿*Phthiria ﻿﻿﻿gaedii* Wiedemann in Meigen, 1820

Séguy 1930a, **MA**, Foum Keneg; Timon-David 1951, **AP**, Zaers, **MA**, Ifrane; Mouna 1998; Koçak and Kemal 2010; El Hawagry 2011; Evenhuis and Greathead 2015 – MISR (**MA**, Ifrane)

﻿*Phthiria ﻿maroccana* Zaitzev, 2005

= ﻿*Phthiria ﻿maroccana* Zaitzev, in Zaitzev 2005: 667

Zaitzev 2005, **MA**, Taferiate, **HA**, Taourirt; Zaitzev 2007, **MA**, Taferiate, **HA**, Taourirt

﻿*Phthiria ﻿﻿﻿merlei* Zaitzev, 2005

= ﻿*Phthiria ﻿﻿﻿merlei* Zaitzev, in Zaitzev 2005: 665

Zaitzev 2005, **AP** (south), Tamri, Inchaden (south of Aït Melloul); Zaitzev 2007, **AP** (south), Tamri, Inchaden (south of Aït Melloul)

﻿*Phthiriaminuta* (Fabricius, 1805)

Séguy 1930a, **HA**, Tenfecht, **AA**, Souss; Mouna 1998; Koçak and Kemal 2010; El Hawagry 2011

﻿Phthiriapulicariavar.flavofasciata Strobl in Morge, 1976

Mouna 1998; Zaitzev 2007, **AA**, Tizi-n’Tiniggigt (1600 m); Koçak and Kemal 2010; Evenhuis and Greathead 2015

﻿*Phthiria ﻿﻿﻿scutellaris* Wiedemann in Meigen, 1820

Séguy 1930a, **MA**, Meknès; Séguy 1941a, **MA**, Meknès, **HA**, Imi-n’Ouaka (1500 m); Mouna 1998; Evenhuis and Greathead 2015; **Rif** (Sapinière Talassemtane) – MISR

﻿*Phthiria ﻿﻿﻿simonyi* Becker, 1908

Séguy 1949a, **SA**, Guelmim; Mouna 1998; Koçak and Kemal 2010; Evenhuis and Greathead 2015, **MA**, Meknès

﻿*Phthiria ﻿﻿﻿umbripennis* Loew, 1846

Mouna 1998; Koçak and Kemal 2010; Evenhuis and Greathead 2015, **MA**, Meknès

﻿*Phthiria ﻿﻿﻿vagans* Loew, 1846

Zaitzev 2007, **HA**, Taourirt, **AA**, Tizi-n’Taratine

##### Toxophorinae

﻿***Geron* Meigen, 1820**

﻿*Geron ﻿﻿﻿intonsus* Bezzi, 1925*

**MA**, **HA**

﻿*Geron ﻿﻿﻿macquarti* Greathead in Evenhuis & Greathead 1999

Dils and Özbek 2006; Koçak and Kemal 2010; El Hawagry 2011

﻿*Geron ﻿﻿﻿subflavofemoratus* Andréu Rubio, 1959

Andréu Rubio 1959; Mouna 1998; Koçak and Kemal 2010; Evenhuis and Greathead 2015

﻿***Toxophora* Meigen, 1803**

﻿*Toxophora ﻿﻿﻿fasciculata* (Villers, 1789)

Séguy 1930a, **AP**, Rabat; Mouna 1998; Dils and Özbek 2006; Koçak and Kemal 2010; El Hawagry 2011; Evenhuis and Greathead 2015; **AP** (Rabat) – MISR

﻿*Toxophora ﻿﻿﻿fuscipennis* (Macquart, 1840)

Mouna 1998: 84

﻿*Toxophora ﻿﻿﻿pauli* Zaitzev, 2005

Zaitzev 2005, **AA**, Ouarzazate, Jebel Tighermine (SE of Ouarzazate); Zaitzev 2007, **AA**, Ouarzazate, Jebel Tighermine (SE of Ouarzazate)

﻿*Toxophora ﻿﻿﻿shelkovnikovi* Paramonov, 1933

Zaitzev 2007, **AA**, Ouarzazate

##### Heterotropinae

﻿***Heterotropus* Loew, 1873**

﻿*Heterotropus ﻿﻿﻿atlanticus* Séguy, 1930

Séguy 1930a, **AP**, Mogador; Mouna 1998; Koçak and Kemal 2010; Evenhuis and Greathead 2015

﻿*Heterotropus ﻿﻿﻿longitarsus* Séguy, 1930

Koçak and Kemal 2010; Evenhuis and Greathead 2015

﻿*Heterotropusmaroccanus* Zaitzev, 2003

Zaitzev 2003, **AA**, Jebel Tighermine (SE of Ouarzazate); Zaitzev 2007, **AA**, Jebel Tighermine (SE of Ouarzazate)

##### Bombyliinae

﻿***Anastoechus* Osten-Sacken, 1877**

﻿*Anastoechus ﻿﻿﻿bahirae* Becker, 1915

Mouna 1998; Zaitzev 2007, **AA**, Jebel Tighermine (SE of Ouarzazate)

﻿*Anastoechus ﻿﻿﻿hyrcanus* Pallas & Wiedemann in Wiedemann, 1818

Mouna 1998: 84

﻿*Anastoechus ﻿﻿﻿latifrons* (Macquart, 1839)

Timon-David 1951, **AP**, Dradek; Koçak and Kemal 2010; Evenhuis and Greathead 2015

﻿Anastoechus ﻿nitidulus ssp. nitidulus Fabricius, 1794

Mouna 1998: 84

﻿*Anastoechus ﻿﻿﻿stramineus* Wiedemann in Meigen, 1820

Mouna 1998: 84

﻿*Anastoechus ﻿﻿﻿trisignatus* (Portschinsky, 1881)

Bader and Arabyat 2004; Ziatzev 2007, **AP**, Rabat, **AA**, Jebel Tighermine (SE of Ouarzazate), **AA**, 20 km SW Goulmima, **SA**, Tan-Tan, Between Guelmim and Tan-Tan (90 km from Guelmim), Taganint (south of Bou-Izakarn); Koçak and Kemal 2010; El Hawagry 2011; Evenhuis and Greathead 2015

﻿***Bombomyia* Greathead, 1995**

﻿*Bombomyia ﻿﻿﻿discoidea* (Fabricius, 1794)

Séguy 1930a, **AP**, Oued Korifla (Zaers), Sidi Bettache; Bader and Arabyat 2004; Dils and Özbek 2006; Koçak and Kemal 2010; Evenhuis and Greathead 2015

﻿*Bombomyia ﻿﻿﻿stictica* Boisduval, 1835

Zaitzev 2007, **MA**, col de Zeggota (N Meknès), Oulmès

﻿*Bombomyia ﻿﻿﻿vertebralis* (Dufour, 1833)

= ﻿*Bombylius ﻿﻿﻿punctatus* Fabricius, in Timon-David 1951: 144

Becker and Stein 1913, **Rif**, Tanger; Séguy 1930a**AP**, Dradek near Rabat; Timon-David 1951, **MA**, Volubilis; Mouna 1998; Bader and Arabyat 2004; [Bibr B612][Bibr B369]; **AP** (Dradek, Casablanca), **EM** (Oujda), **MA** (Volubilis, Aïn Leuh) – MISR

﻿***Bombylisoma* Rondani, 1856**

﻿*Bombylisoma ﻿﻿﻿algirum* (Macquart, 1840)

= ﻿*Bombylius ﻿﻿﻿nigrifrons* Becker, in Becker and Stein 1913: 83

Becker and Stein 1913, **Rif**, Tanger; Mouna 1998; Zaitzev 2007, **Rif**, Tanger; Koçak and Kemal 2010; Evenhuis and Greathead 2015

﻿*Bombylisoma ﻿﻿﻿breviusculum* (Loew, 1855)

Dils and Özbek 2006; Zaitzev 2007; Evenhuis and Greathead 2015

﻿*Bombylisoma ﻿﻿﻿flavibarbum* Loew, 1855

Mouna 1998: 84

﻿*Bombylisoma ﻿﻿﻿melanocephalum* Fabricius, 1794

Zaitzev 2007, **HA**, Taourirt, south of Tizi-n’Test

﻿***Bombylius* Linnaeus, 1758**

﻿*Bombylius (Bombylius) ﻿albaminis* Séguy, 1949

Séguy 1949a, **HA**, Alnif; Mouna 1998; Koçak and Kemal 2010; Evenhuis and Greathead 2015

﻿*Bombylius (Bombylius) ﻿ambustus* Pallas & Wiedemann, 1818

Mouna 1998: 84

﻿*Bombylius (Bombylius) ﻿﻿analis* (Olivier, 1789)

Becker and Stein 1913, **Rif**, Tanger; Séguy 1930a, **AP**, Oued Korifla, Rabat, Sidi Bettache, Aïn Sferguila; Timon-David 1951, **AP**, Rabat; Mouna 1998; Zaitzev 2007, **MA**, route Fès-Sidi Kacem (30 km from Fès); Koçak and Kemal 2010; Evenhuis and Greathead 2015, **AP** (Rabat, Casablanca) – MISR

﻿*Bombylius (Bombylius) ﻿audcenti* Bowden, 1984

Mouna 1998; Koçak and Kemal 2010; Evenhuis and Greathead 2015

﻿*Bombylius (Bombylius) ﻿canescens* Mikan, 1796

Becker and Stein 1913, **Rif**, Tanger; Mouna 1998; Zaitzev 2007, **Rif**, Cap Malabata (Tanger), **MA**, Tachguelt, route Fès-Sidi Kacem (30 km from Fès)

﻿*Bombylius (Bombylius) ﻿cinerascens* Mikan, 1796

Mouna 1998; Bader and Arabyat 2004

﻿*Bombylius (Bombylius) ﻿discolor* Mikan, 1796

Mouna 1998; Zaitzev 2007, **MA**, route El Hajeb-Ifrane (1 km from Ifrane)

﻿*Bombylius (Bombylius) ﻿eploceus* Séguy, 1949

Séguy 1949a, **SA**, Guelmim; Mouna 1998; Evenhuis and Greathead 2015

﻿*Bombylius (Bombylius) ﻿fimbriatus* Meigen, 1820

Becker and Stein 1913, **Rif**, Tanger; Séguy 1930a, **MA**, Tizi-s’Tkrine, Jebel Ahmar (1700 m); Mouna 1998; Pârvu and Zaharia 2007; Zaitzev 2007; Koçak and Kemal 2010; El Hawagry 2011; Evenhuis and Greathead 2015

﻿*Bombylius (Bombylius) flavipes* Wiedemann, 1828

Becker and Stein 1913, **Rif**, Tanger; Mouna 1998; Zaitzev 2007; Koçak and Kemal 2010; El Hawagry 2011; Evenhuis and Greathead 2015

﻿*Bombylius (Bombylius) ﻿fulvescens* Wiedemann in Meigen, 1820

Becker and Stein 1913, **Rif**, Tanger; Séguy 1953a, **AP**, Cap Ghir; Séguy 1941d, **AA**, Agadir; Mouna 1998

﻿*Bombylius (Bombylius) ﻿fuscus* Fabricius, 1781

Mouna 1998: 84

﻿*Bombylius (Bombylius) ﻿major* (Linnaeus, 1758)

Becker and Stein 1913, **Rif**, Tanger; Séguy 1953a, **MA**, Oulmès; Bader and Arabyat 2004; Zaitzev 2007; Koçak and Kemal 2010; El Hawagry 2011; Evenhuis and Greathead 2015; **AP** (Kénitra) – MISR

﻿*Bombylius (Bombylius) ﻿mauritanus* Olivier, 1789

Koçak and Kemal 2010; Evenhuis and Greathead 2015, **HA**

﻿*Bombylius (Bombylius) ﻿medius* (Linnaeus, 1758)

Becker and Stein 1913, **Rif**, Tanger; Timon-David 1951, **AP**, Sehoul; Mouna 1998; Bader and Arabyat 2004; Dils and Özbek 2006; Pârvu and Zaharia 2007; Zaitzev 2007; Koçak and Kemal 2010; El Hawagry 2011; Evenhuis and Greathead 2015; **AP** (Oued Yquem, Dradek) – MISR

﻿*Bombylius* (Unplaced) ﻿*megacephalus* Portschinsky, 1887*

**EM**, **AA**

﻿*Bombylius (Bombylius) minor* Linnaeus, 1758

Mouna 1998; Zaitzev 2007, **Rif**, Tanger; **MA** (Aguelmane Azigza), **SA**

– MISR

﻿*Bombylius (Bombylius) ﻿mus* Bigot, 1862

Koçak and Kemal 2010; El Hawagry 2011; Evenhuis and Greathead 2015

﻿*Bombylius (Bombylius) ﻿niveus* Meigen, 1804

Mouna 1998: 84; **AP** (Mogador) – MISR

﻿*Bombylius (Bombylius) ﻿numidus* Macquart, 1846

Séguy 1953a, **MA**, Ifrane; El Hawagry 2011; Evenhuis and Greathead 2015

﻿*Bombylius (Bombylius) ﻿pauli* Zaitzev, 2003

Zaitzev 2003, **MA**, route Fès-Sidi Kacem (30 km from Fès); Zaitzev 2007, **MA**, route Fès-Sidi Kacem (30 km from Fès)

﻿*Bombylius (Bombylius) ﻿posticus* (Fabricius, 1805)

Dils and Özbek 2006; Zaitzev 2007; Koçak and Kemal 2010; El Hawagry 2011; Evenhuis and Greathead 2015

﻿*Bombylius (Bombylius) ﻿postversicolor* Evenhuis & Greathead, 1999

= ﻿*Bombyliusversicolor* Fabricius, 1805

Meigen 1820; Bezzi 1906; Séguy 1930a, **AP**, Mogador; Koçak and Kemal 2010; Evenhuis and Greathead 2015

﻿*Bombylius (Bombylius) ﻿pumilus* Meigen, 1820

Mouna 1998: 84

﻿*Bombylius (Bombylius) ﻿semifuscus* (Meigen, 1820)

Séguy 1953a, **AP**, Cap Ghir; Koçak and Kemal 2010; Evenhuis and Greathead 2015

﻿*Bombylius (Bombylius) ﻿torquatus* Loew, 1855

Séguy 1930a, **HA**, Ouaounzert (Glaoua), Arround (Skoutana), Tachdirt (bord de l’Imminen, 2400–2600 m); Timon-David 1951, **AP**, Rabat; Mouna 1998; Evenhuis and Greathead 2015; **AP** (Mogador) – MISR

﻿*Bombylius (Bombylius) ﻿undatus* Mikan, 1796

Pârvu and Zaharia 2007

﻿*Bombylius (Bombylius) ﻿vagans* Meigen, 1830

Koçak and Kemal 2010; Evenhuis and Greathead 2015

﻿*Bombylius (Bombylius) ﻿venosus* Mikan, 1796

Mouna 1998; Zaitzev 2007, **AP**, El Koudia (30 km SW from Rabat); **AP** (Dradek) – MISR

﻿*Bombylius (Zephyrectes) ﻿cruciatus* Fabricius, 1798

Séguy 1930a, **MA**, Aharmoumou (1100 m), Azrou, Ras el Ma, **HA**, Tizi-n’Test, Jebel Imdress (Goundafa, 2000–2450 m); Mouna 1998; Zaitzev 2007; Koçak and Kemal 2010; Evenhuis and Greathead 2015; **Rif** (Talassemtane), **AP** (Mogador), **MA** (Sefrou) – MISR

﻿*Bombylius (Zephyrectes) ﻿leucopygus* (Macquart, 1846)

Becker and Stein 1913, **Rif**, Tanger; Mouna 1998; Zaitzev 2007, **AP**, Larache, **MA**, Moulay Idris (900 m); Evenhuis and Greathead 2015, **SA**, Erfoud; **MA** (Ifrane) – MISR

﻿***Conophorus* Meigen, 1803**

﻿*Conophorus ﻿﻿﻿bellus* Becker, 1906*

**HA**

﻿*Conophorus ﻿﻿﻿fuliginosus* (Wiedemann in Meigen, 1820)

= *Ploas ﻿﻿﻿fuliginisa* (Meigen), in Séguy 1953a: 83

Séguy 1930a, **MA**, Aharmoumou (1100 m); Séguy 1953a, **MA**, Ahermoumou (1100 m); Timon-David 1951, **AP**, Dradek, **MA**, Sefrou, **HA**, Marrakech; Mouna 1998; Zaitzev 2007, **Rif**, Tanger; Evenhuis and Greathead 2015; **AP** (Salé, Mogador) – MISR

﻿*Conophorus ﻿﻿﻿fuscipennis* (Macquart, 1840)

Séguy 1930a, **HA**, Tizi-n’Test, Jebel Imdress (2000–2450 m), Goundafa; Mouna 1998; Evenhuis and Greathead 2015

﻿*Conophorus ﻿﻿﻿griseus* (Fabricius, 1787)

Mouna 1998; Zaitzev 2007; Evenhuis and Greathead 2015

﻿*Conophorus ﻿﻿﻿hamilkar* Paramonov, 1929

Timon-David 1951, **AP**, Mogador; Mouna 1998; Evenhuis and Greathead 2015; **AP** (Mogador) – MISR

﻿*Conophorus ﻿﻿﻿macroglossus* (Dufour, 1852)

Mouna 1998; Zaitzev 2007; Evenhuis and Greathead 201; **AP** (Mogador) – MISR

﻿*Conophorusmauritanicus* Bigot, 1892

= ﻿*Conophorus ﻿heteropilosus* Timon-David, in Timon-David 1951: 141; Mouna 1998: 84; Evenhuis and Greathead 2015: 192

Timon-David 1951, **MA**, Oulmès; Mouna 1998; Zaitzev 2007, **AP**, El Koudia (30 km SW from Rabat), Forêt Zaer (35 km SW from Rabat), N Tretten; Koçak and Kemal 2010; Dils 2013, **MA**, Mrirt; Evenhuis and Greathead 2015

﻿*Conophorus ﻿﻿﻿rossicus* Paramonow, 1929

Dils and Özbek 2006

﻿﻿***Dischistus* Loew, 1855**

﻿*Dischistus ﻿﻿﻿albatus* (Séguy, 1934)

= ﻿*Acanthogeron ﻿﻿﻿albatus* Séguy, 1934, in Séguy 1934d: 73; Zaitzev 2007: 162

Séguy 1934d; Zaitzev 2007, **SA**, 30 km S Tata

﻿*Dischistus ﻿﻿﻿auripilus* (Séguy, 1930)

= ﻿*Acanthogeron ﻿﻿﻿auripilus* Séguy, 1930, in Séguy 1930a: 104

Séguy 1930a, **AP**, Mogador; Séguy 1934b, **AP**, Zaers; Timon-David 1951, **AP**, Oued Korifla; Mouna 1998; Evenhuis and Greathead 2015

﻿*Dischistusmaroccanus* (Séguy, 1930)

= ﻿*Acanthogeronmaroccanus* Séguy, 1930, in Séguy 1930a: 106

Séguy 1930a, **AP**, Mogador; Mouna 1998; Zaitzev 2007, **HA**, Tazzarine; Evenhuis and Greathead 2015

﻿*Dischistus ﻿﻿﻿mittrei* (Séguy, 1930)

= ﻿*Acanthogeron ﻿﻿﻿mittrei* Séguy, in Séguy 1930a: 105

Séguy 1930a, **AP**, Mogador; Mouna 1998; Evenhuis and Greathead 2015

﻿*Dischistus ﻿﻿﻿perniveus* (Bezzi, 1925)

= ﻿*Acanthogeron ﻿﻿﻿perniveus* Bezzi, in Timon-David 1951: 143

Timon-David 1951, **AP**, Djamda de M’Tal; Mouna 1998; El Hawagry 2011; Evenhuis and Greathead 2015

﻿*Dischistus ﻿﻿﻿senex* (Wiedemann in Meigen, 1820)

= ﻿*Acanthogeron ﻿﻿﻿senex* Meigen, 1820, in Séguy 1953a: 83, Mouna 1998: 84, Zaitzev 2007: 162

Séguy 1930a, **HA**, Tafingoult (Goundafa, 1500–1600 m); Villeneuve 1933; Séguy 1953a, **HA**, Aït Ourir; Mouna 1998; Zaitzev 2003, **HA**, Taourirt; Zaitzev 2007, **HA**, Taourirt; El Hawagry 2011; Evenhuis and Greathead 2015; **AP** (Dradek), **MA** (Sefrou) – MISR

﻿*Dischistus ﻿﻿﻿separatus* (Becker, 1906)

= ﻿*Acanthogeron ﻿﻿﻿talboti* Séguy, 1930, in Séguy 1930a: 106

Evenhuis and Greathead 2015

﻿***Efflatounia* Bezzi, 1925**

﻿*Efflatounia ﻿﻿﻿berbera* Bowden, 1973

Ebejer et al. 2019, **AA**, Agadir – NMWC

﻿***Legnotomyia* Bezzi, 1902**

﻿*Legnotomyia ﻿﻿﻿fascipennis* Bezzi, 1925*

**SA**

***Merleus* Zaitzev, 2003**

﻿*Merleus ﻿﻿﻿punctipennis* Zaitzev, 2003

= ﻿*Merleus ﻿﻿﻿punctipennis*Zaitzev 2003: 599

Zaitzev 2003, **AP**, Skhirate; Zaitzev 2007, **AP**, Skhirate

﻿***Prorachthes* Loew, 1869**

﻿*Prorachthes ﻿﻿﻿crassipalpis* Villeneuve, 1930

Evenhuis and Greathead 2015

﻿***Systoechus* Loew, 1855**

﻿*Systoechus ﻿﻿﻿ctenopterus* (Mikan, 1796)

Timon-David 1951, **MA**, Ifrane; Mouna 1998; Dils and Özbek 2006; Zaitzev 2007; El Hawagry 2011; Evenhuis and Greathead 2015

﻿*Systoechus ﻿﻿﻿gomezmenori* Andréu Rubio, 1959

Carles-Tolrá 2002; Evenhuis and Greathead 2015

﻿*Systoechus ﻿﻿﻿gradatus* (Wiedemann in Meigen, 1820)

Timon-David 1951, **AP**, Mouldikht; Mouna 1998; Zaitzev 2007, **MA**, Taferiat, **HA**, Taourirt; El Hawagry 2011; Evenhuis and Greathead 2015

﻿*Systoechus ﻿﻿﻿mixtus* Wiedemann, 1821

= ﻿*Bombylius ﻿﻿﻿stylicornis* Macquart in Séguy 1941: 10

Séguy 1941d, **AA**, Agadir; Mouna 1998

﻿*Systoechus ﻿﻿﻿pumilio* Becker, 1915

Mouna 1998: 84

﻿***Triplasius* Loew, 1855**

﻿*Triplasius ﻿﻿﻿boghariensis* (Lucas, 1852)

Becker and Stein 1913, **Rif**, Tanger; Séguy 1930a, **EM**, Oujda; Mouna 1998; Pârvu and Zaharia 2007; Evenhuis and Greathead 2015

﻿*Triplasiusmaculipennis* (Macquart, 1846)

= ﻿Bombylius ﻿maculipennis var. melanopus Timon-David, in Timon-David 1951: 144

= ﻿*Bombylius (Triplasius) maculipennis* Macquart, 1849, in Zaitzev 2007: 166

Timon-David 1951, **MA**, Azrou; Zaitzev 2007, **MA**, route El Hachef, Criosement route Raubei Idris-Merhassine; Evenhuis and Greathead 2015

##### Ecliminae

﻿***Eclimus* Loew, 1844**

﻿*Eclimus ﻿﻿﻿gracilis* Loew, 1844

Séguy 1930a, **MA**, Ras el Ma; Timon-David 1951, **AP**, Oued Korifla; Mouna 1998; Bader and Arabyat 2004; Dils and Özbek 2006; Zaitzev 2007, **MA**, Maaziz; Evenhuis and Greathead 2015

﻿***Thevenetimyia* Bigot, 1875**

﻿*Thevenetimyia ﻿﻿﻿quedenfeldti* (Engel, 1885)*

**Rif**, **AP**, **MA**

##### Crocidiinae

﻿***Crocidium* Loew, 1860**

﻿*Crocidium ﻿﻿﻿aegyptiacum* Bezzi, 1925*

**SA**

﻿*Crocidium ﻿﻿﻿nudum* Efflatoun, 1945*

**EM**, **AA**

﻿***Semiramis* Becker in Becker and Stein 1914**

﻿*Semiramis ﻿﻿﻿punctipennis* Becker, 1914

Zaitzev 2007, **AA**, Aoulouz

##### Cythereinae

﻿***Amictus* Wiedemann, 1817**

﻿*Amictus ﻿﻿﻿castaneus* (Macquart, 1840)

Séguy 1930a, **AP**, Rabat, **HA**, Ank el Djemal; Mouna 1998; Evenhuis and Greathead 2015

﻿*Amictus ﻿﻿﻿compressus* (Fabricius, 1805)

Evenhuis and Greathead 2015

﻿*Amictus ﻿﻿﻿heteropterus* Macquart, 1838

Zaitzev 2007, **Rif**, Tanger, **AP**, Rabat, **HA**, S Tizi-n’Test, **AA**, Tizi-n’Taratine

﻿*Amictus ﻿﻿﻿oblongus* (Fabricius, 1805)

= ﻿*Bombylius ﻿﻿﻿oblongus* Fabricius, in Macquart 1834: 390

Macquart 1834

﻿*Amictus ﻿﻿﻿pulchellus* Macquart, 1846

Séguy 1930a, **AP**, Rabat, Maâmora; Mouna 1998; Zaitzev 2007, **HA**, Taourirt; El Hawagry 2011; Evenhuis and Greathead 2015; **AP** (Rabat) – MISR

﻿*Amictus ﻿﻿﻿setosus* Loew, 1869*

**AP**

﻿*Amictus ﻿﻿﻿tener* Becker, 1906

Zaitzev 2007, **AP**, Rabat

﻿*Amictus ﻿﻿﻿validus* Loew, 1869

Bader and Arabyat 2004; Dils and Özbek 2006; Karimpour 2012; Evenhuis and Greathead 2015

﻿*Amictus ﻿﻿﻿variegatus* Meigen in Waltl, 1835

Mouna 1998: 84

﻿***Chalcochiton* Loew, 1844**

﻿*Chalcochiton ﻿﻿﻿argentifrons* (Macquart in Lucas, 1849)

Séguy 1953a, **AP**, Cap Ghir, Salé, Sidi Battache, **MA**, Tizi-n’Bou Zabal (2300 m), **AA**, Aïn Chaïb (Souss); Evenhuis and Greathead 2015

﻿*Chalcochiton ﻿﻿﻿argyrocephalus* (Macquart, 1840)

= ﻿*Chalcochiton (Anthrax) ﻿argyrocephala* (Macquart), in Engel 1938: 328

Engel 1938; Séguy 1953a, **AA**, Agadir; El Hawagry 2011; Evenhuis and Greathead 2015

﻿*Chalcochiton ﻿﻿﻿atlantica* Dils, 2008

Dils 2008, **SA**, Guelmim

﻿*Chalcochiton ﻿﻿﻿holosericeus* (Fabricius, 1794)

= ﻿*Chalcochiton ﻿﻿﻿semiargentaea* Macquart, in Zaitsev 2007: 172

Séguy 1930a, **AP**, Maâmora, Sidi Bettache, **HA**, Tizi-n’Test, Jebal Imdress (2000–2450 m), Tafingoult (Goundafa, 1500–1600 m); Séguy 1941d; Mouna 1998; Zaitzev 2007, **Rif**, Tanger, **AP**, Skhirate, **EM**, Taourirt, **MA**, Taferiat, Meknès-Moulay Idriss, Merhassine, **AA**, Agadir, Ouarzazate; Evenhuis and Greathead 2015; **AP** (Salé, Forêt Temara, Oued Yquem, Meshra) – MISR

﻿*Chalcochiton ﻿﻿﻿maghrebi* Dils, 2017

Dils 2017, **Rif**, Souk El Kolla, Bab Taza, 10 km S of Mjara, **AP**, Sidi Bettache, Temsia, Imsouane, Mansouria, Rommani, Béni Slimane, Tioulit, **EM**, El Aioun, **MA**, Béni Mellal, el Ksiba, 10 km SE Bir Tamtam, Merchouch, Mrirt, Fès, **HA**, Azilal, Asni, Tizi-Mlil, **AA**, Taroudant, Tizi-n’Test, Tiznit, Agouim, Sidi Ifni, Mesti, Tafinegoult, Tizi-n’Tinififft, El Mrabtine, **SA**, Semara

﻿*Chalcochitonmaroccanus* Zaitzev, 2006

Séguy 1953a, **HA**, Tafingoult (Goundafa, 1500–1600 m); Zaitzev 2006, **AP** (south), Aït Melloul; Zaitzev 2007, **AP** (south), Aït Melloul

﻿*Chalcochiton ﻿﻿﻿merlei* Zaitzev, 2006

Zaitzev 2006, **AP**, Skhirate; Zaitzev 2007, **AP**, Skhirate

﻿*Chalcochiton ﻿﻿﻿pallasii* Loew, 1856

Bader and Arabyat 2004; Dils and Özbek 2006; Zaitzev 2007; Karimpour 2012; Evenhuis and Greathead 2015

﻿***Callostoma* Macquart, 1840**

﻿*Callostoma ﻿﻿﻿fascipenne* Macquart, 1840

Bader and Arabyat 2004

﻿***Cyllenia* Latreille, 1802**

﻿*Cylleniarustica* Rossi, 1790

Mouna 1998; Zaitzev 2007; **AP** (Mogador) – MISR

﻿﻿***Cytherea* Fabricius, 1794**

﻿﻿*Cytherea ﻿﻿﻿albolineata* Bezzi, 1925*

**SA**

﻿﻿*Cytherea ﻿﻿﻿alexandrina* Becker, 1902

Becker 1902: 30; Zaitzev 2007, **AA**, Jebel Tighermine (SE of Ouarzazate)

﻿﻿*Cytherea ﻿﻿﻿aurea* (Fabricius, 1794)

Séguy 1930a, **AP**, Rabat, **HA**; Mouna 1998; Bader and Arabyat 2004; Zaitzev 2007, **AA**, Tizi-n’Taratine; El Hawagry 2011; Evenhuis and Greathead 2015, **HA**, Tafingoult (Goundafa, 1500–1600 m); **AP** (Rabat, Oued Cherrat) – MISR

﻿﻿*Cytherea ﻿﻿﻿cinerea* Fabricius, 1805

= *Muliodelicatus* Becker, 1906

Becker 1906b: 153; Timon-David 1951, **AP**, Meshra; Mouna 1998; Bader and Arabyat 2004; El Hawagry 2011; Evenhuis and Greathead 2015

﻿﻿*Cytherea ﻿﻿﻿delicata* Becker, 1906

Zaitzev 2007, **HA**, S Tizi-n’Test, **AA**, Zagora, Taroudant, Tizi-n’Taratine

﻿﻿*Cythereadispar* (Loew, 1873)

Bader and Arabyat 2004; Dils and Özbek 2006; Evenhuis and Greathead 2015

﻿﻿*Cytherea ﻿﻿﻿fenestrata* (Loew, 1873)

Bader and Arabyat 2004; Evenhuis and Greathead 2015

﻿﻿*Cytherea ﻿﻿﻿infuscata* (Meigen, 1820)

Séguy 1930a, **EM**, Itzr (Haute Moulouya), **MA**, Forêt Timelilt (1900 m), **HA**, Aït el Hadj, Marrakech; Mouna 1998; Evenhuis and Greathead 2015; **AP** (Meskara) – MISR

﻿﻿*Cytherea ﻿maroccana* (Becker, 1903)

= *Muliomaroccanus* Becker, in Becker 1903: 89

Becker 1903, **Rif**, Tanger; Bezzi 1906: 249; Timon-David 1951, **AP**, Azemmour; Mouna 1998; El Hawagry 2011; Evenhuis and Greathead 2015

﻿﻿*Cytherea ﻿﻿﻿obscura* Fabricius, 1794

Séguy 1930a, **EM**, Haute Moulouya, **AP**, Sidi Bettache, **HA**, Ouaouenzert; Séguy 1941d; Mouna 1998; Dils and Özbek 2006; Zaitzev 2007, **MA**, Taferiat, **AA**, Agadir, Amredi, Jebel Tighermine (SE of Ouarzazate), Tizi-n’Tiniggigt, Tizi-n’Taratine, Tizi-n’Bachkoun; Karimpour 2012; Evenhuis and Greathead 2015

﻿﻿*Cytherea ﻿﻿﻿rungsi* Timon-David, 1951

Timon-David 1951, **EM**, Guenfouda; Mouna 1998; Evenhuis and Greathead 2015

﻿﻿*Cytherea ﻿﻿﻿thyridophora* (Bezzi, 1925)

Ebejer et al. 2019, **Rif**, Moulay Abdelsalam (Jebel Bouhachem, 965 m)

﻿﻿*Cytherea ﻿﻿﻿trifaria* (Becker, 1906)

Evenhuis and Greathead 2015

##### Lomatiinae

﻿***Lomatia* Meigen, 1820**

﻿*Lomatia ﻿﻿﻿abbreviata* Villeneuve, 1911

Séguy 1930a, **MA**, Forêt Zaers; Timon-David 1951, **EM**, Guercif; Mouna 1998; Bader and Arabyat 2004; Evenhuis and Greathead 2015; **AP** (Maâmora, Oued Cherrat, Dradek), **HA** – MISR

﻿*Lomatia ﻿﻿﻿belzebul paramonovi* Fabricius, 1794

Séguy 1930a, **AP**, Dar Salem, **MA**, Timhadit, Meknès, Aïn Leuh; Mouna 1998; Dils and Özbek 2006; Zaitzev 2008; Karimpour 2012; Evenhuis and Greathead 2015

﻿*Lomatia ﻿﻿﻿erynnis* (Loew, 1869)

Mouna 1998; Dils and Özbek 2006; Zaitzev 2008, **AP**, Rabat; Evenhuis and Greathead 2015

﻿*Lomatia ﻿﻿﻿hamifera* Becker, 1915

Mouna 1998: 84

﻿*Lomatia ﻿﻿﻿lachesis* Egger, 1859

Dils and Özbek 2006; Evenhuis and Greathead 2015

﻿*Lomatialateralis* (Meigen, 1820)

Séguy 1930a, **MA**, Ras el Ma, **HA**, Forêt Timelilt; Mouna 1998; Evenhuis and Greathead 2015; **AP** (Rabat), **MA** (Volubilis, Ras el Ma) – MISR

﻿*Lomatia ﻿﻿﻿obscuripennis* Loew, 1869

Zaitzev 2008, **AP**, Nkheila; Evenhuis and Greathead 2015

﻿*Lomatia ﻿﻿﻿sabaea* (Fabricius, 1781)

Mouna 1998: 84

﻿*Lomatia ﻿﻿﻿tysiphone* Loew, 1860

Zaitzev 2008, **MA**, Azrou, **AA**, Tizi-n’Taratine

##### Antoniinae

***Antonia* Loew, 1856**

*Antonia ﻿﻿﻿bouillonae* Séguy, 1932

Evenhuis and Greathead 2015

##### Anthracinae

Aphoebantini

﻿***Aphoebantus* Loew, 1872**

﻿*Aphoebantus ﻿﻿﻿wadensis* Becker, 1925*

**SA**

##### Anthracini

﻿***Anthrax* Scopoli, 1763**

﻿*Anthrax ﻿﻿﻿aethiops* (Fabricius, 1781)

Mouna 1998; Bader and Arabyat 2004; Dils and Özbek 2006; Evenhuis and Greathead 2015; **AP** (Forêt Maâmora) – MISR

﻿*Anthrax ﻿﻿﻿﻿anthrax* (Schrank, 1781)

= *Argyramoeba ﻿﻿﻿﻿anthrax* Schrank, in Séguy 1930a: 93

Séguy 1930a, **MA**, Aïn Leuh, Soufouloud (1900–2100 m), **HA**, Tizi-n’Test, Jebel Imdress (2000–2450 m), Goundafa; Timon-David 1951, **MA**, El Ksiba, Ifrane; Mouna 1998; Dils and Özbek 2006; Evenhuis and Greathead 2015

﻿*Anthrax ﻿﻿﻿binotatus* (Wiedemann in Meigen, 1820)

= *Argyramoeba ﻿﻿﻿binotata* Meigen, in Séguy 1926: 209, Séguy 1930a: 94

Séguy 1926; Séguy 1930a, **AP**, Rabat, **HA**, Tizi-n’Test, Jebel Imdress (2000–2450 m); Séguy 1949a, **HA**, Alnif; Mouna 1998; Dils and Özbek 2006; Koçak and Kemal 2010; El Hawagry 2011; Evenhuis and Greathead 2015; **AP** (Rabat) – MISR

﻿*Anthrax ﻿﻿﻿dentatus* (Becker, 1906)

Bader and Arabyat 2004; Zaitzev 2008, **AA**, Tizi-n’Tiniggigt; El Hawagry 2011; Evenhuis and Greathead 2015

﻿*Anthrax ﻿﻿﻿hemimelas* Speiser, 1910

Zaitzev 2008, **AA**, Ouarzazate, Jebel Tighermine (SE of Ouarzazate)

﻿*Anthrax ﻿﻿﻿kiritshenkoi* Paramonov, 1935

Evenhuis and Greathead 2015

﻿*Anthrax ﻿﻿﻿lucidus* (Becker, 1902)

Ebejer et al. 2019, **AA**, Ziz river (13 km N of Erfoud, 800 m)

﻿*Anthrax ﻿﻿﻿morio* Fabricius, 1775

Mouna 1998; **MA** (Ifrane, Azrou) – MISR

﻿*Anthrax ﻿﻿﻿trifasciatus* (Meigen, 1804)

= *Argyramoeba ﻿﻿﻿trifasciata* Meigen, in Timon-David 1951: 139

Séguy 1930a, **MA**, Meknès; Timon-David 1951, **AP**, south of Rabat; Mouna 1998; Dils and Özbek 2006; Koçak and Kemal 2010; El Hawagry 2011; Evenhuis and Greathead 2015

﻿*Anthrax ﻿﻿﻿varius* Fabricius, 1794

Séguy 1930a, **AP**, Rabat; Mouna 1998; Evenhuis and Greathead 2015 – MISR

﻿*Anthrax ﻿﻿﻿virgo* Egger, 1859

= *Argyramoeba ﻿﻿﻿virgo* Egger, in Séguy 1930a: 94

Séguy 1930a, **AP**, Rabat; Zaitzev 2008, **MA**, Taferiat, **AA**, Jebel Tighermine (SE of Ouarzazate)

﻿***Cononedys* Hermann, 1907**

﻿*Cononedys ﻿﻿﻿efflatouni* (Bezzi, 1925)*

**SA**

﻿*Cononedys ﻿﻿﻿escheri* Bezzi, 1908

Zaitzev 2008, **AP**, Skhirate, Rabat

﻿*Cononedys ﻿﻿﻿lyneborgi* (François, 1969)

Evenhuis and Greathead 2015

﻿*Cononedys ﻿﻿﻿scutellatus* Meigen, 1835

Zaitzev 2008, **Rif**, Jebala, Haouta el Kazdir, **AA**, Aouzlida near Aoulouz

﻿***Satyramoeba* Sack, 1909**

﻿*Satyramoeba ﻿﻿﻿hetrusca* (Fabricius, 1794)

Mouna 1998: 84

﻿***Spogostylum* Macquart, 1840**

﻿*Spogostylum ﻿﻿﻿isis* (Meigen, 1820)

Mouna 1998; Bader and Arabyat 2004; Dils and Özbek 2006; El Hawagry 2011; Karimpour 2012; Evenhuis and Greathead 2015

﻿*Spogostylum ﻿﻿﻿trinotatum* Dufour, 1852

Mouna 1998: 84

﻿*Spogostylum ﻿﻿﻿tripunctatum* (Pallas in Wiedemann, 1818)

Timon-David 1951, **HA**, Aït Mhamed Sgatt; Mouna 1998; Dils and Özbek 2006; Zaitzev 2008, **AA**, Jebel Tighermine (SE of Ouarzazate); El Hawagry 2011; Karimpour 2012; Evenhuis and Greathead 2015; **HA** (Aïn Mhamed Sgatt) – MISR

﻿***Turkmeniella* Paramonov, 1940**

﻿*Turkmeniella ﻿﻿﻿crosi* (Villeneuve, 1910)

Evenhuis and Greathead 2015

﻿***Exoprosopa* Macquart, 1840**

﻿*Exoprosopa ﻿﻿﻿aeacus* Meigen, 1804

Mouna 1998: 84

﻿*Exoprosopa ﻿﻿﻿baccha* Loew, 1869

Mouna 1998; Zaitzev 1999; Dils and Özbek 2006; Zaitzev 2008; Evenhuis and Greathead 2015

﻿*Exoprosopa ﻿﻿﻿capucina* (Fabricius, 1871)

Mouna 1998: 84

﻿*Exoprosopa ﻿﻿﻿circeoides* Paramonov, 1928

Zaitzev 2008, **AA**, Jebel Tighermine (SE of Ouarzazate)

﻿*Exoprosopa ﻿﻿﻿cleomene* Egger, 1859

Mouna 1998: 84

﻿*Exoprosopa ﻿﻿﻿decrepita* (Wiedemann, 1828)

Zaitzev 2008, **AA**, Zagora

﻿*Exoprosopa ﻿﻿﻿efflatouni* Bezzi, 1925

Zaitzev 2008, **AA**, Jebel Tighermine (SE of Ouarzazate), Ouarzazate, **SA**, Taganint (south of Bou-Izakarn)

﻿*Exoprosopa ﻿﻿﻿grandis* Wiedemann in Meigen, 1820

Mouna 1998; Zaitzev 2008, **HA**, Tishka (2200 m)

﻿*Exoprosopa ﻿﻿﻿italica* (Rossi, 1794)

Zaitzev 2008, **HA**, Taourirt, **AA**, Tizi-n’Taratine, Jebel Tighermine (SE of Ouarzazate), **SA**, Taganint (south of Bou-Izakarn)

﻿*Exoprosopa ﻿﻿﻿jacchus* (Fabricius, 1805)

Séguy 1930a, **AP**, Mogador, Sidi Taibi, **MA**, Tizi-s’Tkrine (1700 m), Dar Salem, Aïn Leuh, **HA**, Bou Tazzert; Mouna 1998; Mirceni and Pârvu 2009; Evenhuis and Greathead 2015; **Rif** (Talassemtane, Forêt Izarine, road of Jebha, Zoumi) – MISR

﻿*Exoprosopa ﻿﻿﻿minos* (Meigen, 1804)

Séguy 1949a, **AA**, Tata; Mouna 1998; Bader and Arabyat 2004; Dils and Özbek 2006; Zaitzev 2008, **MA**, Taferiat; El Hawagry 2011; El Hawagry and Dhafer 2015; Evenhuis and Greathead 2015; **MA** (Jebel Lachhab) – MISR

﻿*Exoprosopa ﻿pandora* (Fabricius, 1805)

Greathead 2001; Bader and Arabyat 2004; Evenhuis and Greathead 2015

﻿*Exoprosopa ﻿﻿﻿rutila* (Pallas & Wiedemann, 1818)

Evenhuis and Greathead 2015

﻿***Micomitra* Bowden, 1964**

﻿*Micomitra ﻿﻿﻿stupida* Rossi, 1790

= ﻿*Exoprosopa ﻿﻿﻿stupida* Rossi, in Mouna 1998: 84

Mouna 1998

﻿***Plesiocera* Macquart, 1840**

﻿*Plesiocera ﻿﻿﻿﻿﻿﻿﻿﻿﻿algira* (Macquart, 1840)

Zaitzev 2008, **MA**, Taferiat; Evenhuis and Greathead 2015

﻿***Heteralonia* Rondani, 1863**

﻿*Heteralonia (Homolonia) ﻿megerlei* (Hoffmansegg in Wiedemann, 1818)

Zaitzev 2008, **SA**, Goulimine

﻿*Heteralonia (Mesoclis) ﻿pygmalion* (Fabricius, 1805)

= ﻿*Exoprosopa ﻿﻿﻿pygmalion* Fabricius, in Timon-David 1951: 139

= ﻿*Mesoclis ﻿﻿﻿pygmalion* Fabricius, 1805, in Zaitzev 2008: 191

Séguy 1930a, **Rif**, Tanger, **AP**, Maâmora, Rabat, **MA**, Aïn Leuh; Timon-David 1951, **AP**, Temara; Mouna 1998; Zaitzev 2008, **AP**, Cherrat El Hawagry 2011; Evenhuis and Greathead 2015

﻿*Heteralonia (Zygodipla) ﻿﻿﻿﻿﻿﻿﻿﻿﻿algira* (Fabricius, 1794)

Séguy 1930a, **Rif**, Tanger, **AP**, Mogador, **HA**, Bou Tazzert; Mouna 1998; Zaitzev 2008, **HA**, Tifnite (south of Aït Melloul); El Hawagry 2011; Evenhuis and Greathead 2015

﻿*Heteralonia (Zygodipla) ﻿bagdadensi*s (Macquart, 1840)

Zaitzev 2008, **AA**, Zagora

﻿*Heteralonia (Zygodipla) singularis* (Macquart, 1840)

Bader and Arabyat 2004; Evenhuis and Greathead 2015

﻿*Heteraloniaarenacea* Becker, 1906

Evenhuis and Greathead 2015

﻿*Heteraloniadispar* (Loew, 1869)

= ﻿*Exoprosopadispar* Loew, in Timon-David 1951: 139

Timon-David 1951, **HA**, Marrakech; Mouna 1998; Dils and Özbek 2006; Evenhuis and Greathead 2015

﻿*Heteraloniarivularis* (Meigen, 1820)

= ﻿*Exoprosopa ﻿﻿﻿rivularis* Meigen, in Timon-David 1951: 139

Séguy 1930a, **AP**, Rabat, Maâmora; Timon-David 1951, **AP**, Oued Akreuch; Mouna 1998; Zaitzev 1999; Bader and Arabyat 2004; Zaitzev 2008, **AP**, Rabat

﻿***Oestranthrax* Bezzi, 1921**

﻿*Oestranthrax ﻿﻿﻿brunnescens* (Loew, 1857)

Bader and Arabyat 2004

﻿*Oestranthrax ﻿﻿﻿pallifrons* Bezzi, 1926

Evenhuis and Greathead 2015

﻿***Pachyanthrax* François, 1964**

﻿*Pachyanthrax ﻿﻿﻿albosegmentatus* (Engel, 1936)

Zaitzev 2008, **AA**, Jebel Tighermine (south of Ouarzazate)

﻿*Pachyanthrax ﻿﻿﻿nomadorum* (Greathead, 1970)

Koçak and Kemal 2010; Evenhuis and Greathead 2015

﻿***Exhyalanthrax* Becker, 1916**

﻿*Exhyalanthrax ﻿﻿﻿afer* (Fabricius, 1794)

= ﻿*Anthrax ﻿﻿﻿tangerinus* Bigot, 1892

Bezzi 1906; Mouna 1998; Bader and Arabyat 2004; Dils and Özbek 2006; Zaitzev 2008; El Hawagry 2011; Evenhuis and Greathead 2015, **Rif**, Tanger; **MA** (Ifrane) – MISR

﻿***Hemipenthes* Loew, 1869**

﻿*Hemipenthes ﻿﻿﻿morio* (Linnaeus, 1758)

Séguy 1930a, **MA**, Azrou, **HA**, Arround (Skoutana, 2000–2400 m); Dils and Özbek 2006; Koçak and Kemal 2010; Karimpour 2012; Evenhuis and Greathead 2015; **MA** (Azrou, Ifrane) – MISR

﻿*Hemipenthes ﻿﻿﻿velutinus* (Meigen, 1820)

Séguy 1930a, **MA**, Azrou; Mouna 1998; Bader and Arabyat 2004; Dils and Özbek 2006; Koçak and Kemal 2010; Evenhuis and Greathead 2015

﻿***Thyridanthrax* Osten-Sacken, 1886**

﻿*Thyridanthrax ﻿﻿﻿alphonsi* Sánchez Terrón and Roldan Bravo, 2000

Sánchez Terrón and Roldan Bravo 2000

﻿*Thyridanthrax ﻿﻿﻿elegans ssp. ﻿elegans* (Wiedemann in Meigen, 1820)

Séguy 1930a, **AP**, Rabat; Mouna 1998; Dils and Özbek 2006; El Hawagry 2011; Evenhuis and Greathead 2015; **AP** (Oued Cherrat, Rabat), **MA** (Volubilis) – MISR

﻿*Thyridanthrax ﻿﻿﻿fenestratus* (Fallén, 1814)

Séguy 1926; Séguy 1930a, **EM**, Berkane (1350–1400 m); Mouna 1998; El Hawagry 2011; Evenhuis and Greathead 2015; **Rif** (Tomorot) – MISR

﻿*Thyridanthrax ﻿﻿﻿griseolus* Klug, 1832

Zaitzev 2008, **SA**, Taganint (south of Bou-Izakarn)

﻿*Thyridanthrax ﻿﻿﻿hispanus* (Loew, 1869)

Becker and Stein 1913, **Rif**, Tanger; Sánchez Terrón and Roldan Bravo 2000

﻿*Thyridanthrax ﻿﻿﻿incanus* (Klug, 1832)

= ﻿*Anthrax ﻿﻿﻿incana* Klug, 1832, in Séguy 1953a: 83

Séguy 1930a, **AP**, Oued Korifla (Zaers); Timon-David 1951, **AP**, Zaer; Séguy 1953a, **MA**, Tarda; Mouna 1998; Bader and Arabyat 2004; Dils and Özbek 2006; El Hawagry 2011; Karimpour 2012; Evenhuis and Greathead 2015

﻿*Thyridanthrax ﻿﻿﻿loustaui* Andréu Rubio, 1961

Sánchez Terrón and Roldan Bravo 2000

﻿*Thyridanthraxmaroccanus* Dils, 2012

Dils 2012, **AA**, Ouarzazate, Skoura (1250 m), Amerzgane (1350 m)

﻿*Thyridanthrax ﻿﻿﻿mutilus* (Loew, 1869)*

**AA**

﻿*Thyridanthrax ﻿﻿﻿nebulosus* (Dufour, 1852)

Becker and Stein 1913, **Rif**, Tanger; Andréu Rubio 1959; Mouna 1998; Sánchez Terrón and Roldan Bravo 2000, **Rif**, Benibuifrur, Melilla, Restinga; Evenhuis and Greathead 2015

﻿*Thyridanthrax ﻿﻿﻿﻿perspicillaris ssp. ﻿perspicillaris* (Loew, 1869)

Séguy 1930a, **MA**, Aïn Leuh, Forêt Azrou, **HA**, Tizi-n’Test, Jebel Imdress (2000–2450 m), Goundafa; Mouna 1998; Bader and Arabyat 2004; Dils and Özbek 2006; El Hawagry 2011; Evenhuis and Greathead 2015

﻿*Thyridanthrax ﻿﻿﻿polyphemus* (Hoffmansegg, 1819)

Séguy 1930a, **MA**, Volubilis (400 m); Mouna 1998; Bader and Arabyat 2004; Dils and Özbek 2006; Karimpour 2012; Evenhuis and Greathead 2015

﻿***Veribubo* Evenhuis, 1978**

﻿*Veribubo ﻿﻿﻿angusteoculatus* (Becker, 1902)

Zaitzev 2008, **AA**, Zagora

﻿*Veribubo ﻿﻿﻿saudensis* (François, 1970)*

**AA**

﻿*Veribubo ﻿﻿﻿tabaninus* (François, 1970)*

**AA**, **SA**

﻿***Villa* Lioy, 1864**

﻿*Villa ﻿﻿﻿brunnea* Becker, 1916

Mouna 1998: 84

﻿*Villa ﻿﻿﻿ceballosi* Andréu Rubio, 1959

Mouna 1998; Koçak and Kemal 2010; Evenhuis and Greathead 2015

﻿*Villa ﻿﻿﻿cingulata* Meigen, 1804

Mouna 1998; **AP** (Rabat, Casablanca), **MA** (Volubilis, Fès) – MISR

﻿*Villa ﻿﻿﻿distincta* (Meigen in Waltl, 1835)

Dils and Özbek 2006; Koçak and Kemal 2010; Evenhuis and Greathead 2015

﻿*Villafasciata* (Meigen, 1804)

= ﻿*Villa ﻿﻿﻿circumdata* (Meigen), in Séguy 1941a: 29

Séguy 1930a, **AP**, Rabat; Séguy 1941a, **AP**, Rabat, **HA**, Tachdirt (Toubkal, 2500 m); Mouna 1998; Koçak and Kemal 2010; Evenhuis and Greathead 2015

﻿*Villa ﻿﻿﻿hottentotta* (Linnaeus, 1758)

= ﻿*Anthrax ﻿﻿﻿hottentotus* Linnaeus, in Séguy 1926: 198, Séguy 1930a: 92, Bléton and Fleuzet 1939: 64

Séguy 1930a, **AP**, Rabat, **MA**, Aïn Leuh; Bléton and Fleuzet 1939, **MA**, Fès; Séguy 1941d, **HA**, Tizi-n’Test; Mouna 1998; Dils and Özbek 2006; Evenhuis and Greathead 2015 – MISR

﻿*Villa ﻿﻿﻿ixion* (Fabricius, 1794)

Dils and Özbek 2006; Koçak and Kemal 2010; Karimpour 2012; Evenhuis and Greathead 2015

﻿*Villalaevis* Becker, 1915

Bader and Arabyat 2004; Dils and Özbek 2006; Koçak and Kemal 2010; Evenhuis and Greathead 2015

﻿*Villa﻿leucostoma* (Meigen, 1820)

Mouna 1998: 84; **AP** (Bou-Regreg) – MISR

﻿*Villaluculenta* Séguy, 1941

Séguy 1941d, **AA**, Taroudant; Mouna 1998; Koçak and Kemal 2010; Evenhuis and Greathead 2015

﻿*Villa﻿niphobleta* (Loew, 1869)

Bader and Arabyat 2004; Koçak and Kemal 2010; Karimpour 2012; Evenhuis and Greathead 2015

﻿*Villavenusta* (Meigen, 1820)

Mouna 1998: 84

﻿***Desmatoneura* Williston, 1895**

﻿*Desmatoneura﻿albifacies* (Macquart, 1840)

Ebejer et al. 2019, **AA**, Merzouga (714 m)

﻿*Desmatoneura﻿flavifrons* Becker, 1915

Zaitzev 2008, **AA**, Ouarzazate, Taroudant, Jebel Tighermine (SE of Ouarzazate)

﻿***Petrorossia* Bezzi, 1908**

﻿*Petrorossia ﻿albula* Zaitzev, 1962

Zaitzev 1999; Bader and Arabyat 2004; Zaitzev 2008, **AA**, Jebel Tighermine (SE of Ouarzazate); El Hawagry 2011; Evenhuis and Greathead 2015

﻿*Petrorossia ﻿﻿﻿freidbergi* Zaitzev, 1999

Zaitzev 2008, **AA**, Jebel Tighermine (SE of Ouarzazate)

﻿*Petrorossia ﻿﻿﻿hespera* (Rossi, 1790)

Séguy 1949a, **AA**, Tata; Mouna 1998; Zaitzev 1999; Dils and Özbek 2006; El Hawagry 2011; Evenhuis and Greathead 2015; **AP** (Bou-Regreg), **MA** (Timahdit) – MISR

﻿*Petrorossia ﻿﻿﻿margaritae* Zaitzev, 1999

Zaitzev 2008, **AA**, Ouarzazate, Jebel Tighermine (SE of Ouarzazate)

##### New records for Morocco

﻿*Amictus ﻿﻿﻿setosus* Loew, 1869

Atlantic Plain: Rommani, Marmouch, 33.568°N, 06.533°W, 400 m, 1♂1♀, Dils J.- Faes J., coll: PCJD.

﻿*Aphoebantus ﻿﻿﻿wadensis* Becker, 1925

Sahara: Tata, 9 km. W Tissint, 29.851°N, 07.265°W, 535 m, 1♂1♀, 03.iii.2007, Dils J.- Faes J., coll: PCJD.

*Bombylius* (Unplaced) ﻿*megacephalus* Portschinsky, 1887

Eastern Morocco: Figuig, Abbou Lakhal, 32.1587°N, 01.507°W, 1050 m, 1♀, 07.iii.2009, Dils J.- Faes J., coll: PCJD.

Anti Atlas: Tiznit, 84 km. SSE Guelmim, 28.631°N, 10.75522°W, 235 m, 1♂, 27.ii.2007, Dils J.- Faes J., coll: PCJD; Tiznit, Abaynou, 29.057°N, 10.026°W, 360 m, 1♀, 13.iii.2009, Dils J.- Faes J., coll: PCJD.

*Cononedys ﻿﻿﻿efflatouni* Bezzi, 1925

Sahara: Guelmim, Souk Tnine Nouaday, 29.166°N, 09.279°W, 680 m, 2♂3♀, 07.iv.2015, Dils J.- Faes J., coll: PCJD.

*Conophorus ﻿﻿﻿bellus* Becker, 1906

High Atlas: Marrakech, Oukaimeden, 31.233°N, 07.817°W, 2200 m, 3♂, 06.iv.2006, Dils J.- Faes J., coll: PCJD.

﻿*Crocidium ﻿﻿﻿aegyptiacum* Bezzi, 1925

Anti Atlas: Tiznit, Mesti, 29.274°N, 10.139°W, 280 m, 1♂, 23.iii.2006, Dils J.- Faes J., coll: PCJD.

Sahara: Tata, 28 km E of Tachjicht, 29.106°N, 09.149°W, 700 m, 1♀, 02.iii.2007, Dils J.- Faes J., coll: PCJD.

﻿*Crocidium ﻿﻿﻿nudum* Efflatoun, 1945

Eastern Morocco: Oujda, Plateau du Rekkam, 33.839°N, 02.55781°W, 1150 m, 1♀, 25.iv.2010, Dils J.- Faes J., coll: PCJD.

Anti Atlas: Agadir, Imsouane, 30.885°N, 09.780°W, 270 m, 3♂13♀, 09.iv.2009, Dils J.- Faes J., coll: PCJD; Ouarzazate, Amerzgane, 31.024°N, 07.223°W, 1370 m, 17♂12♀, 31.iii.2006, Dils J.- Faes J., coll: PCJD; Taliouine, 18 km. W of Taliouine, 30.6003°N, 08.082°W, 900 m, 2♀, 24.iii.2009, Dils J.- Faes J., coll: PCJD; Taroudant, Tafinegoult, 30.734°N, 08.430°W, 680 m, 3♀, 24.iii.2009, Dils J.- Faes J., coll: PCJD; Tiznit, Arbaa Sahel, 29.657°N, 09.869°W, 320 m, 11♂26♀, 21.iii.2006, Dils J.- Faes J., coll: PCJD.

﻿﻿*Cytherea ﻿﻿﻿albolineata* Bezzi, 1925

Sahara: Guelmim, Tainzirt, 29.121°N, 09.333°W, 670 m, 1♀, 31.iii.2010, Dils J.- Faes J., coll: PCJD.

*Geron ﻿﻿﻿intonsus* Bezzi, 1925

Middle Atlas: Khenifra, Boulôjoul, 32.873°N, 04.945°W, 1500 m, 7♂10♀, 26.iv.2008, Dils J.- Faes J., coll: PCJD.

High Atlas: Midelt, 32.680°N, 04.677°W, 1400 m, 2♂2♀, 20.iv.2015, Dils J.- Faes J., coll: PCJD; Midelt, Zeïda, 32.781°N, 04.964°W, 1500 m, 9♂11♀, 24.iv.2015, Dils J.- Faes J., coll: PCJD.

*Legnotomyia ﻿﻿﻿fascipennis* Bezzi, 1925

Anti Atlas: Zagora, Tazarinne, 30.798°N, 05.584°W, 900 m, 1♂, 07.iii.2007, Dils J.- Faes J., coll: PCJD.

Sahara: Tata, 9 km W of Tissint, 29.851°N, 07.265°W, 535 m, 2♂1♀, 03.iii.2007, Dils J.- Faes J., coll: PCJD.

﻿﻿﻿﻿*Thevenetimyia ﻿﻿﻿quedenfeldti* (Engel, 1885)

Rif: Tanger-Tétouan, Souk El Kolla (Quolla), 35.083°N, 05.538°W, 150 m, 5♂4♀, 30.iv.2017, Dils J.- Faes J., coll: PCJD.

Atlantic Plain: Rommani, Merchouch, 33.568°N, 06.753°W, 400 m, 5♂22♀, 04.v.2010, Dils J.- Faes J., coll: PCJD.

Middle Atlas: Tadla-Azilal, Afourer, 32.180°N, 06.520°W, 1150 m, 5♂10♀, 07.v.2008, Dils J.- Faes J., coll: PCJD; Béni Mellal, El Ksiba, 32.576°N, 06.050°W, 870 m, 7♂23♀, 23.iv.2008, Dils J.- Faes J., coll: PCJD.

﻿﻿﻿﻿*Thyridanthrax ﻿﻿﻿mutilus* Loew, 1869

Anti Atlas: Tiznit, Sidi Ifni, 29.384°N, 10.172°W, 0 m, 7♂1♀, 10.iv.2008, Dils J.- Faes J., coll: PCJD.

﻿*Veribubo ﻿﻿﻿saudensis* François, 1970

Anti Atlas: Erfoud, Tikkert-N-Ouchane, 31.223°N, 04.784°W, 830 m, 1♂3♀, 03.iv.2009, Dils J.- Faes J., coll: PCJD.

﻿*Veribubo ﻿﻿﻿tabaninus* François, 1970

Anti Atlas: Ouarzazate, Amerzgane, 31.024°N, 07.223°W, 1370 m, 2♂9♀, 31.iii.2006, Dils J.- Faes J., coll: PCJD; Erfoud, Tikkert-N-Ouchane, 31.250°N, 04.617°W, 860 m, 1♀, 07.iii.2007, Dils J.- Faes J., coll: PCJD; Ouarzazate, Amerzgane, 31.017°N, 07.229°W, 1350 m, 6♂27♀, 25.iii.2009, Dils J.- Faes J., coll: PCJD; Ouarzazate, Amerzgane, 31.017°N, 07.229°W, 1350 m, 12♂8♀, 25.iii.2009, Dils J.- Faes J., coll: PCJD; Ouarzazate, 30.847°N, 06.817°W, 1200 m, 1♀, 30.iii.2009, Dils J.- Faes J., coll: PCJD.

Sahara: Guelmim, Tainzirt, 29.121°N, 09.333°W, 670 m, 22♀, 31.iii.2010, Dils J.- Faes J., coll: PCJD.

#### ﻿﻿MYDIDAE

K. Kettani, T. Dikow

Number of species: **9**. Expected: 10

Faunistic knowledge of the family in Morocco: moderate

##### Leptomydinae

﻿***Leptomydas* Gerstaecker, 1868**

﻿*Leptomydas ﻿﻿﻿lusitanicus* (Wiedemann, 1820)

Mouna 1998

##### Rhopaliinae

﻿***Rhopalia* Macquart, 1838**

﻿*Rhopalia ﻿﻿﻿berlandi* Séguy, 1949a: 153

Séguy 1949a, **AA**, Tagounit, Asni; Mouna 1998; Dikow 2017

﻿***Perissocerus* Gerstaecker, 1868**

﻿*Perissocerus ﻿﻿﻿rungsi* Séguy, 1953

Séguy 1953a, **SA**

##### Syllegomydinae

Syllegomydini

﻿***Syllegomydas* Becker, 1906**

﻿*Syllegomydas ﻿algiricus* (Gerstaecker, 1868)

= ﻿*Rhopalia ﻿﻿﻿algirica* Gerstaecker, in Séguy 1928c: 149

Gerstaecker 1868, **AP**, Casablanca; Séguy 1928c, **AP**, Rabat; Séguy 1930a, **AP**, Rabat; Mouna 1998; El Hawagry 2011; Dikow 2017

﻿*Syllegomydas ﻿﻿﻿berlandi* (Séguy, 1941)

Séguy 1941, **AA**, Agadir; Dikow 2017

﻿*Syllegomydas ﻿bueni* Arias, 1914

Arias 1914, **AA**, Tafilalt; Séguy 1928c; Séguy 1930a; Carles-Tolrá 2015; Carles-Tolrá 2017, **EM**, Mariouri, Trifa; Dikow 2017

﻿*Syllegomydas ﻿cinctus* Macquart, 1835

Macquart 1835, **MA**, Immouzer road, **AA**, Agadir, Taroudant; Séguy 1930a; Mouna 1998; Carles-Tolrá 2017, **EM**, Quebdana, douar Shila, **AA**, Agadir coast; Dikow 2017

﻿*Syllegomydasmaroccanus* Séguy, 1928

Séguy 1928c, **AP**, Kénitra, Rabat, Oued Korifla, Forêt Zaers; Séguy 1930a, **AP**, Rabat, Temara, Oued Korifla, Forêt Zaers; Séguy 1932c; Mouna 1998; Carles-Tolrá 2017, **AP**, Larache, Ras Remel; Dikow 2017; **AP** (Kénitra) – MISR

﻿*Syllegomydas ﻿﻿﻿merceti* Arias, 1914

Arias 1914, **AP**, Mogador; Séguy 1930a; Mouna 1998; El Hawagry 2011, **AP**, Mogador; Dikow 2017

#### ﻿﻿MYTHICOMYIIDAE

K. Kettani, N. Evenhuis

Number of species: **8**. Expected: 15

Faunistic knowledge of the family in Morocco: poor

##### Empidideicinae

﻿***Empidideicus* Becker, 1907**

﻿*Empidideicus ﻿﻿﻿crocea* Séguy, 1949

= ﻿*Cyrtosia ﻿﻿﻿crocea* Séguy, in Séguy 1949a: 85

Séguy 1949a, **SA**, Guelmim; Séguy 1949c; Mouna 1998; Evenhuis 2002

##### Glabellulinae

﻿***Glabellula* Bezzi, 1902**

﻿*Glabellula ﻿maroccana* Evenhuis & Kettani, 2018

Evenhuis and Kettani 2018, **Rif**, Adrou (PNPB) – BPBM, MISR

﻿***Leylaiya* Efflatoun, 1945**

﻿*Leylaiya ﻿﻿﻿pellea* Evenhuis & Kettani, 2018

Evenhuis and Kettani 2018, **AA**, Tiznit – BPBM

##### Mythicomyiinae

﻿***Mythenteles* Hall & Evenhuis, 1991**

﻿*Mythenteles ﻿﻿﻿signifera* Evenhuis & Kettani, 2018

Evenhuis and Kettani 2018, **Rif**, Talassemtane (maison forestière, 1699 m) – BPBM, MISR

##### Platypyginae

﻿***Cyrtisiopsis* Séguy, 1930**

﻿*Cyrtisiopsis ﻿﻿﻿melleus* (Loew, 1856)

Evenhuis 2002; Zaitzav 2008, **AA**, Jebel Tighermine (SE Ouarzazate); Koçak and Kemal 2010; El Hawagry 2011

﻿*Cyrtisiopsissingularis* Séguy, 1930

Evenhuis 2002

﻿***Cyrtosia* Perris, 1839**

﻿*Cyrtosia ﻿﻿﻿aglota* Séguy, 1930

Evenhuis 2002

﻿*Cyrtosia ﻿marginata* Perris, 1839

Séguy 1930a, **HA**; Mouna 1998; Evenhuis 2002; Evenhuis and David 2004

#### ﻿﻿SCENOPINIDAE

K. Kettani, M. Carles-Tolrá

Number of species: **8** (+3 unidentified). Expected: 12

Faunistic knowledge of the family in Morocco: good

##### Scenopininae

﻿***Scenopinus* Latreille, 1802**

﻿*Scenopinus ﻿﻿﻿albicinctus* (Rossi, 1794)

= ﻿*Omphrale ﻿﻿﻿﻿albicincta* Rossi, in Séguy 1930a: 110

Séguy 1930a; Mouna 1998

﻿*Scenopinus ﻿﻿﻿fenestralis* (Linnaeus, 1758)

= ﻿*Omphrale ﻿﻿﻿fenestralis* Linnaeus, in Séguy 1930a: 110

Séguy 1930a; Mouna 1998

﻿*Scenopinus ﻿﻿﻿glabrifrons* Meigen, 1824

= ﻿*Omphrale ﻿﻿﻿glabrifrons* Meigen, in Séguy 1930a: 110

Séguy 1930a, **MA**, Aïn Leuh; Mouna 1998

﻿*Scenopinus ﻿﻿﻿niger* (De Geer, 1776)

Becker and Stein 1913, **Rif**, Tanger; Mouna 1998

﻿*Scenopinus ﻿﻿﻿parallelus* Kelsey, 1969

Kelsey 1969, **AP**, ﻿Villa Cisneros (Dakhla), **SA**, Río de Oro (Oued Eddahab)

﻿*Scenopinus ﻿﻿﻿physadius* (Séguy, 1930)

= ﻿*Omphrale ﻿﻿﻿physadia* Séguy, in Séguy 1930a: 111

Séguy, 1930, **EM**, Bou Denib; Kelsey 1969, **EM**, Bou Denib; Mouna 1998

﻿*Scenopinus ﻿﻿﻿pilosus* (Séguy, 1930)

= ﻿*Omphrale ﻿﻿﻿pilosa* Séguy, in Séguy 1930a: 111

Séguy 1930a, **AP**, Bou Knadel; Kelsey 1969, **AP**, Bou Knadel; Mouna 1998; Carles-Tolrá 2001

﻿*Scenopinus* undescribed ﻿﻿sp. 1

Ebejer et al. 2019, **Rif**, Martil (9 m)

﻿*Scenopinus* undescribed ﻿﻿sp. 2

Ebejer et al. 2019, **Rif**, Adrou (556 m)

﻿***Stenomphrale* Kröber, 1937**

﻿*Stenomphrale ﻿﻿﻿teutankhameni* (Kröber, 1923)

Ebejer et al. 2019, **AP**, forest of Maâmora (56 m)

﻿*Stenomphrale* ﻿sp.

**AP** (Essaouira (J.-P. Haenni leg.)) – MHNN Neuchâtel

#### ﻿﻿THEREVIDAE

K. Kettani, M. Hauser

Number of species: **27**.

Faunistic knowledge of the family in Morocco: moderate

##### Phycusinae

Phycusini

﻿***Actorthia* Kröber, 1912**

﻿*Actorthia ﻿﻿﻿micans* (Kröber, 1924)

Kröber 1924, **AA**, Errachidia (45 km S Erfoud), Merzouga

﻿***Phycus* Walker, 1850**

﻿*Phycuslacteipennis* Lyneborg, 2002

Lyneborg 2002, **AA**, 25 km S Goulmima (1000 m), **SA**, Mekn s-Tafilalet; Winterton et al. 2012; Badrawy and Mohammad 2013

﻿***Salentia* Costa, 1857**

﻿*Salentia ﻿﻿﻿anancitis* (Séguy, 1941)

= *Apioeicoceras ﻿﻿﻿anancitis* Séguy, in Séguy 1941d: 10

Séguy 1941d, **AA**, Agadir; Mouna 1998

﻿*Salentiacostalis* (Wiedemann, 1824)

= *Apioeicocerascostalis* Wiedemann, in Séguy 1930a: 108

Wiedemann 1824, **AP**, Mogador, **HA**, Marrakech-Tensift-Al Haouz; Séguy 1930a; Mouna 1998; Koçak and Kemal 2010

﻿*Salentia ﻿﻿﻿fuscipennis* Costa, 1857

Costa 1857, **Rif**, Tanger, **AA**, Tagadirt (Agadir); Becker and Stein 1913, **Rif**, Tanger; Séguy 1930a, **HA**, Bou Knadel, Tinmel; Mouna 1998

##### Therevinae

Therevini

﻿***Acanthothereva* Séguy, 1935**

﻿*Acanthothereva ﻿﻿﻿rungsi* Séguy, 1935

Séguy 1935b, **AP**, Mehdia (20 km S Rabat); Lyneborg 1968; Koçak and Kemal 2010; **Rif** (Cap Spartel) – MISR

﻿***Acrosathe* Irwin & Lyneborg, 1981**

﻿*Acrosathe ﻿﻿﻿annulata* (Fabricius, 1805)

Ebejer et al. 2019, **Rif**, Oued Kbir (Béni Ratene, 157 m)

﻿***Chrysanthemyia* Becker, 1912**

﻿*Chrysanthemyia ﻿﻿﻿chrysanthemi* (Fabricius, 1787)

Fabricius 1787, **EM**, Béni Snassen Mountains, Tafouralt (800 m); Séguy 1930a, **MA**, Meknès, Berrechid; Mouna 1998; **MA** (Oued Grou, Timahdit) – MISR

﻿*Chrysanthemyia ﻿﻿﻿velutinifrons* (Becker, 1912)

= ﻿*Chrysanthemyia ﻿﻿﻿lucidifrons*Becker 1912: 81

= *Oedicera ﻿﻿﻿velutinifrons* Becker, in Becker and Stein 1913: 82

Becker 1912, **Rif**, Region de Tanger-Tétouan, Cercle d’Ouezzane (300 m), **AP**, 3 km S Settat, **EM**, Figuig, **MA**, Fès-Boulmane, Sidi Harazem (223 m); Becker and Stein 1913, **Rif**, Tanger; Séguy 1930a, **MA**, Meknès; Mouna 1998; **MA** (Oued Grou) – MISR

﻿***Hoplosathe* Lyneborg & Zaitsev, 1980**

﻿*Hoplosathe ﻿﻿﻿distincta* Lyneborg & Zaitsev, 1980

Lyneborg and Zaitsev 1980, **HA**, Oued Tensift (Marrakech)

﻿***Neotherevella* Lyneborg, 1978**

﻿*Neotherevella ﻿﻿﻿macularis* (Wiedemann, 1828)

Hauser et al. 2017, **AA**, Tifnite (10 Km S Agadir), Merzouga (45 Km S Erfoud)

﻿***Thereva* Latreille, 1797**

﻿*Thereva ﻿﻿﻿atra* Kröber, 1913

El Hawagry 2011

*Thereva ﻿﻿﻿aureoscutellata* Kröber, 1914

Ebejer et al. 2019, **Rif**, Moulay Abdelsalam (965 m)

﻿﻿*Thereva ﻿﻿﻿binotata* Loew, 1847

Koçak and Kemal 2010

﻿*Thereva ﻿﻿﻿bipunctata* Meigen, 1820^16^

Ebejer et al. 2019, **MA**, Khénifra (17 km SW of Midelt, 1940 m; 17 km NW of Zaida, 1878 m; 28 km S of Timahdit, 2100 m), Lac Aguelmane Afennourir (30 km SW of Azrou, 2050 m)

﻿*Thereva ﻿﻿﻿brevicornis* Loew, 1847^17^

Pârvu et al. 2006, **AP**, Cap Sim; Popescu-Mirceni 2011

﻿*Therevachrysargyrea* Séguy, 1953

Séguy 1953a, **SA**, Amguilli Sguelma

﻿*Thereva ﻿﻿﻿cincta* Meigen, 1820

Ebejer et al. 2019, **Rif**, Oued Aliane (Ksar Sghir, 1 m)

﻿*Thereva ﻿﻿﻿funebris* Meigen, 1820^18^

= ﻿*Thereva ﻿﻿﻿lugubris* Meigen

Mouna 1998; **AP** (Rabat), **MA** (Ifrane) – MISR

﻿*Thereva ﻿﻿﻿graeca* Kröber, 1912**^3^**

Séguy 1930a, **Rif**, Tanger; Mouna 1998

﻿*Thereva ﻿﻿﻿plebeja* (Linnaeus, 1758)^3^

Séguy 1953a, **AA**, Tifnit (Souss); Mouna 1998; **MA** (Ras el Ma) – MISR

﻿*Thereva ﻿﻿﻿powelli* Séguy, 1930

Séguy 1930a, **MA**, Forêt Azrou; **MA** (Ras el Ma) – MISR

﻿*Thereva ﻿﻿﻿spiloptera* Wiedemann, 1824

Wiedemann 1824, **HA**, Ouirgane (Marrakech, 1000 m); Séguy 1930a, **Rif**, Tanger, **AP**, Mogador, **MA**, Meknès; Séguy 1953a, **AP**, Temara; Mouna 1998; El Hawagry 2011

﻿*Thereva ﻿﻿﻿spinulosa* Loew, 1847

Loew 1847, **AP**, Maâmora, **MA**, Khemisset

﻿*Thereva ﻿﻿﻿stigmatica* Kröber, 1912

EL-Hawagy 2011, **Rif**, Tanger

﻿*Thereva ﻿﻿﻿strigata* (Fabricius, 1794)^19^

Koçak and Kemal 2010

*Thereva﻿ ﻿tuberculata* Loew, 1847

= *Thereva﻿ ﻿algirica* Kröber, 1913, in Séguy 1953a: 83

Loew 1847, **AP**, Salé; Séguy 1930a, **MA**, Meknès; Séguy 1953a, **AP**, Salé; Mouna 1998; Koçak and Kemal 2010

##### Acknowledgments

We gratefully acknowledge Martin Ebejer (UK) for material, comments and cooperation, as well as Gail Kampmeier (USA) for sharing data of Moroccan Therevidae out of the mandala database (http://wwx.inhs.illinois.edu/research/mandala/about/).

### ﻿Empidoidea

#### ﻿﻿ATELESTIDAE

K. Kettani, P. Gatt

Number of species: **1**. Expected: 1

Faunistic knowledge of the family in Morocco: poor

﻿***Atelestus* Walker, 1837**

﻿*Atelestus* ﻿﻿sp. nov.

Ebejer et al. 2019, **Rif**, Dardara (484–730 m), Aïn Tissemlal (Azilane, 1255 m), Douar El Hamma (338 m), Chrabkha pond (Al Manzla, 58 m)

#### ﻿﻿EMPIDIDAE

K. Kettani, C. Daugeron

Number of species: **40**. Expected: 100

Faunistic knowledge of the family in Morocco: poor

##### Clinocerinae

﻿***Clinocera* Meigen, 1803**

﻿*Clinocera ﻿maroccana* (Séguy, 1941): 29 (= ﻿*Hydrodromia*)

= *Atalanta (Hydrodromia) ﻿﻿﻿﻿﻿﻿﻿﻿﻿algira* (Vaillant, 1952): 65

Séguy 1941a, **HA**, Anrhemer (Toubkal, 2500 m); Vaillant 1956b, **HA**, Sidi Chamarouch; Vaillant 1964; Dakki 1997

﻿*Clinocera ﻿﻿﻿megalatlantica* (Vaillant, 1957): 65 (= Atalanta (Atalanta))

Vaillant 1956b, **HA**; Dakki 1997

﻿*Clinocera ﻿﻿﻿nigra* Meigen, 1804: 292

= *Heleodromia ﻿﻿﻿unicolor* (Curtis, 1834): plate 513, *Paramesiaroberti* (Macquart, 1835): 657

Vaillant 1956b, **HA**, Izourar, Sidi Chamarouch, Aguelmous; Dakki 1997

﻿***Dolichocephala* Meigen, 1803**

﻿*Dolichocephala ﻿﻿﻿ocellata* (Costa, 1858): 7 (= ﻿*Ardoptera*)

= ﻿*oculata* (Loew, 1858~7): 7 (= ﻿*Ardoptera*)

= ﻿*novemguttata* (Strobl, 1893): 98 (= ﻿*Ardoptera*)

= ﻿*albohalterata* (Strobl, 1898): 399 (= ﻿*Ardoptera*)

= ﻿*barbarica* Vaillant, 1952: 65

= ﻿﻿﻿﻿﻿﻿﻿﻿﻿*algira* Vaillant, 1957: 64

Vaillant 1956b, **HA**, Imi-N’Ifri; Dakki 1997

﻿*Dolichocephala ﻿﻿﻿pavonica* Vaillant & Gagneur, 1998: 380

Vaillant and Gagneur 1998, **HA**, Demnat

﻿***Kowarzia* Mik, 1881**

﻿*Kowarzia ﻿﻿﻿barbatula* (Mik, 1880): 347 (= ﻿*Clinocera*)

= ﻿*Clinoceradorieri* (Vaillant, 1968): 88

Vaillant 1956b, **HA**, Cascade Siroua, Lac Tamhda (Anremer), Aguelmous, Sidi Chamarouch, Izourar, Oukaimeden; Dakki 1997; Vaillant and Moubayed 1998

﻿*Kowarzia ﻿﻿﻿bipunctata* (Haliday, 1833) (= *Heleodromia*)

Vaillant 1956b, **HA**, Oukaimeden; Vaillant 1964; Dakki 1997

﻿*Kowarzia ﻿﻿﻿dieuzedei* Vaillant, 1953: 60

Vaillant 1956b, **HA**, Lac Tamhda (Anremer); Vaillant 1964, **HA**; Dakki 1997

﻿*Kowarziamadicola* (Vaillant, 1965): 152 (= *Atalanta*)

Vaillant 1956b, **HA**, Tahanaout

﻿*Kowarziatenella* (Wahlberg, 1844): 107 (= *Parmesia*)

= *Heleodromiazetterstedti* (Walker, 1851): 105

= ﻿*Wiedemannia ﻿﻿﻿securigera* (Engel, 1918): 70

Vaillant 1956b, **HA**, Cascade Siroua, Lac Tamhda (Anremer), Aguelmous, Sidi Chamarouch, Izourar, Oukaimeden; Dakki 1997; Vaillant and Moubayed 1998

﻿***Wiedemannia* Zetterstedt, 1838**

﻿*Wiedemannia (Chamaedipsia) ﻿beckeri* (Mik, 1889): 71 (= ﻿*Chamaedipsia*)

= ﻿*jugorum* (Strobl, 1893): 105 (= ﻿*Chamaedipsia*)

= ﻿*crinita* Engel, 1918: 217 (as var. of ﻿*W.beckeri*)

= ﻿﻿*alticola* Vaillant, 195l: 54

= *alpina* Vaillant, 1967: 274 (as ssp. of W.beckeri)

= *glaciola* Wagner, 1985: 86 (as ssp. of W.﻿beckeri; new name for *W.﻿beckeri alpina* Vaillant)

Dakki 1997

﻿*Wiedemannia* (﻿*Chamaedipsia) mgounica* Vaillant, 1957: 69

Vaillant 1956a, **HA**, M’Goum; Dakki 1997

﻿*Wiedemannia (Philolutra) ﻿azurea* (Vaillant, 1951): 3 (= ﻿*Philolutra*)

El Mezdi and Giudicelli 1985, **HA**, Khettaras Marrakech; Dakki 1997; Vaillant and Moubayed 1998

﻿*Wiedemannia (Philolutra) ﻿fallaciosa* Loew, 1873: 44

Vaillant 1964, **HA**; Dakki 1997

﻿Wiedemannia (Philolutra) ﻿﻿fallaciosa ssp. litardierei Vaillant, 1957: 67

Vaillant 1956a, **HA**; Dakki 1997

﻿*Wiedemannia (Roederella) ﻿ouedorum* Vaillant, 1952: 371

= ﻿*ovedorum* (error) Vaillant, 1978: 469

Vaillant 1964, **HA**; Dakki 1997

##### Hemerodromiinae

﻿***Hemerodromia* Meigen, 1822**

﻿*Hemerodromia ﻿﻿﻿bethiana* Vaillant & Gagneur, 1998

Vaillant and Gagneur 1998, **MA**, Tigrigra (Azrou)

﻿*Hemerodromia ﻿﻿﻿subapicalis* Yang, Zhang & Zhang, 2007

= ﻿*Hemerodromia ﻿﻿﻿﻿﻿﻿﻿﻿﻿﻿apicalis*Vaillant and Gagneur 1998: 372 (preoccupied by Smith, 1969)

Vaillant and Gagneur 1998, **HA**, Oum-er-Rbia; Yang et al. 2007

﻿*Hemerodromia ﻿﻿﻿tigrigrana* Vaillant & Gagneur, 1998

Vaillant and Gagneur 1998, **MA**, Tigrigra (Azrou)

﻿*Hemerodromia ﻿﻿﻿todrhana* (Vaillant, 1956)

Vaillant 1956, **HA**, Todrha; El Mezdi and Guidicelli 1985; Dakki 1997; Vaillant and Gagneur 1998

﻿*Hemerodromia ﻿﻿﻿zarcana* Vaillant & Moubayed, 1998

Vaillant and Gagneur 1998, **MA**, Tigrigra (Azrou)

##### Empidinae

Empidini

﻿***Empis* Linnaeus, 1758**

﻿*Empis (Coptophlebia) ﻿confluens* Becker, 1907

**MA** (Meknès), 19.v.1997, K. Deneš leg. – OUMNH

﻿*Empis (Empis) ﻿decora* Meigen, 1822

Bahid 2018, **Rif**, Oued Tkarae (PNPB); Ebejer et al. 2019, **Rif**, Oued Nakhla, Moulay Abdelsalam

﻿*Empis (Empis) ﻿nikita* Shamshev, 2018

Shamshev 2018, **AP**, Essaouira

﻿*Empis (Kritempis) ﻿taffertensis* Daugeron, 2009

Daugeron 2009, **MA**, forest of Taffert; Bahid 2018

﻿*Empis (Leptempis) ﻿tenuis* Bahid & Daugeron, 2017

Bahid et al. 2017, **MA**, Tizi-s’Tkrine (Jebel Amar, 1760 m); Bahid 2018

﻿*Empis (Pachymeria) ﻿suberis* Becker, 1907

Ebejer et al. 2019, **Rif**, Moulay Abdelsalam, Issaguen, Bab Berred, Jebel Lakraâ

﻿*Empis (Polyblepharis) ﻿eumera* Loew, 1866

Ebejer et al. 2019, **MA**, Ifrane

﻿*Empis (Xanthempis) ﻿chopardi* Daugeron, 1997

Daugeron 1997, **MA**, Ifrane; Daugeron 2000; Bahid 2018

﻿*Empis (Xanthempis) ﻿edithae* Daugeron, 1997

Daugeron 1997, **HA**, High Imminen, Tachdirt; Daugeron 2000; Bahid 2018

﻿*Empis (Xanthempis) ﻿ifranensis* Daugeron, 1997

Daugeron 1997, **MA**, Ifrane; Daugeron 2000; Bahid 2018

﻿*Empis (Xanthempis) ﻿styriaca* (Strobl, 1893)

Chvála and Wagner 1989, **HA** [doubtful record]; Bahid 2018

﻿*Empis (Xanthempis) ﻿widanensis* Bahid & Daugeron, 2018

Bahid et al. 2018, **Rif**, Dayat Bayan Widane, Aïn Sedraouia, Tazia, Anissar, Lalla Outka

﻿***Rhamphomyia* Meigen, 1822**

﻿*Rhamphomyia (Rhamphomyia) maroccana* Collin, 2009

Chvála and Wagner 1989, **MA**, Ifrane; Collin 2009, **MA**, Ifrane; Bahid 2018, **Rif**, Oued Akrir (Fifi)

﻿*Rhamphomyia (Holoclera) ﻿tenuipes* Becker, 1907*

**AP**, Haenni pers. comm.

##### Hilarini

﻿***Hilara* Meigen, 1822**

﻿*Hilara ﻿﻿﻿algecirasensis* Strobl, 1899

Ebejer et al. 2019, **MA**, Lac Aguelmane Afennourir, **HA**, Ziz river

﻿*Hilara ﻿﻿﻿almeriensis* Strobl, 1906

Chvála 2008, **AA**, Tifoultoute (1146 m)

﻿*Hilara ﻿﻿﻿fusitibia* Strobl, 1899

Chvála 2008, **MA**, Ifrane (Forêt de Cédres, 1500 m)

﻿*Hilara ﻿﻿﻿longeciliata* Strobl, 1906

Chvála 2008, **AP**, Rabat (near Oued Bou-Regreg, 0–10 m)

﻿*Hilara ﻿﻿﻿schachti* Chvála, 2008

Chvála 2008, **MA**, Ifrane (Ghabat al Behar, 1650–1700 m); Bahid 2018

##### New record for Morocco

﻿*Rhamphomyia (Holoclera) ﻿tenuipes* Becker, 1907

Atlantic Plain: Essaouira, 6 km W, 3.iv.2002, Forêt de genévriers pâturée, 1♂1♀, J.-P. Haenni leg., coll. MHNN.

##### Acknowledgements

We are very grateful to Bradley Sinclair (Canadian Food Inspection Agency, Canada) and Adrian Plant (Mahasarakham University, Thailand) for reviewing parts of this family.

#### ﻿﻿DOLICHOPODIDAE

K. Kettani, I.Ya. Grichanov, O.P. Negrobov

Number of species: **112**. Expected: 300

Faunistic knowledge of the family in Morocco: poor

##### Diaphorinae

﻿***Argyra* Macquart, 1834**

﻿*Argyra ﻿﻿﻿argentina* Meigen, 1824

Parent 1924, **Rif**, Cap Spartel, Tétouan; Parent 1927, **Rif**, Tétouan

﻿*Argyra ﻿﻿﻿argyria* (Meigen, 1824)

Parent 1924 (females only), **Rif**, Cap Spartel, Tétouan; Kechev and Ivanova 2015

﻿*Argyra ﻿﻿﻿biseta* Parent, 1929

Parent 1929a, **Rif**, Tanger; Vaillant 1955a

﻿*Argyra ﻿﻿﻿grata* Loew, 1857^^20^^

Negrobov 1991 (no material provided)

﻿***Asyndetus* Loew, 1869**

﻿*Asyndetus ﻿﻿﻿separatus* (Becker, 1902)

Ebejer et al. 2019, **AA**, 14 km E of Rich (Errachidia, 1278 m)

﻿***Chrysotus* Meigen, 1824**

﻿*Chrysotus ﻿﻿﻿albibarbus* Loew, 1857

Ebejer et al. 2019, **MA**, Lac Aguelmane Afennourir (30 km SW of Azrou, 1760 m); Grichanov 2019, **AA**, Aït Melloul

﻿*Chrysotus ﻿﻿﻿gramineus* (Fallén, 1823)

Parent 1924, **Rif**, Tanger; Parent 1927

﻿*Chrysotus ﻿﻿﻿larachensis* Grichanov, Nourti & Kettani, 2020

Grichanov et al. 2020b, **Rif**, El Hamma (338 m)

﻿*Chrysotus ﻿﻿﻿pennatus* Lichtwardt, 1902

Ebejer et al. 2019, **Rif**, Smir Barrage (145 m), **AA**, 1 km N of Tarda (Errachidia, 1023 m)

﻿*Chrysotus ﻿﻿﻿suavis* Loew, 1857

Grichanov 2009, **HA**, Asni area (1100–1400 m); Nourti et al. 2019a, **Rif**, Amsemlil env. (1067 m), **MA**, Dayat Ifrane (1607 m), **HA**, Tahanout (956 m); Dawah et al. 2020

﻿***Diaphorus* Meigen, 1824**

﻿*Diaphorus ﻿﻿﻿africus* Parent, 1924

Parent 1924, **Rif**, Tétouan, Tanger; Parent 1927, **Rif**, Tanger; Ebejer et al. 2019, **Rif**, Oued Siflaou (281 m); Grichanov 2019, **AA**, Ouarzazate (1100 m)

﻿*Diaphorusvitripennis* Loew, 1859

Grichanov 2019, **AA**, Aït Melloul

##### Dolichopodinae

﻿***Dolichopus* Latreille, 1796**

﻿*Dolichopus ﻿﻿﻿andalusiacus* (Strobl, 1899)

Ebejer et al. 2019, **AP**, Loukous marsh (2 m); Nourti et al. 2019a, **Rif**, plage Stihat (0 m)

﻿*Dolichopus ﻿﻿﻿griseipennis* Stannius, 1831

Parent 1924, **Rif**, Tanger; Parent 1927; Séguy 1930a, **Rif**, Tanger; Nourti et al. 2019a, **Rif**, Adrou (PNPB, 556 m)

﻿*Dolichopus ﻿﻿﻿sabinus* Haliday, 1838

Ebejer et al. 2019, **Rif**, Martil (9 m), Oued Laou (2 m); Nourti et al. 2019a, **Rif**, plage Stihat (4 m)

﻿*Dolichopus ﻿﻿﻿scutopilosus* Parent, 1933

Parent 1933, **HA**, Arround

﻿*Dolichopus ﻿﻿﻿signifer* Haliday, 1832

Parent 1929b, “Maroc”; Séguy 1930a, **MA**, Ras el Ma; Grichanov 2019, **HA**, Oukaimeden (2600 m)

﻿*Dolichopus ﻿﻿﻿strigipes* Verrall, 1875

Grichanov 2009, **AP**, 40 km S Larache (0–20 m)

﻿***Gymnopternus* Loew, 1857**

﻿*Gymnopternus ﻿﻿﻿assimilis* (Staeger, 1842)

Nourti et al. 2019a, **Rif**, Amsemlil (1067 m)

﻿***Hercostomus* Loew, 1857**

﻿*Hercostomus ﻿﻿﻿apollo* (Loew, 1869)

Nourti et al. 2019a, **Rif**, Talassemtane (1696 m), Adrou (PNPB, 556 m), Amsemlil (PNPB, 1067 m)

﻿*Hercostomus ﻿﻿﻿canariensis* Santos Abreu, 1929

Grichanov et al. 2020a, **Rif**, Pont de Dieu (Akchour, 536 m); Nourti et al. 2019a (as H.aff.exarticulatoides Stackelberg, 1949)

﻿*Hercostomus ﻿﻿﻿chetifer* (Haliday, 1849)

Ebejer et al. 2019, **Rif**, Sidi Yahia Aarab (377 m)

﻿*Hercostomus ﻿﻿﻿discriminatus* Parent, 1925

Parent 1925, **Rif**, “Favier, Environs de Tanger”; Parent 1927, **Rif**, Tanger; Vaillant 1950, **Rif**, Tanger

﻿*Hercostomus ﻿exarticulatus* (Loew, 1857)

Vaillant 1956b, **HA**, Lac Tamhda (Anremer), Aguelmous; Grichanov et al. 2020a

﻿*Hercostomus ﻿﻿﻿excipiens* Becker, 1907

Parent 1924, **Rif**, Tétouan; Parent 1927; Séguy 1930a, **Rif**, Oued Judios (Tanger); Nourti et al. 2019a, **Rif**, Talembote (440 m)

﻿*Hercostomus ﻿﻿﻿germanus* (Wiedemann, 1817)

Parent 1924, **Rif**, Cap Spartel; Parent 1927; Kettani and Negrobov 2016, **Rif**, Chefchaouen, Ketama – MISR (**Rif**, Ketama)

﻿*Hercostomus ﻿﻿﻿longiventris* (Loew, 1857)

Vaillant 1956b, **HA**, Lac Tamhda (Anremer), Izourar

﻿***Muscidideicus* Becker, 1917**

﻿*Muscidideicus ﻿﻿﻿praetextatus* (Haliday, 1855)

Grichanov 2019, **AP**, Oualidia lagune

﻿***Ortochile* Berthold, 1827**

﻿*Ortochile ﻿﻿﻿morenae* (Strobl, 1899)

= ﻿*Hercostomus ﻿﻿﻿morenae* (Strobl, 1899), in Becker 1917: 225; Nourti et al. 2019a: 124

Grichanov and Nourti 2021; Nourti et al. 2019a, **Rif**, Mnezla (74 m), Talassemtane (980 m), Oued Ametrasse (841 m), estuary Tahaddart (dune marshland, 0 m)

﻿*Ortochile ﻿﻿﻿nigrocaerulea* Latreille, 1779

Parent 1924, **Rif**, Tanger, Cap Spartel, Tétouan, Béni Hozmar; Parent 1927, **Rif**, Tanger; Séguy 1930a, **Rif**, Oued Judios (Tanger); Grichanov 2009, **Rif**, Ouezzane (300 m); Nourti et al. 2019a, **Rif**, Douar El Hamma (338 m), Triwa Bni Hassane (654 m), Taida (501 m)

﻿***Platyopsis* Parent, 1929**

﻿*Platyopsismaroccanus* (Parent, 1929)

Parent 1929b, **Rif**, Tanger; Vaillant 1950, **Rif**, Tanger

﻿***Poecilobothrus* Mik, 1878**

﻿*Poecilobothrus ﻿﻿﻿appendiculatus* (Loew, 1859)

= ﻿*Hercostomus ﻿﻿﻿appendiculatus* (Loew): Ebejer et al. 2019: 146

Parent 1924, **Rif**, Tanger, Cap Spartel; Ebejer et al. 2019, **Rif**, Oued Nakhla (200 m), Moulay Abdelsalam (965 m), Dardara (730 m), Cap Spartel (155 m); Nourti et al. 2019a, **Rif**, Perdicaris Park (223 m)

﻿*Poecilobothrus ﻿﻿﻿infuscatus* (Stannius, 1831)

Ebejer et al. 2019, **Rif**, Tahaddart (2 m); **Rif** (Tahaddart) – MISR

﻿***Sybistroma* Meigen, 1824**

﻿*Sybistroma ﻿﻿﻿dufouri* Macquart, 1838

= *Haltericerus ﻿﻿﻿spathulatus* Loew, in Becker and Stein 1913: 86

Becker and Stein 1913, **Rif**, Tanger

﻿*Sybistroma ﻿﻿﻿obscurellum* Fallén, 182320

= *Hypophyllus ﻿﻿﻿obscurellus* Fallén, in Dakki 1997: 61

Dakki 1997 (no material provided)

﻿*Sybistroma ﻿﻿﻿﻿quadrifilatum* (Strobl, 1899)

= ﻿*Sybistroma ﻿﻿﻿parvulum* (Parent, 1927), in Grichanov and Nourti 2021: 190

Grichanov and Nourti 2021, **Rif**, Fahs Anjra (372 m)

﻿*Sybistroma ﻿﻿﻿theodori* Grichanov & Nourti, 2021

Grichanov and Nourti 2021, **Rif**, Moulay Abdelsalam (649 m)

﻿***Tachytrechus* Haliday, 1851**

﻿*Tachytrechus ﻿﻿﻿consobrinus* (Haliday, 1851)20

Parent 1938 (no material provided)

﻿*Tachytrechus ﻿﻿﻿goudoti* (Macquart, 1842)

= ﻿*Dolichopus ﻿﻿﻿goudoti* Macquart, 1842

Macquart 1842, **Rif**, Tanger; Parent 1926 (redescription), 1927

﻿*Tachytrechus ﻿﻿﻿insignis* (Stannius, 1831)

Parent 1927, “Maroc”; Séguy 1930a, **Rif**, Tanger, **HA**, Aguerd el Had, Talekjount (1000–1100 m); Vaillant 1956b, **HA**, Lac Tamhda (Anremer); Popescu-Mirceni 2011, **AP**, Merja Zerga; Grichanov 2019, **AP**, Essaouira

﻿*Tachytrechus ﻿﻿﻿notatus* (Stannius, 1831)

Vaillant 1950 (no material provided), 1956b, **HA**, Lac Tamhda (Anremer); Grichanov 2009, **AA**, 15 km SW Tazenakcht; Dawah et al. 2020

﻿*Tachytrechus ﻿﻿﻿planitarsis* Becker, 1907

Vaillant 1950, **HA**, Touggourt; Grichanov 2009, **AA**, 15 km SW Tazenakcht; Grichanov 2019, **AA**, Ouarzazate (1100 m)

##### Hydrophorinae

﻿***Anahydrophorus* Becker, 1917**

﻿*Anahydrophorus ﻿﻿﻿cinereus* (Fabricius, 1805)

= *Scatophaga ﻿﻿﻿cinerea* Fabricius, 1805: 205

Fabricius 1805, **AP**, Mogador (Essaouira); Séguy 1930a, **Rif**, Tanger; Vaillant 1955a, **AP**, Temara, Port-Lyautey; Boumezzough and Vaillant 1986a, **AP**, beach of Rabat; Kettani and Negrobov 2016, **AP**, Skhirat; **AP** (Skhirat) – MISR

﻿***Aphrosylus* Haliday, 1851**

﻿*Aphrosylusmaroccanus* Vaillant, 1955

Vaillant 1955a, **AP**, Port Lyautey

﻿*Aphrosylus ﻿﻿﻿mitis* Verrall, 1912

Grichanov 2019, **AP**, Oualidia lagune

﻿*Aphrosylus ﻿raptorluteipes* Parent, 1929

Parent 1929b, **AP**, Mogador (as a variation of ﻿*Aphrosylusraptor* Haliday, 1851); Vaillant 1955a; Negrobov, 1979 (as a subspecies of ﻿*Aphrosylusraptor* Haliday, 1851); Kettani and Negrobov 2016 (as ﻿*Aphrosylusraptor* Haliday, 1851); Grichanov 2019, **AP**, Oualidia lagune

﻿*Aphrosylus ﻿﻿﻿temaranus* Vaillant, 1955

Vaillant 1955a, **AP**, Temara; Grichanov 2019, **AP**, Oualidia lagune

﻿*Aphrosylus ﻿﻿﻿venator* Loew, 1857

Parent 1927, **Rif**, Tanger; Séguy 1930a, **Rif**, Tanger

﻿***Epithalassius* Mik, 1891**

﻿*Epithalassius ﻿﻿﻿corsicanus* Becker, 1910

Pârvu 2008, **AP**, Merja Zergha, Cap Sim, Essaouira

***Hydrophoprus* Fallén, 1823**

﻿*Hydrophorus ﻿﻿﻿balticus* (Meigen, 1824)

Vaillant 1956b, **HA**, Jebel Toubkal, Lac Tamhda (Anremer), Oukaimeden, Izourar; Boumezzough and Vaillant 1986a, **HA**, Jebel Toubkal (3100 m); Grichanov 2019, **HA**, Aguelmouss (2050 m), Oukaimeden (2600 m); Nourti et al. 2019a, **MA**, Mont Habri (2071 m)

﻿*Hydrophorus ﻿﻿﻿nilicola* Parent, 1927

= ﻿*Hydrophorus ﻿﻿﻿viridis ﻿nilicola* Parent, in Boumezzough and Vaillant 1986a: 297

Boumezzough and Vaillant 1986a, **MA**, Tizi-n’Imdrhas (1800 m), **HA**, Oued N’fis (650 m), **AA**, near Agadir N’oussbai (400 m); Grichanov 2019, **AP**, Essaouira

﻿*Hydrophorus ﻿﻿﻿oceanus* (Macquart, 1838)

= ﻿*Hydrophorus ﻿﻿﻿bisetus* Loew, 1857, in Parent 1927, Séguy 1930a, Grichanov 2019

Parent 1927, **AP**, Rabat; Séguy 1930a, **Rif**, Tandja el Balia (Tanger) (﻿*Hydrophorus ﻿﻿﻿bisetus* Loew); Vaillant 1955a, **AP**, Port-Lyautey; Boumezzough and Vaillant 1986a, **AP**, beach of Rabat

﻿*Hydrophorus ﻿﻿﻿praecox* (Lehmann, 1822)

Parent 1924, **Rif**, Cap Spartel, de Tanger à Tétouan, Rincón de Medik, Dar Riffien (Ceuta); Parent 1927; Séguy 1941a, **HA**, Toubkal (2500 m); Boumezzough and Vaillant 1986a, **HA**, Lac Tamhda, Lac Tamdhanit (Massif Anremer, 2900 m), Lac Izourar (Massif Azourki, 2650 m)

﻿*Hydrophorus ﻿﻿﻿viridis* (Meigen, 1824)^21^

Parent 1927, **AP**, Rabat; Boumezzough and Vaillant 1986a: 297

﻿***Liancalus* Loew, 1857**

﻿*Liancalusvirens* (Scopoli, 1763)

Vaillant 1956b, **HA**, Toubkal, Assif Tassouat (M’Goum), Aguelmous, Sidi Chamarouch, Imi-N’Ifri; Boumezzough and Vaillant 1986a, **HA**, Tahanaout (750 m), Adrar Anremer (2900 m), **AA**, Jebel Siroua (3000 m); Kettani and Negrobov 2016, **AP**, S-Tifni; Nourti et al. 2019a, **Rif**, Amsemlil env. (1059 m) – MISR (**AP**, S Tifni)

﻿***Machaerium* Haliday, 1832**

﻿*Machaerium ﻿﻿﻿maritimae* Haliday, 1832

Parent 1927, **Rif**, Tanger; Séguy 1930a, **Rif**, Tanger; Kettani and Negrobov 2016, **AP**, Oued Bou-Regreg; Grichanov 2019, **AP**, Oualidia lagune; **AP** (Oued Bou-Regreg) – MISR

﻿***Orthoceratium* Schrank, 1803**

﻿*Orthoceratium ﻿﻿﻿sabulosum* (Becker, 1907)

= ﻿*Orthoceratium ﻿lacustre* (Scopoli, 1763)^22^, in Ebejer et al. 2019: 146

Ebejer et al. 2019, **Rif**, Jebel Lakraâ (Talassemtane, 1541 m)

﻿***Thinophilus* Wahlberg, 1844**

﻿*Thinophilus (Thinophilus) ﻿flavipalpis* (Zetterstedt, 1843)

Pârvu et al. 2006, **AA**, Lac Edehby, Ouarzazate; Grichanov 2009, **AP**, 40 km S Larache (0–20 m)

﻿*Thinophilus (Thinophilus) ﻿indigenus* Becker, 1902

Ebejer et al. 2019, **AA**, 14 km E of Rich (Errachidia, 1278 m), Oued Laou (2 m); Grichanov 2019, **AA**, Ouarzazate (572 m)

﻿*Thinophilus (Thinophilus) ﻿mirandus* Becker, 1907

Negrobov 1971, **Rif**, Tanger; Grichanov 2019, **AA**, Ouarzazate (572 m)

﻿*Thinophilus (Schoenophilus) ﻿versutus* Haliday, 1851

= ﻿*Schoenophilus ﻿﻿﻿versutus* (Haliday, 1851), in Parent 1924, 1927

Parent 1924, **Rif**, Cap Spartel, Tétouan; Parent 1927; Pârvu et al. 2006, **AP**, Merja Zerga; Nourti et al. 2019a, **Rif**, Oued Souk Lhad (613 m), Dayat Tazia (733 m)

##### Medeterinae

﻿***Medetera* Fisher, 1819**

﻿*Medetera ﻿﻿﻿diadema* (Linnaeus, 1767)

= *Medeterus* (cf. ﻿*diadema*), in Séguy 1953a: 84

Parent 1938 (no material provided); Séguy 1953a, **AP**, Rabat; Nourti et al. 2019a, **Rif**, Kitane (49 m), **Rif**, plage Stihat (beach)

﻿*Medetera ﻿﻿﻿media* Parent, 1925

Nourti et al. 2019a, **Rif**, Faculty of Sciences of Tétouan (garden: on trunk of olive tree, 14 m)

﻿*Medetera ﻿﻿﻿micacea* Loew, 1857

Ebejer et al. 2019, **Rif**, Dardara (730 m); Nourti et al. 2019a, **Rif**, Issaguen (1547 m),

**HA**, Lac Tislit (Imilchil, 2254 m)

﻿*Medetera ﻿﻿﻿pallipes* (Zetterstedt, 1843)^23^

Nourti et al. 2019a, **Rif**, Douar El Hamma (338 m)

﻿*Medetera ﻿﻿﻿petrophila* Kowarz, 187720

Parent 1938 (no material provided)

﻿*Medetera ﻿﻿﻿petrophiloides* Parent, 1925

Nourti et al. 2019a, **Rif**, Issaguen (1547 m)

﻿*Medetera* ﻿aff. ﻿*roghii* Rampini & Canzoneri, 1979

Nourti et al. 2019a, **Rif**, Douar El Hamma (338 m)

﻿*Medetera ﻿﻿﻿truncorum* Meigen, 1824^24^

Ebejer et al. 2019a, **Rif**, Dardara (484 m)

﻿*Medetera ﻿﻿﻿varvara* Grichanov & Vikhrev, 2009

Grichanov and Vikhrev 2009, **AP**, Essaouira

﻿***Thrypticus* Gerstäcker, 1864**

﻿*Thrypticus ﻿﻿﻿bellus* Loew, 1869

Séguy 1930a, **EM**, Dayat Sidi Kacem; Nourti et al. 2019a, **Rif**, Amsemlil env. (1067 m), estuary Oued Tahaddart (0 m), Barrage 9 Avril, plage Stihat (0 m)

##### Microphorinae

﻿***Schistostoma* Becker, 1902**

﻿*Schistostoma ﻿﻿﻿eremita* Becker, 1902

Ebejer et al. 2019, **AA**, Ziz river (12 km S of Rissani, 737 m), Lac Tiffert (4 km W of Merzouga, 702 m)

##### Neurigoninae

﻿***Neurigona* Rondani, 1856**

﻿*Neurigona ﻿﻿﻿solodovnikovi* Grichanov, 2010

= ﻿*Neurigona ﻿﻿﻿punctifera* Becker, 1907, in Kettani and Negrobov 2016 (misidentification)

Grichanov 2010, **AP**, 40 km S Larache; Kettani and Negrobov 2016, **Rif**, Aïn Tissemlal (Azilane, 1255 m); Nourti et al. 2019a, **Rif**, Perdicaris Park (223 m), Taria Bni Faghloum (894 m), Chrafate (832 m)

##### Parathalassiinae

﻿***Microphorella* Becker, 1909**

﻿*Microphorella ﻿﻿﻿ulrichi* Gatt, 2003

Gatt 2003, **Rif**, Tanger, Oued Armal (Ksar Sghir)

﻿***Parathalassius* Mik, 1891**

﻿*Parathalassius ﻿﻿﻿blasigii* Mik, 1891

Ebejer et al. 2019, **AP**, Larache (5 m)

##### Peloropeodinae

﻿***Chrysotimus* Loew, 1857**

﻿*Chrysotimus ﻿﻿﻿molliculoides* Parent, 1937

Parent 1937a, **MA**, Ifrane (1600 m); Parent 1937b; Nourti et al. 2019a, **Rif**, Dayat Tazia (733 m)

﻿***Micromorphus* Mik, 1878**

﻿*Micromorphus ﻿﻿﻿albipes* (Zetterstedt, 1843)

Parent 1924, **Rif**, Béni Hozmar (Tétouan); Parent 1927, **Rif**, Tétouan; Kechev and Ivanova 2015; Nourti et al. 2019a, **Rif**, Oued Souk Lhad (613 m), estuary Oued Tahaddart (0 m), Barrage 9 Avril, Kharouba, plage Stihat (0 m), Aïn Jdioui, **EM**, Bouanane (Figuig, 855 m), **AA**, Boudnib (951 m); Dawah et al. 2020

﻿*Micromorphus ﻿﻿﻿minusculus* Negrobov, 2000

Nourti et al. 2019a, **EM**, Bouanane (Figuig, 855 m), **AA**, Taliouine (Taroudant, 1014 m); Grichanov 2019, **AA**, Ouarzazate

##### Rhaphiinae

﻿***Rhaphium* Meigen, 1803**

﻿*Rhaphium ﻿﻿﻿appendiculatum* Zetterstedt, 184920

= ﻿*Rhaphium ﻿﻿﻿macrocerum* (Parent, 1925), in Parent 1938, Grichanov 2019

Parent 1938 (no material provided)

﻿*Rhaphium ﻿﻿﻿brevicorne* Curtis, 1835

= *Xiphandrium ﻿﻿﻿pectinatum* Becker, in Vaillant 1956b: 112, Grichanov 2019

Vaillant 1956b, **AP**, Rabat, **HA**, Oukaimeden; Kettani and Negrobov 2016, **AP**, Rabat; Grichanov 2019, **HA**, Oukaimeden (1000 m); Nourti et al. 2019a, **Rif**, Pont de Dieu (Akchour, 536 m), Dayat Tazia (733 m), Moulay Abdelsalam (1177 m); **AP** (Rabat) – MISR

﻿*Rhaphium ﻿﻿﻿caliginosum* Meigen, 1824

= *Raphium ﻿﻿﻿lanceolatum* Loew, 1850, in Kazerani et al. 2013

Parent 1924, **Rif**, Cap Spartel; Kazerani et al. 2013 (no material provided); Kechev 2017; Nourti et al. 2019a, **Rif**, Amsemlil env. (1067 m), Dbani (Bni Selmane, 1046 m)

﻿*Rhaphium ﻿﻿﻿fascipes* (Meigen, 1824)

= *Porphyrops ﻿﻿﻿fascipes* Meigen, 1824, in Parent 1927

Parent 1927, **Rif**, Tétouan

﻿*Rhaphium ﻿﻿﻿fissum* Loew, 1850

Grichanov 2009, **AA**, Tizi-n’Test pass (2100 m)

##### Sciapodinae

﻿***Sciapus* Zeller, 1842**

﻿*Sciapus ﻿﻿﻿adumbratus* Becker, 1902

Grichanov and Negrobov 2014, **AP**, near Essaouira, **AA**, Oued Souss, near Ouarzazate (1100 m)

﻿*Sciapus ﻿﻿﻿costae* (Mik, 1890)

Negrobov 1991 (no material provided); Ebejer et al. 2019, **Rif**, Oued Laou (30 m)

﻿*Sciapus ﻿﻿﻿euzonus* (Loew, 1859)

= *Psilopus ﻿﻿﻿euzonus* Loew, in Dakki 1997: 62

Parent 1927, **Rif**, El Mahadi; Séguy 1930a, **Rif**, Tanger; Séguy 1941d, **AA**, Taroudant

﻿*Sciapus ﻿﻿﻿glaucescens* (Loew, 1856)

Grichanov and Negrobov 2014, **AP**, Oualidia

﻿*Sciapus ﻿﻿﻿heteropygus* Parent, 1926

Nourti et al. 2019c, **Rif**, Talassemtane National Park (1696 m)

*Sciapus ﻿﻿﻿holoxanthos* Parent, 1926

Nourti et al. 2019c, **Rif**, Jbel Bouhachem, Adrou (556 m), Talassemtane National Park (1696 m)

*Sciapus ﻿﻿﻿laetus* (Meigen, 1838)

Grichanov 2009, **AA**, 40 km S Larache (0–20 m); Ebejer et al. 2019, **Rif**, Martil (9 m)

﻿*Sciapus ﻿﻿﻿longulus* (Fallén, 1823)^25^

Pârvu et al. 2006, **AP**, Merja Zerga

﻿*Sciapus* ﻿aff. ﻿*negrobovi* Naglis & Barták, 2015

Nourti et al. 2019a, **Rif**, plage Stihat (0 m), Kitane (49 m) (as Sciapusaff.negrobovi); Nourti et al. 2019c

##### Sympycninae

﻿***Campsicnemus* Haliday in Walker, 1851**

﻿*Campsicnemus ﻿﻿﻿crinitarsis* Strobl, 1906

Dakki 1997 (no material provided); Grichanov 2012, **AP**, Essaouira; Kettani and Negrobov 2016, **Rif**, Oued Amsa

﻿*Campsicnemus ﻿﻿﻿curvipes* (Fallén, 1823)^26^

Frey 1936 (no material provided)

﻿*Campsicnemus ﻿﻿﻿filipes* Loew, 1859

Grichanov 2009, **AA**, 40 km S Larache (0–20 m)

﻿*Campsicnemus ﻿﻿﻿loripes* (Haliday, 1832)

Ebejer et al. 2019, **Rif**, Moulay Abdelsalam (965 m)

﻿*Campsicnemus ﻿﻿﻿magius* (Loew, 1845)

Pârvu et al. 2006, **AA**, Lac Edehby, Ouarzazate; Grichanov 2019, **AP**, Essaouira

﻿*Campsicnemus ﻿﻿﻿simplicissimus* Strobl, 1906

Nourti et al. 2019a, **Rif**, plage Stihat (0 m)

﻿***Sympycnus* Loew, 1857**

﻿*Sympycnus ﻿﻿﻿pulicarius* (Fallén, 1823)

Nourti et al. 2019a, **HA**, Aïn Taferaout (Sidi Masali, 1237 m)

﻿***Syntormon* Loew, 1857**

﻿*Syntormon ﻿﻿﻿aulicus* Meigen, 1824

= *Eutarsus ﻿﻿﻿aulicus* Meigen, in Parent 1924, 1927; Séguy 1930a: 125

Parent 1927, **Rif**, Tanger; Séguy 1930a, **Rif**, Tanger; Vaillant 1952, 1956b, **HA**, Imi-N’Ifri

﻿*Syntormon ﻿﻿﻿codinai* Parent, 1924

Parent 1924, **Rif**, Cap Spartel, Tanger; Parent, 1927, **Rif**, Tanger

﻿*Syntormon ﻿﻿﻿denticulatus* (Zetterstedt, 1843)

= ﻿*Syntormon ﻿﻿﻿pumilus* Parent, 1925 (nec Meigen, 1824; misidentification), in Pârvu et al. 2006, Grichanov 2019

Parent 1924, **Rif**, Tétouan; Parent 1927, **Rif**, Tétouan; Séguy 1930a, **Rif**, Tanger; Pârvu et al. 2006, **AA**, Lac Edehby, Ouarzazate; Nourti et al. 2019a, **Rif**, plage Stihat (0 m), Amlay (294 m), Koudiat Taifour (100 m), Amsemlil env. (1067 m), Dayat Tazia (733 m), Oued Souk Lhad (613 m), **HA**, Aïn Taferaout (Sidi Masali, 1237 m), **AA**, Taliouine (1014 m)

﻿*Syntormon ﻿﻿﻿mikii* Strobl, 1899

Parent 1927, “Maroc”; Kechev and Ivanova 2015; Nourti et al. 2019a, **Rif**, Pont de Dieu (Akchour, 536 m), Moulay Abdelsalam (1177 m)

﻿*Syntormon ﻿﻿﻿monilis* (Haliday, 1851)

Parent 1924, **Rif**, Cap Spartel; Parent 1927, **Rif**, Cap Spartel

﻿*Syntormon ﻿﻿﻿pallipes* (Fabricius, 1794)

Parent 1924, **Rif**, Cap Spartel, Tétouan, Chefchaouen; Parent 1927, **Rif**, Cap Spartel; Nourti et al. 2019a, **Rif**, Oued Souk Lhad (613 m), Talassemtane (339 m), Perdicaris Park (223 m), Amsemlil env. (1067 m); Dawah et al. 2020

﻿*Syntormon ﻿﻿﻿pilitibia* Grichanov, 2013

Nourti et al. 2019a, **Rif**, Amsemlil (PNPB, 1067 m)

﻿*Syntormon ﻿﻿﻿pumilus* (Meigen, 1824)

= ﻿*Syntormon ﻿﻿﻿rufipes* auctt. (nec Meigen, 1824; misidentification), in Pârvu et al. 2006; Grichanov 2019

Pârvu et al. 2006, **AA**, Lac Edehby, Ouarzazate

﻿*Syntormon ﻿﻿﻿zelleri* (Loew, 1850)

Vaillant 1956b, **HA**, Oukaimeden, Izourar; Pârvu et al. 2006, **AP**, Merja Zerga; Nourti et al. 2019a, **Rif**, Pont de Dieu (Akchour, 536 m), Amsemlil env. (1059–1067 m)

﻿***Teuchophorus* Loew, 1857**

﻿*Teuchophorus ﻿﻿﻿cristulatus* Mueffels & Grootaert, 1990

Ebejer et al. 2019, **AA**, 14 km E of Rich (Errachidia, 1278 m)

﻿*Teuchophorusrifensis* Nourti, Grichanov & Kettani, 2019

Nourti et al. 2019b, **Rif**, Oued Souk Lhad (613 m)

﻿*Teuchophorusspinigerellus* (Zetterstedt, 1843)

Pârvu et al. 2006, **AA**, Lac Edehby, Ouarzazate

##### Xanthochlorinae

﻿***Xanthochlorus* Loew, 1857**

﻿*Xanthochlorus ﻿﻿﻿tenellus* (Wiedemann, 1817)

Grichanov 2009, **AA**, 40 km S Larache (0–20 m); Ebejer et al. 2019, **Rif**, Moulay Abdelsalam (1180 m)

#### ﻿﻿HYBOTIDAE

K. Kettani, P. Gatt

Number of species: **44**. Expected: 120

Faunistic knowledge of the family in Morocco: poor

##### Ocydromiinae

Bicellariini

﻿***Bicellaria* Macquart, 1823**

﻿*Bicellaria ﻿﻿﻿spuria* Fallén, 1816

Becker and Stein 1913, **Rif**, Tanger

##### Ocydromiini

﻿***Ocydromia* Meigen, 1820**

﻿*Ocydromia ﻿﻿﻿glabricula* (Fallén, 1816)

Becker and Stein 1913, **Rif**, Tanger

﻿***Oropezella* Collin, 1926**

﻿*Oropezella ﻿﻿﻿sphenoptera* (Loew, 1873)

Cassar et al. 2005, **Rif**, lagoon Smir

##### Tachydromiinae

Drapetini

﻿***Chersodromia* Haliday in Walker, 1851**

﻿*Chersodromia ﻿﻿﻿albopilosa* Chvála, 1970

Cassar et al. 2008, **Rif**, Basin Laou

﻿*Chersodromia ﻿﻿﻿nigrosetosa* Chvála, 1970

Chvála 1981 (Ceuta); Chvála and Kovalev 1989

﻿*Chersodromia ﻿﻿﻿pseudohirta* Chvála, 1970

Ebejer et al. 2019, **Rif**, Kabila beach

﻿***Crossopalpus* Bigot, 1857**

﻿*Crossopalpus ﻿﻿﻿﻿﻿aeneus* (Walker, 1871)^27^

Shamshev et al. 2005, **HA**, Marrakech, Ouirgane

﻿*Crossopalpus ﻿﻿﻿dilutipes* (Strobl, 1906)

Ebejer et al. 2019, **AP**, 9 km SE of Aïn Chouk (Lower Loukous marsh, 6 m)

﻿*Crossopalpus ﻿﻿﻿nigritellus* (Zetterstedt, 1842)

Ebejer et al. 2019, **Rif**, Talassemtane (maison forestière), Issaguen (1620 m)

﻿*Crossopalpus ﻿﻿﻿setiger* (Loew, 1859)

Ebejer et al. 2019**Rif**, Smir lagoon

﻿***Drapetis* Meigen, 1822**

﻿*Drapetis ﻿﻿﻿disparilis* Frey, 1936

Chvála 1981 (Ceuta); Chvála and Kovalev 1989

﻿*Drapetislaevis* Becker, 1914

Becker and Stein 1913, **Rif**, Tanger; Kovalev 1970; Chvála and Kovalev 1989

﻿***Elaphropeza* Macquart, 1827**

﻿*Elaphropeza ﻿﻿﻿boergei* Chvála, 1971

Ebejer et al. 2019, **Rif**, Smir lagoon, Oued Laou (saltmarsh)

﻿*Elaphropeza ﻿﻿﻿hutsoni* Smith, 1967

Ebejer et al. 2019, **Rif**, Jnane Niche (46 m)

﻿***Stilpon* Loew, 1859**

﻿*Stilpon ﻿﻿﻿demnatensis* Vaillant, 1956^28^

Vaillant 1956b: 244, **HA**, Imi-N’Ifri

﻿*Stilpon ﻿﻿﻿moroccensis* Grootaert & Zouhair, 2021

Grootaert et al. 2021, **Rif**, beach of Stehat, Bab Tariouant, Amsemlil, **EM**, Bouanane

﻿*Stilpon ﻿﻿﻿subnubilus* Chvála, 1988

Ebejer et al. 2019, **Rif**, Smir lagoon, M’Diq (Kabila beach and dunes), Martil (beach and dunes)

##### Tachydromiini

﻿***Platypalpus* Macquart, 1827**

﻿*Platypalpus* ﻿﻿﻿*alluaudi* Grootaert & Chvála, 1992

Grootaert and Chvála 1992, **HA**, Chichaoua; Vaňhara and Rozkošný 1997

﻿*Platypalpus ﻿﻿﻿anomalicerus* (Becker, 1902)

= *Coryneta ﻿﻿﻿aerivaga* Séguy, in Séguy 1941d: 11

Séguy 1941d, **EM**, Oued Guir, **AA**, Agadir; Grootaert and Chvála 1992, **EM**, Oued Guir, **AA**, Agadir

﻿*Platypalpus ﻿﻿﻿annulatus* (Fallén, 1815)

Pârvu et al. 2006, **AP**, Merja Zerga; Popescu-Mirceni 2011

﻿*Platypalpus ﻿﻿﻿anomalitarsis* Chvála & Kovalev, 1974

Ebejer et al. 2019, **MA**, 10 km S of Azrou (1775 m), 10 km S of Azrou (1720 m), **AA**, Ziz river (30 km N of Erfoud, 894 m)

﻿*Platypalpus ﻿﻿﻿approximatus* (Becker, 1902)

Grootaert and Chvála 1992, **AP**, Casablanca

﻿*Platypalpusasniensis* Grootaert & Chvála, 1992

Grootaert and Chvála 1992, **HA**, Asni; Vaňhara and Rozkošný 1997

﻿*Platypalpus ﻿﻿﻿calceatus* (Meigen, 1822)

Pârvu et al. 2006, **MA**, Meknès, **AA**, Foum Zghouig

﻿*Platypalpus ﻿﻿﻿chillcotti* Chvála, 1981

Grootaert and Chvála 1992, **MA**, Ifrane

﻿*Platypalpus ﻿﻿﻿chrysonotus* (Strobl, 1899)

Ebejer et al. 2019, **Rif**, Oued Laou (saltmarsh)

﻿*Platypalpus ﻿﻿﻿desertorum* (Becker, 1907)

Ebejer et al. 2019, **AA**, 14 km E of Rich (Errachidia, 1278 m)

﻿*Platypalpus ﻿﻿﻿distichus* Grootaert & Chvála, 1992

Ebejer et al. 2019, **AP**, 9 km SE of Aïn Chouk (Lower Loukous marsh, 6 m), **AA**, Ziz river (10 km S of Errachidia, 1008 m)

﻿*Platypalpus ﻿﻿﻿flavicornis* (Meigen, 1822)

Ebejer et al. 2019, **AA**, 2 km N of Erfoud (818 m), Ziz river (30 km N of Erfoud, 894 m)

﻿*Platypalpus ﻿﻿﻿longicauda* Grootaert & Chvála, 1992

Ebejer et al. 2019, **Rif**, Smir lagoon

﻿*Platypalpus ﻿﻿﻿lyneborgi* Chvála, 1981

Grootaert and Chvála 1992, **AP**, Dradek, **MA**, Azrou

﻿*Platypalpusnigritarsis* (Fallén, 1816)

Pârvu et al. 2006, **MA**, Ifrane; Popescu-Mirceni 2011

﻿*Platypalpus ﻿﻿﻿obscuripes* (Strobl, 1899)

Ebejer et al. 2019, **Rif**, Martil (9 m), **AP**, Larache (Loukous marsh, 2 m)

﻿*Platypalpus ﻿﻿﻿ostiorum* (Becker, 1902)

Grootaert and Chvála 1992

﻿*Platypalpus ﻿﻿﻿pachycerus* (Collin, 1949)

Ebejer et al. 2019, **AA**, Ziz river (10 km S of Errachidia, 1008 m)

﻿*Platypalpus ﻿﻿﻿pallidiventris* (Meigen, 1822)

Grootaert and Chvála 1992, **MA**, Ifrane; Maarouf 2003, **HA**, Chaouia

﻿*Platypalpus ﻿﻿﻿pseudoexiguus* (Strobl, 1909)

Ebejer et al. 2019, **Rif**, Oued Laou (saltmarsh)

﻿*Platypalpus ﻿﻿﻿pseudounguiculatus* (Strobl, 1909)

= ﻿*Tachydromia ﻿﻿﻿pseudounguiculata* Strobl 1909

Grootaert and Chvála 1992

﻿*Platypalpus ﻿﻿﻿riojaensis* Chvála, 1981

Grootaert and Chvála 1992, **EM**, Oujda, **MA**, Meknès, **HA**, Chichaoua

﻿*Platypalpus ﻿﻿﻿turgidus* (Becker, 1907)

Grootaert and Chvála 1992, **MA**, Takkat-n- Sountat

﻿*Platypalpus ﻿﻿﻿vockerothi* Chvála, 1981

Grootaert and Chvála 1992, **HA**, Asni

﻿***Tachydromia* Meigen, 1803**

﻿*Tachydromia ﻿﻿﻿arrogans* (Linnaeus, 1761)

Ebejer et al. 2019, **Rif**, Oued Laou, El-Fahsa (maquis)

﻿*Tachydromia ﻿﻿﻿annulimana* Meigen, 1822^29^

= *Tachista ﻿﻿﻿annulimana* Meigen, in Becker and Stein 1913: 84

Becker and Stein 1913, **Rif**, Tanger

﻿*Tachydromia ﻿﻿﻿undulata* (Strobl, 1906)

Chvála 1969

### ﻿Platypezoidea

#### ﻿﻿PHORIDAE

K. Kettani, H. Disney

Number of species: **3**. Expected: >400

Faunistic knowledge of the family in Morocco: very poor

﻿***Diplonevra* Lioy, 1864**

*Diplonerva ﻿crassicornis* (Meigen, 1830)

= *Phoracrassicornis* Meigen, in Meigen 1830: 220

= *Dohrniphora ﻿﻿﻿dudai* Schmitz, in Schmitz 1920: 100

Raclin 1957; Mouna 1998; **AP** (Rabat) – MISR

﻿*Diplonevra ﻿﻿﻿tangeriana* (Becker, 1913)^30^

= *Phora ﻿﻿﻿tangeriana* Becker and Stein, in Becker and Stein 1913: 90; Schmitz 1949: 238 (*Species ﻿﻿incerta*)

Becker and Stein 1913, **Rif**, Tanger; Schmitz 1949

﻿***Megaselia* Rondani, 1856**

﻿*Megaselia ﻿minor* (Zetterstedt, 1848)

= *Trineura ﻿minor* Zetterstedt, in Zetterstedt 1848: 2864; Wood 1912: 167

= *Aphiochaeta ﻿﻿﻿﻿﻿angustifrons* Wood, in Wood 1912: 167; Disney 1984: 239

= *Phoraminor* Shob, in Mouna 1998: 86

Zetterstedt 1848; Wood 1912; Disney 1984; Mouna 1998; **AP** (Rabat) – MISR

#### ﻿﻿PLATYPEZIDAE

K. Kettani, M.J. Ebejer

Number of species: **3**. Expected: 6

Faunistic knowledge of the family in Morocco: poor

##### Platypezinae

﻿***Lindneromyia* Meigen, 1804**

﻿*Lindneromyia ﻿﻿﻿dorsalis* (Meigen, 1804)

Chandler 2001, **Rif**, Chefchaouen (600 m), **AP**, Rabat, Maâmora; Tkoč and Roháček 2014; **AP** (Maâmora) – MISR

﻿***Microsania* Zetterstedt, 1837**

﻿*Microsaniaraclinae* Collart^31^

Mouna 1998: 86

﻿***Protoclythia* Kessel, 1949**

﻿*Protoclythia ﻿﻿﻿rufa* (Meigen, 1830)

Ebejer et al. 2019, **Rif**, Tahaddart (1 m)

#### ﻿﻿LONCHOPTERIDAE

K. Kettani, M. Barták

Number of species: **4**. Expected: 5

Faunistic knowledge of the family in Morocco: moderate

##### Lonchopterinae

﻿***Lonchoptera* Meigen, 1803**

﻿*Lonchoptera ﻿﻿﻿bifurcata* (Fallén, 1810)

= *Dispa ﻿﻿﻿furcata* Fallén, in Vaillant 1989: 217

= *Muscidora ﻿﻿﻿furcata* Fallén, in Mouna 1998: 86

Vaillant 1989; Mouna 1998

﻿*Lonchoptera ﻿﻿﻿fallax* De Meijere, 1906

= *Muscidora ﻿﻿﻿fallax* Meigen, in Mouna 1998: 86

Mouna 1998

﻿*Lonchopteralutea* Panzer, 1809

= *Muscidora ﻿lutea* Panzer, in Mouna 1998: 86

Vaillant 1989, **HA** (>3000 m); Mouna 1998; Pârvu et al. 2006, **MA**, Ifrane; Popescu-Mirceni 2011; **MA** – MISR

﻿*Lonchoptera ﻿﻿﻿tristis* Meigen, 1824

= *Muscidora ﻿﻿﻿tristis* Meigen, in Mouna 1998: 86

Mouna 1998

### ﻿Syrphoidea

#### ﻿﻿PIPUNCULIDAE

K. Kettani, M.J. Ebejer

Number of species: **16**. Expected: 50

Faunistic knowledge of the family in Morocco: poor

##### Chalarinae

﻿***Chalarus* Walker, 1834**

﻿*Chalarus ﻿﻿﻿brevicaudis* Jervis, 1992

Ebejer and Kettani 2019b, **Rif**, Dardara (730 m), Azilane (1255 m), El Hamma (338 m)

﻿*Chalarus* ﻿sp. ﻿aff. ﻿*brevicaudis* Jervis, 1992

Ebejer and Kettani 2019b, **Rif**, Azilane (1255 m)

##### Pipunculinae

Eudorylini

﻿***Claraeola* Aczél, 1940**

﻿*Claraeola* ﻿sp. ﻿aff. ﻿*halterata* (Meigen, 1838)

Ebejer and Kettani 2019b, **Rif**, Akchour (424 m)

﻿***Clistoabdominalis* Skevington, 2001**

﻿*Clistoabdominalis ﻿﻿﻿dilatatus* (De Meyer, 1997)

Ebejer and Kettani 2019b, **Rif**, Jebel Talassemtane (1546 m), El Hamma (338 m)

﻿***Dasydorylas* Skevington, 2001**

﻿*Dasydorylas ﻿﻿﻿setosus* (Becker, 1908)

Kehlmaier 2005; Motamedinia et al. 2017

﻿***Eudorylas* Aczél, 1940**

﻿*Eudorylas ﻿﻿﻿ibericus* Kehlmaier, 2005

Ebejer and Kettani 2019b, **Rif**, Jebel Talassemtane (1546 m)

##### Pipunculini

﻿***Pipunculus* Latreille, 1802**

﻿*Pipunculuscarlestolrai* Kuznetzov, 1993

Ebejer and Kettani 2019b, **Rif**, Jebel Talassemtane (1546 m)

##### Tomosvaryellini

﻿***Tomosvaryella* Aczél, 1939**

﻿*Tomosvaryella ﻿﻿﻿cilifemorata* (Becker, 1907)

Ebejer and Kettani 2019b, **Rif**, Adrou (PNPB, 556 m)

﻿*Tomosvaryella ﻿﻿﻿debruyni* De Meyer, 1995

Ebejer and Kettani 2019b, **Rif**, Adrou (PNPB, 556 m)

﻿*Tomosvaryella ﻿﻿﻿frontata* (Becker, 1897)

Ebejer and Kettani 2019b, **Rif**, Oued Mhajrate (Ben Karrich, 67 m)

﻿*Tomosvaryella ﻿﻿﻿geniculata* (Meigen, 1824)

Ebejer and Kettani 2019b, **HA**, Lac Tislite (Imilchil, 2254 m)

﻿*Tomosvaryella ﻿﻿﻿kuthyi* Aczél, 1944

Ebejer and Kettani 2019b, **Rif**, El Hamma (338 m), Akchour (424 m), Barrage Smir (27 m)

﻿*Tomosvaryella ﻿﻿﻿minima* (Becker, 1897)

Ebejer and Kettani 2019b, **Rif**, Koudiat Taifour (100 m)

﻿*Tomosvaryella ﻿﻿﻿mutata* (Becker, 1898)

Ebejer and Kettani 2019b, **Rif**, Jebel Lakraâ (Talassemtane, 1596 m)

﻿*Tomosvaryella ﻿﻿﻿trichotibialis* De Meyer, 1995

Ebejer and Kettani 2019b, **Rif**, Koudiat Taifour (100 m)

﻿Tomosvaryella﻿sp.subvirescens group

Ebejer and Kettani 2019b, **AP**, Loukous marsh (Larache)

#### ﻿﻿SYRPHIDAE

K. Kettani, M.C.D. Speight

Number of species: **166**. Expected: more than 200

Faunistic knowledge of the family in Morocco: moderate

##### Eristalinae

Brachyopini

﻿***Brachyopa* Meigen, 1822**

﻿*Brachyopa ﻿﻿﻿atlantea* Kassebeer, 2000

Kassebeer 2000, **HA**, Ouirgane (1000 m); Speight 2018; Sahib et al. 2020

﻿***Chrysogaster* Meigen, 1803**

﻿*Chrysogaster ﻿﻿﻿basalis* Loew, 1857

Claußen and Hauser 1990, **MA**, Ifrane (1750 m); Dirickx 1994; Kassebeer 1999d; Sahib et al. 2020

﻿***Ighboulomyia* Kassebeer, 1999**

﻿*Ighboulomyia ﻿﻿﻿atlasi* Kassebeer, 1999

Kassebeer 1999c, **MA**, Azrou, Umgebung, Timahdit, Ighböula Ulaichuor, Quellteich; Sahib et al. 2020

﻿***Myolepta* Newman, 1838**

﻿*Myolepta ﻿﻿﻿difformis* Strobl in Czerny & Strobl 1909

= ﻿*Myolepta ﻿﻿﻿philonis* Séguy, 1961, in Dirickx 1994: 93

Dirickx 1994, **HA**; Reemer et al. 2004, **MA**, **HA**; Speight 2013, 2018; Sahib et al. 2020

﻿***Neoascia* Williston, 1886**

﻿*Neoascia ﻿clausseni* Hauser & Kassebeer, 1998

= ﻿*Neoascia ﻿﻿﻿podagrica* (Fabricius, 1775), in Gil Collado 1929: 40; Claussen 1989b: 373

Gil Collado 1929a; Claußen 1989b; Dirickx 1994; Hauser and Kassebeer 1998, **MA**, **HA**, Taroudant (1800 m); Pârvu et al. 2006, **AP**, Merja Zerga; Popescu-Mirceni 2011; Sahib et al. 2020, **Rif**, Oued Jnane Niche, Oued Maggou

﻿***Orthonevra* Macquart, 1829**

﻿*Orthonevra ﻿﻿﻿bouazzai* Kassebeer, 1999

Kassebeer 1999d, **MA**; Sahib et al. 2020

﻿*Orthonevra ﻿﻿﻿brevicornis* (Loew, 1843)^32^

Sahib et al. 2020, **Rif**, Aïn Afersiw

﻿*Orthonevra ﻿﻿﻿elegans* (Meigen, 1822)

Becker and Stein 1913, **Rif**; Claußen 1989b; Dirickx 1994; Sahib et al. 2020

﻿*Orthonevra ﻿﻿﻿schachti* Claußen, 1989b

Claußen 1989b, **HA**, Oukaimeden (2600 m); Dirickx 1994; Schmid 1995; Kassebeer 1999d, **MA**; Sahib et al. 2020

﻿***Riponnensia* Maibach, Goeldlin & Speight, 1994**

﻿*Riponnensia ﻿﻿﻿longicornis* (Loew, 1843)

= ﻿*Orthonevra ﻿﻿﻿longicornis* Loew, in Kanervo 1939: 2; Séguy 1961: 23; Kassebeer 1999c: 162

Kanervo 1939; Séguy 1961, **AP**; Claußen 1989b; Kassebeer 1999c, **MA**, **HA**; Dirickx 1994; Dousti and Hayat 2006; Speight 2013, 2018; Sahib et al. 2020

﻿*Riponnensia ﻿﻿﻿splendens* (Meigen, 1822)

= ﻿*Chrysogaster ﻿﻿﻿splendens* (Meigen), in Gil Collado 1929: 405

= ﻿*Orthonevra ﻿﻿﻿splendens* (Meigen), in Claußen 1989b: 363, 373; Dirickx 1994: 97

Gil Collado 1929a, **Rif**; Mouna 1998; Claußen 1989b, **HA**, Tizi-n’Test (1900 m); Dirickx 1994; Kassebeer 1999c, **MA**; Sahib et al. 2020, **MA**, Douar Zaouiat Cheikh; **AP** (Dradek) – MISR

##### Callicerini

﻿***Callicera* Panzer, 1806**

﻿*Callicera ﻿﻿﻿fagesi* Guérin-Meneville, 1844

Kassebeer 1998a, **HA**, Ouirgane (1000 m); Sahib et al. 2020

﻿*Callicera ﻿﻿﻿rufa* Schummel, 1842

Gil Collado 1929a, **Rif**, Tanger; Claußen 1989b; Dirickx 1994; Sahib et al. 2020

##### Cerioidini

﻿***Ceriana* Rafinesque, 1815**

﻿*Ceriana ﻿﻿﻿conopsoides* (Linnaeus, 1758)

= *Cerioides ﻿﻿﻿conopsoides* Linnaeus, in Séguy 1930a: 131

Séguy 1930a, **MA**, Ras El Ksar; Claußen 1989b; Dirickx 1994; Mouna 1998; Sahib et al. 2020; **AP** (Maghrawa, Maâmora) – MISR

﻿*Ceriana ﻿﻿﻿vespiformis* (Latreille, 1804)

= *Cerioides ﻿﻿﻿vespiformis* Latreille, in Becker and Stein 1913: 88; Gil Collado 1929: 414; Séguy 1930a: 131; Kanervo 1939: 5; Leclercq 1961: 242

Becker and Stein 1913, **Rif**, Tanger; Gil Collado 1929a, **Rif**, **HA**; Séguy 1930a, **AP**, Rabat, Casablanca, **MA**, Tizi-s’Tkrine, Aïn Leuh, Meknès; Kanervo 1939, **MA**; Leclercq 1961a, **Rif**, Melillia, **MA**, Dayat Aoua, Aïn Leuh; Claußen 1989b; Dirickx 1994; Steenis et al. 2016; Speight 2018; El Hawagry and Gilbert 2019; Sahib et al. 2020, **Rif**, 1 km after Dardara, Meadow Mizoghar, Oued Achekrade

﻿***Sphiximorpha* Rondani, 1850**

﻿*Sphiximorpha ﻿﻿﻿subsessilis* (Illiger in Rossi, 1807)

Steenis et al. 2016

##### Eristalini

﻿***Anasimyia* Schiner, 1864**

﻿*Anasimyia ﻿﻿﻿contracta* Caußen & Torp, 1980

Kassebeer 1998a, **MA**, Timahdit (1850 m); Sahib et al. 2020

﻿***Eristalinus* Rondani, 1845**

﻿*Eristalinus ﻿﻿﻿﻿﻿aeneus* (Scopoli, 1763)

= ﻿*Lumpetia ﻿﻿﻿﻿﻿aenea* (Scopoli), in Becker and Stein 1913: 86

= ﻿*Eristalis ﻿﻿﻿﻿﻿aeneus* (Scopoli), in Gil Collado 1929: 406, 407; Leclercq 1961: 242

= *Lathyrophtalmus ﻿﻿﻿﻿﻿aeneus* (Scopoli), in Séguy 1930: 129; Kanervo 1939: 5

Becker and Stein 1913, **Rif**, Tanger; Gil Collado 1929a, **Rif**, Tanger, **AP**, Mogador; Séguy 1930a, **AP**, Casablanca; Kanervo 1939; Timon-David 1951, **AP**, Rabat; Leclercq 1961a, **EM**, Melilla; Claußen 1989b; Dirickx 1994; Pârvu and Zaharia 2007; Sahib et al. 2020, **Rif**, Jumb Kitane, **HA**, vicinity of Asni

﻿*Eristalinus ﻿﻿﻿megacephalus* (Rossi, 1794)

= ﻿*Eristalis ﻿﻿﻿quinquelineatus* Fabricius, in Becker and Stein 1913: 85, Gil Collado 1929: 407,

= *Lathyrophthalmus ﻿﻿﻿quinquelineatus* Fabricius, in Séguy 1930a: 129

Gil Collado 1929a, **Rif**, Tanger; Séguy 1930a, **AP**, Rabat, Oued Korifla, Sidi Bettache; Séguy 1961; Dakki 1997; Claußen 1989b; Dirickx 1994; Dousti and Hayat 2006; Sahib et al. 2020; **AP** (Rabat) – MISR

﻿*Eristalinus ﻿﻿﻿sepulchralis* (Linnaeus, 1758)

= ﻿*Eristalis ﻿﻿﻿sepuleralis* Linnaeus, in Becker and Stein 1913: 85, Gil Collado 1929: 406

Becker and Stein 1913, **Rif**, Tanger; Gil Collado 1929a, **AP**, Claußen 1989b, **HA**, Ansegmir-Tal W Midelt (1400 m); Dirickx 1994; El Hawagry and Gilbert 2019; Sahib et al. 2020

﻿*Eristalinus ﻿﻿﻿taeniops* (Wiedemann, 1818)

= ﻿*Eristalis ﻿﻿﻿taeniops* Wiedemann, in Becker and Stein 1913: 85

= *Eristalodes ﻿﻿﻿taeniops* Wiedemann, in Séguy 1930a: 130

Becker and Stein 1913, **Rif**, Tanger; Séguy 1930a, **AP**, Oued Korifla, Rabat, **HA**, Tenfecht (Takeljount); Leclercq 1961a, **MA**, Dayet Aoua; Claußen and Hauser 1990, **MA**, Ifrane (1750 m); Dirickx 1994; Dakki 1997; Dousti and Hayat 2006; Koçak and Kemal 2010; Speight 2013, 2018; El Hawagry and Gilbert 2019; Sahib et al. 2020, **Rif**, Oued Martil, Halouma Kitane, Oued Sidi Yahia Aârab, **MA**, bridge Oued Oum-er-Rbia (Douar Ahl Souss), **HA**, Lac Oukaimeden; **AP** (Rabat), **MA** (Volubilis) – MISR

﻿***Eristalis* Latreille, 1804**

﻿*Eristalis ﻿﻿﻿arbustorum* (Linnaeus, 1758)

Becker and Stein 1913, **Rif**, Tanger; Gil Collado 1929a, **Rif**; Séguy 1930a, **MA**, Aïn Leuh, Ras El Ksar, forest of Taffert; Kanervo 1939, **Rif**; Timon-David 1951, **AP**, Rabat, **MA**, Ifrane; Claußen 1989b, **MA**, Azrou (1700 m); Claußen and Hauser 1990, **MA**, Ifrane (1750 m); Dirickx 1994; Dakki 1997; El Hawagry and Gilbert 2019; Sahib et al. 2020, **Rif**, Aïn Sidi Brahim Ben Arrif, **MA**, vicinity of Ifrane, **HA**, vicinity of Asni, Lac Oukaimeden, **AA**, Douar Issafen, Douar Issafen; **MA**, **HA** – MISR

﻿*Eristalis ﻿﻿﻿jugorum* Egger, 1858^33^

Dakki 1997

﻿*Eristalis ﻿﻿﻿pertinax* (Scopoli, 1763)

Séguy 1930a, **AP**, Oued Korifla, Sidi Bettache, **MA**, Forêt de Timelilt; Claußen 1989b; Dirickx 1994; Dakki 1997; Sahib et al. 2020

﻿*Eristalis ﻿﻿﻿similis* (Fallén, 1817)

= ﻿*Eristalis ﻿﻿﻿pratorum* Meigen, in Gil Collado 1929: 407

Gil Collado 1929a, **Rif**; Séguy 1961; Claußen 1989b, **HA**, Oukaimeden (2600 m); Dirickx 1994; Pârvu and Zaharia 2007; Sahib et al. 2020, **Rif**, maison forestière, **HA**, Douar Zaouiat Cheikh, Lac Oukaimeden

﻿*Eristalis ﻿﻿﻿tenax* (Linnaeus, 1758)

= *Eristalomyia ﻿﻿﻿tenax* Linnaeus, in Séguy 1930a: 130; Timon-David 1951: 146

Gil Collado 1929a, **Rif**, **AP**; Kanervo 1939, **MA**; Séguy 1949a, **AA**; Timon-David 1951; Leclercq 1961a; Claußen 1989b; Dirickx 1994; Sahib et al. 2020, **Rif**, Village Sebt Zinnat, Belyounech, Aïn Takhninjoute, maison forestière, Jumb Kitane, meadow Fahs Lmhar, **HA**, Douar Zaouiat Cheikh, vicinity of Asni, Lac Oukaimeden

﻿***Helophilus* Meigen, 1822**

﻿*Helophilus ﻿﻿﻿trivittatus* (Fabricius, 1805)

Becker and Stein 1913, **Rif**, Tanger; Gil Collado 1929a; Claußen 1989b; Dirickx 1994; Sahib et al. 2020

﻿***Mallota* Meigen, 1822**

﻿*Mallota ﻿﻿﻿cimbiciformis* (Fallén, 1817)

= ﻿*Mallota ﻿﻿﻿eristaloides* Loew, in Becker and Stein 1913: 85

Becker and Stein 1913, **Rif**, Tange; Claußen 1989b; Dirickx 1994; Sahib et al. 2020

﻿*Mallota ﻿﻿﻿dusmeti* Andreu, 1926

Kassebeer 1998a, **HA**, Ouirgane; Sahib et al. 2020

﻿***Melanogaster* Rondani, 1857**

﻿*Melanogaster ﻿﻿﻿lindbergi* Kassebeer, 1999

= ﻿*Chrysogaster ﻿﻿﻿macquardti* Loew, in Becker and Stein 1913: 87

= ﻿*Chrysogaster ﻿﻿﻿viduata* Meigen, in Kanervo 1939: 2, Séguy 1961: 27, 28

= ﻿*Chrysogaster ﻿﻿﻿lucida* (Scopoli), in Claußen 1989b: 372

Becker and Stein 1913, **Rif**; Kanervo 1939, **MA**; Séguy 1961; Claußen 1989b; Dirickx 1994; Kassebeer 1999d, **MA**; Popov et al. 2020; Sahib et al. 2020

﻿﻿***Myathropa* Rondani, 1845**

﻿*Myathropa ﻿﻿﻿florea* (Linnaeus, 1758)

Becker and Stein 1913, **Rif**, Tanger; Gil Collado 1929a; Séguy 1930a, **MA**, Aïn Leuh; Claußen 1989b, **MA**, Azrou (1700 m); Claußen and Hauser 1990, **MA**, Ifrane (1750 m); Dirickx 1994; Dakki 1997; Sahib et al. 2020, **Rif**, Oued à 15 km de Fifi

﻿***Parhelophilus* Girschner, 1897**

﻿*Parhelophilusversicolor* (Fabricius, 1794)

= ﻿*Helophilusversicolor* (Fabricius), in Gil Collado 1929: 407

Gil Collado 1929a, **AP**, Oulad Mesbah; Claußen 1989b; Dirickx 1994; El Hawagry and Gilbert 2019; Sahib et al. 2020

##### Eumerini

﻿***Eumerus* Meigen, 1822**

﻿*Eumerus ﻿﻿﻿amoenus* Loew, 1848

Séguy 1961; Claußen 1989b; Dirickx 1994; Dousti and Hayat 2006; Speight 2013, 2018; Sahib et al. 2020, **AA**, Douar Aourir, beach of Tamelallt

﻿*Eumerus ﻿barbarus* (Coquebert, 1804)

= ﻿*Eumerus ﻿﻿﻿australis* Meigen, in Gil Collado 1929: 412

Becker and Stein 1913, **Rif**, Tanger; Gil Collado 1929a, **Rif**; Séguy 1961; Claußen 1989b, **MA**, Azrou (1900 m); Dirickx 1994; Mouna 1998; Speight 2013, 2018; Steenis et al. 2017, **AA**, 11 km NW Taliouine, S Aït-Baha, 10 km NE Tafraoute; El Hawagry and Gilbert 2019; Sahib et al. 2020; **AP** (Cap Cantin) – MISR

﻿*Eumerus ﻿﻿﻿basalis* Loew, 1848

= ﻿*Eumerus ﻿﻿﻿angusticornis* Rondani, in Séguy 1930a: 130

= ﻿*Eumerus ﻿﻿﻿basalis* Loew, in Mouna 1998: 86

Séguy 1930a, **MA**, forest of Timelilt; Claußen 1989b; Dirickx 1994; Mouna 1998; Sahib et al. 2020

﻿*Eumerus ﻿﻿﻿caballeroi* Gil Collado, 1929

Gil Collado 1929a, **AP**, Laguna Gedira; Claußen 1989b; Dirickx 1994; Speight 2013, 2018; Sahib et al. 2020

﻿*Eumerus ﻿﻿﻿hungaricus* Szilády, 1940

Speight 2018

﻿*Eumerus ﻿﻿﻿lunatus* (Fabricius, 1794)

= ﻿*Eumerus ﻿﻿﻿lunulatus* Fabricius, in Becker and Stein 1913: 86

Becker and Stein 1913, **Rif**, Tanger; Leclercq 1961a, **EM**, Melilla; Claußen 1989b; Dirickx 1994; Mouna 1998; Dousti and Hayat 2006; Speight 2013, 2018; Sahib et al. 2020

﻿*Eumerus ﻿﻿﻿melotus* (Séguy, 1941)

= *Lampetia ﻿﻿﻿melota* Séguy, in Séguy 1941d: 13

Séguy 1941d, **AA**, Agadir; Claußen 1989b; Dirickx 1994; Mouna 1998; Sahib et al. 2020

﻿*Eumerus ﻿﻿﻿nudus* Loew, 1848

Becker and Stein 1913, **Rif**, Tanger; Claußen 1989b; Claußen and Hauser 1990, **MA**, Ifrane (1750 m); Dirickx 1994; Speight 2013, 2018; Sahib et al. 2020

﻿*Eumerus ﻿﻿﻿obliquus* (Fabricius, 1805)

Sahib et al. 2020, **Rif**, Oued Jnane Niche, **EM**, Oued Khemis

﻿*Eumerus ﻿﻿﻿ornatus* Meigen, 1822

Séguy 1930a, **MA**, Aïn Leuh, forest of Timelilt; Claußen 1989b; Dirickx 1994; Mouna 1998; Sahib et al. 2020; **AP** (Oued Cherrat) – MISR

﻿*Eumerus ﻿﻿﻿pulchellus* Loew, 1848

Séguy 1961; Claußen 1989b; Dirickx 1994; Sahib et al. 2020

﻿*Eumerus ﻿﻿﻿punctifrons* Loew, 1857

Leclercq 1961a, **EM**, Melilla; Claußen 1989b; Dirickx 1994; Mouna 1998; Sahib et al. 2020

﻿*Eumerus ﻿﻿﻿pusillus* Loew, 1848

Claußen 1989b, **HA**, Tizi-n’Test (1900 m); Dirickx 1994; Sahib et al. 2020

﻿*Eumerus ﻿﻿﻿sabulonum* (Fallén, 1817)

Séguy 1961; Claußen 1989b; Dirickx 1994; Sahib et al. 2020

﻿*Eumerus ﻿﻿﻿schmideggeri* Steenis, Hauser & Zuijen, 2017

Steenis et al. 2017, **AA**, Sidi R’bat (37 km S Agadir); Sahib et al. 2020

﻿*Eumerus ﻿﻿﻿strigatus* (Fallén, 1817)^34^

Kanervo 1939, **Rif**, **HA**; Timon-David 1951, **AP**, Rabat, Sehoul; Claußen 1989b, **MA**, Azrou (1700 m), **HA**, Oukaimeden (2200 m), Ansegmir-Tal W Midelt; Claußen and Hauser 1990, **MA**, Ifrane, Hajeb; Dirickx 1994; Mouna 1998; Speight 2013; Sahib et al. 2020

﻿*Eumerus ﻿﻿﻿subornatus* Claußen, 1989b

Claußen 1989b, **HA**, Tizi-n’Test (1900 m); Schmid 1995; Speight 2013, 2018; Dirickx 1994; Sahib et al. 2020

﻿*Eumerus ﻿﻿﻿truncatus* Rondani, 1868

Steenis et al. 2017, **AA**, S. Aït-Baha, 11 km NW Taliouine, 25 km NE Tizinit, 20 km E Tizinit, Assaka; Speight 2018; Sahib et al. 2020

﻿***Merodon* Meigen, 1803^35^**

﻿*Merodon ﻿﻿﻿aberrans* Egger, 1860

= *Lampetia ﻿﻿﻿aberrans* Egger, in Séguy 1961: 174

Séguy 1961; Claußen 1989b; Dirickx 1994; Marcos-Garcia et al. 2007; Koçak and Kemal 2010; Vujic et al. 2011; Speight 2013, 2018; Sahib et al. 2020

﻿*Merodon ﻿﻿﻿﻿﻿aeneus* Meigen, 1822^36^

= *Lampetia ﻿﻿﻿﻿﻿aenea* Meigen, in Becker and Stein 1913: 86; Kanervo 1939: 5; Timon-David 1951: 146

Becker and Stein 1913, **Rif**, Tanger; Gil Collado 1929a; Séguy 1930a, **AP**, Vallée Oued Korifla, **MA**, Tizi-s’Tkrine; Kanervo 1939, **Rif**; Timon-David 1951; Séguy 1961; Claußen 1989b; Dirickx 1994; Mouna 1998; Sahib et al. 2020

﻿*Merodon ﻿﻿﻿arrasus* Becker, 1921^37^

Becker 1921, **Rif**, Tanger; Claußen 1989b; Dirickx 1994; Sahib et al. 2020

﻿*Merodon ﻿﻿﻿aurifer* Loew, 1862

= ﻿*Merodon ﻿﻿﻿distinctus* Palma, 1864

= *Lampetia ﻿﻿﻿distincta* Palm, in Timon-David 1951: 146

Timon-David 1951, **AP**, Zaer, **MA**, Ifrane; Claußen 1989b; Dirickx 1994; Mouna 1998; Sahib et al. 2020; Vujić et al. 2021c

﻿*Merodon ﻿avidus* Rossi, 1782^38^

= *Lampetia ﻿﻿﻿spinipes* (Fabricius), in Becker and Stein 1913: 86; Timon-David 1951: 146

= ﻿*Merodon ﻿﻿﻿spinipes* (Fabricius), in Gil Collado 1929: 409

= *Lampetia ﻿﻿﻿avida* Rossi, in Séguy 1961: 176

Becker and Stein 1913, **Rif**, Tanger; Gil Collado 1929a, **Rif**, **HA**; Séguy 1930a, **AP**, Chellah (Rabat), Oued Korifla, **MA**, Aïn Leuh; Kanervo 1939; Timon-David 1951, **AP**; Séguy 1961; Claußen 1989b, **HA**, Oukaimeden (2600 m); Hurkmans 1993; Dirickx 1994; Mouna 1998; Koçak and Kemal 2010; Likov et al. 2020; Sahib et al. 2020, **HA**, Lac Oukaimeden

﻿*Merodon ﻿﻿﻿bequaerti* Hurkmans, 1993

Vujić et al. 2020a, **EM**, Mountain of Beni-Snassen, **MA**, Azrou

﻿*Merodon ﻿﻿﻿cabanerensis* Marcos-García, Vujić & Mengual, 2007

Vujić et al. 2018, **HA**, Ait Mhamed (Azilal, 1700 m); Speight 2018; Sahib et al. 2020; Vujić et al. 2021

﻿*Merodon ﻿﻿﻿calcaratus* (Fabricius, 1794)

Vujić et al. 2021b, **EM**, Mountains of Béni Snassen, near Nador, **AP**, 38 km SW of El Jadida, Garbouz

﻿*Merodon ﻿﻿﻿chalybeus* Wiedemann in Meigen, 1822

= *Lampetia ﻿﻿﻿spicata* Becker, in Timon-David 1951: 146

= ﻿*Merodon ﻿﻿﻿spicatus* Becker, in Claußen 1989b: 365, 373

Timon-David 1951, **AP**, forest of Maâmora; Claußen 1989b, **HA** (2500 m); Dirickx 1994; Mouna 1998; Marcos-Garcia et al. 2007; Speight 2013, 2018; Sahib et al. 2020; **AP** (Cap Cantin) – MISR

﻿*Merodon ﻿﻿﻿clavipes* (Fabricius, 1781)

Hurkmans 1993, **MA**; Marcos-García et al. 2007; Koçak and Kemal 2010; Sahib et al. 2020

﻿*Merodon ﻿﻿﻿eques* Fabricius, 1805

= *Lampetia ﻿﻿﻿eques* (Fabricius), in Séguy 1961: 178

Séguy 1961; Claußen 1989b; Dirickx 1994; Ebejer and Bensusan 2010, **AA**; El Hawagry and Gilbert 2019; Sahib et al. 2020

﻿*Merodon ﻿﻿﻿equestris* (Fabricius, 1794)

= ﻿*Eristalis ﻿﻿﻿ferrugineus* (Fabricius), in Fabricius 1805: 240

= *Lampetia ﻿﻿﻿equestris* (Fabricius), in Séguy 1961: 178, 179

Fabricius 1805, **AP**; Séguy 1961; Claußen 1989b; Dirickx 1994; Sahib et al. 2020

﻿*Merodon ﻿﻿﻿escalerai* Gil Collado, 1929

Gil Collado 1929a, **AP**, Essaouira; Claußen 1989b; Dirickx 1994; Speight 2018; Sahib et al. 2020

﻿*Merodon ﻿﻿﻿femoratus* Sack, 1913

= ﻿*Merodon ﻿﻿﻿biarcuatus* Curran, 1939, in Curran 1939: 6, 7; Claußen 1989: 373; Dirickx 1994: 79; Koçak and Kemal 2010: 1199; Speight 2018: 137

= ﻿*Merodonelegans* Hurkmans, 1993, in Hurkmans 1993: 195; Schmid 1995; Marcos-Garcia et al. 2007: 553; Speight 2018: 141

Curran 1939, **AP**, forest of Maâmora (Rabat); Claußen 1989b; Hurkmans 1993, **AP**; Dirickx 1994; Schmid 1995; Marcos-Garcia et al. 2007; Koçak and Kemal 2010; Speight 2018; Likov et al. 2020

﻿*Merodon ﻿﻿﻿geniculatus* Strobl, 1909

Gil Collado 1929a, **Rif**; Claußen 1989b, **HA** (2500 m); Dirickx 1994; Mouna 1998; Marcos-Garcia et al. 2007; Koçak and Kemal 2010; Speight 2013, 2018; Sahib et al. 2020, **HA**, Lac Oukaimeden – MISR

﻿*Merodon ﻿﻿﻿hurkmansi* Marcos-García, Vujić & Mengual, 2007

Marcos-García et al. 2007

﻿*Merodon ﻿﻿﻿ibericus* Vujić, 2015

= ﻿*Merodon ﻿﻿﻿bicolor* Gil Collado, 1930

Popović et al. 2015, **MA**, Azrou, Ifrane; Acanski et al. 2016; Speight 2018; Sahib et al. 2020

﻿*Merodon ﻿﻿﻿italicus* Rondani, 1845

Claußen and Hauser 1990, **MA**, Ifrane (1750 m); Mouna 1998

﻿*Merodon ﻿﻿﻿longicornis* Sack, 1913

Claußen and Hauser 1990, **MA**; Dirickx 1994; Sahib et al. 2020

﻿*Merodonmaroccanus* Gil Collado, 1929

Gil Collado 1929a, **AP**, Essaouira; Claußen 1989b; Dirickx 1994; Sahib et al. 2020

﻿*Merodon ﻿﻿﻿minutus* Strobl, 1893

= *Lampetia ﻿﻿﻿minutus* Strobl, in Séguy 1961: 180

Séguy 1961; Leclercq 1961a; Claußen 1989b; Dirickx 1994; Speight 2013, 2018; Sahib et al. 2020

﻿*Merodon ﻿﻿﻿monticolus* Villeneuve, 1924

Kassebeer 1998a, **HA**, Ouirgane, Taftraoute, Taliouine; Sahib et al. 2020

﻿*Merodon ﻿﻿﻿murorum* (Fabricius, 1794)

= ﻿*Syrphus ﻿﻿﻿murorum* (Fabricius), in Fabricius 1794: 288

= ﻿*Merodon ﻿﻿﻿auripilus* (Meigen), in Meigen 1830: 354

= *Lampetia ﻿﻿﻿auripila* Meigen, in Séguy 1941d: 13; Mouna 1998: 86

Fabricius 1794; Meigen 1830; Séguy 1941d, **AA**, Agadir; Séguy 1961; Claußen 1989b; Dirickx 1994; Mouna 1998; Vujić et al. 2018, **AP**, Essaouira; Sahib et al. 2020

﻿*Merodon ﻿﻿﻿pruni* (Rossi, 1790)

= *Lampetia ﻿﻿﻿pruni* (Rossi), in Becker and Stein 1913: 86

= ﻿*Merodon﻿prunivar.﻿obscurus* Gil Collado, in Gil Collado 1929: 407, 408

Becker and Stein 1913; Gil Collado 1929a, **Rif**, Tanger; Claußen 1989b; Hurkmans, 1993; Dirickx 1994; Koçak and Kemal 2010; Sahib et al. 2020

﻿*Merodon ﻿﻿﻿pumilus* Macquart, 1849

Ebejer et al. 2019, **Rif**, Moulay Abdelsalam (965 m); Sahib et al. 2020

﻿*Merodon ﻿﻿﻿rufus* Meigen, 1838

= *Lampetia ﻿﻿﻿rufa* Meigen, 1838, in Becker and Stein 1913: 86

Becker and Stein 1913, **Rif**; Claußen 1989b; Dirickx 1994; Sahib et al. 2020

﻿*Merodon ﻿﻿﻿segetum* (Fabricius, 1794)

Peck 1988; Claußen 1989b; Dirickx 1994; Sahib et al. 2020

﻿*Merodon ﻿serrulatus* Wiedemann in Meigen, 1822

Hurkmans 1993; Speight 2013, 2018; Sahib et al. 2020; Vujić et al. 2020a

﻿*Merodon ﻿﻿﻿sophron* Hurkmans, 1993

Hurkmans 1993, **MA**, Azrou; Schmid 1995; Koçak and Kemal 2010; Vujić et al. 2020a, **MA**, Azrou

﻿*Merodon ﻿﻿﻿tangerensis* Hurkmans, 1993

Hurkmans 1993, **Rif**, Tanger; Schmid 1995; Koçak and Kemal 2010; Sahib et al. 2020

﻿*Merodon ﻿﻿﻿tricinctus* Sack, 1913

= *Lampetia ﻿﻿﻿tricincta* Sack, in Timon-David 1951: 146

Timon-David 1951, **MA**, Ifrane; Claußen 1989b; Dirickx 1994; Mouna 1998; Sahib et al. 2020

﻿*Merodon ﻿﻿﻿unguicornis* Strobl, 1909

Ebejer et al. 2019, **MA**, 10 km S of Azrou (1775 m); Sahib et al. 2020, **Rif**, maison forestière

﻿***Platynochaetus* Wiedemann, 1830**

﻿*Platynochaetus ﻿﻿﻿rufus* Macquart, 1835

Gil Collado 1929a, **AP**, Mogador; Dirickx 1994; Sahib et al. 2020

﻿*Platynochaetus ﻿﻿﻿setosus* (Fabricius, 1794)

Gil Collado 1929a, **HA**, Marrakech; Séguy 1953a, **AA**, Souss: Aïn Chaib; Claußen 1989b; Dirickx 1994; Speight 2013, 2018; Sahib et al. 2020

##### Milesiini

﻿***Milesia* Latreille, 1804**

﻿*Milesia ﻿﻿﻿crabroniformis* (Fabricius, 1775)

Claußen and Hauser 1990, **MA**, Ifrane, Hajeb; Dirickx 1994; Sahib et al. 2020

﻿***Spilomyia* Meigen, 1803**

﻿*Spilomyia ﻿maroccana* Kuznetzov, 1997

= ﻿*Spilomyia ﻿﻿﻿digitata* (Rondani), in Becker and Stein 1913: 86

Becker and Stein 1913, **Rif**, Tanger; Claußen 1989b, **HA**, Tizi-n’Test (1900 m); Claußen and Hauser 1990, **MA**, Ifrane, Hajeb; Kuznetzov 1997; Dirickx 1994; Kassebeer 1999d; Steenis 2000; Sahib et al. 2020

﻿***Syritta* Le Peletier & Serville, 1828**

﻿*Syritta ﻿﻿﻿flaviventris* Macquart, 1842

Claußen 1989b, **AP**, Kénitra; Dirickx 1994; Sahib et al. 2020, **EM**, farm Saf-Saf

﻿*Syrittapipiens* (Linnaeus, 1758)

Becker and Stein 1913, **Rif**, Tanger; Gill Collado 1929a; Séguy 1930a, **AP**, Rabat, Casablanca, **MA**, Meknès; Kanervo 1939; Timon-David 1951, **AP**, Rabat, **AA**, Agdz; Leclercq 1961a, **EM**, Melilla; Claußen 1989b, **HA**, Tizi-n’Test (1900 m); Claußen and Hauser 1990, **MA**, Ifrane, Hajeb; Dirickx 1994; Mouna 1998; Pârvu et al. 2006, **AP**, Merja Zerga; Popescu-Mirceni 2011; Sahib et al. 2020, **Rif**, Bni Maaden, dam Moulay Bouchta, Oued Koub, Douar Kitane, Oued Maggou, **EM**, farm Saf-Saf, **MA**, vicinity of Ifrane, **HA**, vicinity of Asni, Tizi-n’Test, **AA**, Oued Assa; **AP** (Rabat), **MA** (Aïn Leuh), **SA** – MISR

﻿***Temnostoma* Le Peletier & Serville, 1828**

﻿*Temnostoma ﻿﻿﻿bombylans* (Fabricius, 1805)

Séguy 1961, **MA**; Claußen 1989b; Dirickx 1994; Sahib et al. 2020

﻿***Xylota* Meigen, 1822**

﻿*Xylota ﻿﻿﻿segnis* (Linnaeus, 1758)

= *Zelima (Xylota) ﻿segnis* Linnaeus, in Becker and Stein 1913: 86; Timon-David 1951: 147

Becker and Stein 1913, **Rif**, Tanger; Gil Collado 1929a; Timon-David 1951, **HA**, Zaouia Ahansal; Leclercq 1961a, **Rif**, Azib de Ketama; Claußen 1989b; Claußen and Hauser 1990, **MA**, Ifrane (1750 m); Dirickx 1994; Mouna 1998; Sahib et al. 2020, **Rif**, Aïn El Maounzil, Oued Koub, Oued Sidi Ben Saâda, **HA**, vicinity of Asni; **HA** (Zaouiet Ahansal) – MISR

##### Rhingiini

﻿***Cheilosia* Meigen, 1822**

﻿*Cheilosia ﻿﻿﻿brunnipennis* Becker, 1894

= *Chilosia ﻿flavipes* (Panzer), in Kanervo 1939: 2

Kanervo 1939, **HA**; Kassebeer, 1998c, **MA**; Séguy 1961; Claußen 1989b; Speight 2013, 2018; Sahib et al. 2020

﻿*Cheilosiaflavipes* (Panzer, 1798)

Mouna 1998: 86

﻿*Cheilosia ﻿﻿﻿grossa* (Fallén, 1817)

Kassebeer 1998c, **HA**, Asif Mellah, Tizi-n’Tichka; Speight 2013, 2018; Sahib et al. 2020

﻿*Cheilosia ﻿﻿﻿latifrons* (Zetterstedt, 1843)

= ﻿*Cheilosia ﻿﻿﻿intonsa* Loew, in Timon-David 1951: 144

Timon-David 1951, **AP**, Sehoul; Claußen 1989b; Dirickx 1994; Mouna 1998; Kassebeer 1998c, **Rif**, Ouezzane, **AP**, Oued Loukous, Larache, **MA**, Ifrane, **HA**, Oukaimeden; Sahib et al. 2020

﻿﻿*Cheilosia ﻿﻿﻿mutabilis* (Fallén, 1817)

Speight 2013, 2018

﻿*Cheilosia ﻿﻿﻿griseiventris* Loew, 1857

= *Chilosia ﻿﻿﻿marokkana* Becker, in Becker 1894: 395; Becker and Stein 1913: 87

= ﻿*Cheilosia ﻿maroccana* Becker, 1894, in Gil Collado 1929: 405

Becker 1894; Becker and Stein 1913; Gil Collado 1929a; Séguy 1961; Claußen 1989b; Dirickx 1994; Kassebeer 1998c, **MA**, **HA**; Sahib et al. 2020

﻿*Cheilosia ﻿﻿﻿paralobi* Malski, 1962

= ﻿*Cheilosia ﻿﻿﻿longula* (Zetterstedt, 1838), in Gil Collado 1929: 405

Gil Collado 1929a; Claußen 1989b, **MA**, Claußen and Hauser 1990, **MA**, Ifrane (1750 m); Dirickx 1994; Kassebeer 1998c, **HA**; Speight 2013, 2018; Sahib et al. 2020

﻿*Cheilosia ﻿﻿﻿parva* Kassebeer, 1998

Kassebeer 1998c, **MA**, Azrou, Ifrane (1650 m); Claußen and Speight 2007; Sahib et al. 2020

﻿*Cheilosia ﻿rodgersi* Wainwright, 1911

Becker and Stein 1913, **Rif**, Tanger; Claußen 1989a; Dirickx 1994; Kassebeer 1998c, **Rif**, Tanger; Speight 2013, 2018; Sahib et al. 2020

﻿*Cheilosia ﻿﻿﻿scutellata* (Fallén, 1817)

Gil Collado 1929a, **Rif**; Claußen 1989b; Dirickx 1994; Kassebeer 1998c, **Rif**, Chefchaouen, **MA**, Ouiouane, Ifrane; Sahib et al. 2020

﻿*Cheilosia ﻿﻿﻿soror* (Zetterstedt, 1843)

= ﻿*Cheilosia ﻿﻿﻿rufipes* (Preyssler, 1793), in Claussen and Hauser 1990: 436, Kassebeer 1998c: 65

Claußen and Hauser 1990, **MA**, Ifrane (1750 m); Kassebeer 1998c, **MA**, Ifrane; Dirickx 1994; Sahib et al. 2020

﻿*Cheilosia ﻿﻿﻿variabilis* (Panzer, 1798)

Kassebeer 1998c, **MA**, Ifrane; Speight 2013, 2018; Khaganinia and Kazerani 2014; Sahib et al. 2020

﻿***Ferdinandea* Rondani, 1844**

﻿*Ferdinandea ﻿﻿﻿fumipennis* Kassebeer, 1999

Kassebeer 1999b, **MA**, Ifrane, Azrou, **HA**, Marrakech, Ouirgane; Speight 2013, 2018; Sahib et al. 2020

##### Volucellini

﻿***Volucella* Geoffroy, 1762**

﻿*Volucella ﻿﻿﻿inanis* (Linnaeus, 1758)

Claußen and Hauser 1990, **MA**, Ifrane (1750 m); Dirickx 1994; Sahib et al. 2020

﻿*Volucella ﻿﻿﻿liquida* Erichson, 1841

Gil Collado 1929a, **Rif**; Séguy 1930a, **AP**, Mogador, **MA**, Azrou, Bekrit; Kanervo 1939, **MA**; Séguy 1953a, **MA**, Ifrane; Timon-David 1951, **MA**, Ifrane, Azrou, **HA**, Aït Mohamed Sgatt; Leclercq 1961a, **Rif**, Azib de Ketama, **MA**, Dayat Aoua, Ifrane, Azrou; Claußen 1989b; Claußen and Hauser 1990, **MA**, Ifrane (1750 m); Dirickx 1994; Mouna 1998; Sahib et al. 2020, **Rif**, jumb Kitane – MISR

﻿*Volucella ﻿﻿﻿zonaria* Poda, 1761

Séguy 1930a, **AP**, Casablanca; Claußen 1989b; Dirickx 1994; Mouna 1998; Sahib et al. 2020; **AP** (Rabat, Casablanca) – MISR

﻿***Brachypalpus* Macquart, 1834**

﻿*Brachypalpus ﻿﻿﻿valgus* (Panzer, 1798)

Kassebeer 1998a, **MA**, Ifrane, **HA**, Ouirgane; Sahib et al. 2020

##### Psilotini

﻿***Psilota* Fallén, 1823**

﻿*Psilota ﻿﻿﻿atra* (Fallén, 1817)

= ﻿*Psilota ﻿﻿﻿toubkalana* Kassebeer, 1995, in Kassebeer 1995: 395–400

Kassebeer 1995, **HA**; Smit and Vujic 2008, **HA**, Ouirgane, Marrakech; Speight 2018; Sahib et al. 2020, **MA**, Douar Zaouiat Cheikh

##### Pipizinae

Pipizini

﻿***Heringia* Rondani, 1856**

﻿*Heringia ﻿﻿﻿heringi* (Zetterstedt, 1843)

Kassebeer 1998a, **HA**, Tahanaout, Ouirgane; Sahib et al. 2020

﻿***Pipizella* Rondani, 1856**

﻿*Pipizella ﻿thapsiana* Kassebeer, 1995

Kassebeer 1995, **HA** (1000 m); Speight 2013, 2018; Sahib et al. 2020, **MA**, Douar Zaouiat Cheikh

﻿***Triglyphus* Loew, 1840**

﻿*Triglyphus ﻿﻿﻿escalerai* Gil Collado, 1929

Gil Collado 1929a, **Rif**, Tanger; Dirickx 1994; Speight 2013; Sahib et al. 2020

##### Syrphinae

Bacchini

﻿***Melanostoma* Schiner, 1860**

﻿*Melanostoma ﻿﻿﻿mellinum* (Linnaeus, 1758)

Becker and Stein 1913, **Rif**, Tanger; Séguy 1934b, **AP**, Korifla; Timon-David 1951, **MA**, Ifrane, Azrou, Aïn Leuh, **HA**, Aït Mizane; Leclercq 1961a, **MA**, Ifrane; Claußen 1989b, **HA**, Ansegmir-Tal W Midelt (1400); Claußen and Hauser 1990, **MA**, Ifrane (1750 m); Mouna 1998; Pârvu and Zaharia 2007; Sahib et al. 2020, **Rif**, Dayat Rahrah, Aïn el Ma Bared, Oued Dardara, Dayat El Ânassar, Dayat Lemtahane, Garden Ksar Al Rimal, tributary Oued Tazarine, Oued Farda, Dayat El Birdiyel, maison forestière, stream at 1 km from Sidi Yahia Aârab, Dayat Amsemlil, **EM**, farm Saf-Saf, **MA**, Oued d’Ifrane, **HA**, Lac Oukaimeden – MISR

﻿*Melanostoma ﻿﻿﻿mundum* Czerny & Strobl, 1909

Dakki 1997; Mouna 1998

﻿*Melanostoma ﻿﻿﻿scalare* (Fabricius, 1794)

Gil Collado 1929a, **Rif**; Claußen 1989b; Dirickx 1994; Sahib et al. 2020, **Rif**, Aïn el Ma Bared, Oued Mezine, Aïn Quanquben, Aïn Takhninjoute, Oued Koub

﻿***Platycheirus* Le Peletier & Serville, 1828**

﻿*Platycheirus ﻿﻿﻿﻿albimanus* (Fabricius, 1781)^39^

Séguy 1930a, **HA**, Tizi-n’Test, Jebel Imdress; Mouna 1998; Kassebeer 1998b

﻿*Platycheirus ﻿﻿﻿ambiguus* (Fallén, 1817)

Kassebeer 1998b, **HA**; Sahib et al. 2020

﻿*Platycheirus ﻿﻿﻿atlasi* Kassebeer, 1998

Kassebeer 1998b, **MA**, Azrou, Ifrane; Sahib et al. 2020

﻿*Platycheirus ﻿﻿﻿fulviventris* (Macquart, 1829)

Ebejer et al. 2019, **AA**, 14 km E of Rich (Errachidia, 1278 m); Sahib et al. 2020

﻿*Platycheirus ﻿﻿﻿manicatus* (Meigen, 1822)

Séguy 1961; Claußen 1989b; Dirickx 1994; Mouna 1998; Kassebeer 1998b, **HA**, Toubkal; Sahib et al. 2020

﻿*Platycheirus ﻿﻿﻿marokkanus* Kassebeer, 1998

Kassebeer 1998b, **MA**, **HA**; Speight 2018; Sahib et al. 2020, **Rif**, Aïn Takhninjoute, **HA**, Douar Akhlij Tnine Ourika

﻿***Xanthandrus* Verrall, 1901**

﻿*Xanthandrus ﻿﻿﻿comtus* (Harris, 1776)

Gil Collado 1929a, **Rif**, Tanger; Claußen 1989b; Dirickx 1994; Sahib et al. 2020

##### Paragini

﻿***Paragus* Latreille, 1804**

﻿*Paragus ﻿﻿﻿﻿﻿﻿albifrons* (Fallén, 1817)

Kanervo 1939; Claußen 1989b; Dirickx 1994; Mouna 1998; Sahib et al. 2020

﻿*Paragus ﻿﻿﻿atlasi* Claußen, 1989

Claußen 1989b, **HA**, Tizi-n’Test (1900 m); Dirickx 1994; Schmid 1995; Speight 2013, 2018; Sahib et al. 2020

﻿*Paragus ﻿﻿﻿bicolor* (Fabricius, 1794)

Becker and Stein 1913, **Rif**, Tanger; Séguy 1930a, **MA**, Tizi-s’Tkrine, forest of Timelilt, Azrou; Kanervo 1939, **AP**, **HA**; Séguy 1949a, **SA**, Guelmim; Timon-David 1951, **MA**, Ifrane; Claußen 1989b; Claußen and Hauser 1990, **HA**, Tizi-n’Test; Dirickx 1994; Dakki 1997; Sahib et al. 2020, **Rif**, Douar Dacheryène, **HA**, vicinity of Asni; **MA** (Fès, Ifrane, Azrou) – MISR

﻿*Paraguscinctus* Schiner & Egger, 1853

Claußen 1989b, **HA**, Tizi-n’Test (1900 m); Dirickx 1994; Speight 2013, 2018; Sahib et al. 2020, **HA**, vicinity of Asni

﻿*Paragus ﻿﻿﻿coadunatus* Rondani, 1847

Claußen and Hauser 1990, **MA**, Ifrane, Hajeb; Dirickx 1994; Speight 2013, 2018; Sahib et al. 2020

﻿*Paragus ﻿﻿﻿flammeus* Goeldlin, 1971

Claußen and Hauser 1990; Dirickx 1994; Speight 2013, 2018; Sahib et al. 2020

﻿*Paragus ﻿﻿﻿haemorrhous* Meigen, 1844

Claußen 1989b, **MA**, Azrou (1700 m); Dirickx 1994; Sahib et al. 2020, **Rif**, Oued Mharhar

*Paragus ﻿﻿﻿hermonensis* Kaplan, 1981

Claußen 1989b, **MA**, Azrou (1700 m); Dirickx 1994; Sahib et al. 2020

﻿*Paragus ﻿﻿﻿quadrifasciatus* Meigen, 1822

= ﻿*Paragus ﻿﻿﻿pulcherrimus* Strobl, in Timon-David 1951: 144

Timon-David 1951, **MA**, Ifrane; Claußen 1989b, **AP**, Kénitra; Claußen and Hauser 1990, **MA**, Ifrane, Hajeb; Dirickx 1994; Mouna 1998; Sahib et al. 2020, **Rif**, Douar Kitane, **AA**, Agadir airport; **MA** (Ifrane) – MISR

﻿*Paragus ﻿﻿﻿majoranae* Rondani, 1857

Claußen and Hauser 1990, **MA**; Dirickx 1994; Sahib et al. 2020

﻿*Paragus ﻿﻿﻿pecchiolii* Rondani, 1857

Claußen and Hauser 1990, **MA**, Ifrane (1750 m), Hajeb

﻿*Paragus ﻿﻿﻿strigatus* Meigen, 1822

= ﻿*Paragus ﻿﻿﻿bimaculatus* Meigen, in Wiedemann 1824: 33

Wiedemann 1824, **AP**; Claußen 1989b; Claußen and Hauser 1990, **MA**, Ifrane (1750 m), Hajeb; Dirickx 1994; Speight 2018; Sahib et al. 2020

﻿*Paragus ﻿﻿﻿tibialis* (Fallén, 1817)

= ﻿*Paragus ﻿﻿tibialis meridionalis* Becker, in Becker and Stein 1913: 88; Gil Collado 1929: 403; Leclercq 1961: 241

Becker and Stein 1913, **Rif**, Tanger; Gil Collado 1929a; Kanervo 1939; Séguy 1949a, **SA**, Guelmim; Leclercq 1961a, **Rif**, Bab Taza, **MA**, Taza; Claußen 1989b; Claußen and Hauser 1990, **HA**, Tizi-n’Test (2000 m); Dirickx 1994; Dakki 1997; Grabener 2017; Sahib et al. 2020, **HA**, vicinity of Asni, Ijoukak vicinity; **AP** (Rabat) – MISR

﻿*Paragus ﻿﻿﻿vandergooti* Marcos-Garcia, 1986

Claußen 1989b, **HA**, Tizi-n’Test à (1900 m); Claußen and Hauser 1990, **MA**, Ifrane, Hajeb; Dirickx 1994; Speight 2013, 2018; Sahib et al. 2020

##### Syrphini

﻿***Chrysotoxum* Meigen, 1803**

﻿*Chrysotoxum ﻿﻿﻿bicinctum* (Linnaeus, 1758)

Timon-David 1951, **MA**, Ifrane, **HA**, Haute Réghaya; Leclercq 1961a, **MA**, Mischliffen (2019 m); Claußen 1989b; Dirickx 1994; Mouna 1998; Sahib et al. 2020; **MA**, **HA** – MISR

﻿*Chrysotoxum ﻿﻿﻿intermedium* Meigen, 1822

Becker and Stein 1913, **Rif**, Tanger; Gil Collado 1929a; Séguy 1930a, **MA**, Tizi-S’Tkrine, Aïn Leuh, forest of Taffert, **HA**, Tizi-n’Test, Goundafa; Kanervo 1939; Claußen 1989b; Claußen and Hauser 1990, **MA**, Ifrane (1750 m); Dirickx 1994; Mouna 1998; Pârvu et al. 2006, **AP**, Cap Bedouza; Dousti and Hayat 2006; Pârvu and Zaharia 2007; Kazerani et al. 2013b; Sahib et al. 2020, **Rif**, 1 km after Dardara, Oued Azila, Dayat Jebel Zemzem, Aïn El Maounzil, Oued Tafoughalt, **MA**, Douar Zaouiat Cheikh, **HA**, vicinity of Asni, **AA** Douar Issafen; **AP** (Rabat), **MA** (Aïn Leuh), **HA** (Réghaya) – MISR

﻿*Chrysotoxum ﻿﻿﻿volaticum* Séguy, 1961

Séguy 1961, **MA**; Claußen 1989b, **HA**, Oukaimeden (2600 m); Claußen and Hauser 1990, **MA**, Ifrane (1750 m); Dirickx 1994; Sahib et al. 2020

﻿***Dasysyrphus* Enderlein, 1938**

﻿*Dasysyrphus ﻿﻿﻿albostriatus* (Fallén, 1817)

Kassebeer 1998a, **HA**, Bin-el-Ouidane, Ouirgane, Imlil, Asni; Sahib et al. 2020

﻿***Epistrophe* Walker, 1852**

﻿*Epistrophe ﻿﻿﻿eligans* (Harris, 1780)

= ﻿*Syrphus ﻿﻿﻿ochrostoma* (Zetterstedt), in Becker and Stein 1913: 88; Claußen 1989b: 372

Becker and Stein 1913, **Rif**, Tanger; Claußen 1989b; Kassebeer 1998a, **MA**, Ifrane, **HA**, Imlil, Asni, Ouirgane; Sahib et al. 2020, **Rif**, Dayat Tazia

﻿*Epistrophe ﻿﻿﻿eligans* (Harris, 1870) var. ﻿*trifasciata* Strobl

Ebejer and Bensusan 2010, **AA**; Djellab et al. 2013

﻿***Episyrphus* Matsumura & Adachi, 1917**

﻿*Episyrphus ﻿﻿﻿balteatus* (De Geer, 1776)

= ﻿*Syrphus ﻿﻿﻿balteatus* De Geer, in Becker and Stein 1913: 88; Séguy 1930a: 129

= ﻿*Epistrophe ﻿﻿﻿balteata* (De Geer), in Gil Collado 1929: 406; Kanervo 1939: 3; Timon-David 1951: 144

Becker and Stein 1913, **Rif**, Tanger; Gil Collado 1929a; Séguy 1930a, **AP**, Rabat, **MA**, Aïn Sferguila; Timon-David 1951, **AP**, forest of Maâmora, Rabat, **HA**, Marrakech; Claußen 1989b; Dirickx 1994; Mouna 1998; Sahib et al. 2020, **Rif**, Sebt Zinate, Aïn Sidi Brahim Ben Arrif, dam Nakhla, Aïn Boughaba, Garden Ksar Al Rimal, Oued Aârkoub, Dayat Rahrah, Oued Sahel, dam Moulay Bouchta, maison forestière, Ksar El Kébir, Dayat Jebel Zemzem, Oued Taida, stream at 1 km from Sidi Yahia Aârab, Aïn Quanquben, Oued Maggou, Forest Bab El Karn, Douar Kitane, forest El Mahfoura, **HA**, Aïn Zarka of Meski, **AA**, Douar Zaouia; **AP** (Rabat) – MISR

﻿***Eupeodes* Osten-Sacken, 1877**

﻿*Eupeodes ﻿﻿﻿corollae* (Fabricius, 1794)

= ﻿*Syrphus ﻿﻿﻿berber* Bigot, in Bigot 1884: 88

= ﻿*Syrphus ﻿﻿﻿corollae* Meigen, in Becker and Stein 1913: 88; Séguy 1930a: 129, Kanervo 1939: 3; Gil Collado 1929: 406

= ﻿*Syrphus ﻿﻿﻿corollae* Fabricius, in Timon-David 1951: 144

= *Metasyrphus ﻿﻿﻿corollae* (Fabricius), in Dirickx 1994: 89

Bigot 1884; Becker and Stein 1913, **Rif**, Tanger; Gil Collado 1929a; Séguy 1930a, **HA**, Tizi-n’Test, Jebel Imdress, Goundafa; Kanervo 1939; Timon-David 1951, **AP**, forest of Maâmora, Rabat, Sidi Taibi, **HA**, Réghaya, Tazzarine, **AA**, Agdz, Plaine de Souss (Taroudant); Leclercq 1961a, **MA**, Ifrane; Claußen and Hauser 1990, **MA**, Ifrane (1750 m), **HA**, Oukaimeden (3200 m), **AA**, Tan-Tan; Dirickx 1994; Mouna 1998; Pârvu and Zaharia 2007; Grabener 2017; El Hawagry and Gilbert 2019; Sahib et al. 2020, **Rif**, Village Sebt Zinate, Aïn el Ma Bared, Garden Ksar Al Rimal, Oued Bin EL Ouidane, Oued Sahel, Aïn Takhninjoute, stream at 1 km from Sidi Yahia Aârab, Oued Jnane Niche, dam Smir, Oued Boumarouil, Dayat Jebel Zemzem, Oued Maggou, Meadow Fahs Lmhar, Douar Kitane, forest El Mahfoura, **MA**, Douar Zaouiat Cheikh, **HA**, vicinity of Asni, Ijoukak vicinity, Lac Oukaimeden; **AA** Agdz – MISR

﻿*Eupeodes ﻿﻿﻿latifasciatus* (Macquart, 1829)

= ﻿*Syrphus ﻿﻿﻿latifasciatus* Macquart, in Séguy 1949: 156; Séguy 1953a: 84

= *Metasyrphus ﻿﻿﻿latifasciatus* (Macquart), in Dirickx 1994: 89

Séguy 1949a, **SA**, Guelmim; Séguy 1953a, **AA**, Oued Khoref; Claußen 1989b; Dirickx 1994; Dakki 1997; Sahib et al. 2020, **Rif**, Douar Kitane, maison forestière, Oued Ametrasse

﻿*Eupeodes ﻿﻿﻿luniger* (Meigen, 1822)

= *Metasyrphus ﻿﻿﻿luniger* (Meigen), in Dirickx 1994: 90, 237

= ﻿*Syrphus ﻿﻿﻿luniger* Meigen, in Gil Collado 1929: 406

Gil Collado 1929a, **AP**; Claussen 1989b; Dirickx 1994; Sahib et al. 2020, **Rif**, Oued Maggou, Oued Martil, Belyounech, Douar Kitane, **MA**, Douar Ben Smim, **HA**, vicinity of Asni

﻿*Eupeodes ﻿﻿﻿nuba* (Wiedemann, 1830)

= ﻿*Syrphus ﻿﻿﻿rufinasutus* Bigot, in Bigot 1884: 88, Séguy 1961: 107

= *Metasyrphus ﻿﻿﻿nuba* (Wiedemann), in Dirickx 1994: 90, 238

Bigot 1884; Séguy 1961; Claußen 1989b, **HA**, Ansegmir-Tal W Midelt (1400 m); Dirickx 1994; Dousti and Hayat 2006; Ehteshamnia et al. 2010; Naderloo et al. 2011; Speight 2013, 2018; Kazerani et al. 2013b; El Hawagry and Gilbert 2019; Sahib et al. 2020

﻿*Eupeodes ﻿﻿﻿punctifer* (Frey, 1934)^40^

Mouna 1998: 86

﻿***Ischiodon* Sack, 1913**

﻿*Ischiodon ﻿﻿﻿aegyptius* (Wiedemann, 1830)

= *Simosyrphus ﻿﻿﻿aegyptius* (Wiedemann, 1830)

Gil Collado 1929a; Timon-David 1951, **AP**, Rabat, **AA**, Agdz; Mouna 1998; Grabener 2017; Mengual 2018; Sahib et al. 2020; **AP** (Rabat) – MISR

﻿***Lapposyrphus* Dušek & Láska, 1967**

﻿*Lapposyrphus ﻿﻿﻿lapponicus* (Zetterstedt, 1838)

= ﻿*Syrphus ﻿﻿﻿arcuatus* Fallén, 1817, in Becker and Stein 1913: 88

= *Metasyrphus ﻿﻿﻿lapponicus* (Zetterstedt, 1838), in Dirickx 1994: 89

= ﻿*Eupeodes ﻿﻿﻿lapponicus* (Zetterstedt, 1838), in Claußen 1989b: 372

Becker and Stein 1913, **Rif**; Claußen 1989b; Dirickx 1994; Sahib et al. 2020

﻿***Meliscaeva* Frey, 1946**

﻿*Meliscaeva ﻿﻿﻿auricollis* (Meigen, 1822)

= ﻿*Epistrophe ﻿﻿﻿auricollis* Meigen, in Becker and Stein 1913: 89; Gil Collado 1929: 406; Timon-David 1951: 145

Becker and Stein 1913, **Rif**, Tanger; Gil Collado 1929a; Timon-David 1951, **AP**, Oued Korifla, Zaers, Rabat; Claußen 1989b; Dirickx 1994; Mouna 1998; El Hawagry and Gilbert 2019; Sahib et al. 2020, **Rif**, Aïn el Ma Bared, Dayat El Ânassar, Belyounech, Oued Mezine, dam Moulay Bouchta, Aïn Afersiw, dam Entrasol, Oued à 15 km de Fifi, jumb Kitane, Oued Maggou, Douar Kitane, Oued Sahel, **MA**, Aïn Ouilili; **AP** (Rabat, Zaers) – MISR

﻿*Meliscaeva ﻿﻿﻿cinctella* (Zetterstedt, 1843)

= ﻿*Syrphus ﻿﻿﻿cinctellus* Zeterstedt, in Séguy 1934b: 162

Séguy 1934b, **MA**, Oued Leben (Taounate); Claußen 1989b; Dirickx 1994; Mouna 1998; Sahib et al. 2020 – MISR

﻿***Scaeva* Fabricius, 1850**

﻿*Scaeva ﻿﻿﻿﻿albomaculata* (Macquart, 1842)

= *Lasiopticus ﻿﻿﻿﻿albomaculata* (Macquart), in Gil Collado 1929: 405; Timon-David 1951: 145

= *Lasiophthicus ﻿﻿﻿albomaculatus* Macquart, in Séguy 1953a: 84

Gil Collado 1929a; Séguy 1953a, **MA**, Immouzer; Timon-David 1951, **AP**, Rabat, **MA**, El Harcha; Leclercq 1961a, **MA**, Azrou; Claußen 1989b, **MA**, Azrou (1700 m); Claußen and Hauser 1990, **MA**, Ifrane; Dirickx 1994; Mouna 1998; Dousti and Hayat 2006; Ehteshamnia et al. 2010; Naderloo et al. 2011; Speight 2013, 2018; Kazerani et al. 2013b; Grabener 2017; El Hawagry and Gilbert 2019; Sahib et al. 2020, **Rif**, Belyounech, **HA**, Aïn Zarka of Meski, Lac Oukaimeden; **AP** (Rabat, Cap Cantin), **EM** (Debdou) – MISR

﻿*Scaeva ﻿﻿﻿dignota* (Rondani, 1857)

Claußen and Hauser 1990, **MA**, Ifrane (1750 m); Dirickx 1994; Sahib et al. 2020, **Rif**, Oued Maggou, Dayat Lemtahane

﻿*Scaeva ﻿﻿﻿mecogramma* (Bigot, 1860)

Dirickx 1994; Kassebeer 1998a, **Rif**, Chefchaouen, **AP**, Kénitra, **HA**, Ouirgane; Sahib et al. 2020

﻿*Scaeva ﻿﻿﻿pyrastri* (Linnaeus, 1758)

= *Catabomba ﻿﻿﻿pyrastri* Linnaeus, in Becker and Stein 1913: 88

= *Lasiophthicus ﻿﻿﻿pyrastri* Linnaeus, in Séguy 1930a: 128

= *Lasiopticus ﻿﻿﻿pyrastri* Linnaeus, in Gil Collado 1929: 405, Timon-David 1951: 145

Becker and Stein 1913, **Rif**; Gil Collado 1929a, **AP**; Séguy 1930a, **AP**, forest of Zaers, forest of Maâmora, **MA**, Tizi-s’Tkrine; Timon-David 1951, **AP**, Rabat, **MA**, Ifrane; Claußen 1989b; Dirickx 1994; Mouna 1998; Sahib et al. 2020, **Rif**, Dayat Jebel Zemzem, stream at 1 km from Oued Sidi Yahia Aârab, **AA**, 1 km before Douar Aïn Lahmar; **AP** (Rabat, Cap Cantin) – MISR

﻿*Scaeva ﻿﻿﻿selenitica* (Meigen, 1822)

= *Lasiophthicus ﻿﻿﻿seleniticus* Meigen, in Séguy 1930a: 128

Séguy 1930a, **HA**, Aguerd el Had, **AA**, Talekjount (Souss); Claußen 1989b; Dirickx 1994; Mouna 1998; Sahib et al. 2020

﻿***Sphaerophoria* Le Peletier & Serville, 1828**

﻿*Sphaerophoria ﻿﻿﻿interrupta* (Fabricius, 1805)

= ﻿*Sphaerophoria ﻿﻿﻿menthastri* (Linnaeus), in Becker and Stein 1913: 87; Kanervo 1939: 3; Timon-David 1951: 145; Séguy 1961: 109

Kanervo 1939, **AP**, **MA**, **HA**; Becker and Stein 1913, **Rif**; Timon-David 1951, **AP**, Kénitra, Rabat, **MA**, Harcha, Ifrane, Sefrou, **HA**, Agdz; Claußen 1989b; Dirickx 1994; Mouna 1998; El Hawagry and Gilbert 2019; Sahib et al. 2020; **AP** (Rabat), **MA** (Ifrane, Meknès) – MISR

﻿*Sphaerophoria ﻿﻿﻿rueppelli* (Wiedemann, 1830)

Kanervo 1939, **Rif**, **HA**; Timon-David 1951, **AP**, Rabat, **AA**, Agadir, Agdz, Zagora; Séguy 1961; Claußen 1989b, **HA**, Ansegmir-Tal W Midelt (1400 m); Dirickx 1994; Mouna 1998; El Hawagry and Gilbert 2019; Sahib et al. 2020, **Rif**, Tarmast tributary, Oued Sidi Ben Saâda, Dayat Amsemlil, **EM**, farm Saf-Saf, **MA**, Oued d’Ifrane; **AP** (Rabat), **AA** (Agadir, Agdz, Zagora) – MISR

﻿*Sphaerophoria ﻿﻿﻿scripta* (Linnaeus, 1758)

= ﻿*Sphaerophoriadispar* (Meigen), in Timon-David 1951: 145

Becker and Stein 1913, **Rif**, Tanger; Gil Collado 1929a, **Rif**; Séguy 1930a, **AP**, Tlet n’Rhohr, **MA**, forest of Timelilt, Aïn Leuh, **EM**, Berkane; Kanervo 1939, **Rif**, **AP**, **MA**, **HA**; Séguy 1941a, **HA**, Tachdirt (Toubkal, 2500 m); Timon-David 1951, **AP**, Rabat, **MA**, Harcha, Sefrou, Ifrane; Leclercq 1961a, **MA**, Dayat Aoua, Ifrane, Azrou; Claußen 1989b, **MA**, Azrou (1700 m), **HA**, Oukaimeden, Tizi-n’Test (1900 m), Ansegmir-Tal W midelt (1400 m); Claußen and Hauser 1990, **MA**, Ifrane, Hajeb (1750 m), **HA**, Oukaimeden (3200 m); Dirickx 1994; Mouna 1998; Pârvu and Zaharia 2007; El Hawagry and Gilbert 2019; Sahib et al. 2020, **Rif**, Oued Aârkoub, dam Nakhla, meadow Mizoghar, Oued Dardara, 1 km after Dardara, Dayat El Birdiyel, palm grove Igrane, Aïn Quanquben, maison forestière, Oued Sidi Ben Saâda, Dayat Lemtahane, Dayat Amsemlil, forest El Mahfoura, **EM**, farm Saf-Saf, **MA**, Oued d’Ifrane, **HA**, vicinity of Asni, Lac Oukaimeden, **AA**, Msidira – MISR

﻿*Sphaerophoria ﻿﻿﻿taeniata* (Meigen, 1822)

= ﻿*Sphaerophoria﻿menthastrivar.﻿taeniata* Meigen, in Timon-David 1951: 10

Timon-David 1951, **AP**, Rabat, saline mud; Claußen 1989b; Dirickx 1994; Sahib et al. 2020; **MA** (Aïn Leuh, Azrou, Timahdit) – MISR

﻿***Syrphus* Fabricius, 1775**

﻿*Syrphus ﻿﻿﻿ribesii* (Linnaeus, 1758)

Kassebeer 1998a, **Rif**, Chefchaouen, Tétouan; Sahib et al. 2020, **Rif**, Douar Kitane

﻿*Syrphusvitripennis* Meigen, 1822

Gil Collado 1929a, **Rif**, Tanger; Claußen 1989b; Dirickx 1994; Sahib et al. 2020

﻿***Xanthogramma* Schiner, 1860**

﻿*Xanthogramma ﻿﻿﻿dives* (Rondani, 1857)

Ebejer et al. 2019, **AA**, 29 km N of Rich (Errachidia, 1570 m); Sahib et al. 2020

﻿*Xanthogramma ﻿﻿﻿evanescens* Becker, 1913

Becker and Stein 1913, **Rif**; Claußen 1989b; Dirickx 1994; Sahib et al. 2020

﻿*Xanthogramma ﻿﻿﻿marginale* (Loew, 1854)

= ﻿*Xanthogramma﻿marginalevar.﻿morenae* Loew, in Becker and Stein 1913: 86, Gil Collado 1929: 406

Becker and Stein 1913, **Rif**, Tanger; Gil Collado 1929a, **Rif**; Kanervo 1939, **Rif**, **HA**; Séguy 1961; Claußen and Hauser 1990, **MA**, Ifrane (1750 m); Mouna 1998; Claußen 1989b; Dirickx 1994; Ebejer and Bensusan 2010, **AA**; Speight 2013, 2018; Sahib et al. 2020, **Rif**, village Sebt Zinate, Oued Maggou, Oued Ametrasse, Douar Kitane

﻿*Xanthogramma ﻿﻿﻿pedissequum* (Harris, 1776)

= ﻿*Xanthogramma ﻿﻿﻿ornatum* (Meigen, 1822), in Gil Collado 1929: 406

Gil Collado 1929a, **Rif**, Tanger; Claußen 1989b; Dirickx 1994; Sahib et al. 2020

### ﻿Conopoidea

#### ﻿﻿CONOPIDAE^41^

K. Kettani

Number of species: **34**. Expected: 40

Faunistic knowledge of the family in Morocco: good

##### Conopinae

Conopini

﻿***Conops* Linnaeus, 1758**

﻿*Conops ﻿﻿﻿﻿aegyptiacus* (Rondani, 1850)

Kröber 1915, 1928

﻿*Conops ﻿﻿﻿ceriaeformis* Meigen, 1804

= ﻿*Conops ﻿﻿﻿﻿﻿﻿acuticornis* Loew, 1847, in Becker and Stein 1913: 89

Becker and Stein 1913, **Rif**, Tanger; Kröber 1924, 1928, **Rif**, Tanger

﻿*Conops ﻿﻿﻿djanetianus* Séguy, 1938

Mouna 1998: 85

﻿*Conops ﻿﻿﻿elegans* Meigen, 1824

= ﻿*Conops ﻿﻿﻿semifumosus* Adams, in Séguy 1934b: 162

= ﻿*Conops ﻿﻿﻿ruficornis* Becker, 1913, in Becker and Stein 1913: 89; Kröber 1924: 69

Becker and Stein 1913, **Rif**, Tanger; Kröber 1924, 1927, **AP**, Casablanca; Séguy 1934b, **Rif**, Tanger; Stuke 2016; El Hawagry et al. 2021

﻿*Conops ﻿﻿﻿nubeculipennis* Bezzi, 1901

= ﻿*Conops ﻿﻿﻿atrogonius* Séguy, 1930, in Séguy 1930a: 134; Séguy 1953a: 85; Mouna 1998: 85

Séguy 1930a, **AP**, Rabat, Mogador; Séguy 1953a, **MA**, Dayat Ifrah; Mouna 1998; Stuke and Schmid-Egger 2015, **AA**, 1.5 km S of Tissint, 14 km NW of Icht; Stuke 2016; El Hawagry et al. 2021

﻿*Conops ﻿﻿﻿theryi* Séguy, 1928

Séguy 1928d; Séguy 1930a, **AP**, Rabat, Casablanca; Mouna 1998

﻿*Conops ﻿﻿﻿tifedarius* Séguy, 1928

Séguy 1928d; Séguy 1930a, **AP**, Rabat; Mouna 1998

﻿***Leopoldius* Rondani, 1843**

﻿*Leopoldius ﻿﻿﻿coronatus* (Rondani, 1857)

= *Brachyglossum ﻿﻿﻿coronatum* Rondani, in Séguy 1930a: 132; Mouna 1998: 85

Séguy 1930a, **MA**, Aïn Leuh (1400–1500 m); Mouna 1998

##### Physocephalini

﻿***Physocephala* Schiner, 1861**

﻿*Physocephala ﻿﻿﻿chrysorrhoea* (Meigen, 1824)

Séguy 1930a, **AP**, Sidi Bettache; Mouna 1998; El Hawagry et al. 2021

﻿*Physocephala ﻿﻿﻿laticincta* (Brullé, 1832)

Séguy 1930a, **MA**, Aïn Leuh (1400–1500 m); Bei-Bienko and Steyskal 1989; Mouna 1998

﻿*Physocephala ﻿﻿﻿maculigera* Kröber, 1915

Séguy 1941d, **AA**, Agadir; Mouna 1998

﻿*Physocephala ﻿﻿﻿nigra* (De Geer, 1776)

Séguy 1930a, **AP**, Sidi Bettache

﻿*Physocephala ﻿﻿﻿pusilla* (Meigen, 1804)

Séguy 1928d; Séguy 1930a, **MA**, Meknès, Ras el Ksar (1900 m), **HA**, Asni; Mouna 1998; Stuke and Schmid-Egger 2015, **AA**, Aoulouz, 2 km NW of Tissint; El Hawagry et al. 2021

﻿*Physocephala ﻿﻿﻿rufipes* (Fabricius, 1781)

Mouna 1998; **AP** (Tagulet (Essaouira)) – MISR

﻿*Physocephala ﻿﻿﻿vittata* (Fabricius, 1794)

Séguy 1928d; Séguy 1930a, **AP**, Rabat, Casablanca, **MA**, Meknès, Forêt Zaers; Mouna 1998; Stuke and Schmid-Egger 2015, **AA**, Aoersi (15 km NE of Agadir), **AA**, Oued near beach (19 km W of Tiznit); El Hawagry et al. 2021; **HA** – MISR

﻿***Pseudophysocephala* Kröber, 1940**

﻿*Pseudophysocephalabouvieri* (Séguy, 1936)

= ﻿*Conopsbouvieri* Séguy, in Séguy 1936a: 299

Séguy 1936a, **MA**, Meknès (550 m); Sidney 2001, **MA**, Meknès

##### Dalmanninae

Dalmannini

﻿***Dalmannia* Robineau-Desvoidy, 1830**

﻿*Dalmannia ﻿﻿﻿aculeata* (Linnaeus, 1761)

Séguy 1928d; Séguy 1930a, **MA**, Aïn Leuh, Meknès; Mouna 1998 – MISR

﻿*Dalmannia ﻿﻿﻿dorsalis* (Fabricius, 1794)

= ﻿*Dalmanniaflavescens* (Meigen), in Becker and Stein 1913: 90

Becker and Stein 1913, **Rif**, Tanger; Stuke and Kehlmaier 2008, **MA**, Fès

##### Myopinae

Myopini

﻿***Melanosoma* Robineau-Desvoidy, 1853**

﻿*Melanosoma ﻿﻿﻿bicolor* (Meigen, 1824)

Séguy 1941d, **AA**, Agadir (Admine forest); Mouna 1998

﻿*Melanosoma ﻿﻿﻿mundum* Czerny & Strobl, 1909

Becker and Stein 1913, **Rif**, Tanger; Séguy 1930a, **HA**, Tafingoult (Goundafa, 1500–1600 m), **AA**, Tenfeht (Souss, 1000–1500 m); Séguy 1953a, **HA**, Aït Ourir; Séguy 1949a, **AA**, Foum-el-Hassan; Mouna 1998; Stuke and Schmid-Egger 2015, **AA**, SE of Awir (10 km NNW of Agadir), Talmakant (80 km NE of Agadir), **AA**, 19 km W of Tiznit, Massa river (25 km NE of Tiznit), Imitek (30 km WSW of Tata), Issafen (55 km WNW of Tata); Grabener 2017

﻿***Myopa* Camras, 1953**

﻿*Myopa ﻿﻿﻿buccata* (Linnaeus, 1758)

Mouna 1998; **MA** (Oulmès) – MISR

﻿*Myopa ﻿﻿﻿dorsalis* Fabricius, 1794

Séguy 1930a, **MA**, Jebel Ahmar (1750 m); Mouna 1998; **MA** (Oulmès); El Hawagry et al. 2021 – MISR

﻿*Myopa ﻿﻿﻿hirsuta* Stuke & Clements, 2008

Stuke and Clements 2008, **MA**, Azrou, Ifrane

﻿*Myopa ﻿﻿﻿nigrita* Wiedemann, 1824

Wiedemann 1824, 1830; Kröber 1916, 1928

﻿*Myopa ﻿﻿﻿pellucida* Robineau-Desvoidy, 1830

Stuke and Clements 2008, **Rif**, Chefchaouen, **HA**, Ourika

﻿*Myopa ﻿﻿﻿picta* Panzer, 1798

Séguy 1930a, **AP**, Casablanca, **MA**, Meknès; Mouna 1998; El Hawagry et al. 2021

﻿*Myopa ﻿﻿﻿stigma* Meigen, 1824

Becker and Stein 1913, **Rif**, Tanger; El Hawagry et al. 2021

﻿*Myopa ﻿testacea* (Linnaeus, 1767)

Séguy 1930a, **AP**, Casablanca, Fouarat, **HA**, Arround (Skoutana: 2000–2400 m), Tachdirt, Jebel Likount; Mouna 1998; **AP** (Fouarat) – MISR

﻿*Myopa ﻿﻿﻿palliceps* (Bigot, 1887)

= ﻿*Myopaminor* Strobl, in Clements 2000: 239

= ﻿*Myopa ﻿vaulogeri* Séguy, in Séguy 1930a: 136; Clements 2000: 236

Séguy 1930a, **AP**, Casablanca; Villeneuve 1933; Mouna 1998; Clements 2000, **AP**, Casablanca, **HA**, Marrakech, Ourigane (1000 m), **AA**, Ammelental (10 km NE of Tafraoute)

﻿***Thecophora* Rondani, 1845**

﻿*Thecophora ﻿﻿﻿atra* (Fabricius, 1775)

= *Occemyia ﻿﻿﻿atra* Fabricius, in Séguy 1930a: 137; Becker and Stein 1913: 90

Becker and Stein 1913, **Rif**, Tanger; Séguy 1930a, **AP**, Sidi Bettache, **MA**, Meknès, **HA**, Asni, **AA**, Taroudant (Souss); Mouna 1998; **HA** (Mouldikht (Marrakech)); El Hawagry et al. 2021 – MISR

﻿*Thecophora ﻿﻿﻿cinerascens* (Meigen, 1804)

= ﻿*Thecophora ﻿﻿﻿pusilla* (Meigen, 1824), in Pârvu et al. 2006: 276; Popescu-Mirceni 2011: 35

Pârvu et al. 2006, **MA**, Ifrane; Popescu-Mirceni 2011, **MA**, Ifrane

##### Sicini

﻿***Sicus* Scopoli, 1763**

﻿*Sicus ﻿﻿﻿ferrugineus* (Linnaeus, 1761)

Séguy 1930a, **MA**, Aïn Leuh; Zimina 1975; Mouna 1998; **MA** (Tizi-n’Ifrah, Guisser (1400 m)) – MISR

##### Zodioninae

Zodionini

﻿***Zodion* Latreille, 1797**

﻿*Zodion ﻿﻿﻿cinereum* (Fabricius, 1794)

Séguy 1930a, **MA**, Meknès, **HA**, Around (Skoutana); Mouna 1998; Grabener 2017

﻿*Zodion ﻿﻿﻿erythrurum* Rondani, 1865

Séguy 1930a, **AP**, Casablanca, **HA**, Dar Caïd M’Tougui; Mouna 1998; Stuke and Schmid-Egger 2015, **AA**, Tamzergoute (10 km N of Agadir); El Hawagry et al. 2021

### ﻿Nerioidea

#### ﻿﻿MICROPEZIDAE

K. Kettani, M.J. Ebejer

Number of species: **1**. Expected: 2

Faunistic knowledge of the family in Morocco: poor

﻿***Micropeza* Meigen, 1803**

﻿*Micropeza ﻿﻿﻿kettaniae* Ebejer, 2019

Ebejer 2019b, **Rif**, Oued Kbir (Béni Ratene, 157 m), Dayat Tazia (Tazia, 733 m) – MISR, NHMUK

### ﻿Tanypezoidea

#### ﻿﻿PSILIDAE

K. Kettani, M.J. Ebejer

Number of species: **1**. Expected: 5

Faunistic knowledge of the family in Morocco: poor

﻿***Chamaepsila* Hendel, 1917**

﻿*Chamaepsila ﻿﻿﻿nigricornis* (Meigen, 1826)

Ebejer et al. 2019, **Rif**, Tétouan, Onsar Lile (349 m), Aïn Tissemlal (Azilane, 1255 m), **MA**, 17 km SW of Midelt (1940 m), Lac Aguelmane Afennourir (30 km SW of Azrou, 2050 m)

### ﻿Tephritoidea

#### ﻿﻿LONCHAEIDAE

K. Kettani, I. MacGowan

Number of species: **5**. Expected: 30

Faunistic knowledge of the family in Morocco: poor

##### Dasiopinae

﻿***Dasiops* Rondani, 1856**

﻿*Dasiops ﻿﻿﻿latifrons* (Meigen, 1826)

Séguy 1934a; Mouna 1998; MacGowan and Freidberg 2008; **AP** (Rabat) – MISR; **Rif** (Tanger), **AP** (Rabat) – MHNP

##### Lonchaeinae

﻿***Lamprolonchaea* Bezzi, 1920**

﻿*Lamprolonchaea ﻿﻿﻿smaragdi* (Walker, 1849)

= *Lonchea ﻿﻿﻿aurea* Macquart, 1851, in Séguy 1953a: 85

Becker and Stein 1913, **Rif**, Tanger; Séguy 1934a; Séguy 1953a, **AP**, Rabat, **MA**, Fès; Rungs 1952, **HA**, Arganier; Mouna 1998

﻿***Lonchaea* Fallén, 1820**

﻿*Lonchaea ﻿﻿﻿tarsata* Fallén, 1820

MacGowan and Freidberg 2008

﻿*Lonchaea* ﻿sp.

= Recorded as ﻿*Lonchaea ﻿﻿﻿laticornis* Meigen, 1826 but almost certainly not this species

Becker and Stein 1913, **Rif**, Tanger

﻿***Silba* Macquart, 1851**

﻿*Silba ﻿﻿﻿adipata* McAlpine, 1956

= ﻿*Lonchaea ﻿﻿﻿aristella* Becker, 1903, in Séguy 1934b: 162

Séguy 1934b, **AP**, Rabat; MacGowan and Freidberg 2008; **AP** (Rabat) – MNHN

#### ﻿﻿PALLOPTERIDAE

K. Kettani, M.J. Ebejer

Number of species: **1**. Expected: 3

Faunistic knowledge of the family in Morocco: poor

﻿***Palloptera* Fallén, 1820**

﻿*Palloptera ﻿﻿﻿ustulata* (Fallén, 1820)

Ebejer et al. 2019, **MA**, Zaouia d’Ifrane (Ifrane, 1603 m)

#### ﻿﻿PIOPHILIDAE

K. Kettani, M.J. Ebejer

Number of species: **3**. Expected: 6

Faunistic knowledge of the family in Morocco: poor

##### Piophilinae

﻿***Mycetaulus* Loew, 1845**

﻿*Mycetaulus ﻿﻿﻿hispanicus* Duda, 1927

Mouna 1998; Carles-Tolrá 2002

﻿***Piophila* Fallén, 1810**

﻿*Piophila ﻿﻿﻿casei* (Linnaeus, 1758)

Séguy 1930a, **Rif**, Tanger, **AP**, Rabat, **MA**, Meknès; Mouna 1998; **Rif** (Tanger), **AP** (Rabat), **MA** (Meknès) – MISR

﻿***Prochyliza* Walker, 1849**

﻿*Prochyliza ﻿﻿﻿nigrimana* (Meigen 1826)

Ebejer et al. 2019, **Rif**, Aïn Tissemlal (Azilane, 1255 m)

#### ﻿﻿PLATYSTOMATIDAE

K. Kettani, G.V. Popov

Number of species: **4**. Expected: 4

Faunistic knowledge of the family in Morocco: moderate

##### Platystomatinae

﻿***Platystoma* Meigen, 1803**

﻿*Platystoma ﻿﻿﻿idia* Séguy, 1934^42^

Séguy 1934a, **AP**, Aïn Sferguila (Forêt Zaers); Hennig 1945, **AP**, Aïn Sferguila (Forêt Zaers); Soós 1984a

﻿*Platystoma ﻿﻿﻿meridionale* Hendel, 1913

= ﻿*Platystoma ﻿﻿﻿seminationis* (Fabricius), in Becker 1907: 385

Becker 1907; Hendel 1913, **AP**, Mogador; Hennig 1945, **AP**, Mogador; Soós 1984a, **AP**, Mogador

﻿***Rivellia* Robineau-Desvoidy, 1830**

﻿*Rivellia ﻿﻿﻿hispanica* Lyneborg, 1969

Ebejer et al. 2019, **EM**, Tafoughalt

﻿*Rivelliasyngenesiae* (Fabricius, 1781)

Becker and Stein 1913, **Rif**, Tanger; Mouna 1998; **MA** (Oulmès, Meknès) – MISR

#### ﻿﻿TEPHRITIDAE

K. Kettani, A.L. Norrbom

Number of species: **69**. Expected: 75

Faunistic knowledge of the family in Morocco: moderate

##### Dacinae

Ceratitidini

﻿***Capparimyia* Bezzi, 1920**

﻿*Capparimyiasavastanii* (Martelli, 1911)

= ﻿*Capparimyiasavastanii* (Martelli), in Séguy 1953a: 85

Séguy 1953a, **AA**, Tiznit (on *Capparisspinosa*); El Harym and Belqat 2017

﻿***Ceratitis* MacLeay, 1829**

﻿*Ceratitis ﻿capitata* (Wiedemann, 1824)

Becker and Stein 1913, **Rif**, Tanger; Vayssière 1920, **AP**, Rabat; Séguy 1930a (common in all of Morocco); Rungs 1952, **HA**, Arganier; Harris et al. 1980; Mouna 1998; De Meyer 2000, **AP**, Ouadj-Ouli-Mohamed, env. Settat, Insgane; Vidal et al. 2008; Aboussaid et al. 2009; Koçak and Kemal 2010; El Harym and Belqat 2017, **Rif**, Kitane, El Haouta, **MA**, Sensla, **AA**, Environs Massa, Oued Massa, Douar Sidi Abou, Douar Tighrimt, Douar Zaouia; Elaini and Mazih 2018; Elaini et al. 2019, **HA**, Arganeraie; **AP** (Safi) – MHNNR

##### Dacini

﻿***Bactrocera* Macquart, 1835**

﻿*Bactrocera ﻿﻿﻿oleae* (Rossi, 1790)

= ﻿*Dacus ﻿﻿﻿oleae* Rossi, in Becker and Stein 1913: 94, Vayssière 1920: 256

= ﻿*Dacus ﻿﻿﻿oleae* Gmelin, in Séguy 1930a: 168

Becker and Stein 1913, **Rif**, Tanger; Vayssière 1920, **EM**, Oujda; Séguy 1930a; Mouna 1998; Savio 2011, **EM**, Oujda; El Harym and Belqat 2017, **Rif**, El Haouta, Oued Maâza, Cascade Chrafate, Koudiat El Aouinate, Lâazaba, Dhar Sbagh Mâasra, El Hajria, **MA**, Sensla, **AA**, route Bab El Khemis

﻿***Dacus* Fabricius, 1805**

﻿*Dacus ﻿﻿﻿frontalis* (Becker, 1922)

El Harym and Belqat 2017, **AA**, Oued Foum Ziguid (Douar Ouaiftoute), Oued Draa (Ikhf Mezrou), Isdaoun

﻿*Dacus ﻿﻿﻿longistylus* (Wiedemann, 1830)

El Harym and Belqat 2017, **AA**, Oued Tata, Douar Tighrimt, Oued Foum Ziguid (Douar Ouaiftoute)

##### Tephritinae

Dithrycini

﻿***Oedaspis* Loew, 1862**

﻿*Oedaspis ﻿﻿﻿daphnea* Séguy, 1930

Séguy 1930a, **AP**, El Mers (Rabat); Foote 1984; Soós 1984b; Mouna 1998; Norrbom et al. 1999; El Harym and Belqat 2017

﻿*Oedaspis ﻿﻿﻿multifasciata* (Loew, 1850)

= ﻿*Oedaspis ﻿﻿﻿multifasciatus* (Loew), in Séguy 1953a: 85

Séguy 1953a, **EM**, Itzer (Haute Moulouya); El Harym and Belqat 2017

﻿*Oedaspis ﻿﻿﻿trotteriana* Bezzi, 1913

Soós 1984b; Ribera and Blasco-Zumeta 1998; Norrbom et al. 1999; El Harym and Belqat 2017

##### Myopitini

﻿***Myopites* Blot, 1827**

﻿*Myopites ﻿﻿﻿cypriacus* Hering 1938

El Harym et al. 2020, **Rif**, Douar Halila, Dam Nakhla, Marabout Sidi Bou Hadjel

﻿*Myopites ﻿﻿﻿inulaedyssentericae* Blot, 1827^43^

= ﻿*Myopites ﻿﻿﻿apicatus* Freidberg, 1980, in El Harym and Belqat 2017: 140

El Harym and Belqat 2017, **Rif**, affluent Tarmast, Aïn Afersiw

﻿*Myopites ﻿﻿﻿longirostris* (Loew, 1846)

El Harym et al. 2020, **Rif**, Oued Tahaddart, Douar Kouf, Mkhinak

﻿*Myopites ﻿﻿﻿stylatus* Fabricius, 1794

El Harym and Belqat 2017, **Rif**, affluent Tarmast, Oued El Hamma, El Haouta

﻿*Myopites ﻿﻿﻿variofasciatus* Becker, 1903

= ﻿*Myopites ﻿﻿﻿variofasciata* Becker, in Séguy 1941a: 32

Séguy 1941a, **HA**, Imi-n’Ouaka (1500 m), Mouna 1998

﻿***Urophora* Robineau-Desvoidy, 1830**

﻿*Urophora ﻿﻿﻿congrua* Loew, 1862^44^

= *Euribia ﻿﻿﻿congrua* Loew, in Séguy 1941d: 14

Séguy 1941d, **AA**, Taroudant; Mouna 1998; El Harym and Belqat 2017

﻿*Urophora ﻿﻿﻿jaculata* Rondani, 1870

= ﻿*Urophora ﻿﻿﻿mauritanica* Macquart, in El Harym and Belqat 2017: 149 (misidentification)

﻿*Urophora ﻿﻿﻿mauritanica* Macquart, 1851

= *Euribia ﻿﻿﻿﻿﻿﻿﻿﻿﻿algira* Macquart, in Séguy 1930a: 169

= ﻿*Urophora ﻿﻿﻿﻿﻿﻿﻿﻿﻿algira* Macquart, in Séguy 1934a: 98, Mouna 1998: 87

= ﻿*Urophora ﻿﻿﻿macrura* Loew, in Séguy 1934a: 100, Mouna 1998: 87

Séguy 1930a, **HA**, Imi-N’Takandout, Dar Kaid M’Tougui; Séguy 1934a; White and Korneyev 1989, **HA**, Ito; Mouna 1998; Norrbom et al. 1999; Koçak and Kemal 2010; Koçak and Kemal 2013b

﻿*Urophoraquadrifasciata ﻿algerica* (Hering, 1941)

= *Euribiaquadrifasciata* Meigen, in Séguy 1930a: 169

Séguy 1930a (common in all North Africa); Mouna 1998; El Harym and Belqat 2017

El Harym et al. 2020, **MA**, Douar Oulad Abdoune, Mlakite, Tirra, Douar Oulad Amar, Tihli, Douar Oulad Amar

﻿*Urophora ﻿﻿﻿solstitialis* (Linnaeus, 1758)

= *Euribia ﻿﻿﻿solstitialis* Linnaeus, in Séguy 1930a: 169

Séguy 1930a, **HA**, Haute Réghaya; Mouna 1998; El Harym and Belqat 2017

##### Noeetini

﻿***Ensina* Robineau-Desvoidy, 1830**

﻿*Ensina ﻿﻿﻿sonchi* (Linnaeus, 1767)

El Harym and Belqat 2017, **Rif**, Ksar Rimal, Douar Tizga, Oued Kbir, **MA**, Sensla, **AA**, Oued Massa (Pont Aghbalou), Centre Sidi Ouassay, Aïn Boharroch, Atbane, Oued Tamanarne, Oued Draa (Tahtah), Jnane Makadir, Kasbah Asma, Ait Aissa O Brahim, Oued Ziz (Pont Errachidia), Oued Ouarzazate; **AA** (Tafraout (Al Ourir, 12 km E)) – MHNNR

﻿***Hypenidium* Loew, 1862**

﻿*Hypenidium ﻿﻿﻿graecum* Loew, 1862

= *Stephanaciura ﻿﻿﻿bipartita* Séguy, in Séguy 1930a: 171

Séguy 1930a, **MA**, Tiffert (2000–2200 m); Villeneuve 1933; Soós 1984b; Mouna 1998: 87; Norrbom et al. 1999; El Harym and Belqat 2017

##### Tephrellini

﻿***Aciura* Robineau-Desvoidy**

﻿*Aciura ﻿﻿﻿coryli* (Rossi, 1790)

= ﻿*Aciura ﻿Powelli* Séguy, in Séguy 1930a: 170, Séguy 1953a: 85, Mouna 1998: 87

Séguy 1930a, **MA**, Azrou (larvae from *Phlomis ﻿﻿﻿crinita* Cav.); Séguy 1953a, **AP**, Korifla; Mouna 1998; Norrbom et al. 1999, **MA**, Azrou; El Harym and Belqat 2017; El Harym et al. 2020, **Rif**, Arhil

﻿***Oxyaciura* Hendel, 1927**

﻿*Oxyaciura ﻿﻿﻿tibialis* (Robineau-Desvoidy, 1830)

= ﻿*Aciura ﻿﻿﻿tibialis* Robineau-Desvoidy, in Becker and Stein 1913: 94

Becker and Stein 1913, **Rif**, Tanger; Séguy 1930a, **MA**, Sker; Séguy 1934a; Mouna 1998; Koçak and Kemal 2010; Koçak and Kemal 2013b; El Harym and Belqat 2017, **Rif**, Dayat El Birdiyel, Oued Azila, maison forestière; Oued Maâza (Tarik El Ouasâa); **AP** (Tamri, 10 km S) – MHNNR

﻿***Sphaeniscus* Becker, 1908**

﻿*Sphaeniscus ﻿﻿﻿filiolus* (Loew, 1869)

= ﻿*Spheniscomyia ﻿﻿﻿filiola* Loew, in Séguy 1930a: 170; Mouna 1998: 87

= ﻿*Spheniscomyia ﻿﻿﻿aegyptiaca* Efflatoun, in Séguy 1949a: 157; Mouna 1998: 87

Séguy 1930a; Séguy 1949a, **SA**, Guelmim; Mouna 1998; El Harym and Belqat 2017, **Rif**, affluent Tarmast, Oued Maâza (Tarik El Ouasâa)

##### Tephritini

﻿***Acanthiophilus* Becker, 1908**

﻿*Acanthiophilus ﻿﻿﻿helianthi* (Rossi, 1790)

= ﻿*Tephritis ﻿﻿﻿eluta* Meigen, in Becker and Stein 1913: 94

= *Orellia ﻿﻿﻿eluta* Meigen, in Mouna 1998: 87

Becker and Stein 1913, **Rif**, Tanger; Séguy 1930a, **Rif**, Tanger (Sarf, route Spartel), **MA**, Tizi s’Tkrine (Jebel Ahmar, 1700 m); Séguy 1949a, **AA**, Alnif, Foum-el-Hassan, **SA**, Guelmim; Mouna 1998; Koçak and Kemal 2013b; El Harym and Belqat 2017, **Rif**, Oued Zinat, Ksar Rimal, Oued Dardara, affluent Tarmast, Dayat El Birdiyel, Oued Azila, Dayat Jebel Zemzem, Oued Boumarouil, Douar Abou Boubnar (Mara­bout Sidi Gile), El Haouta, Oued Maâza (Tarik El Ouasâa), Douar Tizga, Dayat Afrate, Oued Mezine, Aïn El Malaâb, Oued Jnane Niche, **MA**, Oued Oum-er-Rbia, **AA**, Centre Sidi Ouassay, Avant Sidi Bin­zarne, route Bab El Khemis, airport Sidi Ifni, Oued Tisla, Oued Sayad, Oued Tamanarne, Oued Foum Ziguid (Douar Ouaiftoute), Jnane Makadir, Douar Rggaga, Oued Tinghir, Oued Ouarzazate; **AP** (Essaouira (Cap Hadid), Tamri, 10 km S) – MHNNR

﻿***Campiglossa* Rondani, 1870**

﻿*Campiglossa ﻿﻿﻿martii* (Becker, 1908)

El Harym and Belqat 2017, **Rif**, Oued Kbir, **AA**, Centre Sidi Ouassay

﻿*Campiglossa ﻿﻿﻿producta* (Loew, 1844)

= *Oxyna ﻿﻿﻿tessellata* Loew, in Becker and Stein 1913: 94 (misidentification)

= *Paroxyna ﻿﻿﻿tessellata* (Loew), in Séguy 1930a: 174; Mouna 1998: 87; Koçak and Kemal 2013b: 38 (misidentification)

Becker and Stein 1913, **Rif**, Tanger; Séguy 1930a, **Rif**, Tanger, **AP**, Mogador, **HA**, Telouet Glaoua; Séguy 1934a; Séguy 1941a, **HA**, Tachdirt (Toubkal, 2500 m); Mouna 1998; Koçak and Kemal 2013b, **HA**; El Harym and Belqat 2017, **Rif**, Oued Al Mizzine, Aïn El Malaâb, Dayat El Hajjami

﻿*Campiglossa ﻿﻿﻿sororcula* (Wiedemann, 1830)

= *Dioxyna ﻿﻿﻿sororcula* (Wiedemann), in El Harym and Belqat 2017: 155

El Harym and Belqat 2017, **Rif**, Ksar Rimal, Oued Jnane Niche, Oued Halila, Oued Zarka, Oued Martil, Oued Amsa, Oued Sahel, Dayat Jebel Zemzem, Oued Maggou, Dhar Sbagh Mâasra, Douar Kitane

﻿***Capitites* Foote & Freidberg, 1981**

﻿*Capitites ﻿﻿﻿augur* (Frauenfeld, 1857)

= *Trypanea ﻿﻿﻿augur* (Frauenfeld), in Séguy 1930a: 176; Mouna 1998: 87

Séguy 1930a, **MA**, Forêt Azrou, Tizi-s’Tkrine (Jebel Ahmar, 1700 m), **HA**, Tenfecht, **AA**, Souss; Mouna 1998; Pârvu et al. 2006, **AA**, Ouarzazate, Lac Edehby; Popescu-Mirceni 2011, **MA**, Tizi-s’Tkrine, Azrou, **HA**, Tenfecht, **AA**, Ouarzazate

﻿*Capitites ﻿﻿﻿ramulosa* (Loew, 1844)

= ﻿*Acanthiophilus ﻿﻿﻿ramulosus* Loew, in Séguy 1930a: 177; Séguy 1941d: 15; Séguy 1949a: 157; Mouna 1998: 87

Séguy 1930a, **MA**, Timelilt, **HA**, Tizi-n’Test (Jebel Imdress, 2000–2450 m); Séguy 1941d, **AA**, Taroudant; Séguy 1949a, **AA**, Foum-el-Hassan, Akka, Agdz, Alnif; Mouna 1998; El Harym and Belqat 2017; El Harym et al. 2020, **Rif**, Amsa, Koudiat Taifour

﻿***Desmella* Munro, 1957**

﻿*Desmella ﻿﻿﻿rostellata* (Séguy, 1941)

= *Paroxyna ﻿﻿﻿rostellata* Séguy, in Séguy 1941d: 14; Mouna 1998: 87

Séguy 1941d, **AP**, Agadir; Soós 1984b; Mouna 1998; Norrbom et al. 1999, **AP**, Agadir; El Harym and Belqat 2017

﻿***Euaresta* Loew, 1873**

﻿*Euaresta ﻿﻿﻿bullans* (Wiedemann, 1830)

Herman and Dirlbek 2006, **AA**, Tiznit environs, Sidi Moussa d’Aglou; El Harym and Belqat 2017, **AA**, Msidira

﻿***Goniurellia* Hendel, 1927**

﻿*Goniurellia ﻿﻿﻿longicauda* Freidberg, 1980

Freidberg 1980, **MA**, Tizi-s’Tkrine (1700 m), Azrou, **AA**, Taroudant; Soós 1984b; Freidberg and Kugler 1989; Norrbom et al. 1999; El Harym and Belqat 2017, **AA**, airport Sidi Ifni, Oued Tisla, Oued Tamanarne, Douar Zaouiet, Oued Tata, Douar Tighrimt, Ksibat Elhdeb, Oued Ziz (Pont Errachidia), Oued Ouarzazate

﻿*Goniurellia ﻿﻿﻿persignata* Freidberg, 1980

Freidberg 1980, **EM**, Defilia, near Figuig; Soós 1984b; Freidberg and Kugler 1989; Norrbom et al. 1999; Herman and Dirlbek 2006, **AA**, Tiffoultoute (1146 m); El Harym and Belqat 2017, **Rif**, Dhar Sbagh Mâasra, **AA**, Douar Zaouiet, Oued Ouarzazate

﻿***Spathulina* Rondani, 1856**

﻿*Spathulina ﻿﻿﻿sicula* Rondani, 1856

= ﻿*Spathulina ﻿﻿﻿tristis* Loew, in Séguy 1930a: 174; Mouna 1998: 87

Séguy 1930a; Mouna 1998; El Harym and Belqat 2017, **Rif**, Barrage Smir

﻿***Sphenella* Robineau-Desvoidy, 1830**

﻿*Sphenellamarginata* (Fallén, 1814)

Becker and Stein 1913, **Rif**, Tanger; Séguy 1930a, **Rif**, Oued Judios (Tanger); Mouna 1998; Cassar et al. 2005, **Rif**, lagoon Smir; Koçak and Kemal 2013b, **HA**; El Harym and Belqat 2017, **Rif**, affluent Tarmast, Dayat Jebel Zemzem, El Malaâb, Oued Maâza (Âachira), Dayat Aïn Jdioui, Oued Maggou, Dayat Afrate

﻿***Tephritis* Latreille, 1804**

﻿*Tephritis ﻿﻿﻿carmen* Hering, 1937

El Harym et al. 2020, **Rif**, forest house of National Park of Talassemtane

﻿*Tephritis ﻿﻿﻿dioscurea* (Loew, 1856)

Séguy 1930a, **MA**, El Hajeb; Mouna 1998; El Harym and Belqat 2017

﻿*Tephritis ﻿﻿﻿divisa* (Rondani, 1871)

El Harym and Belqat 2017, **Rif**, Dayat Amsemlil

﻿*Tephritis ﻿﻿﻿formosa* (Loew, 1844)

Séguy 1930a, **HA**, Asni; Mouna 1998; El Harym and Belqat 2017, **Rif**, Oued Abou Bnar, Oued Sidi Ben Saâda, Oued Achekrade, Oued El Kanar

﻿*Tephritis ﻿﻿﻿leontodontis* (De Geer, 1776)

Séguy 1930a (All North Africa); Mouna 1998; El Harym and Belqat 2017

﻿*Tephritis ﻿﻿﻿matricariae* (Loew, 1844)

Séguy 1930a (all North Africa); Mouna 1998; El Harym and Belqat 2017, **Rif**, affluent Oued Amsemlil, El Haouta, Dayat Jebel Zemzem, Dayat Amsemlil, Oued El Hamma

﻿*Tephritis ﻿﻿﻿nigricauda* (Loew, 1856)

Séguy 1930a, **AP**, Berrechid; Séguy 1934a; Séguy 1941d, **AP**, Agadir, Berrechid; Mouna 1998; El Harym and Belqat 2017, **Rif**, Dayat Jebel Zemzem, Oued Maâza (Tarik El Ouasâa), Aïn El Maounzil, Dayat Tazia, Dayat Amsemlil, Douar Tamakoute

﻿*Tephritis ﻿﻿﻿postica* (Loew, 1844)

Herman and Dirlbek 2006, **MA**, Volubilis (358 m); El Harym and Belqat 2017, **AA**, Ksibat Elhdeb, Oued Tinghir

﻿*Tephritis ﻿﻿﻿praecox* (Loew, 1844)

Séguy 1930a, **Rif**, Oued Judios (Tanger), **MA**, Tizi-s’Tkrine (Jebel Ahmar, 1700 m); Mouna 1998; Herman and Dirlbek 2006, **MA**, Ifrane, Azrou National Park (1743 m); El Harym and Belqat 2017, **Rif**, Dayat El Ânassar, Dayat Amsemlil, affluent Oued Amsemlil, Douar Dacheryène, Douar Taghbaloute, Barrage Nakhla, Oued Sa­hel, Daya Jebel Zemzem, Douar Kitane, Oued El Hamma, Oued Kbir, Aïn el Ma Bared, Aïn El Malaâb, Douar Abou Boubnar (Marabout Sidi Gile), maison forestière, Douar Tizga, Oued Aïn Jdioui (Touaret), Dayat Afrate, Oued Jbara, Aïn El Maounzil, Dayat Tazia, Oued Jnane Niche, Oued Maggou, Aïn Tiouila, Dayat Lemtahane, Lâazaba, Dhar Sbagh Mâasra, El Hajria, Aïn Boharroch, Douar Tamakout, Douar Ouslaf, **EM**, Oued Béni Ouaklane (Béni Snassen); **AP** (Essaouira) – MHNNR

﻿*Tephritis ﻿﻿﻿pulchra* (Loew, 1844)

Séguy 1930a; Mouna 1998; El Harym and Belqat 2017

﻿*Tephritis ﻿﻿﻿simplex* (Loew, 1844)

Séguy 1930a; Mouna 1998; El Harym and Belqat 2017; El Harym et al. 2020, **Rif**, Aïn Soualah, Aïn El Maounzil

﻿*Tephritis ﻿﻿﻿stictica* Loew, 1862

Séguy 1930a, **AP**, Rabat; Mouna 1998; El Harym and Belqat 2017

﻿*Tephritis ﻿﻿﻿theryi* Séguy, 1930

Séguy 1930a, **HA**, Marrakech, Asni; Soós 1984b; Mouna 1998; Norrbom et al. 1999; El Harym and Belqat 2017

﻿*Tephritis ﻿﻿﻿vespertina* (Loew, 1844)

El Harym and Belqat 2017, **Rif**, Dayat Lemtahane, Dhar Sbagh Mâasra

﻿***Tephritomyia* Hendel, 1927**

﻿*Tephritomyia ﻿﻿﻿lauta* (Loew, 1869)

= ﻿*Acanthiophilus ﻿﻿﻿lauta* Loew, in Séguy 1930a: 177; Mouna 1998: 87

Séguy 1930a, **HA**, Tachdirt (Imminen, 2400–2600 m); Freidberg and Kugler 1989; Mouna 1998; Yaran and Kütük 2012; Morgulis et al. 2015, **HA**, Tizi-n’Tichka; El Harym and Belqat 2017, **Rif**, Dayat El Birdiyel, Dayat Amsemlil, Lâazaba, **AA**, Msidira, Oued Ouarzazate

﻿***Trupanea* Schrank, 1795**

﻿*Trupanea ﻿﻿﻿﻿amoena* (Frauenfeld, 1857)

= *Trypanea ﻿﻿﻿﻿amoena* Frauenfeld, in Séguy 1930a: 176; Mouna 1998: 87

Séguy 1930a, **MA**, Aïn Leuh; Mouna 1998; El Harym and Belqat 2017, **Rif**, Ksar Rimal, Oued Jnane niche, affluent Tarmast, Oued Martil (Tamouda), Oued Amsa, Oued El Hamma, Oued Boumarouil, Oued Sidi Yahia Aârab, Aïn Tiouila, **AA**, Oued Massa (Pont Aghbalou), Centre Sidi Ouassay, Avant Sidi Binzarne, Oued Tisla, Douar Tighrimt, Oued Draa (Tahtah), Jnane Makadir, Douar Rggaga, Aït Aissa O Brahim, Oued Draa (Ikhf Mezrou), Isdaoun, Ksibat Elhdeb, Oued Tinghir

﻿*Trupanea ﻿﻿﻿guimari* (Becker, 1908)

El Harym and Belqat 2017, **AA**, Centre Sidi Ouassay, Msidi­ra, Jnane Makadir, Aït Aissa O Brahim, Ksibat Elhdeb; Norrbom 2004: 06600136 – INHS (**AA**, 5 km W Ouarzazate)

﻿*Trupanea ﻿﻿﻿stellata* (Fuesslin, 1775)

Séguy 1930a, **MA**, Timelilt (1900 m); Séguy 1949a, **SA**, Guelmim; Mouna 1998; El Harym and Belqat 2017, **Rif**, Mizoghar, Oued Maâza (Tarik El Ouasâa), Dayat Afrate, Aïn El Malâab, Oued Tkarae, **AA**, Centre Sidi Ouassay; **AA** (Taliouine) – MHNNR

##### Terellini

﻿***Chaetorellia* Hendel, 1927**

﻿*Chaetorellia ﻿﻿﻿conjuncta* (Becker, 1912)

El Harym and Belqat 2017, **AA**, Airport Sidi Ifni, Oued Assa, Oued Sayad, Oued Foum Ziguid (Douar Ouaiftoute), Ksibat Elhdeb, Oued Ziz (Pont Errachidia), Oued Ouarzazate

﻿*Chaetorellia ﻿﻿﻿hestia* Hering, 1937

= ﻿*Chaetorellia ﻿﻿﻿hexachaeta* Loew, in Séguy 1930a: 174 [probably a misidentification]

= *Orellia ﻿﻿﻿hexachaeta* Loew, in Séguy 1934a: 135 [misidentification, see White and Macquart 1989: 476]; Mouna 1998: 87

Séguy 1930a, **AP**, Mogador; Séguy 1934a; Mouna 1998; El Harym and Belqat 2017, **AA**, Centre Sidi Ouassay

﻿*Chaetorellia ﻿﻿﻿succinea* (Costa, 1844)

El Harym et al. 2020, **MA**, Douar Oulad Abdoune

﻿***Chaetostomella* Hendel, 1927**

﻿*Chaetostomellacylindrica* (Robineau-Desvoidy, 1830)

El Harym et al. 2020, **Rif**, Marabout Douar Halila, Mkhinak, Douar Kitane

﻿***Terellia* Robineau-Desvoidy, 1830**

﻿*Terelliacolon* (Meigen, 1826)

= *Orellia ﻿﻿﻿colon* (Meigen), in Séguy 1930a: 173

Séguy 1930a (common in all North Africa); Mouna 1998; El Harym and Belqat 2017

﻿*Terellia ﻿﻿﻿fuscicornis* (Loew, 1844)

Séguy 1930a (common in all North Africa); Mouna 1998; El Harym and Belqat 2017

﻿*Terellia ﻿﻿﻿longicauda* (Meigen, 1838)

Séguy 1930a, **MA**, Aïn Leuh (1200–1400 m), **HA**, Tizi-n’Test, Goundafa (Jebel Imdress, 2000–2450 m); Séguy1934a; Séguy 1941a, **HA**, Tachdirt (Toubkal, 2500 m); Mouna 1998; El Harym and Belqat 2017

﻿*Terellia ﻿﻿﻿﻿luteola* (Wiedemann, 1830)

El Harym et al. 2020, **Rif**, Bakrim, Aforidane, Aïn Siyed

﻿*Terellia ﻿﻿﻿oasis* (Hering, 1938)

El Harym et al. 2020, **Rif**, Douar Halila

﻿*Terellia ﻿﻿﻿ptilostemi*El Harym et al. 2021

El Harym et al. 2021, **Rif**, Douar Chourdane (908 m), Aïn Akorian (1610 m), Aïn Elma Sefli (1345 m), Forest house of the Talassemtane National Park (1674 m)

﻿*Terellia ﻿﻿﻿serratulae* (Linnaeus, 1758)

= ﻿*Tephritis ﻿﻿﻿pallens* Wiedemann, in Wiedemann 1824: 54

= *Trypeta ﻿﻿﻿serratula* Linnaeus, in Becker and Stein 1913: 94

= ﻿*Terellia ﻿﻿﻿serratulae* Linnaeus, in Séguy 1930a: 173

Wiedemann 1824, **Rif**, Tanger; Becker and Stein 1913, **Rif**, Tanger; Séguy 1930a (all North Africa); Mouna 1998; Norrbom et al. 1999; El Harym and Belqat 2017, **Rif**, Dayat Jebel Zemzem, Oued Maâza (Tarik El Ouasâa)

﻿*Terellia ﻿virens* (Loew, 1846)

Séguy 1930a; White 1989, **HA**, Jebel Ayachi; Mouna 1998; Korneyev et al. 2013, **HA**, Tizi-n’Talrhemt; El Harym and Belqat 2017, **AA**, airport Sidi Ifni, Oued Ouarzazate

##### Trypetinae

Carpomyini

﻿***Carpomya* Costa, 1854**

﻿*Carpomya ﻿﻿﻿incompleta* (Becker, 1903)

El Harym and Belqat 2017, **AA**, Douar Zaouia

﻿***Euleia* Walker, 1835**

﻿*Euleia ﻿﻿﻿heraclei* (Linnaeus, 1758)

= *Acidia ﻿﻿﻿heraclei* Linnaeus, in Séguy 1953a: 85

Séguy 1953a, **MA**, Sidi Slimane; Freidberg and Kugler 1989; Koçak and Kemal 2010; El Harym and Belqat 2017, **Rif**, Oued Boumarouil, Aïn El Âakba Larbaâ

﻿*Euleia ﻿﻿﻿marmorea* (Fabricius, 1805)^45^

= *Philophylla ﻿flavescens* Fabricius, in Séguy 1930a: 170; Mouna 1998: 87

= ﻿*Euleiaflavescens* Fabricius, in Soós 1984b: 88

Séguy 1930a, **Rif**, Tanger; Zimsen 1964; Soós 1984b; Mouna 1998; Norrbom et al. 1999, **Rif**, Tanger; El Harym and Belqat 2017

##### Trypetini

﻿***Chetostoma* Rondani, 1856**

﻿*Chetostoma ﻿﻿﻿curvinerve* Rondani, 1856

El Harym and Belqat 2017, **Rif**, Oued Kelaâ, Bab el Karn

##### Acknowledgments

We gratefully acknowledge the assistance and cooperation of Valery Korneyev and the late Amnon Freidberg who contributed to the revision of this family.

#### ﻿﻿ULIDIIDAE

K. Kettani, M.J. Ebejer

Number of species: **13**. Expected: 18

Faunistic knowledge of the family in Morocco: good

##### Otitinae

﻿***Ceroxys* Macquart, 1835**

﻿*Ceroxys ﻿﻿﻿urticae* Linnaeus, 1758

Ebejer et al. 2019, **AP**, Lower Loukous (6 m), Larache (5 m)

﻿***Dorycera* Meigen, 1830**

﻿*Dorycera ﻿﻿﻿griseipennis* (Becker, 1907)

Soós 1984b

﻿***Herina* Robineau-Desvoidy, 1830**

﻿*Herina ﻿﻿﻿ghilianii* Rondani, 1869

Kameneva 2007, **HA**, Ansegmir-Tal, W Midelt (1400 m); Ebejer 2015

﻿*Herina ﻿﻿﻿lacustris* (Meigen, 1826)

Kameneva 2007, **Rif**, Baie de Tanger

﻿*Herina ﻿﻿﻿oscillans* (Meigen, 1826)

= ﻿*Herina ﻿﻿﻿schlueteri* Becker, in Becker and Stein 1913: 92; Soós 1984b: 56

Becker and Stein 1913, **Rif**, Tanger; Soós 1984b; Kameneva 2007, **Rif**, Tanger

﻿***Melieria* Robineau-Desvoidy, 1830**

﻿*Melierianigritarsis* Becker, 1903

Ebejer et al. 2019, **AA**, Merzouga (714 m)

﻿***Otites* Latreille, 1804**

﻿*Otites ﻿﻿﻿tangeriana* Becker, 1913

= ﻿*Otites ﻿﻿﻿tangeriana* Becker, in Becker and Stein 1913, 1918: 92

Becker and Stein 1913, 1918, **Rif**, Tanger; Soós 1984b, **Rif**, Tanger

﻿***Tetanops* Fallén, 1820**

﻿*Tetanopsflavescens* Macquart, 1835

Séguy 1930a, **Rif**, Tanger; Mouna 1998

##### Ulidiinae

﻿***Physiphora* Fallén, 1810**

﻿*Physiphoraalceae* (Preyssler, 1791)

= *Chrysomyza ﻿﻿﻿demandata* (Fabricius, 1798), in Séguy 1953a: 85; Mouna 1998: 87

Becker and Stein 1913, **Rif**, Tanger; Séguy 1930a, **HA**, Goundafa; Séguy 1953a, **SA**, El Aöun du Draa; Séguy 1949a, **AA**, Agdz; Mouna 1998; Koçak and Kemal 2010; Kameneva and Korneyev 2016, **AA**, Tizi-n’Bachkoun (1600 m); **Rif** (M’Diq farm) – MISR; **Rif** (Tanger) – MfN; **AP** (Casablanca) – ZSSM

﻿*Physiphora ﻿﻿﻿smaragdina* (Loew, 1852)

Kameneva and Korneyev 2016, **AA**, 25 km S Goulmima (100 m) – NHMD

﻿***Ulidia* Meigen, 1826**

﻿*Ulidia ﻿﻿﻿﻿﻿﻿﻿﻿﻿﻿apicalis* (Meigen, 1826)

Séguy 1930a, **MA**, Meknès, **HA**, Skoutana; Séguy 1934a; Lyneborg 1969; Soós 1984b; Mouna 1998

﻿*Ulidia ﻿﻿﻿erythrophthalma* Meigen, 1826

Becker and Stein 1913, **Rif**, Tanger; Séguy 1949a, **AA**, Foum-el-Hassan; Mouna 1998

﻿*Ulidia ﻿﻿﻿megacephala* Loew, 1845

Soós 1984b; Zaitzev 1984; Koçak and Kemal 2010

### ﻿Lauxanioidea

#### ﻿﻿CHAMAEMYIIDAE

K. Kettani, M.J. Ebejer

Number of species: **18**. Expected: 24

Faunistic knowledge of the family in Morocco: moderate

##### Chamaemyiinae

﻿***Chamaemyia* Meigen, 1803**

﻿*Chamaemyia ﻿﻿﻿aridella* (Fallén, 1823)

Ebejer 2016a, **Rif**, Moulay Abdelsalam (1180 m), Issaguen (1620 m); **HA** (Jebel Ayachi, Mikdane, maison forestière, MR Jaffar, Tizi-n’Zou) – NHMUK

﻿*Chamaemyia ﻿﻿﻿flavicornis* (Strobl, 1902)

Ebejer 2016a, **Rif**, Martil beach and dunes (on human faeces), **MA**, Khénifra (17 km NW of Zaida, 1878 m), **AA**, Errachidia (29 km N of Rich, 1570 m); **HA** (Asni near Alrene, W Imlil) – NHMUK

﻿*Chamaemyia ﻿﻿﻿flavipalpis* (Haliday, 1838)

= ﻿*Chamaemyia ﻿﻿﻿maritima* Zetterstedt, in Mouna 1998: 85

Mouna 1998; Ebejer 2016a, **Rif**, Ksar Sghir, Oued Araml, Ksar Sghir, **AP**, Larache, Merja Zerga, Loukous marsh

﻿*Chamaemyia ﻿﻿﻿herbarum* (Robineau-Desvoidy, 1830)

Ebejer 2016a, **MA**, Khénifra, 17 km SW of Midelt (1940 m), **AA**, Errachidia, 29 km N of Rich (1570 m); **HA** (Jebel Ayachi, Mikdane, MR Jaffar, Tizi-n’Zou) – NHMUK

﻿*Chamaemyia ﻿﻿﻿juncorum* (Fallén, 1958)

Ebejer 2016a, **MA**, Khénifra (17 km SW of Midelt, 1940 m), **AA**, Errachidia (29 km N of Rich, 1570 m)

﻿*Chamaemyia ﻿﻿﻿polystigma* (Meigen, 1830)

Becker and Stein 1913, **Rif**, Tanger; Ebejer 2016a, **Rif**, Moulay Abdelsalam (1180 m), Sidi Yahia Aarab (377 m); **HA** (Asni) – NHMUK

﻿***Melanochthiphila* Frey, 1958**

﻿*Melanochthiphila* ﻿sp. Ebejer, 2016

Ebejer 2016a, **AA**, Errachidia (30 km W of Errachidia, 1065 m)

﻿***Parochthiphila (Euestelia*) Enderlein, 1927**

﻿*Parochthiphila (Euestelia) ﻿coronata* (Loew, 1858)

Carles-Tolrá 1993; Mouna 1998; Ebejer 2016a, **AP**, Larache (Lower Loukous, 2 m); **HA** (Jebel Ayachi, Mikdane, MR Jaffar) – NHMUK

﻿*Parochthiphila ﻿﻿﻿frontella* (Rondani, 1874)

Ebejer 2016a; **HA** (Jebel Ayachi, Mikdane, Tizi-n’Zou, MR Jaffar) – NHMUK

﻿*Parochthiphila ﻿﻿﻿inconstans* Becker, 1903

Ebejer 2016a, **AA**, Ziz river (9.5 km SE of Rich, 1285 m), Errachidia (6 km N of Errachidia, 1010 m)

﻿*Parochthiphila (Euestelia) ﻿nigripes* Strobl, 1900

Ebejer 2016a, **Rif**, Ksar Sghir, Dardara

##### Leucopinae

﻿***Leucopis* Meigen, 1803**

﻿*Leucopis ﻿﻿﻿﻿annulipes* Zetterstedt, 1848

Mouna 1998; **AP** (Maâmora) – MISR

﻿*Leucopis ﻿﻿﻿griseola* (Fallén, 1823)

Mouna 1998; **AP** (Rabat), **MA** (Meknès), **HA** (Marrakech) – MISR

﻿*Leucopis ﻿﻿﻿formosana* Hennig, 1938

Ebejer 2016a, **AP**, Sidi Smail, Oued Tensift (estuary)

﻿*Leucopis ﻿﻿﻿glyphinivora* Tanasijtshuk, 1958

Ebejer 2016a, **AP**, Ksar Sghir, Azemmour, Oued Tensift (estuary), **AA**, Errachidia (30 km W of Errachidia)

﻿*Leucopis ﻿﻿﻿kerzhneri* Tanasijtshuk, 1970

Ebejer 2016a; **HA** (Jebel Ayachi) – NHMUK

﻿*Leucopis ﻿﻿﻿palumbii* Rondani, 1872

Ebejer 2016a, **Rif**, Ksar Sghir, Oued Aliane

﻿***Lipoleucopis* de Meijere, 1928**

﻿*Lipoleucopis ﻿﻿﻿pulchra* Raspi, 2008

Ebejer 2016a, **AA**, Errachidia, Ziz river (12 km S of Rissani)

#### ﻿﻿LAUXANIIDAE

K. Kettani, M.J. Ebejer

Number of species: **27**. Expected: 45

Faunistic knowledge of the family in Morocco: good

##### Homoneurinae

﻿***Homoneura* Wulp, 1891**

﻿*Homoneura ﻿﻿﻿ericpoli* Carles-Tolrá, 1993

Ebejer and Kettani 2019a, **Rif**, Azilane (1255 m), Adrou (556 m), **HA**, Lac Tislit (Imlchil, 2254 m)

﻿*Homoneura ﻿﻿﻿licina* Séguy, 1941

Séguy 1941d, **AA**, Agadir; Mouna 1998; Schacht et al. 2004; Ebejer and Kettani 2019a, **Rif**, Oued Laou (2 m), **AP**, Merja Zerga, Sidi Smaine, Safi (estuary of Oued Tensift), Azemmour (El Jadida), Diabat (Essaouira), Larache (5 m)

﻿*Homoneura ﻿﻿﻿transversa* (Wiedemann, 1830)

Wiedemann 1830; Ebejer and Kettani 2019a

﻿***Prosopomyia* Loew, 1856**

﻿*Prosopomyia ﻿pallida* Loew, 1856

Vaillant 1956b, **HA**, Oukaimeden, Imi-N’Ifri; Carles-Tolrá 1993; Mouna 1998; Ebejer and Kettani 2019a, **Rif**, Adrou (556 m), Issaguen (1547 m), **HA**, Jebel Ayachi – NHMUK

##### Lauxaniinae

﻿***Calliopum* Strand, 1928**

﻿*Calliopum ﻿﻿﻿oosterbroeki* Shatalkin, 2000

Ebejer and Kettani 2019a, **Rif**, Oued Zarka (Yarghite, 137 m), Moulay Abdelsalam (1180 m), Ketama, Rahbat Amlay (284 m), Ikadjiouen (294 m), Mechkralla (NPT, 981 m), Kharouba (roadside meadow between Chefchaouen and Tétouan, 385 m), Dbani (Béni Selmane, 1046 m), Aïn Ben Ali (Béni Selmane, 1014 m), **HA**, Lalla Takrkoust (Oued N'fis, 1141 m) – NHMUK

﻿*Calliopum ﻿﻿﻿tuberculosum* (Becker, 1895)

Ebejer and Kettani 2019a, **Rif**, Oued Zarka (Yarghite, 135 m), Azilane (1255 m), Perdicaris Park (Tanger, 223 m), cascade Chrafate (859 m)

﻿***Meiosimyza* Hendel, 1925**

﻿*Meiosimyza (Lyciella) ﻿rorida* (Fallén, 1820)

= ﻿*Homoneura ﻿﻿﻿rorida* Fallén, in Mouna 1998: 85

Mouna 1998; **MA** (Aïn Leuh) – MISR

﻿***Minettia* Robineau-Desvoidy, 1830**

﻿*Minettia ﻿﻿﻿aenigmatica* Ebejer, 2019

Ebejer 2019a, **Rif**, Perdicaris Park (223 m), Oued Khmis (Khmis Anjra, 61 m), **HA**, Mikdane (Jebel Ayachi); Ebejer and Kettani 2019a

﻿*Minettia ﻿﻿﻿biseriata* (Loew, 1847)

Ebejer and Kettani 2019a, **Rif**, Oued Khmis (Khmis Anjra, 61 m), Jnane Niche (46 m), Oued Kbir (Béni Ratene (PNPB), 157 m)

﻿*Minettia ﻿﻿﻿cantolraensis* Carles-Tolrá, 1998

Ebejer and Kettani 2019a, **Rif**, Oued Laou, El-Fahsa, Oued Guallet (Bni Boufrah, 942 m), Oued Ouringa (2 m), Maggou (NPT, 962 m), Ksar el-Kbir road to Chefchaouen at bridge near oued Azla (80 m), Oued Siflaou (281 m), Dardara (484 m), Azilane (1255 m), Adrou (556 m), Perdicaris Park (Tanger, 223 m), Bni Bahlou (986 m), El Hamma (936 m), Amaghouse (Oued Laou), Oued Koub (Laghdir, 148 m), **EM**, Aïn Sfa (Berkane, 638 m), Tafoughalt (Berkane, 788 m), **HA**, Lalla Takrkoust (Oued N'fis, 1141 m), **AA**, Taliouine (Taroudant, 1049 m)

﻿*Minettia ﻿fasciata* (Fallén, 1826)

Merz 2004, **AP**, Rabat; Ebejer and Kettani 2019a, **Rif**, Oued Khmis (Khmis Anjra, 61 m), Ksar el-Kbir road to Chefchaouen at bridge near oued Azla (80 m), Azilane (1255 m), Adrou (556 m), Issaguen (1547 m), Oued Taida (Al Andalous, 503 m), Perdicaris Park (Tanger, 223 m), El Hamma (936 m), **EM**, Aïn Sfa (Berkane, 683 m)

﻿*Minettia ﻿﻿﻿flavipalpis* (Loew, 1847)^46^

= ﻿*Sapromyza ﻿﻿﻿flavipalpis* Loew, 1847, in Becker and Stein 1913: 93 (**Rif**, Tanger), Ebejer and Kettani 2019a: 144 (???)

﻿*Minettia ﻿﻿﻿flaviventris* (Costa, 1844)

Ebejer and Kettani 2019a, **Rif**, Azilane (1255 m)

﻿*Minettia ﻿﻿﻿longiseta* (Loew, 1847)

Ebejer and Kettani 2019a, **Rif**, Maggou (NPT, 962 m), Adrou (PNPB, 556 m)

﻿*Minettia ﻿﻿﻿plumicornis* (Fallén, 1820)46

Schacht et al. 2004; Ebejer and Kettani 2019a

﻿*Minettia ﻿﻿﻿subvittata* (Loew, 1847)

Ebejer and Kettani 2019a, **Rif**, Oued Khmis (Khmis Anjra, 61 m), Azilane (1255 m)

﻿*Minettia ﻿﻿﻿suillorum* (Robineau-Desvoidy, 1830)

= ﻿*Minettia ﻿﻿﻿muricata* Becker, 1895, in Mouna 1998: 85

Mouna 1998; Ebejer and Kettani 2019a, **Rif**, Adrou (556 m), Perdicaris Park (Tanger, 223 m), **HA**, Mikdane (Jebel Ayachi) – NHMUK

﻿*Minettia ﻿﻿﻿tabidiventris* (Rondani, 1877)

Ebejer and Kettani 2019a, **Rif**, Adrou (556 m), Oued Kbir (Béni Ratene (PNPB), 157 m), Oued Koub (Laghdir, 148 m), Rahbat Amlay (284 m)

﻿***Pachycerina* Macquart, 1835**

﻿*Pachycerina ﻿﻿﻿pulchra* Loew, 1850

Ebejer and Kettani 2019a, **Rif**, Oued Zarka (Yarghite, 137 m)

﻿***Peplominettia* Szilády, 1943**

﻿*Peplominettia ﻿﻿﻿codinai* (Hennig, 1951)

Ebejer and Kettani 2019a, **Rif**, Oued Zarka (Yarghite, 137 m), Azilane (1255 m)

﻿*Peplominettia ﻿﻿﻿striata* Szilády, 1943

Ebejer and Kettani 2019a, **Rif**, Oued Zarka (Yarghite, 137 m)

﻿***Sapromyza* Fallén, 1810**

﻿*Sapromyza ﻿﻿﻿﻿﻿﻿﻿﻿﻿﻿apicalis* Loew, 1847

Schacht et al. 2004; Ebejer and Kettani 2019a, **HA**, Jebel Ayachi, Mikdane (stream), Lac Tislit (Imlchil, 2254 m) – NHMUK

﻿*Sapromyza ﻿﻿﻿gozmanyi* Papp, 1981

Ebejer and Kettani 2019a, **Rif**, Oued Kbir (Béni Ratene (PNPB), 157 m), Perdicaris Park (Tanger, 223 m), Barrage Smir (M’Diq, 27 m)

﻿*Sapromyza ﻿﻿﻿obscuripennis* Loew, 1847

Ebejer and Kettani 2019a, **Rif**, Jebel Talassemtane (1554 m), Jebel Lakraâ (1541 m)

﻿*Sapromyza ﻿﻿﻿unizona* Hendel, 1908

Ebejer and Kettani 2019a, **Rif**, Perdicaris Park (Tanger, 223 m), **AP**, Larache (5 m)

﻿*Sapromyza (Sapromyzosoma) ﻿laevatrispina* Carles-Tolrá, 1992

Ebejer and Kettani 2019a, **Rif**, Adrou (556 m), Issaguen (maison morestière, 1547 m)

﻿*Sapromyza (Sapromyzosoma) ﻿parallela* Carles-Tolrá, 1992

Ebejer and Kettani 2019a, **Rif**, Adrou (556 m), Azilane (1255 m), Talassemtane (maison forestière, 1699 m), **HA**, Jaffar river (Jebel Ayachi, at maison forestière), Mikdane (Jebel Ayachi) – NHMUK

### ﻿Sciomyzoidea

#### ﻿﻿COELOPIDAE

K. Kettani

Number of species: **1**. Expected: 2

Faunistic knowledge of the family in Morocco: moderate

##### Coelopinae

﻿***Coelopa* Meigen, 1830**

﻿*Coelopa ﻿﻿﻿pilipes* Haliday, 1838

Séguy 1930a, **Rif**, Tanger; Dakki 1997; Lair 2013

#### ﻿﻿DRYOMYZIDAE

K. Kettani

Number of species: **1**. Expected: 1

Faunistic knowledge of the family in Morocco: poor

##### Dryomyzinae

﻿***Dryope* Robineau-Desvoidy, 1830**

﻿*Dryope ﻿﻿﻿flaveola* (Fabricius, 1794)

Ebejer et al. 2019, **Rif**, Jebel Lakraâ (Talassemtane, 1596 m)

#### ﻿﻿HELCOMYZIDAE

K. Kettani

Number of species: **1**. Expected: 2

Faunistic knowledge of the family in Morocco: poor

##### Helcomyzinae

﻿***Helcomyza* Curtis, 1825**

﻿*Helcomyza ﻿﻿﻿ustulata* Curtis, 1825

Cassar et al. 2005, **Rif**, Smir lagoon

#### ﻿﻿SCIOMYZIDAE

K. Kettani, J-C. Vala

Number of species: **25**. Expected: 40

Faunistic knowledge of the family in Morocco: good

##### Sciomyzinae

Sciomyzini

﻿***Ditaeniella* Sack, 1939**

﻿*Ditaeniella ﻿﻿﻿grisescens* (Meigen, 1830)

Vala and Ghamizi 1991, **MA**, Ras el Ma, **AA**, Agadir; Kassebeer 1999a, **MA**, **HA**; Knutson and Vala 2011

﻿***Pherbellia* Robineau-Desvoidy, 1830**

﻿*Pherbellia ﻿﻿﻿cinerella* (Fallén, 1820)

= *Ditaenia ﻿﻿﻿cinerella* Fallén, in Séguy 1941a: 31

Séguy 1941a, **HA**, Tachdirt (Toubkal, 2500 m), Imi-n’Ouaka (1500 m); Leclercq and Schacht 1987, **HA**, Ansegmir-Tal, W Midelt, **HA**, Tizi-n’Test (1900 m); Rozkošný 1987, **HA**; Vala and Ghamizi 1991, **HA**, **MA**; Carles-Tolrá 1993; Kassebeer 1999a, **MA**, **HA**; **Rif** (Talassemtane) – MISR

﻿*Pherbellia ﻿﻿﻿dorsata* (Zetterstedt, 1846)

Kassebeer 1999a, **MA**; Knutson and Vala 2011

﻿*Pherbellia ﻿﻿﻿griseola* (Fallén, 1820)

Kassebeer 1999a, **MA**; Knutson and Vala 2011

﻿*Pherbellia ﻿﻿﻿hermonensis* Knutson & Freidberg, 1983

Kassebeer 1999a, **HA**; Knutson and Vala 2011

﻿*Pherbellia ﻿﻿﻿nana* (Fallén, 1820)

= ﻿*Pherbellia ﻿﻿﻿villiersi* Séguy, in Séguy 1941a: 31; Knutson 1981: 336 (new comb.)

Séguy 1941a, **HA**, Tachdirt (Toubkal, 2500 m); Knutson 1981, **HA**, Tachdirt; Leclercq and Schacht 1987, **HA**; Vala and Ghamizi 1989, **HA**; Kassebeer 1999a, **MA**, **HA**; Knutson and Vala 2011; **HA** – MISR

##### Tetanocerini

﻿***Dichaetophora* Rondani, 1868**

﻿*Dichaetophora ﻿﻿﻿obliterata* (Fabricius, 1805)

Séguy 1941a, **HA**, Tachdirt (Toubkal, 2500 m); Leclercq and Schacht 1987; Rozkošný 1987; Vala 1989; Vala and Ghamizi 1991; Kassebeer 1999a

﻿***Elgiva* Meigen, 1838**

﻿*Elgiva ﻿﻿﻿cucularia* (Linnaeus, 1767)

Leclercq and Schacht 1987, **HA**, Ansegmir-Tal, W Midelt (1400 m); Vala 1989; Kassebeer 1999a

﻿***Euthycera* Latreille, 1829**

﻿*Euthycera ﻿﻿﻿﻿﻿﻿﻿﻿﻿algira* (Macquart, 1849)

Vala and Reidenbach 1982, **Rif**, Tanger; Vala 1989, **Rif**, Tanger; Vala and Ghamizi 1991; Kassebeer 1999a, **Rif**, Tanger

﻿*Euthycera ﻿﻿﻿soror* (Robineau-Desvoidy, 1830)

= ﻿*Euthycera ﻿﻿﻿alaris* Vala, 1983, syn. nov.Vala (pers. comm.)

Vala 1989, **Rif**, Tanger; Vala and Ghamizi 1991, **MA**; Carles-Tolrá 1993; Kassebeer 1999a, **MA**, **HA**

﻿*Euthycera ﻿﻿﻿stichospila* (Czerny, 1909)

= ﻿*Euthycera ﻿﻿﻿leclercqi* Vala & Reidenba﻿ch, 1982, syn. by Rozkošný (1987)

Czerny and Strobl 1909; Vala and Reidenbach 1982; Rozkošný 1987

﻿*Euthycera ﻿﻿﻿zelleri* (Loew, 1847)

Kassebeer 1999a, **Rif**, Tanger

﻿***Hydromya* Robineau-Desvoidy, 1830**

﻿*Hydromya ﻿﻿﻿dorsalis* (Fabricius, 1775)

Séguy 1930a, **Rif**, Tanger; Leclercq and Schacht 1987, **HA**, Ansegmir-Tal, W Midelt (1400 m), **HA**, Oukaimeden (2600 m); Dakki 1997, **Rif**, Ketama; Vala 1989, **Rif**, Tanger; Vala and Ghamizi 1991, **MA**, **HA**; Kassebeer 1999a, **MA**, **HA**; Pârvu et al. 2006, **AP**, Merja Zerga

﻿***Ilione* Haliday in Curtis, 1837**

﻿*Ilione ﻿﻿﻿albiseta* (Scopoli, 1763)

Leclercq and Schacht 1987, **HA**; Vala 1989; Kassebeer 1999a

﻿*Ilione ﻿﻿﻿trifaria* (Loew, 1820)

Séguy 1941a, **HA**, Imi-n’Ouaka (1500 m); Vala 1989, **MA**, Dayat Aoua; Vala and Ghamizi 1991, **EM**, **MA**, **HA**; Carles-Tolrá 1993

﻿*Ilione ﻿﻿﻿﻿unipunctata* (Macquart, 1849)

Leclercq and Schacht 1987, **HA**; Rozkošný 1987; Vala 1989; Kassebeer 1999a

﻿***Oligolimnia* Mayer, 1953**

﻿*Oligolimnia ﻿﻿﻿zernyi* Mayer, 1953

Rozkošný 1987, **HA**, Tachdirt; Leclercq and Schacht 1987, **HA**; Vala 1989, **HA**; Kassebeer 1999a, **HA**

﻿***Pherbina* Robineau-Desvoidy, 1830**

﻿*Pherbina ﻿﻿﻿coryleti* (Scopoli, 1763)

Séguy 1930a, **Rif**, Tanger; Dakki 1997; Pârvu et al. 2006, **AP**, Merja Zerga; **HA** (Issougane n’Ouagouns) – MISR

﻿*Pherbinamediterranea* Mayer, 1953

Leclercq and Schacht 1987, **HA**, Ansegmir-Tal, W Midelt (1400 m); Rozkošný 1987; Vala 1989; Vala and Ghamizi 1991, **AP**, Sidi Boughaba, **HA**, south of Marrakech; Carles-Tolrá 1993; Kassebeer 1999a, **MA**

﻿ ***Psacadina* Enderlein, 1939**

﻿*Psacadina ﻿﻿﻿disjecta* Enderlein, 1939

Verbeke 1964, **AA**, Tlata Reisana; Leclercq and Schacht 1987, **HA**, Ansegmir-Tal, W Midelt (1400 m); Rozkošný 1987; Vala 1989; Kassebeer 1999a, **MA**

﻿*Psacadina ﻿﻿﻿verbekei* Rozkošný, 1975

Vala and Ghamizi 1991, **HA**, south of Marrakech; Kassebeer 1999a

﻿***Sepedon* Latreille, 1804**

﻿*Sepedon ﻿﻿﻿hispanica* Loew, 1862

Verbeke 1964; Vala 1989, **AP**, Mohammedia; Vala and Ghamizi 1991, **AP**, Mohammedia, **HA**, Oued Tissaout (Kelaâ Sraghna); Kassebeer 1999a

﻿*Sepedon ﻿﻿﻿sphegea* (Fabricius, 1775)

Séguy 1930a**Rif**, Tanger; Leclercq and Schacht 1987, **HA**, Ansegmir-Tal, W Midelt (1400 m); Rozkošný 1987; Vala 1989; Vala and Ghamizi 1991, **HA**, Tamesloht (south of Marrakech), **AA**, Bou Acheiba (Agadir); Carles-Tolrá 1993; Dakki 1997; Kassebeer 1999a

﻿*Sepedon ﻿﻿﻿spinipes* (Scopoli, 1763)

Leclercq and Schacht 1987, **HA**, Ansegmir-Tal, W Midelt (1400 m); Vala and Ghamizi 1991, **MA**, Imouzzer, Tifounassine; Carles-Tolrá 1993; Kassebeer 1999a, **MA**; Knutson and Vala 2011

﻿***Trypetoptera* Hendel, 1900**

﻿*Trypetoptera ﻿﻿﻿punctulata* (Scopoli, 1763)

Leclercq and Schacht 1987, **HA**; Vala 1989; Kassebeer 1999a; Knutson and Vala 2011

#### ﻿﻿SEPSIDAE

K. Kettani, J.-P. Haenni

Number of species: **12**. Expected: 20

Faunistic knowledge of the family in Morocco: poor

##### Sepsinae

Saltellini

﻿***Saltella* Robineau-Desvoidy, 1830**

﻿*Saltella ﻿﻿﻿sphondylii* (Schrank, 1803)

Ebejer et al. 2019, **Rif**, Barrage Smir (145 m), Oued Mhajrate (Ben Karrich, 180 m)

##### Sepsini

﻿***Nemopoda* Robineau-Desvoidy, 1830**

﻿*Nemopoda ﻿﻿﻿nitidula* (Fallén, 1820)

Mouna 1998 (no locality given)

﻿***Sepsis* Fallén, 1810**

﻿*Sepsis ﻿﻿﻿biflexuosa* Strobl, 1893

Zuska and Pont 1984; Mouna 1998; Pont and Meier 2002; Ozerov 2005; **HA** (Asni) – NHMUK

﻿*Sepsis ﻿﻿﻿cynipsea* (Linnaeus, 1758)

Séguy 1930a, **AP**, Rabat, **MA**, **HA**, Marrakech; Séguy 1941a, **HA**, Imi-n’Ouaka (1500 m); Zuska and Pont 1984; Mouna 1998

﻿*Sepsis ﻿﻿﻿flavimana* Meigen, 1826

Ebejer et al. 2019, **AA**, Ziz river (10 km S of Errachidia, 1008 m)

﻿*Sepsis ﻿﻿﻿fulgens* Meigen, 1826

Séguy 1941d, **HA**, Tizi-n’Test (2000 m), **AA**, Agadir; Zuska and Pont 1984; Mouna 1998; Pârvu et al. 2006, **MA**, Ifrane; Popescu-Mirceni 2011, **MA**, Ifrane; **MA** (Ifrane), **HA** (Asni, near Alrene, Mikdane) – NHMUK

﻿*Sepsislateralis* Wiedemann, 1830

Séguy 1941a, **HA**, Imi-n’Ouaka (1500 m); Zuska and Pont 1984; Mouna 1998; **AP** (Sale Tropical Garden), **HA** (Marrakech) – NHMUK

﻿*Sepsis ﻿﻿﻿punctum* (Fabricius, 1794)

Séguy 1941a, **HA**, Aït Souka (Toubkal); Zuska and Pont 1984; Mouna 1998; Pont and Meier 2002, **Rif**, Tanger, **AP**, Rabat; Pârvu et al. 2006, **AP**, Merja Zerga – MISR; **MA** (Ifrane), **HA** (Asni, Mikdane), **AA** (Souss Massa, Agadir) – NHMUK

﻿*Sepsis ﻿﻿﻿thoracica* (Robineau-Desvoidy, 1830)

Zuska and Pont 1984; Mouna 1998; Pârvu et al. 2006, **AP**, Merja Zerga; Popescu-Mirceni 2011; **MA** (near Azrou), **HA** (Mikdane, Marrakech, Amizmiz) – NHMUK

﻿*Sepsis ﻿﻿﻿violacea* Meigen, 1826

= ﻿*Sepsis ﻿﻿﻿ciliforceps* Duda, 1926, in Mouna 1998: 86

Becker and Stein 1913, **Rif**, Tanger; Zuska and Pont 1984; Mouna 1998; Pârvu et al. 2006, **AP**, Merja Zerga; Popescu-Mirceni 2011 – MISR

﻿***Themira* Robineau-Desvoidy, 1830**

﻿*Themiraminor* (Haliday, 1833)

Zuska and Pont 1984; Mouna 1998 (no locality given); **HA** (Mikdane) – NHMUK

﻿*Themira ﻿﻿﻿paludosa* Elberg, 1963^47^

Pârvu et al. 2006, **AP**, Merja Zerga; Popescu-Mirceni 2011

An additional species, *Australosepsis ﻿﻿﻿niveipennis* (Becker, 1903) is reported from Morocco in ﻿major catalogues (Pont and Meier 1984, Ozerov 2005). However this report is based upon a misinterpretation by Zuska (1968) of a name of locality given in Duda (1926b: 2): “Marako” [a locality in Mali, or possibly another in Ethiopia] was wrongly understood for “Marokko”, the German name of Morocco. Both Adrian Pont and Andrey Ozerov have confirmed (pers. comm. 27.xi.15) that they have not seen any Moroccan specimen of *A.﻿niveipennis*.

The Moroccan records from NHMUK were checked by Adrian Pont to whom we express our grateful thanks for his kind help.

### ﻿Opomyzoidea

#### ﻿﻿AGROMYZIDAE

K. Kettani, M. Černý

Number of species: **62**. Expected: 150

Faunistic knowledge of the family in Morocco: poor

##### Agromyzinae

﻿***Agromyza* Fallén, 1810**

﻿*Agromyza ﻿﻿﻿abiens* Zetterstedt, 1848

Spencer 1967, **HA**, Ourika Valley, Marrakech; Černý and Merz 2006

﻿*Agromyza ﻿﻿﻿albipennis* Meigen, 1830

Séguy 1936b; Mouna 1998

﻿*Agromyza ﻿﻿﻿﻿﻿ambigua* Fallén, 1823

= *Domomyza ﻿﻿﻿﻿﻿ambigua* Fallén, in Séguy 1936: 5; Mouna 1998: 84

Séguy 1936b, **AP**, Rabat; Mouna 1998

﻿*Agromyza ﻿﻿﻿bicaudata* (Hendel, 1920)

Černý and Merz 2006, **MA**, Azrou, Ifrane

﻿*Agromyza ﻿﻿﻿frontella* (Rondani, 1875)

Černý and Merz 2006, **MA**, Azrou, Ifrane

﻿*Agromyza ﻿﻿﻿frontosa* (Becker, 1908)

Černý and Merz 2006, **MA**, Azrou, Ifrane

﻿*Agromyza ﻿﻿﻿hiemalis* Becker, 1908

Spencer 1967, **HA**, Marrakech; Černý 2019; Černý et al. 2020

﻿*Agromyza ﻿﻿﻿intermittens* (Becker, 1907)

= ﻿*Phytomyza ﻿﻿﻿secalina* Hering, 1925, in Maarouf 2003: 42

Maarouf 2003, **HA**, Chaouia

﻿*Agromyza ﻿﻿﻿luteitarsis* (Rondani, 1875)

Maarouf 2003, **HA**, Chaouia

﻿*Agromyza ﻿﻿﻿megalopsis* Hering, 1933

Černý et al. 2020, **AA**, Agadir Id Aissa (western end of gorge and village Amtoudi, 854 m)

﻿*Agromyza ﻿﻿﻿nana* Meigen, 1830

Spencer 1967, 1973, **AP**, Casablanca; Černý and Merz 2006

﻿*Agromyza ﻿﻿﻿nigrociliata* (Hendel, 1931)

Maarouf 2003, **HA**, Chaouia

﻿*Agromyza ﻿﻿﻿rondensis* Strobl, 1900

Černý et al. 2020, **AA**, Agadir Id Aissa (western end of gorge and village Amtoudi, 854 m), River Aoulouz (= Asif Tifnout, 697 m)

﻿*Agromyza ﻿﻿﻿spenceri* Griffiths, 1963

Černý and Merz 2006, **HA**, Asni; Černý 2013

﻿***Melanagromyza* Hendel, 1920**

﻿*Melanagromyza ﻿﻿﻿lappae* (Loew, 1850)

Mouna 1998; **AP** (Rabat) – MISR

﻿*Melanagromyza ﻿﻿﻿verbasci* Spencer, 1957

Spencer 1967, **HA**

﻿***Ophiomyia* Braschnikov, 1897**

﻿*Ophiomyia ﻿﻿﻿beckeri* (Hendel, 1923)

Spencer 1967, **HA**; Černý and Merz 2006; Černý 2009; Černý and Tschirnhaus 2014

﻿*Ophiomyia ﻿﻿﻿curvipalpis* (Zetterstedt, 1848)

= ﻿*Ophiomyia ﻿﻿﻿proboscidea* (Strobl, 1900), in Mouna 1998: 84

Spencer 1967, **HA**; Mouna 1998; Černý and Merz 2006; Černý and Merz 2007; Černý 2019

﻿*Ophiomyia ﻿﻿﻿melandryi* de Meijere, 1924

Spencer 1967, **HA**; Černý and Merz 2006; Černý and Merz 2007; Černý 2009

﻿*Ophiomyia ﻿﻿﻿vimmeri* Černý, 1994

Černý and Merz 2006, **AP**, Maâmora (Rabat); Černý and Merz 2007; Černý 2018

##### Phytomyzinae

﻿***Amauromyza* Hendel, 1931**

﻿*Amauromyza (Amauromyza) ﻿morionella* (Zetterstedt, 1848)

= ﻿*Agromyza ﻿﻿﻿morionella* Zetterstedt, in Becker and Stein 1913: 95

﻿***Aulagromyza* Enderlein, 1936**

﻿*Aulagromyza ﻿﻿﻿atlantidis* (Spencer, 1967)

= *Paraphytomyza ﻿﻿﻿atlantidis* Spencer, 1967, in Mouna 1998: 84

Spencer 1967, **HA**, Asni; Mouna 1998

﻿*Aulagromyza ﻿﻿﻿cydoniae* (Hendel, 1936)

= *Phytagromyza ﻿﻿﻿cydoniae* Hendel, 1936, in Hendel 1931–1936: 518

Hendel 1931–1936, **AP**, Rabat

﻿*Aulagromyza ﻿﻿﻿hamata* (Hendel, 1932)*

**HA**, Asni-Ouirgane

﻿***Cerodontha* Rondani, 1861**

﻿*Cerodontha (Cerodontha) ﻿denticornis* (Panzer, 1806)

Spencer 1967, 1973, **HA**; Černý and Merz 2006; Černý 2009

﻿*Cerodontha (Cerodontha) ﻿fulvipes* (Meigen, 1830)

Mouna 1998

﻿*Cerodontha (Dizygomyza) ﻿brisiaca* Nowakowski, 1973

Černý and Merz 2006, **MA**, Azrou, Ifrane

﻿*Cerodontha (Icteromyza) capitata* (Zetterstedt, 1848)

Černý and Merz 2006, **Rif**, Chefchaouen

﻿*Cerodontha (Icteromyza) ﻿rozkosnyi* Černý, 2007

Černý 2007, **AA**, SW Tazenakht (1000 m); Černý 2011

﻿*Cerodontha (Poemyza) ﻿incisa* (Meigen, 1830)

= ﻿*Dizygomyza ﻿﻿﻿incisa* Meigen, in Séguy 1936b: 5; Mouna 1998: 84

Séguy 1936b, **AP**, Rabat; Mouna 1998

﻿*Cerodontha (Poemyza) lateralis* (Macquart, 1835)

= ﻿*Dizygomyzalateralis* Macquart, in Séguy 1936: 5; Mouna 1998: 84

Séguy 1936b, **AP**, Rabat; Mouna 1998; Černý et al. 2020

﻿*Cerodontha (Poemyza) ﻿pygmaea* (Meigen, 1830)

= ﻿*Dizygomyza ﻿﻿﻿pygmaea* Meigen, in Séguy 1936: 5; Mouna 1998: 84

Séguy 1936b, **AP**, Rabat; Mouna 1998

﻿***Chromatomyia* Hardy, 1849**

﻿*Chromatomyia ﻿﻿﻿aprilina* (Goureau, 1851)

Griffiths 1974; Spencer 1967, **Rif**, Tanger (mountains)

﻿*Chromatomyia ﻿﻿﻿horticola* (Goureau, 1851)

= ﻿*Phytomyza ﻿﻿﻿horticola* Goureau, in Griffiths 1967: 14

Griffiths 1967, **AP**, Casablanca; Spencer 1973; Černý 2009

﻿*Chromatomyia ﻿﻿﻿milii* (Kaltenbach, 1864)

= ﻿*Phytomyza ﻿﻿﻿milii* Kaltenbach, in Séguy 1936: 5; Mouna 1998: 84

Séguy 1936b, **AP**, Rabat; Spencer 1967, **HA**, Ourika Valley; Griffiths 1980; Mouna 1998; Černý and Merz 2006

﻿*Chromatomyiasyngenesiae* Hardy, 1849

= ﻿*Phytomyza ﻿atricornis* Meigen, in Kozlowsky and Rungs 1932: 66; Mouna 1998: 84

Kozlowsky and Rungs 1932, **AP**, Rabat, Kénitra, Casablanca; Mouna 1998; **AP** (Rabat) – MISR

﻿***Liriomyza* Mik, 1894**

﻿*Liriomyza ﻿﻿﻿bryoniae* (Kaltenbach, 1858)

Spencer 1967, 1973, **AP**, Casablanca; Ayoub 2002, **AA**, Souss Massa, Agadir; Černý and Merz 2006

﻿*Liriomyza ﻿cicerina* (Rondani, 1874)

Spencer 1973, **HA**; Lahmar and Zeouienne 1992; Mouna 1998

﻿*Liriomyza ﻿﻿﻿congesta* (Becker, 1903)

Černý et al. 2020, **AA**, River bed of Ougni, 0.6 km N Akka N’Ait Sidi and 1.8 km NW Tissint (582 m), River Aoulouz (= Asif Tifnout), bridge of road (697 m), River Oued Draa near hotel, Gardin Oued Tamnougalt (911 m)

﻿*Liriomyzahuidobrensis* (Blanchard, 1926)

Hanafi and Schnitzler 2004, **AA**, Souss Valley

﻿*Liriomyza ﻿﻿﻿orbona* (Meigen, 1830)

= ﻿*Agromyza ﻿﻿﻿fuscolimbata* Strobl, 1900, in Maarouf 2003: 43

= ﻿*Liriomyza ﻿﻿﻿orbonella* Hendel, 1931, in Maarouf 2003: 43

Maarouf 2003, **HA**, Chaouia

﻿*Liriomyza ﻿﻿﻿pedestris* Hendel, 1931

Spencer 1967, **Rif**, Tanger; Černý and Merz 2006; Černý and Merz 2007; Černý 2019; Černý et al. 2020

﻿*Liriomyza ﻿﻿﻿pusilla* (Meigen, 1830)

= ﻿*Agromyza ﻿﻿﻿pusilla* Meigen, in Mouna 1998: 84

Mouna 1998

﻿*Liriomyza ﻿﻿﻿sonchi* Hendel, 1931

Spencer 1967, **AP**, Casablanca

﻿*Liriomyza ﻿﻿﻿trifolii* (Burgess in Comstock, 1880)

Hanafi and Schnitzler 2004, **AA**, Souss Valley

﻿***Napomyza* Westwood, 1840**

﻿*Napomyza ﻿﻿﻿cichorii* Spencer, 1966

Černý and Merz 2006, **MA**, Ifrane; Černý 2009

﻿*Napomyzalateralis* (Fallén, 1823)

Černý and Merz 2006, **MA**, Ifrane

﻿*Napomyza ﻿﻿﻿scrophulariae* Spencer, 1966

Černý and Merz 2006, **MA**, Ifrane; Černý and Merz 2007; Černý 2012, 2013

﻿***Phytoliriomyza* Hendel, 1931**

﻿*Phytoliriomyza ﻿﻿﻿immoderata* Spencer, 1963

Černý and Merz 2006, **MA**, Azrou, Ifrane; Černý 2009; Černý 2019; Černý et al. 2020

﻿*Phytoliriomyza ﻿﻿﻿oasis* (Becker, 1907)

Spencer 1967, **HA**; Černý and Merz 2006

﻿***Phytomyza* Fallén, 1810**

﻿*Phytomyza ﻿﻿﻿conyzae* Hendel, 1920

Spencer 1967, **Rif**, Tanger; Černý 2009

﻿*Phytomyza ﻿﻿﻿ferulae* Hering, 1927

Spencer 1967, **Rif**, Tanger; Černý 2013; Černý et al. 2020

﻿*Phytomyza ﻿﻿﻿gymnostoma* Loew, 1858

Mouna 1998; **AP** (Mechra el kettane) – MISR

﻿*Phytomyza ﻿orobanchia* Kaltenbach, 1864

Geipert 1993; Geipert et al. 1994, **Rif**; Klein 1995; Klein et al. 1995; Boumezzough 1996, **MA**, Saiss, Rommani; Boughdad et al. 1997; Klein et al. 1999; Kroschel and Klein 1999; Yazough and Klein 1999; Kroschel and Klein 2003

﻿*Phytomyza ﻿﻿﻿phillyreae* Hering in Buhr, 1930

= ﻿*Phytomyza ﻿﻿﻿unedo* Séguy, 1953, in Séguy 1953b: 72

Séguy 1953b, **AP**, Korifla; Spencer 1967, **HA**, Ourika Valley; Černý and Merz 2006; Černý 2009

﻿*Phytomyza ﻿﻿﻿ranunculi* (Schrank, 1803)

Spencer 1967, **HA**; Černý and Merz 2006; Černý 2009, 2013

﻿*Phytomyza ﻿﻿﻿wahlgreni* Rydén, 1944

Černý and Merz 2006, **MA**, Azrou, Ifrane; Černý et al. 2020

﻿***Pseudonapomyza* Hendel, 1920**

﻿*Pseudonapomyza ﻿﻿﻿atra* (Meigen, 1830)

= ﻿*Phytomyza ﻿﻿﻿﻿﻿﻿acuticornis* Loew, 1858, in Maarouf 2003: 43

Maarouf 2003, **HA**, Chaouia

﻿*Pseudonapomyza ﻿﻿﻿atratula* Zlobin, 2003

Černý et al. 2020, **AA**, 6 km ESE Quijjane, 85 km S Agadir (353 m)

﻿*Pseudonapomyza ﻿﻿﻿bifida* Zlobin, 2003

Černý et al. 2020, **AA**, Road no. 109/165 from Akna to Taroudant (841 m)

﻿*Pseudonapomyza ﻿﻿﻿spicata* (Malloch, 1914)

Černý et al. 2020, **AA**, River bed of Ougni, 0.6 km N Akka N’Ait Sidi and 1.8 km NW Tissint (582 m)

﻿*Pseudonapomyza ﻿spinosa* Spencer, 1973

Maarouf 2003, **HA**, Chaouia

##### New record for Morocco

﻿*Aulagromyza ﻿﻿﻿hamata* (Hendel, 1932)

High Atlas: Asni-Ouirgane, 31°13'52"N, 8°00'8"W, 1282 m a.s.l., 1♂, 24.iv.2014, river valley, V. Vrabec leg., M. Barták coll. and M. Černý det.

#### ﻿﻿ANTHOMYZIDAE

K. Kettani, M.J. Ebejer

Number of species: **2**. Expected: 4

Faunistic knowledge of the family in Morocco: poor

﻿***Amygdalops* Lamb, 1914**

﻿*Amygdalops ﻿﻿﻿thomasseti* Lamb, 1914

Ebejer et al. 2019, **Rif**, Stehat (0 m)

﻿***Anagnota* Becker, 1902**

﻿*Anagnotamajor* Roháček & Freidberg, 1993

Roháček 2006, **HA**, Marrakech (1000 m)

#### ﻿﻿ASTEIIDAE

K. Kettani, M.J. Ebejer

Number of species: **5**. Expected: 10

Faunistic knowledge of the family in Morocco: poor

##### Asteiinae

﻿***Asteia* Meigen, 1830**

﻿*Asteia ﻿﻿﻿﻿amoena* Meigen, 1830

Mouna 1998; **AP** (Rabat) – MISR

﻿*Asteia ﻿﻿﻿ibizana* (Enderlein, 1935)

Ebejer et al. 2019, **AP**, Larache (2 m)

﻿*Asteia ﻿﻿﻿mahunkai* Papp, 1979

Ebejer et al. 2019, **AP**, Larache (2 m)

﻿***Phlebosotera* Duda, 1927**

﻿*Phlebosotera ﻿﻿﻿clypeata* Freidberg & Carles-Tolrá, 2010

Freidberg and Carles-Tolrá 2010, **HA**, Jaffar river

﻿*Phlebosotera ﻿﻿﻿mirabilis* Papp, 1972

Ebejer et al. 2019, **AA**, 12 km S of Rissani (737 m)

#### ﻿﻿AULACIGASTRIDAE

K. Kettani, M.J. Ebejer

Number of species: **1**. Expected: 2

Faunistic knowledge of the family in Morocco: poor

﻿***Aulacigaster* Macquart, 1835**

﻿*Aulacigaster ﻿﻿﻿leucopeza* (Meigen, 1830)

Ebejer et al. 2019, **Rif**, Jebel Lakraâ (Talassemtane, 1541 m)

#### ﻿﻿CLUSIIDAE

K. Kettani, M.J. Ebejer

Number of species: **1**. Expected: 3

Faunistic knowledge of the family in Morocco: poor

***Clusiodes* Coquillett, 1904**

﻿*Clusioides ﻿﻿﻿verticalis* (Collin, 1912)

Ebejer et al. 2019, **Rif**, Amsemlil bog (PNPB, 1067 m)

#### ﻿﻿ODINIIDAE

K. Kettani, M.J. Ebejer

Number of species: **2**. Expected: 3

Faunistic knowledge of the family in Morocco: poor

##### Odiniinae

﻿***Odinia* Robineau-Desvoidy, 1830**

﻿*Odinia ﻿Boletina* (Zetterstedt, 1848)

Séguy 1934a; Mouna 1998; Gaimari and Mathis 2011

﻿*Odinia ﻿﻿﻿meijerei* Collin, 1952

Ebejer et al. 2019, **Rif**, Adrou (PNPB, 556 m)

#### ﻿﻿OPOMYZIDAE

K. Kettani, M.J. Ebejer

Number of species: **5**. Expected: 6

Faunistic knowledge of the family in Morocco: moderate

﻿***Geomyza* Fallén, 1810**

﻿*Geomyza ﻿﻿﻿﻿﻿﻿﻿﻿﻿﻿apicalis* (Meigen, 1830)

Pârvu et al. 2006, **MA**, Ifrane; Popescu-Mirceni 2011, **AP**, Merja Zerga

﻿*Geomyza ﻿﻿﻿combinata* (Linnaeus, 1767)

Mouna 1998; **AP** (Maâmora) – MISR

﻿*Geomyza ﻿﻿﻿tripunctata* (Fallén, 1823)

Maarouf 2003, **HA**, Chaouia

﻿***Opomyza* Fallén, 1820**

﻿*Opomyza ﻿﻿﻿florum* (Fabricius, 1794)

Maarouf 2003, **HA**, Chaouia

﻿*Opomyza ﻿﻿﻿petrei* Mesnil, 1934

Ebejer et al. 2019, **Rif**, Aïn Tissemlal (Azilane, 1255 m)

### ﻿Carnoidea

#### ﻿﻿CANACIDAE

K. Kettani, L. Munari

Number of species: **15**. Expected: 25

Faunistic knowledge of the family in Morocco: poor

##### Canacinae

﻿***Canace* Haliday in Curtis, 1837**

﻿*Canace ﻿﻿﻿actites* Mathis, 1982

Ebejer et al. 2019, **AP**, Loukous (2 m)

﻿*Canace ﻿﻿﻿nasica* Haliday, 1839

Dahl 1964, **AA**, Tamri (north of Agadir); Mouna 1998

﻿***Xanthocanace* Hendel, 1914**

﻿*Xanthocanace ﻿﻿﻿ranula* (Loew, 1874)

Munari 2010, **AP**, 40 km S Larache; Munari and Mathis 2010; Munari 2011; Munari and Bramuzzo 2018, **Rif**, Briyech, **AP**, Azemmour, El Khaoucha, Fedala, Oued Nefifikh, Oued Loukous, 40 km S Larache

##### Tethininae

﻿***Tethina* Haliday in Curtis, 1837**

﻿*Tethina ﻿﻿﻿alboguttata* (Strobl, 1900)

Freidberg and Beschovski 1996, **AP**, Aïn Diab, Essaouira, Safi, **AA**, Agadir, Ouarzazate; Mathis and Munari 1996; Munari 2002, 2004, 2005, 2011; Munari and Mathis 2010; Koçak and Kemal 2010; Munari and Bramuzzo 2018, **Rif**, Briyech, **AP**, Agadir, Tamri, Essaouira, Cap Hadid, Larache, Loukous, Safi, Dar Caïd-Hadji

﻿*Tethina ﻿﻿﻿albosetulosa* (Strobl, 1900)

Cassar et al. 2008, **Rif**, Oued Laou Basin

﻿*Tethina ﻿﻿﻿flavigenis* (Hendel, 1934)

Munari and Bramuzzo 2018, **AP**, Oued Oum-er-Rbia

﻿*Tethina ﻿﻿﻿grossipes* (Becker, 1908)

Munari 2004, **AA**, Agadir; Munari and Mathis 2010; Munari and Bramuzzo 2018, **AP**, Tamri

﻿*Tethina ﻿﻿﻿incisuralis* (Macquart, 1851)

= ﻿*Tethina ﻿﻿﻿pictipes* (Becker 1903); Séguy 1941d: 18, Mouna 1998: 87

Munari 1997, **AA**, Erfoud, Rissani (900 m); Munari 2002, 2010, 2011; Munari and Mathis 2010; Munari and Bramuzzo 2018, **AA**, Erfoud, Rissani

﻿*Tethina ﻿﻿﻿longirostris* (Loew, 1865)

Cassar et al. 2008, **Rif**, Oued Laou Basin

﻿*Tethina ﻿mariae* Munari, 1997

Munari 1997, **AP**, 40 km S Larache; Munari and Báez 2000; Munari 2002, 2004, 2010, 2011; Munari and Mathis 2010 – NHMD (HT ♂)

﻿*Tethina ﻿﻿﻿pallipes* (Loew, 1865)

= ﻿*Tethina ﻿﻿﻿ochracea* (Hendel, 1913)

Cassar et al. 2008, **Rif**, Oued Laou

﻿*Tethina ﻿﻿﻿pictipennis* Freidberg & Beschovski, 1996

Freidberg and Beschovski 1996, **AP**, 40 km south of Larache; Mathis and Munari 1996, Munari 2002; Munari 2004; Munari and Mathis 2010; Munari 2011, **AA**, Agadir (Tamri); Munari and Bramuzzo 2018, **Rif**, Briyech, **AP**, Tamri, Larache, Loukous – NHMD (HT ♂)

﻿*Tethina ﻿﻿﻿strobliana* (Mercier, 1923)

Munari and Bramuzzo 2018, **AP**, Oued Abou, Rehouna (Rabat)

﻿*Tethina ﻿﻿﻿yaromi* Freidberg & Beschovski, 1996

Cassar et al. 2005, **Rif**, Smir lagoon

﻿Tethina﻿sp. nearsalinicola Beschovski, 1998

Munari 2004, **AA**, Agadir, Ouarzazate; Munari 2005; Munari and Bramuzzo 2018, **AP**, Tamri

#### ﻿﻿CARNIDAE

K. Kettani, M.J. Ebejer

Number of species: **3**. Expected: >10

Faunistic knowledge of the family in Morocco: poor

﻿***Meoneura* Rondani**, **1856**

﻿*Meoneura ﻿﻿﻿hungarica* Papp, 1977

Ebejer et al. 2019, **Rif**, Adrou (PNPB, 556 m)

﻿*Meoneura ﻿﻿﻿prima* Becker, 1905

Brake 2011

﻿*Meoneura ﻿﻿﻿triangularis* Collin, 1930

Brake 2011

#### ﻿﻿CHLOROPIDAE^48^

K. Kettani, M. von Tschirnhaus, M.J. Ebejer

Number of species: **74**. Expected: 140

Faunistic knowledge of the family in Morocco: moderate

##### Chloropinae

﻿***Assuania* Becker, 1903**

﻿*Assuania ﻿melanoleuca* (Séguy, 1949) **comb. nov.^49^**

= ﻿*Chloropsmelanoleuca* Séguy, in Séguy 1949a: 158

Séguy 1949a, **AA**, Agdz; Nartshuk 1984; Mouna 1998

﻿*Assuania ﻿﻿﻿thalhammeri* (Strobl, 1893)

Ebejer and Kettani 2016, **AA**, Oued Ziz (9.5 km SE of Rich, 1285 m)

﻿***Camarota* Latreille, 1805**

﻿*Camarota ﻿﻿﻿curvipennis* (Latreille, 1805)

= ﻿*Camarota ﻿﻿﻿curvipennis* (Latreille), in Becker and Stein 1913: 93

Becker and Stein 1913, **Rif**, Tanger; Nartshuk 1984; Ebejer and Kettani 2016, **Rif**, Oued Laou

﻿***Capnoptera* Loew, 1866**

﻿*Capnoptera ﻿﻿﻿pilosa* Loew, 1866

Duda 1933; Ebejer and Kettani 2016, **Rif**, Jebel Lakraâ (Talassemtane, 1288 m), Aïn Jdioui (Tahaddart, 8 m)

﻿*Capnoptera ﻿﻿﻿scutata* (Rossi, 1790)

= ﻿*Eristalis ﻿﻿﻿rufipes* Fabricius, in Fabricius 1805: 245

Ebejer and Kettani 2016, **AP**, Larache (2 m)

﻿***Cetema* Hendel, 1907**

﻿*Cetema ﻿﻿﻿maroccanum* Nartshuk, 1995

= ﻿*Cetema ﻿maroccana* Nartshuk, in Nartshuk 1995: 277–280

Nartshuk 1995, **HA**, Oukaimeden; Ebejer and Kettani 2016

﻿*Cetema ﻿﻿﻿monticulum* Becker, 1910

= ﻿*Cetema ﻿﻿﻿monticula* Becker, in Séguy 1941a: 33

= ﻿*Cetema ﻿﻿﻿monticula* Rossi, in Mouna 1998: 85

Séguy 1941a, **HA**, Tachdirt (Toubkal, 2500 m); Mouna 1998; Ebejer and Kettani 2016

﻿***Chlorops* Meigen, 1803**

﻿*Chlorops ﻿﻿﻿interruptus* Meigen, 1830

= ﻿*Chlorops ﻿﻿﻿interrupta* Meigen, in Séguy 1953a: 86

Séguy 1953a, **AP**, Oued Yquem (near Rabat); Ebejer and Kettani 2016, **Rif**, Jebel Lakraâ (Talassemtane, 1288 m), Oued Aliane (Ksar Sghir, 1 m), Cap Spartel (155 m)

﻿*Chlorops ﻿﻿﻿limbatus* Meigen, 1830

Ebejer and Kettani 2016, **Rif**, Jebel Moussa (800 m)

﻿*Chlorops ﻿﻿﻿pumilionis* (Bjerkander, 1778)

= ﻿*Chlorops ﻿﻿﻿nasuta* Schrank, in Mouna 1998: 85

Mouna 1998; Ebejer and Kettani 2016

﻿*Chlorops ﻿﻿﻿serenus* Loew, 1866

Ebejer and Kettani 2016, **MA**, 17 km NW of Zaida (Khénifra, 1878 m)

﻿***Cryptonevra* Lioy, 1864**

﻿*Cryptonevra ﻿﻿﻿flavitarsis* (Meigen, 1830)

= *Haplegis ﻿﻿﻿flavitarsis* (Meigen), in Séguy 1949a: 158; Séguy 1953a: 86; Mouna 1998: 85

Séguy 1949a, **AA**, Agdz; Séguy 1953a, **AP**, Oued Yquem (near Rabat); Nartshuk 1984; Mouna 1998; Ebejer and Kettani 2016

﻿***Eurina* Meigen, 1830**

﻿*Eurina ﻿﻿﻿lurida* Meigen, 1830

Ebejer and Kettani 2016, **Rif**, M’Diq, Smir, Kabila beach and dunes

﻿***Eutropha* Loew, 1866**

﻿*Eutropha ﻿﻿﻿albipilosa* (Becker, 1908)

Duda 1930; Deeming and Al-Dhafer 2012, **AP**, 17 km N of Larache, **AA**, Azemmour, estuary of Oued Tensift; Ebejer and Kettani 2016, **AP**, Larache (5 m)

﻿*Eutropha ﻿﻿﻿fulvifrons* (Haliday, 1833)

Séguy 1941d, **AA**, Agadir; Mouna 1998; Cassar et al. 2005, **Rif**, Smir lagoon; Ebejer and Kettani 2016, **AP**, Larache (5 m)

﻿***Lagaroceras* Becker, 1903**

﻿*Lagaroceras ﻿﻿﻿﻿﻿﻿andalusiaca* (Strobl, 1899)

Ebejer and Kettani 2016, **Rif**, Ksar El Kebir (13 m), Oued Aliane (Ksar Sghir, 1 m), **AP**, Larache (2 m), **AA**, Oued Ziz (9.5 km SE of Rich, 1285 m)

﻿***Lasiosina* Becker, 1910**

﻿*Lasiosina ﻿﻿﻿herpini* (Guérin-Méneville, 1843)

= ﻿*Lasiosina ﻿﻿﻿cinctipes* Meigen, in Mouna 1998: 85

Ebejer and Kettani 2016, **Rif**, Smir lagoon

﻿*Lasiosina ﻿﻿﻿lindbergi* (Duda, 1933)

= *Steleocerus ﻿﻿﻿lindbergi* (Duda), in Duda 1933: 142

Duda 1933, **MA**; Ebejer and Kettani 2016, **AA**, Oued Ziz (9.5 km SE of Rich, 1285 m)

﻿***Meromyza* Meigen, 1830**

﻿*Meromyza ﻿﻿﻿athletica* Fedoseeva, 1974

= ﻿*Meromyza ﻿﻿﻿variegata* Meigen, in Mouna 1998: 85

Mouna 1998; Ebejer and Kettani 2016

﻿*Meromyza ﻿﻿﻿curvinervis* (Zetterstedt, 1848)

= *Oxinis ﻿﻿﻿curvinervis* Latreille, in Mouna 1998: 85

Mouna 1998

﻿*Meromyza ﻿﻿﻿nigriventris* Macquart, 1835

Ebejer and Kettani 2016, **MA**, 17 km SW of Midelt (Khénifra, 1940 m), **AA**, 29 km N of Rich (Errachidia, 1570 m)

﻿*Meromyza ﻿﻿﻿pratorum* Meigen, 1830

Séguy 1941a, **HA**, Tachdirt (Toubkal, 2500 m); Mouna 1998; Ebejer and Kettani 2016

﻿***Metopostigma* Becker, 1903**

﻿*Metopostigma ﻿﻿﻿sabulona* Becker, 1910

Ebejer and Kettani 2016, **AA**, Oued Ziz (10 km S of Errachidia, 1008 m), Merzouga (714 m)

﻿***Platycephala* Fallén, 1820**

﻿*Platycephala ﻿﻿﻿scapularum* (Becker, 1907)

Nartshuk 1984; Pârvu et al. 2006, **AA**, Lac Edehby, Ouarzazate; Popescu-Mirceni 2011; Ebejer and Kettani 2016

﻿***Pseudopachychaeta* Strobl, 1902**

﻿*Pseudopachychaeta ﻿﻿﻿pachycera* Strobl, 1902

Ebejer and Kettani 2016, **AA**, Oued Ziz (9.5 km SE of Rich, 1285 m), Oued Ziz (1052 m)

﻿***Thaumatomyia* Zenker, 1833**

﻿*Thaumatomyia ﻿﻿﻿elongatula* (Becker, 1910)

Ebejer and Kettani 2016, **Rif**, Beni Maâdene (Oued Martil, 3 m)

﻿*Thaumatomyia ﻿﻿﻿glabra* (Meigen, 1830)

Ebejer and Kettani 2016, **Rif**, Martil (9 m)

﻿*Thaumatomyia ﻿﻿﻿notata* (Meigen, 1830)

= *Chloropisca ﻿﻿﻿notata* (Meigen), in Séguy 1930a: 179, 1941a: 33; Mouna 1998: 85

Séguy 1930a, **Rif**, Tanger; Séguy 1941a, **HA**, Tachdirt (Jebel Toubkal, 2500 m), Canyon Tessaout (M’Goum, 3000–3200 m); Harris et al. 1980: 229, **HA**, Chichaoua; Mouna 1998; Ebejer and Kettani 2016, **Rif**, Oued Laou estuary, Oued Amsa (Amsa, 14 m), Jnane Niche (27 m), Oued Maggou (Maggou, 786 m), Marj Khayl (Beni Leit (PNPB), 1088 m), Oued Boumarioul (Aïn Hamra, 560 m), Aïn Tissemlal (Azilane, 1255 m), Oued Bouhya (Bou Ahmed, 19 m), Souk Khemis Anjra (Oued Kbir, 55 m), Zinat (231 m), Oued Zarka (Yarghite, 135 m), Oued Talembote (Usine électrique, 120 m), Oued Kelâa (Akoumi, 400 m), Issaguen (maison forestière Issaguen, 1543 m), Bni Boufrah (94 m), Oued Guallet (Bni Boufrah, 946 m), Oued Tabandoute (Bni Boufrah, 540 m), Oued Taâouniya (Koudiat Ajira, 1536 m), Talankramte (Site sacré Sidi Gneiss (PNPB): 461 m), **AP**, Larache (5 m)

﻿*Thaumatomyia ﻿﻿﻿sulcifrons* (Becker, 1907)

= ﻿*Chlorops ﻿﻿﻿sulcifrons* Becker, in Séguy 1949a: 158

= *Chloropisca ﻿﻿﻿sulcifrons* Zetterstedt, in Mouna 1998: 85

Séguy 1934a; Séguy 1949a, **AA**, Foum-el-Hassan, Akka, Agdz; Nartshuk 1984; Mouna 1998; Dawah and Abdullah 2006; Ebejer and Kettani 2016

##### Oscinellinae

﻿***Aphanotrigonum* Duda, 1932**

﻿*Aphanotrigonum ﻿﻿﻿cinctellum* (Zetterstedt, 1848)

Ebejer and Kettani 2016, **AP**, Larache (5 m)

﻿*Aphanotrigonum ﻿﻿﻿femorellum* Collin, 1946

Ebejer and Kettani 2016, **AP**, Larache

﻿*Aphanotrigonum ﻿﻿﻿inerme* Collin, 1946

Ebejer and Kettani 2016, **Rif**, M’Diq (5 m), **AP**, Larache (Loukous marsh, 2 m)

﻿*Aphanotrigonum ﻿﻿﻿parahastatum* Dely-Draskovits, 1981*

**AA**

﻿***Calamoncosis* Enderlein, 1911**

﻿*Calamoncosis ﻿﻿﻿duinensis* (Strobl, 1909)

Ebejer and Kettani 2016, **AP**, Rabat (on *Phragmites*) – MISR

﻿***Conioscinella* Duda, 1929**

﻿*Conioscinella ﻿﻿﻿frontella* (Fallén, 1810)

Ebejer and Kettani 2016, **Rif**, Aïn Tissemlal (Azilane, 1255 m)

﻿*Conioscinella ﻿﻿﻿sordidella* (Zetterstedt, 1848)

Ebejer and Kettani 2016, **Rif**, Aïn Tissemlal (Azilane, 1255 m)

﻿***Elachiptera* Macquart, 1835**

﻿*Elachiptera ﻿﻿﻿bimaculata* (Loew, 1845)

De Lépiney and Mimeur 1932: 110, Rabat; Séguy 1934a: 479; Bléton and Fieuzet 1943; Nartshuk 1984; Mouna 1998; Ebejer and Kettani 2016, **Rif**, Beni Maâdene (Oued Martil: 3 m), Jnane Niche (46 m), Oued Mhannech (18 m), Souk Khemis Anjra (55 m), Boujdad (7 m); **AP** (Rabat) – MISR

﻿*Elachiptera ﻿﻿﻿cornuta* (Fallén, 1820)

Mouna 1998: 85

﻿*Elachiptera ﻿﻿﻿diastema* Collin, 1946

Ebejer and Kettani 2016, **Rif**, Jebel Lakraâ (Talassemtane, 1541 m), Aïn Tissemlal (Azilane, 1255 m), Oued Maggou (Maggou, 786 m), Oued Tiffert (Tiffert, 1230 m), Issaguen (1543 m), **AP**, Larache (5 m)

﻿*Elachiptera ﻿﻿﻿graeca* Becker, 1910

Séguy 1941, **HA**, Imi-n’Ouaka (1500 m); Nartshuk 1984; Mouna 1998

﻿*Elachiptera ﻿﻿﻿megaspis* (Loew, 1858)

Nartshuk 1984; Mouna 1998; Ebejer and Kettani 2016, **Rif**, Dardara (730 m), Aïn Jdioui (Tahaddart, 8 m), Jnane Niche (46 m), Aïn Tissemlal (Azilane, 1255 m), Oued Maggou (Maggou, 786 m); **AP** (Rabat) – MISR

﻿*Elachiptera ﻿﻿﻿orizae* Séguy, 1949*

**AA**

﻿*Elachiptera ﻿﻿﻿rufescens* (Walker, 1871)

Deeming and Al-Dhafer 2012, **AA**, 2 km N Erfoud (818 m); Ebejer and Kettani 2016, **AA**, 2 km N Erfoud (818 m)

﻿*Elachiptera ﻿﻿﻿rufifrons* Duda, 1932

Ebejer and Kettani 2016, **AP**, 9 km SE Aïn Chouk (6 m), Larache (5 m)

﻿*Elachiptera ﻿﻿﻿sarda* Nartshuk, 2009

Ebejer and Kettani 2016, **AA**, 14 km E of Rich (Errachidia, 1278 m)

﻿*Elachiptera ﻿﻿﻿scrobiculata* (Strobl, 1901)

= ﻿*Elachipteratrapezina* (Corti, 1909), in Bléton and Fieuzet 1943: 116

Bléton and Fieuzet 1943; Ebejer and Kettani 2016

﻿*Elachiptera ﻿﻿﻿strobli* (Corti, 1909)

Ebejer and Kettani 2016, **Rif**, Oued Boumarioul (Aïn Hamra, 560 m), Oued Aliane (Ksar Sghir, 1 m); Cap Spartel (155 m), Oued Sidi Ben Saâda (Laghdir, 242 m)

﻿***Epimadiza* Becker, 1910**

﻿*Epimadiza ﻿﻿﻿nigrescens* Duda, 1933^50^

= *Oscinosoma ﻿﻿﻿anthracias* Séguy, in Séguy 1949a: 158, Mouna 1998: 85

= *Oxinosoma ﻿﻿﻿anthracias* Séguy, in Mouna 1998: 85

= ”*Siphonella ﻿﻿﻿oscinina* (Fallén)”, in Nartshuk 1984: 236

Séguy 1949a, **AA**, Alnif; Sabrosky 1965: 406; Nartshuk 1984; Mouna 1998; Ebejer and Kettani 2016

﻿***Hapleginella* Duda, 1933**

﻿*Hapleginella ﻿﻿﻿laevifrons* (Loew, 1858)

El Hassani et al. 1986: 8–11, **Rif**, Nord Occidental, **MA**

﻿***Lasiochaeta* Corti, 1909**

﻿*Lasiochaeta ﻿﻿﻿pubescens* (Thalhammer, 1898)

= ﻿*Elachiptera ﻿﻿﻿pubescens* (Thalhammer), var. ﻿*rufithorax* Duda, 1932 in Duda 1932: 31

= *Melanochaeta ﻿﻿﻿pubescens* Thalh., in De Lépiney and Mimeur 1932: 110

= *Melanochaeta ﻿﻿﻿pubescens* (Thalhammer), in Mouna 1998: 85

De Lépiney and Mimeur 1932, **AP**, Rabat; Duda 1932: 31, **HA**; Nartshuk 1984; Mouna 1998; Ebejer and Kettani 2016, **Rif**, Oued Sidi Yahia Aarab (Sidi Yahia Aarab, 178 m), Beni Maâdene (Oued Martil, 3 m), Oued Mhannech (Tamuda, 18 m), Oued Amsa (Amsa, 14 m), Zinat (231 m), Oued Maggou (Maggou, 786 m), Oued Guallet (Bni Boufrah, 946 m); **AP** (Rabat) – MISR

﻿***Oscinella* Becker, 1909**

﻿*Oscinella ﻿﻿﻿cariciphila* Collin, 1946

Ebejer and Kettani 2016, **Rif**, Beni Maâdene (Oued Martil, 3 m), **AA**, Merzouga (714 m)

﻿*Oscinella ﻿﻿﻿frit* (Linnaeus, 1758)

= *Oxinosoma ﻿﻿﻿frit* Linnaeus, in Mouna 1998: 85

De Lépiney and Mimeur 1932: 109, **AP**, Rabat; Mouna 1998; Ebejer and Kettani 2016, **Rif**, Issaguen (1543 m), Aïn Tissemlal (Azilane, 1255 m), Oued Maggou (Maggou, 786 m), Oued Guallet (Bni Boufrah, 946 m), Oued Ametrasse (Ametrasse, 841 m), **AP**, Lower Loukous saltmarsh (2 m), **MA**, Khénifra (17 km SW of Midelt, 1940 m), Lac Aguelmane Afennourir (30 km SW of Azrou, 1490 m); **AP** (Rabat) – MISR

﻿*Oscinella ﻿﻿﻿nartshukiana* Beschovski, 1978

Ebejer and Kettani 2016, **Rif**, Ksar El Kebir (13 m), **AP**, Larache

﻿*Oscinella ﻿﻿﻿nitidigenis* (Becker, 1908)

Ebejer and Kettani 2016, **AA**, 6 km N of Errachidia (1010 m), Oued Ziz (1052 m)

﻿*Oscinella ﻿﻿﻿nitidissima* (Meigen, 1838)

Ebejer and Kettani 2016, **Rif**, Dardara (730 m), Moulay Abdelsalam (965 m), Cap Spartel (155 m), Oued Laou (dunes, 2 m), **AP**, Larache (5 m), **MA**, Lac Aguelmane Afennourir (30 km SW of Azrou, 1490 m)

﻿*Oscinella ﻿﻿﻿pusilla* (Meigen, 1830)

Ebejer and Kettani 2016, **AA**, Lac Tiffert (4 km W of Merzouga, 702 m)

﻿*Oscinella ﻿﻿﻿ventricosi* Nartshuk, 1955

Ebejer and Kettani 2016, **Rif**, Oued Boumarioul (Aïn Hamra, 560 m), **AP**, Larache (5 m)

﻿*Oscinella ﻿﻿﻿vindicata* (Meigen, 1830)

Ebejer and Kettani 2016, **AP**, Larache (5 m), **AA**, Merzouga (agriculture under date palms, 714 m)

﻿***Oscinimorpha* Lioy, 1864**

﻿*Oscinimorpha ﻿﻿﻿arcuata* (Duda, 1932)

Ebejer and Kettani 2016, **Rif**, Oued Guallet (Bni Boufrah, 946 m), Oued Jnane Azaghar (Bni Boufrah, 997 m)

﻿*Oscinimorpha ﻿﻿﻿longirostris* (Loew, 1858)

Ebejer and Kettani 2016, **Rif**, Ksar El Kebir (13 m)

﻿*Oscinimorpha ﻿﻿﻿minutissima* (Strobl, 1900)

= *Siphonella ﻿﻿﻿minutissima* Strobl, in Becker and Stein 1913: 93

Becker and Stein 1913, **Rif**, Tanger; Nartshuk 1984; Ebejer and Kettani 2016, **Rif**, Dardara (484 m)

﻿*Oscinimorpha ﻿﻿﻿novakii* (Strobl, 1893)

= ﻿*Conioscinella ﻿﻿﻿novakii* (Strobl), in Duda 1933: 58

Duda 1933; Ebejer and Kettani 2016

﻿***Oscinisoma* Lioy, 1864**

﻿*Oscinisoma ﻿﻿﻿cognatum* (Meigen, 1830)

= *Oscinis ﻿﻿﻿rufipes* Meigen, 1830, with homonym *Oscinis ﻿﻿﻿rufipes*Wiedemann 1830: 580, in Wiedemann 1830: 580, Tanger; synonymy and probable specific identity of homonyms established by Becker 1910: 166, but without considering the similar ﻿*Oscinisoma ﻿﻿﻿gilvipes* (Loew, 1858).

﻿***Polyodaspis* Duda, 1933**^51^

﻿*Polyodaspis ﻿﻿﻿sulcicollis* (Meigen, 1838)

= *Siphonella ﻿﻿﻿sulcicollis* (Meigen), in Kroschel and Klein 1999: 138

Kroschel and Klein 1999; Ebejer and Kettani 2016, **MA**, Khénifra (17 km SW of Midelt, 1940 m)

﻿***Pselaphia* Becker, 1911**

﻿*Pselaphia ﻿﻿﻿dimidiocera* Ebejer & Kettani, 2016

Ebejer and Kettani 2016, **Rif**, Adrou (Taghzout, 556 m) – NMWC

﻿***Rhodesiella* Adams, 1905**

﻿*Rhodesiella ﻿﻿﻿fedtshenkoi* Nartshuk, 1978

Deeming and Al-Dhafer 2012, **SA**, Goulimine (Bou Jarif); Ebejer and Kettani 2016, **Rif**, Smir lagoon, Oued Laou (saltmarsh), Jnane Niche (46 m)

﻿***Sabroskyina* Beschovski, 1987**

﻿*Sabroskyina ﻿﻿﻿aharonii* (Duda, 1933)

Ebejer and Kettani 2016, **AA**, 2 km N Erfoud (818 m)

﻿***Siphunculina* Rondani, 1856**

﻿*Siphunculina ﻿﻿﻿ornatifrons* (Loew, 1858)

= *Microneurum ﻿﻿﻿ornatifrons* (Loew), in Duda 1933: 98

Becker and Stein 1913, **Rif**, Tanger; Duda 1933; Nartshuk 1984; Ebejer and Kettani 2016, **Rif**, Martil (9 m), Oued Mhannech (Tamuda, 18 m), Jnane Niche (27 m), **AP**, Aïn Chouk 9 km SE (6 m), Larache (5 m)

﻿***Trachysiphonella* Enderlein, 1936**

﻿*Trachysiphonella ﻿﻿﻿ruficeps* (Macquart, 1835)

Ebejer and Kettani 2016, **AA**, 29 km N of Rich (Errachidia, 1570 m)

﻿***Tricimba* Lioy, 1864**

﻿*Tricimba ﻿﻿﻿cincta* (Meigen, 1830)

Ebejer and Kettani 2016, **Rif**, Dardara (484 m)

﻿*Tricimba ﻿﻿﻿heratica* Dely-Draskovits, 1983*

**AA**

﻿*Tricimba ﻿﻿﻿humeralis* (Loew, 1858)

= ﻿*Tricimba ﻿﻿﻿punctifrons* Becker, in Becker and Stein 1913: 93

= ﻿*Tricimba ﻿﻿﻿humeralis* (Loew), in Séguy 1941d: 18, 1957: 273; Mouna 1998: 85

Becker and Stein 1913, **Rif**, Tanger; Séguy 1941d, **AA**, Agadir; Séguy 1957, **AA**, Agadir; Nartshuk 1984; Mouna 1998; Ebejer and Kettani 2016, **Rif**, Oued Mhannech (Tamuda, 18 m), Aïn Tissemlal (Azilane, 1255 m), Oued Bouhya (Bou Ahmed, 19 m), **AA**, Oued Ziz (9.5 km SE of Rich, 1285 m), Oued Ziz (13 km N of Erfoud, 800 m)

##### Siphonellopsinae

﻿***Apotropina* Hendel, 1907**

﻿*Apotropina ﻿﻿﻿longepilosa* (Strobl, 1893)

Ebejer and Kettani 2016, **Rif**, Oued Sidi Ben Saâda (Laghdir, 242 m), Oued Kbir (Dardara, 345 m), Oued Siflaou (281 m), Jnane Niche (46 m)

﻿***Siphonellopsis* Strobl, 1906**

﻿*Siphonellopsis ﻿﻿﻿lacteibasis* Strobl, 1906

Séguy 1934a: 482; Nartshuk 1984; Mouna 1998

##### New records for Morocco

﻿*Aphanotrigonum ﻿﻿﻿parahastatum* Dely-Draskovits, 1981

Anti Atlas: Ougui river, 1.8 km NW Tissint, 29°55'03"N, 7°19'56"W, 582 m, 30.xii.2016, 3♂♂3♀♀, M. von Tschirnhaus det. – ZSM (M. von Tschirnhaus leg.).

﻿*Elachiptera ﻿﻿﻿orizae* Séguy, 1949

Anti Atlas: river Aoulouz up stream of Idrgane, 30°44'11"N, 7°59'13"W, 866 m, 31.xii.2016, 1♂, M. von Tschirnhaus det. – ZSM (M. von Tschirnhaus leg.).

﻿*Tricimba ﻿﻿﻿heratica* Dely-Draskovits, 1983

Anti Atlas: Oued Souss, streamup of Idrgane, 30°44'11"N, 7°59'13"W, 866 m, 31.xii.2016, 1♀ (males are needed to confirm the identification), M. von Tschirnhaus det. – ZSM (M. von Tschirnhaus leg.).

#### ﻿﻿MILICHIIDAE

K. Kettani

Number of species: **8**. Expected: 14

Faunistic knowledge of the family in Morocco: moderate

##### Madizinae

Madizini

﻿***Desmometopa* Loew, 1866**

﻿*Desmometopa ﻿﻿﻿m-﻿nigrum* (Zetterstedt, 1848)

Séguy 1949a, **SA**, Guelmim; Mouna 1998; **Rif** (M’Diq), **AP** (Larache), **HA** (Tazzarin) – MISR

﻿*Desmometopa ﻿﻿﻿varipalpis* Malloch, 1927

Ebejer et al. 2019, **AA**, 6 km N of Errachidia (1010 m)

﻿***Leptometopa* Becker, 1903**

﻿*Leptometopa ﻿﻿﻿rufifrons* Becker, 1903

Ebejer et al. 2019, **AA**, Merzouga (714 m); **AA** (Merzouga) – MISR

﻿***Madiza* Fallén, 1810**

﻿*Madiza ﻿﻿﻿glabra* Fallén, 1820

Mouna 1998; Ebejer et al. 2019, **HA**, Anafgou (NPHAO, 2271 m)

##### Milichiinae

Milichiini

﻿***Milichia* Meigen, 1830**

﻿*Milichia ﻿﻿﻿﻿albomaculata* (Strobl, 1900)

Pont and Singh 1965; Mouna 1998; Brake 2000; Koçak and Kemal 2010; **HA** – NHMUK

﻿*Milichia ﻿﻿﻿speciosa* Meigen, 1830

Séguy 1930a, **HA**, Arround (Skoutana)

﻿***Milichiella* Giglio-Tos, 1895**

﻿*Milichiella ﻿lacteipennis* (Loew, 1866)

Brake 2000; Raspi et al. 2009; **Rif** (M’Diq farm) – MISR

##### Phyllomyzinae

Phyllomyzini

﻿***Phyllomyza* Fallén, 1810**

﻿*Phyllomyza* ﻿sp. ﻿aff. ﻿*equitans* (Hendel, 1919)

Ebejer et al. 2019, **AA**, Oued Ziz (12 km S of Rissani, 737 m)

##### Acknowledgement

We gratefully acknowledge the cooperation of Martin J. Ebejer who contributed to the revision of this family.

### ﻿Sphaeroceroidea

#### ﻿﻿CHYROMYIDAE

K. Kettani, M.J. Ebejer

Number of species: **22**. Expected: 30

Faunistic knowledge of the family in Morocco: good

##### Aphaniosominae

﻿***Aphaniosoma* Becker, 1903**

﻿*Aphaniosoma ﻿﻿﻿approximatum* Becker, 1903

Ebejer 2018, **SA**, Oued Ougni (0.6 km N of Akka, N’Aït Sidi and 1.8 km NW Tissint, 582 m)

﻿*Aphaniosoma ﻿﻿﻿claridgei* Ebejer, 1995

Ebejer 2016b, **Rif**, Smir lagoon

﻿*Aphaniosoma ﻿﻿﻿collini* Lyneborg, 1973

Ebejer 2016b, **AP**, Larache (5 m)

﻿*Aphaniosomaforcipatum* Ebejer, 1998

Ebejer 2016b, **AA**, Lac de Tiffert (4 km W of Merzouga, 702 m), Erfoud (30 km N, 894 m), Ziz river (14 km E of Rich, 1278 m)

﻿*Aphaniosoma ﻿﻿﻿gatti* Ebejer, 2016

Ebejer 2016b, **AP**, El Jadida, Azemmour; **AP** (El Jadida Azemmour) – MISR

﻿*Aphaniosoma ﻿﻿﻿melitense* Ebejer, 1993

Andrade and Almeida 2010, **Rif**, Mediterranean coast; Ebejer 2016b, **AP**, Larache (2 m)

﻿*Aphaniosoma ﻿﻿﻿nigricauda* Ebejer, 1998

Ebejer 2016b, **AA**, Lac de Tiffert (4 km W of Merzouga, 702 m), Merzouga (714 m), Ziz river (10 km S of Errachidia, 1008 m; 13 km N of Erfoud, 800 m)

﻿*Aphaniosoma ﻿﻿﻿nigripes* Ebejer, 2016

Ebejer 2016b, **AA**, Ziz river (9.5 km SE of Rich, 1285 m), Erfoud (30 km N, 894 m; **AA** (Ziz river) – MISR

﻿*Aphaniosoma ﻿﻿﻿nigrum* Ebejer, 1998

Ebejer 2016b, **AA**, Ziz river (Errachidia, 1052 m; 9.5 km SE of Rich, 1285 m), Erfoud (30 km N, 894 m); **AA** (Ziz river) – MISR

﻿*Aphaniosomanitidum* Ebejer, 2016

Ebejer 2016b, **AA**, Ziz river (30 km N of Erfoud, 894 m)

﻿*Aphaniosoma ﻿﻿﻿propinquans* Collin, 1949

Ebejer 2016b, **Rif**, Smir lagoon, Oued Aliane (Ksar Sghir), **AP**, Larache (2 m); **Rif** (Oued Aliane) – MISR

﻿*Aphaniosoma ﻿﻿﻿proximum* Ebejer, 1998

Ebejer 2016b, **AP**, Larache (5 m), Sidi Smail (El Jadida), Azemmour, **HA**, Oued Tensift

﻿*Aphaniosoma ﻿﻿﻿quadrinotatum* (Becker, 1904)

Ebejer 2018, **AA**, S of village Sidi R’bat (10 m)

﻿*Aphaniosoma ﻿﻿﻿rufum* Frey, 1935

Ebejer 2018, **AA**, S of village Sidi R’bat (10 m)

﻿*Aphaniosoma ﻿﻿﻿soror* Ebejer, 2016

Ebejer 2016b, **AA**, Errachidia (14 km E of Rich, 1278 m)

﻿*Aphaniosoma ﻿﻿﻿trisetum* Ebejer, 2016

Ebejer 2016b, **AA**, Ziz river (9.5 km SE of Rich, 1285 m, 13 km N of Erfoud, 800 m, Errachidia, 1052 m), Merzouga (714 m); **AA** (Ziz river) – MISR

﻿*Aphaniosoma ﻿﻿﻿zizense* Ebejer, 2016

Ebejer 2016b, **AA**, Ziz river (Errachidia, 1052 m; 9.5 km SE of Rich, 1285 m; 10 km S of Errachidia, 1008 m); **AA** (Ziz river) – MISR

##### Chyromyinae

﻿***Chyromya* Robineau-Desvoidy, 1830**

﻿*Chyromya ﻿﻿﻿robusta* Hendel, 1931

Ebejer 2018, **AA**, S of village Sidi R’bat (10 m)

﻿***Gymnochiromyia* Hendel, 1933**

﻿*Gymnochiromyia ﻿﻿﻿flavella* (Zetterstedt, 1848)

Ebejer 2016b, **Rif**, Ksar El Kebir (13 m)

﻿*Gymnochiromyia ﻿﻿﻿homobifida* Carles-Tolrá, 2001

Ebejer 2016b, **Rif**, maison forestière de Talassemtane (NPT)

﻿*Gymnochiromyia ﻿﻿﻿inermis* (Collin, 1933)

Ebejer 1998b; Ebejer 2016b

﻿*Gymnochiromyia ﻿﻿﻿mihalyii* Soós, 1979

Ebejer 2016b, **Rif**, Oued Siflaou (281 m), Aïn Tissemlal (Azilane, 1255 m), **AP**, Larache (5 m)

﻿*Gymnochiromyia ﻿﻿﻿tschirnhausi* Ebejer, 2018

Ebejer 2018, **AA**, S of village Sidi R’bat (10 m)

﻿*Gymnochiromyia ﻿﻿﻿zernyi* (Czerny, 1929)

Ebejer 1998b; Ebejer 2016b

#### ﻿﻿HELEOMYZIDAE

K. Kettani, A.J. Woźnica

Number of species: **19**. Expected: 30

Faunistic knowledge of the family in Morocco: moderate

##### Heleomyzinae

﻿***Gymnomus* Loew, 1863**

﻿*Gymnomus ﻿﻿﻿atlasicus* Woźnica, 2011

Woźnica 2011, **HA**, Tazzeka

﻿*Gymnomus ﻿﻿﻿caesius* (Meigen, 1830)

Ebejer et al. 2019, **Rif**, Jebel Lakraâ (Talassemtane, 1541 m)

﻿***Neoleria* Malloch, 1919**

﻿*Neoleria* ﻿sp.*

**Rif**

##### Heteromyzinae

﻿***Tephrochlamys* Loew, 1862**

﻿*Tephrochlamys ﻿﻿﻿rufiventris* (Meigen, 1830)*

**AA**

##### Suilliinae

﻿***Suillia* Robineau-Desvoidy, 1830**

﻿*Suillia ﻿﻿﻿bicolor* (Zetterstedt, 1838)*

**Rif**

﻿*Suillia ﻿﻿﻿bistrigata* (Meigen, 1830)

Ebejer et al. 2019, **Rif**, Moulay Abdelsalam (Bouhachem, 1098 m), Jebel Lakraâ (Talassemtane, 1541 m), **MA**, 6 km S of Azrou (1610 m), 20 km S of Azrou (1720 m)

﻿*Suillia ﻿﻿﻿humilis* (Meigen, 1830)

= *Helomyza ﻿﻿﻿similis* Meigen, in Mouna 1998: 85

Mouna 1998; **AP** (Maâmora) – MISR

﻿*Suillia ﻿﻿﻿notata* (Meigen, 1830)

Ebejer et al. 2019, **Rif**, Moulay Abdelsalam (Bouhachem, 1180 m), Jebel Lakraâ (Talassemtane, 1541 m), Oued Kbir (Béni Ratene, 157 m)

﻿*Suillia ﻿﻿﻿oxyphora* (Mik, 1900)*

**Rif**

﻿*Suillia ﻿pallida* (Fallén, 1820)

Séguy 1941d, **HA**, Tizi-n’Test (2200 m); Mouna 1998

﻿*Suillia ﻿﻿﻿tuberiperda* (Rondani, 1876)

Ebejer et al. 2019, **MA**, 6 km S of Azrou (1610 m)

﻿*Suillia ﻿﻿﻿variegata* (Loew, 1862)

= *Helomyza ﻿﻿﻿variegata* Loew, in Becker and Stein 1913: 93

Becker and Stein 1913, **Rif**, Tanger; Séguy 1953a, **AP**, Sidi Yahia du Gharb; Mouna 1998

##### Trixoscelidinae

﻿***Trixoscelis* Rondani, 1856**

﻿*Trixoscelis ﻿﻿﻿approximata* (Loew, 1865)

Cassar et al. 2008, **Rif**, Smir lagoon; **Rif**, Tétouan

﻿*Trixoscelis ﻿﻿﻿baliogastra* (Černý, 1909)

Hackman 1970

﻿*Trixoscelis ﻿﻿﻿canescens* (Loew, 1865)

Ebejer et al. 2019, **Rif**, Aïn Tissemlal (Azilane, 1255 m)

﻿*Trixoscelis ﻿﻿﻿curvata* Carles-Tolrá, 1993

Ebejer et al. 2019, **AP**, Larache (5 m)

﻿*Trixoscelis ﻿﻿﻿laeta* (Becker, 1907)

Becker 1907; Séguy 1941d, **AA**, Agadir; Hackman 1970; Mouna 1998; Cassar et al. 2008, **Rif**, Smir lagoon

﻿*Trixoscelis ﻿﻿﻿mendizabali* Hackman, 1970

Hackman 1970, **AA**, Aït Melloul (Oued Souss)

﻿*Trixoscelis ﻿﻿﻿pedestris* (Loew, 1865)

Ebejer et al. 2019, **Rif**, Aïn Jdioui (Tahaddart, 76 m)

##### New records for Morocco

﻿*Neoleria* ﻿sp.

Rif: Tétouan, Jebel Bouhachem (PNPB), Amsemlil, 35.26234°N, 5.43341°W, 1067 m, 11.xii.15, forest (*Pinuspinaster*), K. Kettani leg. (1♀), A.J. Woźnica det.

﻿*Tephrochlamys ﻿﻿﻿rufiventris* (Meigen, 1830)

Rif: Tétouan, Jebel Bouhachem (PNPB), Remla, 35.236865, -5.408025, 961 m, 22.iv.18, forest (*Pinuspinaster*), K. Kettani leg. (1♀), A.J. Woźnica det.

Anti Atlas: Errachidia, 13 km E of Goulmima, 31°44.568N, 4°51.945W, dry stony steppe 1100 m, 3.v.2012, M.J. Ebejer leg. (1♂), A.J. Woźnica det.

﻿*Suillia ﻿﻿﻿bicolor* (Zetterstedt, 1838)

Rif: Talassemtane, Jebel Lakraâ, 35°06.913N, 5°08.034W, 1541 m, 12.vi.2013, meadow in mixed forest, M.J. Ebejer leg. (1♀); Jebel Bouhachem, Taghzout, Adrou, 556 m, 35°22.39N, 5°32.28W, 25.iv.2015, M.J. Ebejer leg. (2♀♀), A.J. Woźnica det.

﻿*Suillia﻿oxyphora* (Mik, 1900)

Rif: Jebel Talassemtane, 35°07'N, 5°07'W, 1554 m, 13.iv.2009, Fir forest (*Abiesmaroccana*), A. Taheri leg. (1♂3♀♀); Azilane, Aïn Tissemlal, 35°11.67N, 5°15.20W, 1255 m, 4.vii.13–13.viii.2013, K. Kettani leg. (1♀), Fir forest (Abiesmaroccana), A.J. Woźnica det.

#### ﻿﻿SPHAEROCERIDAE

K. Kettani, P. Gatt

Number of species: **67**. Expected: 130

Faunistic knowledge of the family in Morocco: poor

##### Copromyzinae

﻿***Borborillus* Duda, 1923**

﻿*Borborillus ﻿vitripennis* (Meigen, 1830)

Becker and Stein 1913, **Rif**, Tanger; Gatt 2006, **Rif**, Tétouan, Oued Laou; Marshall et al. 2011

﻿***Copromyza* Fallén, 1810**

﻿*Copromyza ﻿equina* Fallén, 1820

Gatt 2006, **Rif**, Tétouan, Oued Laou; Marshall et al. 2011

﻿***Crumomyia* Macquart, 1835**

﻿*Crumomyia ﻿﻿﻿glabrifrons* (Meigen, 1830)

Gatt et al. 2016, **Rif**, Tariouma (El Anasser, 1383 m), **MA**, Lac Aguelmane Sidi Ali (2050 m)

﻿***Lotophila* Lioy, 1864**

﻿*Lotophila ﻿﻿﻿atra* (Meigen, 1830)

Gatt 2006, **Rif**, Tanger; Marshall et al. 2011

﻿***Norrbomia* Papp, 1988**

﻿*Norrbomia ﻿﻿﻿hispanica* (Duda, 1923)

Gatt 2006, **Rif**, Tétouan, Oued Laou; Marshall et al. 2011

﻿*Norrbomia ﻿﻿﻿marginatis* (Adams, 1905)

Gatt 2006, **Rif**, Tétouan, Oued Laou; Marshall et al. 2011

﻿*Norrbomia ﻿﻿﻿nilotica* (Becker, 1903)

Roháček et al. 2001; Marshall et al. 2011

﻿*Norrbomia ﻿﻿﻿niveipennis* (Duda, 1923)

Roháček et al. 2001; Marshall et al. 2011

﻿*Norrbomia ﻿﻿﻿somogyii* (Papp, 1973)

Roháček et al. 2001; Marshall et al. 2011

﻿*Norrbomia ﻿﻿﻿sordida* (Zetterstedt, 1847)

Gatt et al. 2016, **Rif**, Oued Guallet (Bni Boufrah, 946 m), **AP**, Larache, Loukous Marsh

##### Limosininae

﻿***Bifronsina* Roháček, 1983**

﻿*Bifronsina ﻿﻿﻿bifrons* (Stenhammar, 1855)

Roháček et al. 2001; Marshall et al. 2011

﻿***Ceroptera* Macquart, 1835**

﻿*Ceroptera ﻿﻿﻿rufitarsis* (Meigen, 1830)

= ﻿*Limosina ﻿﻿﻿picta* Becker, in Becker and Stein 1913: 94

Becker and Stein 1913, **Rif**, Tanger; Papp 1984a; Roháček et al. 2001, **Rif**, Tanger

﻿***Chaetopodella* Duda, 1920**

﻿*Chaetopodella ﻿﻿﻿scutellaris* (Haliday, 1836)

= ﻿*Leptocera ﻿﻿﻿scutellaris* (Haliday), in Séguy 1941a: 32

Séguy 1941a, **HA**, Imi-n’Ouaka (1500 m)

﻿***Coproica* Rondani, 1861**

﻿*Coproica ﻿﻿﻿digitata* (Duda, 1918)

Gatt 2006, **Rif**, Tétouan, Oued Laou; Marshall et al. 2011

﻿*Coproica ﻿﻿﻿ferruginata* (Stenhammar, 1855)

Gatt 2006, **Rif**, Tétouan, Martil; Marshall et al. 2011

﻿*Coproica ﻿﻿﻿hirticula* Collin, 1956

Gatt et al. 2016, **Rif**, Dardara (730 m), Oued Laou (30 m), Zarka waterfall (Yarghit, 135 m), Oued Ouara (Beni Zid, 440 m), Oued Jnane Azaghar (Bni Boufrah, 997 m), Oued Mhannech (125 m), Adrou (Taghzout, 556 m), Oued Jnane Niche (Jnane Niche, 27 m)

﻿*Coproica ﻿﻿﻿hirtula* (Rondani, 1880)

Gatt et al. 2016, **Rif**, Zarka waterfall (Yarghit, 135 m), **AA**, 14 km N Errachidia (Errachidia, 1214 m)

﻿*Coproica ﻿﻿﻿lugubris* (Haliday, 1836)

Gatt 2006, **Rif**, Tétouan, M’Diq, Smir, Oued Laou; Marshall et al. 2011

﻿*Coproica ﻿﻿﻿pusio* (Zetterstedt. 1847)

Gatt et al. 2016, **Rif**, Tamrabete (Oued Laou, 203 m), Smir lagoon (5 m)

﻿*Coproica ﻿﻿﻿rohaceki* Carles-Tolrá, 1990

Gatt et al. 2016, **Rif**, Oued Amsa (Amsa, 14 m), Oued Moulay Bouchta (Dar Akobaâ, 285 m)

﻿*Coproica ﻿﻿﻿rufifrons* Hayashi, 1991

Gatt et al. 2016, **Rif**, Issaguen (1543 m)

﻿*Coproica ﻿﻿﻿vagans* (Haliday, 1833)

Gatt et al. 2016, **AA**, 14 km N Errachidia (1214 m), **AA**, Oued Ziz (13 km N Erfoud, 800 m), **SA**, Merzouga (698 m)

*Eulimosina ﻿﻿﻿ochripes* (Meigen, 1830)

Gatt et al. 2016, **Rif**, Issaguen (1543 m), Ksar el Kebir (20 m), **MA**, Lac Aguelamane Afenourrir (30 km SW Azrou, 1760 m)

﻿***Leptocera* Olivier, 1813**

﻿*Leptocera ﻿﻿﻿caenosa* (Rondani, 1880)

Gatt et al. 2016, **Rif**, Dardara (484 m)

﻿*Leptocera ﻿﻿﻿fontinalis* (Fallén, 1826)

Gatt 2006, **Rif**, Tétouan, M’Diq, Smir; Marshall et al. 2011

﻿*Leptocera ﻿﻿﻿nigra* Olivier, 1813

Munari 1993, **MA**, Azrou; Roháček et al. 2001; Marshall et al. 2011

﻿***Limosina* Macquart, 1835**

﻿*Limosina ﻿﻿﻿silvatica* (Meigen, 1830)

Gatt et al. 2016, **Rif**, Issaguen (1543 m), Talassemtane (NPT, 1696 m), **MA**, 3,5 km S Azrou (1450 m), 17 km NW Zaida (Khénifra, 1878 m)

﻿***Minilimosina* Roháček, 1983**

﻿*Minilimosina (Svarciella) vitripennis* (Zetterstedt, 1847)

Gatt et al. 2016, **MA**, 3,5 km S Azrou (1450 m)

﻿***Opacifrons* Duda, 1918**

﻿*Opacifrons ﻿﻿﻿coxata* (Stenhammar, 1855)

Gatt 2006, **Rif**, Tétouan, M’Diq, Smir, Oued Laou; Marshall et al. 2011

﻿*Opacifrons ﻿﻿﻿maculifrons* (Becker, 1907)

Gatt 2006, **Rif**, Tétouan, Oued Laou; Marshall et al. 2011

﻿***Opalimosina* Roháček, 1983**

﻿*Opalimosina (Opalimosina) ﻿mirabilis* (Collin, 1902)

Gatt 2006, **Rif**, Tétouan, Oued Laou; Marshall et al. 2011

﻿***Paralimosina* Papp, 1973**

﻿*Paralimosina ﻿﻿﻿fucata* (Rondani, 1880)

Gatt et al. 2016, **Rif**, 5 km W Dardara (730 m), Dardara (484 m), Oued Azla (Mokdassen Oulya, 186 m), Oued Talembote (Talembote, 320 m), Oued El Khizana (El Khizana, 980 m), Jebel Kelaâ (Talassemtane, 1554 m), Adrou (Taghzout, 556 m)

﻿***Phthitia* Enderlein, 1938**

﻿*Phthitia (Kimosina) ﻿sicana* (Munari, 1988)

Gatt 2006, **Rif**, Tétouan, Oued Laou; Marshall et al. 2011

﻿*Phthitia (Kimosina) ﻿ciliata* (Duda, 1918)

Gatt et al. 2016, **SA**, Bou Jarif (Goulimine)

﻿*Phthitia (Kimosina) ﻿plumosula* (Rondani, 1880)

Gatt et al. 2016, **Rif**, Jebel Lakraâ (Talassemtane, 1541 m), Jebel Kelaâ (Talassemtane, 1554 m), Zaouiet and Habtiyène (Maggou, 1213 m), Oued Arozane (Bni Moussa, 317 m), Issaguen (1543 m), Zarka waterfall (Yarghit, 135 m), Taghramt (Bine El Ouidane, 276 m)

﻿*Phthitia (Kimosina) ﻿pteremoides* (Papp, 1973)

Gatt et al. 2016, **SA**, Bou Jarif (Goulimine)

﻿***Poecilosomella* Duda, 1925**

﻿*Poecilosomella ﻿﻿﻿﻿angulata* (Thomson, 1869)

Gatt 2006, **Rif**, Tétouan, Martil; Marshall et al. 2011

﻿***Pseudocollinella* Duda, 1924**

﻿*Pseudocollinella ﻿﻿﻿jorlii* (Carles-Tolrá, 1990)

Munari 1993, **MA**, Aguelmane; Roháček et al. 2001; Marshall et al. 2011

﻿***Pullimosina* Roháček, 1983**

﻿*Pullimosina (Pullimosina) ﻿heteroneura* (Haliday, 1836)

Gatt 2006, **Rif**, Tétouan, Oued Laou; Marshall et al. 2011

﻿*Pullimosina (Pullimosina) ﻿zayensis* Marshall, 1986

Roháček et al. 2001; Marshall et al. 2011

﻿***Puncticorpus* Duda, 1918**

﻿*Puncticorpus ﻿﻿﻿lusitanicum* (Richards, 1963)

Papp 1984a; Roháček et al. 2001

﻿***Rachispoda* Lioy, 1864**

﻿*Rachispoda ﻿﻿﻿acrosticalis* (Becker, 1903)

Gatt 2006, **Rif**, Tétouan, M’Diq, Smir; Marshall et al. 2011

﻿*Rachispoda ﻿﻿﻿brevior* (Roháček, 1991)

Roháček 1991, **AP**, Oued Bou-Regreg; Munari 1993; Roháček et al. 2001; Marshall et al. 2011

﻿*Rachispoda ﻿﻿﻿duodecimseta* (Papp, 1973)

Roháček 1991, **EM**, Guercif-Oued Moulouya; Munari 1993; Roháček et al. 2001; Marshall et al. 2011

﻿*Rachispoda ﻿﻿﻿fuscipennis* (Haliday, 1833)

Roháček 1991, **EM**, Oued Bou-Regreg; Munari 1993; Roháček et al. 2001; Marshall et al. 2011

﻿*Rachispoda ﻿﻿﻿gel* (Papp, 1978)

Gatt 2006, **Rif**, Tétouan, M’Diq, Smir; Marshall et al. 2011

﻿*Rachispoda ﻿﻿﻿kabuli* (Papp, 1978)

Roháček 1991, **MA**, Taza, Oued Fès; Munari 1993; Roháček et al. 2001; Marshall et al. 2011

﻿*Rachispoda ﻿﻿﻿lagura* (Roháček, 1991)

Gatt et al. 2016, **Rif**, Ksar el Kebir (20 m), **AA**, Oued Ziz (12 km S Rissani, 737 m), Oued Ziz (13 km N Erfoud, 800 m), Oued Ziz (30 km N Erfoud, 894 m)

﻿*Rachispoda ﻿﻿﻿lutosoidea* (Duda, 1938)

Roháček 1991, **MA**, Azrou, Aguelmane, Oued Sebou; Munari 1993; Roháček et al. 2001; Marshall et al. 2011

﻿*Rachispoda ﻿﻿﻿modesta* (Duda, 1924)

Gatt 2006, **Rif**, Tétouan, M’Diq, Smir; Marshall et al. 2011

﻿*Rachispoda ﻿﻿﻿uniseta* (Roháček, 1991)

Roháček 1991, **MA**, Taza, Oued Fès; Munari 1993; Roháček et al. 2001; Marshall et al. 2011; Vaňhara and Rozkošný 1997

﻿*Rachispoda ﻿﻿﻿varicornis* (Strobl, 1900)

Roháček 1991, **AP**, Oued Bou-Regreg, **MA**, Azrou; Munari 1993, **MA**, Oued Sebou; Roháček et al. 2001; Marshall et al. 2011

﻿***Spelobia* Spuler, 1924**

﻿*Spelobiabaezi* (Papp, 1977)

Roháček et al. 2001; Marshall et al. 2011

﻿*Spelobia ﻿﻿﻿clunipes* (Meigen, 1830)

Gatt et al. 2016, **Rif**, 5 km W Dardara (730 m), Martil (beach and dunes), Oued Mhannech (18 m), Oued Afertane (Afertane, 56 m), Maggou waterfall (Maggou, 786 m), Onsar Akboul (NPB, 1315 m), Oued Ametrasse (Ametrasse, 841 m), Oued Tahaddart (Tahaddart, 87 m), Issaguen (1543 m), Lemtahane (PNPB, 1088 m)

﻿*Spelobiahungarica* (Villeneuve, 1917)

Gatt 2006, **Rif**, Tétouan, Martil; Marshall et al. 2011

﻿*Spelobiaquaesita* Roháček, 1983

Roháček et al. 2001; Marshall et al. 2011

﻿***Spinilimosina* Roháček, 1983**

﻿*Spinilimosina ﻿﻿﻿brevicostata* (Duda, 1918)

Roháček et al. 2001; Marshall et al. 2011

﻿***Telomerina* Roháček, 1983**

﻿*Telomerina ﻿﻿﻿pseudoleucoptera* (Duda, 1924)

Gatt et al. 2016, **Rif**, Maggou waterfall (Maggou, 786 m)

﻿***Thoracochaeta* Duda, 1918**

﻿*Thoracochaeta ﻿﻿﻿brachystoma* (Stenhammar, 1855)

Gatt 2006; Cassar et al. 2008, **Rif**, Tétouan, M’Diq, Smir; Marshall et al. 2011

﻿*Thoracochaeta ﻿﻿﻿erectiseta* Carles-Tolrá, 1994

Gatt 2006, **Rif**, Tétouan, M’Diq, Smir, Oued Laou; Marshall et al. 2011

﻿***Trachyopella* Duda, 1918**

﻿*Trachyopella (Trachyopella) ﻿coprina* (Duda, 1918)

Gatt et al. 2016, **Rif**, Oued Jnane Niche (Jnane Niche, 27 m)

﻿*Trachyopella (Trachyopella) ﻿melania* (Haliday, 1836)

Gatt et al. 2016, **Rif**, Oued Nwawel (Azla, 57 m), M’Diq (5 m)

##### Sphaerocerinae

﻿***Ischiolepta* Lioy, 1864**

﻿*Ischiolepta ﻿﻿﻿pusilla* (Fallén, 1820)

Gatt et al. 2016, **Rif**, Oued Guallet (Bni Boufrah, 946 m), Aïn Tissemlal (Azilane, 1255 m)

﻿*Ischiolepta ﻿﻿﻿vaporariorum* (Haliday, 1836)

Gatt 2006, **Rif**, Tétouan, Oued Laou; Marshall et al. 2011

﻿***Lotobia* Lioy, 1864**

﻿*Lotobia ﻿﻿﻿﻿﻿﻿africana* (Becker, 1907)

Roháček et al. 2001; Marshall et al. 2011

﻿*Lotobia ﻿﻿﻿pallidiventris* (Meigen, 1830)

Gatt 2006, **Rif**, Tétouan, M’Diq, Oued Laou; Marshall et al. 2011

﻿***Sphaerocera* Latreille, 1804**

﻿*Sphaerocera ﻿﻿﻿curvipes* Latreille, 1805

Roháček et al. 2001; Marshall et al. 2011

### ﻿Ephydroidea

#### ﻿﻿BRAULIDAE

K. Kettani

Number of species: **1**. Expected: 1

Faunistic knowledge of the family in Morocco: good

##### Braulinae

﻿***Braula* Nitzsch, 1818**

﻿*Braula ﻿coeca* Nitzsch, 1818

Séguy 1930a; Mouna 1998; Howard et al. 2000

#### ﻿﻿CAMILLIDAE

K. Kettani, M.J. Ebejer

Number of species: **4**. Expected: 5

Faunistic knowledge of the family in Morocco: poor

﻿***Camilla* Haliday in Curtis, 1837**

﻿*Camilla ﻿﻿﻿﻿acutipennis* (Loew, 1865)

Ebejer et al. 2019, **Rif**, Aïn Jdioui (Tahaddart, 76 m), Chrabkha pond (Al Manzla, 58 m)

﻿*Camilla ﻿﻿﻿flavicauda* Duda, 1922

Mouna 1998; Pârvu et al. 2006, **AP**, Cap Sim; Popescu-Mirceni 2011

﻿*Camilla ﻿﻿﻿glabra* (Fallén, 1823)

Séguy 1941d, **HA**, Tizi-n’Test; Mouna 1998

﻿*Camilla ﻿﻿﻿pruinosa* Duda, 1934

Ebejer et al. 2019, **AP**, Larache (5 m)

#### ﻿﻿CRYPTOCHETIDAE

K. Kettani, E.P. Nartshuk

Number of species: **1**. Expected: 2

Faunistic knowledge of the family in Morocco: poor

﻿***Cryptochetum* Rondani, 1875**

﻿*Cryptochetum* (as ﻿*Cryptochaetum) mimeuri* Séguy, 1953

Séguy 1953c, **MA**, Ifrane; Nartshuk 1979, **MA**, Ifrane

#### ﻿﻿DIASTATIDAE

K. Kettani, M.J. Ebejer

Number of species: **2**. Expected: 3

Faunistic knowledge of the family in Morocco: poor

##### Campichoetinae

﻿***Campichoeta* Macquart, 1835**

﻿*Campichoeta ﻿﻿﻿obscuripennis* (Meigen, 1830)

Ebejer et al. 2019, **HA**, Lalla Takrkoust (628 m)

##### Diastatinae

﻿***Diastata* Meigen, 1830**

﻿*Diastata ﻿﻿﻿adusta* Meigen, 1830

= ﻿*Diastata ﻿﻿﻿﻿unipunctata* Zetterstedt, 1847

Pârvu et al. 2006, **AP**, Merja Zerga; Popescu-Mirceni 2011

#### ﻿﻿DROSOPHILIDAE

K. Kettani, G. Bächli

Number of species: **26**. Expected: 30

Faunistic knowledge of the family in Morocco: good

##### Drosophilinae

Drosophilini

﻿***Drosophila* Fallén, 1823**

﻿*Drosophila (Drosophila) ﻿busckii* Coquillett, 1901

= ﻿*Drosophila ﻿﻿﻿rubrostriata* Becker, 1908, in Séguy 1953a: 85

Séguy 1953a, **AP**, Rabat, Sidi Yahia du Gharb; Prevosti 1974, **HA**, Asni; Mouna 1998; Chakir et al. 2011, **HA**, Marrakech – MISR

﻿*Drosophila (Drosophila) ﻿buzzatii* Patterson & Wheeler, 1942

Prevosti 1974, **HA**, Asni (Admin forest); Mouna 1998; Bächli 2015 (TaxoDros); **Rif** (Tanger) – ZSM

﻿*Drosophila (Drosophila) ﻿funebris* (Fabricius, 1787)

Prevosti 1974, **HA**, Asni; Mouna 1998; **AP** (Maâmora) – MISR

﻿*Drosophila (Drosophila) hydei* Sturtevant, 1921

Staiger and Gloor 1952, **AP**, Rabat; Gloor and Satiger 1954, **AP**, Rabat; Prevosti 1974, **HA**, Asni (Admin forest); Mouna 1998; Chakir et al. 2011, **HA**, Marrakech

﻿*Drosophila (Drosophila) immigrans* Sturtevant, 1921

Mouna 1998; Chakir et al. 2011, **HA**, Marrakech; Bächli 2015 (TaxoDros); **Rif** (Tanger) – ZSM

﻿*Drosophila (Drosophila) ﻿kuntzei* Duda, 1924

Mouna 1998; Prevosti 1974, **AP**, Essaouira, **HA**, Asni

﻿*Drosophila (Drosophila) ﻿mercatorum* Patterson & Wheeler, 1942

Prevosti 1974, **AA**, Agadir (Admin forest near Agadir); Mouna 1998

﻿*Drosophila (Drosophila) ﻿phalerata* Meigen, 1830

Prevosti 1974, **AP**, Essaouira, **HA**, Asni; Mouna 1998; Bächli 2015 (TaxoDros); **Rif** (Tanger), **MA** (Ifrane) – ZSM

﻿*Drosophila (Drosophila) ﻿repleta* Wollaston, 1858

Séguy 1953a, **AP**, Rabat, **MA**, Fès

﻿*Drosophila (Drosophila) ﻿tsigana* Burla & Gloor, 1952

Suwito et al. 2014, **MA**, Ifrane

﻿*Drosophila (Sophophora*) ﻿﻿﻿*ambigua* Pomini, 1940

Mouna 1998; Bächli 2015 (TaxoDros); **Rif** (Tanger), **MA** (Ifrane) – ZSM

﻿*Drosophila (Sophophora) melanogaster* Meigen, 1830

Medioni 1958; David and Bocquet 1973, **HA**, Marrakech, **AA**, Agadir, Ouarzazate, Taroudant, **AA**, Zagora; Prevosti 1974, **AP**, Essaouira, **HA**, Asni (Admin forest); Jousset and Plus 1975; Plus et al. 1975, **HA**, Marrakech, **AA**, Agadir, Ouarzazate, Taroudant, **AA**, Zagora; Prevosti et al. 1975, **Rif**, Tanger; Allemand and David 1976, **AA**, Ouarzazate; Ashburner and Lemeunier 1976, **HA**, Marrakech, **AA**, Agadir, Taroudant, **AA**, Zagora; Fleuriet 1976, **AA**, Zagora; Plus et al. 1976, **AA**, Ouarzazate, Zagora; Jousset 1976, **AA**, Ouarzazate; Plus and Scotti 1984, **AA**, Ouarzazate; Thomas-Orillard 1984, **AA**, Ouarzazate; Afonso et al. 1985, **HA**, Asni; Prevosti et al. 1985, **AP**, Essaouira, **AA**, Agadir; David et al. 1986, **AP**, Rabat, Casablanca; Ayala et al. 1989, **Rif**, Chefchaouen; Boulétreau et al. 1992, **AA**, Agadir; Costa et al. 1992, **AP**, Casablanca; Capy et al. 1993, **AP**, Casablanca, Rabat; Ritchie et al. 1994, **AP**, Casablanca; Mouna 1998; Bonnivard and Higuet 1999, **AA**, Agadir; Chakir et al. 2002, 2007, 2008, 2011, **HA**, Marrakech; Ayrinhac et al. 2004, **HA**, Marrakech; Catania et al. 2004, **HA**, Marrakech **AA**, Agadir; Rohmer et al. 2004, **HA**, Marrakech; Dieringer et al. 2005, **HA**, Marrakech, **AA**, Agadir; Yassin and Orgogozo 2013, **HA**, Marrakech; Bächli 2015 (TaxoDros) – HNHM, **AP** (Rabat), **HA** (Marrakech) –MISR, **MA** (Ifrane) – ZSM

﻿*Drosophila (Sophophora) simulans* Sturtevant, 1919

Prevosti 1974, **AP**, Essaouira, **HA**, Asni (Admin forest); Baba-Aissa et al. 1988, **AP**, Rabat; Nigro 1988, **AP**, Larache; Capy et al. 1990, 1992, 1993, **AP**, Rabat, **MA**, Béni Mellal **AA**, Agadir; Chakrani et al. 1993, **AA**, Agadir; Mouna 1998; Charlat et al. 2003, **AA**, Agadir; Biémont et al. 2003, **HA**, Marrakech; Chakir et al. 2002, 2007, 2008, 2011, **HA**, Marrakech; Nardon et al. 2005, **HA**, Marrakech; Yassin and Orgogozo 2013, **HA**, Marrakech; Bächli 2015 (TaxoDros); **AA** (Agadir) – ZSM

﻿*Drosophila (Sophophora) subobscura* Collin, 1936

Götz 1965, **Rif**, Tanger; Prevosti 1971a, **Rif**, Tanger; Prevosti 1971b, **AP**, Essaouira, **HA**, Asni, **AA**, Ait-Melloul; Prevosti 1971c; Gonzalez-Duarte et al. 1973, **AP**, Essaouira, **HA**, Asni **AA**, Agadir; Prevosti 1974, **AP**, Essaouira, **HA**, Asni (Admin forest); Prevosti et al. 1975, **AA**, Agadir; Duarte 1976, **AP**, Essaouira, **HA**, Asni **AA**, Agadir; Gonzalez 1976, **AP**, Essaouira, **HA**, Asni, **AA**, Agadir; Prevosti 1978, **Rif**, Tanger, **AP**, Essaouira, **HA**, Asni **AA**, Agadir; Krimbas and Loukas 1980, **AA**, Agadir; Cabrera et al. 1983, **Rif**, Chefchaouen; Larruga et al. 1983, **Rif**, Chefchaouen; Prevosti et al. 1985, **AA**, Agadir; Pascual et al. 1986, **Rif**, Chefchaouen; Latorre et al. 1986, **Rif**, Chefchaouen; Constanti et al. 1986, **Rif**, Chefchaouen; Ayala et al. 1989, **Rif**, Chefchaouen; Afonso et al. 1990, **Rif**, Chefchaouen; Pascual et al. 1990, **Rif**, Chefchaouen; Paricio et al. 1991, **Rif**, Chefchaouen; Menozzi and Krimbas 1992, **AA**, Agadir; Latorre et al. 1992, **Rif**, Chefchaouen; Fain et al. 1993, **AA**, Agadir; Alberola and Frutos 1993; Alberola and Frutos 1996; Pinto et al. 1997, **HA**, Asni, Marrakech **AA**, Agadir; Mouna 1998; David et al. 2003, **HA**, Marrakech; Brehm et al. 2004, **AA**, Agadir; Nardon et al. 2005, **HA**, Marrakech; Bächli 2015 (TaxoDros); **MA** (Azrou) – NHMD

﻿*Drosophila (Sophophora) suzukii* (Matsumura, 1931)

Landolt et al. 2012, **Rif**, north-eastern Morocco

﻿***Hirtodrosophila* Duda, 1924**

﻿*Hirtodrosophila ﻿﻿﻿cameraria* (Haliday, 1833)

Ebejer et al. 2019, **Rif**, Aïn Ras el Ma, ruisseau maison forestière (Talassemtane)

﻿***Lordiphosa* Basden, 1961**

﻿*Lordiphosa ﻿﻿﻿﻿﻿﻿andalusiaca* (Strobl, 1906)

= ﻿*Lordiphosa ﻿﻿﻿forcipata* (Collin, 1952), in Hackman 1960: 102

Hackman 1960; Bächli 2015 (TaxoDros); **AP** (Rabat) – NHMD

﻿***Scaptomyza* Hardy, 1849**

﻿*Scaptomyza ﻿﻿﻿﻿adusta* (Loew, 1862)

Ebejer et al. 2019, **Rif**, Dardara (730 m), **AP**, Loukous marsh (2 m)

﻿*Scaptomyza ﻿﻿﻿flava* (Fallén, 1823)

= ﻿*Scaptomyza ﻿﻿﻿﻿flaveola* (Meigen, 1830), in Kozlowsky and Rungs 1932: 66

Kozlowsky and Rungs 1932, **AP**, Rabat

﻿*Scaptomyza ﻿﻿﻿graminum* (Fallén, 1823)

Maarouf 2003, **HA**, Chaouia; Bächli 2015 (TaxoDros); **HA** (Asni, Tinerhir) – NHMD

﻿*Scaptomyza (Parascaptomyza) pallida* (Zetterstedt, 1847)

Ibn Jilali 1988 (agricultural areas); Maarouf 2003, **HA**, Chaouia; Bächli 2015 (TaxoDros); **AP** (Essaouira) MHNNR, **MA** (Azrou) – NHMD

﻿***Scaptodrosophila* Duda, 1923**

﻿*Scaptodrosophila ﻿﻿﻿rufifrons* (Loew, 1873)

Mouna 1998; Bächli 2015 (TaxoDros); **Rif** (Tanger) – ZSM

﻿***Zaprionus* Coquillett, 1901**

﻿*Zaprionus ﻿﻿﻿indianus* Gupta, 1970

Yassin and David 2010

##### Steganinae

Gitonini

﻿***Gitona* Meigen, 1830**

﻿*Gitona ﻿﻿﻿microchaeta* Séguy, 1941

Séguy 1941d, **AA**, Agadir; Bächli 1982, **AA**, Agadir; Bächli and Rocha Pité 1984, **AA**, Agadir; Mouna 1998

﻿***Phortica* Fallén, 1823**

﻿*Phortica ﻿﻿﻿variegata* (Fallén, 1823)

Ebejer et al. 2019, **Rif**, Bab Berred (1433 m), Jebel Lakraâ (Talassemtane, 1541 m)

##### Steganini

﻿***Leucophenga* Mik, 1886**

﻿*Leucophenga ﻿﻿﻿maculata* (Dufour, 1839)

Séguy 1934b, **AP**, Port-Liautey (Maâmora)

#### ﻿﻿EPHYDRIDAE

K. Kettani, T. Zatwarnicki

Number of species: **117**. Expected: 140

Faunistic knowledge of the family in Morocco: good

##### Discomyzinae

Discomyzini

﻿***Actocetor* Becker, 1903**

﻿*Actocetor ﻿﻿﻿indicus* (Wiedemann, 1824)

= ﻿*Actocetor ﻿﻿﻿margaritatus* (Wiedemann, 1830), in Séguy 1934b: 162, 1953a: 86

Séguy 1934b, **Rif**, Béni Aross; Séguy 1953a, **SA**, Tindouf

﻿***Discomyza* Meigen, 1830**

﻿*Discomyza ﻿﻿﻿incurva* (Fallén, 1823)

= ﻿*Discomyzaitalica* Séguy, 1929, in Vitte 1991: 3; Dakki 1997: 63

Cresson 1939; Vitte 1991, **AP**, Atlantic coast and Plains; Dakki 1997

##### Psilopini

﻿***Clanoneurum* Becker, 1903**

﻿*Clanoneurum ﻿﻿﻿cimiciforme* (Haliday, 1855)

Séguy 1941d, **AA**, Agadir; Dahl 1964; Vitte 1991, **AP**, Rabat; Dakki 1997

﻿***Diasemocera* Bezzi, 1895**

﻿*Diasemocera ﻿﻿﻿aequalipes* (Becker, 1907)

= ﻿*Psilopa ﻿﻿﻿aequalipes* (Becker, 1907), in Ebejer et al. 2019: 147

Zatwarnicki 2018; Ebejer et al. 2019, **AA**, Lac Tiffert (4 km W of Merzouga, 702 m), Ziz river (13 km N of Erfoud, 800 m)

﻿*Diasemocera ﻿﻿﻿biskrae* (Becker, 1907)

= ﻿*Psilopa ﻿﻿﻿biskrae* (Becker, 1907), in Vitte 1991: 32

Vitte 1991, **AP**, M’Diq; Dakki 1997

﻿*Diasemocera ﻿﻿﻿composita* (Becker, 1903)

= ﻿*Psilopa ﻿﻿﻿composita* (Becker, 1903), in Vitte 1991: 32, Dakki 1997: 63

Vitte 1991, **AP**, Rabat; Dakki 1997

﻿*Diasemocera ﻿﻿﻿fratella* (Becker, 1903)

= ﻿*Psilopa ﻿﻿﻿fratella* (Becker, 1903), in Ebejer et al. 2019: 147

Ebejer et al. 2019, **AA**, Errachidia (1 km N of Tarda, 1023 m), **AA**, Ziz river (13 km N of Erfoud, 800 m)

﻿*Diasemocera ﻿﻿﻿glabricula* (Fallén, 1813)

= ﻿*Psilopa ﻿﻿﻿nigritella* Stenhammar, 1844, in Vitte 1988: 394; Dakki 1997: 63

Vitte 1988, **MA**, lakes of Middle Atlas; Vitte 1991, **Rif**; Dakki 1997

﻿*Diasemocera ﻿﻿﻿leucostoma* (Meigen, 1830)

= ﻿*Psilopa ﻿﻿﻿leucostoma* (Meigen, 1830), in Séguy 1941d: 18

Séguy 1941d, **AA**, Agadir

﻿*Diasemocera ﻿﻿﻿maritima* (Perris, 1847)

= ﻿*Psilopa ﻿﻿﻿maritima* (Perris, 1847), in Cassar et al. 2008: 25

Cassar et al. 2008, **Rif**, Laou Basin

﻿*Diasemocera ﻿﻿﻿nana* (Loew, 1860)

= ﻿*Psilopa ﻿﻿﻿nana* Loew, 1860, in Vitte 1991: 32, Dakki 1997: 63

Vitte 1991, **Rif**, **AP**, Atlantic coast; Dakki 1997

﻿*Diasemocera ﻿﻿﻿rufithorax* (Becker, 1903)

= ﻿*Psilopa ﻿﻿﻿rufithorax* (Becker, 1903), in Ebejer et al. 2019: 147

Ebejer et al. 2019, **AA**, Merzouga (714 m)

﻿***Psilopa* Fallén, 1823**

﻿*Psilopa ﻿﻿﻿clara* (Wollaston, 1858)

= ﻿*Psilopa ﻿﻿﻿rutilans* Canzoneri & Meneghini, 1972, in Cassar et al. 2005: 69

Cassar et al. 2005, **Rif**, Smir lagoon; Zatwarnicki 2018, **AP**, Larache (Lower Loukous), Safi, **AA**, Sidi Moussa, D’Agion (0–50 m)

﻿*Psilopa ﻿﻿﻿meneghinii* Canzoneri, 1986

Vitte 1991, **AP**, Atlantic coast; Dakki 1997

﻿*Psilopacompta* (Meigen, 1830)

Vitte 1988, **MA**, lakes of Middle Atlas; Vitte 1991; Dakki 1997

﻿*Psilopa ﻿﻿﻿nilotica* (Becker, 1903)

Ebejer et al. 2019, **AA**, Ziz river (10 km S of Errachidia, 1008 m), 1 km N of Tarda (Errachidia, 1023 m), Merzouga (714 m), 2 km N Erfoud (818 m)

﻿*Psilopa ﻿﻿﻿nitidula* (Fallén, 1813)

Becker and Stein 1913, **Rif**, Tanger; Séguy 1941a, **HA**, Toubkal; Vitte 1988, **MA**, lakes of Middle Atlas; Vitte 1991, **Rif**; Zatwarnicki 1991, **Rif**, Tanger, Tétouan, **MA**, Ifrane; Dakki 1997

﻿*Psilopa ﻿﻿﻿obscuripes* Loew, 1860

Ebejer et al. 2019, **Rif**, Oued Azla (near bridge, 80 m), **AP**, Larache (5 m), Lower Loukous saltmarsh (2 m), **MA**, Khénifra (28 km S of Timahdit, 2100 m)

﻿*Psilopa ﻿﻿﻿polita* (Macquart, 1835)

Vitte 1988, **MA**, lakes of Middle Atlas; Vitte 1991, **Rif**; Dakki 1997

##### Risini

﻿***Achaetorisa* Papp, 1980**

﻿*Achaetorisa ﻿﻿﻿brevicornis* Papp, 1980

Papp 1980, **HA**, Ouirgane

##### Ephydrinae

Dagini

﻿***Brachydeutera* Leow, 1862**

﻿*Brachydeutera ﻿meridionalis* (Rondani, 1856)

= ﻿*Brachydeutera ﻿﻿﻿ibari* Ninomyia, 1929, in Ebejer et al. 2019: 147

Ebejer et al. 2019, **Rif**, Oued Martil (Taboula, 14 m)

##### ﻿Parydrini

***Parydra* Stenhammar, 1844**

﻿*Parydra (Chaetoapnaea) ﻿fossarum* (Haliday, 1833)

Séguy 1930a, **AP**, Rabat, **MA**, Meknès, **HA**, Marrakech; Vitte 1991; Dakki 1997; Vitte 1988, **MA**, lakes of Middle Atlas; **MA** (Jebel Khazzane) – MISR

﻿*Parydra (Chaetoapnaea) ﻿hecate* (Haliday, 1833)

= *Napaea ﻿﻿﻿hecate* (Haliday), in Vaillant 1956b: 244

Vaillant 1956b, **HA**, Imi-N’Ifri

﻿*Parydra (Chaetoapnaea) ﻿quadripunctata* (Meigen, 1830)

Pârvu et al. 2006, **AP**, Merja Zerga; Pârvu and Zaharia 2007; Popescu-Mirceni 2011, **AP**, Merja Zerga

﻿*Parydra (Paranapaea) ﻿pubera* Loew, 1860

Dahl 1964, **AA**, Aït Melloul, Oued Souss; Vitte 1988, **MA**, lakes of Middle Atlas; Vitte 1991; Dakki 1997

﻿*Parydra (Parydra) ﻿aquila* (Fallén, 1813)

Vitte 1991, **Rif**, Ouezzane, Ketama; Dakki 1997

﻿*Parydra (Parydra) coarctata* (Fallén, 1813)

Vitte 1988, **MA**, lakes of Middle Atlas; Vitte 1991, **Rif**, Ksar el Kbir; Dakki 1997

﻿*Parydra (Parydra) ﻿cognata* Loew, 1860

Vitte 1988, **MA**, lakes of Middle Atlas; Vitte 1991; Dakki 1997

﻿*Parydra (Parydra) ﻿flavitarsis* Dahl, 1964

Vitte 1988, **Rif**, **MA**, lakes of Middle Atlas; Vitte 1991, **Rif**, **MA**, Fès; Dakki 1997; Gatt and Ebejer 2003; Dakki et al. 2003; Chillasse and Dakki 2004, **Rif**, **MA**; Dakki and Himmi 2008, **MA**, Oued Sebou

﻿*Parydra (Parydra) ﻿littoralis* (Meigen, 1830)

Vitte 1988, **MA**, lakes of Middle Atlas; Vitte 1991, **Rif**, Tétouan, Ketama; Dakki 1997

﻿*Parydra (Parydra) nigritarsis* Strobl, 1893

Vitte 1988, **MA**, lakes of Middle Atlas; Vitte 1991, **Rif**, Ketama; Dakki 1997

﻿*Parydra (Parydra) ﻿nubecula* Becker, 1896

Pârvu et al. 2006, **AA**, Lac Edehby, Ouarzazate; Popescu-Mirceni 2011, **AP**, Merja Zerga

﻿*Parydra (Parydra) ﻿quinquemaculata* Becker, 1896

Vitte 1991, **AP**, Moulay Bousselham; Dakki 1997

##### Ephydrini

﻿***Ephydra* Fallén, 1823**

﻿*Ephydra ﻿﻿﻿bivittata* Loew, 1860

Vitte 1991; Dakki 1997

﻿*Ephydraflavipes* (Macquart, 1843)

Vitte 1991, **AP**, Atlantic coast; Dakki 1997

﻿*Ephydra ﻿﻿﻿glauca* Meigen, 1830

Vitte 1991; Dakki 1997

﻿*Ephydra ﻿﻿﻿macellaria* Egger, 1862

Dahl 1964, **AA**, Oued Souss; Vitte 1988, **MA**, lakes of Middle Atlas; Dakki 1997; Vitte 1991, **AP**, Atlantic coast

﻿***Halmopota* Haliday, 1856**

﻿*Halmopotamediterranea* Loew, 1860

Dahl 1964, **AA**, Aït Melloul; Dakki 1997; Vitte 1991, **Rif**, Asilah, Chefchaouen

﻿***Paracoenia* Cresson, 1935**

﻿*Paracoenia ﻿﻿﻿fumosa* (Stenhammar, 1844)

Vitte 1988, **MA**, lakes of Middle Atlas; Dakki 1997

﻿***Setacera* Cresson, 1930**

﻿*Setacera ﻿﻿﻿breviventris* (Loew, 1860)

Vitte 1991, **Rif**, Ksar el Kbir; Dakki 1997

##### Scatellini

﻿***Haloscatella* Mathis, 1979**

﻿*Haloscatella ﻿﻿﻿dichaeta* (Loew, 1860)

= *﻿Scatella ﻿dichaeta* Loew, 1860, in Vitte 1988: 392, 1991: 26; Dakki 1997: 62

Vitte 1988, **MA**, Khemisset, Oued Beth, Dayat Aoua; Vitte 1991, **AP**, Moulay Bousselham; Dakki 1997

﻿***Lamproscatella* Hendel, 1917**

﻿*Lamproscatella ﻿﻿﻿sibilans* (Haliday, 1833)

Ebejer et al. 2019, **AA**, 1 km N of Tarda (Errachidia, 1023 m)

﻿***Limnellia* Malloch, 1925**

﻿*Limnellia ﻿﻿﻿quadrata* (Fallén, 1813)

Vitte 1991, **Rif**, Central Rif; Dakki 1997

﻿***Philotelma* Becker, 1896**

﻿*Philotelma ﻿﻿﻿nigripenne* (Meigen, 1830)

= ﻿*Scatella ﻿﻿﻿nigripennis* (Meigen, 1830), in Vitte 1988: 391; Dakki 1997: 62

Vitte 1988, **MA**, lakes of Middle Atlas; Dakki 1997

﻿***Scatella* Robineau-Desvoidy, 1830**

﻿*Scatella (Neoscatella) ﻿subguttata* (Meigen, 1830)

Vitte 1991, **AP**, Atlantic and Mediterranean coast, Smir lagoon; Dakki 1997; Pârvu et al. 2006, **AP**, Merja Zerga; Popescu-Mirceni 2011

﻿*Scatella (Scatella) ﻿ciliata* Collin, 1930

Vitte 1991, **AP**, Moulay Bousselham, Asilah; Dakki 1997

﻿*Scatella (Scatella) ﻿lacustris* (Meigen, 1830)

= ﻿*Scatella (Scatella) ﻿tenuicosta* Collin, 1930, in Ebejer et al. 2019: 147

Ebejer et al. 2019, **AA**, Ziz river (13 km N of Erfoud, 800 m)

﻿*Scatella (Scatella) ﻿lutosa* (Haliday, 1833)

Vitte 1991, **AP**, Moulay Bousselham; Dakki 1997

﻿*Scatella (Scatella) ﻿obsoleta* Loew, 1861

= ﻿*Scatella ﻿﻿﻿callosicosta* Bezzi, 1895, in Vitte 1988: 392, 1991: 26; Dakki 1997: 62

Vitte 1988, **MA**, lakes of Middle Atlas; Vitte 1991, **Rif**, M’Diq; Dakki 1997

﻿*Scatella (Scatella) ﻿paludum* (Meigen, 1830)

Dahl 1964, **AP**, Oued Korifla; Vitte 1988, **MA**, lakes of Middle Atlas; Vitte 1991; Dakki 1997

﻿*Scatella (Scatella) ﻿rufipes* Strobl, 1906

= ﻿*Scatella ﻿﻿﻿rubida* Becker, 1907, in Olafsson 1991: 21; Vitte 1991: 26; Dakki 1997: 62

Olafsson 1991, **EM**, Figuig, Defilia; Vitte 1991, **Rif**, **AP** (Atlantic coast); Dakki 1997; Gatt and Ebejer 2003

﻿*Scatella (Scatella) ﻿stagnalis* (Fallén, 1813)

Séguy 1941a, **HA**, Toubkal; Dahl 1964, **AA**, Aït Melloul, Oued Souss; Vitte 1988, **MA**, lakes of Middle Atlas; Vitte 1991; Dakki 1997

﻿***Scatophila* Becker, 1896**

﻿*Scatophila ﻿﻿﻿caviceps* (Stenhammar, 1844)

Vitte 1988, **AP**, Rabat, Temara, **MA**, lakes of Middle Atlas; Vitte 1991; Dakki 1997

﻿*Scatophila ﻿﻿﻿despecta* (Haliday, 1839)

Vitte 1988, **MA**, lakes of Middle Atlas; Vitte 1991, **MA**, Khemisset, Oued Beth; Dakki 1997

﻿*Scatophilafarinae* Becker, 1903

Zatwarnicki 1987, **HA**, Vallée de l’Ait Mizane; Vitte 1988, **MA**, lakes of Middle Atlas; Vitte 1991, **MA**, Taounate; Dakki 1997; Gatt and Ebejer 2003

﻿*Scatophila ﻿﻿﻿modesta* Becker, 1908

Vitte 1991, **Rif**, Tétouan; Dakki 1997

﻿*Scatophila ﻿﻿﻿unicornis* Czerny, 1900

Ebejer et al. 2019, **AA**, 14 km E of Rich (Errachidia, 1278 m)

##### Gymnomyzinae

Discocerinini

﻿***Diclasiopa* Hendel, 1917**

﻿*Diclasiopa ﻿﻿﻿galactoptera* (Becker, 1903)

= ﻿*Discocerina ﻿﻿﻿galactoptera* Becker, in Vitte 1988: 394; Dakki 1997: 63

Vitte 1988, **MA**, lakes of Middle Atlas; Dakki 1997; Kirk-Spriggs and McGregor 2009

﻿*Diclasiopalacteipennis* (Loew, 1862)

= ﻿*Discocerinalacteipennis* Loew, 1862, in Vitte 1988: 394, 1991: 32; Dakki 1997: 63

Vitte 1988, **MA**, lakes of Middle Atlas; Rabat; Vitte 1991, **MA**, Khemisset, Taounate; Dakki 1997

﻿*Diclasiopa ﻿﻿﻿niveipennis* (Becker, 1896)

= ﻿*Discocerina ﻿﻿﻿niveipennis* (Becker, 1896), in Vitte 1988: 394, 1991: 32; Dakki 1997: 63

Vitte 1988, **MA**, lakes of Middle Atlas; Vitte 1991, **AP**, Moulay Bousselham; Dakki 1997

﻿***Discocerina* Macquart, 1835**

﻿*Discocerina ﻿﻿﻿obscurella* (Fallén, 1813)

Vitte 1988, **AP**, **MA**, lakes of Middle Atlas; Vitte 1991, **MA**, Fès, Taounate; Mathis 1997; Dakki 1997; Wolff et al. 2016

﻿***Ditrichophora* Cresson, 1924**

﻿*Ditrichophora ﻿﻿﻿calceata* (Meigen, 1830)

= ﻿*Discocerina ﻿﻿﻿calceata* (Meigen, 1830), in Vitte 1988: 394; Dakki 1997: 63

Vitte 1988, **MA**, lakes of Middle Atlas; Dakki 1997

﻿*Ditrichophora ﻿﻿﻿mauritanica* (Vitte, 1991)

= ﻿*Discocerina ﻿﻿﻿mauritanica* Vitte, 1991, in Vitte 1991: 33

Vitte 1991, **Rif**, **MA**, Azrou; Dakki 1997; Chillasse and Dakki 2004, **Rif**, **MA**; Dakki and Himmi 2008, **MA**, Oued Sebou

﻿***Gymnoclasiopa* Hendel, 1930**

﻿*Gymnoclasiopa ﻿﻿﻿plumosa* (Fallén, 1823)

= ﻿*Discocerina ﻿﻿﻿plumosa* (Fallén, 1823), in Vitte 1988: 394, 1991: 33; Dakki 1997: 63

Vitte 1988, **MA**, lakes of Middle Atlas; Vitte 1991, **Rif**; Dakki 1997

﻿*Gymnoclasiopa ﻿﻿﻿pulchella* (Meigen, 1830)

= ﻿*Discocerina ﻿﻿﻿pulchella* (Meigen, 1830), in Vitte 1991: 33; Dakki 1997: 63

Vitte 1991, **Rif**, Ketama, Ouezzane; Dakki 1997

﻿***Hecamedoides* Hendel, 1917**

﻿*Hecamedoides ﻿﻿﻿glaucellus* (Stenhammar, 1844)

= ﻿*Discocerina ﻿﻿﻿glaucella* (Stenhammar, 1844), in Vitte 1991: 33

Vitte 1991, **Rif**, **AP**

﻿***Polytrichophora* Cresson, 1924**

﻿*Polytrichophora ﻿﻿﻿duplosetosa* (Becker, 1896)

**AP** (Rabat) – MISR

##### Gymnomyzini

﻿***Athyroglossa* Loew, 1860**

﻿*Athyroglossa (Athyroglossa) ﻿glabra* (Meigen, 1830)

Vitte 1988, **Rif**, **MA**, lakes of Middle Atlas; Dakki 1997

﻿*Athyroglossa (Athyroglossa) ﻿nudiuscula* Loew, 1860

Vitte 1991, **Rif**; Dakki 1997

﻿*Athyroglossa (Parathyroglossa) ﻿ordinata* Becker, 1896

Vitte 1991, **Rif**; Vitte 1988, **MA**, lakes of Middle Atlas; Mathis and Zatwarnicki 1990, **MA**, Ifrane; Dakki 1997

﻿***Chlorichaeta* Becker, 1922**

﻿*Chlorichaeta ﻿﻿﻿albipennis* (Loew, 1848)

Vitte 1991; Dakki 1997

﻿***Mosillus* Latreille, 1804**

﻿*Mosillus ﻿﻿﻿subsultans* (Fabricius, 1794)

= *Gymnopa ﻿﻿﻿subsultans* Fabricius, in Séguy 1930a: 181

Séguy 1930a, **MA**, M’Rirt, **HA**, Imminen (Tachidirt); Vitte 1988, **MA**, lakes of Middle Atlas; Vitte 1991, **Rif**; Mathis et al. 1993, **MA**, Ifrane (1650 m), maison forestière (cedar forest: 2700 m), Oued Jaffar (N of source, 0–1500 m), **HA**, Mikdane (Jebel Ayachi); Dakki 1997; Koçak and Kemal 2010

##### Hecamedini

﻿***Allotrichoma* Becker, 1896**

﻿*Allotrichoma ﻿﻿﻿laterale* (Loew, 1860)

Vitte 1988, **MA**, lakes of Middle Atlas; Vitte 1991; Dakki 1997

﻿*Allotrichoma ﻿﻿﻿leotoni* Vitte, 1992

Vitte 1992, **Rif**, Ouezzane, Boured

﻿*Allotrichoma ﻿﻿﻿quadripectinatum* (Becker, 1896)

= ﻿*Allotrichoma ﻿﻿﻿bellicosum* Giordani Soika, 1956, in Vitte 1991: 30; Dakki 1997: 63

Vitte 1991, **Rif**, **AP**; Dakki 1997

﻿*Allotrichoma ﻿﻿﻿simplex* (Loew, 1861)

= ﻿*Allotrichoma ﻿﻿﻿filiforme* Becker, 1896, in Vitte 1988: 393, 1991: 30; Dakki 1997: 63

Vitte 1988, **MA**, lakes of Middle Atlas; Vitte 1991, **MA**, Khemisset, Oued Sebou; Dakki 1997

﻿***Elephantinosoma* Becker, 1903**

﻿*Elephantinosoma ﻿﻿﻿chnumi* Becker, 1903

Gatt and Ebejer 2003; Kirk-Spriggs and McGregor 2009

﻿***Hecamede* Haliday, 1837**

﻿*Hecamede ﻿﻿﻿albicans* (Meigen, 1830)

Vitte 1991, **AP**; Dakki 1997; Pârvu et al. 2006, **AP**, Merja Zerga; Cassar et al. 2008, **Rif**, Smir Lagoon; Popescu-Mirceni 2011

##### Lipochaetini

﻿***Glenanthe* Haliday, 1839**

﻿*Glenanthe ﻿﻿﻿ripicola* (Haliday, 1839)

Vitte 1991, **AP**; Dakki 1997; Cassar et al. 2008, **Rif**, Laou Basin; Zatwarnicki and Mathis 2011, **AA**, Tarfaya – HNHM

﻿***Homalometopus* Becker, 1903**

﻿*Homalometopus* ﻿sp.

Vitte 1991

##### Ochtherini

﻿***Ochthera* Latreille, 1802**

﻿*Ochthera ﻿﻿﻿manicata* (Fabricius, 1794)

Vitte 1988, **MA**, lakes of Middle Atlas; Vitte 1991; Dakki 1997

﻿*Ochthera ﻿﻿﻿pilimana* Becker, 1903

Dakki 1997

﻿*Ochthera ﻿﻿﻿schembrii* Rondani, 1847

= ﻿*Ochthera ﻿﻿﻿mantispa* Loew, 1847, in Vitte 1988: 394, 1991: 28; Dakki 1997: 63

Vitte 1988, **MA**, lakes of Middle Atlas; Vitte 1991, **Rif**, **AP**; Dakki 1997

##### Hydrelliinae

Atissini

﻿***Asmeringa* Becker, 1903**

﻿*Asmeringa ﻿﻿﻿inermis* Becker, 1903

Vitte 1991, **AP**, Rabat; Dakki 1997; Gatt and Ebejer 2003

﻿***Atissa* Haliday, 1839**

﻿*Atissa ﻿﻿﻿durrenbergensis* Loew, 1864

Vitte 1991, **AP**; Dakki 1997

﻿*Atissa ﻿﻿﻿hepaticoloris* Becker, 1903

Vitte 1991, **AP**; Dakki 1997; Gatt and Ebejer 2003

﻿*Atissalimosina* Becker, 1896

Vitte 1991, **AP**, Rabat, **MA**, Fès; Dakki 1997

﻿*Atissa ﻿﻿﻿pygmaea* (Haliday, 1839)

Vitte 1988, **MA**, lakes of Middle Atlas; Vitte 1991; Dakki 1997; Pârvu et al. 2006, **AA**, Lac Edehby, Ouarzazate; Popescu-Mirceni 2011

﻿***Ptilomyia* Coquillett, 1900**

﻿*Ptilomyia ﻿﻿﻿angustigenis* (Becker, 1926)

= ﻿*Atissa ﻿﻿﻿angustigenis* Becker, in Vitte 1988: 393, 1991: 30; Dakki 1997: 63

Vitte 1988, **MA**, lakes of Middle Atlas; Vitte 1991; Dakki 1997; Gatt and Ebejer 2003

##### Dryxini

﻿***Dryxo* Robineau-Desvoidy, 1830**

﻿*Dryxo ﻿﻿﻿ornata* (Macquart, 1843)

Mathis and Zatwarnicki 2002, **AA**, Aoulouz

##### Hydrelliini

﻿***Hydrellia* Robineau-Desvoidy, 1830**

﻿*Hydrellia ﻿﻿﻿﻿﻿﻿albifrons* (Fallén, 1813)

Vitte 1991, **AP**, **Rif**; Dakki 1997

﻿*Hydrellia ﻿﻿﻿argyrogenis* Becker, 1896

Vitte 1988, **MA**, lakes of Middle Atlas; Vitte 1991, **AP**, Atlantic coast and Plains; Dakki 1997

﻿*Hydrellia ﻿﻿﻿armata* Canzoneri & Meneghini, 1976

Vitte 1991, **Rif**, Ksar el Kbir, **MA**, Fès; Dakki 1997; Dakki and Himmi 2008, **MA**, Oued Sebou

﻿*Hydrellia ﻿﻿﻿atlas* Vitte, 1989

Vitte 1989, **MA**, Dayat Aoua; Dakki 1997; Dakki et al. 2003; Chilasse and Dakki 2004, **MA**

﻿*Hydrellia ﻿﻿﻿griseola* (Fallén, 1813)

Vitte 1988, **MA**, lakes of Middle Atlas; Vitte 1991; Dakki 1997; Wolff et al. 2016; **Rif** (Oued Laou) – MISR

﻿*Hydrellia ﻿﻿﻿maculiventris* Becker, 1896

Vitte 1991, **Rif**, **AP**, Atlantic coast; Dakki 1997; Gatt and Ebejer 2003

﻿*Hydrellia ﻿﻿﻿maura* Meigen, 1838

= ﻿*Hydrellia ﻿﻿﻿modesta* Loew, 1860, in Vitte 1988: 392, 1991: 29; Dakki 1997: 63

Vitte 1988, **MA**, lakes of Middle Atlas; Zatwarnicki 1988, **MA**, Ifrane, **HA**, Vallée de l’Aït Mizane; Vitte 1991; Dakki 1997

﻿*Hydrellia ﻿﻿﻿nigricans* (Stenhammar, 1844)

Vitte 1988, **MA**, lakes of Middle Atlas; Vitte 1991, **Rif**; Dakki 1997

﻿*Hydrellia ﻿﻿﻿obscura* (Meigen, 1830)

Vitte 1988, **MA**, lakes of Middle Atlas; Vitte 1991, **Rif**; Dakki 1997; **Rif** (Aïn Jdioui) – MISR

﻿*Hydrellia ﻿﻿﻿pubescen*s Becker, 1926

= ﻿*Hydrellia ﻿﻿﻿nasturtii* Collin, 1928, in Vitte 1991: 29; Dakki 1997: 63

Vitte 1991, **MA**, Fès; Dakki 1997; Gatt and Ebejer 2003

﻿*Hydrellia ﻿﻿﻿ranunculi* (Haliday, 1838)

Vitte 1991, **AP**, Moulay Bousselham; Dakki 1997

﻿*Hydrellia ﻿﻿﻿rharbia* Vitte, 1991

Vitte 1989, **AP**, Merja Halloufa (near Moulay Bousselham); Dakki 1997; Dakki and Himmi 2008, **MA**, Oued Sebou

﻿*Hydrellia ﻿﻿﻿subalbiceps* Collin, 1966

Vitte 1991, **Rif**, Ketama; Vitte 1988, **MA**, lakes of Middle Atlas; Dakki 1997

##### Notiphilini

﻿***Notiphila* Fallén, 1810**

﻿*Notiphila (Notiphila) annulipes* Stenhammar, 1844

Vitte 1988, **MA**, lakes of Middle Atlas; Vitte 1991, **Rif**; Dakki 1997

﻿*Notiphila (Notiphila) ﻿cinerea* Fallén, 1830

Séguy 1930a, **MA**, Meknès; Séguy 1941a, **HA**, Imi-n’Ouaka; Vitte 1988, **MA**, lakes of Middle Atlas; Vitte 1991; Dakki 1997; Pârvu et al. 2006, **AP**, Merja Zerga; Kirk-Spriggs and McGregor 2009; **Rif** (Talassemtane, Aïn Jdioui) – MISR

﻿*Notiphila (Notiphila) ﻿cogani* Canzoneri & Meneghini, 1979

Vitte 1988, **MA**, lakes of Middle Atlas; Krivosheina 1998; Vitte 1991; Dakki 1997; **Rif** (Aïn Jdioui) – MISR

﻿*Notiphila (Notiphila) ﻿dorsata* Stenhammar, 1844

Vitte 1988, **MA**, lakes of Middle Atlas; Vitte 1991, **AP**, coastal lake areas; Dakki 1997

﻿*Notiphila (Notiphila) ﻿maculata* Stenhammar, 1844

Vitte 1991, **Rif**, **AP**, coastal plains; Vitte 1988, **MA**, lakes of Middle Atlas; Dakki 1997

﻿*Notiphila (Notiphila) ﻿riparia* Meigen, 1830

Krivosheina 1998, **AA**, Aït Melloul (Souss); Vitte 1988, **MA**, lakes of Middle Atlas and reedbeds; Vitte 1991; Dakki 1997

﻿*Notiphila (Notiphila) ﻿stagnicola* (Robineau-Desvoidy, 1830)

Vitte 1988, **MA**, lakes of Middle Atlas; Vitte 1991, **Rif**, **AP**, coastal plains; Dakki 1997; Pârvu et al. 2006, **AA**, Lac Edehby, Ouarzazate; Popescu-Mirceni 2011, **AA**, Ouarzazate

##### Ilytheinae

Hyadinini

﻿***Hyadina* Haliday, 1837**

﻿*Hyadina ﻿﻿﻿guttata* (Fallén, 1813)

Vitte 1988, **MA**, lakes of Middle Atlas; Vitte 1991; Dakki 1997

﻿*Hyadina ﻿﻿﻿pollinosa* Oldenberg, 1923

Vitte 1991, **MA**, Fès; Dakki 1997

﻿*Hyadina ﻿﻿﻿rufipes* (Meigen, 1830)

= ﻿*Hyadina ﻿﻿﻿nitida* (Macquart, 1835), in Vitte 1991: 27; Dakki 1997: 63

Vitte 1991, **AP**, Moulay Bousselham; Dakki 1997

﻿***Nostima* Coquillett, 1900**

﻿*Nostima ﻿﻿﻿picta* (Fallén, 1813)

Vitte 1991, **AP**; Vitte 1988, **MA**, lakes of Middle Atlas; Dakki 1997

﻿***Pelina* Haliday, 1837**

﻿*Pelina ﻿﻿﻿﻿﻿aenea* (Fallén, 1813)

Vitte 1991, **Rif**, Ouezzane; Dakki 1997

﻿*Pelina ﻿﻿﻿subpunctata* Becker, 1896

Ebejer et al. 2019, **Rif**, Jebel Lakraâ (Talassemtane, 1541 m)

﻿***Philygria* Stenhammar, 1844**

﻿*Philygria ﻿﻿﻿posticata* (Meigen, 1830)

Ebejer et al. 2019, **MA**, Khénifra (17 km SW of Midelt, 1940 m)

##### Acknowledgements

We gratefully acknowledge the assistance and cooperation of Martin J. Ebejer who contributed to the revision of this family.

### ﻿Hippoboscoidea

#### ﻿﻿HIPPOBOSCIDAE

K. Kettani, B. Droz

Number of species: **17**. Expected: 25

Faunistic knowledge of the family in Morocco: moderate

##### Hippoboscinae

Hippoboscini

﻿***Crataerina* von Olfers, 1816**

﻿*Crataerina ﻿﻿﻿﻿acutipennis* Austen, 1926

Mouna 1998: 85

﻿*Crataerina ﻿pallida* (Latreille, 1811)

Maa 1969; Mouna 1998; **AA** (Tiznit) – MISR

﻿***Hippobosca* Linnaeus, 1758**

﻿*Hippobosca ﻿﻿﻿camelina* Leach, 1817

= ﻿*Hippobosca ﻿﻿﻿dromedarina* Speiser, in Séguy 1930a: 184

Séguy 1930a; Bequaert 1939, **AP**, Rabat, **EM**, Taourit, **HA**; Séguy 1953a, **AA**, Zegdou, **SA**, Oued Agouidir; Mouna 1998; **EM** (Oued el Maa) – MISR

﻿*Hippobosca ﻿﻿﻿equina* (Linnaeus, 1758)

Séguy 1930a, **AP**, Rabat, Settat, Mogador; Séguy 1953a, **AA**, Inzegane; Maa 1969; Bequaert 1939, **MA**, Aguelmane, **HA**, Ijoukak – MISR

﻿*Hippobosca ﻿﻿﻿fulva* Austen, 1912

Bequaert 1939, **EM**, Taourirt (Ebner), Tendrara (Ebner); Maa 1963

﻿*Hippobosca ﻿﻿﻿longipennis* Fabricius, 1805

= ﻿*Hippobosca ﻿﻿﻿capensis* Olfers, in Bequaert 1939: 78

Séguy 1930a, **MA**, Meknès; Bequaert 1939, **HA**, Marrakech; Maa 1963, 1969; Mouna 1998; **AA** (Agdz) – MISR

﻿*Hippobosca ﻿﻿﻿variegata* Megerle, 1803

= ﻿*Hippobosca ﻿﻿﻿maculata* Leach, in Séguy 1930a: 184

Séguy 1930a; Moussiaux and Desmecht 2008, **HA** (south)

﻿***Icosta* Speiser, 1905**

﻿*Icostaminor* (Bigot, 1858)

= *Lynchia ﻿minor* Bigot, in Mouna 1998: 85

Báez 1978; Mouna 1998

﻿***Ornithoica* Rondani, 1878**

﻿*Ornithoica ﻿﻿﻿turdi* (Olivier in Latreille, 1811)

Maa 1966, **EM**, Figuig; Maa 1969; Mouna 1998; Droz and Haenni 2011

﻿***Ornithomyia* Latreille, 1802**

﻿*Ornithomyia ﻿﻿﻿avicularia* (Linnaeus, 1758)

Séguy 1930a; Mouna 1998

﻿*Ornithomyia ﻿﻿﻿fringillina* (Curtis, 1836)

Séguy 1930a, **MA**, Meknès; Mouna 1998

﻿***Ornithophila* Rondani, 1879**

﻿*Ornithophila ﻿﻿﻿gestroi* (Rondani, 1878)

= *Ornitheza ﻿﻿﻿gestroi* Rondani, in Mouna 1998: 85

Mouna 1998; Pape and Thompson 2019

﻿*Ornithophila ﻿﻿﻿metallica* (Schiner, 1864)

= *Ornitheza ﻿﻿﻿metallica* Schiner

Mouna 1998: 85

﻿***Pseudolynchia* Bequaert, 1926**

﻿*Pseudolynchia ﻿﻿﻿canariensis* (Macquart, 1839)

= ﻿*Pseudolynchia ﻿﻿﻿maura* Bigot, in Séguy 1930a: 184

Séguy 1930a; Mouna 1998

﻿***Stenepteryx* Leach, 1817**

﻿*Stenepteryxhirundinis* (Linnaeus, 1758)

= ﻿*Crataerina ﻿hirundinis* Linnaeus, in Mouna 1998: 85

Summer 1978, **MA**, Midelt; Mouna 1998

##### Lipopteninae

﻿***Lipoptena* Nitzsch, 1818**

﻿*Lipoptenacapreoli* Rondani, 1878

Mouna 1998: 85

﻿***Melophagus* Latreille, 1802**

﻿*Melophagus ﻿﻿﻿ovinus* (Linnaeus, 1758)

= *Melanophagus ﻿﻿﻿ovinus* Linnaeus, in Mouna 1998: 85

Séguy 1930a; Maa 1969; Mouna 1998

#### ﻿﻿NYCTERIBIIDAE

K. Kettani, G. Graciolli

Number of species: **8**. Expected: 18

Faunistic knowledge of the family in Morocco: poor

##### Nycteribiinae

﻿***Basilia* Miranda-Ribeiro, 1903**

﻿*Basilia ﻿﻿﻿italica* Theodor, 1954

Aellen 1955

﻿***Nycteribia* Latreille, 1796**

﻿*Nycteribia (Acrocholidia) ﻿vexata* Westwood, 1835

Aellen 1952; Aellen 1955, **AP**, Grotte de Sidi Bou Knadel (hosts: *Myotisoxygnathus* Monticelli, 1885, *Miniopterusshreibersii* (Kuhl, 1817) and *Rhinolophusmehelyi* (Matschie, 1901)), **MA**, Grotte de Ras el Oued (host: *Myotisoxygnathus* and *Miniopterusshreibersii*; Aellen 1963; Theodor 1967, **MA**, Oued Mellah; Mouna 1998

﻿*Nycteribia (Nycteribia) ﻿latreillei* (Leach, 1817)

Séguy 1930a, **Rif**, Samsa (Tétouan); Aellen 1952; Aellen 1955, **AP**, Grotte de Sidi Bou Knadel (host: *Miniopterusshreibersii* (Kuhl, 1817)), **MA**, Grotte de Ras el Oued (hosts: *Myotisoxygnathus* Monticelli, 1885 and *Miniopterusshreibersii*); Aellen 1963; Theodor 1967, **AP**, Mazagan (host: *Myotismyotis* (Bourhausen, 1797)); Mouna 1998

﻿*Nycteribia (Nycteribia) ﻿pedicularia* Latreille, 1805

= *Listropodia ﻿﻿﻿pedicularia* Latreille, in Séguy 1930a: 185

Falcoz 1924, **Rif**, Caverne d’Hercule; Séguy 1930a, **Rif**, Caverne d’Hercule; Mouna 1998

﻿*Nycteribia (Nycteribia) ﻿schmidtlii* Schiner, 1853

= *Listropodia ﻿﻿﻿schmidli* Schiner, in Séguy 1930a: 186

Falcoz 1924, **Rif**, Caverne de Samsa (near Tétouan); Séguy 1930a, **Rif**, Caverne de Samsa, **SA**; Aellen 1952; Aellen 1955, **AP**, Grotte de Sidi Bou Knadel (undetermined bat), **MA**, Grotte de Ras el Ma (host: *Rhinolophusferrumequinum* (Schreber, 1774)), Grotte de Ras el Oued (hosts: *Miniopterusshreibersii* (Kuhl, 1817) and *Myotisoxygnathus* Monticelli, 1885); Aellen 1963; Theodor 1967 (host: *Rhinolophusferrumequinum*); Mouna 1998

﻿***Penicillidia* Kolenati, 1963**

﻿*Penicillidia (Penicillidia) ﻿conspicua* Speiser, 1901

Falcoz 1924, **Rif**, Caverne d’Hercule, Caverne de Samsa (near Tétouan); Séguy 1930a, **Rif**, Caverne d’Hercule, Caverne de Samsa; Aellen 1952; Aellen 1955, **MA**, Grotte de Ras el Oued, **AP**, Grotte de Sidi Bou Knadel; Aellen 1963; Mouna 1998; Koçak and Kemal 2010

﻿*Penicillidia (Penicillidia) ﻿dufouri* (Westwood, 1835)

Falcoz 1924, **Rif**, Caverne d’Hercule; Séguy 1930a, **Rif**, Caverne d’Hercule; Aellen 1952; Aellen 1955, **AP**, Grotte de Sidi Bou Knadel, **MA**, Grotte de Ras el Oued, **AA**, Oulad Teima; Aellen 1963; Theodor 1967, **AP**, Mazagan (*Myotismyotis* (Bourhausen, 1797)); Mouna 1998; Koçak and Kemal 2010

﻿***Phthiridium* Hermann, 1804**

﻿*Phthiridium ﻿﻿﻿biarticulatum* Hermann, 1804

= *Stylidia ﻿﻿﻿biarticulata* Herman, in Falcoz 1924: 310; Séguy 1930a: 185

Falcoz 1924, **Rif**, Caverne de Samsa (near Tétouan); Séguy 1930a, **Rif**, Caverne de Samsa; Aellen 1952; Aellen 1955, **MA**, Grotte de Ras el Ma (host: *Rhinolophusferrumequinum* (Schreber, 1774)), **AA**, Oulad Teima (host: *Rhinolophusferrumequinum*); Aellen 1963; Theodor 1967 (host: *Rhinolophusferrumequinum*); Mouna 1998

#### ﻿﻿STREBLIDAE

K. Kettani, G. Graciolli

Number of species: **2**. Expected: 7

Faunistic knowledge of the family in Morocco: poor

##### Brachytarsininae

﻿***Brachytarsina* Macquart, 1851**

﻿*Brachytarsina ﻿﻿﻿flavipennis* Macquart, 1851

= *Nycteribosca ﻿﻿﻿kollari* Frauenfeld, in Falcóz 1924: 226; Aellen 1955: 100

Falcóz 1924, **Rif**, caverne d’Hercule, caverne de Samsa, près Tétouan (host: *Rhinolophusferrumequinum* (Schreber, 1774); Séguy 1930a, **Rif**, caverne d’Hercule, caverne de Samsa; Aellen 1955, **AP**, Grotte de Sidi Bou Knadel (hosts: *Rhinolophusmehelyi* (Matschie, 1901) and *Myotisoxygnathus* Monticelli, 1885); Mouna 1998; Koçak and Kemal 2010

﻿***Raymondia* Frauenfeld, 1855**

﻿*Raymondia ﻿﻿﻿huberi* Frauenfeld, 1855

= ﻿*Raymondia ﻿﻿﻿setosa* Jobling, 1930

Beaucournu et al. 1985, **AA**, Assa (Bas Draa) (host: *Asellia﻿tridens* (E. Geoffroy, 1813))

### ﻿Muscoidea

#### ﻿﻿ANTHOMYIIDAE

K. Kettani, D.M. Ackland

Number of species: **36**. Expected: Many more, especially in the mountains

Faunistic knowledge of the family in Morocco: poor

##### Anthomyiinae

﻿***Adia* Robineau-Desvoidy, 1830**

﻿*Adia ﻿﻿﻿cinerella* (Fallén, 1825)

= *Chortophila ﻿﻿﻿cinerella* Fallén, in Séguy 1930a: 162

= *Hylemyia ﻿﻿﻿cinerella* Fallén, in Séguy 1941d: 18

Séguy 1941d, **AA**, Agadir; Séguy 1930a; Mouna 1998; **AP** (Rabat), **HA** (Marrakech), **AA** (Tifnit (south of Agadir)) – MISR

﻿***Anthomyia* Meigen, 1803**

﻿*Anthomyia ﻿﻿﻿imbrida* Rondani, 1866

Séguy 1930a, **MA**, Meknès; Mouna 1998

﻿*Anthomyia ﻿﻿﻿liturata* (Robineau-Desvoidy, 1830)

= *Hylemyia ﻿﻿﻿pullula* Zetterstedt, in Séguy 1930a: 162

Séguy 1930a, **MA**, Ras el Ksar (1900 m), Tameghilt (1700–1800 m), Forêt Tiffert (2000–2200 m); Mouna 1998

﻿*Anthomyia ﻿﻿﻿quinquemaculata* Macquart, 1839

Ebejer et al. 2019, **Rif**, Jebel Lakraâ (Talassemtane, 1541 m), **MA**, 3.5 km S of Azrou (1450 m)

﻿*Anthomyia ﻿pluvialis* (Linnaeus, 1758)

Séguy 1929b; Séguy 1930a, **MA**, Meknès; Michelsen 1980, **AP**, Aïn Diab; Mouna 1998; Ackland 1987, 2001; Pârvu et al. 2006, **AA**, Foum Zguid (Tata); Popescu-Mirceni 2011 – MISR

﻿*Anthomyia ﻿﻿﻿procellaris* Rondani, 1866

Séguy 1929b; Séguy 1930a, **MA**, Meknès, Berkane (1350–1400 m), Tlet n’Rhohr; Mouna 1998

﻿*Anthomyia ﻿﻿﻿tempestatum* Wiedemann, 1830

Michelsen and Báez 1985, **HA**; Ackland 2001; Grabener 2017

﻿***Botanophila* Lioy, 1864**

﻿*Botanophila ﻿﻿﻿dissecta* (Meigen, 1826)

Mouna 1998; **MA** (Meknès) – MISR

﻿*Botanophila ﻿﻿﻿varicolor* (Meigen, 1826)

Ebejer et al. 2019, **MA**, Lac Aguelmane Sidi Ali (2052 m)

﻿***Delia* Robineau-Desvoidy, 1830**

﻿*Delia ﻿﻿﻿antiqua* (Meigen, 1826)

Mouna 1998

﻿*Deliacoarctata* (Fallén, 1825)

Mouna 1998

﻿*Delia ﻿﻿﻿flavibasis* (Stein, 1903)

= *Hylemyia ﻿﻿﻿hordeacea* Séguy, in Séguy 1941d: 18

Séguy 1934a, **AP**, Casablanca; Séguy 1936b, **AP**, Rabat; Séguy 1941d, **AA**, Taroudant; Mouna 1998; Ackland 2008

﻿*Delia ﻿﻿﻿flavogrisea* (Ringdahl, 1926)^52^

Pârvu et al. 2006, **AP**, Merja Zerga; Pârvu and Zaharia 2007; Popescu-Mirceni 2011

﻿*Delia ﻿﻿﻿planipalpis* (Stein, 1898)

= *Chortophila ﻿﻿﻿pilipyga* Villeneuve, in Mouna 1998: 85

Mouna 1998; **AP** (Mazagan) – MISR

﻿*Delia ﻿platura* (Meigen, 1826)

= *Chortophila ﻿﻿﻿cilicrura* Rondani, in Séguy 1930a: 162

Séguy 1949a, **SA**, Goulimine; Mouna 1998; Singh et al. 2014; Grabener 2017

﻿*Delia ﻿radicum* Linnaeus, 1758

= *Chortophila ﻿﻿﻿brassicae* Bouché, in Séguy 1934b: 162, Mouna 1998: 85

Séguy 1934b, **AP**, Rabat; Mouna 1998; Biron et al. 2000, **AP**, Rabat; Andreassen 2007 – MISR

﻿***Fucellia* Robineau-Desvoidy, 1842**

﻿*Fucellia ﻿﻿﻿maritima* (Haliday, 1838)

Séguy 1930a, **Rif**, Agla near Cap Spartel (on *Fucus*); Cassar et al. 2008, **Rif**, Smir lagoon; Mouna 1998

﻿***Hylemya* Robineau-Desvoidy, 1830**

﻿*Hylemya ﻿﻿﻿vagans* (Panzer, 1798)

Mouna 1998 – MISR

﻿***Leucophora* Robineau-Desvoidy, 1830**

﻿*Leucophora ﻿﻿﻿cinerea* Robineau-Desvoidy, 1830

Ebejer et al. 2019, **MA**, 17 km SW of Midelt (Khénifra, 1940 m)

﻿*Leucophora ﻿﻿﻿dissimilis* (Villeneuve, 1920)

Ebejer et al. 2019, **MA**, 17 km NW of Zaida (Khénifra, 1878 m)

﻿***Paregle* Schnabl, 1911**

﻿*Paregle ﻿﻿﻿audacula* (Harris, 1780)

Pârvu and Zaharia 2007

﻿*Paregle ﻿﻿﻿pilipes* (Stein, 1916)

Mouna 1998

﻿***Phorbia* Robineau-Desvoidy, 1830**

﻿*Phorbia ﻿﻿﻿fumigata* (Meigen, 1826)

= ﻿*Phorbia ﻿securis* Tiensuu, in Maarouf et al. 1996: 17

Balachowsky and Mesnil 1935; Bleuton 1938; Jourdan 1938; Maarouf and Chemseddine 1995, **AP**, Chaouia, Doukkala, Abda; Maarouf and Chemseddine 1995, **HA**, Chaouia, Doukkala, Abda; Maarouf et al. 1996, **AP**, Safi, Settat, Sidi El Aydi, Jemaa Riah, Berrechid, Médiouna, Mohammédia, Bouznika, Skhirat, Kénitra, Sidi Allal Tazi, Souk Larbaa du Gharb, **MA**, Khernisset, Meknès, Douiyat, Fès, Sefrou, Annaceur, Oulad Saïd, El Aounate, Sidi Bennbur, Zemarnra, Chemmaïa, Jemaa, des Shaïm, Tlet Sidi Bouguedra, Khemisset Chaouïa, Béni Mellal, **HA**, Skhour Rehamna, Ben Guérir, Marrakech, Tamellalet, Kelaâ des Sraghna, Oulad Ayad, Afourér, Azilal, El Borouj, Guisser; Lhaloui et al. 1998

﻿*Phorbiasepia* (Meigen, 1826)

Bleuton 1938, **MA**, Fès, Meknès, Taza; Jourdan 1938; Mouna 1998

﻿***Subhylemyia* Ringdahl, 1933**

﻿*Subhylemyia ﻿﻿﻿longula* (Fallén, 1824)

Mouna 1998; **AP** (Cap Cantin) – MISR

##### Pegomyinae

﻿***Calythea* Schnabl in Schnabl and Dziedzicki 1911**

﻿*Calythea ﻿﻿﻿nigricans* (Robineau-Desvoidy, 1830)

= ﻿*Calythea ﻿﻿﻿﻿albicincta* Fallén, in Séguy 1930a: 161

Séguy 1930a, **MA**, Meknès, Aïn Leuh; Mouna 1998

﻿***Mycophaga* Rondani, 1856**

﻿*Mycophagatestacea* (Gimmerthal, 1834)

Ebejer et al. 2019, **Rif**, Jebel Lakraâ (Talassemtane, 1541 m)

﻿***Pegomya* Robineau-Desvoidy, 1830**

﻿*Pegomya ﻿﻿﻿betae* (Curtis, 1847)

Rungs 1962, **AP**; Mouna 1998

﻿*Pegomya ﻿﻿﻿bicolor* (Wiedemann, 1817)

Séguy 1934b, **AP**, Rabat; Koçak and Kemal 2010; Pitkin et al. 2011

﻿*Pegomya ﻿﻿﻿hyoscyami* (Panzer, 1809)

Rungs 1962, **AP**; Mouna 1998; **AP** (Rabat) – MISR

﻿*Pegomya ﻿﻿﻿rufina* (Fallén, 1825)

Mouna 1998

﻿*Pegomyatestacea* (De Geer, 1776)

= ﻿*Pegomya ﻿﻿﻿silacea* Meigen, in Mouna 1998: 85

Séguy 1930a, **MA**, Forêt Tiffert (2000–2200 m); Mouna 1998

﻿*Pegomya ﻿﻿﻿solennis* (Meigen, 1826)

= *Pegomyia ﻿nigritarsis* Fallén, in Séguy 1935a: 119

Séguy 1935a, **AA**, Oued Draa (Taffagount); Rungs 1962; Mouna 1998

﻿*Pegomya ﻿﻿﻿terminalis* Rondani, 1866

Ebejer et al. 2019, **Rif**, Adrou (556 m), Jebel Lakraâ (Talassemtane, 1541 m)

﻿*Pegomya ﻿﻿﻿winthemi* (Meigen, 1826)

Mouna 1998

﻿***Pegoplata* Schnabl & Dziedzicki, 1911**

﻿*Pegoplata ﻿﻿﻿annulata* (Pandellé, 1899)

= ﻿*Pegoplata ﻿﻿﻿virginea* auctt, not Meigen

**AP** (Rabat) – MISR

#### ﻿﻿FANNIIDAE

K. Kettani, A.C. Pont

Number of species: **10**. Expected: 17

Faunistic knowledge of the family in Morocco: poor

﻿***Fannia* Robineau-Desvoidy, 1830**

﻿*Fannia ﻿﻿﻿canicularis* (Linnaeus, 1761)

Becker and Stein 1913, **Rif**, Tanger; Charrier 1927; Séguy 1930a, **HA**, Tenfecht; Séguy 1932a; Séguy 1941a; Séguy 1941d, **HA**; Séguy 1953a, **AP**, Rabat; Pont 1986a; Mouna 1998; Pont pers. comm., **MA**, Azrou; **AP** (Dradek) – MISR

﻿*Fannia ﻿﻿﻿cothurnata* (Loew, 1873)^53^

Mouna 1998

﻿*Fannia ﻿﻿﻿krimensis* Ringdahl, 1934

Pont 1983, **HA**, Jebel Ayachi; Pont 1986a; Mouna 1998

﻿*Fannia ﻿﻿﻿lepida* (Wiedemann, 1817)

Pont pers. comm.

﻿*Fannia ﻿﻿﻿leucosticta* (Meigen, 1838)

Becker and Stein 1913, **Rif**, Tanger; Séguy 1930a, **MA**; Pont 1986a; Mouna 1998

﻿*Fannia ﻿﻿﻿monilis* (Haliday, 1838)

Ebejer et al. 2019, **Rif**, Jebel Lakraâ (Talassemtane, 1541 m), Dardara (484 m)

﻿*Fannia ﻿﻿﻿norvegica* Ringdahl, 1934

Pont 1986a; Pont pers. comm., **HA**, Jebel Ayachi

﻿*Fannia ﻿﻿﻿pallidibasis* Pont, 1983

Pont 1983, **HA**, Jebel Ayachi; Mouna 1998

﻿*Fannia ﻿﻿﻿scalaris* (Fabricius, 1794)

Charrier 1927, **Rif**, Tanger; Séguy 1930a; Séguy 1941a, **HA**; Séguy 1953a, **AP**, Rabat; Pont 1986a; Mouna 1998; **Rif** (Tanger): Caverne d’Hercule (Pont pers. comm.) – MHNP

﻿*Fannia ﻿﻿﻿sociella* (Zetterstedt, 1845)^54^

Mouna 1998

#### ﻿﻿MUSCIDAE

K. Kettani, A.C. Pont

Number of species: **115**. Expected: 140

Faunistic knowledge of the family in Morocco: moderate

##### Atherigoninae

﻿***Atherigona* Rondani, 1856**

﻿*Atherigona ﻿﻿﻿humeralis* (Wiedemann, 1830)

Pont pers. comm., **AP**, Casablanca

﻿*Atherigona ﻿﻿﻿pulla* (Wiedemann, 1830)

Pont 1986b; Pont pers. comm., **AP**, Larache, Rabat, **HA**, Asni, **AA**, Agadir, Taroudant

﻿*Atherigona ﻿soccata* Rondani, 1871

= ﻿*Atherigona ﻿﻿﻿varia* (Meigen, 1826) (misidentifications of authors) in Séguy 1930a: 159

Bléton and Fieuzet 1943, **MA**, Fès; Séguy 1953a, **AP**, Rabat, Sidi Yahia du Gharb; Mouna 1998; Pont 1986b

﻿*Atherigona ﻿﻿﻿varia* (Meigen, 1826)

= ﻿*Atherigona ﻿﻿﻿quadripunctata* Rossi, in Séguy 1949a: 159

Séguy 1930a, **Rif**, Tanger; Séguy 1941d; Séguy 1949a, **AA**, Akka, Alnif; Bléton and Fieuzet 1943, **MA**, Meknès, Gharb, Fouarat; Séguy 1953a, **AP**, Sidi Yahia du Gharb, Rabat; Pont 1986b; Mouna 1998; Pont pers. comm., **Rif**, Meloussa, Tanger, **AP**, Larache, Rabat, **HA**, Jebel Ayachi, **AA**, Akka

﻿Atherigona (Acritochaeta) yorki Deeming, 1971

Pont 1986b, 1991a; Dike 1990; Pont pers. comm., **AP**, Rabat

##### Azeliinae

Azeliini

﻿***Azelia* Robineau-Desvoidy, 1830**

﻿*Azelia ﻿﻿﻿parva* Rondani, 1866

Michelsen pers. comm., **Rif**, Ouezzane

﻿***Hydrotaea* Robineau-Desvoidy, 1830**

﻿*Hydrotaea ﻿aenescens* (Wiedemann, 1830)

Morocco, first record 1989; Pont et al. 2007

﻿*Hydrotaea ﻿﻿﻿armipes* (Fallén, 1825)

Pont 1986b; Pont pers. comm., **MA**, Azrou, **HA**, Jebel Ayachi

﻿*Hydrotaea ﻿﻿﻿capensis* (Wiedemann, 1818)

= *Ophyra ﻿﻿﻿﻿anthrax* (Meigen, 1826), in Séguy 1941d: 20

Séguy 1934b, **AP**, Chellah; Séguy 1941d, **AA**, Agadir; Pont 1986b; Hernández-Moreno and Peris 1989, **Rif**, Tanger; Mouna 1998; Turchetto et al. 2003; Pont pers. comm., **HA**, Jebel Ayachi; **Rif** (Environ de Tanger (Pont pers. comm.)) – MHNP

﻿*Hydrotaea ﻿﻿﻿cinerea* Robineau-Desvoidy, 1830

Pont 1986b; Pont pers. comm., **HA**, Jebel Ayachi

﻿*Hydrotaea ﻿﻿﻿dentipes* (Fabricius, 1805)

Pont 1986b; Pont pers. comm., **HA**, Jebel Ayachi

﻿*Hydrotaea ﻿﻿﻿floccosa* Macquart, 1835

= ﻿*Hydrotaea ﻿﻿﻿armipes* (Fallén, 1825) (misidentifications of authors) in Charrier 1927: 620

Charrier 1927, **Rif**, Tanger; Pont 1986b; Pont pers. comm., **MA**, Azrou, **HA**, Jebel Ayachi

﻿*Hydrotaea ﻿﻿﻿glabricula* (Fallén, 1825)

Pont 1986b; Pont pers. comm., **AP**, Forêt Maâmora

﻿*Hydrotaea ﻿﻿﻿ignava* (Harris, 1780)

= *Ophyra ﻿﻿﻿leucostoma* (Wiedemann, 1817), in Séguy 1953a: 87

Séguy 1953a, **HA**, Tadla; Pont 1986b; Hernández-Moreno and Peris 1989, **Rif**, Tanger; Pont pers. comm., **AP**, Casablanca

﻿*Hydrotaea ﻿﻿﻿pellucens* Portschinsky, 1879

Pont 1986b; Pont pers. comm., **HA**, Jebel Ayachi

﻿*Hydrotaea ﻿﻿﻿tuberculata* Rondani, 1866

Michelsen pers. comm., **AP**, Rabat

﻿*Hydrotaea ﻿﻿﻿velutina* Robineau-Desvoidy, 1830

Pont 1986b; Pont pers. comm., **HA**, Jebel Ayachi

﻿***Thricops* Rondani, 1856**

﻿*Thricops ﻿﻿﻿simplex* (Wiedemann, 1817)

Pont 1986b, 1991b; Pont pers. comm., **HA**, Jebel Ayachi

##### Reinwardtiini

﻿***Muscina* Robineau-Desvoidy, 1830**

﻿*Muscina ﻿﻿﻿levida* (Harris, 1780)

= ﻿*Muscina ﻿﻿﻿assimilis* (Fallén, 1823)

Mouna 1998 – MHNP (no locality, on *Boletus ﻿﻿﻿nigrescens* (Pont pers. comm.)); **AP** (Rabat) –MISR

﻿*Muscina ﻿﻿﻿prolapsa* (Harris, 1780)

= ﻿*Muscina ﻿﻿﻿pabulorum* (Fallén, 1817)

Mouna 1998; Michelsen pers. comm., **AP**, Rabat, Larache; **AP** (Sidi Yahia) – MISR

﻿*Muscina ﻿﻿﻿stabulans* (Fallén, 1817)

Becker and Stein 1913, **Rif**, Tanger; Charrier 1927, **Rif**, Tanger; Séguy 1930a, **AP**, Rabat, Mogador, **MA**, Meknès, **HA**, Aguerd El Had, Souss; Séguy 1932, **HA**, Taroudant; Séguy 1934b, **AP**, Rabat; Séguy 1953a, **AP**, Rabat, Salé, Forêt Maâmora, Salé; Pont 1986b; Mouna 1998; Pont pers. comm., **AP**, Casablanca, **MA**, El Kebab, **HA**, Jebel Ayachi; **AP** (Casablanca, Rabat (Pont pers. comm.)) – MHNP; MISR

##### Coenosiinae

Coenosiini

﻿***Coenosia* Meigen, 1826**

﻿*Coenosia ﻿﻿﻿antennata* (Zetterstedt, 1849)

Michelsen pers. comm., **AP**, Larache

﻿*Coenosia ﻿﻿﻿atra* Meigen, 1830

Pont 1986b; Barták et al. 2004; Barták and Kubik 2005; Pont pers. comm., **HA**, Jebel Ayachi

﻿*Coenosia ﻿﻿﻿attenuata* Stein, 1903

Pont 1986b; Pont pers. comm., **EM**, Figuig

﻿*Coenosia ﻿﻿﻿humilis* Meigen, 1826

Pont 1986b; Pont pers. comm., **EM**, Figuig, **HA**, Imlil, Asni, **AA**, Agadir

﻿*Coenosia ﻿﻿﻿mixta* Schnabl, 1911

Pont 1986b

﻿*Coenosia ﻿﻿﻿nevadensis* Lyneborg, 1970

Pont 1986b; Pont pers. comm., **HA**, Jebel Ayachi

﻿*Coenosia ﻿﻿﻿pedella* (Fallén, 1825)

= ﻿*Coenosia ﻿﻿﻿decipiens* Meigen 1826 (certainly a misidentification) in Charrier 1927: 620

Charrier 1927, **Rif**, Tanger

﻿*Coenosia ﻿﻿﻿praetexta* Pandellé, 1899

Michelsen pers. comm., **AP**, Larache, Rabat

﻿*Coenosia ﻿﻿﻿pumila* (Fallén, 1825)^55^

Pont 1986b; Mouna 1998; Gregor et al. 2002

﻿*Coenosiatestacea* (Robineau-Desvoidy, 1830)

Pont 1986b; Pont pers. comm., **MA**, Ifrane, **HA**, Jebel Ayachi

﻿*Coenosia ﻿﻿﻿tigrina* (Fabricius, 1775)

Séguy 1930a, **AP**, Rabat, **MA**, Meknès; Pont 1986b; Mouna 1998; Pont pers. comm., **MA**, Ifrane, **HA**, Jebel Ayachi; **MA** (Ifrane) – MISR

﻿***Lispocephala* Pokorny, 1893**

﻿*Lispocephala ﻿﻿﻿brachialis* (Rondani, 1877)

Pont 1986b; Gregor et al. 2002; Barták and Kubik 2005; Pont pers. comm., **HA**, Jebel Ayachi

﻿*Lispocephala ﻿﻿﻿mikii* (Strobl, 1893)

Pont 1986b; Pont pers. comm., **EM**, Figuig, **HA**, Jebel Ayachi, **AA**, Agadir

﻿*Lispocephala ﻿ungulata* (Rondani, 1866)

Ackland and Pont 1966, **HA**, Jebel Ayachi; Pont 1986b

﻿***Orchisia* Rondani, 1877**

﻿*Orchisia ﻿﻿﻿costata* (Meigen, 1826)

Charrier 1927, **Rif**, Tanger; Pont 1986b

﻿***Schoenomyza* Haliday, 1833**

﻿*Schoenomyza ﻿﻿﻿litorella* (Fallén, 1823)

Séguy 1941a, **HA**, Tachdirt (Toubkal, 2500 m); Pont 1986b; Mouna 1998; Pont pers. comm., **EM**, Figuig, **HA**, Asni, Imlil, Jebel Ayachi

##### Limnophorini

﻿***Limnophora* Robineau-Desvoidy, 1830**

﻿*Limnophora ﻿﻿﻿bipunctata* Stein, 1908

Pont 1986b; Pont pers. comm., **EM**, Figuig

﻿*Limnophora ﻿﻿﻿flavitarsis* Stein, 1908

Pont pers. comm., **EM**, Figuig, **HA**, Jebel Ayachi

﻿*Limnophoramediterranea* Pont, 2012

Pont pers. comm., **HA**, Jebel Ayachi

﻿*Limnophora ﻿﻿﻿obsignata* (Rondani, 1866)

Séguy 1930a, **MA**, Aïn Leuh, **HA**, Aguerd El Had, Souss (Talekjount); Pont 1986b; Dakki 1997; Mouna 1998; Pont pers. comm., **MA**, Ifrane, **HA**, Jebel Ayachi, **AA**, Agadir

﻿*Limnophora ﻿﻿﻿olympiae* Lyneborg, 1965

Pont 1986b; Pont et al. 2012a; Pont pers. comm., **HA**, Jebel Ayachi

﻿*Limnophora ﻿﻿﻿pandellei* Séguy, 1923

Pont 1986b; Pont pers. comm., **HA**, Jebel Ayachi

﻿*Limnophora ﻿﻿﻿pollinifrons* Stein, 1916

Pont 1986b; Pont et al. 2012a; Pont pers. comm., **MA**, Aïn El Orma

﻿*Limnophora ﻿﻿﻿riparia* (Fallén 1824)

= *Melanochelia ﻿﻿﻿riparia* Fallén, in Séguy 1930a: 160

Séguy 1930a, **AA**, Souss (Tenfecht); Vaillant 1956b, **HA**, Cascade Siroua, Lac Tamhda (Anremer), Sidi Chamarouch; Pont 1986b; Mouna 1998

﻿*Limnophora ﻿﻿﻿rufimana* (Strobl, 1893)

Séguy 1930a, **MA**, Aïn Leuh; Pont 1986b; Pont pers. comm., **EM**, Figuig, **HA**, Jebel Ayachi

﻿*Limnophora ﻿﻿﻿tigrina* (Am Stein, 1860)

Pont 1986b; Pont pers. comm., **HA**, Jebel Ayachi

﻿***Lispe* Latreille, 1797**

﻿*Lispeassimilis* Wiedemann, 1824

= ﻿*Lispe ﻿﻿﻿inexpectata* Canzoneri & Meneghini, 1966

Canzoneri and Meneghini 1966, **MA**, Oued Fès (Taza); Pont 1986b; Vikhrev 2012b, **AP**, Essaouira, **HA**, Marrakech, **SA**, Tan-Tan

﻿*Lispe ﻿﻿﻿﻿﻿﻿﻿﻿﻿﻿apicalis* Mik, 1869

Canzoneri and Meneghini 1966, **MA**, Oued Sebou, **EM**, Guercif (Oued Moulouya); Canzoneri and Meneghini 1972, **MA**, Taza, Oued Fès; Pont 1986b; Koçak and Kemal 2010

﻿*Lispe ﻿﻿﻿bengalensis* (Robineau-Desvoidy, 1830)

= ﻿*Lispe ﻿﻿﻿berlandi* Séguy, in Séguy 1940: 341

Séguy 1940, **AA**, Rio de Oro (Oued Eddahab) (type locality of ﻿*berlandi*)

﻿*Lispe ﻿caesia* Meigen, 1826

= ﻿*Lispe ﻿﻿﻿microchaeta* Séguy, in Séguy 1940: 342

Séguy 1940, **AA**, Rio de Oro (Oued Eddahab) (type locality of ﻿*microchaeta*); Canzoneri and Meneghini 1966, **AP**, Fedhala (Oued Nefifikh), Saline di Sète; Pont 1986b; Mouna 1998; Koçak and Kemal 2010; Vikhrev et al. 2016, **AP**, Oualidia lagoon, Essaouira, **SA**, Tan-Tan (salt lagoon); **AP** (Chellah) – MISR; ZMUM

﻿*Lispe ﻿﻿﻿candicans* Kowarz, 1892

Séguy 1940, **AA**, Rio de Oro (﻿Villa Cisneros)

﻿*Lispe ﻿﻿﻿cilitarsis* Loew, 1856

Vikhrev 2012b, **SA**, Tan-Tan province

﻿*Lispe ﻿﻿﻿draperi* Séguy, 1930

Canzoneri and Meneghini 1966, **MA**, Azrou, Aguelmane, Fès (Oued Sebou); Vikhrev 2011a, **AP**, Essaouira, **HA**, Oued N’fis (east of Marrakech)

﻿*Lispe ﻿﻿﻿halophora* Becker, 1903

Vikhrev pers. comm., **SA**, Tan-Tan province

﻿*Lispe ﻿﻿﻿kowarzi* Becker, 1903

Vikhrev 2012c, **AP**, Essaouira

﻿*Lispe ﻿﻿﻿loewi* Ringdahl, 1922

= ﻿*Lispe ﻿﻿﻿litorea* Fallén, 1825 (misidentification of authors) in Séguy 1930a: 160

Séguy 1930a, **AP**, saline mud in Mediterranean region; Canzoneri and Meneghini 1966, **AP**, Fedhala; Pont 1986b; Dakki 1997; Mouna 1998

﻿*Lispe ﻿﻿﻿marina* Becker, 1913

Michelsen pers. comm., **AP**, Larache

﻿*Lispe ﻿﻿﻿melaleuca* Loew, 1847

Canzoneri and Meneghini 1966, **MA**, Azrou (Aguelmane); Pont 1986b, 1991b

﻿*Lispe ﻿﻿﻿modesta* Stein, 1913

Vikhrev 2012b, **AP**, Essaouira, **HA**, Marrakech, **SA**, Tan-Tan

﻿*Lispe ﻿﻿﻿nana* Macquart, 1835

Canzoneri and Meneghini 1966, **MA**, Taza (Oued Fès), Fès (Oued Sebou), Azrou (Aguelmane), **EM**, Guercif (Oued Moulouya); Pont 1986b; Mouna 1998; Pont pers. comm., **MA**, Aïn el Orma, **EM**, Figuig, **HA**, Jebel Ayachi; **Rif** (Oued Laou dunes), **AP** (Rabat) – MISR

﻿*Lispe ﻿﻿﻿nivalis* Wiedemann, 1830

Canzoneri and Meneghini 1966, **MA**, Taza (Oued Fès); Pont 1986b, 1991; Vikhrev 2012c, **AP**, Essaouira, **HA**, Ouarzazate province, **SA**, Tan-Tan province; Pont pers. comm., **MA**, Aïn El Orma

﻿*Lispe ﻿﻿﻿pectinipes* Becker, 1903

= *Lispa ﻿﻿﻿mixticia* Séguy, in Séguy 1941d: 19

Séguy 1941d, **HA**, Taroudant (type locality of ﻿*mixticia*); Pont 1986b; Mouna 1998; Kirk-Spriggs and McGregor 2009; Vikhrev 2011b, **AP**, Essaouira; Pont pers. comm., **HA**, Jebel Ayachi

﻿*Lispe ﻿﻿﻿pygmaea* Fallén, 1825

Canzoneri and Meneghini 1966, **MA**, Azrou (Aguelmane); Pont 1986b; Vikhrev 2012a, **AP**, Essaouira

﻿*Lispe ﻿﻿﻿rigida* Becker, 1903

Canzoneri and Meneghini 1966, **MA**, Taza (Oued Fès); Pont 1986b, 1991; Vikhrev 2012c, **HA**, Ouarzazate

﻿*Lispe ﻿﻿﻿scalaris* Loew, 1847

= ﻿*Lispe ﻿maroccana* Canzoneri & Meneghini, 1966 (as ﻿*scalaris* ssp.)

= ﻿*Lispepersica* Becker, 1904 in Kirk-Spriggs and McGregor 2009

Canzoneri and Meneghini 1966, **AP**, Fedhala, Dielfa (Oued Tadmid), **EM**, Guercif (Oued Moulouya), **MA**, Fès (Oued Sebou); Pont 1986b; Kirk-Spriggs and McGregor 2009; Vikhrev 2012a, **HA**, Ouarzazate province

﻿*Lispe ﻿tentaculata* (De Geer, 1776)

Séguy 1930a, **HA**, Kasba Taguendaft (Goundafa), Skoutana (Arround); Séguy 1941a, **HA**, Imi-n’Ouaka (1500 m); Pont 1986b; Dakki 1997; Mouna 1998; Kirk-Spriggs and McGregor 2009; Pont pers. comm., **MA**, Aïn el Orma, **EM**, Figuig, **HA**, Jebel Ayachi; **AP** (Rabat), **HA** (Tizi-n’Tichka) – MISR

##### Muscinae

Muscini

﻿***Dasyphora* Robineau-Desvoidy, 1830**

﻿*Dasyphora ﻿﻿﻿albofasciata* (Macquart, 1839)

= *Dasiphora ﻿﻿﻿saltuum* Rondani, 1862

Pont 1986b; Mouna 1998; Pont pers. comm., **HA**, Jebel Ayachi

﻿*Dasyphora ﻿﻿﻿penicillata* (Egger, 1856)

Pont 1986b; Koçak and Kemal 2010; Pont pers. comm., **HA**, Jebel Ayachi

﻿*Dasyphora ﻿﻿﻿cyanella* (Meigen, 1826)

Peris and Llorente 1963, **Rif**, Tanger; Mouna 1998

﻿***Morellia* Robineau-Desvoidy, 1830**

﻿*Morelliaasetosa* Baranov, 1925

= ﻿*Morellia ﻿﻿﻿simplex* (Loew, 1857) (misidentification of authors) in Peris and Llorente 1963, **Rif**, Tanger; Pont 1986b^56^

﻿***Musca* Linnaeus, 1758**

﻿*Musca ﻿﻿﻿autumnalis* De Geer, 1776

= ﻿*Musca ﻿﻿﻿corvina* Fabricius, 1781 in Séguy 1930a: 156

Charrier 1927, **Rif**, Tanger; Séguy 1930a, **AP**, Rabat, **MA**, Oued Korifla, Meknès; Pont 1986b; Mouna 1998; Pont pers. comm., **MA**, Azrou, **HA**, Jebel Ayachi; **MA** (Volubilis), **AA** (Tifnit) – MISR

﻿*Musca ﻿﻿﻿biseta* Hough, 1898

Pont 1986b; Pont pers. comm., **AP**, Temara, **MA**, Timhadit, Meknès, **EM**, near Figuig

﻿*Musca ﻿﻿﻿domestica* Linnaeus, 1758

Charrier 1927, **Rif**, Tanger; Séguy 1930a, 1932, 1934b, **AP**, Casablanca; Séguy 1934c, **AP**, Casablanca; Séguy 1941a, **HA**; Peris and Llorente 1963, **Rif**, Tanger, Melilla, Bab Taza, El Ajmas, Yebala; Pont 1986b; Mouna 1998; Pârvu et al. 2006, **AA**, Tiggane Tata; Popescu-Mirceni 2011; Pont pers. comm., **AP**, Temara, **MA**, Timhadit, Orionane, Lixus, Meknès, **HA**, Jebel Ayachi; Grabener 2017; **HA** (Jebel Tachdirt, 3100 m, Tachdirt (Bords Imminen), 2400–2600 m, Kasba Taguendaft (Goundafa), Andjera (Pont pers. comm.)) – MHNP

﻿*Musca ﻿﻿﻿larvipara* Portschinsky, 1910

Peris and Llorente 1963, **Rif**, Tanger; Pont 1986b; Pont pers. comm., **AP**, Forêt Maâmora

﻿*Musca ﻿﻿﻿osiris* Wiedemann, 1830

= ﻿*Muscavitripennis* Meigen, 1826 (misidentifications of authors) in Séguy 1930a: 157

Séguy 1941d, **AA**, Agadir; Pont 1986b

﻿*Musca ﻿sorbens* Wiedemann, 1830

= ﻿*Musca ﻿﻿﻿﻿﻿angustifrons* Thomson, 1869 (misidentification of authors) in Séguy 1930a: 156, 1940: 245, 1953a: 88

Séguy 1930a, **MA**, Oued Korifla, **HA**, Talingoult (Goundafa), Souss; Séguy 1940, **AA**, Rio de Oro (﻿Villa Cisneros); Séguy 1941d, **AA**, Agadir; Séguy 1949a, **AA**, from Foum Zguid to Zagora; Saccà 1952; Séguy 1953a, **AP**, Rabat; Peris and Llorente 1963; Pont 1986b, **Rif**, Melilla, Tanger, **AP**, Mogador; Mouna 1998; Koçak and Kemal 2010; Pont pers. comm., **MA**, Timhadit, **HA**; Grabener 2017 – MISR

﻿*Musca ﻿﻿﻿tempestiva* Fallén, 1817

Séguy 1941d; Pont 1986b; Pont pers. comm., **AP**, Forêt Maâmora, **HA**, Asni

﻿*Muscavitripennis* Meigen, 1926

= *Plaxemyia ﻿vitripennis* Meigen, 1826 in Becker and Stein 1913: 91

Becker and Stein 1913, **Rif**, Tanger; Séguy 1930a, **MA**, Ras el Ksar, Aïn Leuh, **HA**, Tinmel (Goundafa), Arround (Skoutana); Séguy 1941d, **AA**, Agadir; Peris and Llorent 1963, **Rif**, Tanger, Melilla, **AP**, Mogador; Pont 1986b; Mouna 1998; Pont pers. comm., **HA**, Jebel Ayachi; **Rif** (environs de Tanger, Sart. route de Spartel (Pont pers. comm.)) – MHNP; **AP** (Cap Cantin, Dradek), **MA** (Azrou) – MISR

﻿***Neomyia* Walker, 1859**

﻿*Neomyia ﻿﻿﻿cornicina* (Fabricius, 1781)

= *Cryptolucilia ﻿﻿﻿caesarion* (Meigen, 1826) in Séguy 1930a: 156

Becker and Stein 1913, **Rif**, Tanger; Charrier 1927, **Rif**, Tanger; Séguy 1930a, **Rif**, Tanger (Oued Judios), **AP**, Rabat, **MA**, M’Rirt, Aïn Leuh, Tizi-s’Tkrine, Forêt Zaers, Forêt Tiffert, **HA**, Arround (Skoutana), Tachdirt; Séguy 1941d (very common); Peris and Llorente 1963, **Rif**, Tanger, **AP**, Mogador, Tzalatza, Reisana, Desembocadura del Lixus; Pont 1986b; Dakki 1997; Mouna 1998; Pont pers. comm., **MA**, Azrou, **HA**, Jebel Ayachi – MISR

﻿*Neomyia ﻿﻿﻿viridescens* (Robineau-Desvoidy, 1830)

= *Orthellia ﻿﻿﻿cornicina* (Fabricius, 1781) (misidentifications of authors)

Charrier 1927, **Rif**, Tanger; Pont 1986b; Pont pers. comm., **HA**, Jebel Ayachi

﻿***Polietes* Rondani, 1866**

﻿*Polietes ﻿meridionalis* Peris & Llorente, 1963

Peris and Llorente 1963, **Rif**, Tanger; Pont 1986b; Pont pers. comm., **HA**, Jebel Ayachi

﻿***Pyrellia* Robineau-Desvoidy, 1830**

﻿*Pyrellia ﻿﻿﻿vivida* Robineau-Desvoidy, 1830

= ﻿*Pyrellia ﻿﻿﻿cadaverina* (Linnaeus, 1758) (misidentifications of authors) in Charrier 1927: 620; Séguy 1930a: 156; Peris and Llorente 1963: 252

= ﻿*Pyrellia ﻿﻿﻿serena* (Meigen, 1826) (misidentification of authors) in Charrier 1927: 620

Charrier 1927, **Rif**, Tanger; Séguy 1930a, **MA**, Aïn Leuh; Peris and Llorente 1963, **Rif**, Tanger; Pont 1986b; Pont pers. comm., **HA**, Jebel Ayachi

##### Stomoxyini

﻿***Haematobia* Le Peletier & Serville, 1828**

﻿*Haematobia ﻿﻿﻿irritans* (Linnaeus, 1758)

= *Lyperosia ﻿﻿﻿irritans* (Linnaeus, 1758) in Séguy 1930a: 157

Séguy 1930a, **MA**, Meknès; Pont 1986b; Mouna 1998

﻿***Stomoxys* Geoffroy, 1762**

﻿*Stomoxys ﻿calcitrans* (Linnaeus, 1758)

Charrier 1927, **Rif**, Tanger; Séguy 1930a; Séguy 1941d; Peris 1951, **Rif**, Tanger; Pont 1986b; Mouna 1998; Pârvu et al. 2006, **AA**, Tiggane Tata; Dsouli 2009; Popescu-Mirceni 2011; Pont pers. comm., **MA**, Meknès, Timhadit, Azrou, Sidi Mjber, Neguerett, Tazekka, **HA**, Jebel Ayachi, El Kebab, **AA**, Figuig; **HA** (Haute Réghaya (Pont pers. comm.)) – MHNP; **AP** (Rabat), **MA** (Volubilis), **HA** – MISR

##### Mydaeinae

﻿***Graphomya* Robineau-Desvoidy, 1830**

﻿*Graphomya ﻿﻿﻿maculata* (Scopoli, 1763)

Séguy 1930a, **MA**, Forêt Zaers, Aïn Leuh; Pont 1986b; Mouna 1998; Pont pers. comm., **HA**, Jebel Ayachi; **MA** (Volubilis) – MISR

﻿***Gymnodia* Robineau-Desvoidy, 1863**

﻿*Gymnodia ﻿﻿﻿eremophila* (Brauer & Bergenstamm, 1894)

Pont 1986b; Pont pers. comm., **HA**, Jebel Ayachi

﻿*Gymnodia ﻿﻿﻿polystigma* (Meigen, 1826)

= ﻿*Limnophora ﻿﻿﻿polystigma* (Meigen, 1826)

= *Brontaea ﻿﻿﻿polystigma* (Meigen, 1826)

Mouna 1998; **AP** (Rabat) – MISR

﻿*Gymnodia ﻿﻿﻿genurufa* (Pandellé, 1899)

Pont 1986b; Pont pers. comm., **HA**, Jebel Ayachi

﻿*Gymnodiatonitrui* (Wiedemann, 1824)

= ﻿*Limnophora ﻿tonitrui* (Wiedemann, 1824)

= *Brontaea ﻿tonitrui* (Wiedemann, 1824)

Séguy 1949a, **AA**, Tata; Saccà 1952, **AP**, Rabat; Pont 1986b; Mouna 1998

﻿***Hebecnema* Schnabl, 1889**

﻿*Hebecnema ﻿﻿﻿fumosa* (Meigen, 1826)

Séguy 1930a, **AP**, Casablanca; Pont 1986b; Mouna 1998; Pont pers. comm., **MA**, Azrou, **HA**, Jebel Ayachi; **Rif** (Tanger), **AP** (Mogador (Pont pers. comm.)) – MHNP

﻿*Hebecnema ﻿﻿﻿nigra* (Robineau-Desvoidy, 1830)

= ﻿*Hebecnema ﻿﻿﻿vespertina* (Fallén, 1823) (misidentifications of authors) in Séguy 1930a: 159

Séguy 1930a, **AP**, Casablanca; Pont 1986b; Mouna 1998; Pont pers. comm., **HA**, Jebel Ayachi

﻿*Hebecnema ﻿﻿﻿umbratica* (Meigen, 1826)

Pont 1986b

﻿***Myospila* Rondani, 1856**

﻿*Myospila ﻿﻿﻿meditabunda* (Fabricius, 1781)

Séguy 1941d, **AA**, Agadir; Pont 1970; Pont 1986b; Mouna 1998; Pont pers. comm., **HA**, Jebel Ayachi; **AP** (Cap Cantin) – MISR

##### Phaoniinae

﻿***Helina* Robineau-Desvoidy, 1830**

﻿*Helina ﻿﻿﻿clara* (Meigen, 1826)

= *Mydaea ﻿﻿﻿clara* (Meigen, 1826) in Séguy 1930a: 159

Becker and Stein 1913, **Rif**, Tanger; Charrier 1927, **Rif**, Tanger; Séguy 1930a, **AP**, Rabat; Pont 1986b; Mouna 1998; **AP** (Rabat) – MISR

﻿*Helina ﻿﻿﻿czernyi* Lyneborg, 1970

Michelsen pers. comm., **Rif**, Chefchaouen, Ouezzane, **MA**, Azrou, **HA**, Asni

﻿*Helina ﻿﻿﻿evecta* (Harris, 1780)

= *Mydaea ﻿﻿﻿lucorum* (Fallén, 1823) in Séguy 1930a: 159

Séguy 1930a; Werner 1938, **EM**, Oudjda-Berguent; Pont 1986b; Mouna 1998; Pont pers. comm., **MA**, Ifrane, **HA**, Jebel Ayachi

﻿*Helina ﻿﻿﻿nevadensis* Lyneborg, 1970

Pont 1986b; Pont pers. comm., **Rif**, Talassemtane, **HA**, Jebel Ayachi

﻿*Helina ﻿﻿﻿parcepilosa* (Stein, 1907)

Michelsen pers. comm., **AP**, Rabat, **HA**, Tinerhir

﻿*Helina ﻿﻿﻿quadrum* (Fabricius, 1805)

= *Mydaea ﻿﻿﻿quadrum* “Fallén” in Séguy 1930a: 159

Séguy 1930a, **AP**, Rabat; Pont, 1986b; Mouna 1998

﻿*Helina ﻿﻿﻿reversio* (Harris, 1780)

Pont 1986b; Pont pers. comm., **MA**, Azrou, **HA**, Jebel Ayachi; **MA** (Forêt Timelilt, 1650–1900 m (Pont pers. comm.)) – MNHN

﻿*Helina ﻿richardi* Pont, 2012

Pont 2012b, **Rif**, Ras el Ma, **MA**, Azrou, **HA**, Jebel Ayachi

﻿*Helina ﻿﻿﻿sexmaculata* (Preyssler, 1791)

= *Mydaea ﻿﻿﻿uliginosa* (Fallén, 1825)

Mouna 1998; Grabener 2017; **MA** (Aguelmane Azigza) – MISR

﻿*Helina ﻿﻿﻿vockerothi* Lyneborg, 1970

Michelsen pers. comm., **HA**, Tizi-n’Test (2100 m), Asni

﻿***Phaonia* Robineau-Desvoidy, 1830**

﻿*Phaonia ﻿﻿﻿cincta* (Zetterstedt, 1846)^57^

Charrier 1927, **Rif**, Tanger; Pont 1986b (record queried); Koçak and Kemal 2010

﻿*Phaonia ﻿﻿﻿errans* (Meigen, 1826)

Mouna 1998; Michelsen pers. comm., **MA**, Azrou; **AP** (Chellah), **MA** (Ifrane) – MISR

﻿*Phaonia ﻿﻿﻿exoleta* (Meigen, 1826)

Michelsen pers. comm., **AP**, Larache

﻿*Phaonia ﻿mediterranea* Hennig, 1963

Pont 1973, **HA**, Jebel Ayachi; Pont 1986b; Mouna 1998; Gregor et al. 2002

﻿*Phaonia ﻿﻿﻿rufipalpis* (Macquart, 1835)

Michelsen pers. comm., **Rif**, Ouezzane

﻿*Phaonia ﻿﻿﻿scutellata* (Zetterstedt, 1845)

Michelsen pers. comm., **Rif**, Ouezzane, **HA**, Asni, Tizi-n’Test, **AA**, Aoulouz

﻿*Phaonia ﻿﻿﻿subventa* (Harris, 1780)

Vikhrev and Erofeeva 2018, **HA**, Oukaimeden (2000 m); **Rif** (environs de Tanger (Pont pers. comm.)) – MHNP

﻿*Phaonia ﻿﻿﻿trimaculata* (Bouché, 1834)

Séguy 1930a, **AP**, Casablanca; Séguy 1934b, **AP**, Maâmora; Séguy 1953a, **AP**, Port Lyautey, Maâmora, Rabat; Pont 1986b; Dakki 1997; Mouna 1998; Pont pers. comm., **AP**, Forêt Maâmora, **HA**, Jebel Ayachi; **AP** (Rabat) – MISR

﻿*Phaonia ﻿﻿﻿tuguriorum* (Scopoli, 1763)

= ﻿*Phaonia ﻿﻿﻿signata* (Meigen, 1826) in Séguy 1930a: 158

Séguy 1930a, **MA**, Forêt Timlilt; Pont 1986b; Dakki 1997; Mouna 1998; Pont pers. comm., **HA**, Jebel Ayachi

﻿*Phaonia ﻿﻿﻿valida* (Harris, 1780)

= ﻿*Phaonia ﻿﻿﻿erratica* (Fallén, 1825) (misidentifications of authors) in Séguy 1953a: 87

Séguy 1953a, **MA**, Ifrane; Pont 1986b; Pont pers. comm., **HA**, Jebel Ayachi; **MA** (Ifrane (Pont pers. comm.)) – MHNP

﻿*Phaonia* ﻿sp. near ﻿*szelenyii* Mihályi, 1974

**HA** (Haute Réghaya (Pont pers. comm.)) – MHNP

#### ﻿﻿SCATHOPHAGIDAE

K. Kettani

Number of species: **3**. Expected: 4

Faunistic knowledge of the family in Morocco: poor

##### Scathophaginae

﻿***Norellia* Robineau-Desvoidy, 1830**

﻿*Norellia ﻿﻿﻿tipularia* (Fabricius, 1794)

Ebejer et al. 2019, **Rif**, Dardara (730 m)

﻿***Scathophaga* Meigen, 1803**

﻿*Scathophaga ﻿﻿﻿stercoraria* (Linnaeus, 1758)

= ﻿*Scathophaga ﻿﻿﻿merdaria* Fabricius, 1794, in Séguy 1930a: 163

Becker and Stein 1913, **Rif**, Tanger; Séguy 1930a, **Rif**, Tanger, **MA**, Meknès; Mouna 1998; Koçak and Kemal 2010; **AP** (Azemour, Oued Yakem, Cap Cantin) – MISR

﻿*Scathophaga ﻿﻿﻿lutaria* (Fabricius, 1794)

Ebejer et al. 2019, **Rif**, Talassemtane (1554 m), Jebel Lakraâ (1541 m)

### ﻿Oestroidea

#### ﻿﻿CALLIPHORIDAE

K. Kettani, K. Rognes

Number of species: **8**. Expected: 10

Faunistic knowledge of the family in Morocco: moderate

##### Calliphorinae

﻿***Bellardia* Robineau-Desvoidy, 1863**

﻿*Bellardia ﻿maroccana* (Villeneuve, 1941)

= *Onesia ﻿maroccana* Villeneuve, in Villeneuve 1932: 123

Villeuneuve 1932b; Schumann 1974, **AP**, Aïn Diab (Casablanca); Verves 2004; Koçak and Kemal 2010

﻿*Bellardia ﻿﻿﻿mascariensis* (Villeneuve, 1926)

Schumann 1974, **HA**, Marrakech; Verves 2004; Koçak and Kemal 2010

﻿***Calliphora* Robineau-Desvoidy, 1863**

﻿*Calliphora ﻿﻿﻿vicina* Robineau-Desvoidy, 1830

= ﻿*Calliphora ﻿﻿﻿erythrocephala* Meigen, in Séguy 1930a: 154

Séguy 1930a; Maurice 1947, **Rif**, Tanger; **AP** (Casablanca, Rabat), **MA** (Azrou, Ifrane) – MNHN, MISR

﻿*Calliphora ﻿﻿﻿vomitoria* (Linnaeus, 1758)

Séguy 1930a; Maurice 1947, **Rif**, Tanger; Kurahashi 1971; Kurahashi and Magpayo 2000; Mouna 1998; **AP** (Rabat), **HA** – MISR, NHMD

##### Chrysomyinae

﻿***Chrysomya* Robineau-Desvoidy, 1863**

﻿*Chrysomya ﻿﻿﻿albiceps* (Wiedemann, 1819)

Maurice 1947; Séguy 1949a, **HA**, Alnif; Gonzales-Mora and Peris 1988; Mouna 1998; Verves 2003; Grabener 2017; Dawah et al. 2019; **EM** (Oujda) – MISR, **AP** (Rabat) – MNHN

##### Luciliinae

﻿***Lucilia* Robineau-Desvoidy, 1863**

﻿*Lucilia ﻿﻿﻿sericata* (Meigen, 1824)

Séguy 1930a, **Rif**, Tanger, **EM**, Berkane (1350–1400 m), **MA**, Meknès, Berrechid, **HA**, Tizi-n’Test, Goundafa (Jebel Imdress, 2000–2450 m); Séguy 1953a, **AP**, Rabat; Mouna 1998; Grabener 2017; **AP** (Dradek, Salé), **MA** (Azrou, Volubilis) – MISR, **AP** (Temara), **MA** (Ifrane) – MNHN

##### Melanomyinae

﻿***Melinda* Robineau-Desvoidy, 1863**

﻿*Melinda ﻿﻿﻿gentilis* (Robineau-Desvoidy, 1830)

**MA** (Ifrane) – MNHN

﻿*Melinda ﻿﻿﻿viridicyanea* (Robineau-Desvoidy, 1830)

**MA** (Ifrane) – MNHN

#### ﻿﻿OESTRIDAE

K. Kettani, T. Pape

Number of species: **10**. Expected: 12

Faunistic knowledge of the family in Morocco: moderate

##### Gastrophilinae

﻿***Gasterophilus* Leach, 1817**

﻿*Gasterophilus﻿flavipes* (Olivier, 1811)

Séguy 1928d, **EM**, Haute Moulouya; Séguy 1930a, **EM**, Itzer (Haute Moulouya); Mouna 1998; Li et al. 2019

﻿*Gasterophilus ﻿﻿﻿haemorrhoidalis* (Linnaeus, 1758)

Maurice 1947; Mouna 1998; **AP** (Rabat) – MISR

﻿*Gasterophilus ﻿intestinalis* (De Geer, 1776)

Séguy 1930a; Mouna 1998; Pandey et al. 1992

﻿*Gasterophilusnasalis* (Linnaeus, 1758)

= ﻿*Gasterophilus ﻿﻿﻿veterinus* Clark, in Mouna 1998: 85

Maurice 1947; Pandey et al. 1992; Mouna 1998

﻿*Gasterophilus ﻿﻿﻿pecorum* (Fabricius, 1794)

Séguy 1930a; Mouna 1998

##### Hypodermatinae

﻿***Hypoderma* Rondani, 1856**

﻿*Hypoderma ﻿bovis* (Linnaeus, 1758)

Maurice 1947; Mouna 1998

﻿*Hypoderma ﻿lineatum* (De Villers, 1789)

Dakkok et al. 1978, **MA**, Benslimane; Mouna 1998

##### Oestrinae

﻿***Cephalopina* Strand, 1928**

﻿*Cephalopina ﻿﻿﻿titillator* (Clark, 1797)

= *Cephalopsis ﻿﻿﻿titillator* Clark, in Séguy 1930a: 139

Séguy 1930a, **Sub-SA** (camel breeding region); Mouna 1998

﻿***Oestrus* Linnaeus, 1758**

﻿*Oestrus ﻿﻿﻿ovis* Linnaeus, 1761

Séguy 1930a, **AP**, Rabat (sheep breeding area), **HA**, Marrakech, Bou Tazzert; Maurice 1947; Mouna 1998 – MISR

﻿***Rhinoestrus* Brauer, 1886**

﻿*Rhinoestrus ﻿﻿﻿purpureus* (Brauer, 1858)

Maurice 1947; Mouna 1998

#### ﻿﻿POLLENIIDAE

K. Kettani, K. Rognes

Number of species: **12**. Expected: 15

Faunistic knowledge of the family in Morocco: moderate

﻿***Pollenia* Fabricius, 1794**

﻿*Pollenia ﻿﻿﻿amentaria* (Scopoli, 1763)

Séguy 1949a, **AA**, Foum-el-Hassan; Séguy 1953a, **MA**, Sidi Allal Tazi; Mouna 1998; Pârvu et al. 2006, **SA**, Foum Zghouig; Popescu-Mirceni 2011; Gisondi et al. 2020

﻿*Pollenia ﻿﻿﻿bicolor* Robineau-Desvoidy, 1830

Rognes 1991a, **HA**, Mikdane (Jebel Ayachi); Rognes 1991b, **HA**, Mikdane (Jebel Ayachi); Gisondi et al. 2020; **HA** (Mikdane) – NHMUK

﻿*Pollenia ﻿﻿﻿contempta* Robineau-Desvoidy, 1863

Séguy 1930a, **MA**, Meknès, from M’Rirt to El Hajeb; Mouna 1998; Rognes 1992

﻿*Pollenia ﻿haeretica* Séguy, 1928

Séguy 1934b, **AP**, Rabat; Rognes 2010; **AP** (40 km S Larache) – NHMD

﻿*Pollenia ﻿﻿﻿ibalia* Séguy, 1930

= ﻿*Pollenia ﻿﻿﻿funebris* Villeneuve, in Villeneuve 1932: 284

= ﻿*Pollenia ﻿﻿﻿rungsi* Séguy, in Séguy 1953a: 88

Séguy 1930a, **EM**, Berkane, **MA**, Tlet n’Rhohr (in garden), Douar Ras el Ksar (900 m); Villeneuve 1932, **HA**, Marrakech; Séguy 1953a, **AP**, Rabat; Séguy 1941a, **HA**, cañon Tessaout (M’Goum, 3000–3200 m); Mouna 1998; Rognes 2010; Grabener 2017; Gisondi et al. 2020; **HA** (Asni) – NHMUK; **HA** (Ijoukak) – MNHN; **HA** (15 km SW Tazenakht) – NHMD

﻿*Pollenia ﻿﻿﻿leclercqiana* (Lehrer, 1978)

Rognes 2010, 2011; Gisondi et al. 2020

﻿*Pollenia ﻿﻿﻿luteovillosa* Rognes, 1987

Gisondi et al. 2020, **HA**, Mikdane (Jbel Ayachi)

﻿*Pollenia ﻿﻿﻿ponti* Rognes, 1991

Rognes 1991b, **HA**, Jaffar river (Jebel Ayachi); Gisondi et al. 2020; **HA** (Jebel Ayachi) – NHMUK

﻿*Pollenia ﻿rudis* (Fabricius, 1794)

Séguy 1930a, **EM**, Itzer (Haute Moulouya), Berkane (1350 m), **AP**, Casablanca, **MA**, Meknès, Aharmoumou (1100 m), Tlet n’Rhohr (in garden), Douar Ras el Ksar (1900 m), **HA**, Tizi-n’Test, Goundafa (Jebel Imdress, 2000–2450 m); Maurice 1947, **Rif**, Tanger; Rognes 1987, **AP**, Aïn Diab, Larache, **HA**, Asni, Jebel Ayachi, Mikdane; Grabener 2017; Gisondi et al. 2020; **AP** (Casablanca, Rabat), **MA** (Ifrane), **HA** (Haute Réghaya) – MNHN

﻿*Pollenia ﻿﻿﻿ruficrura* Rondani, 1862

Rognes 2011; Gisondi et al. 2020

﻿*Pollenia ﻿﻿﻿stigi* (Rognes, 1992)

Rognes 1992, **MA**, Ifrane, Azrou; Gisondi et al. 2020

﻿*Pollenia ﻿vagabunda* (Meigen, 1826)

= ﻿*Pollenia ﻿﻿﻿hasei* Séguy, in Séguy 1928e: 370, Séguy 1930a: 147

Séguy 1928e; Séguy 1930a, **AP**, Casablanca; Mouna 1998; Rognes 1992; Gisondi et al. 2020

#### ﻿﻿RHINIIDAE

K. Kettani, K. Rognes

Number of species: **17**. Expected: ~20

Faunistic knowledge of the family in Morocco: moderate

﻿***Cosmina* Robineau-Desvoidy, 1830**

﻿*Cosmina ﻿﻿﻿claripennis* Robineau-Desvoidy, 1830

= ﻿*Cosmina ﻿﻿﻿bezziana* Villeneuve, in Villeneuve 1932a: 285

Villeneuve 1932a, **AP**, Mogador; Schumann 1986; Mouna 1998; Rognes 2002

﻿*Cosmina ﻿maroccana* Séguy, 1949

Séguy 1949a, **AA**, Tenfecht, Vallée du Guir, **SA**, Guelmim; Séguy 1953a, **AA**, Tenfecht, Vallée du Guir, **SA**, Guelmim; Mouna 1998; Rognes 2002; Grabener 2017

﻿*Cosmina ﻿﻿﻿punctulata* Robineau-Desvoidy, 1830

Séguy 1930a, **AA**, Tenfecht (Souss, 1000–1500 m)

﻿*Cosmina ﻿﻿﻿viridis* (Townsend, 1917)

Ebejer et al. 2019, **AA**, 13 km E of Goulmima (1100 m)

﻿***Rhinia* Robineau-Desvoidy, 1830**

﻿*Rhinia ﻿﻿﻿﻿﻿﻿﻿﻿﻿﻿apicalis* (Wiedemann, 1830)

Séguy 1930a, **AP**, Rabat, **MA**, Forêt Zaers; Zumpt 1956; Gonzales-Mora and Peris 1988; Mouna 1998; Verves 2003; Lehrer 2007

﻿***Rhyncomya* Robineau-Desvoidy, 1830**

﻿*Rhyncomya ﻿﻿﻿callopis* (Loew, 1856)^58^

Séguy 1941d, **AA**, Agadir; Séguy 1953a, **AP**, Sidi Ifni, **SA**, Tindouf, Amguilli Sguelma, Guelta des Zemmours; Schumann 1986; Mouna 1998; Rognes 1992; Dawah et al. 2019; El Hawagry and El-Azab 2019; **MA** (Tafilalt) – MISR

﻿*Rhyncomya ﻿﻿﻿columbina* (Meigen, 1824)

Peris 1951, **Rif**, Tanger, **MA**, Ifrane; Schumann 1986; Gonzalez-Mora and Peris 1988

﻿*Rhyncomya ﻿﻿﻿cyanescens* (Loew, 1844)

= ﻿*Rhyncomya ﻿﻿﻿hemisia* Séguy, in Séguy 1930a: 150

Séguy 1930a, **EM**, Berkane (1350–1400 m); Gonzales-Mora and Peris 1988; Rognes 2002, **MA**

﻿*Rhyncomya ﻿﻿﻿desertica* (Peris, 1951)

Grabener 2017; El Hawagry and El-Azab 2019

﻿*Rhyncomya ﻿﻿﻿impavida* (Rossi, 1790)

Séguy 1930a, **Rif**, Tanger, **EM**, Berkane (1350–1400 m), **MA**, Forêt Tiffert (2000–2200 m); Schumann 1986; Mouna 1998

﻿*Rhyncomya ﻿﻿﻿nigripes* (Séguy, 1933)

Grabener 2017; El Hawagry and El-Azab 2019

﻿*Rhyncomya ﻿﻿﻿ursina* Séguy 1928

= *Rhynchomyia ﻿﻿﻿ursina* Séguy, in Séguy 1928b: 152

Séguy 1928b, **AP**, Atlantic coast of **SA**

﻿*Rhyncomya ﻿﻿﻿yahavensis* Rognes, 2002

Grabener 2017; Ebejer et al. 2019, **AA**, 30 km W of Errachidia (1065 m)

﻿*Rhyncomya ﻿﻿﻿zernyana* Villeneuve, 1926

Zumpt 1956; Gonzales-Mora and Peris 1988

﻿***Stomorhina* Rondani, 1861**

﻿*Stomorhina ﻿﻿﻿lunata* (Fabricius, 1805)

Séguy 1930a; Peris 1951; Gonzales-Mora and Peris 1988; Mouna 1998; **AP** (Rabat) – MISR

﻿***Villeneuviella* Austen, 1914**

﻿*Villeneuviellaicadion* (Séguy, 1953)

= *Rhynchoestrusicadion* Séguy, in Séguy 1953a: 89

Séguy 1953a, **SA**, Tindouf

﻿*Villeneuviellaweissi* (Séguy, 1926)

= *Rhynchoestrusweissi* Séguy, in Séguy 1934b: 162

Séguy 1934b, **EM**, Berkane Zobzit (1100 m); Séguy 1953a, **SA**, Mader Bergat

#### ﻿﻿RHINOPHORIDAE

K. Kettani, T. Pape

Number of species: **8**. Expected: 15

Faunistic knowledge of the family in Morocco: poor

##### Rhinophorinae

﻿***Melanophora* Meigen, 1803**

﻿*Melanophora ﻿﻿﻿roralis* (Linnaeus, 1758)

Peris 1963, **Rif**, Tanger, **AP**, Larache; Mouna 1998

﻿***Oplisa* Rondani, 1862**

﻿*Oplisa ﻿﻿﻿aterrima* (Strobl, 1899)

= *Hoplisa ﻿﻿﻿aterrima* Strobl, in Peris 1963: 602

Peris 1963, **Rif**, Tanger, Zoco de Taleta, Ketama; Mouna 1998; Cerretti et al. 2020

﻿***Paykullia* Robineau-Desvoidy, 1830**

﻿*Paykullia ﻿﻿﻿carmela* (Peris, 1963)

= *Chaetostevenia ﻿﻿﻿carmela* Peris, in Peris 1963: 606

Peris 1963, **Rif**, Tanger; Cerretti et al. 2020; Pape and Thompson 2019 – MNCN

﻿***Phyto* Robineau-Desvoidy, 1830**

﻿*Phyto ﻿﻿﻿atrior* (Villeneuve, 1941)

= *Styloneuria ﻿﻿﻿atrior* Villeneuve, in Villeneuve 1941: 122

Villeneuve 1941, **AP**, Rabat; Cerretti et al. 2020; Pape and Thompson 2019 – IRSNB

﻿*Phyto ﻿﻿﻿discrepans* Pandellé, 1896

Cerretti et al. 2020, **Rif**, Chefchaouen (600 m), Ouezzane (300 m), **MA**, 40 km N Fès (1150 m) – NHMD

﻿*Phyto ﻿﻿﻿melanocephala* (Meigen, 1824)

Ebejer et al. 2019, **Rif**, Barrage Smir (145 m); Cerretti et al. 2020

﻿***Stevenia* Robineau-Desvoidy, 1830**

﻿*Stevenia ﻿deceptoria* (Loew, 1847)

Mulieri et al. 2010; Cerretti et al. 2020

﻿***Tricogena* Rondani, 1856**

﻿*Tricogena ﻿﻿﻿rubricosa* (Meigen, 1824)

= *Frauenfeldia ﻿﻿﻿rubricosa* Meigen, in Peris 1963: 603

Peris 1963, **Rif**, Tanger; Mouna 1998; Cerretti et al. 2020 – NHMD

#### ﻿﻿SARCOPHAGIDAE

K. Kettani, D. Whitmore, T. Pape

Number of species: **66**. Expected: ~150

Faunistic knowledge of the family in Morocco: poor

##### Miltogramminae

﻿***Amobia* Robineau-Desvoidy, 1830**

﻿*Amobia ﻿﻿﻿signata* (Meigen, 1824)

Pape 1996; Verves 2019

﻿***Apodacra* Macquart, 1854**

﻿*Apodacra ﻿﻿﻿﻿﻿﻿africana* Rohdendorf, 1930

Pape 1996, **Rif**, Tanger; Verves 2019

﻿***Craticulina* Pandellé, 1895**

﻿*Craticulina ﻿﻿﻿antachates* (Séguy, 1949)

= ﻿*Apodacra ﻿﻿﻿antachates* Séguy, in Séguy 1949a: 160

Séguy 1949a, **AA**, Zagora; Pape 1996, **AA**, Zagora; Mouna 1998

﻿*Craticulina ﻿﻿﻿tabaniformis* (Fabricius, 1805)

Fabricius 1805, **AP**, Mogador; Séguy 1930a, **AP**, Mogador; Séguy 1935a, **AP**, beach of Rabat; Pape 1996; El Hawagry and El-Azab 2019; Verves 2019

﻿***Dolichotachina* Villeneuve, 1913**

﻿*Dolichotachina ﻿﻿﻿marginella* (Wiedemann, 1930)

Pape 1996; Grabener 2017; El Hawagry and El-Azab 2019; Verves 2019

﻿***Macronychia* Rondani, 1859**

﻿*Macronychia ﻿﻿﻿lemariei* Jacentkovský, 1941*

**AP**

﻿*Macronychia ﻿﻿﻿polyodon* (Meigen, 1824)

Pape 1996; Verves 2019

﻿***Metopia* Meigen, 1803**

﻿*Metopia ﻿﻿﻿argyrocephala* (Meigen, 1824)

= ﻿*Metopia ﻿﻿﻿leucocephala* (Rossi), in Mouna 1998: 86

Mouna 1998

﻿***Miltogramma* Meigen, 1803**

﻿*Miltogramma ﻿﻿﻿aurifrons* Dufour, 1850

Séguy 1930a, **AP**, Rabat, **MA**, Meknès; Pape 1996; Mouna 1998; El Hawagry and El-Azab 2019; Verves 2019

﻿*Miltogramma ﻿﻿﻿germari* Meigen, 1824

Séguy 1930a, **MA**, Meknès, from M’Rirt to El Hajeb, Sidi Taibi; Pape 1996; Mouna 1998; El Hawagry and El-Azab 2019; Verves 2019

﻿*Miltogramma ﻿maroccana* (Séguy, 1941)

= *Sphecapatodes﻿maroccana* Séguy, in Séguy 1941d: 22

Séguy 1941d, **AA**, Taroudant; Pape and Szpila 2012

﻿*Miltogramma ﻿﻿﻿murina* Meigen, 1824

Pape 1996; Verves 2019

﻿*Miltogramma ﻿﻿﻿oestracea* (Fallén, 1820)

Ebejer et al. 2019, **Rif**, Belwazen (M’Diq, 200 m), **AP**, Lower Loukous saltmarsh (2 m)

﻿*Miltogramma ﻿﻿﻿rutilans* Meigen, 1824

Ebejer et al. 2019, **Rif**, Oued Mhajrate (Ben Karrich, 180 m)

﻿*Miltogramma ﻿﻿﻿testaceifrons* (Roser, 1840)

= ﻿*Miltogramma ﻿﻿﻿pilitarsis* Rondani, in Séguy 1930a: 145; Mouna 1998: 86

Séguy 1930a, **MA**, Aïn Leuh; Pape 1996; Mouna 1998

﻿***Protomiltogramma* Townsend, 1916**

﻿*Protomiltogramma ﻿fasciata* (Meigen, 1824)

= *Setuliafasciata* (Meigen), in Mouna 1998: 86

Pape 1996; El Hawagry and El-Azab 2019; Verves 2019

﻿***Senotainia* Macquart, 1846**

﻿*Senotainia ﻿﻿﻿﻿﻿﻿albifrons* (Rondani, 1859)

= *Sphecapata ﻿﻿﻿﻿﻿﻿albifrons* Rondani, in Séguy 1930a: 145

Séguy 1930a

﻿***Taxigramma* Macquart, 1850**

﻿*Taxigramma ﻿﻿﻿heteroneura* (Meigen, 1830)

Pape 1996; El Hawagry and El-Azab 2019; Verves 2019

﻿*Taxigramma ﻿﻿﻿pluriseta* (Pandellé, 1895)

Ebejer et al. 2019, **Rif**, Oued Mhajrate (Ben Karrich, 180 m), **AP**, Lower Loukous saltmarsh (2 m)

##### Paramacronychiinae

﻿***Nyctia* Robineau-Desvoidy, 1830**

﻿*Nyctia ﻿﻿﻿halterata* (Panzer, 1798)

= ﻿*Musca ﻿﻿﻿maura* Fabricius, in Fabricius 1805: 302

Fabricius 1805, **Rif**, Tanger; Pape 1996, **Rif**, Tanger; Grabener 2017; El Hawagry and El-Azab 2019; Verves 2019

﻿*Nyctia ﻿﻿﻿lugubris* (Macquart, 1834)

Ebejer et al. 2019, **AP**, Lower Loukous saltmarsh (2 m)

﻿***Sarcophila* Rondani, 1856**

﻿*Sarcophila ﻿﻿﻿latifrons* (Fallén, 1817)^59^

Séguy 1930a, **AP**, Maâmora, **HA**, Skoutana (Arround, 2000–2400 m); Mouna 1998

﻿***Wohlfahrtia* Brauer & Bergenstamm, 1889**

﻿*Wohlfahrtia ﻿﻿﻿bella* (Macquart, 1839)

= *Disjunctio ﻿﻿﻿bella* (Macquart), in Séguy 1930a: 144

Séguy 1930a, **MA**, Aïn Leuh; Pape 1996; Mouna 1998; Hall et al. 2009; El Hawagry and El-Azab 2019; Verves 2019

﻿*Wohlfahrtia ﻿﻿﻿indigens* Villeneuve, 1928

Pape 1996; El Hawagry and El-Azab 2019; Verves 2019

﻿*Wohlfahrtia ﻿magnifica* (Schiner, 1862)

Delanoë 1922, **AP**, Doukkala; Séguy 1930a, **AP**, Maâmora; Séguy 1941a, **HA**, Tizi-n’Icheden (3000 m); Maurice 1947; Pape 1996; Mouna 1998; Lmimouni et al. 2004; Tliqui et al. 2007; Farkas et al. 2009, **Rif**, Al Hoceima, Taguidit, Tafensa, **EM**, Aghbal; Hall et al. 2009; El Hawagry and El-Azab 2019; Verves 2019

﻿*Wohlfahrtia ﻿﻿﻿nuba* (Wiedemann, 1830)

Pape 1996; El Hawagry and El-Azab 2019; Verves 2019

﻿*Wohlfahrtia ﻿﻿﻿trina* (Wiedemann, 1830)

Pape 1996; Mouna 1998; El Hawagry and El-Azab 2019

##### Sarcophaginae

﻿***Blaesoxipha* Loew, 1861**

﻿*Blaesoxipha (Blaesoxipha) ﻿lapidosa* Pape, 1994

Pape 1996; Grabener 2017; El Hawagry and El-Azab 2019

﻿*Blaesoxipha (Blaesoxipha) ﻿litoralis* (Villeneuve, 1911)

Pape 1996; Verves 2019

﻿*Blaesoxipha (Blaesoxipha) ﻿pygmaea* (Zetterstedt, 1844)

Pape 1996; Verves 2019

﻿*Blaesoxipha (Servaisia) ﻿rossica* Villeneuve, 1912

Pape 1996; Verves 2019

﻿***Ravinia* Robineau-Desvoidy, 1863**

﻿*Ravinia ﻿﻿﻿pernix* (Harris, 1780)

= *Gesneriodes ﻿﻿﻿disjuncta* Séguy, in Séguy 1938: 43

= ﻿*Sarcophaga ﻿﻿﻿striata* (Fabricius), in Séguy 1941d: 22, Séguy 1949a: 159

Séguy 1938, **HA**, Skoutana; Séguy 1941d, **AA**, Taroudant; Séguy 1949a, **AA**, Akka; Pape 1996; Grabener 2017; El Hawagry and El-Azab 2019; Verves 2019

﻿***Sarcophaga* Meigen, 1826**

﻿*Sarcophaga (Bercaea) ﻿africa* (Wiedemann, 1824)

Pape 1996; Abkari et al. 1998; Verves 2003, 2019; Grabener 2017; El Hawagry and El-Azab 2019

﻿*Sarcophaga (Helicophagella) ﻿maculata* Meigen, 1835

Pape 1996; El Hawagry and El-Azab 2019; Verves 2019

﻿*Sarcophaga (Helicophagella) ﻿melanura* Meigen, 1826

El Hawagry and El-Azab 2019; Verves 2019

﻿*Sarcophaga (Helicophagella) ﻿novercoides* Böttcher, 1913*

**Rif**, **HA**, **AA**

﻿*Sarcophaga (Heteronychia) ﻿balanina* Pandellé, 1896

Whitmore et al. 2013, **AP**, Larache; Fendane et al. 2018, **AP**, Diabat (Essaouira), Sidi Abed (El Jadida), Bir Retma (Casablanca); Verves 2019

﻿*Sarcophaga (Heteronychia) ﻿cucullans* Pandellé, 1896

Séguy 1930a, **HA**, Maharidja; Mouna 1998

﻿*Sarcophaga (Heteronychia) ﻿ferox* Villeneuve, 1908

Whitmore 2011, **Rif**, Ouezzane, **AP**, Larache, **MA**, Béni Mellal, Afourer, **AA**, Aoulouz; Whitmore et al. 2013; El Hawagry and El-Azab 2019; Verves 2019

﻿*Sarcophaga (Heteronychia) ﻿filia* Rondani, 1860

Whitmore 2011, **MA**, Azrou, Timahdit; Whitmore et al. 2013; Verves 2019

﻿*Sarcophaga (Heteronychia) ﻿javita* (Peris, González-Mora & Mingo, 1998)*

**AA**

﻿*Sarcophaga (Heteronychia) ﻿longestylata* Strobl, 1906

Pape 1996; Whitmore et al. 2013, **MA**, Ifrane, Azrou; Fendane et al. 2018, **AP**, Sidi Abed (El Jadida)

﻿*Sarcophaga (Heteronychia) ﻿minima* Rondani, 1862

Whitmore 2011, **MA**, Azrou, Ifrane, Afourer (Béni Mellal), **HA**, Ijoukak, Ouirgane (Marrakech), **AA**, Oulma (Agadir); Whitmore et al. 2013; Fendane et al. 2018, **AP**, Smimou (Essaouira), El Akarta (Oualidia); Verves 2019

﻿*Sarcophaga (Heteronychia) ﻿obvia* (Povolný, 2004)

Whitmore et al. 2013, **MA**, Afourer (Béni Mellal), **HA**, Ait Lekak (Marrakech), S Asni (Imlil, Marrakech), Tagadirt, Quirgane, **AA**, Oulma Ort (Agadir)

﻿*Sarcophaga (Heteronychia) ﻿pandellei* (Rohdendorf, 1937)

Séguy 1930a, **MA**, Tizi-s’Tkrine; Mouna 1998; Whitmore et al. 2013, **MA**, Azrou, Ifrane, Afourer (Béni Mellal)

﻿*Sarcophaga (Heteronychia) ﻿tangerensis* Whitmore, 2011

= ﻿*Heteronychia (Heteronychia) ﻿amica* Peris, González-Mora & Mingo, in Peris et al. 1998: 173

Peris et al. 1998, **Rif**, Tanger; Whitmore 2011, **Rif**, Tanger

﻿*Sarcophaga (Heteronychia) ﻿villeneuveana* (Enderlein, 1928)

= *Pierretia (Bercaea) maroccana* Rohdendorf, in Rohdendorf 1937: 325

= ﻿*Sarcophaga (Heteronychia) ﻿penicillata* Villeneuve, in Coupland and Barker 2004: 113 (misidentification)

Rohdendorf 1937, **MA**, Aïn Defali; Coupland and Barker 2004; Whitmore 2009; Fendane et al. 2018, **AP**, Diabat (Essaouira), Ghabat Tansift (Souiria), Lalla Fatna (Safi), Laatoutate (Safi), El Akarta (Oualidia), Sidi Abed (El Jadida), Bir Retma (Casablanca); Verves 2019

﻿*Sarcophaga (Heteronychia) ﻿uncicurva* Pandellé, 1896

Fendane et al. 2018, **AP**, Smimou (Essaouira), Diabat (Essaouira), Lalla Fatna (Safi), Laatoutate (Safi), Bir Retma (Casablanca)

﻿*Sarcophaga (Liopygia) ﻿argyrostoma* (Robineau-Desvoidy, 1830)*

**HA**

﻿*Sarcophaga (Liopygia) ﻿crassipalpis* Macquart, 1839

Pape 1996; Grabener 2017; El Hawagry and El-Azab 2019; Verves 2019

﻿*Sarcophaga* (﻿﻿*Liosarcophaga) ﻿aegyptica* Salem, 1935

Fendane et al. 2018, **AP**, Sidi Abed (El Jadida)

﻿*Sarcophaga* (﻿﻿*Liosarcophaga) ﻿dux* Thomson, 1869*

**SA**

﻿*Sarcophaga* (﻿﻿*Liosarcophaga) ﻿jacobsoni* (Rohdendorf, 1937)

Pape 1996; El Hawagry and El-Azab 2019; Verves 2019

﻿*Sarcophaga* (﻿﻿*Liosarcophaga) ﻿marshalli* Parker, 1923

Fendane et al. 2018, **AP**, Smimou (Essaouira), Diabat (Essaouira), Ghabat Tansift (Souiria), Laatoutate (Safi); El Hawagry and El-Azab 2019; Verves 2019

﻿*Sarcophaga* (﻿﻿*Liosarcophaga) ﻿pharaonis* Rohdendorf, 1934

Carles-Tolrá 2002; El Hawagry and El-Azab 2019; Verves 2019

﻿*Sarcophaga* (﻿﻿*Liosarcophaga) ﻿tibialis* Macquart, 1851

= ﻿*Sarcophaga ﻿﻿﻿beckeri* Villeneuve, in Maurice 1947: 57; Mouna 1998: 86

Maurice 1947; Mouna 1998; Fendane et al. 2018, **AP**, Laatoutate (Safi)

﻿*Sarcophaga* (﻿﻿*Liosarcophaga) ﻿teretirostris* Pandellé, 1896

= ﻿*Parasarcophaga ﻿﻿﻿decellei* Lehrer, in Lehrer 1976: 3

Lehrer 1976, **MA**, Kandar, Imouzzer; Pape 1996, **MA**, Kandar, Imouzzer; Verves 2019

﻿*Sarcophaga* (﻿﻿*Liosarcophaga) tuberosa* Pandellé, 1896^60^

Mouna 1998

﻿*Sarcophaga (Myorhina) ﻿nigriventris* Meigen, 1826

Pape 1996; Mouna 1998; Fendane et al. 2018, **AP**, Ghabat Tansift (Souiria), Laatoutate (Safi), El Akarta (Oualidia), Sidi Abed (El Jadida), Bir Retma (Casablanca); Verves 2019

﻿*Sarcophaga (Myorhina) ﻿soror* Rondani, 1860

Fendane et al. 2018, **AP**, Sidi Abed (El Jadida)

﻿*Sarcophaga (Pandelleana) ﻿protuberans* Pandellé, 1896

Séguy 1949a, **AA**, Agadir Tissint, **SA**, Guelmim, Foum-el-Hassan, Tata; Mouna 1998

﻿*Sarcophaga (Parasarcophaga) hirtipes* Wiedemann, 1830

Pape 1996; Verves 2003; El Hawagry and El-Azab 2019; Verves 2019

﻿*Sarcophaga (Sarcophaga) ﻿lehmanni* Müller, 1922

Pape 1996; Cassar et al. 2005, **Rif**, Smir lagoon; El Hawagry and El-Azab 2019; Verves 2019

﻿*Sarcophaga (Sarcophaga) ﻿marcelleclercqi* Lehrer, 1975

Lehrer 1975; Pape 1996, **MA**, Azrou; Verves 2019

﻿*Sarcophaga (Thyrsocnema) ﻿belgiana* (Lehrer, 1976)

Lehrer 1976; Pape 1996

﻿*Sarcophaga (Thyrsocnema*) ﻿sp.^61^

Ebejer et al. 2019 [as *incisilobata*, misidentification], **Rif**, Tahaddart (8 m)

##### New records for Morocco

﻿*Macronychia ﻿﻿﻿lemariei* Jacentkovský, 1941

Atlantic plain: Rabat, Forêt de Maâmora, 100 m, 25–26.iv.1989, 1♂1♀, Zoological Museum of Copenhagen Expedition (NMHD).

﻿*Sarcophaga (Helicophagella) ﻿novercoides* Böttcher, 1913

Rif: Ouezzane, 300 m, 21–22.iv.1989, 1♂, Zoological Museum of Copenhagen Expedition (NMHD).

High Atlas: Marrakech, Ouirgane, 1000 m, 1–9.iv.1997 Mai, 1♂, C. Kassebeer leg. (NMHD).

Anti Atlas: 30 km NW Aoulouz, 1400 m, 10.iv.1989, 1♂, Zoological Museum of Copenhagen Expedition (NMHD).

﻿*Sarcophaga (Heteronychia) ﻿javita* (Peris, González-Mora & Mingo, 1998)

Anti Atlas: Agadir, S Oulma, 30°31'N, 9°09'W, 200 m, 21.iv.1997, 1♂, C. Kassebeer leg. (NMHD).

﻿*Sarcophaga (Liopygia) ﻿argyrostoma* (Robineau-Desvoidy, 1830)

High Atlas: Marrakech, Tagadirt, Ouirgane, 1000 m, 1.x.1994, 1♀, C. Kassebeer leg. (NMHD).

﻿*Sarcophaga (Liosarcophaga) ﻿dux* Thomson, 1869

Sahara: Erfoud, Rissani area, 900 m, 13–14.iv.1989, Zoological Museum of Copenhagen Expedition (NHMD).

#### ﻿﻿TACHINIDAE

K. Kettani, P. Cerretti, H.-P. Tschorsnig

Number of species: **147**. Expected: 200

Faunistic knowledge of the family in Morocco: poor

##### Dexiinae

Dexiini

﻿***Billaea* Robineau-Desvoidy, 1830**

﻿*Billaea ﻿﻿﻿lata* (Macquart, 1849)

= *Rhynchodinera ﻿﻿﻿lata* Macquart, in Séguy 1930a: 143

Séguy 1930a, **MA**, Aharmoumou, Camp Boulhout, Sidi Bettache, Aïn Sferguila, Meknès; Mouna 1998; **AP** (Mehdia) – MISR; **AP** (Essaouira, 4 km E Ounara), **HA** (Marrakech, Lakhdar, N Demnate) – PCPT

﻿***Estheria* Robineau-Desvoidy, 1830**

﻿*Estheria ﻿﻿﻿atripes* Villeneuve, 1920

Cerretti and Tschorsnig 2012

﻿*Estheria ﻿﻿﻿iberica* Tschorsnig, 2003*

**MA**

﻿*Estheria ﻿﻿﻿nigripes* (Villeneuve, 1920)

Cerretti and Tschorsnig 2012; **MA** (Béni Mellal, El Ksiba), **AA** (Agadir, Oulma) – PCPT

﻿*Estheria ﻿﻿﻿picta* (Meigen, 1826)^62^

Moutia 1940

﻿***Zeuxia* Meigen, 1826**

﻿*Zeuxia ﻿﻿﻿aberrans* (Loew, 1847)

= ﻿*Zeuxia ﻿﻿﻿nigripes* Meigen, in Séguy 1941d: 23

Brémond 1938; Séguy 1941d, **AP**, Rabat, **MA**, Volubilis, **AA**, Agadir; IOBC-List 11; Mesnil 1980; Mouna 1998; Tschorsnig 2017; **AA** (10 km NW Aït-Baha) – PCPT

##### Dufouriini

﻿***Dufouria* Robineau-Desvoidy, 1830**

﻿*Dufouria ﻿﻿﻿nigrita* (Fallén, 1810)

Ebejer et al. 2019, **AP**, Larache (Lower Loukous saltmarsh, 2 m); **MA** (Ouzoud) – PCPT

##### Voriini

﻿***Athrycia* Robineau-Desvoidy, 1830**

﻿*Athrycia ﻿﻿﻿trepida* (Meigen, 1824)*

**MA**

﻿***Cyrtophloeba* Rondani, 1856**

﻿*Cyrtophloeba ﻿﻿﻿ruricola* (Meigen, 1824)

= *Plagia ﻿﻿﻿ruricola* Meigen, in Séguy 1935a: 120, in Rungs 1940: 14

Séguy 1935a, **MA**, Ifrane; Rungs 1940, **MA** (Cédraie); Mouna 1998; **HA** (Tizi-n’Test), **AA** (Taroudant) – PCPT

﻿***Eriothrix* Meigen, 1830**

﻿*Eriothrix ﻿﻿﻿apennina* (Rondani, 1862)

Herting and Dely-Draskovits 1993; Koçak and Kemal 2010

﻿*Eriothrix ﻿﻿﻿rufomaculata* (De Geer, 1776)*

**MA**, **HA**

﻿***Hypovoria* Villeneuve, 1913**

﻿*Hypovoria ﻿﻿﻿hilaris* Villeneuve, 1912

Séguy 1935a, **AP**, Oued Beth; Séguy 1953a, **AP**, Sehoul; Mouna 1998; **AA** (10 km SE Aït-Ourir) – PCPT

﻿*Hypovoria ﻿﻿﻿pilibasis* (Villeneuve, 1922)

Zeegers 2010; **HA** (Tizi-n’Test), **AA** (Taroudant) – PCPT

﻿***Kirbya* Robineau-Desvoidy, 1830**

﻿*Kirbya ﻿﻿﻿moerens* (Meigen, 1830)*

**MA**

﻿***Nanoplagia* Villeneuve, 1929**

﻿*Nanoplagiasinaica* (Villeneuve in Hermann & Villeneuve 1909)

Cerretti 2009; Grabener 2017; **HA** (Marrakech, 8 km N Ouirgane), **AA** (40 km SW Ouarzazate, 10 km SW Tazenakht, NE Agadir, 12 km W Oulma) – PCPT

﻿***Periscepsia* Gistel, 1848**

﻿*Periscepsia ﻿﻿﻿meyeri* (Villeneuve, 1930)

Ebejer et al. 2019, **Rif**, Adrou (PNPB, 556 m)

﻿***Stomina* Robineau-Desvoidy, 1830**

﻿*Stomina ﻿﻿﻿caliendrata* (Rondani, 1862)

= *Morphomyia ﻿﻿﻿caliendrata* Rondani, in Séguy 1930a: 143

Séguy 1930a, **MA**, from M’Rirt to Hajeb, **HA**, Kasba Taguendaft (Gounfada); Mouna 1998; **HA** (Massif Toubkal) – PCPT

﻿***Thelaira* Robineau-Desvoidy, 1830**

﻿*Thelaira ﻿﻿﻿haematodes* (Meigen, 1824)^63^

= *Phoenicella ﻿﻿﻿haematodes* Meigen: Séguy 1930a: 142

Séguy 1930a, **HA**, Arround; Mouna 1998

﻿***Uclesia* Girschner, 1901**

﻿*Uclesia ﻿﻿﻿fumipennis* Girschner, 1901

Séguy 1934b; Herting and Dely-Draskovits 1993; Mouna 1998; **HA** (Marrakech) – MISR

﻿***Voria* Robineau-Desvoidy, 1830**

﻿*Voria ﻿﻿﻿ruralis* (Fallén, 1810)

Jourdan 1935c

﻿***Wagneria* Robineau-Desvoidy, 1830**

﻿*Wagneria ﻿﻿﻿dilatata* Kugler, 1977

Kugler 1977

##### Exoristinae

Acemyini

﻿***Ceracia* Rondani, 1865**

﻿*Ceracia ﻿﻿﻿mucronifera* Rondani, 1865

= *Myothyria ﻿benoisti* (Mesnil), in Mesnil 1959: 20

Mesnil 1959, **MA**, Forêt Maâmora near Tiflet; Herting and Dely-Draskovits 1993; Cerretti and Ziegler 2004

##### Blondeliini

﻿***Compsilura* Bouché, 1834**

﻿*Compsilura ﻿﻿﻿concinnata* (Meigen, 1824)

IOBC-list 1 (1956); Hérard and Fraval 1980

﻿***Istocheta* Rondani, 1859**

﻿*Istocheta ﻿﻿﻿cinerea* (Macquart, 1850)

Herting 1960

﻿*Istocheta ﻿﻿﻿longicornis* (Fallén, 1810)

= *Latigena ﻿﻿﻿longicornis* Fallén, in Séguy 1953a: 91

Séguy 1953a, **AP**, Forêt Zaers

﻿***Lomachantha* Rondani, 1859**

﻿*Lomachantha ﻿﻿﻿parra* Rondani, 1859

Efetov and Tarmann 1999

﻿***Robinaldia* Herting, 1983**

﻿*Robinaldia ﻿﻿﻿angustata* (Villeneuve, 1933)

Herting and Dely-Draskovits 1993; Tschorsnig and Herting 1994

﻿***Zaira* Robineau-Desvoidy, 1830**

﻿*Zaira ﻿﻿﻿cinerea* (Fallén, 1820)

Ebejer et al. 2019, **Rif**, Aïn Jdioui (Tahaddart, 8 m)

##### Eryciini

﻿***Alsomyia* Brauer & Bergenstamm, 1891**

﻿*Alsomyia ﻿﻿﻿olfaciens* (Pandellé, 1896)

IOBC-List 12 (1993)

﻿***Amphicestonia* Villeneuve, 1939**

﻿*Amphicestoniadispar* (Villeneuve, 1922)

Herting and Dely-Draskovits 1993; Cerretti and Ziegler 2004; **MA** (Ifrane) – PCPT

﻿***Aplomyia* Robineau-Desvoidy, 1830**

﻿*Aplomyia ﻿﻿﻿confinis* (Fallén, 1820)

Ebejer et al. 2019, **Rif**, Dardara (484 m)

﻿***Carcelia* Robineau-Desvoidy, 1830**

﻿*Carcelia ﻿﻿﻿dilaticornis* Mesnil, 1950

Mesnil 1950

﻿*Carcelia ﻿﻿﻿iliaca* (Ratzeburg, 1840)63

Mouna 1998

﻿*Carcelia ﻿﻿﻿lucorum* (Meigen, 1824)*

**HA**

﻿***Drino* Robineau-Desvoidy, 1863**

﻿*Drino ﻿﻿﻿atropivora* (Robineau-Desvoidy, 1830)

= ﻿*Sturmia ﻿﻿﻿atropivora* Robineau-Desvoidy, in Bléton and Fieuzet 1939: 64

De Lépiney and Mimeur 1932; Bléton and Fieuzet 1939, **MA**, Fès; Mouna 1998; Tschorsnig 2017; **AP** (Rabat), **MA** (Bel Lakssiri) – MISR

﻿*Drino ﻿﻿﻿galii* (Brauer & Bergenstamm, 1891)*

**HA**, **AA**

﻿*Drino ﻿﻿﻿gilva* (Hartig, 1838)63

= ﻿*Sturmia ﻿﻿﻿gilva* Hartig

Mouna 1998 – MISR (no locality given)

﻿*Drino ﻿﻿﻿imberbis* (Wiedemann, 1830)63

Rungs 1954; Grabener 2017

﻿*Drino ﻿﻿﻿inconspicua* (Meigen, 1830)

Séguy 1935a, **AP**, Sehoul (Rabat); Bléton and Fieuzet 1939, **MA**, Dayat Achleff; Mouna 1998; **MA** (Meknès, Béni Mellal) – MISR

﻿*Drino ﻿maroccana* Mesnil, 1951

De Lépiney 1930; De Lépiney and Mimeur 1932 (probably misidentified as ﻿*Sturmia ﻿﻿﻿inconspicua*); Mesnil 1951; Herting and Dely-Draskovits 1993; Ziegler 2011

﻿*Drinotriplaca* Herting, 1979

Herting 1979, **AP**, Rabat

﻿*Drino ﻿﻿﻿vicina* (Zetterstedt, 1849)

= ﻿*Sturmia ﻿﻿﻿vicina* Zetterstedt, 1849

De Lépiney and Mimeur 1932; Bouclier-Maurin 1923; **AP** (Rabat) – MISR

﻿***Gymnophryxe* Villeneuve, 1922**

﻿*Gymnophryxe ﻿﻿﻿carthaginiensis* (Bischof, 1900)

Mesnil 1956

﻿***Nilea* Robineau-Desvoidy, 1863**

﻿*Nilea ﻿﻿﻿innoxia* Robineau-Desvoidy, 1863

Bléton and Fieuzet 1939

﻿***Phryxe* Robineau-Desvoidy, 1830**

﻿*Phryxe ﻿caudata* (Rondani, 1859)

Biliotti 1956; El Yousfi 1994; IOBC-list 11 (1989)

﻿*Phryxe ﻿﻿﻿setifacies* (Villeneuve, 1910)

IOBC-list 11 (1989); IOBC-list 12 (1993)

﻿*Phryxe ﻿﻿﻿vulgaris* (Fallén, 1810)

Séguy 1953a, **MA**, Tamrabta (1700 m) – PCPT (**HA**, Marrakech, Imlil, S Asni)

﻿***Ptesiomyia* Brauer & Bergenstamm, 1893**

﻿*Ptesiomyia ﻿﻿﻿microstoma* Brauer & Bergenstamm, 1893

Séguy 1953a, **AP**, Rabat; **EM** (Mte des Béni Snassen, Taforalt), **MA** (Béni Mellal, Bin-el-Ouidane; Meknès, Ifrane (NPI)), **HA** (Marrakech, Oukaimeden) – PCPT

﻿***Senometopia* Macquart, 1834**

﻿*Senometopia ﻿﻿﻿separata* (Rondani, 1859)

Hérard and Fraval 1980

﻿***Tryphera* Meigen, 1838**

﻿*Tryphera ﻿﻿﻿lugubris* (Meigen, 1824)

IOBC-List 1 (1956)

##### Ethillini

﻿***Atylomyia* Brauer, 1898**

﻿*Atylomyia ﻿﻿﻿﻿﻿﻿albifrons* Villeneuve, 1911

= ﻿*Atylomyia ﻿﻿﻿rungsi* Mesnil, in Mesnil 1962: 778

Mesnil 1962, **AA** (near Agadir), Aït Melloul; Herting and Dely-Draskovits 1993

##### Exoristini

﻿***Bessa* Robineau-Desvoidy, 1863**

﻿*Bessa ﻿﻿﻿parallela* (Meigen, 1824)

Séguy 1935a (probably misidentified as ﻿*Bessaselecta*); Tschorsnig 2017

﻿***Chetogena* Rondani, 1856**

﻿*Chetogena ﻿﻿﻿filipalpis* Rondani, 1859

Ebejer et al. 2019, **Rif**, Aïn Jdioui (Tahaddart, 8 m); **MA** (Fès, Sidi Harazem, Ifrane, Forêt de Cèdres) – PCPT

﻿*Chetogena ﻿﻿﻿mageritensis* (Villeneuve & Mesnil, 1936)

Herting and Dely-Draskovits 1993

﻿*Chetogena ﻿﻿﻿media* Rondani, 1859*

**MA**

﻿*Chetogena ﻿﻿﻿nigrofasciata* (Strobl, 1902)

= ﻿*Chetogena ﻿﻿﻿repanda* (Mesnil, 1939), in Herting and Dely-Draskovits 1993 (type locality: Skel): 17

Gheibi et al. 2010

﻿*Chetogena ﻿﻿﻿obliquata* (Fallén, 1810)

De Lépiney and Mimeur 1932; Herting 1960; IOBC-list 12 (1993); **HA** (Marrakech, Oukaimeden) – PCPT

﻿***Exorista* Meigen, 1803**

﻿*Exorista ﻿﻿﻿deligata* Pandellé, 1896

Mesnil 1946, **AP**, Sidi Taibi near Kénitra; Herting and Dely-Draskovits 1993; Cerretti and Ziegler 2004; Gheibi et al. 2010

﻿*Exorista ﻿﻿﻿grandis* (Zetterstedt, 1844)

Ebejer et al. 2019, **Rif**, Dardara (484 m)

﻿*Exorista ﻿﻿﻿larvarum* (Linnaeus, 1758)

Mouna 1998

﻿*Exorista ﻿﻿﻿nova* (Rondani, 1859)

Tschorsnig 2017

﻿*Exorista ﻿﻿﻿rendina* (Herting, 1975)*

**AA**

﻿*Exorista ﻿﻿﻿segregata* (Rondani, 1859)

Mouna 1998; **AA** (Agadir, Oulma) – PCPT

##### Goniini

﻿***Anurophylla* Villeneuve, 1938**

﻿*Anurophylla ﻿﻿﻿aprica* (Villeneuve, 1912)*

**MA**

﻿***Baumhaueria* Meigen, 1838**

﻿*Baumhaueria ﻿﻿﻿goniaeformis* (Meigen, 1824)

De Lépiney and Mimeur 1932; **AP** (Maâmora) – MISR; **HA** (Marrakech, Oukaimeden) – PCPT

﻿***Blepharipa* Rondani, 1856**

﻿*Blepharipa ﻿﻿﻿pratensis* (Meigen, 1824)*

**HA**

﻿***Ceratochaetops* Mesnil, 1970**

﻿*Ceratochaetops ﻿﻿﻿triseta* (Villeneuve, 1922)

= *Ceratochoeta ﻿﻿﻿triseta* Villeneuve, in Rungs 1940: 15

Rungs 1940, **MA** (Cédraie); Mouna 1998; **MA** (Khénifra, El-Herri, Ifrane (NPI), Meknès), **HA** (Marrakech, 8 km N Ouirgane) – PCPT

﻿***Ceromasia* Rondani, 1856**

﻿*Ceromasia ﻿﻿﻿rubrifrons* (Macquart, 1834)

IOBC-list 11 (1989); IOBC-list 12 (1993); **HA** (Marrakech, Ouirgane, Tagadirt, S Asni) – PCPT

﻿***Clemelis* Robineau-Desvoidy, 1863**

﻿*Clemelis ﻿﻿﻿pullata* (Meigen, 1824)

IOBC-list 13 (1997)

﻿***Gaedia* Meigen, 1838**

﻿*Gaedia ﻿﻿﻿connexa* Meigen, 1824

Séguy 1953a, **EM**, Berkane

﻿***Gonia* Meigen, 1803**

﻿*Gonia ﻿﻿﻿aterrima* Tschorsnig, 1991

Tschorsnig 1991

﻿*Gonia ﻿﻿﻿atra* Meigen, 1826

Séguy 1930a, **MA**, Tizi-n’Bouftene, between Azrou and Ras el Ma, Forêt Azrou, **HA**, Arround (Skoutana), Jebel Likount, Asni; Mouna 1998; Grabener 2017; **MA** (Tighassaline, El-Herri, Aïn Leuh-Tagounit, Meknès, Ifrane (NPI)), **HA** (40 km SW Ouarzazate, Marrakech, Ouirgane, Lakhdar, N Demnate, Oukaimeden), **AA** (Agadir, Oulma) – PCPT

﻿*Gonia ﻿﻿﻿bimaculata* Wiedemann, 1819

= ﻿*Gonia ﻿﻿﻿cilipeda* Rondani, in Séguy 1953a: 91

Séguy 1930a, **AP**, Rabat, **MA**, Tizi-s’Tkrine, Tizi-n’Bouftene, between Azrou and Ras el Ma, Forêt Azrou, Berkane, **HA**, Arround (Skoutana), Jebel Likount, Asni, Ouaouzert (Glaoua); Séguy 1953a, **AP**, Temara; **AP** (Rabat) – MISR; **HA** (S Asni Ouirgane, Marrakech), **AA** (80 km N Taroudant, Aoulouz), **AA** (15 km NW Zagora) – PCPT

﻿*Goniacapitata* (De Geer, 1776)

Mouna 1998; **MA** (Ifrane) – MISR

﻿*Goniamaculipennis* Egger, 1862*

**MA**

﻿*Gonia ﻿﻿﻿ornata* Meigen, 1826

Séguy 1953a, **AP**, Rabat, **MA**, Ifrane, **SA**, Kelaâ M’Goum; **MA** (Ifrane, Forêt de Cèdres), **HA** (Oukaimeden (2600 m), Marrakech) – PCPT

﻿*Gonia ﻿﻿﻿vacua* Meigen, 1826*

**MA**

﻿***Pales* Robineau-Desvoidy, 1830**

﻿*Pales ﻿﻿﻿pavida* (Meigen, 1824)

IOBC-list 1 (1956); Cerretti 2005; **MA** (Fès, Sidi Harazem) – PCPT

﻿***Platymya* Robineau-Desvoidy, 1830**

﻿*Platymya ﻿﻿﻿antennata* (Brauer & Bergenstamm, 1891)

Efetov and Tarmann 1999

﻿***Pseudogonia* Brauer and von Bergenstamm, 1889**

﻿*Pseudogonia ﻿﻿﻿rufifrons* (Wiedemann, 1830)

De Lépiney and Mimeur 1932; IOBC-list 1 (1956); Mouna 1998; Tschorsnig 2017; Grabener 2017; **AA** (S Tafraoute, Aït Mansur, Agadir, Oulma) – PCPT

﻿***Sturmia* Robineau-Desvoidy, 1830**

﻿*Sturmia ﻿﻿﻿bella* (Meigen, 1824)

Stefanescu et al. 2012; Tschorsnig 2017

##### Winthemiini

﻿***Nemorilla* Rondani, 1856**

﻿*Nemorilla ﻿maculosa* (Meigen, 1824)

Kozlovsky and Rungs 1933; Brémond and Rungs 1938 [as *N.floralis*; probable misidentification]; IOBC list 1 (1956); Mouna 1998; **EM** (Oujda, Col de Jerada) – PCPT

##### Phasiinae

Cylindromyiini

﻿***Besseria* Robineau-Desvoidy, 1830**

﻿*Besseria ﻿﻿﻿lateritia* (Meigen, 1824)*

**HA**

﻿***Cylindromyia* Meigen, 1803**

﻿*Cylindromyia ﻿﻿﻿bicolor* (Olivier, 1812)

Séguy 1930a, **AP**, Rabat; Mouna 1998

﻿*Cylindromyia ﻿﻿﻿brassicaria* (Fabricius, 1775)

Séguy 1930a, **EM**, Soufouloud, **MA**, Aharmoumou, Berrechid, Meknès, Sidi Bettache, Berkane, **HA**, Tenfecht, Ouaounzert, Marrakech, Asni; Séguy 1935a, **AP**, Gharb; Séguy 1941a, **HA**, Jebel Ayachi; Séguy 1949, **SA**, Guelmim; Dupuis 1963; Mouna 1998; **MA** (Sefrou) – MISR; **AP** (Essaouira, 4 km E Ounara), **HA** (10 km W Chichaoua, Oukaimeden), **AA** (140 km E Agadir, Aoulouz, Tizi-n’Tichka) – PCPT

﻿*Cylindromyia ﻿﻿﻿intermedia* Meigen, 1824

Becker and Stein 1913, **Rif**, Tanger; **HA** (Ouirgane, Imlil, Tizi-n’Test), **AA** (10 km SE Ouarzazate (﻿oasis), Taroudant) – PCPT

﻿*Cylindromyia ﻿maroccana* Tschorsnig, 1997

Tschorsnig 1997, **HA**, Ouirgane, Tagadirt (1000 m)

﻿*Cylindromyia ﻿﻿﻿pilipes* Loew, 1844

Becker and Stein 1913, **Rif**, Tanger; Herting and Dely-Draskovits 1993; Gilasian et al. 2013

﻿***Phania* Meigen, 1824**

﻿*Phania ﻿﻿﻿albisquama* (Villeneuve, 1924)*

**MA**

##### Gymnosomatini

﻿***Clytiomya* Rondani, 1861**

﻿*Clytiomya ﻿﻿﻿continua* (Panzer, 1798)

= *Clytiomyia ﻿﻿﻿dalmatica* Robineau-Desvoidy, in Séguy 1935a: 119

Séguy 1935a, **AP**, Gharb; Mouna 1998; **AP** (Rabat) – MISR

﻿*Clytiomya ﻿﻿﻿sola* (Rondani, 1861)

Séguy 1935a; Dupuis 1963; **MA** (Khénifra, Tighassaline) – PCPT

﻿***Ectophasia* Townsend, 1912**

﻿*Ectophasia ﻿﻿﻿crassipennis* (Fabricius, 1794)

= ﻿*Phasia ﻿﻿﻿crassipennis* Fabricius, in Séguy 1930a: 141

Séguy 1930a, **MA**, Aïn Leuh; Mouna 1998

﻿***Eliozeta* Rondani, 1856**

﻿*Eliozetahelluo* (Fabricius, 1805)

= *Clytiomyia ﻿helluo* Fabricius, in Séguy 1935: 119

Séguy 1930a, **MA**, Meknès (Aïn Sferguila); Jourdan 1935; Séguy 1935a, **AP**, Gharb; Thompson 1950; Dupuis 1963; Mouna 1998; Tschorsnig 2017

﻿***Gymnosoma* Meigen, 1803**

﻿*Gymnosoma ﻿﻿﻿carpocoridis* Dupuis, 1961

Dupuis 1963; Herting and Dely-Draskovits 1993

﻿*Gymnosoma ﻿﻿﻿clavatum* (Rohdendorf, 1947)

Dupuis 1963; Grabener 2017; **MA** (Meknès, Ifrane (NPI)), **AA** (140 km E Agadir, Aoulouz), **AA** (80 km S Zagora, Oued Draa, Mhamid) – PCPT

﻿*Gymnosoma ﻿﻿﻿dolycoridis* Dupuis, 1960

Dupuis 1963; **MA** (Fès, Sidi Harazem) – PCPT

﻿*Gymnosoma ﻿﻿﻿rotundatum* Linnaeus, 1758^64^

Becker and Stein 1913, **Rif**, Tanger; Séguy 1930a, **Rif**, Tanger, **AP**, Mogador, **MA**, Tizi-s’Tkrine, Sidi Bettache, Moulay Aïn Djemine, **HA**, Asni; Séguy 1934b; Séguy 1935a; Thompson 1950; Mouna 1998; **AP** (Mogador), **MA** (Maghrawa) – MISR; **MA** (Meknès, Ifrane (NPI)) – PCPT

﻿*Gymnosoma ﻿﻿﻿rungsi* (Mesnil, 1952)

= *Rhodogynerungsi* (Mesnil), in Mesnil 1952: 151

Mesnil 1952, **AP**, Rabat; Dupuis 1963; Herting and Dely-Draskovits 1993

##### Leucostomatini

﻿***Clairvillia* Robineau-Desvoidy, 1830**

﻿*Clairvillia ﻿﻿﻿biguttata* (Meigen, 1824)*

**HA**

﻿***Dionomelia* Kugler, 1978**

﻿*Dionomeliahennigi* Kugler, 1978*

**SA**

﻿***Leucostoma* Meigen, 1803**

﻿*Leucostoma ﻿﻿﻿abbreviatum* Herting, 1971

Ziegler 2012

﻿*Leucostoma ﻿﻿﻿crassum* Kugler, 1966*

**HA**

﻿*Leucostoma ﻿﻿﻿obsidianum* (Wiedemann, 1830)

Ebejer et al. 2019, **AA**, Ziz river (10 km S of Errachidia, 1008 m)

﻿*Leucostomatetraptera* (Meigen, 1824)

Dupuis 1953; Ebejer et al. 2019, **Rif**, Barrage Smir (27 m)

﻿***Weberia* Robineau-Desvoidy, 1830**

﻿*Weberia ﻿﻿﻿digramma* (Meigen, 1824)*

**AA**

##### Phasiini

﻿***Elomya* Robineau-Desvoidy, 1830**

﻿*Elomyalateralis* (Meigen, 1824)

Séguy 1930a, **AP**, From Zarjoulea to Larache, **MA**, Berkane; Dupuis 1952; Dupuis 1963; Herting and Dely-Draskovits 1993; Mouna 1998; Cerretti and Ziegler 2004; **MA** (Khénifra, Tighassaline, Meknès, Ifrane (NPI)), **HA** (Marrakech, Ouirgane) – PCPT

﻿***Phasia* Latreille, 1804**

﻿*Phasia ﻿﻿﻿mesnili* (Draber-Monko, 1965)

Sun and Marshall 2003, **HA**; **AP** (10 km E Essaouira), **AA** (S Tafraoute, Aït Mansur, S Aït-Baha) – PCPT

﻿*Phasia ﻿﻿﻿obesa* (Fabricius, 1798)

Sun and Marshall 2003, **HA**, Asni

﻿*Phasia ﻿﻿﻿pusilla* Meigen, 1824

Dupuis 1963; Sun and Marshall 2003, **MA**

﻿*Phasia ﻿﻿﻿subcoleoptrata* (Linnaeus, 1767)

Dupuis 1963; Herting and Dely-Draskovits 1993; Sun and Marshall 2003, **MA**; Cerretti and Ziegler 2004

﻿*Phasia ﻿﻿﻿venturii* (Draber-Monko, 1965)

Sun and Marshall 2003, **HA**, Asni; **AP** (Essaouira, 4 km E Ounara), **AA** (11 km NW Taliouine, 10 km SE Aït-Ourir) – PCPT

##### Trichopodini

﻿***Trichopoda* Berthold, 1827**

﻿*Trichopoda ﻿﻿﻿pennipes* (Fabricius, 1794)

Ebejer et al. 2019, **Rif**, Tahaddart (8 m)

##### Xystini

﻿***Xysta* Meigen, 1824**

﻿*Xysta ﻿﻿﻿holosericea* (Fabricius, 1805)*

**HA**

##### Tachininae

Graphogastrini

﻿***Graphogaster* Rondani, 1868**

﻿*Graphogaster ﻿﻿﻿vestita* Rondani, 1868*

**MA**

﻿***Phytomyptera* Rondani, 1845**

﻿*Phytomyptera ﻿﻿﻿nigrina* (Meigen, 1824)

= ﻿*Phytomyptera ﻿﻿﻿nitidiventris* Rondani, in Bléton and Fieuzet 1939: 64

Bléton and Fieuzet 1939; Mouna 1998

##### Leskiini

﻿***Aphria* Robineau-Desvoidy, 1830**

﻿*Aphria ﻿﻿﻿longirostris* (Meigen, 1824)

Ebejer et al. 2019, **Rif**, Jnane Niche (46 m)

﻿***Bithia* Robineau-Desvoidy, 1863**

﻿*Bithia ﻿﻿﻿demotica* (Egger, 1861)

Tschorsnig and Bläsius 2001; IOBC-list 14 (2005); Tschorsnig 2017

﻿*Bithia ﻿﻿﻿modesta* (Meigen, 1824)

Tschorsnig and Bläsius 2001; Tschorsnig 2017

##### Linnaemyini + Ernestiini

﻿***Gymnochaeta* Robineau-Desvoidy, 1830**

﻿*Gymnochaeta ﻿﻿﻿viridis* Fallén, 1810

Séguy 1930a, **HA**, Arround (Skoutana); Mouna 1998

﻿***Linnaemya* Robineau-Desvoidy, 1830**

﻿*Linnaemya ﻿﻿﻿soror* Zimin, 1954*

**MA**, **HA**, **AA**

﻿***Loewia* Egger, 1856**

﻿*Loewia ﻿﻿﻿setibarba* Egger, 1856

Becker and Stein 1913, **Rif**, Tanger

﻿***Panzeria* Robineau-Desvoidy, 1830**

﻿*Panzeria ﻿﻿﻿castellana* (Strobl, 1906)*

**HA**

﻿*Panzeria ﻿﻿﻿nemorum* (Meigen, 1824)*

**MA**

﻿***Zophomyia* Macquart, 1835**

﻿*Zophomyia ﻿﻿﻿temula* (Scopoli, 1763)

Séguy 1930a, **AP**, Casablanca, **MA**, Meknès; Mouna 1998; **MA** (Khénifra, Tighassaline, Meknès, Ifrane (NPI)) – PCPT

##### Macquartiini

﻿***Macquartia* Robineau-Desvoidy, 1830**

﻿*Macquartia ﻿﻿﻿chalconota* (Meigen, 1824)

Ebejer et al. 2019, **Rif**, Smir lagoon; **HA** (Marrakech, Ouirgane, Tizi-n’Test), **AA** (Taroudant) – PCPT

﻿*Macquartia ﻿﻿﻿﻿macularis* Villeneuve, 1926

Herting and Dely-Draskovits 1993

﻿*Macquartia ﻿﻿﻿tessellum* (Meigen, 1824)

= ﻿*Macquartia ﻿﻿﻿brevicornis* Macquart, in Séguy 1941d: 23

Séguy 1941d, **MA**, Meknès, **HA**, Tizi-n’Test; Mouna 1998; **HA** (Imlil, S Asni, Tizi-n’Test), **AA** (Taroudant) – PCPT

##### Megaprosopini

﻿***Microphthalma* Macquart, 1844**

﻿*Microphthalma ﻿﻿﻿europaea* Egger, 1860

Ebejer et al. 2019, **AA**, Ziz river (30 km N of Erfoud, 894 m)

##### Minthoini

﻿***Hyperaea* Robineau-Desvoidy, 1863**

﻿*Hyperaeafemoralis* (Meigen, 1824)

Herting and Dely-Draskovits 1993

﻿***Mintho* Robineau-Desvoidy, 1830**

﻿*Minthocompressa* (Fabricius, 1787)

Walker 1849; Herting and Dely-Draskovits 1993

﻿*Mintho ﻿﻿﻿rufiventris* (Fallén, 1817)

= ﻿*Mintho ﻿﻿﻿praeceps* (Scopoli, 1763), in Séguy 1930a: 143; Séguy 1953a: 91

Séguy 1930a, **AP**, Rabat, Casablanca, **MA**, Meknès; Séguy 1953a, **SA**, El Aïoun du Draa; Séguy 1949a, **AA**, Tata; Mouna 1998; Dawah 2011; **AP** (Rabat, Salé), **MA** (Meknès) – MISR; **MA** (Meknès, Ifrane (NPI)), **AA** (11 km NW Taliouine) – PCPT

﻿***Minthodes* Brauer & Bergenstamm, 1889**

﻿*Minthodes ﻿﻿﻿numidica* Villeneuve, 1932*

**AA**

﻿*Minthodes ﻿﻿﻿setifacies* Mesnil, 1939

= ﻿Minthodes (Myxominthodes) setifacies Mesnil, in Mesnil 1939: 211

Mesnil 1939, **MA**, Forêt Azrou; Herting and Dely-Draskovits 1993

﻿***Plesina* Meigen, 1838**

﻿*Plesina ﻿﻿﻿phalerata* (Meigen, 1824)

Herting and Dely-Draskovits 1993; Cerretti and Tschorsnig 2008

﻿***Pseudomintho* Brauer & Bergenstamm, 1889**

﻿*Pseudomintho ﻿﻿﻿diversipes* (Strobl, 1889)

Ebejer et al. 2019, **Rif**, Moulay Abdelsalam (PNPB, 965 m); **AP** (Essaouira, 4 km E Ounara) – PCPT

##### Siphonini

﻿***Actia* Robineau-Desvoidy, 1830**

﻿*Actia ﻿﻿﻿infantula* (Zetterstedt, 1844)

Ebejer et al. 2019, **Rif**, Tanger (Douar Dakchire forest, 320 m)

﻿***Peribaea* Robineau-Desvoidy, 1863**

﻿*Peribaea ﻿﻿﻿﻿﻿﻿﻿﻿﻿﻿apicalis* Robineau-Desvoidy, 1863

Ebejer et al. 2019, **Rif**, Dardara (484 m)

﻿*Peribaea ﻿﻿﻿tibialis* (Robineau-Desvoidy, 1851)

Draber-Mońko 2011

﻿***Siphona* Meigen, 1803**

﻿*Siphona ﻿﻿﻿geniculata* (De Geer, 1776)^65^

Séguy 1930a, **HA**; Mouna 1998; **HA** (Vallée Oued N’fis) – MISR

﻿*Siphona ﻿maroccana* Cerretti & Tschorsnig, 2007

Cerretti and Tshorsnig 2007, **HA**, Asif Mellah, W Tizi-n’Tichka

﻿*Siphona ﻿﻿﻿variata* Andersen, 1982

Ebejer et al. 2019, **Rif**, Sidi Yahia Aârab (377 m), Oued Kbir (PNPB, 157 m)

##### Tachinini

﻿***Germaria* Robineau-Desvoidy, 1830**

﻿*Germaria ﻿﻿﻿barbara* Mesnil, 1963*

**HA**

﻿***Peleteria* Robineau-Desvoidy, 1830**

﻿*Peleteria ﻿﻿﻿ruficornis* (Macquart, 1835)*

**HA**, **AA**

﻿***Tachina* Meigen, 1803**

﻿*Tachina ﻿﻿﻿corsicana* (Villeneuve, 1931)*

**HA**, **AA**

﻿*Tachina ﻿﻿﻿fera* (Linnaeus, 1761)

Becker and Stein 1913, **Rif**, Tanger; Séguy 1930a, **MA**, Tizi-s’Tkrine, Forêt Tiffert, Sidi Bettache, **HA**, Arround (Skoutana), Jebel Likount; Séguy 1953a, **MA**, Ifrane (1650 m); Mouna 1998; **Rif** (fir forest of Talassemtane), **AP** (Dradek) – MISR (**MA**, Meknès); **MA** (Ifrane (NPI)), Béni Mellal, Bin-el-Ouidane), **HA** (Marrakech, Ouirgane) – PCPT

﻿*Tachina ﻿﻿﻿magnicornis* (Zetterstedt, 1844)

Séguy 1930a, **MA**, Ras el Ksar, Aïn Leuh, Sidi Bettache; Mouna 1998; **MA** (Béni Mellal, El Ksiba, 5 km N) – PCPT

﻿*Tachina ﻿﻿﻿praeceps* Meigen, 1824

**HA**, **AA**

##### Triarthriini

﻿***Lissoglossa* Villeneuve, 1912**

﻿*Lissoglossabequaerti* Villeneuve, 1912

Herting and Dely-Draskovits 1993

##### New records for Morocco

The data added under the abbreviation “PCPT” (for “personal communication Hans-Peter Tschorsnig”) are based on (unpublished) material which was identified by HPT for several collectors (M. Hauser, M. Hradský, C.F. Kassebeer, U. Koschwitz, J.A.W. Lucas, G. Miksch, H. and T. v. Oorschot, C. Schmid-Egger, M. Schwarz, K. Špatenka, V. Vrabec) during the last ~ 30 years. Usually only a few duplicate specimens were retained in the collection of SMNS. The main part was sent back to the collectors, but the data were noted by HPT on handwritten lists.

﻿*Estheria﻿iberica* Tschorsnig, 2003

Middle Atlas: Ifrane, National Park of Ifrane, 19.ix.1989, K. Špatenka leg, 1 specimen, PCPT.

﻿*Athrycia ﻿trepida* (Meigen, 1824)

Middle Atlas: Meknès; Ifrane, National Park of Ifrane, 22.v.1995, C. Kassebeer leg., 1 specimen, PCPT.

﻿*Eriothrix ﻿﻿﻿rufomaculata* (De Geer, 1776)

Middle Atlas: Ifrane, Forêt de Cèdres, 29.iv.1999, V. Vrabec leg., 1 specimen, PCPT.

High Atlas: Marrakech, Oukaimeden, 19.v.1995, C. Kassebeer leg., 7 specimens, PCPT.

﻿*Kirbya ﻿moerens* (Meigen, 1830)

Middle Atlas: Ifrane, Forêt de Cèdres, 29.iv.1999, V. Vrabec leg., 1 specimen, PCPT.

﻿*Carcelia ﻿lucorum* (Meigen, 1824)

High Atlas: Marrakech, Imlil, S Asni, 24.iii.1995, C. Kassebeer leg., 1 specimen, PCPT.

﻿*Drino ﻿galii* (Brauer & Bergenstamm, 1891)

High Atlas: Marrakech, Ouirgane, 24.iii.1995, C. Kassebeer leg., 1 specimen, PCPT.

Anti Atlas: 11 km NW Taliouine; Agadir, Ameskroud, 17.v.1995, C. Kassebeer leg., 2 specimens, PCPT.

﻿*Chetogena ﻿media* Rondani, 1859

Middle Atlas: Béni Mellal, El Ksiba, 30.iii.1995, C. Kassebeer leg., 1 specimen, PCPT.

﻿*Exorista ﻿rendina* (Herting, 1975)

Anti Atlas: 11 km NW Taliouine, 15.iii.1997, M. Hauser leg., 1 male in SMNS; 10 km NE Tafraoute, 14.iii.1997, G. Miksch leg., 1 male in SMNS, PCPT.

﻿*Anurophylla ﻿aprica* (Villeneuve, 1912)

Middle Atlas: Béni Mellal, Afourer, 28.iii.1995, C. Kassebeer leg., 1 female in SMNS, PCPT.

﻿*Blepharipa ﻿pratensis* (Meigen, 1824)

High Atlas: Tizi-n’Test, 2000 m, 21.v.1995, M. Hauser leg., 2 specimens, PCPT.

Anti Atlas: Taroudant, PCPT.

﻿*Goniamaculipennis* Egger, 1862

Middle Atlas: Ifrane, Forêt de Cèdres, 29.iv.1999, V. Vrabec leg., 1 female in SMNS, PCPT.

﻿*Gonia ﻿vacua* Meigen, 1826

Middle Atlas: Meknès; Ifrane, National Park of Ifrane, 29.iii.1995 and 22.v.1995, C. Kassebeerleg., 2 specimens, PCPT.

﻿*Besseria ﻿lateritia* (Meigen, 1824)

High Atlas: SE Asni, Imlil, 23.v.1995, M. Hauser leg., 2 specimens; Marrakech, Lakhdar, N Demnate, 27.iii.1995, C. Kassebeer leg., 1 specimen, PCPT.

﻿*Phania ﻿albisquama* (Villeneuve, 1924)

Middle Atlas: Ifrane, Forêt de Cèdres, 29.iv.1999, V. Vrabec and L. Vrabcová leg., 1 specimen, PCPT.

﻿*Clairvillia ﻿biguttata* (Meigen, 1824)

High Atlas: Marrakech, Lakhdar, N Demnate, 27.iii.1995, C. Kassebeer leg, 1 specimen, PCPT.

﻿*Dionomelia ﻿hennigi* Kugler, 1978

**SA**: Boujdour, 8.v.1999, V. Vrabec leg, 1 male in SMNS, PCPT.

﻿*Leucostoma ﻿crassum* Kugler, 1966

High Atlas: Tizi-n’Test, pass 23.vi.1996, U. Koschwitz leg., 1 male in SMN, PCPT.

﻿*Weberia ﻿digramma* (Meigen, 1824)

Anti Atlas: 10 km NW Aït-Baha, PCPT.

﻿*Xysta ﻿holosericea* (Fabricius, 1805)

High Atlas: Marrakech, Lakhdar, N Demnate, 27.iii.1995, C. Kassebeer leg, 1 specimen, PCPT.

﻿*Linnaemya ﻿soror* Zimin, 1954

Middle Atlas: Béni Mellal, El Ksiba, 5 km N; Béni Mellal, Afourer; Khénifra, Tighassaline; Meknès; National Park of Ifrane.

High Atlas: Marrakech, Ouirgane; Marrakech, Tagaddirt, S Asni; Marrakech, Lakhdar, N Demnate.

Anti Atlas: 11 km NW Taliouine, all C. Kassebeer leg., 57 specimens (collected between 25.iii. and 23.v.1995), PCPT.

﻿*Panzeria ﻿castellana* (Strobl, 1906)

High Atlas: Marrakech, Ouirgane, 26.iii.1995, C. Kassebeer leg., 1 specimen, PCPT.

﻿*Panzeria﻿nemorum* (Meigen, 1824)

Middle Atlas: Meknès; National Park of Ifrane, 22.v.1995, C. Kassebeer leg., 1 specimen, PCPT.

﻿*Graphogaster﻿vestita* Rondani, 1868

Middle Atlas: Ifrane, Forêt de Cèdres, 29.iv.1999, V. Vrabec leg., 1 specimen, PCPT.

﻿*Minthodes ﻿numidica* Villeneuve, 1932

Anti Atlas: S Aït-Baha, PCPT.

﻿*Germaria ﻿barbara* Mesnil, 1963

High Atlas: S Tizi-n’Test, 1900 m, PCPT.

﻿*Peleteria﻿ruficornis* (Macquart, 1835)

High Atlas: Marrakech, Ouirgane; Marrakech, Tagaddirt, S Asni; Tizi-n’Test.

Anti Atlas: Taroudant, all C. Kassebeer leg., 6 specimens (collected between 28.ix.1994 and 1.iv.1995), PCPT.

﻿*Tachina﻿corsicana* (Villeneuve, 1931)

High Atlas: Marrakech, Oukaimeden, 19.v.1995, C. Kassebeer leg., 1 specimen; Tizi-n’Test. Anti Atlas: Taroudant, 21.v.1995, M. Hauser leg., 2 specimens, PCPT.

﻿*Tachina ﻿﻿﻿praeceps* Meigen, 1824

High Atlas: Marrakech, Oukaimeden, 2500 m, 27.vi.1987, M. Schwarz leg., 1 specimen; Tizi-n’Test.

Anti Atlas: Taroudant, 29.vi.1987, M. Schwarz leg., 1 specimen, PCPT.

## Footnotes

﻿ Dicranomyia ﻿ majuscula Pierre, is a doubtful species of which the identity is not known. There is only one record, from Pierre 1924a, all other citations come from this record.

﻿ Tipula (﻿ Emodotipula) ﻿ obscuriventris is widespread in the western Palaearctic, but reaches its south-western limits in the the Spanish Pyrenees. Therefore, it is very unlikely that ﻿ obscuriventris actually occurs in Morocco, disregarding the references of Oosterbroek and Theowald (1992) and CCW 2020 (which provide Morocco with a question mark for ﻿ obscuriventris). Thus far, the only positively identified ﻿ Emodotipula species from Morocco and the Rif region for which evidence in the form of specimens is available is T. leo.

The following species were considered as Moroccan on the basis of the species represented in the collections of the MISR (Rabat, Morocco): Aspistes berolinensis Meigen, 1818, Apiloscatopse ﻿ flavicollis (Meigen, 1818), ﻿ Reichertella pulicaria (Loew, 1846). However, none of these species is represented by specimens originating from Morocco. Accordingly, they are excluded from the present checklist.

﻿ Prosimulium ﻿ latimucro (Enderlein, 1925) is a species complex, but only one cytoform has been found in Morocco. It is called ﻿ Prosimulium ﻿ latimucro cytoform C Kúdela, Kúdelová & Adler, 2017 (Arrondo and de Blas Giral 2017: 16).

The original reports of ﻿ Prosimulium ﻿ rachiliense in Morocco appeared under the name P. rufipes. The chromosomes described by Adler and Belqat (2001), however, indicate that populations in Morocco’s Rif Mountains are not P. rufipes, but are a species closer to P. rachiliense (Belqat et al. 2018; Adler and Şirin 2014).

﻿ Metacnephia nuragica was listed with faunistic and bibliographical details in Belqat et al. (2011: 48) but its identity is uncertain.

The identity of ﻿ Thaumalea spinata is not finally clarified. No specimen outside the French Alps was found in Vaillant’s collection.

The species of this family are considered as Moroccan on the basis that they are represented by specimens in the collections of the MISR (Mouna 1998). However, it has not been possible to confirm if the specimens present in this collection actually originated from Morocco.

Three specimens identified as Rhyphus fenestralis are present in the collection of MISR (Rabat, Morocco). However, genital examination is required for identification of S. fenestralis and the closely related widespread S. cinctus (Fabricius, 1787). Both species could probably occur in Morocco.

The following species have also been considered as Moroccan on the basis of specimens present in the collections of the MISR: ﻿ Bibio clavipes Meigen, 1818, B. ferruginatus Linnaeus, 1767, B. fulvipes Zetterstedt, 1838, B. hybridus Haliday, 1833, B. lacteipennis Zetterstedt, 1850, B. nigriventris Haliday, 1833, B. pomonae (Fabricius, 1775), B. reticulatus Loew, 1846, B. siculus Loew, 1846, B. varipes Meigen, 1830, B. villosus Meigen, 1818, D. femoratus Meigen, 1804, D. humeralis Zetterstedt, 1850 (Mouna 1998). However, none of these species is represented in this collection by specimens originating from Morocco. Accordingly, they are excluded from the present checklist. An additional, still undescribed, species of ﻿ Bibio has been collected in the Rif Mountains (Ketama) in 2004 and 2012 by K. Kettani and will be described elsewhere.

The identity of the material of D. bispinosus mentioned in Séguy (1953a) should be checked, as D. clavicornus Skartveit & Kaplan, 1996, a closely related species that may be present in Morocco, has been described from Israel since this publication.

﻿ Pangonius sobradieli, said to be known from Morocco, was considered to be the same species as P. powelli by Chvála et al. (1972: 90).

﻿ Vermileo vermileo (Linnaeus, 1758) is a species complex currently undergoing revision (Kehlmaier pers. comm.); additionally, V. vermileo is possibly not a species that occurs in Morocco.

The record of ﻿ Usia ﻿ forcipata is doubtful as this species is probably endemic to Greece (Gibbs 2011).

﻿ Usia (﻿ Usia) ﻿ bicolor is an east Mediterranean species (Gibbs 2014) and the record from Morocco is most likely a misidentification.

﻿ Thereva ﻿ bipunctata has been considered a junior synonym of unica, but these are valid sister species because of differences in the aedeagus between the southern and northern populations (Ebejer pers. comm.).

﻿ Thereva ﻿ brevicornis may be a misidentification of authors. ﻿ Thereva ﻿ brevicornis is more central European in distribution, and is not found in Spain.

﻿ Thereva ﻿ funebris, ﻿ Thereva ﻿ graeca, and ﻿ Thereva ﻿ plebeja are cited only on the basis of the list of Diptera species present in the collections of the MISR published by Mouna (1998). The identifications of this material could not be verified.

﻿ Thereva ﻿ strigata may be a misidentification of authors.

The following species records from Morocco are known to the authors by various checklists with no material provided: ﻿ Argyra ﻿ grata Loew, 1857; ﻿ Sybistroma ﻿ obscurellum Fallén, 1823; ﻿ Tachytrechus ﻿ consobrinus (Haliday, 1851); ﻿ Medetera ﻿ petrophila Kowarz, 1877; ﻿ Rhaphium ﻿ appendiculatum Zetterstedt, 1849. Their presence in Morocco must be confirmed.

Hydrophorus ﻿ viridis may be a misidentification from Boumezzough and Vaillant (1986a) as this species is doubtfully present in North Africa. It does not reach Iberia in the west or Greece in the east.

Pollet and Stark (2018) have changed the species concept of ﻿ Orthoceratium lacustre (Scopoli, 1763). As a result, all previous records of the species from Atlantic countries and almost all records from Africa (except for a female record from Tunisia) have been assigned to O. sabulosum (Becker, 1907). The Moroccan material also belongs to the latter species (Martin Ebejer, pers. comm.).

Morocco was most probably erroneously included into the distribution section for ﻿ Medetera ﻿ flavipes Meigen, 1824 and ﻿ Medetera ﻿ pallipes (Zetterstedt, 1843) in some papers (e.g. Kazerani et al. 2014). M. ﻿ pallipes was recorded in the country by Nourti et al. (2019a). M. ﻿ flavipes is not included in the present Catalog.

Medetera ﻿ truncorum Meigen, 1824 was identified by a female only (Ebejer et al. 2019). However, ﻿ Medetera ﻿ truncorum is a group of closely related species present in the Mediterranean that can be identified only by fine structures of the male hypopygium (Negrobov and Naglis 2016). Its presence in Morocco must be confirmed.

Sciapus ﻿ longulus (Fallén, 1823) was identified by females only (Pârvu et al. 2006). Its presence in Morocco must be confirmed, as in most species in this genus from the Mediterranean they can only be distinguished by males (Grichanov and Negrobov 2014).

Campsicnemus ﻿ curvipes (Fallén, 1823) was recorded by Parent (1924) from Algeciras (Spain) and mentioned by Parent (1927) as collected from “Morocco: Algésiras”. ﻿ Argyra ﻿ grata Loew, 1857 was recorded by Parent (1927) from Algeria. These species may occur in Morocco.

Crossopalpus aenescens (Wiedemann, 1830) was recorded by Séguy (1941d: 12) under the name of ﻿ Drapetis aenescens, confusing it with C. aeneus. These two species have often been confused by authors. This is a misidentification, as C. aenescens is an Afrotropical species.

Stilpon demnatensis Vaillant, 1956 was indicated as a nomen ﻿ nudum by Cumming and Cooper (1992).

Becker and Stein’s record of ﻿ Tachydromia annulimana is retained, but is considered doubtful as ﻿ annulimana is a European species. The record could equally refer to T. umbrarum Haliday, 1833 another European species which has often been misidentified as ﻿ annulimana by authors.

Diplonevra tangeriana (Becker, 1913) was recorded from Morocco, but fresh specimens are required to ascertain its identity.

Microsania raclinae Collart is listed in Mouna 1998, but raclinae is a nomen nudum and the material upon which this citation was given is unknown to us. It cannot be determined whether or not the error is with the species name itself while the genus ﻿ Microsania is actually represented in Morocco.

The range of ﻿ Orthonevra ﻿ brevicornis does not extend to Iberia. The identification of the single specimen from Morocco was based on a female in Sahib et al. (2020). The status of this species in Morocco needs to be confirmed.

Eristalis ﻿ jugorum is not known closer to Morocco than the mountains of northern Spain and Turkey. Its occurrence in high mountains in Morocco is possible, but confirmation of the records would be worthwhile.

Eumerus ﻿ strigatus is not recorded from Morocco by Claußen and Hauser 1990, or by Claußen 1989b. However, it is listed for Morocco by Claußen 1989b, based on records from earlier authors (Kanervo 1939; Timon-David 1951). ﻿ Eumerus strigatus is now known to be a complex of at least four species (E. consimilis, E. narcissi, E. sogdianus, E. strigatus). Any of those four species might be present in Morocco, but E. strigatus is the least likely to be present, given its rarity in the Mediterranean zone of Europe.

Revision of the ﻿ Merodon constans species group (Vujić et al. 2020) has revealed the existence of cryptic taxa whose status is as yet uncertain. The name ﻿ constans can only be applied provisionally to the species recorded from Morocco, until these issues are resolved and the identity of Moroccan specimens can be meaningfully re-assessed.

Revision of ﻿ Merodon shows that M. aeneus (often also referred to as M. aureus) is confined to the Alps of southern Europe. Two very similar species, M. pumilus and M. unicolor, are known from N. Africa. Moroccan records of M. aeneus almost certainly refer to either M. pumilus or M. unicolor.

Merodon arrasus is probably a junior synonym of ﻿ Merodon eques Fabricius, 1805 (Ebejer and Bensusan 2010).

Revision of the ﻿ Merodon avidus complex by Popović et al. (2015) demonstrates the presence of M. ibericus in Morocco, but not M. avidus. Given the confusion between these species, earlier records of M. avidus from Morocco could refer either to ﻿ avidus or ﻿ ibericus.

Platycheirus albimanus is removed from the list for Morocco by Kassebeer 1998, who describes two new Moroccan species closely related to P. albimanus.

Eupeodes punctifer is almost confined to Northern Europe, north of the Arctic Circle, and a record from Morocco seems almost impossible. It is not possible to suggest on which species the Moroccan record might be based.

Although the faunistic knowledge is good in that there are many species recorded, this does not mean the identifications and taxonomy are all correct (Stuke, in litteris 26.v.2016). Given several recent revisions, especially within the genera ﻿ Myopa, ﻿ Physocephala, and ﻿ Thecophora, most of the Moroccan species listed here are in need of careful reconsideration.

﻿ Platystoma idia is possibly a synonym of P. meridionale Hendel.

﻿ Myopites apicatus is probably a synonym of M. ﻿ inulaedyssentericae Blot and at least the Moroccan record appears to be the latter species. A revision of ﻿ Myopites is in preparation (Korneyev, pers. comm.).

Records of ﻿ Urophora congrua are possibly based on misidentifications. Confirmation requires re-examination of the specimens.

﻿ Euleia marmorea may be a synonym of ﻿ Euleia heraclei.

Minettia flavipalpis and M. ﻿ plumicornis are not confirmed for Morocco; see Ebejer and Kettani 2019a.

﻿ Themira paludosa is a dubious record since T. paludosa seems reliably recorded only from NW Europe (Estonia, Finland, NW Russia).

Many species (﻿ Chlorops pumilionis, ﻿ Eutropha albipilosa, ﻿ Meromyza athletica, ﻿ Meromyza curvinervis, ﻿ Elachiptera cornuta, ﻿ Oscinisoma cognatum) are cited only on the basis of the list of Diptera species present in the collections of the MISR (Rabat, Morocco) published by Mouna (1998). The identification of this material could not be verified. However, the presence of most of these species is possible in Morocco.

﻿ Assuania melanoleuca comb. nov.: a female type specimen of ﻿ Chlorops melanoleucus is a species of ﻿ Assuania (Nartshuk, pers. comm.), see also Ebejer and Kettani (2016: 226). It may belong to A. thalhammeri with its three synonyms A. glabra Becker, 1903, A. frontata Becker, 1907, and A. siciliensis Duda, 1933 or to one of the further five ﻿ Assuania ﻿spp. (Sabrosky 1980: 707) present on the African continent. Dawah and Abdullah (2006: 30) and Deeming and Al-Dhafer (2012: 56, 88) reported an unidentifiable species from the Arabian Peninsula. Until a revision of this genus is made, A. melanoleuca must be treated as a valid taxon.

﻿ Epimadiza nigrescens: Nartshuk (1984: 236) erroneously sank Oscinosoma [sic! misspelling of Séguy] ﻿ anthracias Séguy, 1949 as a junior synonym of Siphonella oscinina (Fallén, 1820), although this synonymy had not been proposed previously. This error was clarified by Ebejer and Kettani (2016: 227) after correspondence with Nartshuk, but the puzzle increased as the latter authors introduced O. anthracias as a new junior synonym of ﻿ Polyodaspis ruficornis after personal correspondence of MJE with E.P. Nartshuk who had examined the type of O. anthracias in the year 2001. Both had overlooked the hidden synonymisation of ﻿ Oscinisoma anthracias with ﻿ Epimadiza nigrescens by Sabrosky (1965: 406), cited by Sabrosky (1980: 699) and by Dawah and Abdullah (2006: 26). Séguy did not mention in his description if he had only one male or more. Sabrosky may have studied a male other than the one studied by Nartshuk. The type(s) of O. anthracias should be examined again. If there is more than one specimen this might explain why two different senior taxa resulted. Here we follow the oldest examination of a type specimen by Sabrosky.

﻿ Polyodaspis ruficornis (Macquart), a widely distributed species, was reported by Ebejer and Kettani (2016) only on the basis of the literature, but as explained in footnote 38 it must be deleted from the list of Moroccan Diptera as the cited records are based on an earlier misidentification.

The record of ﻿ Delia flavogrisea needs to be verified. No details or genitalia figures are given. Although it is possible that this species could occur in Morocco, it is not recorded from Spain. Described from northern Sweden, it was recorded by G.C.D. Griffiths from Yukon, northern Canada (1993, Flies of the Nearctic Region Vol. VIII, Part 2, No 10: 1509), where excellent figures of the genitalia are given. It has also been recorded from northern China.

We do not have any knowledge of ﻿ Fannia cothurnata in Morocco. Identification is difficult, but it could be correct.

We do not have any knowledge of ﻿ Fannia sociella in Morocco. Its occurrence there seems doubtful.

﻿ Coenosia pumila requires verification. It is not known from Spain and is distributed in temperate areas of Europe. It may be found in the High Atlas Mts, but specimens from there require confirmation.

﻿ Morellia ﻿ simplex is almost certainly a misidentification of ﻿ Morellia ﻿ asetosa Baranov, 1925, which replaces it in the south and south-east of the West Palaearctic Region.

﻿ Phaonia ﻿ cincta requires verification. It is known from Spain and from the Middle East, and its occurrence in Morocco is possible.

According to Rognes (2002: 38) ﻿ Rhyncomya ﻿ callopis records may refer to similar species with spines on the surstylus.

The records of ﻿ Sarcophila latifrons (Fallén) require verification. The genus ﻿ Sarcophila is in need of revision and is likely much more diverse in the Mediterranean area than previously thought, so older records of S. latifrons from Morocco must be considered uncertain.

This record requires verification, as ﻿ Sarcophaga (﻿ Liosarcophaga tuberosa has often been confused with other species in the literature.

After checking photographs of the male terminalia of the specimen reported by Ebejer et al. (2019) as ﻿ Sarcophaga (﻿ Thyrsocnema incisilobata Pandellé, 1896, we consider that it most likely represents an undescribed species.

﻿ Estheria picta: the identity of this species is not confirmed; see note in Tschorsnig 2017.

The following species ﻿ Thelaira haematodes, ﻿ Carcelia iliaca, ﻿ Drino gilva, and ﻿ Drino imberbis, have been considered as Moroccan on the basis of specimens represented in the collection of MISR (Rabat, Morocco) or cited in some old Moroccan references: However, the identifications are not yet confirmed.

﻿ Gymnosoma rotundatum older identifications of G. rotundatum are not reliable. Possibly they were the true G. rotundatum, but it is more likely that other species of the genus ﻿ Gymnosoma were concerned.

﻿ Siphona geniculata: the identifications of this species in the cited references were made before the revision of the genus by Andersen (1996) and may be mistaken. Although this species could be present in Morocco, this needs to be confirmed.

## References

[B1] Abkari A Jouhadi Z Hamdani A Mikou N Guessous N Khalifa H (1998) La myiase gastro‑intestinale. A propos d’une observation marocaine. Manuscrit №1928. “Clinique”. 10214514

[B2] Abonnenc E (1972) Les phlébotomes de la région éthiopienne (Diptera: Psychodidae). Mémoire ORSTOM 55: 1–288.

[B3] Aboussaid H El Messoussi S Lhor Oufdou K (2009) Activité insecticide d’une souche marocaine de *Bacillusthuringiensis* sur la mouche méditerranéenne: *Ceratitiscapitata* (Wied.) (Diptera: Tephritidae). Afrique Science 5(1): 160–172. 10.4314/afsci.v5i1.61719

[B4] Ačanski J Vujić A Djan M Obreht Vidaković D Ståhls G Radenknović S (2016) Defining species boundarie in the *Merodonavidus* complex (Diptera: Syrphidae) using interative taxonomy, with the description of a new species. European Journal of Taxonomy 237: 1–25. 10.5852/ejt.2016.237

[B5] Ackland DM (1987) The genus *Anthomyia* in the Oriental Region (Diptera: Anthomyiidae). Insecta matsumurana 36: 39–60.

[B6] Ackland DM (2001) Revision of Afrotropical *Anthomyia* Meigen, 1803 (Diptera: Anthomyiidae), with descriptions of ten new species. African Invertebrates 42: 1–94.

[B7] Ackland DM (2008) Revision of Afrotropical *Delia* Robineau-Desvoidy, 1830 (Diptera: Anthomyiidae), with descriptions of six new species. African Invertebrates 49(1): 1–75. 10.5733/afin.049.0101

[B8] Ackland DM Pont AC (1966) *Lispocephalaungulata* (Rondani, 1866) (Diptera: Muscidae), a species distinct from *L.alma* (Meigen, 1826). Stuttgarter Beiträge zur Naturkunde 161: 1–5.

[B9] Adghir A de Jong H Kettani K (2018) The Tipulidae (Diptera) of northern Morocco with a focus on the Rif region, including the description of a new species of Tipula (Lunatipula) and an updated checklist for Morocco. Annales de la Société entomologique de France (N.S.) 54(6): 522–538. 10.1080/00379271.2018.1530949

[B10] Adlaoui E Faraj C El Bouhmi M El Aboudi A Ouahabi S Tran A Fontenille D El Aouad R (2011) Mapping Malaria Transmission Risk in Northern Morocco Using Entomological and Environmental Data. Malaria Research and Treatment 2011: e391463. 10.4061/2011/391463 PMC3265283 22312566

[B11] Adler P (2019) World blackflies (Diptera: Simuliidae): A comprehensive revision of the taxonomic and geographical inventory [2019]. Zootaxa 4455(1): 1–144. http://biomia.sites.clemson.edu/pdfs/blackflyinventory.pdf [accessed in 2019]

[B12] Adler PH Belqat B (2001) Cytotaxonomy of the *Prosimuliumhirtipes* species group (Diptera: Simuliidae) in Morocco. Journal of Insect Systematics and Evolution 32: 411–418. 10.1163/187631201X00281

[B13] Adler PH Şirin Ü (2014) Cytotaxonomy of the *Prosimulium* (Diptera: Simuliidae) of Western Asia. Zoological Journal of the Linnean Society 171: 753–768. 10.1111/zoj.12150

[B14] Adler PH Cherairia M Arigue SF Samraoui B Belqat B (2015) Cryptic biodiversity in the cytogenome of bird-biting blackflies in North Africa. Medical and Veterinary Entomology 29(3): 276–289. 10.1111/mve.12115 25801314

[B15] Adler PH Crosskey RW (2017) World blackflies (Diptera: Simuliidae): A comprehensive revision of the taxonomic and geographical inventory [2017]. https://biomia.sites.clemson.edu/pdfs/blackflyinventory.pdf [accessed September 2017]

[B16] AEFCS [Administration des Eaux et Forêts et de la Conservation des Sols] (1996) Plan d’aménagement et de gestion du Parc National du Toubkal. 226 pp.

[B17] Aellen V (1952) Contribution à la connaissance des diptères pupipares du Maroc. Bulletin de la Société scientifique naturelle et physique du Maroc 31: 149–152.

[B18] Aellen V (1955) Etude d’une collection de Nycteribiidae et de Streblidae de la région paléarctique occidentale, particulierement de la Suisse. Bulletin de la Société neuchâteloise des sciences naturelles 78: 81–104.

[B19] Aellen V (1963) Les Nycteribiidés de la Suisse, diptères parasites de chauves-souris. Bulletin de la Société neuchâteloise des sciences naturelles 86: 143–154.

[B20] Afonso JM Hernandez M Padron G Gonzalez AM (1985) Gametic non‑random associations in North-West African populations of *Drosophilamelanogaster*. Genetica 67: 3–11. 10.1007/BF02424455

[B21] Afonso JM Volz A Hernandez M Ruttkay H Gonzalez M Larruga JM Cabrera VM Sperlich D (1990) Mitochondrial DNA variation and genetic structure in Old-World populations of *Drosophilasubobscura*. Molecular Biology and Evolution 7: 123–142. 1969605 10.1093/oxfordjournals.molbev.a040590

[B22] Afzan H Belqat B (2016) Faunistic and bibliographical inventory of the Psychodinae moth-flies of North Africa (Diptera, Psychodidae). ZooKeys 558: 119–145. 10.3897/zookeys.558.6593 PMC4768284 27006599

[B23] Alaoui Slimani N (2002) Faune culicidienne d’une zone marécageuse de Rabat-Salé: Biotypologie et contribution à la lutte par des substances naturelles. Thèse de Doctorat d’Etat, Université Mohammed V, Rabat, 192 pp.

[B24] Alaoui Slimani N Jouid N Benhoussa A Hajji K (1999) Typologie des habitats d’*Anopheles* dans une zone urbaine (DipteraCulicidae). L’entomologiste 55(5): 181–190.

[B25] Alberola TM de Frutos R (1993) Gypsy Homologous Sequences in *Drosophilasubobscura* (gypsyDS). Journal of molecular Evolution 36: 127–135. 10.1007/BF00166248 8381880

[B26] Alberola TM Frutos R (1996) Molecular structure of a gypsy element of *Drosophilasubobscura* (gypsyDs) constituting a degenerate form of insect retroviruses. Nucleic Acids Research 24(5): 914–923. 10.1093/nar/24.5.914 8600460 PMC145713

[B27] Alluaud C (1923) Sur la présence d’un diptère de la Famille des Blépharoceridés. Bulletin de la Société scientifique naturelle du Maroc Tome III: 23–28.

[B28] Allemand R David JR (1976) The circadian rhythm of oviposition in *Drosophilamelanogaster*: A genetic latitudinal cline in wild populations. Experientia 32: 1403–1405. 10.1007/BF01937401

[B29] Amraoui F (2012) Le moustique *Culexpipiens*, vecteur potentiel des virus West Nile et fièvre de la vallée du Rift dans la région du Maghreb. Thèse de Doctorat National, Université Mohammed V, Rabat, Maroc, 105 pp.

[B30] Amraoui F Krida G Bouattour A Rhim A Daaboub J Harrat Z Boubidi SC Tijane M Sarih M Failloux AB (2012) *Culexpipiens*, an Experimental Efficient Vector of West Nile and Rift Valley Fever Viruses in the Maghreb Region. PLoS ONE 7(5): e36757. 10.1371/journal.pone.0036757 PMC3365064 22693557

[B31] Amraoui F Tijane M Sarih M Failloux AB (2012) Molecular evidence of Culexpipiensformmolestus and hybrids *pipiens*/*molestus* in Morocco, North Africa. Parasites & Vectors 5(1): 83–86. 10.1186/1756-3305-5-83 22541050 PMC3409039

[B32] Amraoui F Ben Ayed W Madec Y Faraj C Himmi O Ameur B Sarih M Failloux B (2019) Potential of *Aedesalbopictus* to cause the emergence of arboviruses in Morocco. PLOS Neglected Tropical Diseases 13(2): e0006997. 10.1371/journal.pntd.0006997 PMC6392334 30763312

[B33] Amri A El Bouhssini M Jlibene M Cox TS Hatchett JH (1992) Evaluation of *Aegilops* and *Triticum* species for resistance to the Moroccan Hessian fly (Diptera: Cecidomyiidae). Al Awamia 77: 109–118.

[B34] Andersen S (1996) The *Siphonini* (Diptera: Tachinidae) of Europe. Fauna entomologica Scandinavica 33: 146 pp.

[B35] Andrade R Almeida J (2010) New records of the family Chyromyidae (Diptera: Brachycera) for mainland Portugal. Boletín de la Sociedad Entomológica Aragonesa 46: 146.

[B36] Andreassen L (2007) Post diapause development of *Deliaradicum* (Diptera: Anthomyiidae) and host range of *Aleocharabipustulata* (Coleoptera: Staphylinidae) for classical biological control in Canadian Canola. A Thesis/Practicum submitted to the Faculty of Graduate Studies of the University of Manitoba in partial fulfïllment of the requirement of the degree Master of Science. 171 pp.

[B37] Andréu Rubio JN (1959) Bombilidos marroquies del Instituto español de Entomología (Díptera). EOS, Revista Española de Entomologia 35(1): 7–19.

[B38] d’Anfreville L (1916) Les Moustiques de Salé (Maroc). Bulletin de la Société de pathologie exotique 9: 104–142.

[B39] Aouinty B Oufaras S Mellouki F Mahari S (2006) Évaluation préliminaire de l’activité larvicide des extraits aqueux des feuilles du ricin (*Ricinuscommunis* L.) et du bois de thuya (*Tetraclinisarticulata* (Vahl) Mast.) sur les larves de quatre moustiques culicidés: *Culexpipiens* (Linné), *Aedescaspius* (Pallas), *Culisetalongiareolata* (Aitken) et *Anophelesmaculipennis* (Meigen). Biotechnologie, Agronomie, Société et Environnement 10(2): 67–71.

[B40] Arahou M (2008) Catalogue de l’entomofaune du Chêne vert du Moyen Atlas (Maroc). Documents de l’Institut scientifique Rabat 22: 1–36.

[B41] Arias J (1913) Sobre los nemestrínidos de Marruecos. Boletín de la Real Sociedad Española de Historia Natural 13: 150–154.

[B42] Arias J (1914) Dipteros de España. Fam. Mydaidae. Con descripcion de algunas especies del Norte de Africa. Trabajos del Museo de Ciencias Naturales de Madrid, Serie zoológica XV: 1–40. [10 fig, 6 pl.]

[B43] Arrondo IR de Blas Giral I (2017) Book of abstracts. VII International Simuliidae Symposium, 5–8 September 2016. Zaragoza, Spain. Simuliid Bulletin 47 (Supplement): 1–43.

[B44] Ashburner M Lemeunier F (1976) Relationships within the *melanogaster* species subgroup of the genus Drosophila (Sophophora). I. Inversion polymorphisms in *Drosophilamelanogaster* and *Drosophilasimulans*. Proceedings of the Royal Society of London Series B 193: 137–157. 10.1098/rspb.1976.0036 5729

[B45] Ashe P Cranston PS (1990) Family Chironomidae. In: Soós Á, Papp L (Eds) Akadémiai Kiadó, Budapest, 113–355. [499 pp]

[B46] Ashe P O’Connor JP (2009) A World Catalogue of Chironomidae (Diptera). Part 1. Buchonomyiinae, Chilenomyiinae, Podonominae, Aphroteniinae, Tanypodinae, Usambaromyiinae, Diamesinae, Prodiamesinae and Telmatogetoninae. Irish Biogeographical Society & National Museum of Ireland, Dublin, 445 pp.

[B47] Ashe P O’Connor JP (2012) A World Catalogue of Chironomidae (Diptera). Part 2. Orthocladiinae (Section A & Section B). Irish Biogeographical Society & National Museum of Ireland, Dublin, 968 pp.

[B48] Ashe P O’Connor JP Murray DA (2015) A review of the distribution and ecology of *Buchonomyiathienemanni* Fittkau (Diptera: Chironomidae) including a first record for Russia. European Journal of environmental Sciences 5(1): 5–11. 10.14712/23361964.2015.69

[B49] Ayala FJ Serra L Prevosti A (1989) A grand experiment in evolution: the *Drosophilasubobscura* colonization of the Americas. Genome 31: 246–255. 10.1139/g89-042

[B50] Ayoub M (2002) Study on the introduction of organic tomato greenhouse in Massa plain, Agadir, South Morocco. DSPU thesis in Mediterranean Organic Agriculture, Organic Agriculture, Mediterranean agronomic institute of Bari, 30 pp.

[B51] Ayrinhac A Debat V Gibert P Kister AG Legout H Moreteau B Vergilino R David JR (2004) Cold adaptation in geographical populations of *Drosophilamelanogaster*: phenotypic plasticity is more important than genetic variability. Functional Ecology 18: 700–706. 10.1111/j.0269-8463.2004.00904.x

[B52] Azzam A Azzam S Lhaloui S Amri A El Bouhssini M Moussaoui M (1997) Economic Returns to Research in Hessian fly (Diptera: Cecidomyiidae). Resistant Bread-Wheat Varieties in Morocco. Journal of economic Entomology 90(1): 1–5. 10.1093/jee/90.1.1

[B53] Azzouzi A Laville H (1987) Premier inventaire faunistique des Chironomidés (Diptera, Chironomidae) du Maroc. Annals de Limnologie 23(3): 217–224. 10.1051/limn/1987020

[B54] Azzouzi A Laville H Reiss F (1992) Nouvelles récoltes de Chironomidés (Diptera) du Maroc. Annals de Limnologie 28(3): 225–232. 10.1051/limn/1992019

[B55] Baba-Aissa F Solignac M Dennebouy N David JR (1988) Mitochondrial DNA variability in *Drosophilasimulans*: quasi absence of polymorphism within each of the three cytoplasmic races. Heredity 61: 419–426. 10.1038/hdy.1988.133 2906636

[B56] Bächli G (1982) On the type material of Palaearctic species of Drosophilidae (Diptera). Beiträge zur Entomologie 32: 289–301.

[B57] Bächli G (2015) TaxoDros (The database on Taxonomy of Drosophilidae) compiled by Gerhard Bächli. http://www.TaxoDros.uzh.ch/ [accessed March 2015]

[B58] Bächli G (2020) TaxoDros (The database on Taxonomy of Drosophilidae) compiled by Gerhard Bächli. http://www.TaxoDros.uzh.ch/ [accessed March 2019]

[B59] Bächli G Rocha Pité MT (1984) Family Drosophilidae. In: Soós Á Papp L (Eds) Catalogue of Palaearctic Diptera.Vol. 10: Clusiidae – Chloropidae. Akadémiai Kiadó, Budapest, 186–220. [402 pp]

[B60] Bader K Arabyat S (2004) The beeflies (Diptera: Bombyliidae) of Jordan. Denisia 14, zugleich Kataloge der OÖ. Landesmuseum, neue Serie 2: 353–384.

[B61] Badrawy H Mohammad SK (2013) A revision of the Egyptian species of the genus *Actorthia* Kröber (Diptera: Therevidae: Phycinae). Zootaxa 3613(2): 181–189. 10.11646/zootaxa.3613.2.6 24698910

[B62] Báez M (1978) Los Hipoboscidos de las Islas Canarias (Diptera, Hippoboscidae). Boletín de la Estación central de Ecología 13: 59–72.

[B63] Bahid FZ (2018) Les Empidinae (Insecta, Diptera, Empidoidea) du Maroc: Taxonomie, systématique, atlas de répartition et écologie. Thèse Es-Sciences, Université Abdelmalek Essaadi, Tétouan, Maroc, 274 pp.

[B64] Bahid FZ Kettani K Daugeron C (2017) Two new species of the Empis (Leptempis) rustica group of the Mediterranean basin (Diptera: Empididae: Empidinae). Annales de la Société entomologique de France (N.S.). International Journal of Entomology 53(6): 422–427. 10.1080/00379271.2017.1399085

[B65] Bahid FZ Kettani K Daugeron C (2018) A new species of Empis (Xanthempis) from Morocco (Diptera, Empididae, Empidinae). Bulletin de la Société entomologique de France 123(1): 119–123. 10.32475/bsef_2026

[B66] Bailly-Choumara H (1965a) Présence de Mansonia (Coquillettidia) richiardii (Ficalbi, 1896) et Mansonia (Coquillettidia) buxtoni (Edwards) 1923 au Maroc. Première récolte d’espèce du genre *Mansonia* Blanchard, 1901 en Afrique du Nord (*Diptera*, *Culicidae*). Bulletin de la Société de pathologie exotique 58: 676–679. 4381082

[B67] Bailly-Choumara H (1965b) Présence au Maroc d’*Orthopodomyiapulchripalpis* Rondani, 1872 (Diptera, Culicidae). Bulletin de la Société des sciences naturelles et physiques du Maroc 1e et 2e trimestres, 45: 39–41.

[B68] Bailly-Choumara H (1965c) Rapport d’une mission entomologique effectuée dans la province d’Agadir, du 19.04.65 au 09.05.65. Laboratoire d’Entomologie, Institut Scientifiqie Chérifien, Rabat, 11 pp.

[B69] Bailly-Choumara H (1966) Rapport d’une mission entomologique effectuée dans les provinces d’Agadir et de Tarfaya du 30.05.66 au 09.06.66. Laboratoire d’Entomologie, Institut Scientifique Chérifien, Rabat, 7 pp.

[B70] Bailly-Choumara H (1967a) Récapitulation des récoltes d’entomologie médicale dans le Moyen Atlas de 1964 à 1966, Laboratoire d’Entomologie, Institut Scientifique Chérifien, Maroc, 9 pp.

[B71] Bailly-Choumara H (1967b) Rapport d’une mission entomologique effectuée dans la vallée de la Moulouya du 1.10.67 au 10.10.67. Laboratoire d’Entomologie, Institut scientifique Chérifien, Rabat, 11 pp.

[B72] Bailly-Choumara H (1967c) Récapitulation des récoltes d’entomologie médicale effectuées dans le Rif de 1964 à 1966. Laboratoire d’Entomologie, Institut Scientifique Chérifien, Maroc, 8 pp.

[B73] Bailly-Choumara H (1968a) Contribution à l’étude des moustiques du Maroc (Diptera, Culicidae). Six espèces nouvelles pour le pays. Cahiers ORSTOM, Série Entomologie médicale 6(2): 139–144.

[B74] Bailly-Choumara H (1968b) Etude du rendement *Anopheleslabranchiae* des pièges C.D.C. fonctionnant sur batteries de 4, 5 volts ou 6 volts avec ou sans ampoule réalisée à Larache-Sheishat, Maroc, Rapport №70/36, Laboratoire d’Entomologie, Institut Scientifique Chérifien, Rabat, 5 pp.

[B75] Bailly-Choumara H (1970) Comparaison entre différentes méthodes de récolte de Moustiques adultes au Maroc. Rapport final du contrat de recherches O.M.S. M2/181/91, №58/70, Laboratoire d’Entomologie, Institut Scientifique Chérifien, Maroc, 42 pp.

[B76] Bailly-Choumara H (1972a) Etude détaillée d’une récolte d’*Anopheleslabranchiae* par pièges lumineux portatifs C.D.C. (4,5 volts), avec examen de la faune résiduelle, réalisée à Larache, Maroc. Rapport №10/72, Laboratoire d’Entomologie, Institut Scientifique Chérifien, Rabat, 18 pp.

[B77] Bailly-Choumara H (1972b) Evaluation de la capture manuelle à l’aspirateur par des récoltes consécutives au pyrèthre, faite pour *Anopheleslabranchiae* à Larache-Sheishat, Maroc (le 31 mai 1968). Rapport №27/72, Laboratoire d’Entomologie, Institut Scientifique Chérifien, Rabat, 4 pp.

[B78] Bailly-Choumara H (1973a) Etude préliminaire d’une récolte d’*Anopheleslabranchiae* par piège CDC réalisée dans la région de Larache, Maroc. Bulletin de l’Organisation Mondiale de la Santé 49: 49–55. PMC2481079 4545156

[B79] Bailly-Choumara H (1973b) Etude comparative de différentes techniques de récolte de Moustiques adultes (Diptera, Culicidae) faite au Maroc, en zone rurale. Bulletin de la Société des sciences naturelles et physiques du Maroc 1^er^ et 2^ème^ trimestre 53: 135–188.

[B80] Bailly-Choumara H (1977) Bibliographie entomologique marocaine. Retrospective de 1870 à 1975 inclus. Documents de l’Institut Scientifique 1, Rabat, 216 pp.

[B81] Bailly-Choumara H Kremer M (1970) Deuxième contribution à l’étude des *Culicoides* du Maroc (Diptera, Ceratopogonidae). Cahiers ORSTOM, Série Entomologie médicale et Parasitologie 8(4): 383–391.

[B82] Bailly-Choumara H Abonnenc E Pastre J (1971) Contribution à l’étude des Phlébotomes du Maroc (Diptera, Psychodidae). Données faunistiques et écologiques. Cahiers ORSTOM, Série Entomologie médicale et Parasitologie 9(4): 431–460.

[B83] Bailly-Choumara H Leger N (1976) Présence de *Sergentomyiaschwetzi* Adler, Théodor et Parrot (Diptera, Phlebotomidae) dans le Sud marocain. Cahiers ORSTOM., Série Entomologie médicale et Parasitologie vol. XIV, no 1: 13–14.

[B84] Bailly-Choumara H Beaucournu-Saguez F (1978) Contribution à l’étude des simulies du Maroc (Diptera, Simuliidae). 1. Le Rif. Bulletin de l’Institut scientifique, Rabat 3: 121–144.

[B85] Bailly-Choumara H Beaucournu-Saguez F (1981) Contribution à l’étude des simulies (Diptera, Simuliidae) du Maroc. 2. Le Haut-Atlas. Bulletin de l’Institut scientifique, Rabat 5: 39–57.

[B86] Balachowsky A Mesnil L (1935) Les insectes nuisibles aux plantes cultivées. Tome 1: 1058–1061. Paris.

[B87] Balenghien T Pagès N Goffredo M Carpenter S Augot D Jacquier E Talaver S Monaco F Depaquit J Grillet C Pujols J Satta G Kasbari M Setier-Rio ML Izzo F Alkan C Delécolle JC Quaglia M Charrel R Polci A Bréard E Federici V Cêtre-Sossah C Garros C (2014) The emergence of Schmallenberg virus across *Culicoides* communities and ecosystems in Europe. Preventive Veterinary Medicine 116(4): 360–369. 10.1016/j.prevetmed.2014.03.007 24698329

[B88] Banamar O Chandler PJ Driauach O Belqat B (2020) New faunistic records of the family Mycetophilidae (Insecta, Diptera) from Morocco. ZooKeys 934: 93–110. 10.3897/zookeys.934.49157 32508496 PMC7250956

[B89] Barták M Gregor F Rozkošný R (2004) New records of interesting Palaearctic Muscidae. In: Kubík Š Barták M (Eds) Dipterologica bohemoslovaca Vol. 11. Folia Facultatis Scientiarum Naturalium.Universitatis Masarykianae Brunensis, Biologia 109: 7–16.

[B90] Barták M Kubik Š (2005) Diptera of Podyjí National Park and its Environs. Česka Zemedelska Univerzita V Praze Fakulta Agrobiologie, Portvinovych a Přirodnich Zdrju, 432 pp.

[B91] Baylis M El Hasnaoui H Bouayoune H Touti J Mellor PS (1997) The spatial and seasonal distribution of African horse sickness and its potential *Culicoides* vectors in Morocco. Medical and Veterinary Entomology 11(3): 203–212. 10.1111/j.1365-2915.1997.tb00397.x 9330250

[B92] Bazyar Z Dousti AF von Tschirnhaus M Fallahzadeh M (2015) A first overview of the fauna of Chloropidae of Iran (Diptera, Acalyptratae). Turkish Journal of Zoology 39(6): 1041–1049. 10.3906/zoo-1405-3

[B93] Beaucournu-Saguez F Bailly-Choumara H (1981) Prosimulium (Prosimulium) laamii n. sp. (Nematocera, Simuliidae), simulie nouvelle du nord du Maroc. Cahiers ORSTOM, Série Entomologie médicale et Parasitologie 19: 113–119.

[B94] Beaucournu JC Beaucournu-Saguez F Guiguen C (1985) Nouvelles données sur les diptères pupipares (Hippoboscidae et Streblidae) de la sous-région méditerranéenne occidentale. Annales de Parasitologie Humaine et Comparée 60: 311–327. 10.1051/parasite/1985603311

[B95] Bechev D Koç H (2006) Two new species of *Sciophila* Meigen (Diptera: Mycetophilidae) from Turkey, with a key to the Western Palaearctic species of the *S.lutea* Macquart group. Zootaxa 1253: 61–68.

[B96] Becker T (1902) Aegyptische Dipteren. Mitteilungen aus dem zoologischen Museum in Berlin 2(2): 1–66. 10.1002/mmnz.4830020237

[B97] Becker T (1903) Die paläarktischen Formen der Gattung *Mulio* Latreille (Dipt.). Zeitschrift für systematische Hymenopterologie und Dipterologie 3: 89–96.

[B98] Becker T (1906a) *Usia* Latreille. Berliner entomologische Zeitschrift 50 [1905]: 193–228.

[B99] Becker T (1906b) Die Ergebnisse meiner dipterologischen Frühjahrsreise nach Algier und Tunis 1906 [part]. Zeitschrift für systematische Hymenopterologie und Dipterologie 6: 145–158. 10.5962/bhl.title.9280

[B100] Becker T (1907) Die Ergebnisse meiner dipterologischen Frühjahrsreise nach Algier und Tunis. 1906. Zeitschrift für systematische Hymenopterologie und Dipterologie 7(5): 369–407. 10.5962/bhl.title.9280

[B101] Becker T (1910) Chloropidae. Eine monographische Studie. I. Teil. Paläarktische Region. Archivum zoologicum 1(10): 33–174, Tab. I–III. [Budapest] 10.5962/bhl.title.9555

[B102] Becker T (1912) Beitrag zur Kenntnis der Thereviden. Verhandlungen der kaiserlich-königlichen zoologisch-botanischen Gesellschaft in Wien 62: 289–319.

[B103] Becker T (1917) Dipterologische Studien. Dolichopodidae A. Paläarktische Region // Nova acta Academiae Caesareae Leopoldino-Carolinae Germanicae Naturae Curiosorum Vol. 102(2): 113–361.

[B104] Becker T (1921) Neue Dipteren meiner Sammlung. Mitteilungen aus dem zoologischen Museum in Berlin 10: 1–93. 10.1002/mmnz.4830100102

[B105] Becker T Stein P (1912) Dipteren aus Marokko. Bulletin de l’Académie Impériale des Sciences de St.-Petersbourg VI Série 6(9): 602–603.

[B106] Becker T Stein P (1913) Dipteren aus Marokko. Annuaire du Musée zoologique de l’Académie impériale de Sciences de St.-Petersbourg 18: 62–95.

[B107] Bei-Bienko GYa Steyskal GC (1989) Keys to the Insects of the European Part of the USSR. Vol. V Diptera and Siphonaptera, part 1, 1233 pp/ and part 2, xxii + 1505 pp. [E.J. Brill, Leiden]

[B108] Belakoul N (1985) Contribution à l’étude de la faune culicidienne dendrolimnique de la Subéraie en pays Zaèr (Maroc). Colonisation par *Culexpipiens* d’un nouvel habitat: causalité et étude comparée de deux formes de *Culexpipiens* (daya, creux). Mémoire de DEA, Université Paul Sabatier, Toulouse, 113 pp.

[B109] Belqat B (2000) Découverte de Simulium (Obuchovia) galloprovinciale et Simulium (Obuchovia) auricoma: deux nouvelles espèces pour le Nord de l’Afrique. British Simuliid Group Bulletin 15: 15–18.

[B110] Belqat B (2002) Etude Systématique, Ecologique et Caryologique des Simulies (Diptera: Simuliidae) du Maroc: Cas Particulier du Rif. Thèse d’Etat Es Sciences. Université Abdelmalek Essaâdi, Faculté des Sciences, Tétouan, 322 pp.

[B111] Belqat B Adler PH (2001) Ecologie et biogéographie du genre *Prosimulium* Roubaud (Diptera, Simuliidae) dans le Rif (Nord du Maroc). Zoologica baetica 12: 119–134.

[B112] Belqat B Dakki M (2004) Clés analytiques des Simulies (Diptera) du Maroc. Zoologica baetica 15: 77–137.

[B113] Belqat B Adler PH Dakki M (2001a) Distribution summary of the Simuliidae of Morocco with new data for the Rif mountains. British Simuliid Group Bulletin 17: 10–16.

[B114] Belqat B Dakki M Errami M (2001b) Deux nouvelles simulies pour le Nord de l’Afrique: Simulium (Nevermannia) angustitarse et Simulium (Simulium) trifasciatum. British Simuliid Group Bulletin 17: 7–10.

[B115] Belqat B Dakki M El Alami M (2005) Estructura biotipológica de las principales redes hídricas Rifeñas a través de los Simúlidos (Diptera: Simuliidae). Ecosistemas 14: 50–56.

[B116] Belqat B Bennas N El Alami M Kettani K Aoulad Ali S (2008) Faune Simulidienne (Diptera: Simuliidae) du bassin versant de l’Oued Laou (Maroc). In: Bayed A Ater M (Eds) Du bassin versant vers la mer: Analyse multidisciplinaire pour une gestion durable- Cas du bassin méditerranéen de Oued Laou. Travaux de l’Institut scientifique, Rabat.Série générale 5: 61–74.

[B117] Belqat B Adler PH Crosskey RW (2011) Faunistic and bibliographical inventory of the blackflies (Diptera: Simuliidae) of Morocco. Zootaxa 2829: 46–58. 10.11646/zootaxa.2829.1.2

[B118] Belqat B Adler PH Cherairia M Chaoui Boudghane-Bendiouis C (2018) Inventory of the Black Flies (Diptera: Simuliidae) of North Africa. Zootaxa 4442(2): 201–220. 10.11646/zootaxa.4442.2.1 30313958

[B119] Benabdelkrim Filali O Kabine M El Hamouchi A Lemrani M Debboun M Sarih M (2018) First Molecular Identification and Phylogeny of Moroccan *Anophelessergentii* (Diptera: Culicidae) Based on Second Internal Transcribed Spencer (ITS2) and Cytochrome c Oxidase I (COI) Sequences. Vector borne and zoonotic Diseases 18(9): 479–484. 10.1089/vbz.2018.2269 29870316

[B120] Benhoussa A Aguesse P Dakki M (1993) Microdistribution larvaire de trois populations de simulies (Insecta, Diptera) de l’oued Bou Regreg (Maroc). Vie et Milieu 43: 247–253.

[B121] Benhoussa A El Agbani MA Qninba A (1988) Dynamique et cycle biologique de quelques populations simulidiennes (Diptera–Simuliidae) du Bou Regreg (Plateau Central marocain). Bulletin de l’Insitut Scientifique, Rabat 12: 157–165.

[B122] Benmansour N Aadel A Mouki B (1972) Étude de la sensibilité au DDT de l’*Anophelesmaculipennislabranchiae* au Maroc de 1959 à 1971. Annales médico-chirurgicales d’Avicenne 213–219.

[B123] Benmansour N Laaziri M Mouki B (1972) Note sur la faune anophilienne du Maroc. Bulletin de l’Institut d’Hygiène (Nouvelle Série), Rabat 52: 103–112.

[B124] Bennouna A Balenghien T El Rhaffouli H (2016) First record of *Aedesalbopictus* (Diptera: Culicidae) in Morocco: a major threat to public health in northern Africa? Medical and Veterinary Entomology 31(1): 102–106. 10.1111/mve.12194 27775162

[B125] Bequaert J (1939) Notes on Hippoboscidae. 13. A Second Revision of the Hippoboscinæ. Psyche 46: 70–90. 10.1155/1939/48398

[B126] Bernard MR Grenier P Bailly-Choumara H (1972) Description de Prosimulium (Prosimulium) faurei n.sp. (Diptera: Simuliidae). Cahiers ORSTOM, Série Entomologie médicale et Parasitologie 10: 63–68.

[B127] Bernardi N (1973) The genera of the family Nemestrinidae (Diptera: Brachycera). Arquivos de Zoologia 24(4): 211–318. 10.11606/issn.2176-7793.v24i4p211-318

[B128] Berrahou A Cellot B Richoux P (2001) Distribution longitudinale des macroinvertébrés benthiques de la Moulouya et de ses principaux affluents (Maroc). Annales de Limnologie 37(3): 223–235. 10.1051/limn/2001020

[B129] Bezzi M (1906) Ditteri Eritrei raccolti dal Dott. Andreini e dal Prof. Tellini. Parte prima. DipteraOrthorrhapha. Bulletino della Società entomologica italiana 37: 195–304.

[B130] Biémont C Nardon C Deceliere G Lepetit D Loevenbruck C Vieira C (2003) Worldwide distribution of transposable element copy number in natural populations of *Drosophilasimulans*. Evolution 57: 159–167. 10.1111/j.0014-3820.2003.tb00225.x 12643577

[B131] Bigot JMF (1884) Diptères nouveaux ou peu connus. 24e partie. XXXII. Syrphidi (2e partie). Espèces nouvelles, III. Annales de la Société entomologique de France 6(4): 73–116.

[B132] Biliotti É (1956) Biologie de *Phryxecaudata* Rond., parasite de la chenille processionnaire du pin (*Thaumetopoeapityocampa* Schiff.). Revue de Pathologie végétale et d’Entomologie agricole de France 35: 50–65.

[B133] Biron DG Landry BS Nénon JP Coderre D Boivin G (2000) Geographical origin of an introduced pest species, *Deliaradicum* (Diptera: Anthomyiidae), determined by RAPD analysis and egg micromorphology. Bulletin of entomological Research 90: 23–32. 10.1017/S0007485300000675 10948360

[B134] Bkhache M Tmimi FZ Charafeddine O Filali OB Lemrani M Labbé P Sarih M (2018) G119S ace-1 mutation conferring insecticide resistance detected in the *Culexpipiens* complex in Morocco. Pest Management Science 75(1): 286–291. 10.1002/ps.5114 29885052

[B135] Bléton C-A (1938) Observations sur la biologie d’*Hylemyiasepia* Meigen (Diptera, Muscidae), parasite du blé au Maroc. Bulletin de la Société scientifique naturelle du Maroc 18: 3–5.

[B136] Bléton C-A Fieuzet L (1939) Notes sur quelques insectes auxiliaires observés dans la région de Fès. Bulletin de la Société scientifique naturelle du Maroc 19(2): 57–65.

[B137] Bléton C-A Fieuzet L (1943) Sur la présence et la biologie au Maroc, d’*Atherigonasoccata* Rondani, Diptère parasite du Sorgho cultivé. Bulletin de la Société d’histoire naturelle d’Afrique du Nord 34(4): 112–117.

[B138] Bonjean M (1947) L’épidémiologie du paludisme au Maroc. Bulletin de l’Institut d’Hygiène du Maroc 7, 119 pp.

[B139] Bonnivard E Higuet D (1999) Stability of European natural populations of *Drosophilamelanogaster* with regard to the P-M system: a buffer zone made up of Q populations. Journal of evolutionary Biology 12: 633–647. 10.1046/j.1420-9101.1999.00063.x

[B140] Borkent A (2012) World Species of Biting Midges (Diptera: Ceratopogonidae). [Catalog on-line] www.inhs.uiuc.edu/research/FLYTREE/CeratopogonidaeCatalog.pdf

[B141] Borkent A Wirth WW (1997) World species of biting midges (Diptera: Ceratopogonidae). Bulletin of the American Museum of Natural History №233, 264 pp.

[B142] Bouallam S Ramdani M (1992) Culicidae de la région de Marrakech, principalement les Anophèles, vecteurs potentiels du Paludisme: répartition, taux d’infestation et lutte anti-larvaire. Actes de la IV^ème^ CILEF. Hydroécologie appliquée, publication particulière 1: 111–139.

[B143] Bouallam S Badri A Maaroufi A Bouzidi A (1997a) Typologie d’habitats d’*Anopheles* dans une zone urbaine (Diptera: Culicidae). L’entomologiste 55(5): 181–190.

[B144] Bouallam S Badri A Maaroufi A Bouzidi A (1997b) *Gambusiaaffinis* (Poecillidae des Khettaras) comme outil de lutte biologique contre les anophèles vecteurs potentiels du paludisme au Maroc. Mémoires de Biospéologie 24: 83–87.

[B145] Bouallam S Maaroufi A Bouzidi A Badri A (1998) Efficacité des traitements chimique et biologique sur les Culicidae: effet létal du téméphos et taux de consommation par *Gambusiaaffinis*. Annales de Limnologie 34: 99–105. 10.1051/limn/1998010

[B146] Bouclier-Maurin H (1923) Le Sphinx “*Deilephilalineata*” et ses parasites dans la région de Mascara. Revue agricole de l’Afrique du Nord 212: 541–542.

[B147] Boughdad A Boumezzough A Taher A (1997) Impact of *Phytomyzaorobanchia* Kalt. (Diptera: Agromyzidae) on *Orobanchecrenata* Forsk. (Orobanchaceae) in Morocco. In: Sixth Arab Congress of Plant Protection, Beirut, Lebanon (Abstracts Book), Lebanon, 418–418.

[B148] Boulétreau-Merle J Fouillet P Terrier O (1992) Clinal and seasonal variations in initial retention capacity of virgin *Drosophilamelanogaster* females as a strategy for fitness. Evolutionary Ecology 6: 223–242. 10.1007/BF02214163

[B149] Boumezzough A (1996) Elements de biologie, d’écologie de *Phytomyzaorobanchia* Kalt. (Dipt., Agromyzidae) et Statut de l’infestion d’*Orobanchecrenata* Forsk. dans les Régions du Saïss et Rommani. Mémoire de troisième Cycle en Agronomie. Option: Protection des Plantes. Département de Zoologie agricole, École nationale d’Agriculture de Meknès, Maroc, xii + 70 pp, Meknès.

[B150] Boumezzough A Vaillant F (1986a) Les Diptères DolichopdidaeHydrophorinae du Maroc. L’entomologiste 42(5): 295–300.

[B151] Boumezzough A Vaillant F (1986b) Quelques diptères Psychodidae, Psychodinae du Grand-Atlas marocain. L’entomologiste 424: 237–239.

[B152] Boumezzough A Thomas AGB (1987) *Chrysopilustsacasi* Thomas, 1979: morphologie et écologie des larves (Diptera, Rhagionidae). Bulletin de la Société d’histoire naturelle 123: 85–87.

[B153] Boumezzough A Sibold B Alves-Pires C Marquez F Glasser N Pesson B (2009) Phlebotomine sandflies (Diptera: Psychodidae) of the genus *Sergentomyia* in Marrakech region, Morocco. Parasitology Research 104(5): 1027–1033. 10.1007/s00436-008-1285-9 19043738

[B154] Bounamous A (2010) Biosystematique et caractérisation par la biologie moléculaire des phlébotomes de l’Est algérien. Thèse de Doctorat Es Sciences, Université Mentouri de Constantine, 302 pp.

[B155] Bourquia M Claire Garros C Rakotoarivony I Gardès L Huber K Boukhari I Delécolle JC Baldet T Mignotte A Lhor Y Khallaayoune K Balenghien T (2019) Update of the species checklist of *Culicoides* Latreille, 1809 biting midges (Diptera: Ceratopogonidae) of Morocco. Parasites Vectors 12: e459. 10.1186/s13071-019-3720-4 PMC6757417 31551074

[B156] Boussaa S (2008) Epidémiologie des leishmanioses dans la région de Marrakech, Maroc: effet de l’urbanisation sur la répartition spatio-temporelle des Phlebotomes et caractérisation moléculaire de leurs populations. Thèse de Doctorat, Université Louis Pasteur Strasbourg I. France, 181 pp.

[B157] Boussaa S Guernaoui S Pesson B Boumezzough A (2005) Seasonal fluctuations of phlebotomine sand fly populations (Diptera: Psychodidae) in the urban area of Marrakech, Morocco. Acta Tropica 95(2): 86–91. 10.1016/j.actatropica.2005.05.002 15985259

[B158] Boussaa S Pesson B Boumezzough A (2007) Phlebotomine sandflies Diptera: Psychodidae) of Marrakech city, Morocco. Annals of tropical medicine and parasitology 101(8): 715–724. 10.1179/136485907X241398 18028733

[B159] Boussaa S Boumezzough A Remy PE Glasser N Pesson B (2008) Morphological and isoenzymatic differentiation of *Phlebotomusperniciosus* and *Phlebotomuslongicuspis* (Diptera: Psychodidae) in Southern Morocco. Acta Tropica 106(3): 184–189. 10.1016/j.actatropica.2008.03.011 18456222

[B160] Boussaa S Boumezzough A Sibold B Alves-Pires C Morillas Marquez F Glasser N Pesson B (2009) Phlebotomine sandflies (Diptera: Psychodidae) of the genus *Sergentomyia* in Marrakech region, Morocco. Parasitology Research 104(5): 1027–1033. 10.1007/s00436-008-1285-9 19043738

[B161] Boussaa S Neffa M Pesson B Boumezzough A (2010) Phlebotomine sandflies (Diptera: Psychodidae) of southern Morocco: Results of entomological surveys along the Marrakech–Ouarzazate and Marrakech–Azilal roads. Annals of tropical medicine and parasitology 104(2): 163–170. 10.1179/136485910X12607012374235 20406583

[B162] Bouzidi A (1989) Recherches Hydrobiologiques sur les cours d’eau des Massifs du Haut Atlas (Maroc): Bio-écologie des Macroinvertébrés et Distribution Spatiale des Peuplements. Thèse d’Etat, Université d’Aix–Marseille III, 190 pp.

[B163] Bouzidi A Giudicelli J (1986) Contribution à l’étude faunistique et écologique des simulies (Diptera, Simuliidae) du Maroc. I. Une nouvelle espèce du Haut-Atlas: Simulium (Nevermannia) toubkal n.sp. Annales de Limnologie 22: 41–52. 10.1051/limn/1986005

[B164] Bouzidi A Giudicelli J (1988) Contribution à l’étude faunistique et écologique des simulies (Diptera, Simuliidae) du Maroc. II. Simulium (Obuchovia) marocanum n.sp. et les espèces méditerranéennes d’*Obuchovia* Rubzov. Annales de Limnologie 23: 185–195. 10.1051/limn/1987017

[B165] Brake I (2000) Phylogenetic systematics of the Milichiidae (Diptera, Schizophora). Entomologica scandinavica Supplement 57: 1–120.

[B166] Brake I (2011) World Catalog of the Family Carnidae (Diptera, Schizophora). Myia 12: 113–169.

[B167] Brehm A Harris DJ Hernandez M Perez JA Larruga JM Pinto FM Gonzalez AM (2004) Phylogeography of *Drosophilasubobscura* from north Atlantic islands inferred from mtDNA A + T rich region sequences. Molecular Phylogenetics and Evolution 30: 829–834. 10.1016/j.ympev.2003.10.018 15012961

[B168] Brémond P (1938) , Recherches sur la biologie de *Lixusjunci* Boeh., charançon nuisible à la betterave au Maroc. Revue de Pathologie végétale et d’Entomologie agricole de France 25: 59–73.

[B169] Brémond P Rungs C (1938) Observations sur la pyrale dorée (*Pyraustaauratameridionalis* Stgr.), ravageur de la menthe cultivée au Maroc. Revue de Pathologie végétale et d’Entomologie agricole de France 25: 190–194.

[B170] Brunetti EA (1926) New and little-known Cyrtidae (Diptera). Annals and Magazine of natural History 18(9): 561–606. 10.1080/00222932608633552

[B171] Bruun HH Jørgensen J Skuhravá M (2012) Nineteen species of gall midges (Diptera: Cecidomyiidae) new to Denmark. Entomologiske Meddelelser 80(2): 87–98.

[B172] Cabrera VM Gonzalez AM Larruga JM Vega C (1983) Linkage disequilibrium in chromosome A of *Drosophilasubobscura*. Genetica 61: 3–8. 10.1007/BF00563226

[B173] Callot J (1940) Sur quelques moustiques du Maroc. Annales de l’Institut Pasteur du Maroc 2: 361–665.

[B174] Callot J Kremer M Bailly-Choumara H (1968) Preliminary faunistic note on *Culicoides* (Dipt., Ceratopogonides) of Morocco. Gynandromorphism of *C.circumscriptus* parasited by a Mermis. Bulletin de la Société de pathologie exotique et de ses filiales 61(6): 885–889. 5757327

[B175] Callot J Kremer M Bailly-Choumara H (1970) Description de *Culicoidescoluzzii* n. sp. (Diptera, Ceratoponidae). Bulletin de la Société zoologique de France 95(4): 709–718.

[B176] Calvo D Molina JMª (2002) First Iberian record of *Drinomaroccana* Mesnil, 1951 (Diptera, Tachinidae, Exoristinae), a parasitoid of *Streblotepanda* Hübner, [1820] (Lasiocampidae) caterpillars. Graellsia 58(1): 85–86. 10.3989/graellsia.2002.v58.i1.270

[B177] Canzoneri S Meneghini D (1966) *Lispe* Latreille del Mediterraneo e Medio Oriente raccolte da A. Giordani Soika. Bollettino del Museo civico di Storia naturale di Venezia 16 [1963]: 109–148.

[B178] Canzoneri S Meneghini D (1972) Nuovo contributo alla conoscenza del genere *Lispe* Latr. Bollettino del Museo civico di Storia naturale di Venezia 22–23[1969–1970]: 211–214.

[B179] Capy P Koga A David JR Hartl DL (1992) Sequence Analysis of Active mariner Elements in Natural Populations of *Drosophilasimulans*. Genetics 130: 499–506. 10.1093/genetics/130.3.499 1312979 PMC1204867

[B180] Capy P Pla E David JR (1993) Phenotypic and genetic variability of morphometrical traits in natural populations of *Drosophilamelanogaster* and *D.simulans*. I. Geographic variation. Genetics Selection Evolution 25: 517–536. 10.1186/1297-9686-25-6-517

[B181] Carles-Tolrá M (1993) New and interesting records of DipteraAcalyptrata from Spain. Part III: Lauxaniidae, Chamaemyiidae, Coelopidae, Dryomyzidae, Sciomyzidae and Sepsidae. Fragmenta Entomologica 25(1): 21–41.

[B182] Carles-Tolrá M (2001) Two new species of *Scenopinus* Latreille from Spain (Diptera, Scenopinidae). Boletín de la Asociación española de Entomología 25(1–2): 35–41.

[B183] Carles-Tolrá M (2002) Catálogo de los Diptera de España, Portugal y Andorra (Insecta). Monografías SEA (Sociedad Entomológica Aragonesa) 8: 1–323. [Zaragoza]

[B184] Carles-Tolrá M (2015) Estudio morfológico de las antenas de *Syllegomydasbueni* Arias y eliminación de *Syllegomydasalgiricus* Gerstaecker de la fauna europaea (Diptera: Mydidae). Boletín de la Sociedad Entomológica Aragonesa 56: 367–369.

[B185] Carles-Tolrá M (2017) Sobre la identidad de *Syllegomydascinctus* (Macquart, 1835) y *Syllegomydasbueni* Arias, 1914 (Diptera: Mydidae). Boletín de la Sociedad Entomológica Aragonesa 60: 259–276.

[B186] Carnevale P Trari B Izri A Manguin S (2012) Les cinq piliers de la protection familiale et personnelle de l’homme contre les moustiques vecteurs d’agents pathogènes. Médecine et Santé Tropicales 22: 13–21. 10.1684/mst.2012.0004 22868720

[B187] Cassar LF Gatt P Lanfranco E Lanfranco S Mallia A (2005) Smir Lagoon (Northern Morocco) and its surroundings: an environmental management approach. In: Bayed A Scapini F (Eds) Ecosystèmes côtiers sensibles de la Méditerranée: cas du littoral de Smir. Travaux de l’Institut Scientifique, Rabat.Série générale 4: 65–74.

[B188] Cassar LF Conrad ES Gatt P Lanfranco E Rolé A (2008) The termo-Mediterranean biotopes of the Oued Laou basin: a landscape approach. In: Bayed A Ater M (Eds) Du bassin versant vers la mer: analyse multidisciplinaire pour une gestion durable. Travaux de l’Institut Scientifique, Rabat.Série générale 5: 17–26.

[B189] Catania F Kauer MO Daborn PJ Yen JL French-Constant RH Schlötterer C (2004) World-wide survey of an Accord insertion and its association with DDT resistance in *Drosophilamelanogaster*. Molecular Ecology 13: 2491–2504. 10.1111/j.1365-294X.2004.02263.x 15245421

[B190] Černý M (2007) Two new species of the genus *Cerodontha* (Diptera: Agromyzidae). Folia Heyrovskyana 14(3): 95–104.

[B191] Černý M (2009) New faunistic data on the Agromyzidae (Diptera) from the West Palaearctic Region. Klapalekiana 45(1–2): 9–21.

[B192] Černý M (2011) A review of the species of *Cerodontha* Rondani (Diptera: Agromyzidae) of Israel, with a new species of the subgenus Poemyza Hendel, 1931. Israel Journal of Entomology 40(2010): 117–143.

[B193] Černý M (2012) The fauna of Agromyzidae (Diptera) in the Gemer region (Central Slovakia), with descriptions of three new species from Slovakia. Časopis Slezského zemského muzea, Opava (A) 61: 49–76.

[B194] Černý M (2013) Additional records of Agromyzidae (Diptera) from the West Palaearctic Region. Časopis Slezského zemského muzea, Opava (A) 62: 281–288.

[B195] Černý M (2019) Additional new records of Agromyzidae (Diptera) from the Palaearctic Region. Acta Musei Silesiae, Scientiae naturales 67(2018): 117–137. 10.2478/cszma-2018-0010

[B196] Černý M Merz B (2006) New records of Agromyzidae (Diptera) from the Palaearctic Region. Mitteilungen der schweizerischen entomologischen Gesellschaft 79(1): 77–106.

[B197] Černý M Merz B (2007) New records of Agromyzidae (Diptera) from the West Palaearctic Region, with an updated checklist for Switzerland. Mitteilungen der schweizerischen entomologischen Gesellschaft 80(1/2): 107–121.

[B198] Černý M von Tschirnhaus M (2014) New records of Agromyzidae (Diptera) from the Afrotropical Region, with a checklist. Acta Musei Silesiae, Scientiae naturales 63(2): 159–176. 10.2478/cszma-2014-0017

[B199] Černý M von Tschirnhaus M Winqvist K (2020) First records of Palaearctic Agromyzidae (Diptera) from 40 countries and major islands. Acta Musei Silesiae, Scientiae naturales 69(3): 193–229. 10.2478/cszma-2020-0017

[B200] Cerretti P (2005) Revision of the West Palaearctic species of the genus *Pales* Robineau-Desvoidy (Diptera: Tachinidae). Zootaxa 885: 1–36. 10.11646/zootaxa.885.1.1

[B201] Cerretti P Ziegler J (2004) Chorologic data on tachinid flies from mainland Greece (Diptera, Tachinidae). Fragmenta entomologica 36: 275–317.

[B202] Cerretti P Tschorsnig HP (2007) Two new species of *Siphona* Meigen (Diptera: Tachinidae) from Sardinia and Morocco. Stuttgarter Beiträge zur Naturkunde, Serie A (Biologie) 704: 1–7.

[B203] Cerretti P Tschorsnig HP (2008) A new species of *Plesina* Meigen (Diptera: Tachinidae) from the Mediterranean. Stuttgarter Beiträge zur Naturkunde A. Neue Serie 1: 445–450.

[B204] Cerretti P Tschorsnig HP (2012) Three new species of *Estheria* Robineau-Desvoidy (Diptera: Tachinidae) from the Mediterranean, with a key to the European and Mediterranean species of the genus. Stuttgarter Beiträge zur Naturkunde A. Neue Serie 5: 271–286.

[B205] Cerretti P Badano D Gisondi S Lo Giudice L Pape T (2020) The world woodlouse flies (Diptera: Rhinophoridae). ZooKeys 903: 1–130. 10.3897/zookeys.903.37775 31997887 PMC6976704

[B206] Cêtre-sossah C Baldet T (2004) Mission d’expertise entomologique et virologique, fièvre catarrhale ovine – Maroc. Rapport de mission à l’IAH Hassan II. Montpellier: Cirad, 12 pp.

[B207] Chaker E Bailly-Choumara H Kremer M (1979) Sixième contribution à l’étude faunistique des *Culicoides* du Maroc (Diptera, Ceratopogonidae). Bulletin de l’Institut scientifique, Rabat 4: 81–86.

[B208] Chaker E Bailly-Choumara H Kremer M (1980) Sixième contribution à l’étude faunistique des *Culicoides* du Maroc (Diptera, Ceratopogonidae). Bulletin de l’Institut scientifique, Rabat 4: 81–86.

[B209] Chakir M Chafik A Gibert P David JR (2002) Phenotypic plasticity of adult site and pigmentation in *Drosophila*: thermosensitive periods during development in two sibling species. Journal of Thermal Biology 27: 61–70. 10.1016/S0306-4565(01)00016-X

[B210] Chakir M Moreteau B Capy P David JR (2007) Phenotypic variability of wild living and laboratory grown *Drosophila*: Consequences of nutritional and thermal heterogeneity in growth condition. Journal of Thermal Biology 32: 1–11. 10.1016/j.jtherbio.2006.06.001

[B211] Chakir M Negoua H Moreteau B David JR (2008) Quantitative morphometrical analysis of a North African population of *Drosophilamelanogaster*: sexual dimorphism and comparison with European populations. Journal of Genetics 87: 373–382. 10.1007/s12041-008-0060-0 19147927

[B212] Chakir M Negoua H Capy P David JR (2011) Phenotypic variability and sex dimorphism in *Drosophila* (Diptera: Drosophilidae): comparison of wild and laboratory grown adults of two sympatric cosmopolitan species. Annales de la Société entomologique (N.S.) 47(3–4): 371–383. 10.1080/00379271.2011.10697731

[B213] Chakrani F Capy P David JR (1993) Developmental temperature and somatic excision rate of mariner transposable element in three natural populations of *Drosophilasimulans*. Genetics Selection Evolution 25: 121–132. 10.1186/1297-9686-25-2-121

[B214] Chandler PJ (1987) The families Diastatidae and Campichoetidae (Diptera, Drosophiloidea) with a revision of Palaearctic and Nepalese species of *Diastata* Meigen. Entomologica scandinavica 18: 1–50. 10.1163/187631287X00016

[B215] Chandler PJ (1994) Fungus gnats of Israel (Diptern: Sciaroidea, excluding Sciaridae). Israel Journal of Entomology 28: 1–100.

[B216] Chandler PJ (2001) The Flat-footed Flies (Dipetra: Opetiidae and Platypezidae) of Europe. Fauna entomologica Scandinavica 36: 1–278. 10.1163/9789047400776

[B217] Chandler PJ Ribeiro E (1995) The Sciaroidea (Diptera) of the Atlantic Islands (Canary Islands, Madeira and the Azores). Boletim do Museu Municipal do Funchal (História Natural), Suplemento No. 3, 88 pp.

[B218] Chandler PJ Gatt P (2000) Fungus Gnats (Diptera: Bolitophilidae, Keroplatidae and Mycetophilidae) from the Maltese islands. Studia dipterologica 7(1): 69–81.

[B219] Chandler PJ Blasco-Zumeta J (2001) The fungus gnats (Diptera, Bolithophilidae, Keroplatidae and Mycethophilidae) of the Monegros region (Zaragoza, Spain) and five other new European species of *Pyratula* Edwards and *Sciophila* Meigen. Zapateri, Revista aragonesa de Entomología 9: 1–24.

[B220] Chandler PJ Bechev DN Caspers N (2005) The Fungus Gnats (Diptera: Bolitophilidae, Diadocidiidae, Ditomyiidae, Keroplatidae and Mycetophilidae) of Greece, its islands and Cyprus. Studia dipterologica 12: 255–314.

[B221] Charlat S Le Chat L Merçot H (2003) Characterization of non-cytoplasmic incompatibility inducing *Wolbachia* in two continental African populations of *Drosophilasimulans*. Heredity 90: 49–55. 10.1038/sj.hdy.6800177 12522425

[B222] Charrier H (1924) Les Moustiques de la région de Tanger. Bulletin de la Société de pathologie exotique et de ses filiales 17: 570–572.

[B223] Charrier H (1927) Note préliminaire sur les mouches de la région de Tanger. Bulletin de la Société de pathologie exotique 20(7): 619–622.

[B224] Chillasse L Dakki M (2004) Potentialités et statuts de conservation des zones humides du Moyen-Atlas (Maroc), avec référence aux influences de la sécheresse. Sécheresse №4, 15: 337–345.

[B225] Chlaida M (1997) Étude hydrobiologique des berges de la retenue du barrage Al Massira (sud-est de Casablanca). Physicochimie, structure des peuplements des macroinvertébrés, dynamique des populations culicidiennes (Moustiques) et cartographie de leurs gîtes larvaires. Thèse de Doctorat d’Etat, Université Hassan II, Mohammedia, Casablanca, 216 pp.

[B226] Chlaida M Bouzidi A (1995) Contribution à l’étude des Culicidés du Maroc: Dynamique et cartographie écologique de quelques espèces au sein de la retenue de barrage Al Massira (Sud de Settat). Bulletin de l’Institut scientifique, Rabat 19: 83–92.

[B227] Chvála M (1969) Revision of Palaearctic species of the genus *Tachydromia* Meigen (= *Tachista* Loew) (Diptera, Empididae). Acta Entomologica Musei Nationalis Pragae 38: 415–524.

[B228] Chvála M (1981) Empididae (Insecta: Diptera) from Southern Spain, with Descriptions of Twenty New Species and Notes on Spanish Fauna. Steenstrupia 7(6): 113–177.

[B229] Chvála M (2008) Monograph of the genus *Hilara* Meigen (Diptera: Empididae) of the Mediterranean region. Studia dipterologica, Supplement 15: 1–138.

[B230] Chvála M Lyneborg L (1970) A revision of Palaearctic Tabanidae (Diptera) described by J.C. Fabricius. Journal of Medical Entomology 7: 543–555. 10.1093/jmedent/7.5.543 5501214

[B231] Chvála M Lyneborg L Moucha J (1972) The horse flies of Europe (Diptera, Tabanidae). Entomological Society of Copenhagen, Copenhagen, 499 pp.

[B232] Chvála M Kovalev VG (1989) Family Hybotidae. Vol. 6. In: Soós Á Papp L (Eds) Catalogue of Palaearctic Diptera (Therevidae – Empididae).Akadémiai Kiadó, Budapest, 174–227. [435 pp]

[B233] Chvála M Wagner R (1989) Family Empididae. In: Soós Á Papp L (Eds) Catalogue of Palaearctic Diptera.Vol. 6: Therevidae – Empididae. Akadémiai Kiadó, Budapest, 228–336. [435 pp]

[B234] Claußen C (1989a) Das bisher unbeschriebene Männchen von *Cheilosiarodgersi* Wainwright aus Südspanien (Diptera, Syrphidae). Entomologische Zeitung 99(19): 283–288.

[B235] Claußen C (1989b) Syrphiden aus Marokko (Diptera, Syrphidae). Entomofauna 10(24): 357–375.

[B236] Claußen C Hauser M (1990) Neue Syrphidenvorkommen aus Marokko und Tunesien (Diptera, Syrphidae). Entomofauna 11(25): 433–438.

[B237] Claußen C Speight MCD (2007) Names of uncertain application and some previously unpublished synonyms, in the European *Cheilosia* fauna (Diptera, Syrphidae). Volucella 8: 73–86.

[B238] Clements DK (2000) *Myopavaulogeri* (Séguy) a synonym of *M. minor* Strobl, with a redescription (Dipt., Conopidae). Entomologist´s Monthly Magazine 136: 235–240.

[B239] Clergue-Gazeau M Lek S Lek S (1991) Les simulies d’Afrique du Nord: nouvelles données sur la répartition de la faune du Maroc et biogéographie des espèces maghrébines (Diptera, Simuliidae). Revue d’Hydrobiologie Tropicale 24: 47–59.

[B240] Coetzer JAW Guthrie AJ (2004) African horse sickness. In: Coetzer JAW Tustin RC (Eds) Infectious diseases of livestock.2^nd^ edn. Oxford University Press, Cape Town, 1231–1246.

[B241] Colacicco-Mayhugh MG Masuoka PM Grieco JP (2010) Ecological niche model of *Phlebotomusalexandri* and *P.papatasi* (Diptera: Psychodidae) in the Middle East. International Journal of Health Geographics 9: e2. 10.1186/1476-072X-9-2 PMC2823717 20089198

[B242] Coliado JG (1929) Sirfidos de Marruecos del Musco de Madrid. Memorias de la Real Sociedad Española de Historia Natural, XII.

[B243] Collin JE (2009) A New species of *Rhamphomyia* Meigen (Diptera: Empididae) from Morocco. Proceedings of the Royal Entomological Society of London. Series B, Taxonomy 22(3–4): 57–58. 10.1111/j.1365-3113.1953.tb00057.x

[B244] Constanti M Pascual M Ribo G Prevosti A (1986) Sexual isolation between populations of *Drosophilasubobscura*. I. European strains. Genetica Iberica 38: 213–222.

[B245] Cooper BE O’Hara JE (1996) Diptera types in the Canadian National Collection of Insects. Part 4 Tachinidae, 94 pp.

[B246] Cornet M Brunhes J (1994) Révision des espèces de *Culicoides* apparentées à *C.schultzei* (Enderlein, 1908) dans la région afrotropicale (Diptera, Ceratopogonidae). Bulletin de la Société entomologique de France 99(2): 149–164. 10.3406/bsef.1994.17053

[B247] Costa A (1857) Contribuzione alla fauna ditterologica italiana. Giambattista Vico 2: 438–460.

[B248] Costa R Peixoto AA Barbujani G Kyriacou CP (1992) A latitudinal cline in a *Drosophila* clock gene. Proceedings of the Royal Entomological Society of London, Series B 250: 43–49. 10.1098/rspb.1992.0128 1361061

[B249] Coupland JB Barker GM (2004) Flies as predators and parasitoids of terrestrial Gastropods, with emphasis on Phoridae, Calliphoridae, Sarcophagidae, Muscidae and Fanniidae. In: Barker GM (Ed.) Natural enemies of terrestrial Molluscs.CABI Publishing, CAB International, Wallingford, 85–158. 10.1079/9780851993195.0085

[B250] Cresson ET (1939) Description of a new genus and ten new specis of Ephydridae, with a discussion of the species of the genus *Discomyza* (Diptera). Notulae Naturae 21: 1–12.

[B251] Croset H Rioux JA Maistre M Bayar N (1978) Les Phlébotomes de Tunisie (Diptera, Phlebotomidae). Mise au point systématique, chorologique et éthologique. Annales de Parasitologie (Paris) 53(6): 711–749. 10.1051/parasite/1978536711 754625

[B252] Crosskey RW (1964) Subgeneric assignment and synonymy of some southern Palaearctic species of *Simulium* (Diptera: Simuliidae). Annals and Magazine of natural History, Ser. 13(7): 665–672. 10.1080/00222936408651513

[B253] Cumming JM Cooper BE (1992) A revision of the Nearctic species of the Tachydromiine fly genus *Stilpon* Loew (Diptera: Empidoidea). The Canadian Entomologist 124(6): 951–998. 10.4039/Ent124951-6

[B254] Czerny L Strobl G (1909) Spanische Dipteren. III. Beitrag. Verhandlungen der Zoologisch-Botanischen Gesellschaft in Wien 59: 121–301.

[B255] Dahl RG (1964) Some Ephydridae and Canaceidae (Dipt., Brachycera) from Morocco and the Spanish Sahara. Notulae entomologicae 44(3): 101–104.

[B256] Dakki M (1997) Faune aquatique continentale. In: Etude Nationale sur la Biodiversité. Ministère de l’Environnement-PNUE. Maroc, 117 pp.

[B257] Dakki M Himmi O (2008) Bases Stratégiques pour la Conservation de la Biodiversité des Eaux Douces du Bassin du Sebou (Maroc). Etude menée grâce à un microfinancement du Programme WWF/MedPo ‘ACROSS THEWATERS’ (phase 5) (projet réf. 9E0647.MOR.01.P.06) 59 pp. Avec la contribution de Abdelhamid Azeroual (poissons), Boutaina Belqat (insectes diptères), Sidi Imad Cherkaoui (oiseaux nicheurs), Majida El Alami El Moutaouakil (insectes trichoptères, éphéméroptères et plécoptères), Rhimou El Hamoumi (amphibiens & reptiles), Fatima Fadil (crustacés des eaux superficielles), Soumaya Hammada (flore), Oumnia Himmi (annélides hirudinées, insectes odonates et hétéroptères), Kawtar Kettani (insectes diptères), Mohamed Messouli (crustacés phréatiques, oligochètes), Bennas Nard (insectes coléoptères), Abdeljebbar Qninba (oiseaux hivernants), Mohamed Ghamizi (insectes mollusques), Ahmed Yahyaoui (poissons).

[B258] Dakkok A Cabaret J Dudbib M (1978) Etude du cycle des hypodermes et des facteurs de risques pour les bovins dans la region de Sidi-Slimane (Maroc). Utilisation pour l’établissement d’une prophylaxie [*Hypodermalineatum*, *Hypodermabovis*, hypodermose bovine, varron; traitement]. Recueil de Médecine Vétérinaire 154: 753–760.

[B259] Daugeron C (1997) Découverte du sous-genre *Xanthempis* Bezzi en Afrique du Nord et description de trois espèces nouvelles (Diptera: Empididae). Annales de la Société entomologique de France 33(2): 155–164.

[B260] Daugeron C (2000) The subgenus Xanthempis: new species and taxonomical data (Diptera: Empididae). Annales de la Société entomologique de France 36: 371–388.

[B261] Daugeron C (2009) Systematics of the Euro-Mediterranean Empis (Kritempis) (Diptera: Empididae: Empidinae). Zootaxa 2318: 531–544. 10.11646/zootaxa.2318.1.21

[B262] David JR Bocquet C (1973) Sur certains caractères quantitatifs des souches de *Drosophilamelanogaster* provenant du Sud du Maroc. Comptes Rendus de l’Académie des Sciences Paris 277: 877–890. 4214160

[B263] David JR Merçot H Capy P McEvey SF van Herewee J (1986) Alcohol tolerance and ADH gene frequencies in European and African populations of *Drosophilamelanogaster*. Genetics Selection Evolution 18: 405–416. 10.1186/1297-9686-18-4-405 PMC2713961 22879258

[B264] David JR Gibert P Moreteau B Gilchrist GW Huey RB (2003) The fly that came infrom the cold: geographic variation of recovery time from low temperature exposure in *Drosophilasubobscura*. Functional Ecology 17: 425–430. 10.1046/j.1365-2435.2003.00750.x

[B265] Dawah HA (2011) Some Tachinidae (Diptera: Calyptrata) from South Western Saudi Arabia. Journal of Jazan University, Applied Sciences Branch 1(1): 28–38.

[B266] Dawah HA Abdullah MA (2006) New Records of Chloropidae (Diptera) from Southwest Saudi Arabia with some Biological Information, World-wide Geographical Distribution and Taxonomic Features. Saudi Journal of biological Sciences 13(1): 24–34 [English], 132–192 [Arabic].

[B267] Dawah HA Abdullah MA Kamran Ahmad S (2019) An Overview of the Calliphoridae (Diptera) of Saudi Arabia with New Records and Updated List of Species. Journal of the Entomological Research Society 21(1): 65–93.

[B268] Dawah HA Syed Kamran A Abdullah MA Grichanov IYa (2020) The family Dolichopodidae (Diptera) of the Arabian Peninsula: identification key, an updated list of species and new records from Saudi Arabia. Journal of Natural History 54: 21–22. 10.1080/00222933.2020.1800118

[B269] Deeming JC Al-Dhafer HM (2012) Chloropidae from the Arabian Peninsula (Diptera: Cyclorrhapha). Zoology in the Middle East 58: 3–88. 10.1080/09397140.2012.10648977

[B270] De Jong H (1993) The phylogeny of the *Nephrotomaflavescens* species group (Diptera: Tipulidae). Tijdschrift voor Entomologie 136: 235–256.

[B271] De Jong H (1994a) The phylogeny of the Tipula (Acutipula) maxima species group, with notes on its distribution (Diptera: Tipulidae). Entomologica scandinavica 24: 433–457. 10.1163/187631293X00208

[B272] De Jong H (1994b) The phylogeny of the subgenus Tipula (Savtshenkia) (Diptera: Tipulidae), with special reference to the western Mediterranean fauna. Tijdschrift voor Entomologie 137: 271–323.

[B273] De Jong H (1995) The phylogeny of the Tipula (Lunatipula) bullata and *falcata* species groups (Diptera: Tipulidae). Tijdschrift voor Entomologie 138: 245–267.

[B274] De Jong H (1998) In search of historical biogeographic patterns in the western Mediterranean terrestrial fauna. Biological Journal of the Linnean Society 65: 99–164. 10.1006/bijl.1998.0239

[B275] De Jong H Adghir A Bosch EJ Kettani K (2021) Taxonomy of *Nephrotomaguestfalica* (Westhoff, 1879) (Diptera, Tipulidae), with the description of a new subspecies from Morocco. Tijdschrift voor Entomologie 163: 31–45. 10.1163/22119434-20192086

[B276] Delanoë P (1917) Contribution à l’étude du paludisme au Maroc Occidental. Bulletin de la Société de pathologie exotique 10: 586–611.

[B277] Delanoë P (1922) Myiases du bétail du cercle des Doukkala causées par les larves d’une mouche sarcophile *Wohlfahrtiamagnifica* Schiner, 1862. Bulletin de la Société des sciences naturelles de Maroc 2: 132–136.

[B278] De Lépiney J (1930) Contribution à l’étude du complexe biologique de *Lymantriadispar*. Mémoires de la Société des sciences naturelles du Maroc 23: 100 pp.

[B279] De Lépiney J Mimeur JM (1932) Notes d’entomologie agricole et forestière du Maroc. Mémoires de la Société des sciences naturelles du Maroc 31: [III +] 195 pp. [Rabat, Paris, London]

[B280] De Meyer M (2000) Systematic revision of the subgenus Ceratitis MacLeay s.s. (Diptera, Tephritidae). Zoological Journal of the Linnaean Society 128: 439–467. 10.1111/j.1096-3642.2000.tb01523.x

[B281] Depaquit J Hadj-Henni L Bounamous A Strutz S Boussaa S Morillas-Marquez F Pesson B Gállego M Delécolle JC Afonso MO Alves-Pires C Capela RA Couloux A Léger N (2015) Mitochondrial DNA Intraspecific Variability in *Sergentomyiaminuta* (Diptera: Psychodidae). Journal of Medical Entomology 52(5): 819–828. 10.1093/jme/tjv075 26336215

[B282] De Zulueta J Ramsdale CD Cianchi R Bullini L Coluzzi M (1983) Observations on the taxonomic status of *Anophelessicaulti*. Parasitologia 23: 73–92. 6543939

[B283] Dieringer D Nolte V Schlötterer C (2005) Population structure in African *Drosophilamelanogaster* revealed by microsatellite analysis. Molecular Ecology 14: 563–573. 10.1111/j.1365-294X.2004.02422.x 15660946

[B284] Dike MC (1990) Two new species of *Atherigona* from Nigeria with a key for the identification of Afrotropical species of the subgenus Acritochaeta (Diptera: Muscidae). Systematic Entomology 15(3): 297–303. 10.1111/j.1365-3113.1990.tb00065.x

[B285] Dikow T (2017) Mydidae. Asiloid Flies, deciphering their diversity and evolutionary history. Smithsonian, National Museum of natural History. http://www.asiloidflies.si.edu [accessed May 2017]

[B286] Dils J (2008) A new species of *Chalcochiton* (Diptera: Bombyliidae) from Morocco. Phegea 36(1): 31–33.

[B287] Dils J (2012) A new species of *Thyridanthrax* from Morocco (Diptera: Bombyliidae). Phegea 40(1a): 5–6.

[B288] Dils J (2013) Remarks on *Conophorusheteropilosus* (Diptera: Bombyliidae). Phegea 41(3): 61–62. [ISSN 0771-5277] 10.1016/j.biologicals.2012.07.008

[B289] Dils J (2017) A new species of *Chalcochiton* (Diptera: Bombyliidae) from Morocco. Phegea 45(3): 75–78.

[B290] Dils J Özbek H (2006) Contribution to the Knowledge of the Bombyliidae of Turkey (Diptera). Linzer biologische Beiträge 38(1): 455–505.

[B291] Dirickx HG (1994) Atlas des Diptères syrphides de la région méditerranéenne. Doc. de Travail, Institut royal des sciences naturelles de Belgique 75: 1–314.

[B292] Disney RHL (1984) The holotype of *Megaseliaminor* (Zett.) (Dipt., Phoridae) and two new synonyms. Entomologist’s Monthly Magazine 120: 239–240.

[B293] Djellab S Van Eck A Samraoui B (2013) A survey of the hoverflies of northeastern Algeria (Diptera: Syrphidae). Egyptian Journal of Biology 15: 1–12. 10.4314/ejb.v15i1.1

[B294] Dominiak P (2012) Biting midges of the genus *Dasyhelea* Kieffer (Diptera: Ceratopogonidae) in Poland. Polish Journal of Entomology 81(3): 211–304. 10.2478/v10200-012-0009-8

[B295] Dousti AF Hayat R (2006) A Catalogue of the Syrphidae (Insecta: Diptera) of Iran. Journal of the Entomological Research Society 8(3): 5–38.

[B296] Dowling C (1983) A description of Two New species of Tanypodinae (Diptera, Chironomidae) from North Africa. Memories of American Entomological Society 34: 89–94.

[B297] Dowling C (1987) A description of two new species of the genus *Thienemannimyia* (Diptera, Chironomidae) from North Africa. Entomologica scandinavica Supplements 29: 157–160.

[B298] Draber-Mońko A (2011) State of knowledge of the tachinid fauna of Eastern Asia, with new data from North Korea. Part IE. Tachininae. Fragmenta Faunistica 54(2): 157–177. 10.3161/00159301FF2011.54.2.157

[B299] Driauach O Belqat B (2015) A new species of the genus *Baeoura* from Morocco, with a key to the West Palaearctic species (Diptera, Tipuloidea, Limoniidae). ZooKeys 532: 99–105. 10.3897/zookeys.532.5994 PMC4668895 26692808

[B300] Driauach O Belqat B (2016) Additions to the Limoniidae and Pediciidae fauna of Morocco, with an updated checklist (Diptera, Tipuloidea). ZooKeys 563: 129–146. 10.3897/zookeys.563.7384 PMC4797214 27047241

[B301] Driauach O Belqat B De Jong H (2013) A First Checklist of the short-palped Crane flies (Diptera: Limoniidae, Pediciidae) of Morocco. Boletín de la Sociedad Entomológica Aragonesa 53: 187–190.

[B302] Driauach O Krzemińsk E Belqat B (2015) Genus *Trichocera* in Morocco: first records from Africa and a new species (Diptera: Trichoceridae). Zootaxa 4059(1): 181–190. 10.11646/zootaxa.4059.1.10 26701560

[B303] Droz B Haenni JP (2011) Une mouche pupipare nouvelle pour la faune de Suisse (Diptera, Hippoboscidae). Entomologica Helvetica 4: 59–63.

[B304] Dsouli N (2009) Contribution à la phylogénie du genre *Stomoxys* (Diptera, Muscidae) et à la phylogéographie de *Stomoxyscalcitrans* (L., 1758). Université Montpellier III–PAUL VALERY. Arts et Lettres, Langues et Sciences Humaines et Sociales. Département de Biologie- Écologie- Environnement, 157 pp.

[B305] Duda O (1932–1933) 61. Chloropidae. In: Lindner E (Ed.) Die Fliegen der paläarktischen Region VI, 1: [iv +] 248 pp. [pl. i–iii] [p. 49–248 and plates were published in 1933] [E. Schweizerbart, Stuttgart]

[B306] Dufour C (2003) Descriptions of four new species of Tipulidae from the Alpes Maritimes in southern France (Diptera, Tipulidae). Bulletin de la Société neuchâteloise des sciences naturelles 126: 69–80.

[B307] Duisit MJ (1960) Localisations nouvelles de Stratiomyiidae au Maroc. Société des sciences naturelles et physiques du Maroc №7, 121.

[B308] Dupuis C (1952) Contributions à l’étude des Phasiinae cimicophages. XIV. Hôtes inédits et localités nouvelles. Annales de Parasitologie humaine et comparée 27: 332–338. 10.1051/parasite/1952271329 14944074

[B309] Dupuis C (1953) Contribution à l’étude des Phasiinae cimicophages (DipteraLarvaevoridae). XV. Données sur les *Leucostomatina* et, en particulier, *Leucostomaanalis* (Meigen) s. str. Annales de Parasitologie humaine et comparée 28: 64–97. 10.1051/parasite/1953281064 13080827

[B310] Dupuis C (1963) Essai monographique sur les Phasiinae. Mémoires du Muséum national d’histoire naturelle. Nouvelle série, série A. Zoologie 26: 1–461.

[B311] Du Toit RM (1944) The transmission of bluetongue and horse sickness by *Culicoides*. Onderstepoort Journal of Veterinary 19: 7–16.

[B312] Ebejer MJ (1998a) A Review of the Palaearctic Species of *Aphaniosoma* Becker (Diptera, Chyromyidae), with Descriptions of New Species and a Key for the Identification of Adults. Mitteilungen Museum für Naturkunde Berliner Deutsche entomologische Zeitschrift 45(2): 191–230. 10.1002/mmnd.4810450208

[B313] Ebejer MJ (1998b) A new species of *Gymnochiromyia* Hendel (Diptera: Chyromyidae) from the Mediterranean, with notes, lectotype designations and a key to the species from the West Palaearctic. Studia dipterologica 5(1): 19–29.

[B314] Ebejer MJ (2002) Fauna Europaea. Diptera, Chyromyidae. http://www.faunaeur.org

[B315] Ebejer MJ (2015) The picture-winged flies and related families (Diptera, Tephritoidea) of the Maltese Islands. Bulletin of the Entomological Society of Malta 7: 73–91.

[B316] Ebejer MJ (2016a) The first record of the genus *Melanochthiphila* Frey (Diptera: Chamaemyiidae) from the Palaearctic, and new data on other Chamaemyiidae from Morocco. Studia dipterologica 22(1) 2015: 111–120.

[B317] Ebejer MJ (2016b) The Moroccan species of Chyromyidae (Diptera) with descriptions of five new species of *Aphaniosoma* Becker. Zootaxa 4208(3): 221–236. 10.11646/zootaxa.4208.3.2 27988523

[B318] Ebejer MJ (2018) Recent additions to the Chyromyidae of Morocco with description of a new species of *Gymnochiromyia* Hendel (Diptera: Acalyptrata). Entomologist´s Monthly Magazine 154: 207–212. 10.31184/M00138908.1543.3940

[B319] Ebejer MJ (2019a) Taxonomic notes on West Palaearctic species of *Lauxania* Latreille, *Sapromyza* Fallén, *Calliopum* Strand and *Minettia* Robineau-Desvoidy, with a description of a new species of *Minettia* (Diptera, Acalyptrata: Lauxaniidae) from Morocco. Zootaxa 4543(1): 037–051. 10.11646/zootaxa.4543.1.2 30647311

[B320] Ebejer MJ (2019b) A new species of *Micropeza* Meigen from Morocco and a provisional key to the West Palaearctic species (Diptera: Acalyptrata, Micropezidae). Entomologist´s Monthly Magazine 155(2): 71–76. 10.31184/M00138908.1552.3977

[B321] Ebejer JM Bensusan K (2010) Hoverflies (Diptera, Syrphidae) recently encountered on Gibraltar, with two new species for Iberia. Dipterists Digest 17: 123–139.

[B322] Ebejer MJ Kettani K (2016) An overview of the Chloropidae (Diptera) of Morocco with new records, description of a new species of *Pselaphia* (Becker) and an updated list of species. Entomologist´s Monthly Magazine 152(4): 225–244.

[B323] Ebejer MJ Kettani K (2019a) A review of the Moroccan species of Lauxaniidae (Diptera: Acalyptrata). Entomologist’s Monthly Magazine 155(3): 139–150. 10.31184/M00138908.1553.3986

[B324] Ebejer MJ Kettani K (2019b) The Pipunculidae, a neglected family of Diptera (Insecta) in Morocco. Dipterists Digest 26(1): 13–18.

[B325] Ebejer MJ Kettani K Gatt P (2019) First records of families and species of Diptera (Insecta) from Morocco. Boletín de la Sociedad Entomológica Aragonesa 64: 143–153.

[B326] Edwards FW (1921) New species of Palaearctic Simuliidae in the British Museum (DipteraNematocera). Annals and Magazine of natural History 9(7): 141–143. 10.1080/00222932108632496

[B327] Efetov KA Tarmann GM (1999) Forester moths. The genera Theresimima Strand, 1917, Rhagades Wallengren, 1863, Jordanita Verity, 1946, and Adscita Retzius, 1783 (Lepidoptera: Zygaenidae, Procridinae). Steenstrupia (Apollo Books), 192 pp.

[B328] Ehteshamnia N Khaghaninia S Farshbaf Pourabad R (2010) Some Hoverflies of Subfamily Syrphinae of Qurigol fauna in east Azerbayjan province Iran (Diptera: Syrphidae). Munis Entomology & Zoology 5(2): 449–505.

[B329] Eiroa E (1990) Nuevos datos sobre la distribucion de tipulidos en Marruecos. Nouvelle Revue d’Entomologie (N.S.) 7: 259–261.

[B330] Eiroa E (2000) Primera cita de *Dicranoptychafuscescens* (Schummel, 1829) para Marruecos (Diptera: Limoniidae). Boletín de la Asociación española de Entomología 24: 204.

[B331] Elaini R Mazih A (2018) Current status and future prospects of *Ceratitiscapitata* Wiedemann (Diptera: Tephritidae) control in Morocco. Journal of Entomololgy 15(1): 47–55. 10.3923/je.2018.47.55

[B332] Elaini R Mazih A Alonso Valiente M Rahal Y (2019) Domestication and mass rearing of the Mediterranean fruit fly, *Ceratitiscapitata* (Diptera: Tephritidae) from Argan forest. Revue marocaine des Sciences Agronomiques et Vétérinaires 7(1): 88–94.

[B333] El Barmaki S (1993) Étude hydrobiologique de quelques mares temporaires et permanentes de la région de Casablanca. Dynamique et cycles biologiques des espèces culicidiennes. Thèse de Doctorat de 3ème cycle, Université Hassan II, Casablanca, 141 pp.

[B334] El Bouhssini A Amri A Hatchett JH (1988) Wheat genes conditioning resistance to the Hessian fly (Diptera: Cecidomyiidae) in Morocco. Journal of Econonic Entomology 81: 709–712. 10.1093/jee/81.2.709

[B335] El Bouhssini M Amri A Hatchett JH Lhaloui S (1992a) New sources of resistance in wheat to Hessian fly, *Mayetioladestructor* (Say) (Diptera: Cecidomyiidae) in Morocco. Al Awamia 77: 89–107.

[B336] El Bouhssini M Hatchett JH Lhaloui S Amri A (1992b) Suppression of Hessian fly (Diptera: Cecidomyiidae) populations in Morocco by the use of resistant wheat cultivars. Al Awamia 77: 129–145.

[B337] El Bouhssini M Hatchett JH Lhaloui S (1996a) Larval Survival on Wheat Plants carrying Resistance Genes to Hessian fly (Diptera: Cecidomyiidae) in Morocco. Arab Journal for Plant Protection 13(2): 103–105.

[B338] El Bouhssini M Lhaloui S Amri A Jlibene M Hatchett JH Nssarellah N Nachitt M (1996b) Wheat genetic control of Hessian fly (Diptera: Cecidomyiidae) in Morocco. Field Crops Research 45: 111–114. 10.1016/0378-4290(95)00063-1

[B339] El Bouhssini M Lhaloui S Hatchett JH Naber N (1997) Nouveaux gènes de résistance efficaces contre la mouche de Hesse (Diptera: Cecidomyiidae) au Maroc. Al Awamia 96: 55–63.

[B340] El Bouhssini M Benlhabib O Nachit MM Houari A Bentika A Nsarellah N Lhaloui S (1998) Identification in *Aegilops* species of resistant sources to Hessian fly (Diptera: Cecidomyiidae) in Morocco. Genetic Resources and Crop Evolution 45: 343–345. 10.1023/A:1008675029389

[B341] El Bouhssini M Nsarellah N Nachit MM Bentika A Benlhabib O Lhaloui S (1999) First source of resistance in durum wheat to Hessian fly (Diptera: Cecidomyiidae) in Morocco. Genetic Resources and Crop Evolution 46: 107–109. 10.1023/A:1008615515704

[B342] El Haouari H Kettani K (2014) Premier inventaire des Tabanidae (Diptera: Tabanidae) du Rif (Nord du Maroc): Bulletin de l’Institut scientifique, Rabat 36: 77–88.

[B343] El Haouari H Kettani K Ghamizi M (2014) Les Tabanidae (Insecta: Diptera) du Maroc. Bulletin de Société zoologique de France 139(1–4): 91–105.

[B344] El Harym Y Belqat B (2017) First checklist of the fruit flies of Morocco, including new records (Diptera, Tephritidae). ZooKeys 702: 137–171. 10.3897/zookeys.702.13368 PMC5673970 29118602

[B345] El Harym Y Belqat B Korneyev VA (2020) The fruit flies of Morocco: new records of the Tephritinae (Diptera, Tephritidae). Zoodiversity 54(6): 439–452. 10.15407/zoo2020.06.439

[B346] El Harym Y Belqat B Korneyev VA (2021) A new species of *Terellia* (Diptera, Tephritidae) from Morocco. Zoodiversity 55(3): 233–238. 10.15407/zoo2021.03.233

[B347] El Hassani A Messaoudi J (1986) Les ravageurs des cônes et graines de coniferes et leur distribution au Maroc. In: Roques A (Ed.) Proceedings of the 2nd Conference of the Cone and seed insects working party S2.07–01, IUFR (= International Union of Forestry Research Organisations), Briançon, France, September 3–5, 1986: 5–14; Station de Zoologie Forestière, I.N.R.A. (Institut National de la Recherche Agronomique), Ardon, Olivet (France), 5–14.

[B348] El Hawagry MSA (2011) Catalogue of Superfamily Asiloidea (Diptera: Brachycera) of Egypt. Efflatoun’s Journal of Entomology, 195 pp.

[B349] El Hawagry MSA Dhafer H (2015) Five new records of beeflies (Bombyliidae, Diptera) from Saudi Arabia with zoogeographical remarks. ZooKeys 489: 125–133. 10.3897/zookeys.489.8794 PMC4395840 25878533

[B350] El Hawagry MSA El-Azab SA (2019) Catalog of the Calliphoridae, Rhiniidae, and Sarcophagidae of Egypt (Diptera: Oestroidea). Egyptian Journal of Biological Pest Control 29(15): 1–50. 10.1186/s41938-019-0118-8

[B351] El Hawagry MSA Gilbert F (2019) Catalogue of the Syrphidae of Egypt (Diptera). Zootaxa 4577(2): 201–248. 10.11646/zootaxa.4577.2.1 31715720

[B352] El Hawagry M Soliman AM Al Dhafer HM (2021) The family Conopidae (Diptera) in Egypt and Saudi Arabia. Biodiversity Data Journal 9: e60287. 10.3897/BDJ.9.e60287 PMC7817652 33519263

[B353] El Imrani K Kettani K (2012) Dynamique de la structure des communautés benthiques après des perturbations hydrologiques et morphologiques dans l’oued Ez-Zarka (bassin Martil, Rif occidental, Maroc). Méditerranée 118: 7–17. 10.4000/mediterranee.6166

[B354] El Joubari M Louah A Himmi O (2014) Les moustiques (Diptera, Culicidae) des marais de Smir (nord-ouest du Maroc): inventaire et biotypologie. Bulletin de la Société de pathologie exotique 107(1): 48–59. 10.1007/s13149-014-0327-4 24402963

[B355] El Joubari M Faraj C Louah A Himmi O (2015a) Sensibilité des moustiques *Anopheleslabranchiae*, *Culexpipiens*, *Ochlerotatusdetritus* et *Ochlerotatuscaspius* de la région de Smir (Nord-Ouest du Maroc) aux organophosphorés utilisés en santé publique. Environnement, Risques & Santé 14(1): 72–79.

[B356] El Joubari M Hajji K Himmi O El Alami M El Agbani MA Louah A (2015b) Etude des Macroinvertébrés (Gastéropodes, Diptères et Odonates) des marais de Smir-Restinga (Nord-Ouest du Maroc). Entomologie faunistique 68: 17–31.

[B357] El Kaim B (1972) Contribution à l’étude écologique et biologique des Culicides: *Aedesdetritus* Haliday et *Aedescaspius* Pallas. Bulletin de la Société des sciences naturelles et physiques du Maroc 52(3–4): 197–204.

[B358] El Mezdi Z Giudicelli J (1985) Etude d’un écosystème limnique peu connu: Les Khettaras de la région de Marrakech (Maroc), Habitats et Peuplements. Sciences de l’eau 6(3): 281–297.

[B359] El Ouali Lalami A (2012) Surveillance et caractérisation écologique, biologique et génétique des Culicides vecteurs des maladies parasitaires à Fès. Thèse de Doctorat Nationale, Université Sidi Mohammed Ben Abdellah, Fès, 158 pp.

[B360] El Ouali Lalami A Hindi T Azzouzi A Elghadraoui L Maniar S Faraj C Adlaoui E Ameur I Ibnsouda Koraichi S (2010a) Inventaire et répartition saisonnière des Culicidae dans le centre du Maroc. Entomologie faunistique 62(4): 131–138.

[B361] El Ouali Lalami A El Hilali O Benlamlih M Merzouki M Raiss N Ibnsouda Koraichi S Himmi O (2010b) Etude entomologique, physicochimique et bactériologique des gîtes larvaires de localités à risque potentiel pour le paludisme dans la ville de Fès. Bulletin de l’Institut scientifique, Rabat 32(2): 119–127.

[B362] El Ouazzani NH Heller K Kettani K (2019) The first checklist of Black Fungus Gnats (Diptera: Sciaridae) of Morocco. Annales de la Société entomologique de France (N.S.) 55(3): 274–290. 10.1080/00379271.2019.1570826

[B363] El Yousfi M (1994) La santé de cèdre de l’Atlas au Maroc. Annales de la Recherche forestière au Maroc 27: 593–611.

[B364] Engel EO (1938) 25. Bombyliidae. In: Lindner E (Ed.) Die Fliegen der paläarktischen Region 4(3): 619 pp. [+ 15 pls]

[B365] Evenhuis NL (2002) Catalog of the Mythicomyiidae of the World (Insecta: Diptera). Bishop Museum Bulletin in Entomology 10, Bishop Museum Press Honolulu, 85 pp.

[B366] Evenhuis NL (2006) Catalog of Keroplatidae of the World (Insecta: Diptera). Bishop Museum Bulletin in Entomology 13, Bishop Museum Press Honolulu, 178 pp.

[B367] Evenhuis NL (2007) Catalog of the Diptera of the Australasian and Oceanian Regions. (online version). http://hbs.bishopmuseum.org/aocat/mythico.html [accessed: 14 March 2008]

[B368] Evenhuis NL David C (2004) Status of *Cyrtosiamarginata* Perris (Diptera: Mythicomyiidae) with remarks on the type and new distribution records. Zootaxa 731: 1–10.

[B369] Evenhuis NL Greathead DJ (2015) World catalog of bee flies (Diptera: Bombyliidae). Revised September 2015. http://hbs.bishopmuseum.org/bombcat/bombcat.pdf

[B370] Evenhuis NL Kettani K (2018) Genera of Mythicomyiidae (Insecta, Diptera) new to the fauna of Morocco, with descriptions of new species. Zootaxa 4429(2): 348–356. 10.11646/zootaxa.4429.2.9 30313273

[B371] Evenhuis NL Pape T [Eds] (2021) *Systema Dipterorum*, version 3.5. http://diptera.org/ [accessed 20 October 2021]

[B372] Fabricius JC (1787) Mantissa Insectorum sistens species nuper detectas adiectis characteribus, genericis, differentiis, specificis, emendationibus, observationibus. Tome II. Hafniae [= Copenhagen], 382 pp. [The entire work consists of 2 volumes] 10.5962/bhl.title.36471

[B373] Fabricius JC (1794) entomologiae systematicae emendate et aucta 4(6). 472 pp.

[B374] Fabricius JC (1805) Systema antliatorum secundum ordines, genera, species, adiectis synonymis, locis, observationibus, descriptionibus. Carolus Reichard, Brunsvigae [= Brunswick], [xiv + [15]–372 + [4] + 1–30]. 10.5962/bhl.title.15806

[B375] Fain A Athias-Binche F Carton Y (1993) *Histiostomaunidentatum* n. sp. (Acari, Astigmata) associated with a fly *Drosophilabipectinata* originating from Thailand. Bulletin de l’Institut royal des Sciences naturelles de Belgique Entomologie & Biologie 63: 71–76.

[B376] Falcoz L (1924) Dipteres Pupipares du Museum national d’Histoire naturelle de Paris (Streblidae et Nycteribiidae). Bulletin du Muséum d’histoire naturelle, Paris 30: 223–230, 309–315, 386–389. [14 figs]

[B377] Faraj C Daghmoussi F Mouki B El Hayati M Benmansour N Bennouna M Mahjour J (1997) Évaluation de l’efficacité de la lambdacyalothrine, pyrethrinoide de synthèse, dans la lutte contre *Anopheleslabranchiae* en aspersions intradomicilaires. Bulletin épidémiologique hebdomadaire, Ministère de la Santé, Maroc 25: 2–8.

[B378] Faraj C Adlaoui E Rhajaoui M Lyagoubi M (2003) Estimation of Malaria transmission in high-risk provinces of Morocco. Eastern Mediterranean Health Journal 9(4): 542–547. 10.26719/2003.9.4.542 15748051

[B379] Faraj C Adlaoui E Saaf N Romi R Boccolini D Di Luca M Lyagoubi M (2004) Note sur le complexe *Anophelesmaculipennis* au Maroc. Bulletin de la Société de pathologie exotique 97(4): 293–294. 17304755

[B380] Faraj C Elkohli M Lyagoubi M (2006) Cycle gonotrophique de *Culexpipiens* (DipteraCulicidae), vecteur potentiel du virus West Nile, au Maroc: estimation de la durée en laboratoire. Bulletin de la Société de pathologie exotique 99(2): 119–121. 16821445

[B381] Faraj C Adlaoui E Ouahabi S Lakraa E Elkohli M El Aouad R (2007) Extension vers le nord du Maroc de l’aire de distribution d’*Anopheles (Cellia) d’thali* Patton, 1905. Bulletin de la Société de pathologie exotique 101(1): 62–64. 18432013

[B382] Faraj C Adlaoui E Brengues C Fontenille D Lyagoubi M (2008a) Résistance d’*Anopheleslabranchiae* au DDT au Maroc: identification des mécanismes et choix d’un insecticide de remplacement. Eastern Mediterranean Health Journal 14(4): 776–783. 19166159

[B383] Faraj C Ouahabi S Adlaoui E Buccolini D Romi R El Aouad R (2008b) Risque de réémergence du paludisme au Maroc. Etude de la capacité vectorielle d’*Anopheleslabranchiae* dans une zone rizicole au nord du pays. Parasite 15(4): 604–610. 10.1051/parasite/2008154605 19202769

[B384] Faraj C Adlaoui E Elkohli M Herrak T Ameur B Chandre F (2010) Review of Temephos Discriminating Concentration for Monitoring the Susceptibility of *Anopheleslabranchiae* (Falleroni, 1926), Malaria Vector in Morocco. Malaria Research and Treatment, Article ID 126085: 1–5. 10.4061/2010/126085 PMC3276067 22332019

[B385] Faraj C Ameur B Himmi O Sarih M Herrak T Herrak R Ouahabi S Haddaf M Wahabi R Maaroufi A (2018) Enquête entomologique sur les vecteurs des arbovirus: Zika, Dengue et Chikungunya au Maroc. Bulletin d’épidémiologie et de Santé publique 56(76): 38–41.

[B386] Farkas R Hall MJR Bouzagou AK Lhor Y Khallaayoune K (2009) Traumatic myiasis in dogs caused by *Wohlfahrtiamagnifica* and its importance in the epidemiology of wohlfahrtiosis of livestock. Medical and Veterinary Entomology 23(Suppl. 1): 80–85. 10.1111/j.1365-2915.2008.00772.x 19335833

[B387] Faucheux MJ (2009) Signalement de *Nemoteluscylindricornis* Rozkošny, 1977 au Maroc (Diptera: Stratiomyidae: Nemotelinae). Bulletin de la Société des sciences naturelles de l’Ouest de la France 31(3): 144.

[B388] Fekhaoui M Dakki M Aghbani M (1993) Faune benthique d’une rivière polluée: l’oued Sebou à l’aval de la ville de Fès (Maroc). Bulletin de l’Institut scientifique, Rabat 17: 21–38.

[B389] Fendane Y Richet R Thomann T Jourdan M Baker G Ghamizi M Sheppar A (2018) First records of flesh flies (Diptera: Sarcophagidae) emerging from terrestrial snails in Morocco. African Entomology 26(1): 124–130. 10.4001/003.026.0124

[B390] Filali Mouatassem T Faraj C Guemmouh R Rais N El Ouali Lalami A (2019) Quantitative Inventory of Mosquito Larvae (Diptera: Culicidae) and Physicochemical Analysis of Aquatic Habitats in the Region of Fez, Morocco. Bulletin de la Société de pathologie exotique 112(2): 105–113. 10.3166/bspe-2019-0084 31478623

[B391] Fittkau EJ (1962) Die Tanypodinae (Diptera, Chironomidae). Abhandlungen zur Larvalsystematik der Insekten 6: 1–453.

[B392] Fittkau EJ Reiss F (1978) Chironomidae (Diptera). In: Illies J (Ed.) Limnofauna Europaea 2.Aufl. G. Fischer, Stuttgart, 404–440.

[B393] Fleuriet A (1976) Presence of the hereditary *rhabdovirus* sigma and polymorphism for a gene for resistance to this virus in natural populations of *Drosophilamelanogaster*. Evolution 30: 735–739. 10.1111/j.1558-5646.1976.tb00953.x 28563328

[B394] Foote RH (1984) Family Tephritidae. In: Soós Á Papp L (Eds) Catalogue of Palaearctic Dip­tera.Vol. 9, Micropezidae – Agromyzidae. Akademiae Kiado, Budapest & Elsevier Science Publishers, Amsterdam, 66–149. [460 pp]

[B395] Freidberg A (1980) A revision of the genus *Goniurellia* Hendel (Diptera: Tephritidae). Journal of the Entomological Society of southern Africa 43(2): 257–274.

[B396] Freidberg A Kugler J (1989) Fauna Palaestina, Insecta IV Diptera: Tephritidae. The Israel Academy of Sciences and Humanities, Jerusalem, 212 pp.

[B397] Freidberg A Beschovski V (1996) A new species group within *Tethina* Haliday (Diptera: Tethinidae) with descriptions of six new Mediterranean species. Israel Journal of Entomology 30: 91–113.

[B398] Freidberg A Carles-Tolrá M (2010) A new species of *Phlebosotera* Duda (Diptera: Asteiidae) from the Mediterranean, with comments on its biology and a key to *Phlebosotera* species. Studia dipterologica 17(2): 91–102.

[B399] Frey R (1936) Die Dipterenfauna der Kanarischen Inseln und ihre Probleme. Commentationes biologicae 6(1): 1–237.

[B400] Gagné RJ (1995) Revision of tetranychid (Acarina) mite predators of the genus *Feltiella* (Diptera: Cecidomyiidae). Annals of the Entomological Society of America 88: 16–30. 10.1093/aesa/88.1.16

[B401] Gagné RJ (2004) A catalog of the Cecidomyiidae (Diptera) of the world. Memoirs of the Entomological Society of Washington 25: 1–408.

[B402] Gagné RJ (2010) Update for a catalog of the Cecidomyiidae (Diptera) of the world. 545 pp.

[B403] Gagné RJ Hatchett JH Lhaloui S El Bouhssini M (1991) The Hessian fly and the barley stem Gall midge, two different species of *Mayetiola* (Diptera: Cecidomyiidae) in Morocco. Annals of the Entomological Society of America 84(4): 436–443. 10.1093/aesa/84.4.436

[B404] Gagné RJ Jaschhof M (2014) A Catalog of the Cecidomyiidae (Diptera) of the World. 3^rd^ edn. Digital version 2, 493 pp.

[B405] Gaimari SD Mathis WN (2011) World Catalog and Conspectus on the Family Odiniidae (Diptera: Schizophora). Myia 12: 291–339.

[B406] Gatt P (2003) New species and records of *Microphorella* Becker (Diptera: Empidoidea, Dolichopodidae) from the Mediterranean region. Revue suisse de Zoologie 110(4): 669–684. 10.5962/bhl.part.80205

[B407] Gatt P (2006) New records of Sphaeroceridae (Diptera) from the Mediterranean, with distributional notes. Studia dipterologica 13(2): 297–308.

[B408] Gatt P Ebejer MJ (2003) The Ephydridae (Diptera: Brachycera, Muscomorpha) of the Maltese Islands. Studia dipterologica 10(1): 199–214.

[B409] Gatt P Kettani K Ebejer MJ (2016) New records of lesser dung flies (Diptera, Sphaeroceridae) from Morocco. Dipterists Digest 23: 77–82.

[B410] Gaud J (1945a) Présence au Maroc d’Anopheles (Myzomyia) turkhudi Liston, 1901. Archives de l’Institut Pasteur du Maroc 4: 144–147. 21013556

[B411] Gaud J (1945b) Contribution à l’étude des Culicidés au Maroc, quatre espèces nouvelles pour la faune locale. Bulletin de la Société des sciences naturelles du Maroc, Rabat 25–27: 204–206.

[B412] Gaud J (1947a) Phlébotomes du Maroc. Bulletin de la Société des sciences naturelles du Maroc 17: 207–212.

[B413] Gaud J (1947b) Larves d’Anophèles à soies clypéales doublées. Annales de Parasitologie 22: 394–396. 10.1051/parasite/1947225394

[B414] Gaud J (1947c) Lutte antipaludique au Maroc en 1947. Bulletin de l’Institut d’Hygiène du Maroc 7: 119–125.

[B415] Gaud J (1948) Rythmes saisonniers d’activité d’*A.maculipennis* et d’*A.claviger* au Maroc en fonction de l’altitude. Bulletin de la Société de pathologie exotique 12: 494–498.

[B416] Gaud J (1952) Données sur la biocoenose culicidienne de quelques gîtes du Gharb en 1952. Bulletin de l’Institut d’Hygiène, Maroc 12: 55–72.

[B417] Gaud J (1953a) Notes bibliographiques sur les Culicidae du Maroc. Archives de l’Institut Pasteur du Maroc 4: 443–490.

[B418] Gaud J (1953b) Larves d’Anophèles à palettes thoraciques hyperchitinisées. Annales de Parasitologie 28: 326–328. 13125110

[B419] Gaud J (1954) Phlébotomes du Maroc. Bulletin de l’Institut d’Hygiène, Maroc 14(1–2): 91–107.

[B420] Gaud J (1957a) Note de terrain. Comptes Rendus de la Société des sciences naturelles du Maroc 23: 116.

[B421] Gaud J (1957b) Présence au Maroc de *Theobaldiasubochrea* Edwards 1921. Archives de l’Institut Pasteur du Maroc 5: 268–269.

[B422] Gaud J Mechali D Clier JL (1948) Emploi et avenir des insecticides de contact au Maroc dans la prophylaxie des maladies épidémiques. Bulletin de l’Institut d’Hygiène du Maroc 8: 35–90. 10.1051/parasite/1948231035

[B423] Gaud J Faure F Maurice A (1949) Biogéographie des espèces anophéliennes au Maroc. Bulletin de l’Institut d’Hygiène du Maroc, Nouvelle Série 9: 145–163.

[B424] Gaud J Faure F Maurice A (1950) Répartition et fréquence relative des espèces anophiliennes au Maroc. Annales de Parasitologie 25: 53–60. 10.1051/parasite/1950251053

[B425] Gaud J Laurant J (1952) Observations sur les Phlébotomes de la région de Rabat. Bulletin de l’Institut d’Hygiène du Maroc 12(1–2): 75–76.

[B426] Gaud J Duthu P (1954) La variété marocaine d’*Anophelesturkhudi*, ses rapports avec *Anopheleshispaniola*. Bulletin de l’Institut d’Hygiène du Maroc 5: 59–69.

[B427] Geipert S (1993) Entwicklung integrierter Verfahren zur Kontrolle von *Orobanchecrenata* Forsk. in Marokko. Internal report, Supra–regional GTZ–project “Ecology and Management of Parasitic Weeds”, University of Hohenheim, Germany, unpublished.

[B428] Geipert S Kroschel J Sauerborn J (1994) Incidence of *Phytomyzaorobanchia* Kalt. (Diptera: Agromyzidae) in northern Morocco and perspectives for a biological control of *Orobanche* spp. – Results of a field survey. In: Fifth Arab Congress of Plant Protection, 27^th^ November – 2^nd^ December 1994, Fès, Morocco, 235–235.

[B429] Geller-Grimm F (2004) A world catalogue of the genera of the family Asilidae (Diptera). Studia dipterologica 10(2): 473–526.

[B430] Geller-Grimm F Hradsky M (1998) A new species of *Eremonotus* from Morocco, with some remarks about the subfamily Apocleinae in the Palaearctic region (Diptera, Asilidae). [Eine neue *Eremonotus*-Art aus Marokko und einige Bemerkungen über die Unterfamilie Apocleinae in der Paläarktis.]. Studia dipterologica 5(1): 61–66.

[B431] Geller-Grimm F (2015) Asilidae Homepage: Catalog of Species of Asilidae. http://www.geller-grimm.de/catalog/species.htm [accessed September 2015]

[B432] Gerstaecker A (1868) Systematische Uebersicht der bis jetzt bekannt gewordenen Mydaiden (Mydasii Latr.). Stettiner Entomologische Zeitung 29: 65–103. [1 pl.]

[B433] Ghahari H Lehr PA Lavigne RJ Hayat R Ostovan H (2007) New records of robber flies (Diptera, Asilidae) for the Iranian fauna with their prey records. Far Eastern Entomologist 179: 1–9.

[B434] Ghahari H Hayat R Lavigne RJ Ostovan H (2014) An annotated checklist of Iranian Asilidae (Insecta: Diptera: Brachycera: Asiloidea). Linzer biologische Beiträge 46(2): 1379–1446.

[B435] Gheibi M Ostovan H Kamali K (2010) A contribution to the Tachinid flies of the subfamilies Exoristinae and Tachininae (Diptera: Tachinidae) from Fars province, Iran. Turkish Journal of Zoology 34: 35–43.

[B436] Gibbs D (2011) A world revision of the beefly tribe Usiini (Diptera, Bombyliidae). Part 1: UsiasubgenusMicrusia, *U.versicolor* (Fabricius) (= black-haired species) and *Usiamartini* François. Zootaxa 2960: 1–77. 10.11646/zootaxa.2960.1.1

[B437] Gibbs D (2014) A world revision of the beefly tribe Usiini (Diptera, Bombyliidae). Part 2: *Usia* sensu strict. Zootaxa 3799(1): 1–85. 10.11646/zootaxa.3799.1.1 24870868

[B438] Gil Collado J (1929a) Sirefidos de Marruecos del Museo de Madrid (Dipt., Syrphidae). Memorias de la Real Sociedad Española de Historia Natural 12(6): 408–414.

[B439] Gil Collado J (1929b) Cyrtidos españoles y marroquies del museo de Madrid. Memorias de la Real Sociedad Española de Historia Natural 15: 539–552.

[B440] Gil Collado J (1932) Notas sobre pupíparos de España y Marruecos del Museo de Madrid (Dipt., Pupiparos). EOS-Revista Española de Entomología 8: 29–41.

[B441] Gil Collado J (1934) Dos nuevas formas del género *Nemestrellus* Sack de España y Marruecos (Dipt., Nemestrinidae). EOS-Revista Española de Entomología 9: 321–327.

[B442] Gilasian E Talebi AA Ziegler J Manzari S Parchami Araghi M (2013) A review of the genus *Cylindromyia* Meigen (Diptera: Tachinidae) in Iran, with the description of two new species and the newly discovered male of *C.persica* Tschorsnig. Studia dipterologica 20(2): 299–324.

[B443] Gioia Martins-Neto R (2003) The Fossil Tabanids (DipteraTabanidae): When They Began to Appreciate Warm Blood and When They Began Transmit Diseases? Memórias do Instituto Oswaldo Cruz, Rio de Janeiro 98(Suppl. I): 29–34. 10.1590/S0074-02762003000900006 12687759

[B444] Gisondi S Rognes K Badano D Pape T Cerretti P (2020) The world Polleniidae (Diptera, Oestroidea): key to genera and checklist of species. ZooKeys 971: 105–155. 10.3897/zookeys.971.51283 33061774 PMC7538466

[B445] Giudicelli J Lavandier P (1974) Les Blépharocerides de la vallée d’Aure (Hautes Pyrénées, France): Systématique et Ecologie (Dipt., Nematocera). Annals of Limnology 10(3): 245–261. 10.1051/limn/1974004

[B446] Giudicelli J Thiery A (1985) About a peculiar type of rheocrene spring in the High Atlas (Morocco). Description of a simuliid characteristic of this habitat, *Simulium* (*Crenosimulium* n. sp.) *knidirii* n.sp. (Diptera, Simuliidae). Bulletin Zoölogisch museum Universiteit van Amsterdam 10: 109–123.

[B447] Giudicelli J Bouzidi A (1987) Les blepharicerides (diptera) du Maroc. Taxonomie et écologie. Vie et Milieu 37(3–4): 201–206.

[B448] Giudicelli J Bouzidi A (1989) Contribution à l’étude faunistique et écologique des simulies (Diptera, Simuliidae) du Maroc. III. Deux espèces nouvelles du Haut Atlas: Simulium (Simulium) atlasicum n. sp. et Simulium (Simulium) berberum n. sp. Annales de Limnologie 25: 145–158. 10.1051/limn/1989015

[B449] Giudicelli J Bouzidi A Ait Abdelaali N (2000) Contribution à l’étude faunistique et écologique des simulies (Diptera: Simuliidae) du Maroc. IV. Les simulies du Haut Atlas. Description d’une nouvelle espèce. Annales de Limnologie 36: 57–80. 10.1051/limn/2000005

[B450] Gloor H Staiger H (1954) Lethal-polyploid – a polyploid gene in *Drosophilahydei*. Journal of Heredity 45: 289–293. 10.1093/oxfordjournals.jhered.a106497

[B451] González-Duarte R (1976) Estudio de esterases en poblaciones naturals de *Drosophilasubobscura*. Thesis, University of Barcelona, Barcelona, 207 pp.

[B452] González-Duarte R González Izquierdo M Prevosti A (1973) Polymorphism for esterases and alcohol dehydrogenases in natural populations of *Drosophilasubobscura*. Accademia delle Scienze dell’Istituto di Bologna 261 (Ser. iii) 1: 63–70.

[B453] González-Mora D Peris SV (1988) Los Calliphoridae de España: 1: Rhiniinae y Chrysomyinae (Diptera). EOS-Revista Española de Entomología 64: 91–139.

[B454] Götz W (1965) Chromosomaler Polymorphismus in einem Muster von *Drosophilasubobscura* aus Marokko.mit Darstellung der Heterozygotieverhältnisse als Heterozygotiediagramm. Zeitschrift für induktive Abstammungs und Vererbungslehre 97: 40–45. 10.1007/BF00898571 5881947

[B455] Grabener S (2017) Pollination syndromes and networks along an environmental gradient in southern Morocco. Master’s thesis, Universität Hamburg, Germany, 227 pp.

[B456] Greathead DJ (2001) *Exoprosopapandora* (Fabricius, 1805) (Diptera: Bombyliidae) and related species in Europe and the Mediterranean Basin. Insect Systematics & Evolution 32(3): 279–284. 10.1163/187631201X00218

[B457] Gregor F Rozkošný R Barták M Vaňhara J (2002) The Muscidae (Diptera) of Central Europe. Universitatis Masarykianae Brunensis, Biologia 107, 280 pp.

[B458] Grenier P (1953) Simuliidae de France et d’Afrique du Nord (systématique, biologie, importance médicale). Encyclopédie entomologique (A) 29: 1–170.

[B459] Grenier P Théodoridès J (1953) Simulies (Dipt., Simuliidae) du Maroc. Archives de l’Institut Pasteur du Maroc 4: 429–441.

[B460] Grenier P Faure PR (1957) Description d’une simulie nouvelle du Maroc: *Simuliumgaudi* n. sp. (Diptera, Simuliidae). Bulletin de la Société de pathologie exotique 49: 838–840.

[B461] Grenier P Faure PR Laurent J (1957) Simulies (Diptera, Simuliidae) du Maroc (Deuxième mémoire). Archives de l’Institut Pasteur du Maroc 5: 218–242.

[B462] Grenier P Bailly-Choumara H (1970) *Simuliumgracilipes* Edwards, 1921: description de la larve, nymphe, imago mâle et description complémentaire de la femelle. Diagnose du sous-genre *Crosskeyellum* nov. sp. (Diptera: Simuliidae). Cahiers O.R.S.T.O.M. Série Entomologie médicale et Parasitologie 8: 95–105.

[B463] Grichanov IYa (2006) A checklist and keys to North European genera and species of Dolichopodidae (Diptera). All-Russian Institute of Plant Protection RAAS, 120 pp.

[B464] Grichanov IYa (2007) A checklist and keys to Dolichopodidae (Diptera) of the Caucasus and East Meditrranean. Plant Protection News, Supplement, 160 pp.

[B465] Grichanov IYa (2009) New records for Mediterranean Dolichopodidae (Diptera). International Journal of Dipterological Research 20(4): 207–215.

[B466] Grichanov IYa (2012) Review of *Campsicnemus* species from the Atlantic Ocean Islands (Diptera: Dolichopodidae). European Journal of Taxonomy 11: 1–12. 10.5852/ejt.2012.11

[B467] Grichanov IYa (2019) New records of long-legged flies (Diptera, Dolichopodidae) from Morocco. Amurian Zoological Journal vol. XI(2): 126–130. 10.33910/2686-9519-2019-11-2-126-130

[B468] Grichanov IYa (2020) A checklist of species of the family Dolichopodidae (Diptera) of the World arranged by alphabetic list of generic names. http://grichanov.aiq.ru [accessed July 2019]

[B469] Grichanov IYa (2013) Systematic notes on west Palaearctic species of the genus *Syntormon* Loew (Diptera: Dolichopodidae). In: Grichanov IYa Negrobov OP (Eds) Fauna and taxonomy of Dolichopodidae (Diptera).Collection of papers. St. Petersburg: VIZR RAAS, 2013, 3–26. [96 pp] [«Plant Protection News, Supplement»] 10.5852/ejt.2013.61

[B470] Grichanov IYa Negrobov OP (2014) Palaearctic species of the genus *Sciapus* Zeller (Diptera: Dolichopodidae). St. Petersburg: VIZR, 84 pp. [«Plant Protection News Supplements», N13]

[B471] Grichanov IYa Vikhrev N (2009) Mediterranean species of the *Medeteraplumbella* species group with description of a new peculiar species from Morocco (Diptera: Dolichopodidae). Zootaxa 2170: 46–52. 10.11646/zootaxa.2170.1.5

[B472] Grichanov IYa Nourti M Kettani K (2020a) The *Hercostomusexarticulatus* species group in the Palaearctic Region (Diptera: Dolichopodidae). Caucasian Entomological Bulletin 16(1): 27–34. 10.23885/181433262020161-2734

[B473] Grichanov IYa Nourti M Kettani K (2020b) A new species of *Chrysotus* Loew, 1857 (Dolichopodidae, Diptera) from Morocco. Acta Zoologica Bulgarica 72(3): 333–338.

[B474] Grichanov IYa Nourti M (2021) Notes on taxonomy and distribution of some *Ortochile* Latreille, 1809, *Sybistroma* Meigen, 1824 and *Teuchophorus* Loew, 1857 (Diptera: Dolichopodidae) species from Mediterranean Region. Russian Entomological Journal 30(2): 189–195. 10.15298/rusentj.30.2.14

[B475] Griffiths GCD (1967) Revision of the *Phytomyzasyngenesiae* group (Diptera, Agromyzidae), including species hitherto known as “*Phytomyzaatricornis* Meigen”. Stuttgarter Beiträge zur Naturkunde 177: 1–28.

[B476] Griffiths GCD (1974) Studies on Boreal Agromyzidae (Diptera). V. On the genus *Chromatomyia* Hardy, with revision of Caprifoliaceae-mining species. Quaestiones entomologicae 35(1): 35–69.

[B477] Griffiths GCD (1980) Studies on Boreal Agromyzidae (Diptera). XIV. *Chromatomyia* miners on Monocotyledones. Entomologica scandinavica Supplement 13: 1–61.

[B478] Grootaert P Chvála M (1992) Monograph of the genus *Platypalpus* (Diptera: Empidoidea, Hybotidae) of the Mediterranean region and the Canary Islands. Acta Universitatis Carolinae. Biologica 36: 3–226.

[B479] Grootaert P Zouhair L Kettani K (2021) New species of the genus *Stilpon* Loew, 1859 from Morocco (Diptera: Empidoidea, Hybotidae). Belgian Journal of Entomology 113: 1–12.

[B480] Guernaoui S Pesson B Boumezzough A Pichon G (2005) Distribution of Phlebotomine Sandflies, of the subgenus Larroussius, in Morocco. Medical and Veterinary Entomology 19: 111–115. 10.1111/j.0269-283X.2004.00548.x 15752186

[B481] Guernaoui S Boumezzough A (2009) Habitat Preferences of Phlebotomine Sand Flies (Diptera: Psychodidae) in Southwestern Morocco. Journal of Medical Entomology 46(5): 1187–1194. 10.1603/033.046.0529 19769053

[B482] Guy Y (1958) Extension vers le Nord de l’aire d’Anopheles (Anopheles) coustani Laveran, 1900. Bulletin de la Société des sciences naturelles et physiques du Maroc 38: 207–212.

[B483] Guy Y (1959a) Les rapports entre l’anophélisme et le paludisme. Bulletin de la Société des sciences naturelles et physiques du Maroc 39(2): 83–90.

[B484] Guy Y (1959b) Les Anophèles du Maroc. Mémoires de la Société des sciences naturelles et physiques du Maroc, Nouvelle série, Rabat 7: 1–235.

[B485] Guy Y (1961) A propos d’une larve monstrueuse d’Anophèle. Annales de Parasitologie 36: 788–796. 10.1051/parasite/1961365788 13903223

[B486] Guy Y (1962) Renseignements fournis par l’étude du rapport larves–adultes d’Anophèles. Annales de Parasitologie 37: 633–643. 10.1051/parasite/1962374633 13951502

[B487] Guy Y (1963) Bilan épidémiologique du paludisme au Maroc (données recueillies entre 1960, 1961 et 1962). Annales de Parasitologie Humaine et Comparée 38: 823–857. 10.1051/parasite/1963385823 14122070

[B488] Guy Y (1967) Une hypothèse au sujet des larves monstrueuses dans le genre «*Anopheles*». Archives de l’Institut Pasteur d’Algérie 45: 51–61.

[B489] Guy Y Dupuy R (1958) Importance d’Anopheles (Myzomyia) sergenti Theobald, 1907 au Maroc. Comptes Rendus de la Société des sciences naturelles et physiques du Maroc 24(8): 194.

[B490] Guy Y Holstein M (1968) Données récentes sur les Anophèles du Maghreb. Archives de l’Institut Pasteur d’Algérie 46: 142–150.

[B491] Hackman W (1960) Coelopidae, Drosophilidae, Sphaeroceridae and Scatophagidae (Diptera, Ciclorrapha) from Azores and Madeira. Boletim do Museu Municipal do Funchal (História Natural) № XIII, Art 38.

[B492] Hackman W (1970) Trixoscelidae (Diptera) from Southern Spain and Description of a New *Trixoscelis* species from Northern Europe. Entomologica scandinavica 1: 127–134. 10.1163/187631270X00168

[B493] Hadji M Belghiti D Elomari F El Assal M El Marsini M (2013) Étude de la dynamique stationnelle des populations des culicidés dans la province de Sidi Slimane (Maroc). Afrique Science 9(1): 128–139.

[B494] Hadji M Belghiti D El Assal M El Marsini M Laaroussi T (2014) Cartographie de la faune culicidienne dans la province de Sidi Slimane (Maroc). World Journal of Biological Research 6(1): 7–13.

[B495] Haenni JP (1981) North African *Dilophus* Meigen, with description of *D.maghrebensis* n. sp. (Diptera: Bibionidae). Entomologica scandinavica 12: 429–432. 10.1163/187631281X00490

[B496] Haenni JP (1990) Note sur la présence en Europe de *Chorisopstunisiae* (Beck.) (Diptera, Stratiomyidae). Bulletin de la Société neuchâteloise des sciences naturelles 113: 285–288.

[B497] Haenni JP (2009) The Bibionidae (Diptera) of Sardinia, with description of two new species. Zootaxa 2318: 427–439. 10.11646/zootaxa.2318.1.17

[B498] Haenni JP Kettani K (2011) Première note sur les Scatopsidae du Maroc, avec la description d’une espèce nouvelle (Diptera). Bulletin de la Société entomologique de France 116(1): 73–79. 10.3406/bsef.2011.2907

[B499] Haenni JP Kettani K (2016) Note faunistique sur les Scatopsidae du Rif (nord du Maroc) (Diptera). Bulletin de la Société entomologique de France 121(4): 517–520. 10.3406/bsef.2016.2789

[B500] Hafraoui A (1966) Premières constatations de dégâts causés par une Cécidomyie sur l’olivier *Climodiplosiscleisuga* Targ (Dipt., Cecidomyiidae). Al Awamia 20: 139–141.

[B501] Hall MJR Testa JM Smith L Adams ZJO Khallaayoune K Sotiraki S Stefanakis A Farkas R Ready PD (2009) Molecular genetic analysis of populations of Wohlfahrt’s wound myiasis fly, *Wohlfahrtiamagnifica*, in outbreak populations from Greece and Morocco. Medical and Veterinary Entomology 23(Suppl. 1): 72–79. 10.1111/j.1365-2915.2009.00780.x 19335832

[B502] Hanafi A Schnitzler WH (2004) Integrated production and protection in greenhouse tomato in Morocco. ISHS. (International Society for Horticultural Science): Acta Agriculturae 659: 323–330. 10.17660/ActaHortic.2004.659.38

[B503] Handaq N (1998) Les moustiques du Maroc: écologie et biogéographie des peuplements culicidiens dans les régions montagneuses, semi arides et arides du Maroc occidental: Essai de biotypologie des gîtes larvaires et étude comparative de la dynamique des populations marocaines et tunisiennes. Thèse de Doctorat de 3ème cycle, Université Cadi Ayad, Marrakech, 189 pp.

[B504] Handaq N Boumezzough A (1999) *Aedescaspius* dans le sud-ouest marocain: impact de l’aridité sur la dynamique des populations pré imaginales (Diptera, Culicidae). Bulletin de la Société entomologique de France 104(2): 183–191. 10.3406/bsef.1999.16567

[B505] Handaq N Boumezzough A (2002) Confirmation de la présence au Maroc d’*Aedesaegypti* Linné, 1762, en zone continentale dans la région de Marrakech (Diptera, Culicidae). Bulletin de la Société entomologique de France 107(2): 200–200. 10.3406/bsef.2002.16840

[B506] Harris KM (1968) A systematic revision and biological review of the cecidomyiid predators (Diptera, Cecidomyiidae) on world Coccoidea. Transactions of the Royal Entomological Society of London 119: 409–494. 10.1111/j.1365-2311.1968.tb00504.x

[B507] Harris EJ Hafraoui A Toulouti B (1980) Mortality of Nontarget Insects by Poison Bait Applied to Control the Mediterranean Fruit Fly, *Ceratitiscapitata* (Diptera: Tephritidae), in Morocco. Proceedings, Hawaiian Entomological Society Vol. 23, No. 2.

[B508] Hauser M Kassebeer CF (1998) *Neoasciaclausseni* spec. nov. aus Nordafrika (Diptera, Syrphidae). Dipteron 1(2): 37–44.

[B509] Hauser M Winterton SL Kirk-Sprigges AH Holsten KC (2017) 49. Therevidae (Stiletto Flies). In: Kirk-Spriggs AH Sinclair BJ (Eds) Manual of Afrotropical Diptera.Volume 2. Nematocerous Diptera and lower Brachycera. Suricata, Volume 5. SANBI Graphics & Editing, Pretoria, 1183–1220.

[B510] Hayat R Ghahari H Lavigne R Ostovan H (2008) Iranian Asilidae (Insecta: Diptera). Turkish Journal of Zoology 32: 175–195.

[B511] HCEFLCD [Haut-Commissariat aux Eaux et Forêts et à la Lutte Contre la Desertification] (2017) Plan d’Aménagement et de Gestion du Parc National de Toubkal. Mission I – Diagnostic : analyse du périmetre d’intervention et du territoire environnant. Mai 2017, 305 pp.

[B512] Hendel FG (1913) Die Gattung *Platystoma* Meigen (Dipt.). Eine monographische Übersicht über die Arten. Mit Taf. 1–2. Zoologische Jahrbücher (Abteilung für Systematik) 35: 55–126. [+ 3] [2 tables]

[B513] Hendel FG (1927) 49. Trypetidae. In: Lindner E (Ed.) Die Fliegen der palaearktischen Region 5, 221 pp.

[B514] Hendel FG (1931–36) 59. Agromyzidae. In: Lindner E (Ed.) Die Fliegen der palaearktischen Region 6(2): xi + 570 pp., pls i–xvi + 16 pp.

[B515] Hennig W (1945) 48. Platystomatidae. E. Lindner (Herausgeber). Die Fliegen der palaearktischen Region 5(155): 1–56. [+ 3 tables]

[B516] Hérard F Fraval A (1980) La répartition et les ennemis naturels de *Lymantriadispar* (L.) (Lép.: Lymantriidae) au Maroc, 1973–1975. Acta oecologica. Oecologia applicata 1: 35–48.

[B517] Herman P Dirlbek J (2006) Contribution to the knowledge of the fruit fly fauna of Algeria, Morocco and Tunisia (Diptera: Tephritidae). Acta Universitatis Carolinae. Biologica 50: 53–56.

[B518] Hernández-Moreno S Peris SV (1989) *Ophyraaenescens* (Wiedemann, 1830), aclimatada en la Península Ibérica, con notas sobre algunas otras especies del grupo (Diptera, Muscidae). Boletín de la Real Sociedad Española de Historia Natural (Sección Biológica) 84(3–4): 207–217.

[B519] Herting B (1979) Beschreibungen neuer Raupenfliegen (Dipt., Tachinidae) und Revision der *Besseriaanthophila*-Gruppe. Stuttgarter Beiträge zur Naturkunde Serie A (Biologie) 323: 1–10.

[B520] Herting B Dely-Draskovits A (1993) Tachinidae. In: Soós Á Papp L (Eds) Catalogue of Palaearctic Diptera Vol.13. Hungarian Academy of Science, Budapest, 118–624. [624 pp]

[B521] Hervy JP Garulli F Brunhes J Geoffroy B (1994) Les entomologistes médicaux de l’ORSTOM et la diversité du vivant. Un demi-siècle de descriptions d’espèces nouvelles ORSTOM éditions, 76 pp.

[B522] Himmi O (1991) Culicidae (Diptera) du Maroc: Clé de détermination actualisée et étude de la dynamique et des cycles biologiques de quelques populations de la région de Rabat – Kénitra. Thèse de doctorat de 3ème cycle, Faculté des Sciences, Rabat, 185 pp.

[B523] Himmi O (2007) Les culicides (Insectes, Diptères) au Maroc: Systématique, écologique et études épidémiologiques pilotes. Thèse d’Etat. Université Mohammed V, Agdal, Faculté des Sciences, 363 pp.

[B524] Himmi O Bayed A El Agbani MA Dakki M (1994) Critère d’âge et détermination des stades larvaires chez les Culicides (Diptera) du Maroc. Actes de la VIème CILEF, Hydroécologie appliquée, Publication particulière 1: 61–84.

[B525] Himmi O Dakki M Trari B El Agbani MA (1995) Les Culicidae du Maroc: Clés d’identification, avec données biologiques et écologiques. Travaux de l’Institut Scientifique. Série Zoologie, Rabat 44: 1–50.

[B526] Himmi O El Agbani MA Trari B Dakki M (1998) Contribution à la connaissance de la cinétique et des cycles biologiques des Moustiques (Diptera, Culicidae) dans la région de Rabat – Kénitra (Maroc). Bulletin de l’Institut scientifique, Rabat 21: 71–79.

[B527] Hippa H Vilkamaa P Heller K (2010) Review of the Holarctic *Corynoptera* Winnertz, 1867, s. str. (Diptera, Sciaridae). Zootaxa 2695: 1–197. 10.11646/zootaxa.2695.1.1 26106694

[B528] Houard C (1912) Les Zoocécidies du Nord de l’Afrique. Annales de la Société entomologique de France 81: pls 1–2, 1–236.

[B529] Houard C (1917) Cécidies Nord-Africaines. Troisiéme Contribution. Marcellia 15(1916): 121–132.

[B530] Houard C (1919) Les Collections cécidologiques du Laboratoire d´Entomologie du Muséum d´Histoire naturelle de Paris: Galles de l´Ancien Continent, extra-européennes. Marcellia 16(1917): 79–100.

[B531] Houard C (1921) Les Collections cécidologiques du Laboratoire d´Entomologie du Muséum d´Histoire naturelle de Paris: Galles du Nord de l’Afrique. Marcellia 17(1918): 114–135.

[B532] Houard C (1922) Les Collections cécidologiques du Laboratoire d´Entomologie du Muséum d´Histoire naturelle de Paris: Cécidies récoltées au Maroc par C-J. Pitard. Marcellia 19(1920): 86–116.

[B533] Houard C (1922–1923) Les Zoocécidies des Plantes d’Afrique, d’Asie et d’Océanie. Vols 1+2. Jules Hermann, Paris, 1–498, 1 pl., I–V, 501–1058.

[B534] Houard C (1923) Les Collections cécidologiques du Laboratoire d´Entomologie du Muséum d´Histoire naturelle de Paris: Galles du Maroc et de l´Algérie. Marcellia 20(1921–1923): 122–162.

[B535] Howard V Weems Jr Sanford MT (2000) Bee louse, *Braulacoeca* Nitzsch (Insecta: Diptera: Braulidae). EENY–171 (IN328) (originally published as DPI Entomology Circular 252). Featured Creatures series of the Entomology and Nematology Department, Cooperative Extension Service, Institute of Food and Agricultural Sciences, University of Florida.

[B536] Hradský M Hüttinger E (1995) Übersicht übert die Arten der Gatung *Eriopogon* Loew, 1847, mit Beschreibung von *E.spatenkai* spec. nov. aus Nordafrika (Insecta: Diptera: Asilidae). Reichenbachia 31(20): 103–106.

[B537] Hradský M Geller-Grimm F (1997) Eine neue Art aus der Gattung *Stiphrolamyra* (Diptera, Asilidae, Laphriinae). Studia dipterologica 4(1): 193–196.

[B538] Hradský M Geller-Grimm F (2005) The *appendiculatus* species-group of *Habropogon* Loew, 1847 (Diptera: Asilidae) in the Palaearctic Region, including the description of new species. Zootaxa 1004: 1–14. 10.11646/zootaxa.1004.1.1

[B539] Hudault E Zelensky V (1939) Contribution à la connaissance de la biologie de la Cécidomyie destructive (*Mayetioladestructor* Say) au Maroc. Revue de Pathologie végétale et d’Entomologie agricole de France 26(1): 93–100.

[B540] Hurkmans W (1993) A monograph of *Merodon* (Diptera: Syrphidae) Part 1. Tijdschrift voor Entomologie 136: 147–234.

[B541] Ibn Jilali S (1988) Les Diptères Drosophilidae du Maroc: Morphologie, Ecologie et Demographie d’une espèce de drosophile phytophage *Scaptomyzapallida* Zetterstedt 1847. Thèse, University Mohamed V, Rabat, Maroc, 157 pp.

[B542] IOBC-List 1 [The International Organization for Biological Control] (1956) Liste d’identification no 1. Entomophaga 1: 113–127. 10.1007/BF02377895

[B543] IOBC-List 11 [The International Organization for Biological Control] (1989) Bestimmungsliste entomophager Insekten 11. IOBC/WPRS Bulletin 12(7): 63 pp.

[B544] IOBC-List 12 [The International Organization for Biological Control] (1993) Determination list of entomophagous insects Nr. 12. IOBC/WPRS Bulletin 16(3): 56 pp.

[B545] IOBC-List 13 [The International Organization for Biological Control] (1997) Determination list of entomophagous insects Nr. 13. IOBC/WPRS Bulletin 20(2): 53 pp.

[B546] IOBC-List 14 [The International Organization for Biological Control] (2005) Determination list of entomophagous insects Nr. 14. IOBC/WPRS Bulletin 28(11): [VII +] 71 pp.

[B547] Jaschhof M (1994) Eine neue *Lestremia*-Art aus Marokko (Cecidomyiidae, Lestremiinae). [A new *Lestremia* species from Morocco (Cecidomyiidae, Lestremiinae)]. Studia dipterologica 1(1): 122–125.

[B548] Jaschhof M (1998) Revision der “Lestremiinae” (Diptera, Cecidomyiidae) der Holarktis. Studia dipterologica, Supplement 4, 552 pp.

[B549] Jaschhof M Jaschhof C (2009) The Wood Midges (Diptera: Cecidomyiidae: Lestremiinae) of Fennoscandia and Denmark. Studia dipterologica, Supplement 18: 333 pp. [+ appendix i–viii]

[B550] Ježek J (1999) Rare and new Palaearctic *Tonnoiriella*-species (Diptera, Psychodidae). Časopis Národního muzea, Řada přírodovědná 168: 7–18.

[B551] Ježek J (2004) New faunistic data of non-phlebotomine moth flies (Diptera, Psychodidae) from the Palaearctic region. In: Kubik Š Barták M (Eds) Dipterologica bohemoslovaca Vol.11. Folia facultatis scientiarum naturalium, Universitatis Masarykianae Brunensis, Biologia, 141–151. [109: 346 pp]

[B552] Ježek J Yağci Ş (2005) Common Non-biting Moth Flies (Insecta, Diptera, Psychodidae) New to the Fauna of Turkey. Acta parasitologica Turcica 29(3): 188–192. 17160821

[B553] Ježek J Hájek J (2007) Psychodidae (Diptera) of the Orlické hory Protected Landscape Area and neighbouring areas with descriptions of two new species from the Czech Republic. Acta Entomologica Musei Nationalis Pragae 47: 237–285.

[B554] Ježek J Barták M Vanek J Kubík Š (2012) Ovádovití (Diptera, Tabanidae) Ceské Cásti Krkonoš. Opera Corcontica 49: 173–180.

[B555] Jourdan L (1935a) *Clytiomyiahelluo* F. parasite d’*Eurygasteraustriaca* Schr. (Hem. Pent.). Revue d’Entomologie France 2(1): 83–88.

[B556] Jourdan L (1935b) Notes sur deux mouches parasites *Clytiomyia* et *Gymnosoma* (Larvaevoridae). Encyclopédie entomologique B. II. (Diptera) 8: 117–119.

[B557] Jourdan L (1935c) Observations biologiques sur les Macrolépidoptères du Maroc. Revue de Pathologie végétale et d’Entomologie agricole de France 22: 132–167.

[B558] Jourdan L (1937) Observations sur la biologie de la Cécidomyie destructive (*Mayetioladestructor* Say) au Maroc. (l^ère^ note). Bulletin de la Société des sciences naturelles du Maroc 17: 154–162.

[B559] Jourdan L (1938) Une nouvelle mouche parasite des blés au Maroc, *Hylemyiasepia* Meigen (Diptère Anthomyiidae). Bulletin de la Société des sciences naturelles du Maroc 18: 25–26.

[B560] Jousset FX (1976) Etude expérimentale du spectre d’hôtes du virus C de *Drosophilamelanogaster* chez quelques Diptères et Lepidoptères. Annals of Microbiology 127(Ser. A): 529–544. 823856

[B561] Jousset FX Plus N (1975) Etude de la transmission horizontale et de la transmission verticale des Picornavirus de *Drosophilamelanogaster* et de *Drosophilaimmigrans*. Annals of Microbiology Ser. B, 126: 231–249. 814853

[B562] Kameneva EP (2007) A new species of *Herina* (Diptera, Ulidiidae) from Switzerland, with a key to the European species and notes on nomenclature and distribution. Vestnik zoologii 41(5): 405–421.

[B563] Kameneva EP Korneyev V (2016) Revision of the Genus *Physiphora* Fallén, 1810 (Diptera: Ulidiidae: Ulidiinae). Zootaxa 4087(1): 001–088. 10.11646/zootaxa.4087.1.1 27394323

[B564] Kanervo E (1939) Inventa entomologica itineris Hispanici et Maroccani, quod. 1926 fecerunt Harald et Hakam Lindberg. XXIV Diptera. Commentationes biologicae 7(8): 1–6.

[B565] Karimpour Y (2012) On the beefly (Diptera: Bombyliidae) fauna from West Azarbaijan Province of Iran. Biharean Biologist, Oradea, Romania 6(2): 81–86.

[B566] Kassebeer CF (1995) *Pipizellathapsiana* n. sp. aus dem Hohen Atlas (Diptera: Syrphidae) Beiträge zur Schwebfliegenfauna Marokkos, I. Entomologische Zeitschrift 105: 260–264.

[B567] Kassebeer CF (1996) Eine neue Art der Gattung *Beris* Latreille, 1802 aus Marokko (Diptera, Stratiomyidae). Studia dipterologica 3(1): 155–159.

[B568] Kassebeer CF (1998a) Ergänzende Nachweise von Schwebfliegen aus Marokko (Diptera: Syrphidae). Beiträge zur Schwebfliegenfauna Marokkos III. Dipteron (Kiel) 1(1): 21–24.

[B569] Kassebeer CF (1998b) Die marokkanischen Arten der Gattung *Platycheirus* Le Peletier & Serville, 1828 (Diptera, Syrphidae); Beiträge zur Schwebfliegenfauna Marokkos V. Dipteron (Kiel) 1(2): 25–36.

[B570] Kassebeer CF (1998c) Die marokkanischen Arten der Gattung *Cheilosia* Meigen, 1822 (Diptera, Syrphidae). Beiträge zur Schwebfliegenfauna Marokkos VII. Dipteron (Kiel) 1(3): 57–68.

[B571] Kassebeer CF (1999a) Neue Nachweise von Netzfliegen (Diptera, Sciomyzidae) aus Marokko, mit einer Übersicht der Fauna des Maghreb. Dipteron (Kiel) 2(1): 1–10.

[B572] Kassebeer CF (1999b) Eine neue Art der Gattung *Ferdinandea* Rondani, 1844 (Diptera, Syrphidae) aus Nordafrika. Beiträge zur Schwebfliegenfauna Marokkos IX. Dipteron (Kiel) 2(8): 153–162.

[B573] Kassebeer CF (1999c) Eine neue Gattung der Brachyopini (Diptera, Syrphidae) aus dem Mittleren Atlas. Beiträge zur Schwebfliegenfauna Marokkos VIII. Dipteron (Kiel) 2(1): 11–24.

[B574] Kassebeer CF (1999d) Zur Kenntnis einiger Gattungen der Chrysogasterini in Marokko (Diptera: Syrphidae). Beiträge zur Schwebfliegenfauna Marokkos, IV. Entomologische Zeitschrift 109(4): 155–164.

[B575] Kassebeer CF (2000) Eine neue *Brachyopa* Meigen, 1822 (Diptera, Syrphidae) aus dem Atlas Beiträge zur Schwebfliegenfauna Marokkos X. Dipteron (Kiel) 3(2): 141–148.

[B576] Kazerani F Khaghaninia S Grichanov IYa (2013a) The genus *Rhaphium* Meigen (Diptera: Dolichopodidae) in Iran, with new species records for the country. Studia dipterologica 20(1): 113–119.

[B577] Kazerani F Talebi AA Gilasian E (2013b) An annotated checklist of the subfamily Syrphinae (Diptera: Syrphidae) of Iran. Entomofauna 34(34): 517–556.

[B578] Kazerani F Khaghaninia S Talebi AA Grichanov IYa (2014) Faunistic survey of Dolichopodidae in forests of northern Iran with nine species as new records for the country. Zoology and Ecology 24(3): 266–273. 10.1080/21658005.2014.937926

[B579] Kechev M (2012) Long-Legged Flies (Diptera: Dolichopodidae) from the Upper Thracian Plain New to Bulgaria: Habitats and Distribution. Acta Zoologica Bulgarica 64(2): 205–208.

[B580] Kechev M (2017) New records of long-legged flies (Diptera: Dolichopodidae) from Greece. Silva Balcanica 18(1): 53–57.

[B581] Kechev M Ivanova M (2015) New records of Dolichopodidae (Diptera) for Bulgaria. Comparison of Dolichopodid diversity on river banks and in some forests of the upper Thracian plain. Silva Balcanica 16(1): 87–104.

[B582] Kehlmaier C (2004) Faunistic and taxonomic notes of Anisopodidae, Acroceridae, Conopidae and Stratiomyidae (Diptera) collected on the Iberian Peninsula. Faunistische Abhandlungen 25: 125–137.

[B583] Kehlmaier C (2005) Taxonomic revision of European Eudorylini (Insecta, Diptera, Pipunculidae). Verhandlungen des naturwissenschaftlichen Vereins in Hamburg (NF) 41: 345–353.

[B584] Kehlmaier C (2014) A new *Lampromyia* Macquart from Europe (Diptera: Vermileonidae). Zootaxa 3887(3): 481–493. 10.11646/zootaxa.3887.4.6 25543945

[B585] Kelsey LP (1969) A revision of the Scenopinidae (Diptera) of the world. United States National Museum, Bulletin 277: [i–v +] 1–336. 10.5962/bhl.title.16405

[B586] Kelsey LP Soós Á (1989) Family Scenopinidae. In: Soós Á Papp L (Eds) Catalogue of Palaearct­ic Diptera.6. Therevidae–Empididae. Elsevier, Amsterdam & Akadémiai Kiadó, Buda­pest, 35–43. [435 pp]

[B587] Kerr PH (2004) Revision of the genera of the Rhagionidae of the world (Diptera: Brachycera). Dissertation submitted to the Faculty of the Graduate School of the University of Maryland, College Park in partial fulfillment of the requirements for the degree of Doctor of Philosophy, 635 pp.

[B588] Kettani K (1994) Etude faunistique et écologique des Chironomidés (Diptera) de l’Oued Laou et de l’Oued Martil. Thèse de troisième cycle, Université Abdelmalek Essaadi, Tétouan, 280 pp.

[B589] Kettani K (1998) Etude Hydrobiologique de l’Oued Laou et de l’Oued Martil (Rif – Maroc): Peuplements chironomidiens (Diptera, Nematocera) et distribution longitudinale. Thèse d’Etat Es-Sciences, Université Abdelmalek Essaadi, Tétouan, 250 pp.

[B590] Kettani K Vilchez A Calle D El Ouazzani T (1994) Les Chironomidés (Diptera) du bassin de l’Oued Laou (Versant méditerranéen du Rif, Maroc). Annales de Limnologie 30(1): 25–32. 10.1051/limn/1994002

[B591] Kettani K Vilchez A Calle D El Ouazzani T (1995) Nouvelles récoltes de Chironomidés (Diptera) du Maroc: Les Chironomidés de l’Oued Martil (Rif). Annales de Limnologie 31(1): 253–261. 10.1051/limn/1995023

[B592] Kettani K Calle D El Ouazzani T (1996) Données faunistiques actuelles sur les Chironomidés (Diptera) du Rif. Bulletin de l’Institut scientifique, Rabat 20: 131–141.

[B593] Kettani K Calle D El Ouazzani T (1997) Nuevas especies de Quironomidos (Insecta – Diptera) en el alto Laou (Rif, norte de Marruecos). Zoologica baetica 8: 181–189.

[B594] Kettani K Calle D El Ouazzani T (2001) Mise à jour de l’inventaire des Chironomidés (Diptera) connus du Maroc. Annales de Limnologie 37(4): 323–333. 10.1051/limn/2001027

[B595] Kettani K Belqat B El Haouari H (2010) Les Chironomidés (Diptera) du Parc National de Talassemtane. Actes CIFE VI. Travaux de l’Institut Scientifique, Rabat. Série Zoologie 47(1): 67–72.

[B596] Kettani K Langton P (2011) New data on the Chironomidae (Diptera) of the Rif (Northern Morocco). Polish Journal of Entomology 80: 583–595. 10.2478/v10200-011-0046-8

[B597] Kettani K Langton P (2012) Les Chironomidae du Maroc (Diptera, Nematocera). Bulletin de la Société entomologique de France 117(4): 411–424. 10.3406/bsef.2012.3065

[B598] Kettani K Negrobov O (2016) The update check list of Dolichopodidae of Morocco (Diptera). Centre for Entomological Studies Ankara 128: 1–9.

[B599] Kettani K Moubayed-Breil J (2018) Communities of Chironomidae from four ecological zones delimited by the Mediterranean coastal ecosystem of Morocco (Moroccan Rif). Updated list and faunal data since the last two decades. Journal of Limnology 77(s1): 141–144. 10.4081/jlimnol.2018.1727

[B600] Khaganinia S Kazerani F (2014) A review of the genus *Cheilosia* (Diptera, Syrphidae) from Iran. Vestnik zoologii 48(5): 401–410. 10.2478/vzoo-2014-0048

[B601] Khaghaninia S Zarghani E Gharajedaghi Y (2014) A recent contribution to the black scavenger flies (Diptera: Sepsidae) in Iran. Biharean Biologist 8(1): 21–23.

[B602] Khalifi L El Bouhssini M Amri A Lhaloui S (1996) Effet de la Température sur l’expression de la Résistance à la mouche de Hesse (Diptère: Cecidomyiidae) chez le blé tendre (*Triticumaestivum* L.). Al Awamia 92: 33–42.

[B603] Kiliç AY (1999) Checklist of Tabanidae (Diptera) From Turkey. Turkish Journal of Zoology 23: 123–132.

[B604] Kirk-Spriggs AH Mc Gregor G (2009) Disjunctions in the Diptera (Insecta) fauna of the Mediterranean Province and southern Africa and a discussion of biogeographical considerations. Transactions of the Royal Society of South Africa 64(1): 32–52. 10.1080/00359190909519236

[B605] Klein O (1995) Untersuchungen zur Populationsdynamik und zur Verwendung von *Phytomyzaorobanchia* in der biologischen Schädlingsbekämpfung von *Orobanche* spp. in Marokko. – Diplomarbeit, Universität Hohenheim, Institut für Pflanzenproduktion in den Tropen und Subtropen 1 + II + 99 + VIII pp. + 1 Bildtafel [Studies on the population dynamics and the use of *Phytomyzaorobanchia* for the biological control of *Orobanche* spp. in Morokko].

[B606] Klein O Kroschel J Sauerborn J (1995) *Phytomyzaorobanchia* Kalt. (Diptera: Agromyzidae) in Morocco and its potential use for biological control of *Orobanche* spp. – XIII International Plant Protection Congress, The Hague, The Netherlands, 2–7 July 1995. European Journal of Plant Pathology, Abstracts no. 528.

[B607] Klein O Kroschel J Sauerborn J (1999) Efficacité de lâchers périodiques de *Phytomyzaorobanchia* Kalt. (Diptera: Agromyzidae) pour la lutte biologique contre l’*Orobanche* au Maroc. In: Kroschel J, Betz H, Abderahibi M (Eds) Advances in Parasitic Weed Control at On-farm Level. Vol. II. Joint Action to control Orobanche in the WANA region. Margraf Verlag, Weikersheim, Germany, IX + 347 pp.

[B608] Klein O Kroschel J (2002) Biological control of *Orobanche* spp. with *Phytomyzaorobanchia*, a review. BioControl 47(3): 245–277. 10.1023/A:1014862302818

[B609] Knight KL (1971) Comparative anatomy of the mandible of the fourth instar mosquito larva (Diptera, Culicidae). Journal of Medical Entomology 8: 189–205. 10.1093/jmedent/8.2.189 4400542

[B610] Knutson LV (1981) New combinations and synonymies in Palaearctic and Nearctic Sciomyzidae (Diptera). Proceedings of the entomological Society of Washington 83: 332–338.

[B611] Knutson LV Vala JC (2011) Biology of Snail-killing Sciomyzidae Flies. Cambridge University Press, New York, xix + 506 pp.

[B612] Koçak AÖ Kemal M (2010) Synonymic and distributional list of the pterygot of Morocco (results of the Entomofauna of the World, based upon info-system of the Cesa): 1946–1979. PRIAMUS. Serial Publication of the Center for Entomological Studies, Ankara, Supplement 18, 3187 pp.

[B613] Koçak AÖ Kemal M (2013a) Diptera of Turkey. PRIAMUS, Serial Publication of the Centre for Entomological Studies, Ankara 28, 411 pp.

[B614] Koçak AÖ Kemal M (2013b) Tephritidae in Turkey. An evaluation of its status from various standpoints (Diptera). Centre for Entomological Studies Ankara. Cesa News 86: 1–50.

[B615] Korneyev VA Evstigneev DA Karimpour Y Kütük M Mohamadzade Namin S Ömür Ko­yuncu M Yaran M (2013) Revision of the *Terelliavirens* group (Diptera, Tephritidae) with description of three new species. Vestnik zoologii 47(1): 3–25. 10.2478/vzoo-2013-0001

[B616] Kovalev VG (1970) A redescription of the type specimen of Drapetis(s. str.)laevisDipteraEmpididae from Tangier, Morocco. Entomological Review 49(3): 688–690.

[B617] Kowarz F (1892) Die europäischen Arten der Dipterengattung Lispa Latr. Wiener entomologische Zeitung 11: 33–54. 10.5962/bhl.part.27698

[B618] Kozlowsky S Rungs C (1932) Sur quelques insectes ennemis des plantes maraichères au Maroc. Bulletin de la Société des sciences naturelles du Maroc 12: 66–68.

[B619] Kozlovsky S Rungs C (1933) Note sur *Depressariacynarivora* Meyr., ravageur du *Cynarascolymus* (artichaut) au Maroc. Bulletin de la Société des sciences naturelles du Maroc 12: 101–103.

[B620] Kremer M Hommel M Bailly-Choumara H (1971) Troisième contribution à l’étude faunistique des *Culicoides* du Maroc. Annales de Parasitologie (Paris) 46(5): 661–670. 10.1051/parasite/1971465661 5153515

[B621] Kremer M Rebholtz-Hirtzel C Bailly-Choumara H Délecolle JC (1975) Quatrième contribution à l’étude faunistique des *Culicoides* (Diptera, Ceratopogonidae) du Maroc. Description de *C.landauae* n. sp. (Diptera, Ceratopogonidae). Redescription de *C.faghihi* Navai et d’une forme de *C.subfascipennis* Kieffer. Cahiers ORSTOM, Série Entomologie médicale et Parasitologie 13(4): 205–214.

[B622] Kremer M Délecolle JC Bailly-Choumara H Chaker E (1979) Cinquième contribution à l’étude faunistique des *Culicoides* (Diptera, Ceratopogonidae) du Maroc. Description de *C.calloti* n. sp. Cahiers ORSTOM, Série Entomologie médicale et Parasitologie 17(3): 195–199.

[B623] Krimbas CD Loukas M (1980) The Inversion Polymorphism of *Drosophilasubobscura*. Evolutionary Biology 12: 163–234. 10.1007/978-1-4615-6959-6_4

[B624] Krivosheina MG (1998) A revision of the shore–fly genus *Notiphila* Fallén of Palaearctic (Diptera, Ephydridae). An International Journal of Dipterological Research 9(1): 31–63.

[B625] Kröber O (1913) Monographie der paläarktischen und afrikanischen Thereviden. (Dipt.) [part]. Deutsche Entomologische Zeitschrift 1: 17–32. 10.1002/mmnd.48019120102

[B626] Kröber O (1915) Die afrikanischen Arten der Gattung *Conops*. Archiv für Naturgeschichte (Abteilung A): 35–68. [Berlin]

[B627] Kröber O (1916) Die *Myopa*-Arten der nicht-palaearktischen Region. Archiv für Naturgeschichte (Abteilung A) 81: 23–39. [Berlin]

[B628] Kröber O (1924) Ägyptische Dipteren aus den Familien der Conopidae, Omphralidae, und Therevidae. Bulletin de la Société royale entomologique d’Egypte 7: 57–116. [Kairo]

[B629] Kröber O (1927) Beiträge zur Kenntnis der Conopidae. Konowia 6: 122–143. [Wien]

[B630] Kröber O (1928) Neue Dipteren aus Ägypten aus den Familien Therevidae, Omphralidae und Conopidae. Bulletin de la Société royale entomologique d’Egypte: 43–84. [Kairo]

[B631] Kröber O (1931) Nachträge zu meiner Arbeit: Die Conopidae Südafrikas in Annals of the Transvaal Museum XIV Part II. 1931. Konowia 12: 272–288.

[B632] Kroschel J Klein O (1999) Biological control of *Orobanche* spp. with *Phytomyzaorobanchia* Kalt., a review. In: Kroschel J Abderabihi M Betz H (Eds) Advances in Parasitic Weed Control at On-farm Level.Vol. II. Joint Action to Control Orobanche in the WANA Region. Margraf Verlag, Weikersheim, Germany, 135–159. [ix + 347 pp]

[B633] Kroschel J Klein O (2003) Biological control of *Orobanche* spp. in the Near East and North Africa by inundative releases of the herbivore *Phytomyzaorobanchia*. Proceedings of Expert Consultation on IPM for Orobanche in Food Legume System in the Near East and North Africa, Rabat, 128 pp.

[B634] Kügler J (1977) Neue Tachinidae aus Israel (Diptera). Stuttgarter Beiträge zur Naturkunde, Serie A (Biologie) 301: 1–14.

[B635] Kügler J Wool D (1968) Chironomidae (Diptera) from the Hula Nature Preserve Israel. Annales Zoologici Fennici 5: 76–83.

[B636] Kügler J Reiss F (1973) Die triangularis-Gruppe der Gattung *Tanytarsus* v.d.w. (Chironomidae, Diptera). Entomologisk Tidskrift 94: 59–82.

[B637] Kurahashi H (1971) The tribe Calliphorini from Australian and Oriental Regions. II. *Calliphora*-group (Diptera: Calliphoridae). Pacific Insects 13(1): 141–204.

[B638] Kurahashi H Magpayo FR (2000) Blow flies (Insecta: Diptera: Calliphoridae) of the Philippines. The Raffles Bulletin of Zoology, Supplement 9, 78 pp.

[B639] Kuznetzov SY (1997) Five new Palaearctic Syrphidae (Diptera). International Journal of Dipterological Research 8(4): 199–213.

[B640] Laboudi M Faraj C Sadak A Azelmate M Rhajaoui M El Aouad R (2012) Some environmental factors associated with *Anopheleslabranchiae* larval distribution during summer 2009, in Larache Province, Morocco. African Entomology 20(2): 229–238. 10.4001/003.020.0211

[B641] Laboudi M Sadak A Ouahabi S Daniela Boccolini D Faraj C (2014) Molecular characterization of *Anophelesmaculipennis* complex (Diptera: Culicidae) in Northern Morocco. Faunistic Entomology 67: 37–42

[B642] Lackschewitz P (1940a) Die palaarktischen Rhamphidiinen und Eriopterinen des Wiener Naturhistorischen Museums. Annalen des Naturhistorischen Museums Wien 50: 1–67.

[B643] Lackschewitz P (1940b) Die palaarktischen Limnophilinen, Anisomerinen und Pediciinen des Wiener Naturhistorischen Museums. Annalen des Naturhistorischen Museums Wien 50: 68–122.

[B644] Lahmar M Zeouienne M (1992) Données Bio-Ecologiques et importance des dégâts de la mineuse du pois-chiche (*Liriomyzacicerina* Rondani) au Maroc. Al Awamia 72: 108–118.

[B645] Lair X (2013) Les Diptères. In: Courtial C (Coord.) Invertébrés continentaux du littoral sableux breton, poursuite de l’inventaire des dunes et des plages sableuses, évaluation de l’impact d’activités humaines et valorisation des résultats. Contrat Nature, Rapport de synthèse. Conseil Régional de Bretagne, DREAL Bretagne, Conseils Généraux du Finistère, du Morbihan, des Côtes d’Armor et d’Ille-et-Vilaine, 93–120.

[B646] Landolt PJ Adams T Thomas S Rogg H (2012) Spotted wing *Drosophila*, *Drosophilasuzukii* (Diptera: Drosophilidae), trapped with combinations of wines and vinegars. Florida Entomologist 95(2): 326–332. 10.1653/024.095.0213

[B647] Langeron M (1938) Anophèles du Grand Atlas et de l’Anti Atlas marocain. Comptes Rendus des Sciences CCVIII, 260–262.

[B648] Larhbali Y Belghiti D El Guamri Y Lahlou O El Kharrim K Khamri Z El Madhi Y (2010) Sensibilité de deux moustiques Culicidés (*Anopheleslabranchiae* et *Culexpipiens*) aux insecticides. Bulletin de la Société de Pharmacie de Bordeaux 149: 33–42.

[B649] Larhbali Y Belghiti D El Guamri Y Lahlou O El Kharrim K Kirami A Khamri Z (2011) Cartographie de la faune culicidienne dans la province de Khémisset. Science Lib, Editions Mersenne, 3 (110603), 7 pp.

[B650] Larhbali Y Belghiti D El Guamri Y Lahlou O El Kharrim K Kirami A Khamri Z (2014) Situation épidémiologique du paludisme importé et étude entomologique des gîtes larvaires de localités à risque potentiel dans la province de Khémisset (Maroc). Médecine et Santé Tropicales 24: 397–402. 10.1684/mst.2014.0388 25295572

[B651] Larruga JM Cabrera VM González AM Gullón A (1983) Molecular and chromosomal polymorphism in continental and insular populations from the southwestern range of *Drosophilasubobscura*. Genetica 60: 191–205. 10.1007/BF00122374

[B652] Latorre A Moya A Ayala FJ (1986) Evolution of mitochondrial DNA in *Drosophilasubobscura*. Proceedings of the National Academy of Sciences of the United States of America 83: 8649–8653. 10.1073/pnas.83.22.8649 16578796 PMC386988

[B653] Latorre A Hernandez C Martinez D Castro JA Ramon M Moya A (1992) Population structure and mitochondrial DNA gene flow in Old World populations of *Drosophilasubobscura*. Heredity 68: 15–24. 10.1038/hdy.1992.2 1346531

[B654] Laville H Reiss F (1988) *Rheomus*, un nouveau genre du complexe *Harnischia* avec deux nouvelles especes d’Afrique du Nord (Diptera, Chironomidae). Spixiana Supplement 14: 183–190.

[B655] Laville H Reiss F (1993) The Chironomid fauna of the mediterranean region reviewed. Journal of Aquatic Ecology 26(2–4): 239–245. 10.1007/BF02255247

[B656] Laville H Langton P (2002) The lotic Chironomidae (Díptera) of Corsica (France). Annales de Limnology 38(1): 53–64. 10.1051/limn/2002006

[B657] Lebard T Haenni JP Martinez M (2020) Note sur la présence de *Chorisopstunisiae* (Becker, 1915) en France et de *Chorisopsmasoni* Troaino & Toscano, 1995 en France et en Espagne (Diptera, Stratiomyidae). Revue française d’entomologie générale 2(5–6): 94–106.

[B658] Leclercq M (1960) Tabanidae (Dipt.) du Maroc. I. *Pangoniusraclinae* n. sp. Note sur *Silviussingularis* Meigen. Bulletin de la Société des sciences naturelles et physiques du Maroc 40: 297–302.

[B659] Leclercq M (1961a) Syrphidae (Diptera) du Maroc. I. Extrait des Bulletins et annales de la Société entomologique de Belgique 97: 241–244.

[B660] Leclercq M (1961b) Tabanidae (Dipt.) du Maroc. II. Bulletin de l’Institut agronomique et des Stations de Recherches de Gembloux 29(2): 138–147.

[B661] Leclercq M (1967) Tabanidae (Diptera) du Maroc, III. Description de *Tabanuschoumarae* n. sp. Cah. ORSTOM. Série Entomologie médicale 5(2): 127–131.

[B662] Leclercq M (1968) Tabanidae (Diptera) du Maroc. IV. Diagnose de *Pangoniushassani* n. sp. Bulletin des Recherches agronomiques de Gembloux 3: 703–711.

[B663] Leclercq M (1986) Diptera: Family Tabanidae of Saudi Arabia (part 2). Fauna of Saudi Arabia 8: 340–343.

[B664] Leclercq M Olsufjev NG (1975) Catalogue des Tabanidae (Diptera) Paléarctiques. Bulletin & Annales de la Société royale belge d’entomologie 111: 25–36.

[B665] Leclercq M Maldès JM (1987) Inventaire des Tabanidae d’Algérie et du Maroc et description d’une espèce nouvelle (DipteraBrachycera). Nouvelle Revue d’Entomologie (N.S.) 4: 79–84.

[B666] Leclercq M Schacht W (1987) The Sciomyzidae of Morocco (Diptera, Sciomyzidae). Entomofauna 8(30): 449–451.

[B667] Léger N Rioux JA Croset H Soussi MC Benmansour N (1974) Le «complexe» Sergentomyia (Sergentomyia) antennata (Newstead, 1912). Remarques systématiques et écologiques à propos de 948 exemplaires récoltés dans le Sud-marocain. Annales de Parasitologie 49(5): 577–591. 10.1051/parasite/1974495577 4463765

[B668] Lehmann J (1970) Revision der europäischen Arten (Imagines und Puppen) der Gattung *Rheotanytarsus* Bause (Dipt, Chironomidae). Zoologischer Anzeiger 185: 345–378.

[B669] Lehmann J (1981) Chironomidae (Diptera) aus Fliessgewässern Zentralafrikas. II Teil: die Region um Kisangani, Zentralzaire. Spixiana Supplement 5: 3–85.

[B670] Lehr PA Ghahari H Ostovan H (2007) A contribution to the robber flies of subfamilies Stenopogoninae and Asilinae (Diptera: Asilidae) from Iran. Far Eastern Entomologist 173: 1–14.

[B671] Lehrer AZ (1975) Deux nouvelles espèces paléarctiques du genre *Sarcophaga* Meigen (Diptera, Sarcophagidae). Bulletin et Annales de la Société royale belge d’entomologie 111(4–6): 102–108.

[B672] Lehrer AZ (1976) Cinq espèces nouvelles pour la faune paléarctique des diptères Sarcophagidae. Annotationes zoologicae et botanicae Bratislava 115: 1–11.

[B673] Lehrer AZ (2007a) Une nouvelle espèce paléarctique du genre *Rhinia* R.D. (Diptera, Calliphoridae). Fragmenta Dipterologica 10: 21–23.

[B674] Lehrer AZ (2007b) Analyse des fausses et inconséquentes conceptions taxonomiques sur les genitalia mâles des Sarcophagidae et Calliphoridae (Diptera). Fragmenta Dipterologica 7: 1–28.

[B675] Letana SD (2014) Taxonomy of black scavenger flies (Diptera: Sepsidae) from Luzon, Philippines. Philippines Science Letters 7(1): 155–170.

[B676] Lhaloui S El Houssaini K Starks K (1988) Etude de la biologie et du niveau d’infestation de la mouche grise (*Phorbiacoarctata* Fall.) sur les céréales au Maroc. Rapport d’activité 1987–88, Programme Aridoculture, INRA–NIAC–CRRA, Maroc, 2 pp.

[B677] Lhaloui S Buschman L El Bouhssini M Amri A Hatchett JH Keith D Starks K El Houssaini K (1992) Infestations of *Mayetiola* spp. (Diptera: Cecidomyiidae) in Bread Wheat, Durum Wheat and Barley: Results of the five annual Surveys in the major Cereal growing Regions of Morocco. Al Awamia 77: 21–53.

[B678] Lhaloui S El Bouhssini M Amri A (2001) The Hessian fly in Morocco: Surveys, loss assessment, and genetic resistance in bread wheat. Bulletin of the International Organization of Biological Control for the “West Palaearctic Regional Section” 24(6): 101–107.

[B679] Lhaloui S El Bouhssini M Naserlhaq N Amri A Nachit M El Haddoury J Jlibène M (2005) Les cécidomyies des céréales au Maroc: Biologie, dégâts et moyens de lutte, INRA Rabat, Maroc, 52 pp.

[B680] Lhor Y Khayli M Bouslikhane M El Harrak M Fassi Fihri O (2015) Spatial and Seasonal Distribution of *Culicoides* Species in Morocco in relation to the Transmission of Bluetongue Viruses. British Journal of Virology 2: 88–95. 10.17582/journal.bjv/2015.2.6.88.95

[B681] Li X-y Pape T Zhang D (2019) *Gasterophilusflavipes* (Oestridae: Gasterophilinae): A horse stomach bot fly brought back from oblivion with morphological and molecular evidence. e. PLoS ONE 14(8): e0220820. [19 pp] 10.1371/journal.pone.0220820 PMC6690546 31404100

[B682] Likov L Vujić A Tubić NK Dan M Veličković N Rojo S Pérez-Bañon C Veselić S Barkalov A Hayat R Radenković S (2020) Systematic position and composition of *Merodonnigritarsis* and *M. avidus* groups (Diptera, Syrphidae) with a description of four new hoverfly species. Contributions to Zoology 89: 74–125. 10.1163/18759866-20191414

[B683] Lindner E (1936) 18. Stratiomyiidae [part]. Lieferung 104. In: Lindner E (Ed.) Die Fliegen der paläarktischen Region. Band IV. E. Schweizerbart’sche Verlagsbuchhandlung (Erwin Nägele), Stuttgart, 218 pp.

[B684] Lindner E (1939) Neue westpaläarktische Stratiomyiiden (Diptera). Zoologischer Anzeiger 127: 312–315.

[B685] Lindner E (1949) Notizen zu einigen palaearktischen Stratiomyiiden des Münchener Museums. Entomon, Munich 1: 179–180.

[B686] Lmimouni BE Baba NE Yahyaoui A (2004) Wound myiasis due to *Wohlfahrtiamagnifica*. First human case in Morocco. Bulletin de la Société de pathologie exotique 97(4): 235–237. 17304740

[B687] Loew H (1847) Dipterologische Beiträge. Zweiter Theil. Zu der öffentlichen Prüfung der Schüler des Königlichen Friedrich-Wilhelms-Gymnasiums, Posen [= Poznan], 50 pp.

[B688] Loew H (1860) Dipteren-Fauna Sudafrika’s. Erste Abtheilung. Abhandlungen des Naturwissenschaftlichen vereines für Sachsen und Thüringen in Halle 2: 57–402.

[B689] Londt JGH (2009) A review of Afrotropical *Sisyrnodytes* Loew, 1856 (Diptera: Asilidae: Stenopogoninae). African Invertebrates 50(1): 137–183. 10.5733/afin.050.0106

[B690] Londt JGH (2010) A Review of Afrotropical *Acnephalum* Macquart, 1838, Including the Reinstatement of *Sporadothrix* Hermann, 1907 and Descriptions of Two New Genera (Diptera: Asilidae: Stenopogoninae). African Invertebrates 51(2): 431–481. 10.5733/afin.051.0212

[B691] Louah A (1995) Ecologie des Culicidae (Diptera) et état du paludisme dans la péninsule de Tanger. Thèse doctorat Es-Sciences, Faculté des Sciences, Tétouan, 266 pp.

[B692] Louah A Ramdani M Saoud Y Mahjour J (1995) Biotypologie de la faune culicidienne de la péninsule tingitane. Bulletin de l’Institut scientifique, Rabat 19: 93–102.

[B693] Lyneborg L (1968) The genus *Acanthothereva* Séguy, 1935, with description of a new species from Algeria (Diptera, Therevidae). Journal of the Swiss Entomological Society 41: 1–4.

[B694] Lyneborg L (1969) Some Micropezidae, Psilidae, Platystomidae, Otitidae, Pallopteridae, Odiniidae, Aulacigasteridae, Atelestidae and Milichiidae (Diptera) collected in Southern Spain, with descriptions of six new species. Entomologiske Meddelelser 37: 27–46.

[B695] Lyneborg L (1989) Family Therevidae. In: Soós Á Papp L (Eds) Catalogue of Palaearctic Diptera.Vol. 6: Therevidae – Empididae. Akadémiai Kiadó, Budapest, 11–35. [435 pp]

[B696] Lyneborg L (2002) A New Species of *Phycus* (Dipt., Therevidae) from Morocco. The Entomologist’s Monthly Magazine 138: 11–13.

[B697] Lyneborg L Zaitzev VF (1980) *Hoplosathe*, a new genus of Palaearctic Therevidae (Diptera), with description of six new species. Entomologica scandinavica 11: 81–93. 10.1163/187631280X00400

[B698] Maa TC (1963) Genera and species of Hippoboscidae: Types, synonymy, habitats and natural groupings. Pacific Insects Monographs 6: 1–186. 10.1093/jmedent/1.1.4

[B699] Maa TC (1966) Studies in Hippoboscidae (Diptera): *Ornithoicazamicra*, *rabori*, *bistativa*, *tridens*, *simplicis*, *hovana*, *podargi*, *aequisenta*, *punctatissima*, *Pseudolynchiaserratipes*, n. spp.; *Lobolepis*, n. subg, for *Ornithoica*. Addendum. Pacific Insects Monographs 10: 139–140.

[B700] Maa TC (1969) A Revised checklist and concise host index of Hippoboscidae (Diptera). Pacific Insects Monographs 20: 261–299. 10.1093/jmedent/6.2.146

[B701] Maarouf A (2003) Diptères Cyclorrhaphes et Brachycères nouveaux pour le Maroc. L’entomologiste 59(1–2): 41–47.

[B702] Maarouf A Chemseddine M (1995) Surveillance de l’infestation des céréales par la mouche noire (*Phorbiasecuris* Tiensuu; Diptera, Anthomyiidae). Ecologia mediterranea 21(3–4): 93–99. 10.3406/ecmed.1995.1794

[B703] Maarouf A Chemseddine M Ramdani M (1996) Sur la mouche noire des céréales au Maroc *Phorbiasecuris* Tiensuu (Diptera, Anthomyiidae). L’entomologiste 52(1): 17–27.

[B704] Maamri A Pattee E Dolédec S Chergui H (2005) The benthic macroinvertebrate assemblages in the Zegzel–Cherraa, a partly temporary river system, Eastern Morocco. Annales de Limnologie – International Journal of Limnology 41(4): 247–257. 10.1051/limn/2005017

[B705] MacGowan I Freidberg A (2008) The Lonchaeidae (Diptera) of Israel, with descriptions of three new species. Israel Journal of Entomology 38: 61–92.

[B706] Macquart M (1834–1835) Histoire naturelle des insectes. Diptères 1. Ouvrage accompagné de planches. Roret, Paris, 578 pp. 10.5962/bhl.title.14274

[B707] Macquart J (1842) Diptères exotiques nouveaux ou peu connus. Tome deuxième. 2^ème^ partie. Mémoire de la Société Royale des sciences, de l’agriculture et des arts de Lille 1841(1): 65–200.

[B708] Maldès JM (2000) Confirmation de la présence au Maroc de *Satanasgigas* (Eversmann, 1855) et quelques compléments sur sa biologie (Diptera: Asilidae). Bulletin de la Société entomologique de France 105(4): 426–426. 10.3406/bsef.2000.16700

[B709] Mannheims B (1951) 15. Tipulidae. In: Lindner E (Ed.) Die Fliegen der palaearktischen Region 3(2), Lief.167: 1–64. [Stuttgart]

[B710] Mannheims B (1952) 15. Tipulidae. In: Lindner E (Ed.) Die Fliegen der palaearktischen Region 3(2), Lief.170: 65–112. [Stuttgart]

[B711] Mannheims B (1963) Eine *Tipula* der ostasiatischen *sempiterna*-Gruppe in Finnland (Dipt., Tipulidae). Notulae entomologicae 43: 69–74.

[B712] Mannheims B (1964) Zur Synonymie der europaischen Tipuliden (Dipt.), IX. Bonner zoologische Beiträge 15: 109–113.

[B713] Mannheims B (1967) 15. Tipulidae. In: Lindner E (Ed.) Die Fliegen der palaearktischen Region 3(2), Lief.270: 257–288. [Stuttgart]

[B714] Mannheims B (1968) 15. Tipulidae. In: Lindner E (Ed.) Die Fliegen der palaearktischen Region 3(5), Lief.275: 289–320. [Stuttgart]

[B715] Marc I Chibani A Alemad A Alkhali A Belala A Hadji M Belghyti D El kharrim K (2016) Etude écologique et entomologique des Culicides larvaires des gîtes de la province de Kénitra (Maroc). European Scientific Journal 12(32): 398–409. 10.19044/esj.2016.v12n32p398

[B716] Marcos-Garcia MA Vujić A Mengual X (2007) Revision of Iberian species of the genus *Merodon* (Diptera: Syrphidae). European Journal of Entomology 104: 531–572. 10.14411/eje.2007.073

[B717] Marshall SA (2012) Flies. The Natural History & Diversity of Diptera. Firefly Books, Richmond Hill, Ontario, 616 pp.

[B718] Marshall SA Roháček J Dong H Buck M (2011) The state of Sphaeroceridae (Diptera: Acalyptratae): a world catalog update covering the years 2000–2010, with new generic synonymy, new combinations, and new distributions. Acta Entomologica Musei Nationalis Pragae 51(1): 217–298.

[B719] Mason F Cerretti P Nardi G Whitmore D Birtele D Hardersen S Gatti E (2006) Aspects of biological diversity in the CONECOFOR plots. IV. The Invertebrate Biodiversity pilot project. Annali dell'Istituto Sperimentale per la Selvicoltura 30(Supplement 2): 51–70.

[B720] Mathis WN (1997) Shore Flies of the Belizean Cays (Diptera: Ephydridae). Smithsonian Contributions to Zoology 592: 1–77. 10.5479/si.00810282.592

[B721] Mathis WN Zatwarnicki T (1990) A review of the western Palaearctic species of *Athyroglossa* (Diptera: Ephydridae), Transactions of the American Entomological Society 116(1): 103–133.

[B722] Mathis WN Zatwarnicki T Krivosheina MG (1993) Studies of Gymnomyzinae (Diptera: Ephydridae), V: A Revision of the Shore-Fly Genus *Mosillus* Latreille, Smithsonian Contributions to Zoology 548, [iii +] 38 pp. 10.5479/si.00810282.548

[B723] Mathis WN Munari L (1996) World Catalog of the Family Tethinidae (Diptera). Smithsonian Contributions to Zoology 584: iv + 1–27. 10.5479/si.00810282.584

[B724] Mathis WN Sueyoshi M (2011) World Catalog and Conspectus of the Family Dryomyzidae (Diptera: Schizophora). Myia 12: 207–233.

[B725] Mathis WN Zatwarnicki T (2002) A Phylogenetic Study of the Tribe *Dryxini* Zatwarnicki (Diptera: Ephydridae). Smithsonian Contributions to Zoology 617. Smithsonian Institution Press, Washington DC, 112 pp. 10.5479/si.00810282.617

[B726] Mathis WN Zatwarnicki T (2012) Revision of New World Species of the Shore-fly SubgenusAllotrichoma Becker of the Genus Allotrichomawith Description of theSubgenusNeotrichoma (Diptera, Ephydridae, Hecamedini). ZooKeys 161: 1–101. 10.3897/zookeys.161.2016 PMC3267475 22303122

[B727] Matile L (1986) L’identité du «ver de la tipule de l’Agaric» de Réaumur, et notes taxonomiques sur les *Keroplatus* paléarctiques (Diptera, Mycetophiloidea, Keroplatidae). Annales de la Société entomologique de France 22(3): 353–367.

[B728] Maurice TJ (1947) The flies that cause myiasis in man. Miscellaneous publication №631. United States Departement of Agriculture, 173 pp.

[B729] Médail F Quézel P (1999) Biodiversity hotspots in the Mediterranean Basin: setting global conservation priorities. Conservation Biology 13: 1510–1513. 10.1046/j.1523-1739.1999.98467.x

[B730] Medioni J (1958) Le comportement de *Drosophilamelanogaster* Meigen dans un appareil à choix lumineux: étude comparative de souches sauvages de provenances géographiques diverses. Comptes rendus des séances de la Société de Biologie Paris 152: 1004–1007. 13609111

[B731] Meigen JW (1820) Systematische Beschreibung der bekannten europäischen zweiflügeligen Insekten. Zweiter Theil. F.W. Forstmann, Aachen, [x +] 363 pp.

[B732] Meigen JW (1830) Systematische Beschreibung bekannten europaeischen zweifliegelingen Insecten. Hamm 6, [iv,] 401 pp.

[B733] Mellor PS Pitzolis G (1979) Observations on breeding sites and light-trap collectionsof *Culicoides* during an outbreak of bluetongue in Cyprus. Bulletin of Entomological Research 69: 229–234. 10.1017/S0007485300017697

[B734] Mellor PS Boorman J Baylis M (2000) *Culicoides* biting midges. Their role as arbovirus vectors. Annual Review of Entomology 45: 307–340. 10.1146/annurev.ento.45.1.307 10761580

[B735] Mellor PS Hamblin C (2004) African horse sickness. Veterinary Research 35: 445–466. 10.1051/vetres:2004021 15236676

[B736] Mengual X (2018) A new species of *Ischiodon* Sack (Diptera, Syrphidae) from Madagascar. African Invertebrates 59(1): 55–73. 10.3897/afrinvertebr.59.24461

[B737] Menozzi P Krimbas CB (1992) The inversion polymorphism of *D.subobscura* revisited: Synthetic maps of gene arrangement frequencies and their interpretation. Journal of Evolutionary Biology 5: 625–641. 10.1046/j.1420-9101.1992.5040625.x

[B738] Menzel F Heller K (2004) Sechs neue Arten aus den Gattungen *Bradysia*, *Camptochaeta* und *Corynoptera* (Diptera: Sciaridae) nebst einigen Bemerkungen zur Nomenklatur europäischer Trauermücken. Studia dipterologica 11: 335–357.

[B739] Merz B (2004) Revision of the *Minettiafasciata* species-group (Diptera, Lauxaniidae). Revue suisse de Zoologie 111(1): 183–211. 10.5962/bhl.part.80234

[B740] Mesnil LP (1939) Descriptions d’espèces de Tachinaires (Dipt., Larvaevoridae). Bulletin et Annales de la Société royale belge d’entomologie 79: 209–212.

[B741] Mesnil LP (1946) Revision des Phorocerini de l’Ancien Monde (Larvaevoridae). Encyclopédie entomologique (B II) Diptera 10: 37–80.

[B742] Mesnil LP (1950) Notes sur les *Carceliina* (Dipt., Tachinidae) et révision des espèces d’Afrique. Revue de Zoologie et de Botanique Africaines 43: 3–24.

[B743] Mesnil LP (1952) Notes détachées sur quelques Tachinaires paléarctiques. Bulletin et Annales de la Société royale belge d’entomologie 88: 149–158.

[B744] Mesnil LP (1951) Larvaevorinae (Tachininae), 64g. In: Lindner E (Ed.) Die Fliegen der palaearktischen Region 10(1): 554 pp. [+ 17 pls] [E. Schweizerbart, Stuttgart]

[B745] Mesnil LP (1956) Larvaevorinae (= Tachininae), 64g. In: Lindner E (Ed.) Die Fliegen der palaarktischen Region: E.Schweizerbart, Stuttgart, 465–560.

[B746] Mesnil LP (1959) Description de *Myothyriabenoisti*, Larvaevorinae, Tachynina, Forêt de Mâamora. Stuttgarter Beiträge zur Naturkunde 23: 20.

[B747] Mesnil LP (1965) Larvaevorinae (Tachininae), 10(2–3). In: Lindner E (Ed.) Die Fliegen der palaearktischen Region.E. Schweizerbart, Stuttgart, 555–1435.

[B748] Messerlin A (1938) L’*Aedesmariae* au Maroc occidental. Bulletin de la Société de pathologie exotique et de ses filiales 31: 110–115.

[B749] Messerlin A Treillard M (1938) Sur une nouvelle station du groupe *Myzomyia* s. s. Anopheline en Afrique du Nord: A. *Myzomyiasergenti* Theobald au Maroc occidental. Bulletin de la Société de pathologie exotique 31: 107–109.

[B750] Mestari M (1997) Les peuplements culicidiens de la ville de Mohammedia et des régions avoisinantes: caractérisation hydrologique et hydrochimique des principaux gîtes et dynamique spatio-temporelle. Thèse de doctorat de 3ème cycle, Faculté des Sciences, Rabat, 138 pp.

[B751] Metge G (1986) Etude des écosystèmes hydromorphes (dayas et merjas) de la meseta marocaine: typologie et synthèse cartographique à objectif sanitaire appliquée aux populations d’*A.labranchiae* (Falleroni, 1926). Thèse doctorat Es-Sciences, Aix-Marseille III, 245 pp.

[B752] Metge G (1991) Contribution à l’étude d’*Anophelesmaculipennislabranchiae* au Maroc: activité des imagos et dynamique des stades préimaginaux dans la région de Sidi Bettache. Bulletin d’Ecologie 22(3–4): 419–426.

[B753] Metge G El Alaoui M (1987) Etude de la dynamique des populations d’*Aedesechinus* (Culicide dendrolimnique) en écophase aquatique au Maroc. Annales de Limnologie 23(2): 129–134. 10.1051/limn/1987009

[B754] Metge G Belakoul M (1989) Colonisation d’un nouvel habitat par *Culexpipiens* (Diptera: Culicidae) le creux d’arbre des Subéraies en pays Zaer, Maroc. Annales de Limnologie 25(1): 73–80. 10.1051/limn/1989009

[B755] Michelsen V (1980) The *Anthomyiapluvialis* complex in Europe (Diptera, Anthomyidae). Systematic Entomology 5: 281–290. 10.1111/j.1365-3113.1980.tb00416.x

[B756] Michelsen V Báez M (1985) The Anthomyiidae (Diptera) of the Canary Islands. Entomologica scandinavica 16: 277–304. 10.1163/187631285X00171

[B757] Mikuška A Stjepan Krčmar S Mikuska J (2008) Horseflies (Diptera: Tabanidae) of south-east Herzegovina (Bosnia and Herzegovina). Entomologia Croatica 12(2): 101–109.

[B758] Mimeur JM (1949) Contribution à l´étude des Zoocécidies du Maroc. Encyclopédie entomologique 24. P. Le Chevalier, Paris, 259 pp.

[B759] Mirceni RV Pârvu C (2009) Distributional data on some east Mediterranean Brachycera (Diptera) [Results of the Euphrates” Expedition – 2008, in Turkey and Syria]. Travaux du Muséum National d’Histoire Naturelle «Grigore Antipa» 52: 429–436.

[B760] Mohammadi DK Khaghaninia S (2015) Additional notes about the soldier flies (Diptera, Stratiomyidae) of Varzqan region-Iran. Entomofauna 36(15): 209–216.

[B761] Möhn E (1966) 6. Itonididae (= Cecidomyiidae). In: Lindner E (Ed.) Die Fliegen der palaearktischen Region 2(1), Lief.269: 1–48.

[B762] Möhn E (1971) 6. Itonididae (= Cecidomyiidae). In: Lindner E (Ed.) Die Fliegen der palaearktischen Region 2(1), Lief.288: 201–248.

[B763] Möhn E (1966–1971) 6.7. Cecidomyiidae (Itonididae). In: Lindner E (Ed.) Die Fliegen der palearktischen Region 2.Lieferung 269, 273, 274, 277, 288, 1–248.

[B764] Mohrig W Menzel F (1992) Neue Arten europäischer Trauermücken (Diptera, Sciaridae). International Journal of Dipterological Research 3: 1–16.

[B765] Mohrig W Kauschke E Menzel F Jaschhof M (1997) Trauermücken von der Kanarischen Insel La Gomera und West–Marokko (Diptera, Sciaridae). Berichte des Naturwissenschaftlich medizinischen Vereins in Innsbruck 84: 379–390.

[B766] Mohrig W Heller K Hippa H Vilkamaa P Menzel F (2012) Revision of the Black Fungus Gnats (Diptera: Sciaridae) of North America. Studia dipterologica 19: 141–286.

[B767] Mohrig W Heller K Hippa H Vilkamaa P Menzel F (2013) Revision of the Black Fungus Gnats (Diptera: Sciaridae) of North America. Studia dipterologica 19: 141–286.

[B768] Morgulis E Freidberg A Dorchin N (2016) Phylogenetic Revision of *Tephritomyia* Hendel (Diptera: Tephritidae), With Description of 14 New Species. Annals of the Entomological Society of America (2015) 109(4): 595–628. 10.1093/aesa/saw026

[B769] Motamedinia B Kehlmaier C Mokhtari A Rakhshani E Gilasian E (2017) The genus *Dasydorylas* Skevington in Iran, with the description of two new species (Diptera: Pipunculidae). European Journal of Taxonomy 362: 1–13. 10.5852/ejt.2017.362

[B770] Moubayed-Breil J Kettani K (2018) Description of *Rheotanytarsuslangtoni* sp. n. from the Rif of north-western Morocco [Diptera, Chironomidae, Tanytarsini]. Ephemera 19(2): 83–94.

[B771] Moubayed-Breil J Kettani K (2019) Rheocricotopus(s. str.)rifensis, a new rheobiontic species from waterfalls located in the Moroccan Rif [Diptera: Chironomidae, Orthocladiinae]. Ephemera 20(2): 87–97.

[B772] Mouna M (1998) Biodiversité des Invertébrés terrestres. In: Étude Nationale sur la Biodiversité. Deuxième Rapport nationale sur la Biodiversité du Maroc. Ministère de l’Environnement–PNUE [Programme des Nations Unies pour l'environnement], Rabat, iv + 125 pp.

[B773] Mouna M (2010) Les recherches entomologiques au Maroc de 1792 à 2006. Actes de la CIFE VI, Travaux de l’Institut Scientifique, Série Zoologie, Rabat 47(1): 79–84.

[B774] Moussalim S (1997) Zones humides naturelles et artificielles des régions nord-ouest marocaines; qualité hydrochimique et valeur biologique par l’analyse de la faune culicidienne. Thèse de doctorat de 3ème cycle, Faculté des Sciences, 138 pp.

[B775] Moussiaux AN Desmecht D (2008) Epidémiologie de l’infection par *Trypanosomaevansi*. Annales de Médecine Vétérinaire 152: 191–201.

[B776] Moutia LA (1940) The search for parasites of white grubs (Melolonthids) in Zanzibar, Algeria, Marocco and France. Bulletin of Entomological Research London 31: 193–208. 10.1017/S0007485300004971

[B777] Mulieri PR Patitucci LD Mariluis JM Pape T (2010) Long-distance introduction: first New World record of *Steveniadeceptoria* (Loew) and a key to the genera of New World Rhinophoridae (Diptera). Zootaxa 2524: 66–68. 10.11646/zootaxa.2524.1.6

[B778] Müller GC Hogsette JA Revay EE Kravchenko VD Leshvanov A Schlein Y (2011) New records for the horse fly fauna (Diptera: Tabanidae) of Jordan with remarks on ecology and zoogeography. Journal of Vector Ecology 36(2): 447–450. 10.1111/j.1948-7134.2011.00186.x 22129417

[B779] Müller GC Zeegers T Hogsette J Revay EE Kravchenko V Leshvanov A Schlein Y (2012) An annotated checklist of the horseflies (Diptera: Tabanidae) of Lebanon with remarks on ecology and zoogeography: Pangoniinae and Chrysopsinae. Journal of Vector Ecology 37(1): 216–220. 10.1111/j.1948-7134.2012.00219.x 22548556

[B780] Munari L (1993) Limosininae from Maghreb and Middle East collected by *A. Giordani* Soika (Diptera, Sphaeroceridae). Bolletino della Societá Entomologica Italiana, Genova 125(2): 150–156.

[B781] Munari L (1997) New records of Tethinidae (Diptera) with description of *Tethinamariae* sp. nov. from Morocco. Società Veneziana di Scienze Naturali-Lavori 22: 29–34.

[B782] Munari L (2002) Beach Flies (Diptera: Tethinidae) of the Palaearctic Region: an Annotated Checklist, including World Distribution. Società Veneziana di Scienze Naturali-Lavori, 27: 17–25.

[B783] Munari L (2004) On some species of Tethinidae from Morocco and Cape Verde Islands. Bollettino del Museo civico di Storia naturale di Venezia 55: 107–113.

[B784] Munari L (2005) Species of *Tethina* Haliday from the Sahara and inland biotopes of the Mediterranean subregion (Diptera: Tethinidae). Stuttgarter Beiträge zur Naturkunde, Serie A (Biologie) 683: 1–11.

[B785] Munari L (2010) Canacidae and Australimyzidae (Diptera) collected by Danish Scientific Expeditions and by N.L.H. Krauss, with descriptions of four new species. Steenstrupia 32(1): 51–68.

[B786] Munari L (2011) The Euro-Mediterranean Canacidae (including Tethinidae): keys and remarks to genera and species (Insecta, Diptera). Bollettino del Museo civico di Storia naturale di Venezia 62: 55–86.

[B787] Munari L Báez M (2000) The Tethinidae of Macaronesia: A faunal revision, with descriptions of two new species (Diptera). Bollettino del Museo civico di Storia naturale di Venezia (1999) 50: 3–30.

[B788] Munari L Bramuzzo S (2018) A Catalogue of the Canacidae housed in the Diptera Collection of the Natural History Museum of Venice (Italy) (Diptera, Brachycera, Carnoidea). Bollettino del Museo di Storia naturale di Venezia 69: 27–42.

[B789] Munari L Mathis WN (2010) World Catalog of the Family Canacidae (including Tethinidae) (Diptera), with keys to the supraspecific taxa. Zootaxa 2471: 1–84. 10.11646/zootaxa.2471.1.1

[B790] Murray DA (1980) *Telopelopiamaroccana* sp. n. a second palaearctic species of *Telopelopia* Roback (Diptera, Chironomidae). Acta Universitatis Carolinae. Serie Biologica (1978) 12: 151–156.

[B791] Naber M El Bouhssini M Labhilili M Udupa SM Nachit MM Baum M Lhaloui S Benslimane A El Abbouyi H (2000) Genetic variation among populations of the Hessian fly *Mayetioladestructor* (Diptera: Cecidomyiidae) in Morocco and in Syria. Bulletin of Entomological Research 90: 245–252. 10.1017/S0007485300000365 10996865

[B792] Naber N El Bouhssini M Lhaloui S (2003) Biotypes of Hessian fly (Diptera; Cecidomyiidae) in Morocco. Journal of Applied Entomology 127: 174–176. 10.1046/j.1439-0418.2003.00738.x

[B793] Naderloo M Pashaei Rad Sh Taghaddosi MV (2001) Faunistic study on hover flies (Diptera: Syrphidae) in the eastern part of Zanjan province, Iran. Journal of Entomological Research 4(4): 313–323.

[B794] Nagatomi A (1982) Geographical distribution of the lower Brachycera (Diptera). Pacific Insects 24(2): 139–150.

[B795] Naglis S (2009) New records of Sympycninae (Diptera, Dolichopodidae) from Turkey, with the description of a new species of *Teuchophorus*. Bulletin de la Société entomologique suisse 82: 173–180.

[B796] Naglis S Barták M (2015) Dolichopodidae (Diptera) from the Iberian Peninsula, with description of three new species. Zootaxa 3964(1): 125–137. [ISSN: 1175-5326] 26249426 10.11646/zootaxa.3964.1.9

[B797] Nardon C Deceliere G Loevenbruck C Weiss M Biemont C (2005) Is genome size influenced by colonization of new environments in dipteran species? Molecular Ecology 14: 869–878. 10.1111/j.1365-294X.2005.02457.x 15723678

[B798] Nartshuk EP (1979) Novij vid dvukrilikh roda *Cryptochetum* Rd. (Diptera, Cryptochetidae) parazit tshervetsov iz sprednei azii. Trudy Zoologicheskogo Instituta. Leningrad 88: 120–123. [in Russian]

[B799] Nartshuk EP (1984) Family Chloropidae. In: Soós Á Papp L (Eds) Catalogue of Palaearctic Diptera.Vol. 10: Clusiidae – Chloropidae. Akadémiai Kiadó, Budapest, 222–298. [402 pp]

[B800] Nartshuk EP (1988) Family Acroceridae. In: Soós Á Papp L (Eds) Catalogue of Palaearctic Diptera.Vol. 5: Athericidae – Asilidae. Akadémiai Kiadó, Budapest, 186–196. [446 pp]

[B801] Nartshuk EP (1995) A new species of *Cetema* Hendel with reference to the distribution of the genus (Insecta, Diptera, Choropidae), Spixiana 18(3): 277–281.

[B802] Naya A (1988) Peuplements chironormdiens (Diptera) du bassin du Haut et Moyen Sebou: Biotypologie et valeurs Bio-indicatrices. Thèse 3^ème^ cycle. Université Mohammed V. Rabat (Maroc), 127 pp.

[B803] Negrobov OP (1971) Revision of Palaearctic species of the genus *Thinophilus* Whlbg. (Diptera, Dolichopodidae). Entomologicheskoe Obozrenie 50(4): 896–910. [In Russian; English translation: Entomological Review, 1971, 50(4): 511–519]

[B804] Negrobov OP (1979) 29. DolichopodidaeHydrophorinae. In: Lindner E (Ed.) Die Fliegen der palaearktischen Region 4(5). E. Schweizerbart’sche Verlagsbuchhandlung, Stuttgart, Lief.321: 419–474. [taf. CLXXIV–LXXXVII, figs 1422–1659]

[B805] Negrobov OP (1991) Family Dolichopodidae. In: Sóos Á Papp L (Eds) Catalogue of Palaearctic Diptera.Vol. 7. Dolichopodidae – Platypezidae. Budapest, Akadémiai Kiadó, 11–139. 10.1016/B978-0-444-98731-0.50008-9

[B806] Negrobov OP Satô M Selivanova OV (2012) New species of the genus *Argyra* Macquart, 1834 (Diptera, Dolichopodidae) from the Russian Far East and Japan. Far Eastern Entomologist 247: 1–7.

[B807] Negrobov OP Naglis S (2016) Palaearctic species of the genus *Medetera* (Diptera: Dolichopodidae). Zoosystematica Rossica 25(2): 333–379. 10.31610/zsr/2016.25.2.333

[B808] Nigro L (1988) Natural populations of *Drosophilasimulans* show great uniformity of the mitochondrial DNA restriction map. Genetica 77: 133–136. 10.1007/BF00057763 3215515

[B809] Nikookar SH Moosa-Kazemi SH Oshaghi MA Yaghoobi-Ershadi MR Vatandoost H Kianinasab A (2010) Species Composition and Diversity of Mosquitoes in Neka County, Mazandaran Province, Northern Iran. Iran Journal of Arthropod-Borne Diseases 4(2): 26–34. PMC3385557 22808397

[B810] Norrbom AL Carroll LE Thompson FC White IM Freidberg A (1999) Systematic Database of Names. In: Thompson FC (Ed.) Fruit Fly Expert Identification System and Systematic Information Database.Myia (1998) 9, 65–251.

[B811] Norrbom AL (2004) Updates to Biosystematic Database of World Diptera for Tephritidae through 1999. Diptera Data Dissemination Disk 2.

[B812] Nourti M Grichanov IYa Kettani K (2019a) New records of long-legged flies (Diptera, Dolichopodidae) from Morocco. Acta Biologica Sibirica 5(3): 118–130. 10.14258/abs.v5.i3.6514

[B813] Nourti M Grichanov IYa Kettani K (2019b) A new species of *Teuchophorus* Loew, 1857 (Dolichopodidae, Diptera) from Morocco. Nature Conservation Research 4(4): 106–110. 10.24189/ncr.2019.064

[B814] Nourti M Kettani K Grichanov IYa (2019c) Faunistic notes on the genus *Sciapus* Zeller (Diptera, Dolichopodidae) of Morocco. Amurian Zoological Journal vol. XI, no. 4, 309–313. 10.33910/2686-9519-2019-11-4-309-313

[B815] Nsarellah N Amri A Nachit M El Bouhssini M Lhaloui S (2003) New durum wheat with Hessian fly resistance from *Triticumararaticum* and *Triticumcarthlicum* in Morocco. Plant Breeding 122: 435–437. 10.1046/j.1439-0523.2003.00871.x

[B816] Nsarella N Lhaloui S (2006) Les variétés de blé résistantes à la cécidomyie/ nouvel atout pour la céréaliculture au Maroc. Bulletin mensuel d’information et de liaison du PNTTA 140: 1–4.

[B817] Olafsson E (1991) Taxonomic revision of western Palaearctic species of the genera *Scatella* R.-D. and *Lamproscatella* Hendel, and studies on their phylogenetic position within the subfamily Ephydrinae (Diptera: Ephydridae). Entomologica scandinavica Supplement 37: 100 pp.

[B818] Oldroyd H (1957) The genus *Sisyrnodytes* Loew (Diptera: Asilidae). Proceedings of the Royal Entomological Society B 26(5–6): 79–88. 10.1111/j.1365-3113.1957.tb00383.x

[B819] Oldroyd H (1980) Family Asilidae. In: Crosskey RW (Ed.) Catalogue of the Diptera of the Afrotropical Region. London: British Museum (Natural History), 334–373, 1218, 1226, 1229.

[B820] Omelková M Ježek J (2012a) A new species of the genus *Trichomyia* (Diptera: Psychodidae) and new faunistic data on non-phlebotomine moth flies from the Podyjí NP and its surroundings (Czech Republic). Acta Entomologica Nusei Nationalis Pragae 52(2): 505–533.

[B821] Omelková M Ježek J (2012b) Two new species of *Pneumia* Enderlein (Diptera, Psychodidae, Psychodinae) from the Palaearctic region. Zootaxa 3180: 1–18. 10.11646/zootaxa.3180.1.1 28609995

[B822] Oosterbroek P (1978) The western Palaearctic species of *Nephrotoma* Meigen, 1803 (Diptera, Tipulidae), part 1. Beaufortia 27: 1–137.

[B823] Oosterbroek P (1979a) The western Palaearctic species of *Nephrotoma* Meigen, 1803 (Diptera, Tipulidae), part 2. Beaufortia 28: 57–111.

[B824] Oosterbroek P (1979b) The western Palaearctic species of *Nephrotoma* Meigen, 1803 (Diptera, Tipulidae), part 4, including a key to the species. Beaufortia 29: 129–197.

[B825] Oosterbroek P (1982) New taxa and data of western Palaearctic *Nephrotoma* (Diptera, Tipulidae). Entomologische berichten 42: 41–44.

[B826] Oosterbroek P (1994a) Notes on western Palaearctic species of the Tipula (Yamatotipula) lateralis group, with the description of a new species from Turkey (Diptera: Tipulidae). European Journal of Entomology 91: 429–435.

[B827] Oosterbroek P (1994b) Biodiversity of the Mediterranean Region. In: Forey PL Humphries CJ Vane-Wright RI (Eds) Systematics and Conservation Evaluation.Systematic Association, Special Volume 50, 289–307.

[B828] Oosterbroek P (2007) Diptera, Tipulidae. In: Nardi G Vomero V (Eds) Artropodi del Parco Nazionale del Vesuvio: Richerche preliminari.Conservazione Habitat Invertebrati 4: 377–454.

[B829] Oosterbroek P (2009) New distributional records for Palaearctic Limoniidae and Tipulidae (Diptera: Craneflies), mainly from the collection of the Zoological Museum, Amsterdam. In: Lantsov V (Ed.) Crane flies. History, taxonomy and ecology (Diptera: Tipulidae, Limoniidae, Pediciidae, Trichoceridae, Ptychopteridae, Tanyderidae). Memorial volume dedicated to Dr. Charles Paul Alexander (1889–1981), Dr. Bernhard Mannheims (1909–1971) and Dr. Evgeniy Nikolaevich Savchenko (1909–1994).Zoosymposia 3: 179–197. 10.11646/zoosymposia.3.1.15

[B830] Oosterbroek P (2011) The Craneflies of Sardinia (Diptera: Tipulidae). Conservazione Habitat Invertebrati 5: 641–658.

[B831] Oosterbroek P (2020) Catalogue of the Craneflies of the World (CCW). http://nlbif.eti.uva.nl/ccw/ [accessed March 2020]

[B832] Oosterbroek P Arntzen JW (1992) Area-cladograms of Circum-Mediterranean taxa in relation to Mediterranean palaeogeography. Journal of Biogeography 19: 3–20. 10.2307/2845616

[B833] Oosterbroek P Theowald Br (1992) Family Tipulidae. In: Soós Á Papp L (Eds) Catalogue of Palaearctic Diptera.Vol. 1: Trichoceridae – Nymphomyiidae. Akadémiai Kiadó, Budapest, 56–178. [520 pp]

[B834] Oosterbroek P Lantsov V (2011) Review of the Western Palaearctic species of *Dolichopeza* Curtis (Diptera, Tipulidae). Tijdschrift voor Entomologie 154: 269–281. 10.1163/004074912X13397496980987

[B835] Osborne RS Leppla NC Osborne LS (2002) Predatory Gall Midge (unofficial common name), *Feltiellaacarisuga* (Vallot) (Insecta: Diptera: Cecidomyiidae). University of Florida, EENY–269, 4 pp.

[B836] Ovazza M Camicas JL Pichon G (1968) Notes pour une révision systématique de l’espèce *Atylotusagrestis* (Wiedemann, 1828) Diptera: Tabanidae. Cahiers ORSTOM, Série Entomologie médicale 6(1): 3–14.

[B837] Özdikmen H (2008) *Turka* nom. nov., for the preoccupied Palaearctic robber flies genus *Turkiella* Lehr, 1996 (Diptera: Asilidae). Munis Entomology & Zoology 3(2): 554–555.

[B838] Ozerov AL (2005) World catalogue of the family Sepsidae (Insecta: Diptera). Zoologicheskie issledovania 8, 74 pp.

[B839] Pandey VS Ouhelli H Verhulst A (1992) Epidemiological observations on *Gasterophilusintestinalis* and *G.nasalis* in donkeys from Morocco. Veterinary Parasitology 41(3–4): 285–292. 10.1016/0304-4017(92)90087-P 1502790

[B840] Pape T (1996) Catalogue of the Sarcophagidae of the world (Insecta: Diptera). Memoirs of Entomology International 8: 1–558.

[B841] Pape T Blagoderov V Mostovski MB (2011) Order DIPTERA Linnaeus, 1758. In: Zhang ZQ (Ed.) Animal biodiversity: An outline of higher-level classification and survey of taxonomic richness.Zootaxa 3148: 222–229. 10.11646/zootaxa.3148.1.42 26146682

[B842] Pape T Szpila K (2012) Taxonomy and nomenclature of *Eremasiomyia ﻿﻿﻿macularis* and *Miltogrammamaroccana* (Diptera: Sarcophagidae: Miltogramminae). The Canadian Entomologist 144: 169–181. 10.4039/tce.2012.14

[B843] Pape T Thompson FC [Eds] (2019) Systema Dipterorum (version 2.0, Jan 2011). In: Roskov Y, Abucay L, Orrell T, Nicolson D, Bailly N, Kirk PM, Bourgoin T, DeWalt RE, Decock W, De Wever A, Nieukerken E, van Zarucchi J, Penev L (Eds) Species 2000 & ITIS Catalogue of Life, 29^th^ September 2017. Digital resource at www.catalogueoflife.org/col. Species 2000: Naturalis, Leiden, the Netherlands. ISSN 2405–8858. [accessed September 2019]

[B844] Papp L (1984a) Family Sphaeroceridae. In: Soós Á Papp L (Eds) Catalogue of Palaearctic Diptera.Vol. 10: Clusiidae – Chloropidae. Akadémiai Kiadó, Budapest, 68–107. [402 pp]

[B845] Papp L (1984b) Family Milichiidae. In: Soós Á Papp L (Eds) Catalogue of Palaearctic Diptera.Vol. 10: Clusiidae – Chloropidae. Akadémiai Kiadó, Budapest, 110–118. [402 pp]

[B846] Papp L (2007) Further Diptera species new for Hungary. Folia entomologica hungarica Rovartani Kozlemenyek 68: 111–122.

[B847] Paramonov SJ (1945) Bestimmungstabelle der palaearktischen *Nemestrinus* Arten (Nemestrinidae, Diptera) (Nebst Neubeschreibungen und Kritischen Bemerkungen). EOS-Revista Española de Entomología 21(3–4): 279–295.

[B848] Paramonov SJ (1947) Uebersicht der mit der gattung *Usia* latr. (Bombyliidae, Diptera) naechstverwandten gattungen. EOS-Revista Española de Entomología 23(3): 205–220.

[B849] Paramonov SJ (1950) Bestunmungstabelle der *Usia*-arten der Welt (Bombyliidae, Diptera). EOS-Revista Española de Entomología 26: 341–378.

[B850] Parent O (1924) Deux Dolichopodides nouveaux capturés au Maroc Espagnol par M. Asenci Codina. Treballs del Museu de Ciències Naturals de Barcelona 4(6): 1–15.

[B851] Parent O (1925) Trois Dolichopodides nouveaux de l’Afrique mineure (diptères). Bulletin de la Société royale entomologique d’Égypte 9: 186–197.

[B852] Parent O (1926) Notes synonymiques sur les espèces de Macquart (Dolichopodidés). Annales de la Société scientifique de Bruxelles (B) 46: 205–229.

[B853] Parent O (1927) Contribution à l’étude de la distribution géographique de quelques espèces de Dolichopodides. Comptes Rendus du congrès des sociétés savantes en 1926. Science, 449–484.

[B854] Parent O (1929a) Études sur les Dolichopodides. 1. Espèces nouvelles de Dolichopodides de la région paléarctique. Encyclopédie entomologique (B II) Diptera 5: 1–16.

[B855] Parent O (1929b) Les Dolichopodidae de la région éthiopienne. Etude systématique. Bulletin de la Société royale entomologique d’Égypte 13: 151–190.

[B856] Parent O (1933) Quelques espèces nouvelles de Diptères Dolichopodides de la région paléarctique. Annales de la Société scientifique de Bruxelles (B) 53: 74–78.

[B857] Parent O (1937a) Diptères Dolichopodidae nouveaux du Congo belge et du Maroc. Bulletin du Musée royal d'histoire naturelle de Belgique 13(18): 2–19.

[B858] Parent O (1937b) Diptères Dolichopodides, espèces et localités nouvelles. Bulletin et annales de la Société royale d’entomologie de Belgique 77: 125–148.

[B859] Parent O (1938) Faune de France 35. Diptères Dolichopodidae. Paris, 723 pp.

[B860] Paricio N Pascual L Martinez-Sebastian MJ de Frutos R (1991) Sequences homologous to P elements of *Drosophilamelanogaster* are widely distributed in *Drosophilasubobscura*. Drosophila Information Service 70: 171–172.

[B861] Pârvu C (1997) Some species of Dolichopodidae (Diptera) recorded from Serbia (Yugoslavia). Travaux du Muséum National d’Histoire Naturelle «Grigore Antipa» 39: 175–178.

[B862] Pârvu C (2008) Three Mediterranean Dolichopodidae little known. (Results of some Romanian expeditions in Israel, 1995 and Morocco, 2007). Travaux du Muséum d’Histoire Naturelle «Grigore Antipa» 51: 345–352.

[B863] Pârvu C Mirceni RP Zaharia R (2006) Faunistic data on some dipteran families (Insecta, Diptera) from Morocco (Results of «Hamada» Expedition 2005). Travaux du Muséum National d’Histoire Naturelle «Grigore Antipa» 49: 271–281.

[B864] Pârvu C Zaharia R (2007) Faunistic contributions on some Diptera families (Insecta : diptera) from Tunisia. Travaux du Muséum National d’Histoire Naturelle «Grigore Antipa» 50: 447–461.

[B865] Pascual M Constanti M Ribo G Prevosti A (1986) Sexual isolation between populations of *Drosophilasubobscura*. II. American and European strains. Genetica Iberica 38: 223–230.

[B866] Pascual M Constanti M Ribo G Prevosti A (1990) Genetic changes in mating activity in laboratory strains of *Drosophilasubobscura*. Genetica 80: 39–43. 10.1007/BF00120118 2323564

[B867] Pasteur N Verdier JM Rioux JA Guilvard H Perieres J (1978) Le complexe *Aedesdetridus* (Haliday, 1833): Existence de deux espèces jumelles en Afrique du Nord. Annales de Parasitologie 53(6): 761–763. 10.1051/parasite/1978536761 754628

[B868] Peck LV (1988) Family Syrphidae. In: Soós Á Papp L (Eds) Catalogue of Palaearctic Diptera.Vol. 8: Syrphidae – Conopidae. Akadémiai Kiadó, 11–230.

[B869] Peris SV (1951) Descripciónes preliminares de nuevos Rhiniini (Dip. Calliphoridae). EOS-Revista Española de Entomología 27: 237–247.

[B870] Peris SV (1963) Sobre los Rhinophorinae españoles con descripción de una nueva especie de *Chaetostavenia* de Marruecos (Dipt., Calliphoridae). EOS-Revista Española de Entomología 38: 601–609.

[B871] Peris SV Llorente V (1963) Notas sobre Muscini paleárticos y revisión de las especies españolas (Diptera, Muscidae). Boletin de la Real Sociedad Española de Historia Natural (Sección Biológica) 61(1–2): 209–269.

[B872] Peris SV González-Mora D Mingo E (1998) Los Heteronychiina de la Península Ibérica: Subgénero *Heteronychia* s.str., y descripción de una especie nueva de Tánger. (Diptera, Sarcophagidae). Boletin de la Real Sociedad Española de Historia Natural (Sección Biológica) 94(1–2): 165–178. [In Spanish with Spanish and English abstracts]

[B873] Pernot-Visentin O Beaucournu-Saguez F (1974) Les Tabanidae (Diptera) de France. Bulletin de la Société Linnéenne de Lyon, 43^è^ année 5: 142–155. 10.3406/linly.1974.10119

[B874] Pesson B Ready JS Benabdennbi I Martín-Sánchez J Esseghir S Cadi-Soussi M Morillas-Marquez F Ready PD (2004) Sandflies of the *Phlebotomusperniciosus* complex: mitochondrial introgression and a new sibling species of *P.longicuspis* in the Moroccan Rif. Medical and Veterinary Entomology 18(1): 25–37. 10.1111/j.0269-283x.2004.0471.x 15009443

[B875] Pierre C (1922a) Nematocera polyneura recueillis au Maroc par M. Charles Alluaud. Tipulidae. Bulletin de la Société des sciences naturelles du Maroc 1: 21–24.

[B876] Pierre C (1922b) Nematocera polyneura recueillis au Maroc par M. Charles Alluaud. Tipulidae. Bulletin de la Société des sciences naturelles du Maroc (2^è^ liste, 1920–1921) 1: 148–151.

[B877] Pierre C (1924a) Nematocera Polyneura receuillis au Maroc par M. Charles Alluaud (3C liste, 1922–1923) (Insectes Diptères). Bulletin de la Société des sciences naturelles du Maroc 4: 198–201.

[B878] Pierre C (1924b) Tipulidae nouveaux. Encyclopédie entomologique, Série B, Mémoires et Notes II, Diptera 1(2): 79–93.

[B879] Pinto FM Brehm A Hernandez M Larruga JM Gonzalez AM Cabrera VM (1997) Population genetic structure and colonization sequence of *Drosophilasubobscura* in the Canaries and Madeira Atlantic islands as inferred by autosomal, sex-linked and mtDNA traits. Journal of Heredity 88: 108–114. 10.1093/oxfordjournals.jhered.a023067 9099006

[B880] Piqué A (1994) Géologie du Maroc. Editions PUMAG. Marrakech, 284 pp.

[B881] Pitkin B Ellis W Plant C Edmunds R (2011) Leaf and stem mines of British flies and other insects. http://www.ukflymines.co.uk/resources.php

[B882] Pleske T (1930) Revue des espèces paléarctiques de la famille des Cyrtidae (Diptera). Konowia 9: 156–173.

[B883] Plus N Croizier G Jousset FX David J (1975) Picornaviruses of laboratory and wild *Drosophilamelanogaster*: Geographical distribution and serotypic composition. Annals of Microbiology 126A: 107–117. 811144

[B884] Plus N Croizier G Veyrunes JC David J (1976) A comparison of buoyant density and polypeptides of *Drosophila* P, C and A viruses. Intervirology 7: 346–350. 10.1159/000149975 1025039

[B885] Plus N Scotti PF (1984) The biological properties of eight different isolates of cricket paralysis virus. Annales de l’Institut Pasteur Virologie 135E: 257–268. 10.1016/S0769-2617(84)80027-1

[B886] Pollet M Stark A (2018) The quest for the identity of *Orthoceratiumlacustre* (Scopoli, 1763) reveals centuries of misidentifications (Diptera, Dolichopodidae). ZooKeys 782: 49–79. [+ Suppl. 1–15] 10.3897/zookeys.782.26329 PMC6160834 30275719

[B887] Pont AC (1970) *Myospilahennigi* Gregor and Povolný, 1959 (Dipt., Muscidae), new to Britain, and notes on the European species of *Myospila* Rondani, 1856. The Entomologist’s Monthly Magazine 106: 111–113.

[B888] Pont AC (1973) *Phaoniamediterranea* Hennig (Dipt., Muscidae), new to Britain. The Entomologist’s Monthly Magazine 108: 238–239.

[B889] Pont AC (1983) Notes on two Palaearctic species of *Fannia* Robineau-Desvoidy (Diptera, Fannidae). The Entomologist’s Monthly Magazine 119: 111–115.

[B890] Pont AC (1986a) Family Fanniidae. In: Soós Á Papp L (Eds) Catalogue of Palaearctic Diptera.Vol. 11: Scathophagidae – Hypodermatidae. Akadémiai Kiadó, Budapest, 41–57. [346 pp]

[B891] Pont AC (1986b) Family Muscidae. In: Soós Á Papp L (Eds) Catalogue of Palaearctic Diptera.Vol. 11: Scathophagidae – Hypodermatidae. Akadémiai Kiadó, Budapest, 57–215. [346 pp]

[B892] Pont AC (1991a) A Review of the Fanniidae and Muscidae (Diptera) of the Arabian Peninsula. Fauna of Saudi Arabia 12: 312–365.

[B893] Pont AC (1991b) A preliminary list of the Fanniidae and Muscidae (Insecta: Diptera) from Turkey and the Middle East. Zoology in the Middle East 5: 63–112. 10.1080/09397140.1991.10637603

[B894] Pont AC (2012a) Muscoidea (Fanniidae, Anthomyiidae, Muscidae) described by P.J.M. Macquart (Insecta, Diptera). Zoosystema 34(1): 39–111. 10.5252/z2012n1a3

[B895] Pont AC (2012b) *Helinarichardi* (Diptera: Muscidae), a remarkable new species from the Mediterranean subregion. The Canadian Entomologist 144: 348–352. 10.4039/tce.2012.31

[B896] Pont AC Singh B (1965) Oxford University Expedition to the High Atlas, Morocco 1963. Oxford, 14 pp.

[B897] Pont AC Meier R (2002) The Sepsidae (Diptera) of Europe. Fauna entomologica Scandinavica, 37. Brill, 198 pp. 10.1163/9789047401391

[B898] Pont AC Lole MJ LeBlanc HN Cole JH (2007) The American black dump fly *Hydrotaeaaenescens* (Wiedemann, 1830) (Diptera, Muscidae) in Britain and Ireland. Dipterists Digest (2), 14(1): 23–29.

[B899] Pont AC Harutyunova K Harutyunova M Werner D (2012) The hunter-flies of Armenia. III. New records of the genus *Limnophora* Robineau-Desvoidy, 1830, with the description of a new species. Zoology in the Middle East 57: 127–136. 10.1080/09397140.2012.10648972

[B900] Popescu-Mirceni RV (2011) Studiul sistematic şi zoogeographic asupra unor specii de diptere Brachicere (Diptera–Brachycera) din sectoarele Sudic şi estic ale subregiunii mediteraneene. Teză de doctorat, Universitatea din Bucuresti, Facultatea de Biologia. Bucuresti, 256 pp. [in Romanian]. [A systematic and zoogeographic study on some Brachycera species (Diptera, Brachycera) from the South and the Eastern part of the submediterranean subregion].

[B901] Popov GV Prokhorov AV Kustov SYu (2020) Revision of the *Melanogasterjaroslavensis* group (Diptera: Syrphidae), with description of a new species from Afghanistan. Zootaxa 4743(4): 536–552. 10.11646/zootaxa.4743.4.4 32230312

[B902] Popović D Ačanski J Djan M Obreht D Vujić A Radenković S (2015) Sibling species delimitation and nomenclature of the *Merodonavidus* complex (Diptera: Syrphidae). European Journal of Entomology 112(4): 790–809. 10.14411/eje.2015.100

[B903] Portillo M (1989) Tabanidae (Díptera) de España: VI. *Tabanus* Linnaeus, 1758. Boletín de la Asociación española de Entomología 13: 407–430.

[B904] Preradović J Andrić A Radenković S Šašić Zorić L Pérez-Bañón C Campoy A Vujić A (2018) Pupal stages of three species of the phytophagous genus *Merodon* Meigen (Diptera: Syrphidae). Zootaxa 4420(2): 229–242. 10.11646/zootaxa.4420.2.5 30313544

[B905] Prevosti A (1971a) Chromosomal polymorphism in *Drosophilasubobscura* Coll. populations from the Canary Islands. Genetica Iberica 23: 69–84.

[B906] Prevosti A (1971b) Ordenaciones cromosomicas en poblaciones de *Drosophila*subobscura del sur de Marruecos. Book: Resum. Comun. VIII. Journal of Genetics, Luso–Española, 63 pp.

[B907] Prevosti A (1971c) Polimorphisme cromosomatic en poblacions de *Drosophilasubobscura* Coll. de Madeira. Treballs de la Societat Catalana de Biologia 30: 61–69.

[B908] Prevosti A (1974) Chromosomal inversion polymorphism in the southwestern range of *Drosophilasubobscura* distribution area. Genetica 45: 111–124. 10.1007/BF01508935

[B909] Prevosti A (1978) Polimorphismo cromosomico y evolucion. Investigacion y Ciencia, Barcelona 26: 90–103.

[B910] Prevosti A Ocana J Alonso G (1975) Distances between populations of *Drosophilasubobscura*, based on chromosome arrangement frequencies. Theoretical and Applied Genetics 45: 231–241. 10.1007/BF00831894 24419466

[B911] Prevosti A Serra L Ribo G Aguade M Sagarra E Monclus M Pilar Garcia M (1985) The colonization of *Drosophilasubobscura* in Chile. II. Clines in the chromosomal arrangements. Evolution 39(4): 838–844. 10.1111/j.1558-5646.1985.tb00425.x 28561355

[B912] Prudhomme J Gunay F Rahola N Ouanaimi F Guernaoui S Boumezzough A Banuls AL Sereno D Alten B (2012) Wing size and shape variation of *Phlebotomuspapatasi* (Diptera: Psychodidae) populations from the south and north slopes of the Atlas Mountains in Morocco. Journal of Vector Ecology 37: 137–147. 10.1111/j.1948-7134.2012.00210.x 22548547

[B913] Raclin J (1957) Une famille de Diptères nouvelle pour le Maroc (Phoride: *Diploneuracrassicornis*). Société des sciences naturelles et physiques du Maroc 23(4): 59–60.

[B914] Ramdani M (1997) Hydrogéologie de quelques écosystèmes côtiers et action des produits chimiques et bactériologiques sur les larves de Moustiques de la meseta côtière (Témara – Casablanca). Thèse de doctorat de 3ème cycle, Faculté des Sciences, Rabat, 115 pp.

[B915] Ramdani M (1981) Recherches hydrobiologiques sur la Merja Sidi Boughaba. Bulletin de l’Institut scientifique, Rabat 5: 73–133.

[B916] Ramdani M Tourenq JN (1982) Contribution à l’étude faunistique de la Merja de Sidi Bou Ghaba. Bulletin de l’Institut scientifique, Rabat 6: 179–223.

[B917] Raspi A Pisciotta S Sajeva M (2009) *Milichiellalacteipennis*: new record for Lampedusa Island (Italy). Bulletin of Insectology 62(2): 133–135.

[B918] Reemer M Hauser M Speight M (2004) The genus *Myolepta* Newman in the West-Palaearctic region (Diptera, Syrphidae). Studia dipterologica 11(2): 553–580.

[B919] Reiss F (1977) Verbreitungsmuster bei palàarktischen Chironomidenarten (Diptera, Chironomidae). Spixiana 1: 85–97.

[B920] Reiss F (1985) Contribution to the zoogeography of the turkish Chironomidae (Diptera). Israel Jourrnal of Entomology 19: 161–170.

[B921] Reiss F (1986) Ein Beitrag zur chironomidenfauna Syriens (Diptera, Chironomidae). Entomofauna Zeitschrift für Entomology 7(11): 153–168.

[B922] Reiss F (1987) *Tanytarsuscretensis* sp. n. eine neue westpalaeaktische Chironomidenart aus Fliessgewässern (Diptera, Chironomidae). Nachrichtenblatt der Bayerischen Entomologen 36: 26–30.

[B923] Reiss F (1991) Drei neue Tanytarsini-Arten aus Marokko (Diptera, Chironomidae). Nachrichtenblatt der Bayerischen Entomologen 40(2): 45–52.

[B924] Reiss F Fittkau EJ (1971) Taxonomie und Ökologie europaisch verbreiteter *Tanytatsus*-Arten (Chironomidae, Diptera). Archiv fuer Hydrobiologie 40: 75–200.

[B925] Reiss F Säwedal L (1981) Keys to males and pupae of the Palaearctic (excl. Japan) *Paratanytasus* Thienemann & Bause, 1913, n. comb., with description of three new species (Diptera: Chironomidae). Entomologica scandinavica Supplement 15: 73–104.

[B926] Reiss F Schurch M (1984) *Virgatanytasusansatus* n. sp. aus Mitteleuropa und Nordafrika. Spixiana 7(3): 319–322.

[B927] Remm H (1988a) Family Ceratopogonidae. In: Soós Á Papp L (Eds) Catalogue of Palaearctic Diptera.Vol. 3: Ceratopogonidae – Mycetophilidae. Akadémiai Kiadó, Budapest, 11–110. [448 pp]

[B928] Remm H (1988b) Family Leptoconopidae. In: Soós Á Papp L (Eds) Catalogue of Palaearctic Diptera.Vol. 3: Ceratopogonidae – Mycetophilidae. Akadémiai Kiadó, Budapest, 110–114. [448 pp]

[B929] Ricarte A Marcos-García MÁ Rotheray GE (2008) The early stages and life histories of three *Eumerus* and two *Merodon* species (Diptera: Syrphidae) from the Mediterranean region. Entomologica Fennica 19(3): 129–141. 10.33338/ef.84424

[B930] Rioux JA (1958) Les Culicides du Midi méditerranéen. P. Lechevalier, Paris, 303 pp.

[B931] Rioux JA Croset H Léger N Bailly-Choumara H (1974) Phlebotomus (Larroussius) mariae n. sp. (Diptera: Psychodidae). Annales de Parasitologie (Paris) 49: 91–101. 10.1051/parasite/1974491091

[B932] Rioux JA Croset H Léger N Benmansour N Cadi Soussi M (1975) Présence au Maroc de *Phlebotomusbergeroti*, *Phlebotomuschabaudi*, *Phlebolomuschadlii* et *Sergentomyiachristophersi*. Annales de Parasitologie Humaine et Comparée (Paris) 50: 493–506. 10.1051/parasite/1975504493 1211776

[B933] Rioux JA Cousserans J Croset H Ben Osman F Gabinaud A Sinègre G Belmonte A (1975) Présence du caractère autogène chez *Aedespullatus* (Coquillett, 1904) et nouvelles localisations géographiques pour *Aedescaspius* (Pallas, 1771), *Aedesmariae* (Sergent, 1903), *Aedesdetritus* (Haliday, 1833) et *Culisetasubochrea* (Edwards, 1921). Annales de Parasitologie Humaine et Comparée 50: 131–142. 10.1051/parasite/1975501131 240306

[B934] Rioux JA Croset H Léger N Rosin G (1977) Presence of *Phlebotomusperfiliewi* Parrot, 1930 in Morocco. Annales de Parasitologie Humaine et Comparée (Paris) 52(3): 377–380. 10.1051/parasite/1977523377 921196

[B935] Ristorcelli A (1941) Sur les Phlébotomes du Maroc – Deuxième note. Archives de l’Institut Pasteur du Maroc 21: 521–533. 18902605

[B936] Ristorcelli A (1946a) Sur la présence à Marrakech d’*Anopheleshispaniola*. Annales de Parasitologie 21: 1–4.

[B937] Ristorcelli A (1946b) Sur la zoophilie d’*Anopheleshispaniola*. Annales de Parasitologie 21: 93–96. 10.1051/parasite/1946211093 21027457

[B938] Ristorcelli A (1947) Sur les Phlébotomes du Maroc – Troisième note. Archives de l’Institut Pasteur du Maroc 23: 105–109. 18902605

[B939] Ritchie MG Yate VH Kyriacou CP (1994) Genetic variability of the interpulse interval of courtship song among some European populations of *Drosophilamelanogaster*. Heredity 72: 459–464. 10.1038/hdy.1994.64 8014057

[B940] Robineau-Desvoidy AJB (1830) Essai sur les Myodaires. Mémoires présentés par divers Savants à l’Académie Royale des Sciences de l’Institut de France 2: 813 pp. [+31.xii.1830]

[B941] Rognes K (1987) The taxonomy of the *Polleniarudis* species-group in the Holarctic Region (Diptera: Calliphoridae). Systematic Entomology 12: 475–502. 10.1111/j.1365-3113.1987.tb00219.x

[B942] Rognes K (1991a) Blowflies (Diptera, Calliphoridae) of Fenno­scandia and Denmark. Fauna entomologica Scandinavica 24: 1–272.

[B943] Rognes K (1991b) Revision of the cluster-flies of the *Polleniaviatica* species-group (Diptera: Calliphoridae). Systematic Entomology 16: 439–498. 10.1111/j.1365-3113.1991.tb00678.x

[B944] Rognes K (1992) Revision of the cluster-flies of the *Polleniavagabunda* species-group (Diptera: Calliphoridae). Entomologica scandinavica 23(1): 95–114. 10.1163/187631292X00056

[B945] Rognes K (2002) Blowflies (Diptera, Calliphoridae) of Israel and adjacent areas with a new species from Tunisia. Entomologica scandinavica Supplements 59, 148 pp. [+ 81pls + 302 figs]

[B946] Rognes K (2010) Revision of the cluster flies of the *Polleniahaeretica* species-group (Diptera, Calliphoridae). Zootaxa 2499: 39–56. 10.11646/zootaxa.2499.1.3

[B947] Rognes K (2011) Short notes. 12. Diptera, Calliphoridae. In: Nardi G Whitmore D Bardiani M Birtele D Mason F Spada L Cerretti P (Eds) Biodiversity of Marganai and Montimannu (Sardinia).Research in the framework of the ICP Forests network. – Conservazione Habitat Invertebrati 5. Cierre Edizioni, Sommacampagna, Verona, 852–857.

[B948] Roháček J (1991) A monograph of *Leptocera* (*Rachispoda* Lioy) of the West Palaearctic area (Diptera, Sphaeroceridae). Časopis Slezského zemského muzea, Opava (A), 40: 97–288.

[B949] Roháček J (2006) A monograph of Palaearctic Anthomyzidae (Diptera) Part 1. Opava, Czech Republic. Časopis Slezské­ho zemské­ho muzea, Opava (A) 55 supplement 1, 330 pp.

[B950] Roháček J (2007) The Sphaeroceridae (Diptera) of Madeira, with notes on their biogeography. Časopis Slezského zemského muzea, Opava (A) 56: 97–122.

[B951] Roháček J Marshall SA Norrbom AL Buck M Quiros DI Smith I (2001) World catalog of Sphaeroceridae (Diptera). Časopis Slezského zemského muzea, Opava, 395 pp.

[B952] Rohdendorf BB (1937) Fam. Sarcophagidae. (P. 1). Fauna USSR 19: [xv +] 501 pp. [+ [1]] [In Russian with German summary]

[B953] Rohmer C David JR Moreteau B Joly D (2004) Heat induced male sterility in *Drosophilamelanogaster*: adaptive genetic variations among geographic populations and role of the Y chromosome. Journal of Experimental Biology 207: 2735–2743. 10.1242/jeb.01087 15235002

[B954] Romeo Viamonte JM (1950) Los anofelinos de España y de la zona española del Protectorado de Marruecos. Su relación con la difusión del paludismo. (Tesis Doctoral). Revista de Sanidad e Higiene Pública 24: 213–295. 15441539

[B955] Röschmann F Mohrig W (1993) *Corynopterajeskei* Moh. & Rösch., eine neue Species der *C.fulvicollis*-Gruppe aus Marokko. (Insecta: Diptera: Sciaridae). Reichenbachia 30: 109–111.

[B956] Rossi W Santamaría S Andrade R (2013) Notes on the Laboulbeniales (Ascomycota) parasitic on Diptera from Portugal and other countries, Plant Biosystems – An International Journal Dealing with all Aspects of Plant Biology: Official Journal of the Societa Botanica Italiana 147(3): 730–742. 10.1080/11263504.2012.753132

[B957] Rozkošný R (1977) The West Palaearctic species of *Nemotelus* Geoffroy (Diptera, Stratiomyidae). Folia facultatis scientiarum naturalium Universitatis Purkynianae Brunensis 17, Biology 51(3): 1–105.

[B958] Rozkošný R (1982) Three new species of *Pherbellia* and new synonyms of Holarctic and Palaearctic Sciomyzidae (Diptera). Annales entomologici Fennici 48(2): 51–56.

[B959] Rozkošný R (1982–1983) A Biosystematic Study of the European Stratiomyidae (Diptera). Vols 1–2. W. Junk, The Hague–Boston–London, 401 + 431 pp.

[B960] Rozkošný R (1987) A review of the Palaearctic Sciomyzidae (Diptera). Folia Facultatis Scientiarum Naturalium.Universitatis Masarykianae Brunensis, Biologia 86: 100 pp. [+ 56 pls]

[B961] Rübsaamen EH (1899) Mittheilungen über neue und bekannte Gallen aus Europa, Asie, Africa und America. Entomologische Nachrichten 25: 225–282.

[B962] Rungs CEE (1952) Contribution à la connaissance de l’ennemie de l’Arganier, *Arganiaspinosa* (L.). Bulletin de la Société scientifique naturelle du Maroc 32: 61–76.

[B963] Rungs CEE (1962) La faune nuisible à la betterave. Al Awatania 3: 161–174.

[B964] Rungs Ch (1940) Les ennemis du Cèdre au Maroc. Comptes-Rendus des séances de la Société des sciences naturelles du Maroc Tome V, 14–16.

[B965] Rungs Ch (1954) La chenille arpenteuse de l’Acacia à Tanin. Service de la Défense des Végétaux, Travaux originaux 5: 57 pp. [Rabat]

[B966] Sabrosky CW (1965) DipteraChloropidae – Mission zoologique de l’I.R.S.A.C. en Afrique orientale. (P. Basilewsky et N. Leleup 1957). Annales du Musée Royal de l’Afrique centrale (Série in–8° – Sciences zoologiques) 138: 401–412. [Tervuren]

[B967] Sabrosky CW (1980) 80. Family Chloropidae. In: Crosskey RW, Cogan BH, Freeman P, Pont AC, Smith KGV, Oldroyd H (Eds) Catalogue of the Diptera of the Afrotropical Region. British Museum (Natural History), London, 695–712 and 1219. [1437 pp]

[B968] Saccà G (1952) Due mosche nuove per la fauna d’Europa: *Muscasorbens* Wied. e*Limnophoratonitrui* Wied., in Sicilia (Diptera, Muscidae). Rivista di Parassitologia 13(2): 177–180.

[B969] Saccà G (1960) Contributo alla conoscenza delle *Myzomyia* del Sud Marocchino. Rendiconti dell'Istituto superiore di Sanità 23: 275–580.

[B970] Saccá G Guy Y (1960) Résistance de comportement au DDT chez *A.labranchiae* au Maroc. Bulletin de l’Organisation Mondiale de la Santé 22(6): 735–741. PMC2555346 14440582

[B971] Saether OA (1968) Chironomids of the finse Area Norway with special reference to their distribution in a glacier brook. Archiv fuer Hydrobiologie 64: 426–483.

[B972] Sahebaria FS Khaghaninia S Zieglerb J Gilasianc E Talebid AA (2016) On the fauna of the subfamily Phasiinae (Diptera: Tachinidae) in northwestern Iran. Zoology and Ecology 26(3): 181–190. 10.1080/21658005.2016.1174504

[B973] Sahib S Driauach O Belqat B (2020) New data on the Hoverflies of Morocco (Diptera: Syrphidae) with Faunistic and Bibliographical inventory. ZooKeys 971: 59–103. 10.3897/zookeys.971.49416 33061773 PMC7538468

[B974] Samin N Sakenin H Imani S (2011) A contribution to the knowledge of robber flies (Diptera: Asilidae) from some regions of Iran. Calodema 159: 1–5.

[B975] Sánchez Terrón A Roldan Bravo AV (2000) El género*Thyridanthrax* Osten-Sacken, 1886 en el área ibero-balear, con la descripción de una nueva especie (Diptera, Bombyliidae). Boletín de la Asociación española de Entomología 24(1–2): 65–84.

[B976] Sarvašová A Kočišová A Halán M Delécolle JC Mathieu B (2014) Morphological and molecular analysis of the genus *Culicoides* (Diptera: Ceratopogonidae) in Slovakia with five new records. Zootaxa 3872(5): 541–560. 10.11646/zootaxa.3872.5.6 25544100

[B977] Savchenko EN Oosterbroek P Starý J (1992) Family Limoniidae. In: Soós Á Papp L (Eds) Catalogue of Palaearctic Diptera.Vol. 1: Trichoceridae – Nymphomyiidae. Akadémiai Kiadó, Budapest, 183–369. [520 pp]

[B978] Savini G Goffredo M Monaco F Di Gennaro A Cafiero MA Baldi L de Santis P Meiswinkel R Caporale V (2005) Bluetongue virus isolations from midges belonging to the *Obsoletus* complex (*Culicoide*s, Diptera: Ceratopogonidae) in Italy. Veterinary Record 157: 133–139. 10.1136/vr.157.5.133 16055660

[B979] Savio C (2011) Symbiotic and associated bacteria in Tephritid flies. Scuola Di Dottorato Di Ricerca in Scienze delle Produzioni Vegetali. Universita’ Degli Studi di Padova, 159 pp.

[B980] Schacht W Kurina O Merz B Gaimari S (2004) Zweiflügler aus Bayern XXIII (Diptera: Lauxaniidae, Chamaemyiidae). Entomofauna 25(3): 41–80.

[B981] Schlinger EI (1972) New East Asian and American genera of the “*Cyrtus*–*Opsebius*” Branch of the Acroceridae (Diptera). Pacific Insects 14(2): 409–428.

[B982] Schmid U (1995) Neu beschriebene paläarktische Schwebfliegenarten (Diptera, Syrphidae) und neue Synonyme: eine Übersicht. Volucella 1: 29–44.

[B983] Schmitz H (1920) Die Phoriden von Holländisch Limburg mit Bestimmungstabellen aller bisher kenntlich beschrieben europäischen Phoriden. IV. Teil. Jaarboek natuurhistorisch Genootschap in Limburg 1919, 91–153.

[B984] Schmitz H (1949) 33. Phoridae. In: Lindner E (Ed.) Die Fliegen der palaearktischen Region 4(160): 193–240.

[B985] Schumann H (1974) Revision der palaearktischen *Bellardia*-Arten (Diptera, Calliphoridae). Deutsche entomologische Zeitschrift (N.F.) 21: 231–299. 10.1002/mmnd.4810210402

[B986] Schumann H (1986) Family Calliphoridae. In: Soós Á Papp L (Eds) Catalogue of Palaearctic Diptera.Vol. 12: Calliphoridae – Sarcophagidae. Akadémiai Kiadó, Budapest, 11–58. [265 pp]

[B987] Séguy E (1924) Les insectes parasites de l’homme et des animaux domestiques. Paris (P. Lechevalier), 422 pp.

[B988] Séguy E (1925) Description d’un nouveau *Simulium* et synopsis des espèces méditerranéennes (Dipt., Simuliidae). EOS – Revista Espagñola de Entomología 1: 231–238.

[B989] Séguy E (1926) 13. Diptères (Brachycères) (Stratiomyidae, Erinnidae, Coenomyiidae, Rhagionidae, Tabanidae, Codidae, Nemestrinidae, Mydaidae, Bombyliidae, Therevidae, Omphralidae). Faune de France 13: 1–308. [Paris]

[B990] Séguy E (1928a) Diptères nouveaux d´Afrique mineure. Bulletin de la Société entomologique de France 1: 45–46. 10.3406/bsef.1928.27937

[B991] Séguy E (1928b) Description d’un nouveau calliphorine (Dipt.). Bulletin de la Société entomologique de France 97: 152–154. 10.3406/bsef.1928.27992

[B992] Séguy E (1928c) Etude sur quelques Mydaidae nouveaux ou peu connus. Encyclopédie entomologique, Série B II, Diptera 4(3): 129–156.

[B993] Séguy E (1928d) Etudes sur les Mouches parasites. I. Conopides, Oestrides et Calliphorines de l’Europe occidentale. Encyclopédie entomologique, Paris 9: 101–116.

[B994] Séguy E (1928e) Etude sur le *Pollenia Hasei*. Zeitschrift für angewandte Entomologie 28: 369–375. 10.1111/j.1439-0418.1929.tb00082.x

[B995] Séguy E (1929a) Une Pangonie nouvelle du Maroc (Tabanidae). Encyclopédie entomologique, Diptères 5: 100.

[B996] Séguy E (1929b) Etude sur les Diptères à larves commensales ou parasites des oiseaux de l’Europe occidental. Encyclopédie entomologique, Diptera 5: 63–82.

[B997] Séguy E (1930a) Contribution à l’étude des Diptères du Maroc. Mémoires de la Société des sciences naturelles du Maroc 24, 207 pp.

[B998] Séguy E (1930b) Notes sur les moustiques III (1): l’*Anophelescostalis* en Europe méridionale. Encyclopédie entomologique, Diptera 5: 177–178.

[B999] Séguy E (1932a) Étude sur les diptères parasites ou prédateurs des sauterelles. Encyclopédie entomologique, B II, Diptera 6: 11–40.

[B1000] Séguy E (1932b) Trois nouveaux *Stenopogon* marocains (Diptera : Asilidae). Bulletin de la Société entomologique de France 37(18): 259–259. 10.3406/bsef.1932.14482

[B1001] Séguy E (1932c) Spedizione scientifica all’ oasi di Cufra (Marzo-Luglio, 1931). Insectes diptères. Annali del Museo civico di storia naturale di Genova 55(1930–1931): 490–511.

[B1002] Séguy E (1934a) Diptères (Brachycères) (Muscidae acalypterae et Scatophagidae). Faune de France 28: III + 832 pp. [Pl. I–XXVII. Paris]

[B1003] Séguy E (1934b) Contribution à la connaissance des insectes diptères du Maroc. Terre et Vie 4(3): 162–163.

[B1004] Séguy E (1934c) Contribution à l’étude des mouches phytophages de l’Europe occidentale. II. Encyclopédie entomologique, B II, Diptera 7: 167–264.

[B1005] Séguy E (1934d) Diptères d’Afrique. Encyclopédie entomologique, Paris, Série B II, Diptera 7: 63–80.

[B1006] Séguy E (1934e) Diptères d’Espagne. Étude systématique basée principalement sur les collecions formées par le R.P. Longain Nava, S.J. Memorias de la Academia de Ciencias Exactas, Fisico-Quimicas y Naturales de Zaragoza 3: 1–54.

[B1007] Séguy E (1935a) Diptères du Maroc. Encyclopédie entomologique, Paris, Série B II, Diptera 8: 119–120.

[B1008] Séguy E (1935b) Une nouvelle espèce de Thérévide du Maroc. Encyclopédie entomologique Paris, Série B II, Diptera 8: 153–154.

[B1009] Séguy E (1936a) Un nouveau Conopidae Marocain et synopsis générique des Conopides de la région Holartique. In: Neuville H (Ed.) Livre jubilaire de M.Eugène-Louis Bouvier, membre de l’Institut, professeur honoraire au Muséum, 299–302. [379 pp]

[B1010] Séguy E (1936b) Un nouveau Muscide nuisible à l’orge au Maroc. Liste des Diptères vivant sur les *Hordeum*. Bulletin de la Société des sciences naturelles du Maroc 16: 3–5.

[B1011] Séguy E (1938) Étude sur les diptères recuellis par M.H. Lhote dans le Tassili des Ajjer (Sahara Touareg). Encyclopédie entomologique, Paris, Série B II, Diptera 9: 37–45.

[B1012] Séguy E (1940) Récoltes entomologiques de M.L. Berland à Villa Cisneros (Rio de Oro). Insectes Diptères. Bulletin du Muséum national d’Histoire naturelle, Paris (2), 12(5–7): 340–343. [31.xii.1940]

[B1013] Séguy E (1941a) Récoltes de R. Paulian et A. Villiers dans le Haut Atlas marocain, 1938 (xviie note). Diptères. Revue Française d’Entomologie, Paris 8: 25–33.

[B1014] Séguy E (1941b) Un *Syllegomydas* nouveau du nord de l’Afrique (Dipt., Mydaidae). Bulletin de la Société entomologique de France 46: 111–112. 10.3406/bsef.1941.15519

[B1015] Séguy E (1941c) Études sur les mouches parasites. 2. Calliphorides, calliphorines (suite), sarcophagines et rhinophorides de l’Europe occidentale et méridionale. Encyclopédie entomologique (Ser. A) 21: 1–436.

[B1016] Séguy E (1941d) Diptères recueillis par M.L. Berland dans le sud marocain. Annales de la Société entomologique de France 110(1–2): 1–23.

[B1017] Séguy E (1949a) Diptères de Sud-Marocain (Vallée du Draa) recueillis par M.L. Berland en 1947. Revue Française d’Entomologie, Paris 16: 152–161.

[B1018] Séguy E (1949b) Un *Pycnopogon* aberrant du Maroc (Dipt., Asilidae). Revue Française d’Entomologie, Paris 16(3): 36–87.

[B1019] Séguy E (1949c) Un *Cyrtosia* nouveau et synopsis des espèces méditerranéennes (Dipt., Bombyliidae). Revue Française d’Entomologie, Paris 16: 83–85.

[B1020] Séguy E (1952) Un nouveau *Stratioleptis* de Mandchourie. Revue Française d’Entomologie, 4^è^ trimestre 19: 243–244.

[B1021] Séguy E (1953a) Diptères du Maroc. Encyclopédie entomologique. Série B II. Memoires et notes. (1947–1953) 11: 77–92.

[B1022] Séguy E (1953b) Un nouveau Phytomyzidae marocain. Encyclopédie entomologique. Série B II. Memoires et notes 11: 72.

[B1023] Séguy E (1953c) Description d’un *Cryptochaetum* marocain. Encyclopédie entomologique. Série B II. Memoires et notes 11: 46.

[B1024] Séguy E (1957) Chloropides africains du muséum [Dipt.]. Revue Française d’Entomologie, Paris 24(3): 264–277.

[B1025] Séguy E (1961) Dipteres Syrphides de l’Europe occidentale. Mémoires du Muséum d’histoire naturelle. Nouvelle série, série A. Zoologie 23, 248 pp.

[B1026] Senevet G (1935) Les Anophèles de la France et de ses colonies. Encyclopédie entomologique, P. Lechevalier Editions, 361 pp.

[B1027] Senevet G Gaud J Millet A (1949) Validité de l’espèce *Culexmauritanicus* Callot, 1940. Archives de l’Institut Pasteur d’Algérie 27: 42–47.

[B1028] Senevet G Andarelli L (1954) Le genre *Aedes* en Afrique du Nord, I: Les larves. Archives d’Institut Pasteur d’Algérie 32: 310–351. 14350829

[B1029] Senevet G Andarelli L (1956) Les Anophèles de l’Afrique du Nord et du bassin méditerranéen. Encyclopédie entomologique 33. Lechevalier, Paris, 280 pp.

[B1030] Senevet G Adarelli L (1959a) I. Les Moustiques de l’Afrique du Nord et du bassin méditerranéen: les genres *Culex*, *Uranotaenia*, *Theobaldia*, *Orthopodomyia* et *Mansonia*. Encyclopédie entomologique 37. Lechevalier, Paris, 384 pp.

[B1031] Senevet G Andarelli L (1959b) Un nouveau caractère pour la diagnose des larves de *Culex*. Archives de l’Institut Pasteur d’Algérie 37: 447–461. 14444904

[B1032] Serra-Tosio B (1967) Taxonomie et Ecologie et biogéographie des *Diamesa* du groupe *latitarsis* (Diptera, Chironomidae). Travaux du Laboratoire d’Hydrobiologie et de Pisciculture, Université Grenoble 57–58: 65–91.

[B1033] Serra-Tosio B (1973) Ecologie et biogéographie des Diamesinae d’Europe (Diptera, Chironomidae). Travaux du Laboratoire d’Hydrobiologie, Grenoble 63: 5–175.

[B1034] Serra-Tosio B (1983) Données biogéographiques nouvelles sur les Diamesinae des montagnes d’Asie et d’Afrique. 10^è^ Congrès national des Sociétés savantes, Grenoble, Sciences, fascicule II: 257–258.

[B1035] Setyaningrum H Al Dhafer HM (2014) The Calliphoridae the blow flies (Diptera: Oestroidea) of Kingdom of Saudi Arabia. Egyptian Academic Journal of Biological Sciences 7(1): 49–139. 10.21608/eajbsa.2014.13203

[B1036] Shamshev IV (2018) A new species of *Empis* Linnaeus, 1758 (Diptera: Empididae) from Morocco, with a key to *Coptophlebia*-like species of the Mediterranean basin. Caucasian Entomological Bulletin 14 (Supplement): 35–39. 10.23885/18143326201814S35

[B1037] Shamshev IV Grootaert P Stark A (2005) Notes on a remarkable abdominal structure in some *Crossopalpus* Bigot species (DipteraHybotidae), with new records from Southeast Asia. Studia dipterologica 12(2): 331–336.

[B1038] Shoeibi B Karimpour Y (2010) Contributions to the knowledge of Asilidae (Diptera: Brachycera) from Azarbaijan provinces (Iran). Munis Entomology & Zoology 5: 957–963.

[B1039] Sicault G Messerlin A Lumeau J Fritz J (1935) Le Paludisme dans le Gharb. Bulletin de l’Institut d’Hygiène du Maroc 5: 5–91.

[B1040] Sidney C (2001) Additional information on Afrotropical Conopidae. Entomologist’s Monthly Magazine 137: 179–210.

[B1041] Singh S Baba ZA Abas Shah M Kumar R (2014) First record on incidence of Bean Seed Fly, *Deliaplatura* Meigen (Anthomyiidae: Diptera) in Autumn Sown Beans in Kashmir valley (India). Munis Entomology & Zoology 9(1): 586–587.

[B1042] Skuhravá M (1986) Family Cecidomyiidae. In: Soós A Papp L (Eds) Catalogue of Palaearctic Diptera.Vol. 4: Sciaridae – Anisopodidae. Akadémiai Kiadó, Budapest, 72–297. [441 pp]

[B1043] Skuhravá M (1995) A new gall midge species, *Etsuhoathuriferae* sp. n. (Diptera, Cecidomyiidae), from galls on *Juniperusthurifera* L. (Cupressaceae) in Spain. Zapateri, Revista aragonesa de Entomología 5: 135–146.

[B1044] Skuhravá M Skuhravý V Brewer JW (1984) Biology of gall midges. In: Ananthakrishnan TN (Ed.) Biology of Gall Insects.Oxford + IBH Publishing Company, New Delhi, Bombay, Calcutta, 169–222. [362 pp]

[B1045] Skuhravá M Blasco-Zumeta J Skuhravý V (1993) Gall midges (Diptera, Cecidomyiidae) of Aragon. A review of species found in the period 1890–1990 with new records for the Monegros region. Zapateri, Revista aragonesa de Entomología 3: 27–36.

[B1046] Skuhravá M Bayram S Çam H Tezcan S Can P (2005) Gall midges (Diptera: Cecidomyiidae) of Turkey. Türkiye Entomoloji Derneği 29(1): 17–34.

[B1047] Skuhravá M Skuhravý V Elsayed AK (2014a) Gall midges (Diptera: Cecidomyiidae) of Egypt: annotated list and zoogeographical analysis. Acta Societatis Zoologicae Bohemicae 78: 241–268.

[B1048] Skuhravá M Karimpour Y Hussein Sadeghi H Ali Gol A Joghataie M (2014b) Gall midges (Diptera: Cecidomyiidae) of Iran: annotated list and zoogeographical analysis. Acta Societatis Zoologicae Bohemicae 78: 269–301.

[B1049] Skuhravá M Skuhravý V Kettani K (2017) Gall midges (Diptera: Cecidomyiidae) of Morocco. Acta Societatis Zoologicae Bohemicae 81: 61–87.

[B1050] Smit T Vujić A (2007) The Palaearctic species of the genus *Psilota* Meigen (Diptera, Syrphidae) with the description of two new species. Studia dipterologica 14(2): 345–364.

[B1051] Soós Á (1984a) Family Platystomatidae. In: Soós Á, Papp L (Eds) Catalogue of Palaearctic Diptera. Vol. 9: Micropezidae – Agromyzidae. Akadémiai Kiadó, Budapest, 460 pp.

[B1052] Soós Á (1984b) Family Otitidae, pp. 45–59. In: Soós Á Papp L (Eds) Catalogue of Palaearctic Diptera.Vol. 9: Micropezidae – Agromyzidae. Akadémiai Kiadó, Budapest, 38–45. [460 pp]

[B1053] Speight MCD (2013) Species accounts of European Syrphidae (Diptera). Syrph the Net: the database of European Syrphidae Vol. 72, 316 pp. [Dublin]

[B1054] Speight MCD (2018) Species accounts of European Syrphidae. Syrph the Net, the database of European Syrphidae (Diptera) 103, 302 pp. [Syrph the Net publications, Dublin]

[B1055] Spencer KA (1967) Some Agromyzidae (Diptera) from Morocco. Entomologist´s Monthly Magazine 103: 126–130.

[B1056] Spencer KA (1973) Agromyzidae (Diptera) of economic importance. Series entomologica, Volume 9. W. Junk B.V., The Hague, xi + 418 pp. 10.1007/978-94-017-0683-4

[B1057] Staiger H Gloor H (1952) Mitosehemmung und Polyploidie durch einen Letalfaktor (lpl = letal-polyploid) bei *Drosophilahydei*. Chromosoma 5: 221–245. 10.1007/BF01271488 13067189

[B1058] Starý J (1971) A new palaearctic representative of the subgenus Protogonomyia Alexander (Diptera, Tipulidae). Acta Entomologica Bohemoeslovaca 68: 319–321.

[B1059] Starý J (2006) Hoplolabis (Parilisia) species related to H. (P.) punctigera (Lackschewitz, 1940) and H. (P.) spinosa (Nielsen, 1953) with the description of a new species (Diptera, Limoniidae) [Übersicht der mit Hoplolabis (P.) punctigera (Lackschewitz, 1940) und H. (P.) spinosa (Nielsen, 1953) verwandten Arten der Untergattung *Parilisia* nebst der Beschreibung einer neuen Spezies (Diptera, Limoniidae)]. Studia dipterologica 13: 115–125.

[B1060] Starý J (2009a) New records of Limoniidae, Pediciidae and Tipulidae (Diptera) from the Czech Republic and Slovakia. Folia faunistica Slovaca 14(13): 95–97.

[B1061] Starý J (2009b) West Palaearctic species of the genus *Eloeophila* (Diptera: Limoniidae). European Journal of Entomology 106: 425–440. 10.14411/eje.2009.054

[B1062] Starý J (2011) Descriptions and records of the Palaearctic *Molophilus* Curtis (Diptera, Limoniidae). Zootaxa 2999: 45–62. 10.11646/zootaxa.2999.1.5

[B1063] Starý J Freidberg A (2007) The Limoniidae of Israel (Diptera). Israel Journal of Entomology 37: 301–357.

[B1064] Starý J Oosterbroek P (2008) New records of West Palaearctic Limoniidae, Pediciidae and Cylindrotomidae (Diptera) from the collections of the Zoological Museum, Amsterdam. Zootaxa 1922: 1–20. 10.11646/zootaxa.1922.1.1

[B1065] Steenis van J (2000) The West-Palaearctic species of *Spilomyia* Meigen (Diptera, Syrphidae). Mitteilungen der schweizerischen entomologischen Gesellschaft 73: 143–168.

[B1066] Steenis van J Hauser M van Zuijen MP (2017) Review of the *Eumerusbarbarus* species group (Diptera: Syrphidae) from the western Mediterranean Basin. Bonn zoological Bulletin 66(2): 145–165.

[B1067] Steenis van J Ricarte A Vujić A Birtele D Speight MCD (2016) Revision of the West-Palaearctic species of the tribe Cerioidini (Diptera, Syrphidae). Zootaxa 4196(2): 151–209. 10.11646/zootaxa.4196.2.1 27988671

[B1068] Stefanescu C Askew RR Corbera J Shaw MR (2012) Parasitism and migration in southern Palaearctic populations of the painted lady butterfly, *Vanessacardui* (Lepidoptera: Nymphalidae). European Journal of Entomology 109: 85–94. 10.14411/eje.2012.011

[B1069] Stubbs A (2008) Some notes on the Biogeography of British Craneflies. In: Cranefly Recording Scheme Newsletter №16.

[B1070] Stuckenberg BR (1998) A revision of the Palaearctic species of *Lampromyia* Macquart (Diptera, Vermileonidae), with the description of a new Iberian species and a cladogram for the genus. Bonner zoologische Beiträge 48(1): 67–96.

[B1071] Stuke JH (2016) Taxonomic notes on Western Palaearctic Conopidae (Diptera). Zootaxa 4178(4): 521–534. 10.11646/zootaxa.4178.4.4 27811705

[B1072] Stuke JH Clements DK (2008) Revision of the *Myopatestacea* Species-Group in the Palaearctic Region (Diptera: Conopidae). Zootaxa 1713: 1–26.

[B1073] Stuke JH Kehlmaier C (2008) Westpaläarktische Conopidae (Insecta: Diptera) in der Sammlung des Museums für Tierkunde der staatlichen naturhistorischen Sammlungen Dresden. Faunistische Abhandlungen 26: 137–147.

[B1074] Stuke JH Schmid-Egger C (2015) Some new records of Conopidae (Diptera) from Morocco. Studia dipterologica 22(1): 56–58.

[B1075] Sturtevant AH (1921) Genetic studies on *Drosophilasimulans*. II. Sex-linked group of genes. Genetics 6: 43–64. 10.1093/genetics/6.1.43 17245955 PMC1200498

[B1076] Summer RW (1978) A record of *Crataerinahirundinis* from Morocco. Entomologist’s Monthly Magazine 114: 174.

[B1077] Sun X Marshall SA (2003) Systematics of *Phasia* Latreille (Diptera: Tachinidae). Zootaxa 276: 1–320. 10.11646/zootaxa.276.1.1

[B1078] Surcouf J (1921) Notes synonymiques sur le *Diachlorusmaroccanus* Bigot (Dipt: Tabanidae). Bulletin de la Société entomologique de France 9: 143–143. 10.3406/bsef.1921.26801

[B1079] Suwito A Toda MJ Takamori H Harada K Watabe H (2014) Revision of Asian species of the *Drosophilamelanica* species group (Diptera: Drosophilidae), with a description of a new species from Vietnam. Entomological Science 17: 75–85. 10.1111/ens.12028

[B1080] Szadziewski R Dominiak P (2006) News synonyms of European Ceratopogonidae (Diptera). Annales Zoologici 56(1): 139–146.

[B1081] Theischinger G (1977) Neue Taxa von *Lunatipula* Edwards aus der mediterranen Subregion der Palaearktis (Diptera, Tipulidae, *Tipula* Linnaeus). Beaufortia 26: 1–38.

[B1082] Theischinger G (1980) Neue Taxa von *Lunatipula* Edwards aus der mediterranen Subregion der Palaearktis (Diptera, Tipulidae, *Tipula* Linnaeus), III. Fortsetzung. Beaufortia 30: 17–29.

[B1083] Theodor O (1967) An illustrated catalogue of the Rothschild Collection of Nycteribiidae (Diptera) in the British Museum (Natural History) with keys and short descriptions for the identification of subfamilies, genera, species and subspecies. British Museum (Natural History) Publication, 665: 1–506.

[B1084] Theowald Br (1972) Die Tipuliden Algeriens (Diptera, Tipulidae). Entomologische berichten, Amsterdam 32: 3–5.

[B1085] Theowald Br (1973) 15. Tipulidae. In: Lindner E (Ed.) Die Fliegen der palaearktischen Region, 3(2), Lief.300: 321–404.

[B1086] Theowald Br (1980) 15. Tipulidae. In: Lindner E (Ed.) Die Fliegen der palaearktischen Region, 3(2), Lief.324: 437–538.

[B1087] Theowald Br (1984) Taxonomie, Phylogenie und Biogeographie der Untergattung Tipula (Tipula) Linnaeus, 1758 (Insecta, Diptera, Tipulidae). Tijdschrift voor Entomologie 127: 33–78.

[B1088] Theowald Br Oosterbroek P (1980) Zur Zoogeographie der westpalaearktischen Tipuliden, I. Die Tipuliden von Nordafrika (Diptera, Nematocera). Beaufortia 30: 179–192.

[B1089] Theowald Br Oosterbroek P (1983) Zur Zoogeographie der westpalaearktischen Tipuliden, III. Die Tipuliden der europaischen Tiefebenen (Diptera, Tipulidae). Bonner zoologische Beiträge 34: 371–394.

[B1090] Thomas A (1979) *Chrysopilustsacasi* n. sp., a new Rhagionidae from the Moroccan Upper Atlas (Diptera, Brachycera). Bulletin de la Société d’histoire naturelle de Toulouse 115(1–2): 136–139.

[B1091] Thomas A (1985) Athericidae d’Afrique du Nord. II. *Ibisiaamicorum* n. sp. (Diptera, Brachycera). Annales de Limnologie 21(1): 89–91. 10.1051/limn/1985009

[B1092] Thomas A Gagneur J Dakki M (1995) West paleaerctic Athericidae: the genus *Ibisia* Rondani, 1856. I: Rediscovery of *I.maroccana* (Séguy, 1930) at the locus typicus, and its ecology (Diptera: Brachycera: Orthorrhapha). Annales de la Société entomologique de France 31(1): 63–69.

[B1093] Thomas-Orillard M (1984) Modifications of mean ovariole number, fresh weight of adult females and developmental time in *Drosophilamelanogaster* induced by *Drosophila* C virus. Genetics 107: 635–644. 10.1093/genetics/107.4.635 17246225 PMC1202381

[B1094] Thompson FC (1998) Fruit Fly Expert Identification System and Systematic Information Database. Myia 9. Backhuys Publishers, 535 pp.

[B1095] Thompson WR (1950) A catalogue of the parasites and predators of insect pests. Section 1. Parasite Host Catalogue, Part 3. Parasites of the Hemiptera. Commonwealth Agricultural Bureaux, The Commonwealth Institute of Biological Control, Ottawa, Ontario, Canada, 155 pp.

[B1096] Timon-David J (1951) Contribution à la connaissance de la faune entomologique du Maroc, Diptera: Asilidae, Bombyliidae, Nemestrinidae et Syrphidae. Bulletin de la Société des sciences naturelles du Maroc, Rabat 31: 131–148.

[B1097] Tkoč M Roháček J (2014) Diversity, distribution and biology of Romanian flat-footed flies (Diptera, Opetiidae and Platypezidae) with taxonomic notes on *Callomyiasaibhira* Chandler. ZooKeys 459: 95–118. 10.3897/zookeys.459.8376 PMC4283634 25561855

[B1098] Tliqui H Bouazzaoui A Agoumi A (2007) Human auricular myiasis caused by *Wohlfahrtiamagnifica* (Diptera: Sarcophagidae): about three observations in Morocco. Bulletin de la Société de pathologie exotique 100(1): 61–64. 17402700

[B1099] Tmimi FZ Faraj C Bkhache M Mounaji K Failloux AB Sarih M (2018) Insecticide resistance and target site mutations (G119S ace-1 and L1014F kdr) of *Culexpipiens* in Morocco. Parasites & Vectors 11: e51. 10.1186/s13071-018-2625-y PMC5778619 29357900

[B1100] Tomasovic G (1997) Deux Asilidae nouveaux pour la faune marocaine récoltés par la mission «Calypso» (Diptera, Brachyera). Bulletin et Annales de la Société royale belge d’entomologie 133: 315–316.

[B1101] Tomasovic G (2001a) Révision de matériaux typiques du Museum für Naturkunde der Humboldt-Universität, Berlin, du genre *Eutolmus* Loew, 1848 (Diptera, Asilidae) ainsi que de *E.albicapillus* Janssens, 1968, et *E.albiventris* Villeneuve, 1920. Description de *E.wahisi* sp. n. Notes fauniques de Gembloux 42: 67–83.

[B1102] Tomasovic G (2001b) Une nouvelle espèce de *Dysmachus* Loew, 1860 d’Algérie (Diptera, Asilidae). Notes fauniques de Gembloux 42: 85–87.

[B1103] Tomasovic G (2003) Etude systématique et géographique sur les espèces espagnoles de *Machimus* Loew, 1849 (Diptera: Asilidae). Notes fauniques de Gembloux 50: 99–112.

[B1104] Tomasovic G (2006) Présence de *Leptogastercylindrica* sur l’Ile de Crète (Diptera: Asilidae). Notes fauniques de Gembloux 59(3): 155–156.

[B1105] Tomasovic G Weyer G Van (2008) Une espèce nouvelle d`*Amphisbetetus* du Maroc et distribution du genre. Bulletin de la Société royale belge d’entomologie 144: 67–70.

[B1106] Touabay M Aouad N Mathieu J (2002) Etude hydrobiologique d’un cours d’eau du Moyen-Atlas: l’oued Tizguit (Maroc). Annales de Limnologie 38(1): 65–80. 10.1051/limn/2002007

[B1107] Trari B (1991) Culicidae (Diptera): Catalogue raisonné des peuplements du Maroc et études typologiques de quelques gîtes du Gharb et de leurs communautés larvaires. Thèse de 3ème cycle, Faculté des Sciences, Université Mohamed V, Rabat, 209 pp.

[B1108] Trari B (2017) Les moustiques (Insectes, Diptères) du Maroc: Atlas de répartition et études épidémiologiques. Thèse de Doctorat d’État, Université Mohammed V, Rabat, 334 pp.

[B1109] Trari B Himmi O (1987) Biotypologie et répartition spatio-temporelle des Moustiques (Diptera, Culicidae) du Gharb (Maroc). C.E.A., Faculté des Sciences, Université Mohamed V, Rabat, 103 pp.

[B1110] Trari B Dakki M Himmi O El Agbani MA (2002) Les moustiques (Diptera, Culicidae) du Maroc. Revue bibliographique (1916–2001) et inventaire des espèces. Bulletin de la Société de pathologie exotique 95(4): 329–334. 14717054

[B1111] Trari B Fontenille D Agoumi A (2004a) Le paludisme à Larache: Recherche de l’infection plasmodiale chez les anophèles (Diptera: Culicidae) par une technique immuno-enzymatique. Intérêt de l’utilisation de la technique au Maroc. Animalis 3(1): 27–29.

[B1112] Trari B Harbach RE Himmi O Dakki M Agoumi A (2004b) An inventory of the mosquitoes of Morocco. I. Genus *Anopheles* (Diptera: Culicidae). European Mosquito Bulletin 18: 1–19.

[B1113] Trari B Carnevale P (2011) De la préélimination à l’élimination du paludisme au Maroc. Quels risques pour l’avenir? Malaria in Morocco: from pre-elimimation to elimination, what risks for the future? Bulletin de la Société de pathologie exotique et de ses filiales 104: 291–295. 10.1007/s13149-011-0156-2 21638201

[B1114] Trari B Dakki M (2017a) Caractérisation génétique du sous-groupe *Maculipennis* (DipteraCulicidae) au Maroc: un outil fondamental pour lutter contre le paludisme. Eastern Mediterranean Health Journal 23(12): 809–814. 10.26719/2017.23.12.809 29528090

[B1115] Trari B Dakki M (2017b) Atlas des moustiques (DipteraCulicidae) du Maroc. Travaux de l’Institut Scientifique, Rabat. Série Zoologie 51: 1–128.

[B1116] Trari B Dakki M Harbach RE (2017) An update checklist of the Culicidae (Diptera) of Morocco, with notes on species of historical and current medical importance. Journal of Vector Ecology 42(1): 94–104. 10.1111/jvec.12243 28504435

[B1117] Trotter A (1904) Di alcune galle del Marocco. Marcellia 3: 14–15.

[B1118] Tschorsnig HP (1991) New TachinidaeDiptera from Spain and Morocco. Stuttgarter Beitraege zur Naturkunde, Serie A (Biologie) 459: 1–8.

[B1119] Tschorsnig HP (1997) A New Genus and four New Species of Palaearctic Tachinidae (Diptera). Stuttgarter Beiträge zur Naturkunde, Serie A (Biologie) 555: 1–9.

[B1120] Tschorsnig HP Bläsius R (2001) Neue oder interessante Wirtsbefunde von Raupenfliegen (Diptera: Tachinidae) aus Glasflüglern (Lepidoptera: Sesiidae). Mitteilungen des entomologischen Vereins Stuttgart 36: 23–24.

[B1121] Tschorsnig HP Herting B (1994) Die Raupenfliegen (Diptera: Tachinidae) Mitteleuropas: Bestimmungstabellen und Angaben zur Verbreitung und Ökologie der einzelnen Arten. Stuttgarter Beiträge zur Naturkunde, Serie A (Biologie) 506: 1–170. [Online Authorized Version of English Translation by Rayner R., Raper C., Tschorsnig H.P. and Herting B. 1994. The tachinids (Diptera: Tachinidae) of central Europe: identification keys for the species and data on distribution and ecology] http://tachinidae.org.uk/site/downloads.php [accessed 12.06.2006]

[B1122] Tschorsnig HP Richter VA (1998) Family Tachinidae. In: Papp L Darvas B (Eds) Contributions to a Manual of Palaearctic Diptera, Science Herald, Budapest, Vol.3, 691–827. [880 pp]

[B1123] Tschorsnig HP Ziegler J Herting B (2003) Tachinid flies (Diptera: Tachinidae) from the Hautes-Alpes, France. Stuttgarter Beiträge zur Naturkunde, Serie A (Biologie) 656: 1–62.

[B1124] Turchetto M Villemant-Aït L Vanin S (2003) Two fly parasitoids collected during an entomo-forensic investigation: the widespread *Nasoniavitripennis* (HymenopteraPteromalidae) and the newly recorded *Tachinaephaguszealandicus* (HymenopteraEncyrtidae). Bollettino della Società entomologica italiana 135(2): 109–115.

[B1125] Üstüner T Hasbenli A (2013) Contributions to Family Stratiomyidae (Diptera) Fauna of Konya Province (Turkey). Journal of Selçuk University Natural and Applied Science 2(4): 40–55.

[B1126] Vaillant F (1950) Contribution à l’étude des Dolichopodidae d’Algérie (Diptères). Extrait du Bulletin de la Société d’histoire naturelle de l’Afrique du Nord 41: 35–40.

[B1127] Vaillant F (1952) Quelques Dolichopodidae de la zone paléarctique (Diptera). Institut royal des Sciences naturelles de Belgique 28(65): 1–15.

[B1128] Vaillant F (1954a) Une nouvelle contribution à l’étude des Thaumaleidae (Dipt.). L’entomologiste 10: 94–97.

[B1129] Vaillant F (1954b) Note préliminaire sur la faune madicole (hygropétrique s. I.) de France, de Corse et d’Afrique du Nord. L’entomologiste 10: 37–42.

[B1130] Vaillant F (1955a) Les Dolichopodidae des rivages maritimes en Afrique du Nord (Diptères). Bulletin de la Société des Histoires naturelles 46: 303–308.

[B1131] Vaillant F (1955b) Recherches sur la faune madicole de France, de Corse et d’Afrique du Nord. Thèse, Faculté des Sciences, Université Paris, A-2744, 258 pp.

[B1132] Vaillant F (1956a) Contribution à l’étude des Diptères Empididae du Grand Atlas Marocain. I. Hemerodrominae et Atalantinae. Société des sciences naturelles et physiques du Maroc Tome XXXVI, 1^er^ trimestre: 61–71.

[B1133] Vaillant F (1956b) Recherches sur la faune madicole de France, de Corse et d’Afrique du Nord. Mémoires du Muséum national d’histoire naturelle. Nouvelle série, série A. Zoologie Tome XI, 258 pp.

[B1134] Vaillant F (1956c) Les Blepharoceridae d’Afrique du Nord. Bulletin de la Société entomologique de France 61: 113–120. 10.3406/bsef.1956.18843

[B1135] Vaillant F (1959) Quelques Dixidae paléarctiques et les habitats de leurs larves. Bulletin de la Société entomologique de France 64: 178–186. 10.3406/bsef.1959.20487

[B1136] Vaillant F (1964) Revision des EmpididaeHemerodrominae de France, d’Espagne et d’Afrique du Nord. Annales de la Société entomologique de France 133: 143–170.

[B1137] Vaillant F (1965) Quelques Dixidae paléarctiques nouveaux ou mal connus (Diptera). Annales de la Société entomologique de France I(4): 789–795.

[B1138] Vaillant F (1978) 9d. Psychodidae – Psychodinae. In: Lindner E (Ed.) Die Fliegen der palaearktischen Region Vols.317, 207–238.

[B1139] Vaillant F (1989) Contribution à l’étude des Diptères Lonchopteridae d’Europe et d’Afrique du Nord. Bulletin de la Société Vaudoise des Sciences Naturelles 79(3): 209–229.

[B1140] Vaillant F Moubayed-Breil J (1987) Cinq espèces nouvelles de Diptères PsychodidaePsychodinae du Liban. Annales de Limnologie 23(2): 121–127. 10.1051/limn/1987008

[B1141] Vaillant F Gagneur J (1998) The DipteraEmpididaeHemerodromiinae from western Algeria and the Middle Atlas of Morocco. Annales de la Société entomologique (N.S.) 34(4): 365–384.

[B1142] Vaillant F Moubayed-Breil J (1998) Notes sur les Diptères EmpididaeHemerodromiinae du Liban. Revue française d’Entomologie (N.S.) 20(1–2): 51–60.

[B1143] Vala JC (1989) Diptères Sciomyzidae Euro-méditerranéens. Faune de France. France et Régions limitrophes. № 72. Fédération française des Sociétés des Sciences naturelles Paris, 300 pp. [124 figs., 26 maps, 9 pls]

[B1144] Vala JC Reidenbach JM (1982) Description du néallotype mâle de *Euthyceraalgira* (Macquart, 1849) et redescription du lectotype (Dipt., Sciomyzidae). Bulletin de la Société entomologique de France 87: 34–38. 10.3406/bsef.1982.18000

[B1145] Vala JC Ghamizi M (1991) Sciomyzidae du Maroc (Diptera). L’entomologiste 47(4): 205–208.

[B1146] Valdés B Rejdali M Achhal el Kadmiri A Jury JL Montserrat JM (2002) Catalogue des plantes vasculaires du nord du Maroc, incluant des clés d’identification. Consejo superior de investigacione scientíficas, 1007 pp. [Book in Spanish]

[B1147] Vaňhara J Rozkošný R (1997) Czech and Slovak Dipterological Literature 1986–1995. Folia Facultatis Scientiarum Naturalium. Universitatis Masarykianae Brunensis, Biologia 95: 1–177.

[B1148] Vayssière P (1920) Les insectes nuisibles aux cultures du Maroc (2^ème^ Note). Bulletin de la Société entomologique de France 25(15): 256–259. 10.3406/bsef.1920.26676

[B1149] Venter GJ Graham SD Hamblin C (2000) African horse sickness epidemiology: vector competence of South African *Culicoides* species for virus serotypes 3, 5 and 8. Medical and Veterinary Entomology 14(3): 245–250. 10.1046/j.1365-2915.2000.00245.x 11016430

[B1150] Verbeke J (1964) Contribution à l’étude des diptères malacophages. II. Données nouvelles sur la taxonomie et la répartition géographique des Sciomyzidae paléarctiques. Institut royal des sciences naturelles de Belgique Tome XL, № 8, 1–27.

[B1151] Vermoolen D (1983) The Tipula (Acutipula) maxima group (Insecta, Diptera, Tipulidae). 1. Taxonomy and Distribution. Bijdragen tot de Dierkunde 53: 49–81. 10.1163/26660644-05301004

[B1152] Verves Yu-G (2003) A preliminary list of species of Calliphoridae and Sarcophagidae (Diptera) of the Republic of Seychelles. Phelsuma 11 (supplement A).

[B1153] Verves Yu-G (2004) A review of the «*Onesia*» generic group (Diptera: Calliphoridae). Part 2. The species of genus *Bellardia* Robineau-Desvoidy. Far Eastern Entomologist 135: 1–23.

[B1154] Verves Yu-G (2019) Review of Sarcophagidae (Diptera) of North African countries with new faunistic data from Algeria. Halteres 10: 62–74. 10.5281/zenodo.3594368

[B1155] Vialatte C (1922) Le paludisme au Maroc: épidémiologie-prophylaxie. Archives de l’Institut Pasteur d’Afrique du Nord II(4): 596–621.

[B1156] Vialatte C (1923) Contribution à la recherche de l’aire de distribution de *Stegomyiafasciata*. Son existence à Marrakech. Archives de l’Institut Pasteur d’Algérie 1: 688–690.

[B1157] Viamonte JMR (1950) Los anofelinos de España y de la zona española del Protectorado de Marruecos. Su relación con la difusión del paludismo. (Tesis Doctoral). Revista de Sanidad e Higiene Pública 24: 213–295. 15441539

[B1158] Viamonte JMR Ramirez A (1945) Nota previa sobre el anofelismo de la zona del Protectrado espanol de Marruecos. Revista de Sanidad e Higiene Pública 10: 669–674. 21013095

[B1159] Viamonte JMR Ramirez A (1946) Culicinos de la zona espagnola de Marruecos. Revista de Sanidad e Higiene Pública 20: 449–455.

[B1160] Vidal JC Aboussaid H Oufdou K El Messoussi S Castañera P González-Cabrera J (2008) Characterization of *Bacillusthuringiensis* strain collections from Spain and Morocco ecosystems and evaluation of their insecticidal activity against *Ceratitiscapitata* (Diptera: Tephritidae). First Meeting of TEAM, Palma de Mallorca, 7–8 April.

[B1161] Vikhrev NE (2011a) Review of the Palaearctic members of the *Lispetentaculata* species-group (Diptera, Muscidae): revised key, synonymy and notes on ecology. ZooKeys 84: 59–70. 10.3897/zookeys.84.819 PMC3088069 21594166

[B1162] Vikhrev NE (2011b) Taxonomic notes on the *Lispeleucospila* species-group (Diptera: Muscidae). Russian entomological Journal 20(2): 215–218. 10.15298/rusentj.20.2.14

[B1163] Vikhrev NE (2012a) Notes on taxonomy of *Lispe* Latreille (Diptera: Muscidae). Russian entomological Journal 21(1): 107–112. 10.15298/rusentj.21.1.14

[B1164] Vikhrev NE (2012b) Revision of the *Lispelongicollis*-group (Diptera, Muscidae). ZooKeys 235: 23–39. 10.3897/zookeys.235.3306 PMC3496917 23226961

[B1165] Vikhrev NE (2012c) Four new species of *Lispe* Latreille, 1796 (Diptera: Muscidae) with taxonomic notes on related species. Russian entomological Journal 21(4): 423–433. 10.15298/rusentj.21.4.08

[B1166] Vikhrev NE Ge YQ Zhang D (2016) On taxonomy of the *Lispecaesia*-group (Diptera: Muscidae). Russian entomological Journal 25(4): 407–410. 10.15298/rusentj.25.4.10

[B1167] Vikhrev NE Erofeeva EA (2018) Review of the *Phaoniapallida* group (Diptera: Muscidae). Russian entomological Journal 27(3): 315–322. 10.15298/rusentj.27.3.14

[B1168] Villeneuve J (1932) Description de Myodaires supérieurs du Nord Africain (Dipt., Larvaevoridae). Bulletin de la Société entomologique de France 19: 284–286. 10.3406/bsef.1932.14493

[B1169] Villeneuve J (1933) A propos de deux diptères inédits du Maroc. Bulletin de la Société entomologique de France 38: 102–104. 10.3406/bsef.1933.14552

[B1170] Villeneuve J (1941) Myodaires supérieurs nouveaux (Dipt.). I. Afrique du Nord. Bulletin de la Société entomologique de France 8: 122–126. 10.3406/bsef.1941.15524

[B1171] Vitte B (1988) Etude des Diptères Ephydrides du Maroc. II. Les Ephydrides des lacs et des ruisseaux du nord du Moyen-Atlas marocain (Diptera, Brachycera). Nouvelle Revue d’Entomologie (N.S.) 5(4): 389–395.

[B1172] Vitte B (1989) Etude des Diptères Ephydrides du Maroc, II. Deux nouvelles espèces du genre *Hydrellia* (Diptera, Brachycera). Nouvelle Revue d’Entomologie (N.S.) 6(3): 259–263.

[B1173] Vitte B (1991) Les Ephydrides du Rif et de la Plaine au nord de Casablanca (Diptera, Brachycera); description d’une nouvelle espèce. Bollettino del Museo civico di Storia naturale di Venezia 40: 21–36.

[B1174] Vitte B (1992) Une nouvelle espèece du genre *Allotrichoma* Becker (Diptera, Brachycera). Étude des Diptères Ephydrides du Maroc, IV. Nouvelle Revue d’Entomologie (N.S.) 9(3): 255–258.

[B1175] Vujić A Marcos-Garcia MA Sarıbıyık S Ricarte A (2011) New data on the *Merodon* Meigen, 1803 fauna (Diptera: Syrphidae) of Turkey including description of a new species and changes in the nomenclatural status of several taxa. Annales de la Société entomologique de France (N.S.) 47(1–2): 78–88. 10.1080/00379271.2011.10697699

[B1176] Vujić A Radenković S Likov L (2018) Revision of the Palaearctic species of the *Merodondesuturinus* group (Diptera, Syrphidae). ZooKeys 771: 105–138. 10.3897/zookeys.771.20481 PMC6043631 30008578

[B1177] Vujić A Likov L Radenković S Tubić NK Djan N Šebić A Pérez-Bañón C Barkalov A Hayat R Rojo S Andrić A Ståhls G (2020a) Revision of the *Merodonserrulatus* group (Diptera, Syrphidae). ZooKeys 909: 79–158. 10.3897/zookeys.909.46838 32089636 PMC7015954

[B1178] Vujić A Radenković S Likov L Andrić A Janković M Ačanski J Popov G de Courcy Williams M Šašić Zorić L Djan M. (2020b) Conflict and congruence between morphological and molecular data: revision of the *Merodonconstans* group (Diptera: Syrphidae). Invertebrate Systematics 34: 406–448. 10.1071/IS19047

[B1179] Vujić A Radenković S Likov L Veselić S (2021a) Taxonomic complexity in the genus *Merodon* Meigen, 1803 (Diptera, Syrphidae). ZooKeys 1031: 85–124. 10.3897/zookeys.1031.62125 33958908 PMC8060246

[B1180] Vujić A Tot T Andrić A Ačanski J Šašić Zorić L Pérez-Bañón C Aracil A Veselić S Arok M Mengual X van Eck A Rojo S Radenković S (2021b) Review of the *Merodonnatans* group with description of a new species, a key to the adults of known species of the *natans* lineageand first descriptions of some preimaginal stages. Arthropod Systematics & Phylogeny 79: 343–378. 10.3897/asp.79.e65861

[B1181] Vujić A Speight MCD de Courcy Williams M Rojo S Ståhls G Radenković S Likov L Miličić M Pérez-Bañón C Falk S Petanidou T (2020c) Atlas of the hoverflies of Greece (Diptera: Syrphidae). Brill, Leiden, 384 pp. 10.1163/9789004334670

[B1182] Wagner R (1990) Family Psychodidae. In: Soós Á, Papp L (Eds) Catalogue of Palaearctic Dip­tera. 2. Psychodidae – Chironomidae. Akadémiai Kiadó, Budapest, 499 pp.

[B1183] Walker F (1848) List of the specimens of Dipterous insects. The Collection of British Museum (Natural History). Department of Zoology. Edited by Gray John Edward Volume 1, Subject: Diptera.

[B1184] Walker F (1849) List of the specimens of Dipterous insects of the British Museum. London 4: 689–1172.

[B1185] Weinberg M Bächli G (2002) New records of *Acrocerinasanguinea* (Meigen) and some other Acroceridae species (Diptera). Mitteilungen der schweizerischen entomologischen Gesell­schaft 75: 113–118.

[B1186] Weinberg M Blasco-Zumeta J (2004) Robber flies (Diptera, Asilidae) of *Juniperusthurifera* L. forest of los Monegros region (Zaragosa, Spain). Lucas Mallada 11: 245–260.

[B1187] Werner F (1938) Ergebnisse einer zoologischen Forschungsreise nach Marokko unternommen 1930 mit Unterstützung der Akademie der Wissenschaften in Wien von Franz Werner und Richard Ebner. VII. Insekten, Arachnoideen und Crustaceen. Sitzungsberichte der kaiserlichen Akademie der Wissenschaften in Wien 147(1): 111–134.

[B1188] White IM (1989) A new species of *Terellia* Robineau-Desvoidy associated with *Cen­taurea solstitialis* L. and a revision of the *Terelliavirens* (Loew) species group (Dip­tera: Tephritidae). Entomologist’s Monthly Magazine 125: 53–62.

[B1189] White IM Korneyev VA (1989) A revision of the Western Palaearctic species of *Urophora* Rob­ineau-Desvoidy (DipteraTephritidae). Systematic Entomology 14: 327–374. 10.1111/j.1365-3113.1989.tb00289.x 32327873 PMC7169537

[B1190] White IM Marquardt K (1989) A revision of the genus *Chaetorellia* Hendel (Diptera: Tephritidae) including a new species associated with spotted knapweed, *Centaureamaculosa* Lam. (Asteraceae). Bulletin of Entomological Research 79: 453–487. 10.1017/S0007485300018459

[B1191] Whitmore D (2009) A review of the Sarcophaga (Heteronychia) (Diptera: Sarcophagidae) of Sardinia. Zootaxa 2318: 566–588. 10.11646/zootaxa.2318.1.24

[B1192] Whitmore D (2011) New taxonomic and nomenclatural data on Sarcophaga (Heteronychia) (Diptera: Sarcophagidae), with description of six new species. Zootaxa 2778: 1–57.

[B1193] Whitmore D Pape T Cerretti P (2013) Phylogeny of *Heteronychia*: the largest lineage of *Sarcophaga* (Diptera: Sarcophagidae). Zoological Journal of the Linnean Society 169(3): 604–639. 10.1111/zoj.12070

[B1194] Wiedemann CRW (1824) Munus rectoris in Academia Christiana Albertina aditurus analecta entomologica ex Museo Regio Havniensis maxime congesta profert iconibusque illustrat. Kiel: Regio typographico scholarum, 60 pp., 1 plate. 10.5962/bhl.title.77322

[B1195] Wiedemann CRW (1830) Aussereuropäische zweiflügelige Insekten. Als Fortsetzung des Meigenschen Werkes. Zweither Teil. Hamm, Schulzische Buchhandlung XII + 684 pp., Tafeln VII–IX, Xa–Xb.

[B1196] Wiegmann BM Trautwein MD Winkler IS Barr NB Kim JW Lambkin C Bertone MA Cassel BK Bayless KM Heimberg AM Wheeler BM Peterson KJ Pape T Sinclair BJ Skevington JH Blagoderov V Caravas J Kutty SN Schmidt-Ott U Kampmeier GE Thompson FC Grimaldi DA Beckenbach AT Courtney GW Friedrich M Meier R Yeates DK (2011) Episodic radiations in the fly tree of life. Proceedings of the National Academy of Sciences United States 108: 5690–5695. 10.1073/pnas.1012675108 PMC3078341 21402926

[B1197] Wilson AJ Mellor PS (2009) Bluetongue in Europe: past, present and future. Philosophical Transactions of the Royal Society of London B: Biological Sciences 364: 2669–2681. 10.1098/rstb.2009.0091 19687037 PMC2865089

[B1198] Winterton SL Hauser M Haitham BM Badrawy HBM (2012) A Remarkable new genus of *Stiletto* flies from Egypt, with a key to Palaearctic genera of Phycinae (Diptera, Therevidae). ZooKeys 184: 35–45. 10.3897/zookeys.184.2759 PMC3332013 22573950

[B1199] Wolff M Nihei SS De Carvalho CJB [Eds] (2016) Catalogue of Diptera of Colombia. Zootaxa 4122, 949 pp. 10.11646/zootaxa.4122.1.1 27395249

[B1200] Wood JH (1912) Notes on British *Phora* (corrections and additions). Entomologist’s Monthly Magazine 48: 94–99, 166–181.

[B1201] Woodley NE (2001) A World Catalog of the Stratiomyidae (Insecta: Diptera). Myia 11: 1–473. [Backhuys Publishers, Leiden]

[B1202] Woźnica AJ (2011) Taxonomic notes on the genus *Gymnomus* Loew, with a description of a new species from Morocco (Diptera: Heleomyzidae). Polish Journal of Entomology 80(3): 579–586. 10.2478/v10200-011-0045-9

[B1203] Wülker W (1959) Zur Kenntnis der Gattung *Psectrocladius* Kieffer (Diptera, Chironomidae). Individuelle Variabilität, Grenzen und Möglichkeiten der Artentrennung, Ökologie und Verbreitung. Archiv für Hydrobiologie Supplement 24: 1–66.

[B1204] Yang D Zhang KY Yao G Zhang JH (2007) World catalogue of Empididae (Insecta: Diptera). China Agricultural University Press, Beijing, 599 pp.

[B1205] Yaran M Kütük M (2012) The fruit flies (Diptera: Tephritidae) fauna of Gaziantep province, Turkey. Munis Entomology & Zoology 7(2): 957–969.

[B1206] Yassin A David JR (2010) Revision of the afrotropical species of *Zaprionus* (Diptera, Drosophilidae), with descriptions of two new species and notes on internal reproductive structures and immature stages. ZooKeys (51): 33–72. 10.3897/zookeys.51.380 PMC3088026 21594121

[B1207] Yassin A Orgogozo V (2013) Coevolution between male and female genitalia in the *Drosophilamelanogaster* species subgroup. PLoS ONE 8(2): e57158. [12 pp] 10.1371/journal.pone.0057158 PMC3581563 23451172

[B1208] Yazough A Klein O (1999) Problème et gestion de l’*Orobanche* au Maroc. In: Kroschel J Abderabihi M Betz H (Eds) Advances in Parasitic Weed Control at On–farm Level.Vol. II. Joint Action to Control Orobanche in the WANA Region, 3–16. [IX + 347 pp]

[B1209] Yimlahi D Üstüner T Zinebi S Belqat B (2017) New records of the soldier flies of Morocco with a bibliographical inventory of the North African fauna (Diptera, Stratiomyidae). ZooKeys 709: 87–125. 10.3897/zookeys.709.13364 PMC5674172 29118638

[B1210] Zaitzev VF (1984) Family Ulidiidae. In: Soós Á Papp L (Eds) Catalogue of Palaearctic Diptera.Vol. 9: Micropezidae – Agromyzidae. Akadémiai Kiadó, Budapest, 59–66. [460 pp]

[B1211] Zaitzev VF (1999) On the fauna of Flies of the family Bombyliidae (Diptera) of Israel V Entomological Review 79(6): 640–653. [Originally published in Russian. In: Entomologicheskoe Obozrenie 78(1): 703–718]

[B1212] Zaitzev VF (2003) A New Genus and New Species of the Family Bombyliidae (Diptera) from the South-western Palaearctic Region. Entomological Review 83(5): 599–605. [Originally published in Russian. In: Entomologicheskoe Obozrenie 82(4): 909–916]

[B1213] Zaitzev VF (2005) New species of Flies of the family Bombyliidae (Diptera) from the Palaearctic Region. Entomological Review 85(6): 702–706. [Originally published in Russian. In: Entomologicheskoe Obozrenie 84(3): 767–772]

[B1214] Zaitzev VF (2006) New Species of the Genus *Chalcochiton* Loew (Diptera, Bombyliidae) from Spain and Morocco. Entomological Review 86(6): 728–732. [Originally published in Russian. In: Entomologicheskoe Obozrenie 85(3): 680–685] 10.1134/S0013873806060121

[B1215] Zaitzev VF (2007) Contributions to the Palaearctic Fauna of the Dipteran Families Bombyliidae and Mythicomyiidae (Diptera) I. Entomological Review 87(2): 159–173. [Originally published in Russian. In: Entomologicheskoe Obozrenie 86(1): 83–99] 10.1134/S0013873807020054

[B1216] Zaitzev VF (2008) Contributions to the Palaearctic Fauna of the Dipteran Families Bombyliidae and Mythicomyiidae (Diptera): II. Entomological Review 88(2): 186–198. [Originally published in Russian. In: Entomologicheskoe Obozrenie 87(1): 74–88] 10.1134/S0013873808020061

[B1217] Zarrouk A Kahime K Boussaa S Belqat B (2016) Ecological and epidemiological status of species of the *Phlebotomusperniciosus* complex (Diptera: Psychodidae, Phlebotominae) in Morocco. Parasitology Research 115(3): 1045–1051. 10.1007/s00436-015-4833-0 [Epub 2015 Nov 23] 26593735

[B1218] Zatwarnicki T (1987) New synonyms and records of Palaearctic *Scatophila* (Diptera, Ephydridae). Polskie pismo entomologiczne 57(2): 277–298.

[B1219] Zatwarnicki T (1988) Materials to the knowledge of the genus *Hydrellia* Robineau-Desvoidy (Diptera, Ephydridae). Polskie pismo entomologiczne 58(3): 587–634.

[B1220] Zatwarnicki T (1991) Changes in nomenclature and synonymies of some genera and species of Ephydridae (Diptera). Deutsche entomologische Zeitschrift (N.F.) 39(4–5): 295–333. 10.1002/mmnd.4800380403

[B1221] Zatwarnicki T (2018) Solving the puzzle of taxonomic position of the *petroleum* fly by resurrection of *Diasemocera* Bezzi from *Psilopa* Fallén (Diptera: Ephydridae) with proposed specific and generic synonymies. Annales Zoologici (Warszawa) 68(3): 527–552. 10.3161/00034541ANZ2018.68.3.012

[B1222] Zatwarnicki T Mathis WN (2011) Heterogeneity in shore flies – the case of *Glenanthe* Haliday (Diptera: Ephydridae) in the Old World. Annales de la Société entomologique de France (N.S.) 47(3–4): 418–443. 10.1080/00379271.2011.10697735

[B1223] Zetterstedt JW (1848) Diptera Scandinaviae disposita et descripta. Lundae 7: 2581–2934.

[B1224] Ziegler J (2011) First records and other interesting finds of Tachinidae from Israel and adjacent areas. In: O’Hara J (Ed.) The Tachinid Times 24: 7–11.

[B1225] Ziegler J (2012) Rezente Arealerweiterungen bei Wanzenfliegen (Diptera: Tachinidae, Phasiinae) in Nordostdeutschland und eine Übersicht zur Gesamtverbreitung von fünf Arten. Studia dipterologica 18 [2011]: 29–54.

[B1226] Zimina LV (1975) Conopidae (Diptera) of the fauna of the USSR. Genus *Sicus* Scop., 1763. Entomologicheskoe Obozrenie 54: 180–185.

[B1227] Zimsen E (1964) The type material of I.C. Fabricius. Munksgaard, Copenhagen, 656 pp.

[B1228] Zumpt F (1956) 64i. Calliphorinae. In: Lindner E (Ed.) Die Fliegen der palaearktischen Region 11: 1–140.

[B1229] Zuska J Pont A (1994) Family Sepsidae. In: Soós Á Papp L (Eds) Catalogue of Palaearctic Diptera Vol.9. Akadémiai Kiadó, Budapest, 154–167.

[B1230] Zwick P (2013) *Liponeurarifincola* spec. nov., another new net-winged midge (Diptera: Blephariceridae) from North Africa. Studia dipterologica 20(2): 332–334.

